# Complex Polyheterocycles and the Stereochemical Reassignment of Pileamartine A via Aza-Heck Triggered Aryl C–H Functionalization Cascades

**DOI:** 10.1021/jacs.1c08615

**Published:** 2021-09-21

**Authors:** Benjamin T. Jones, Javier García-Cárceles, Lewis Caiger, Ian R. Hazelden, Richard J. Lewis, Thomas Langer, John F. Bower

**Affiliations:** † School of Chemistry, University of Bristol, Bristol, BS8 1TS, United Kingdom; ‡ Department of Medicinal Chemistry, Research and Early Development, Respiratory and Immunology, BioPharmaceuticals R&D, AstraZeneca, SE 43183 Mölndal, Sweden; § Chemical Development, Pharmaceutical Technology & Development, Operations, Astra Zeneca, Charter Way, Macclesfield, SK10 2NA, United Kingdom; ¥ Department of Chemistry, University of Liverpool, Crown Street, Liverpool, L69 7ZD, United Kingdom

## Abstract

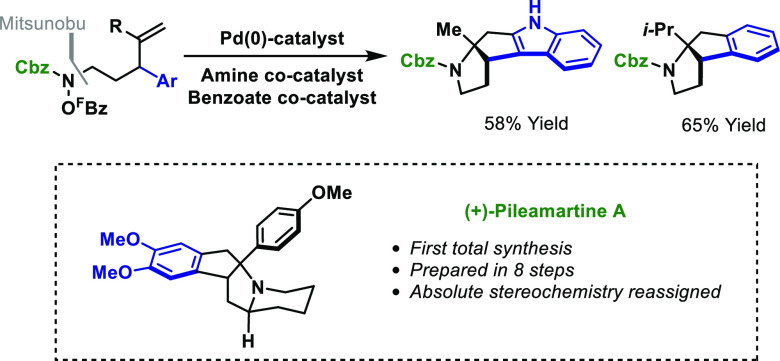

Structurally complex benzo- and spiro-fused N-polyheterocycles can be accessed via intramolecular Pd(0)-catalyzed alkene 1,2-aminoarylation reactions. The method uses *N*-(pentafluorobenzoyloxy)carbamates as the initiating motif, and this allows aza-Heck-type alkene amino-palladation in advance of C–H palladation of the aromatic component. The chemistry is showcased in the first total synthesis of the complex alkaloid (+)-pileamartine A, which has resulted in the reassignment of its absolute stereochemistry.

Pd-catalyzed cascade reactions are widely used for the rapid assembly of structurally complex ring systems, especially within the context of total synthesis.^1^ A valuable framework for accessing complex N-polyheterocycles resides in intramolecular Pd-catalyzed alkene aminocarbonations, where C–H palladation is used to install the new C–C bond (Scheme 1A). Building upon Hegedus’ seminal report,^2a^ Yang and co-workers have developed several oxidative 1,2-aminocarbonation processes,^2b^ including variants that involve aryl C–H palladation (Scheme 1B).^3a,3b^ Enantioselective 1,2-aminoarylations of this type have been reported by Liu and co-workers.^3c^ Mechanistically distinct processes that exploit external (hetero)aryl C–H units have been developed by the groups of Michael^4a,4b^ and Sigman.^4c^ External 1,3-dienes undergo 1,2-aminoarylation via the intermediacy of Pd(II)-π-allyls, as reported by Lloyd-Jones, Booker-Milburn, and co-workers.^5,6^

**Scheme 1:**
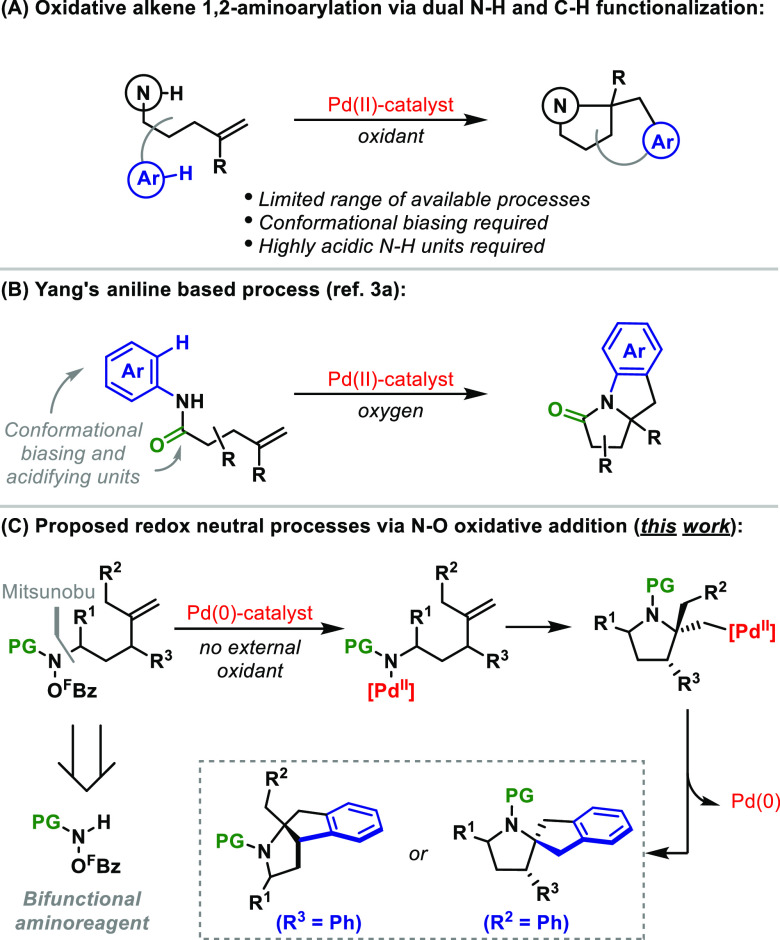
Introduction

A key feature of the processes in Scheme 1B is that they usually require (a) relatively acidic NH units and (b) a high degree of conformational bias.^3^ The former presumably aids NH palladation,^7^ whereas the latter enhances the efficiency of one or both of the cyclization steps. Consequently, these oxidative processes offer very specific scope, such that selective examples require systems where the aromatic unit is attached directly to the amide NH unit (i.e., anilide-based systems). Nevertheless, the value of these cascade reactions is clear, and so the development of complementary or broader scope alternatives is a pressing and worthwhile objective. To this end, we considered whether redox neutral processes might be developed that exploit a N–O bond as an internal oxidant (Scheme 1C). In this design, N–H palladation is replaced by N–O oxidative addition, which alleviates, at least in part, the requirement for an acidifying functionality. Further, by using an internal oxidant, substrate binding and catalyst oxidation are united (cf. Scheme 1B). Accordingly, catalysis should be more robust, and less conformationally biased cyclizations might be achievable. Indeed, we have recently shown that activated *N*-hydroxy-sulfonamides^8a^ and -carbamates^8b,8c^ are viable substrates for aza-Heck cyclizations and that these methods offer enhanced scope versus oxidative alternatives.^9^ Watson and co-workers have outlined similar benefits using other types of N–O-based functionality as the initiating unit.^8d−8f^ In this report, we describe the first examples of processes where aza-Heck cyclization of activated *N*-hydroxycarbamates is used to trigger intramolecular aryl C–H functionalization cascades (Scheme 1C).^10^ The method provides a new and powerful framework for the 1,2-aminoarylation of alkenes and, in so doing, provides direct access to alkaloid-like scaffolds. This is demonstrated through the first total synthesis of the complex alkaloid pileamartine A, which has led to the stereochemical reassignment of this natural product.^11^

The envisaged cascade amino-arylation processes are mechanistically complex, and key considerations are outlined in Scheme 2A. Following N–O oxidative addition to **I**, efficient cyclization requires *dissociation* of pentafluorobenzoate to access cationic aza-Pd species **I′**, as supported by earlier studies.^8a,8b^ For substrates of type **1**, aza-palladation is expected to be selective for ***trans-*****II**; this diastereoselectivity has been observed for processes involving external aryl boronic ester nucleophiles (Scheme 2A, gray box). However, aryl C–H palladation from ***trans*****-II** is expected to be demanding due to geometric constraints. Consequently, the establishment of a Curtin–Hammett scenario is required wherein reversible aza-palladation allows access to ***cis*****-II**, which, although thermodynamically disfavored, is geometrically set up for aryl C–H palladation. Although not exploited as a design tactic, reversible alkene aza-palladation has been established in other contexts^12^ and requires a free coordination site, which can, in principle, be provided by maintaining a cationic Pd center.^13^ At the stage of ***cis*****-II**, efficient C–H palladation likely requires *association* of a carboxylate ligand to facilitate concerted metalation deprotonation (CMD).^14^ One option would be for the Pd center to reengage the pentafluorobenzoate leaving group; however, this species is expected to be suboptimal because it dissociates readily and its carbonyl unit is not especially basic. To address this, we considered evaluating external benzoate additives (ArCO_2_M) to improve CMD efficiency. A beneficial aspect of this strategy is that it releases ArCO_2_H, which can then trigger Et_3_N-mediated protodecarboxylation of the pentafluorobenzoate leaving group.^8a,15^ This (a) drives equilibrium access to the requisite cationic aza-Pd-intermediate **I′** and (b) allows the benzoate additive to be used catalytically. The latter is important because stoichiometric quantities of strongly coordinating benzoate additives are expected to inhibit alkene aza-palladation by preventing access to cationic intermediate **I′**.

**Scheme 2:**
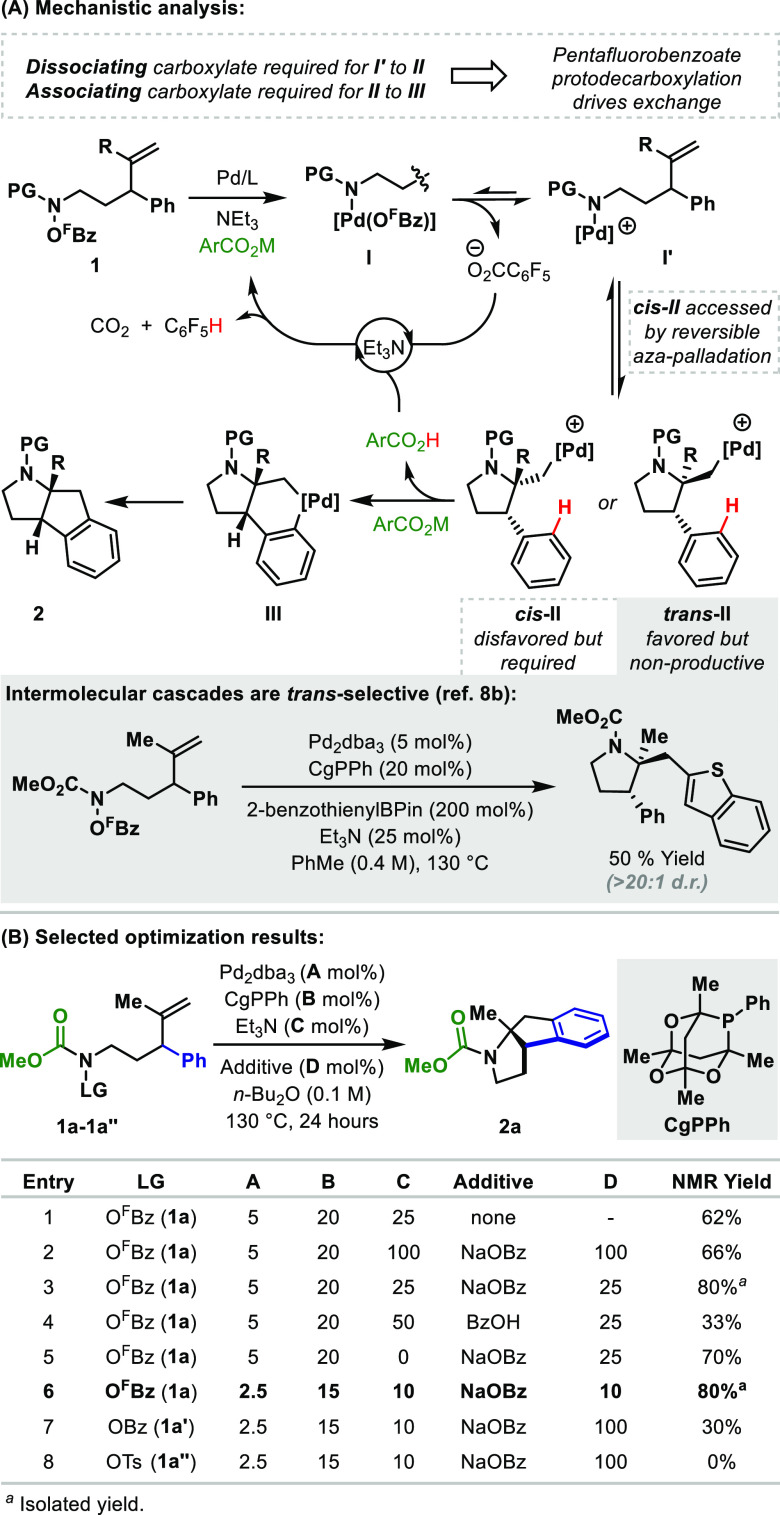
Mechanistic Analysis and Optimization of the Cascade Process Isolated yield.

In early efforts toward the envisaged cascades, we established that exposure of **1a** (LG = O^F^Bz) to a CgPPh-ligated Pd catalyst (10 mol %) at 130 °C generates tricyclic system **2a** in 62% yield and as a single diastereomer (Scheme 2B, entry 1). Under these conditions, *n*-Bu_2_O was the most effective solvent. To optimize the process, we evaluated a variety of carboxylate additives leading to the observation that addition of 25 mol % NaOBz improves the yield of **2a** to 80% (entry 3). The use of (in situ generated) triethylammonium benzoate was substantially less effective (entry 4), and a control experiment established that Et_3_N is required for optimal yields (entry 5).^15^ With optimized components in hand, we reassessed catalyst and additive loadings to provide the conditions in entry 6, which deliver **2a** in 80% yield using 5 mol % of the Pd catalyst and 10 mol % of the NaOBz and Et_3_N cocatalysts. Other leaving groups (entries 7 and 8), P-ligands, and carboxylate additives were less efficient, and the N-Ts analogue of **1a** (see the SI) did not undergo cyclization. The failure of system **1a″** (entry 8) supports the notion that a cationic manifold is required; previous studies indicate that *O*-tosyl activated systems cyclize in “neutral” mode.^8c^ Note that the C–N bond of **1a** is easily installed in 68% yield via Mitsunobu reaction of the corresponding alcohol with MeO_2_CNHO^F^Bz (see the SI).

Having established optimized conditions with **1a**, other carbamate protecting groups were evaluated (Table 1). Cyclization of N-Boc and N-Cbz systems **1b** and **1c** delivered **2b** and **2c** in 54% and 75% yield, respectively; notably, these processes were slower than with methyl carbamate **1a** (48 vs 24 h). Nevertheless, further scope studies were pursued using an N-Cbz group because this offered the best balance between yield and synthetic utility. A variety of electronically distinct *para*-substituted arenes (**1d**–**g**) engaged with minimal variation in efficiency. The process offers a good degree of flexibility for the alkene R-group, as evidenced by efficient cyclizations of systems possessing more bulky (**2i**) or conjugated substituents (**2h**). *Meta*-substituted arenes **1j** and **1k** have two different positions available for C–C bond formation, and the use of NaOBz as the additive offered no selectivity (1:1 r.r.). To address this, further carboxylate additives were screened, and these studies revealed that, in both cases, 2-MeOC_6_H_4_CO_2_Na favors C–C bond formation at the *para*-position with respect to the substituent (1.3:1 **2j**:**2j′** and 1.7:1 **2k**:**2k′**). Conversely, use of 2-NO_2_C_6_H_4_CO_2_Na switched this selectivity to provide **2j′** and **2k′** preferentially. These results are consistent with the carboxylate additive playing a key role in C–H palladation, although there is insufficient data to offer a precise rationalization for the observed regioselectivities. C–C bond formation was highly selective for heteroaromatics **2l** and **2m** using NaOBz as the carboxylate additive, presumably because these systems offer a substantial electronic bias for metalation. For **2l**, complete selectivity for the furan C2-position was observed, whereas C4 selectivity was observed for C3-pyridyl system **2m**. An unprotected indole participated efficiently to provide **2n** in 58% yield.

**Table 1:**
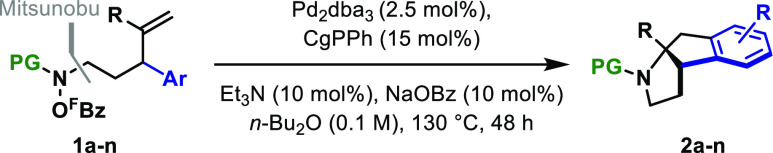

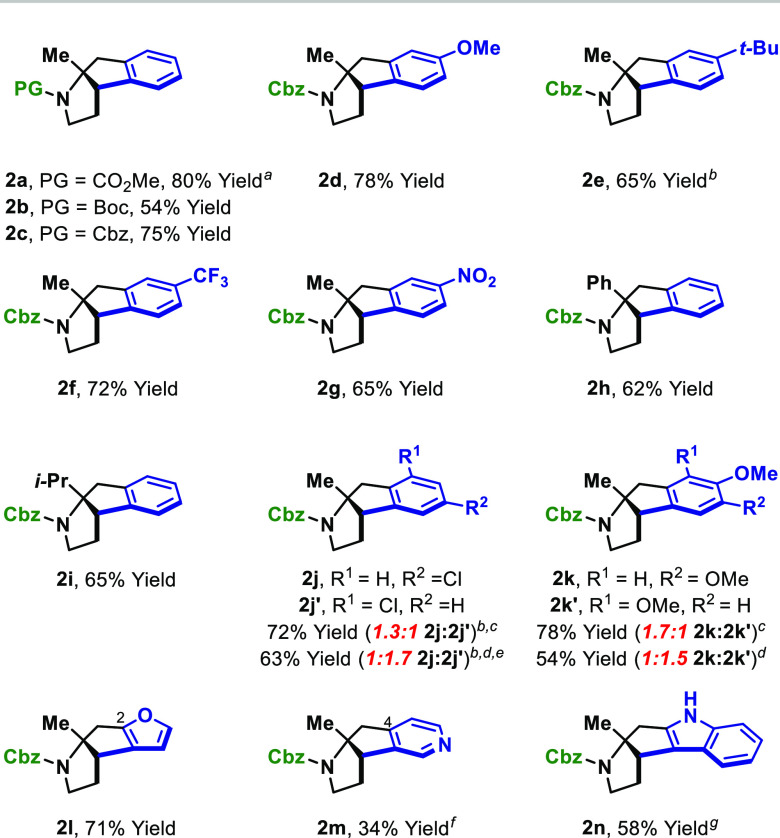
Cascades to Access Benzofused Polyheterocycles

a The reaction time was 24 h.

b The reaction time was 72 h.

c 10 mol % 2-MeOC_6_H_4_CO_2_Na was used in place of NaOBz.

d 10 mol % 2-NO_2_C_6_H_4_CO_2_Na was used in place of NaOBz.

e 5 mol % Pd_2_dba_3_ and 30 mol % CgPPh were used.

f 5 mol % Pd_2_dba_3_, 50 mol % CgPPh, and 150 °C were used.

g 5 mol % Pd_2_dba_3_, 30 mol % CgPPh, and 200 mol % Et_3_N were used.

We next evaluated distinct processes where the aromatic unit is appended to the internal position of the alkene (Table 2). For phenyl-substituted precursor **3a**, cyclization proceeded efficiently under optimized conditions to deliver spirocycle **4a** in 77% yield. As seen earlier, these processes are relatively insensitive to the electronics of the aromatic unit, such that methoxy and nitro variants **3b** and **3c** participated with similar levels of efficiency. Systems **4d** and **4e**, which possess α-substituents, were tolerated; the structure of product **4e** was confirmed by single-crystal X-ray diffraction. Cyclizations involving 3-quinolinyl (**4g**) and 3-furyl (**4f**) acceptors were also feasible, and C–C bond formation was completely selective for C4 and C2, respectively. To probe the possibility of diastereoselective processes, cyclizations of α-substituted systems **3h** and **3i**, which possess a *para*-methoxy substituent on the arene, were evaluated. In these cases, **4h** and **4i** were generated as single diastereomers, whose structures were verified by NOE analysis (see the SI). The outcome of these processes reflects the very high C2–C5 *cis*-diastereoselectivity associated with the alkene aza-palladation step. This enables control of the diastereoselectivity of the product even though the two desymmetrizing elements (the α-substituents and the methoxy groups) are distant from one another.

**Table 2:**
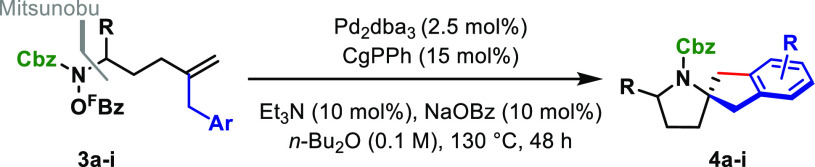

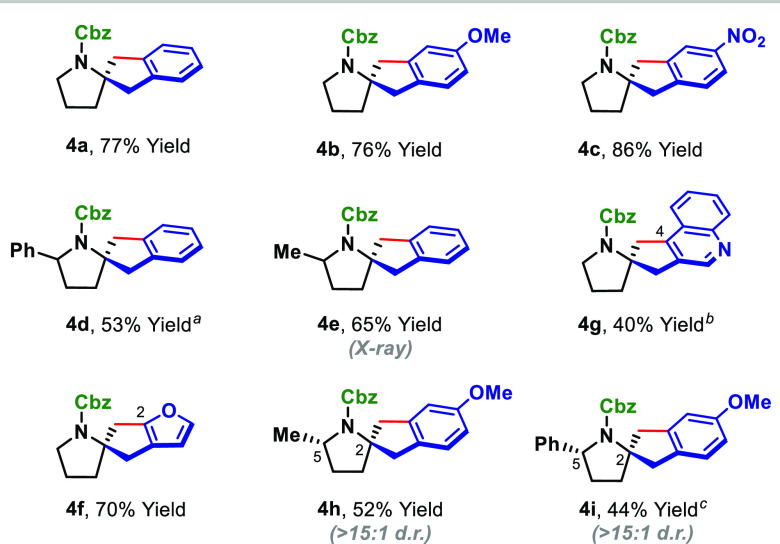
Cascades to Access Spirofused Polyheterocycles

a 150 °C, 3.75 mol % Pd_2_dba_3_, 30 mol % CgPPh.

b 150 °C, 5 mol % Pd_2_dba_3_, 50 mol % CgPPh.

c 160 °C, 5 mol % Pd_2_dba_3_, 40 mol % CgPPh.

Pileamartines A and B were recently isolated from the leaves of *Pilea* aff. *martinii* by Thanh, Pham, and co-workers and possess a stereochemically rich and compact framework.^11^ To demonstrate the utility of the aza-Heck cascades described here, we targeted a synthesis of the proposed natural enantiomer (**17**) (Scheme 3). This required an enantio- and diastereoselective synthesis of alcohol **11**. After extensive experimentation, we developed an efficient four-pot procedure for the installation of the challenging 1,3-stereorelationship. Exposure of epoxide **5** (>99% e.e.), which can be prepared in two steps,^16^ to the sodium enolate of **6** provided lactone **7** in 83% yield and 3:1 d.r.; the desired *cis*-diastereomer (***cis*****-7**) could be isolated in 61% yield. Optimization studies revealed three key observations: (1) a dilute DMF solution (0.04 M) is optimal for diastereoselectivity, (2) the diastereoselectivity is likely under kinetic control,^17^ and (3) the isopropyl ester of **6** is more efficient than the corresponding methyl ester. The latter is consistent with a bulkier ester suppressing a competing Claisen reaction. Lactone **7** was converted to *p*-bromobenzyl ester **8**, whose absolute stereochemistry was confirmed by single-crystal X-ray diffraction.

**Scheme 3:**
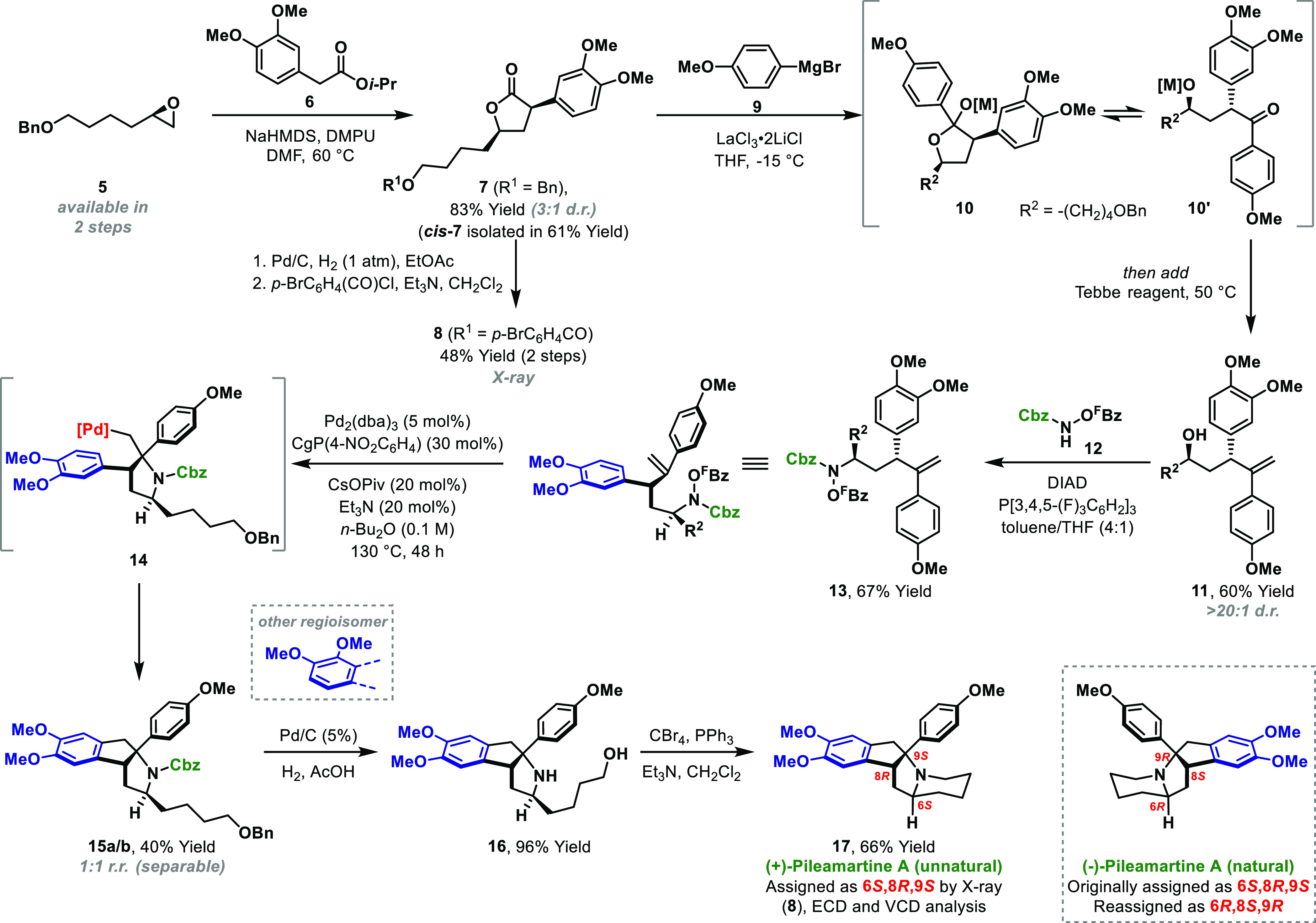
Total Synthesis of (+)-Pileamartine A

Conversion of **7** to alcohol **11** was nontrivial. In the event, we discovered that monoselective addition of Grignard **9** to lactone **7** can be achieved under Knochel conditions (LaCl_3_·LiCl),^18^ which presumably forms a mixture of **10** and **10′** in situ. Indeed, the protonated form of **10** was unstable and readily underwent dehydration to the corresponding dihydrofuran. To circumvent this, the subsequent olefination step was telescoped by direct exposure of **10/10′** to the Tebbe reagent at 50 °C, which provided alkene **11** (>20:1 d.r.) in 60% yield. The alcohol of **11** is relatively hindered, such that Mitsunobu reaction with **12** occurred in only 21% yield under standard conditions (PPh_3_, DIAD), with the mass balance consisting predominantly of elimination products. To address this, we sought to improve the leaving group ability of the oxyphosphonium intermediate by replacing PPh_3_ with a more electron poor phosphine [P(3,4,5-(F)_3_C_6_H_2_)_3_], and this modification provided **13** in 67% yield and >20:1 d.r.

The pivotal aza-Heck cascade to form **15** performed poorly under the conditions outlined in Table 1 (19% yield, 1:1 r.r.). Ultimately, by switching to a more electron poor phosphine [(CgP(4-NO_2_C_6_H_4_)] and using CsOPiv as the carboxylate additive, the core ring system **15a/b** could be accessed in 40% yield, albeit as a 1:1 mixture of regioisomers, resulting from nonselective C–H palladation at the stage of **14**. Efforts to improve selectivity by exploring alternative carboxylate additives were partially successful, but resulted in lower yields (see the SI). Nevertheless, the efficiency of the process is notable given it simultaneously installs the tetrasubstituted stereocenter and key C–C and C–N bonds. The process also serves to validate α-substituted substrates in cascades of this type (cf. Table 1). The desired regioisomer **15a** was advanced to the proposed structure of the natural product (**17**) via hydrogenative removal of the O-benzyl and N-Cbz units (to **16**) and subsequent cyclization under Appel conditions.

The ^1^H and ^13^C NMR data of **17** were in agreement with reported data; however, the specific rotation value [**17**: [α]^25^_D_ = +144.1 (*c* 0.32, CHCl_3_); natural pileamartine A [α]^25^_D_ = −141.2 (*c* 0.33, CHCl_3_)] and ECD spectrum (see the SI) were opposite of those determined for natural material. These data indicate that the original absolute stereochemical assignment of the natural product, which was made by comparison of experimental and calculated ECD spectra,^11^ is incorrect. In view of this, we recalculated the ECD spectrum of the 6*S*,8*R*,9*S* enantiomer at the B3PW91/cc-pVTZ level of theory and obtained a convincing match to the data for **17** (Figure S1).^19^ Measured and calculated VCD spectra were also in complete agreement (Figure S2). Taken together with X-ray data for **8**, there can be little doubt about the absolute configuration of **17**, and so we conclude that the configuration of natural pileamartine A should be reassigned as 6*R*,8*S*,9*R* (boxed structure).^20^

In summary, we show that aza-Heck cyclization of activated *N*-hydroxycarbamates can be used to trigger intramolecular aryl C–H functionalization cascades. These processes offer a counterpoint to oxidative Pd-catalyzed alkene 1,2-aminoarylations, and their utility has been validated in an eight-step synthesis of pileamartine A. Efforts to develop related methodologies are ongoing and will be reported in due course.

## Supporting Information Available

Experimental details, characterization data, and crystallographic data (PDF)

## Accession Codes

CCDC 2103391–2103392 contain the supplementary crystallographic data for this paper. These data can be obtained free of charge via www.ccdc.cam.ac.uk/data_request/cif, or by emailing data_request@ccdc.cam.ac.uk, or by contacting The Cambridge Crystallographic Data Centre, 12 Union Road, Cambridge CB2 1EZ, UK; fax: +44 1223 336033.
