# Supplementary material for: Complex Polyheterocycles and the Stereochemical Reassignment of Pileamartine A via Aza-Heck Triggered Aryl C–H Functionalization Cascades
Source: J Am Chem Soc. 2021 Sep 21;143(38):15593–8. doi: 10.1021/jacs.1c08615 (PMC8485351; doi:10.1021/jacs.1c08615)
Supplement: Supplementary file 1 — ja1c08615_si_001.pdf [file ja1c08615_si_001.pdf]

Complex Polyheterocycles and the Stereochemical Reassignment of Pileamartine A via Aza-Heck Triggered Aryl C-H Functionalization Cascades

Benjamin T. Jones,[†] Javier García-Cárceles,[†] Lewis Caiger,[†] Ian R. Hazelden,[†] Richard J. Lewis,[‡] Thomas Langer,[§] and John F. Bower*,[¥]

† School of Chemistry, University of Bristol, Bristol, BS8 1TS, United Kingdom

‡ Department of Medicinal Chemistry, Research and Early Development, Respiratory and Immunology, BioPharmaceuticals R&D, AstraZeneca, SE 43183 Mölndal, Sweden

§ Pharmaceutical Technology & Development, AstraZeneca, Charter Way, Macclesfield, SK10 2NA, United Kingdom

¥ Department of Chemistry, University of Liverpool, Crown Street, Liverpool, L69 7ZD, United Kingdom

Supporting Information

Table of Contents

General Experimental Details ……………………………………………………..……..……S2

Experimental Procedures and Data …………………………………….………..……………….S3

Preactivated Reagents for Mitsunobu Alkylation …………………….……..………………..S6

Phosphaadamantane Ligand synthesis……………………………...….………..….……….S8

Carboxylate Salt Synthesis………………………...………………….………..………...S11

Optimization of the Aza-Heck Cascade ……………………………………….……….……….S15

Substrate Synthesis and Catalysis for Table 1……………………….…………..………S17

Substrate Synthesis and Catalysis for Table 2……………………………..…..……………S58

Total Synthesis of (+)-Pileamartine A……………………….………..…………….……………S85

Experimental Procedures and Data …………………………………….…………….…S85

Optimization of the Key Catalysis Cyclization……………………………………..…...S96

Synthesis of (–)-Pileamartine A………………………………………………………...…S97

Vibrational Circular Dichroism (VCD) and Electronic Circular Dichroism (ECD) ……….S104

^{1}H and ^{13}C NMR Spectra of Novel Compounds ……………………………………….….S109

References …………………………………………………………………….………..……….S233

General Experimental Details

Starting materials were purchased from commercial sources (Acros, Aldrich, Alfa Aesar) and used without further purification unless otherwise stated. Anhydrous solvents were obtained by passage through drying columns supplied by Anhydrous Engineering Ltd. The removal of solvents in vacuo was achieved using both a Büchi rotary evaporator (bath temperatures up to 45 °C) at a pressure of either 15 mmHg (diaphragm pump) or 0.1 mmHg (oil pump), as appropriate, and a high vacuum line at room temperature. Reactions requiring anhydrous conditions were run under a dry atmosphere of nitrogen or argon; glassware was either flame dried immediately prior to use or placed in an oven (200 °C) for at least 2 hours and allowed to cool either in a desiccator or under an atmosphere of nitrogen or argon; liquid reagents, solutions or solvents were added *via* syringe through rubber septa. Flash column chromatography (FCC) was performed using silica gel (Aldrich 40-63 μm, 230-400 mesh). Thin-layer chromatography was performed using aluminium backed 60F254 silica plates. Visualisation was achieved by UV fluorescence or a basic KMnO4 solution and heat. Proton nuclear magnetic resonances were recorded at 400 MHz or 500 MHz. ^{13}C NMR spectra were recorded at 100 MHz or 125 MHz. 19F NMR spectra were recorded at 283 MHz. Chemical shifts (δ) are given in parts per million (ppm) and referenced to the appropriate residual solvent peak. Peaks are described as singlets (s), doublets (d), triplets (t), quartets (q), pentets (pent), sextets (sext), heptets (hept), multiplets (m) and broad (br). Coupling constants (J) are quoted to the nearest 0.5 Hz. Assignments of ^{1}H NMR and ^{13}C NMR signals were made, where possible, using COSY, HSQC, HMBC, and NOE experiments. Numbering systems for NMR signal assignments are specified on the structure and are not related to those used for the compound names. In situ yields were determined by integration of the 1H NMR of the crude material employing 1,3,5-trimethoxybenzene or 1,4-dinitrobenzene as internal standard. Mass spectra were determined by the University of Bristol mass spectrometry service using a Shimadzu GCMS QP2010+ (EI+ mode), Bruker ultrafleXtreme II TOF/TOF (MALDI, using a colloidal graphite matrix), Brüker Daltonics FT-ICR-MS Apex 4e 7.0T FT-MS (ESI+ mode), Thermo Scientific Orbitrap Elite (APCI mode). Infrared spectra were recorded on a Perkin Elmer Spectrum Two FTIR spectrometer as either neat films or solids compressed on a diamond plate. Abbreviations used are: weak (w), medium (m), strong (s) and broad (br). Melting points were determined using a Stuart SMP30 melting point apparatus and temperature controller and are uncorrected. Optical rotations were measured using an ADP440+ polarimeter at the concentration and temperature stated. Enantiomeric excesses were determined using an Agilent 1290 Infinity chiral SFC as stated for each compound. CD spectra were acquired with a JASCO J-815 CD spectrometer at 25 °C in a 1 mm path length quartz cuvette, with the solvent and concentration stated.

Experimental Procedures and Data

General Procedure A: Aldol condensation

To a stirred solution of benzaldehyde (1 eq.) in MeOH (1.3 M) was added aqueous NaOH solution (2.5 M, 1 eq.). The reaction was cooled to 0 °C and the enolizable aldehyde (equivalents specified in each case) was added dropwise using a syringe pump (1 mL/hour). The reaction was monitored by ^{1}H NMR, and enolizable aldehyde was added until complete consumption of benzaldehyde was observed (total equivalents specified in each case). Upon completion, MeOH was removed *in vacuo* and the residue was diluted with water (*approx.* 5 mL/mmol). The aqueous layer was extracted with Et_2O (*approx.* 3 × 5 mL/mmol), and the combined organic layers washed with brine (*approx.* 5 mL/mmol), dried (Na_2SO_4) and concentrated *in vacuo*. The residue was purified by FCC under the conditions noted.

General Procedure B: Mannich reaction

To a solution of 4-(dimethylamino)benzoic acid (0.1 eq.) and pyrrolidine (0.1 eq.) in CH_2Cl_2 (1 mL/mmol), was added formaldehyde (37% wt. in H_2O, 1.0 eq.) and aldehyde (1.0 eq.). The reaction was stirred at 45 °C for 1 hour. Upon completion, the reaction was quenched with sat. $NaHCO_3$ solution (*approx.* 5 mL/mmol) and the aqueous layer was extracted with CH_2Cl_2 (*approx.* 3 × 5 mL/mmol). The organic phase was washed with brine (*approx.* 5 mL/mmol), dried (Na_2SO_4) and concentrated *in vacuo*. The residue was purified by FCC under the conditions noted.

General Procedure C: Reduction of aldehydes

To a stirred solution of acrolein (1 eq.) in MeOH (1.8 M) at 0 °C, was added $NaBH_4$ (1.05 eq.) portionwise. The reaction was stirred at 0 °C for 30 minutes. Upon completion, the reaction was quenched with sat. NH_4Cl solution (*approx.* 2 mL/mmol), diluted with water (*approx.* 5 mL/mmol) and extracted with Et_2O (*approx.* 3 × 5 mL/mmol). The organic phase was washed with brine (*approx.* 5 mL/mmol), dried (Na_2SO_4), and concentrated *in vacuo*. The residue was purified by the technique specified in each case.

General Procedure D: Johnson-Claisen rearrangement

A solution of allylic alcohol (1 eq.) and either pivalic acid or propionic acid (equivalents specified) in triethyl orthoacetate (equivalents specified) equipped with a short-path distillation apparatus, was heated at 140 °C for the time noted (typically 16 hours). Upon completion, the reaction mixture was cooled to room temperature and concentrated *in vacuo*. If required, the residue was purified by FCC under the conditions noted.

<u>General Procedure E</u>: Johnson-Claisen rearrangement followed by hydrolysis

The hydrolysis step is required to obtain pure material.

Using a reaction vessel equipped with a short-path distillation apparatus, a solution of allylic alcohol (1 eq.) and either pivalic acid or propionic acid (equivalents specified) in triethyl orthoacetate (equivalents specified), was heated at 140 °C until the production of ethanol was no longer observed (approximately 3 hours). After cooling to room temperature, excess triethyl orthoacetate was removed *in vacuo* and the procedure was repeated until complete consumption of alcohol was observed by TLC (typically 2-3 cycles). Upon completion, the reaction mixture was cooled to room temperature and concentrated *in vacuo*. The residue was then dissolved in MeOH (0.2 M), and an aqueous solution of KOH (3M, 15 eq.) was added. The reaction was stirred at 60 °C for the time noted. MeOH was then removed *in vacuo*, and the aqueous layer was washed with Et_2O (*approx.* 3 × 10 mL/mmol) before being acidified (pH = 2) with 2M aq. HCl solution. The aqueous layer was extracted with Et_2O (*approx.* 3 × 10 mL/mmol), and the organic phase was washed with brine (*approx.* 5 mL/mmol), dried (Na_2SO_4), and concentrated *in vacuo* to afford the product.

<u>General Procedure F</u>: Reduction of carboxylic acids or esters using $LiAlH_4$

To a solution of carboxylic acid/ester (1.0 eq.) in anhydrous THF or Et_2O (0.2 M) at 0 °C was added $LiAlH_4$ (equivalents specified) dropwise. The reaction mixture was stirred at the temperature noted and monitored by TLC. Upon completion, the reaction mixture was quenched at 0 °C with the addition of water (1 mL/g of $LiAlH_4$), 15% aqueous NaOH (1 mL/g of $LiAlH_4$) and a final portion of water (3 mL/g of $LiAlH_4$). The reaction mixture was stirred at room temperature for around 15 minutes before being dried over Na_2SO_4 and concentrated *in vacuo*. If required, the residue was purified by FCC under the conditions noted.

<u>General Procedure G</u>: Swern oxidation of primary alcohols

To a solution of oxalyl chloride (1.2 eq.) in CH_2Cl_2 (4.2 mL/mmol) at -78 ºC was added DMSO (3.0 eq.) dropwise. The reaction was stirred at -78 ºC for 30 minutes. Next, a solution of the alcohol (1.0 eq.) in CH_2Cl_2 (0.75 mL/mmol) was added dropwise, and the reaction was stirred for an additional 30 minutes, after which time triethylamine (5.0 eq.) was added dropwise. The reaction was stirred at -78 ºC for 15 minutes before warming to room temperature. Upon completion, the reaction was diluted with water (*approx.* 5 mL/mmol) and the aqueous layer was extracted with CH_2Cl_2 (*approx.* 3 × 5 mL/mmol). The combined organic layers washed with water (*approx.* 5 mL/mmol), brine (*approx.* 5 mL/mmol), dried (Na_2SO_4) and concentrated *in vacuo*. The residue was purified by FCC under the conditions noted.

General Procedure H: Reaction of aliphatic aldehydes with Grignard reagents

To a solution of the Grignard reagent (1.6 eq.) in anhydrous Et_2O (1.6 mL/mmol) at 0 ºC was added the aldehyde (1.0 eq.) as a solution in anhydrous Et_2O (0.5 mL/mmol). The reaction mixture was stirred for 2 hours before of addition of saturated aqueous NH_4Cl (1.5 mL/mmol). The organic layer was dried (Na_2SO_4) and concentrated *in vacuo*. If required, the residue was purified by FCC under the conditions noted.

General Procedure I: Mitsunobu reaction

Triphenylphosphine (1.1 eq.) and carbamate (1 eq.) were added to a flame dried flask under nitrogen. Anhydrous solvent (0.4 M) was added (THF for OTs systems, toluene/THF 4:1 for $O^{F}Bz$ systems) and the reaction was cooled to 0 °C before adding the alcohol (1 eq.) *via* syringe. Diisopropyl azodicarboxylate (1.1 eq.) was then added and the reaction was stirred at room temperature for 16 hours. Upon completion, the reaction mixture was concentrated *in vacuo* and the residue was purified by FCC under the conditions noted.

General Procedure J: C-H functionalization aza-Heck cascade reaction

Note –The cyclization substrate must be dried prior to catalysis by passing through a plug of silica, followed by 3 cycles of dissolving and concentrating from anhydrous toluene. Thorough drying under high vacuum is required to remove all solvent.

A flame-dried Schlenk tube, fitted with a rubber septum, was charged with cyclization substrate, $Pd_2(dba)_3$, phosphine ligand and the carboxylate salt additive. The tube was purged with nitrogen, and anhydrous solvent and triethylamine were added *via* syringe. The tube was sealed with a plastic cap and stirred at the specified temperature for the time noted. The reaction mixture was concentrated *in vacuo* and the crude mixture purified by FCC under the conditions noted to afford the pure product.

Preactivated Substrates for Mitsunobu Alkylation

Methyl ((perfluorobenzoyl)oxy)carbamate

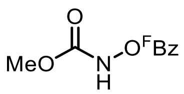

This compound was prepared according to our previously reported literature procedure.[1]

The spectroscopic properties were consistent with the data available in the literature.[1]

Methyl (tosyloxy)carbamate

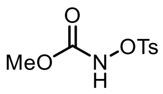

This compound was prepared according to a literature procedure.[2]

m.p.: 102-103 °C (CH_2Cl_2/hexane); R_f: 0.19 (10% EtOAc/toluene); ν_{max} / cm^{-1}: 3228 (m), 1733 (s), 1490 (m), 1389 (s), 1194 (s), 1181 (s); ^{1}H NMR (400 MHz, $CDCl_3$) δ 7.95 (1H, s, NH), 7.91 – 7.82 (2H, m, ArCH), 7.43 – 7.31 (2H, m, ArCH), 3.63 (3H, s, OCH_3), 2.46 (3H, s, Ts CH_3); ^{13}C NMR (101 MHz, $CDCl_3$) δ 156.1 (C=O), 146.3 (ArC), 130.4 (ArC), 129.9 (ArCH), 129.7 (ArCH), 53.8 (OCH_3), 21.9 (Ts CH_3); *m/z* (ESI^+) HRMS: Calculated for $C_9H_{11}NNaO_5S$: 268.0250. Found $[M+Na]^+$: 268.0262.

Methyl (benzoyloxy)carbamate

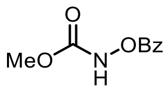

To a solution of methyl *N*-hydroxycarbamate (2.00 g, 22.0 mmol) in CH_2Cl_2 (55 mL) cooled to 0 °C, was added benzoyl chloride (2.55 mL, 22.0 mmol) and triethylamine (3.07 mL, 22.0 mmol) *via* syringe. The reaction was allowed to slowly warm to room temperature and stirred for 16 hours. Upon completion, the reaction was quenched with water (100 mL) and extracted with CH_2Cl_2. The combined organic layers were washed with brine, dried (Na_2SO_4) and concentrated *in vacuo*. The addition of hexane and subsequent concentration *in vacuo* gave the title compound (4.19g, 98%) as a colorless solid. m.p.: 83-84 °C (CH_2Cl_2/hexane); ν_{max} / cm^{-1}: 3259 (br s), 1748 (s), 1453 (s), 1236 (s); ^{1}H NMR (400 MHz, $CDCl_3$) δ 8.32 (1H, br s, NH), 8.13 – 8.07 (2H, m, ArCH), 7.65 (1H, tt, *J* = 7.5, 1.5 Hz, ArCH), 7.52 – 7.47 (2H, m, ArCH), 3.85 (3H, s, OCH_3); ^{13}C NMR (101 MHz, $CDCl_3$) δ 166.0 (Bz C=O), 157.2 (carbamate C=O), 134.4 (ArCH), 130.1 (ArCH), 128.9 (ArCH), 126.8 (ArC), 53.7 (OCH_3); *m/z* (ESI^+) HRMS: Calculated for $C_9H_9NNaO_4$: 218.0424. Found $[M+Na]^+$: 218.0425.

tert-Butyl ((perfluorobenzoyl)oxy)carbamate

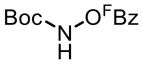

This compound was prepared according to our previously reported literature procedure.[1]

The spectroscopic properties were consistent with the data available in the literature.[1]

Benzyl ((perfluorobenzoyl)oxy)carbamate (12)

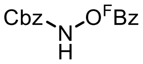

This compound was prepared according to our previously reported literature procedure.[1]

The spectroscopic properties were consistent with the data available in the literature.[1]

***N*-Hydroxy-4-methylbenzenesulfonamide**

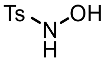

This compound was prepared according to a previously reported literature procedure.[3]

The spectroscopic properties were consistent with the data available in the literature.[4]

4-Methyl-*N*-((perfluorobenzoyl)oxy)benzenesulfonamide

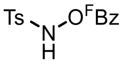

This compound was prepared according to a previously reported literature procedure.[5]

The spectroscopic properties were consistent with the data available in the literature.[5]

Phosphaadamantane Ligand synthesis

Ethyl 4-(1,3,5,7-tetramethyl-2,4,6-trioxa-8-phosphaadamantan-8-yl)benzoate

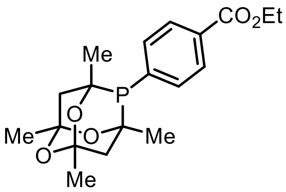

This compound was prepared according to our previously reported literature procedure.[1]

The spectroscopic properties were consistent with the data available in the literature.[1]

8-(3,5-Bis(trifluoromethyl)phenyl)-1,3,5,7-tetramethyl-2,4,6-trioxa-8-phosphaadamantane

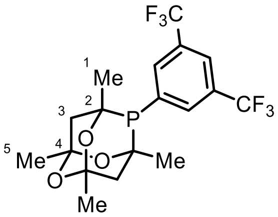

A suspension of 1,3,5,7-tetramethyl-2,4,6-trioxa-8-phosphaadamantane (1.30 g, 6.00 mmol), $Pd(PPh_3)_4$ (208 mg, 0.18 mmol), 1-bromo-3,5-bis(trifluoromethyl)benzene (0.88 mL, 5.00 mmol) and K_2CO_3 (1.66 g, 12.0 mmol) in anhydrous xylenes (30 mL) was heated at 110 °C for 20 hours. The reaction mixture was cooled to room temperature before being filtered through silica and rinsed with Et_2O. The filtrate was concentrated *in vacuo*. FCC (10% hexane/toluene) gave the title compound (891 mg, 42%) as a colorless solid. m.p. 133-134 °C (CH_2Cl_2:petrol). ν_{max} / cm^{-1}: 2980 (m), 2940 (m), 1615 (m), 1357 (s), 1276 (s), 1124 (s); ^{1}H NMR (400 MHz, $CDCl_3$) δ 8.33 (2H, br d, *J* = 6.0 Hz, ArC<u>H</u>), 7.88 (1H, br s, ArC<u>H</u>), 2.05 (1H, dd, *J* = 13.5, 8.0 Hz, C**3**-<u>H</u>), 1.96 (1H, dd, *J* = 24.5, 13.5 Hz, C**3**-<u>H</u>’), 1.55 – 1.53 (2H, m, C**3’**-$\underline{H_2}$), 1.51 (3H, d, *J* = 13.0 Hz, C**1**-$\underline{H_3}$), 1.43 (3H, s, C**5**-$\underline{H_3}$), 1.41 (3H, s, C**5’**-$\underline{H_3}$), 1.26 (3H, d, *J* = 13.5 Hz, C**1’**-$\underline{H_3}$); ^{13}C NMR (126 MHz, $CDCl_3$) δ 138.4 (d, *J* = 35.5 Hz, Ar<u>C</u>), 135.1 (d, *J* = 19.5 Hz, Ar<u>C</u>H), 131.6 (qd, *J* = 33.5, 6.5 Hz, Ar<u>C</u>), 123.3 (q, *J* = 273.0 Hz, <u>C</u>F_3), 123.3 (m, Ar<u>C</u>H), 97.1 (C**4**), 96.2 (C**4’**), 73.4 (d, *J* = 22.0 Hz, C**2**), 73.2 (d, *J* = 8.0 Hz, C**2’**), 45.1 (d, *J* = 17.5 Hz, C**3**), 36.3 (d, *J* = 2.0 Hz, C**3’**), 28.0 (C**5’**), 27.8 (C**5**), 27.5 (d, *J* = 22.0 Hz, C**1**), 26.9 (d, *J* = 11.0 Hz, C**1’**); ^{19}F NMR (377 MHz, $CDCl_3$) δ -63.0 (6F, s); ^{31}P NMR (162 MHz, $CDCl_3$) δ -26.2; *m/z* (ESI^+) HRMS: Calculated for $C_{18}H_{20}F_6O_3P$: 429.1049. Found $[M+H]^+$: 429.1031.

1,3,5,7-Tetramethyl-8-(*o*-tolyl)-2,4,6-trioxa-8-phosphaadamantane

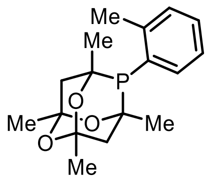

This compound was prepared according to a literature procedure.[6]

The spectroscopic properties were consistent with the data available in the literature.[6]

1,3,5,7-Tetramethyl-8-(4-nitrophenyl)-2,4,6-trioxa-8-phosphaadamantane

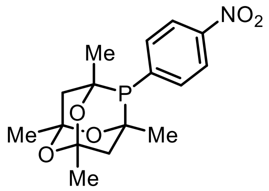

This compound was prepared according to a literature procedure.[7]

The spectroscopic properties were consistent with the data available in the literature.[7]

8-(3,5-Difluoro-4-nitrophenyl)-1,3,5,7-tetramethyl-2,4,6-trioxa-8-phosphaadamantane

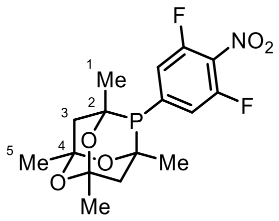

A suspension of 1,3,5,7-tetramethyl-2,4,6-trioxa-8-phosphaadamantane (324 mg, 1.50 mmol), $Pd(PPh_3)_4$ (52.0 mg, 0.045 mmol), 1-bromo-3,5-fluoro-4-nitrobenzene (357 mg, 1.50 mmol) and K_2CO_3 (622 mg, 4.50 mmol) in anhydrous toluene (7.50 mL) was heated at 110 °C for 20 hours. The reaction mixture was cooled to room temperature before being filtered through silica and rinsed with Et_2O. The filtrate was concentrated *in vacuo*. FCC (20% Et_2O/hexane) gave the title compound (429 mg, 77%) as a yellow solid. m.p. 147-149 °C (CH_2Cl_2/hexane); ν_{max} / cm^{-1}: 2973 (m), 2944 (m), 1606 (s), 1531 (s), 1353 (s), 1212 (s), 1050 (s), 890 (s); ^{1}H NMR (400 MHz, $CDCl_3$) δ 7.64 – 7.59 (2H, m, ArCH), 1.99 (1H, dd, *J* = 15.0, 13.5 Hz, C**3**-H), 1.95 (1H, dd, *J* = 28.0, 13.5 Hz, C**3**-H'), 1.59 – 1.57 (2H, m, C**3'**-H_2), 1.51 (3H, d, *J* = 13.0 Hz, C**1**-H_3), 1.42 (6H, s, 2 × C**5**-H_3), 1.31 (3H, d, *J* = 13.5 Hz, C**1'**-H_3); ^{13}C NMR (126 MHz, $CDCl_3$) δ 155.0 (dd, *J* = 8.5, 1.5 Hz, ArC), 152.9 (dd, *J* = 8.5, 1.5 Hz, ArC), 143.3 (dt, *J* = 40.5 Hz, 6.0 Hz, ArC), 118.9 (td, *J* = 19.5, 4.0 Hz, ArCH), 97.1 (C**4**), 96.3 (C**4'**), 73.5 (d, *J* = 8.5 Hz, C**2**). 73.3 (d, *J* = 23.0 Hz, C**2'**), 44.9 (d, *J* = 17.5 Hz, C**3**), 36.7 (d, *J* = 2.0 Hz, C**3'**), 28.0 (C**5**), 27.7 (C**5'**), 27.6 (d, *J* = 21.0 Hz, C**1**), 26.9 (d, *J* = 11.0 Hz, C**1'**); ^{19}F NMR (377 MHz, $CDCl_3$) δ -118.0 (2F,

s); ^{31}P NMR (162 MHz, $CDCl_3$) δ -23.5; *m/z* (ESI^+) HRMS: Calculated for $C_{16}H_{18}F_2NNaO_5P$: 396.0783. Found $[M+Na]^+$: 396.0780.

8-(3,5-Dinitrophenyl)-1,3,5,7-tetramethyl-2,4,6-trioxa-8-phosphaadamantane

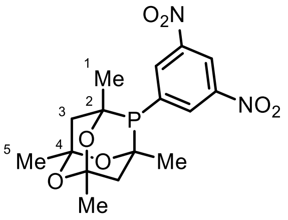

A suspension of 1,3,5,7-tetramethyl-2,4,6-trioxa-8-phosphaadamantane (324 mg, 1.50 mmol), $Pd(PPh_3)_4$ (52.0 mg, 0.045 mmol), 1-bromo-3,5-dinitrobenzene (347 mg, 1.50 mmol) and K_2CO_3 (622 mg, 4.50 mmol) in anhydrous toluene (7.50 mL) was heated at 110 °C for 20 hours. The reaction mixture was cooled to room temperature before being filtered through silica and rinsed with Et_2O. The filtrate was concentrated *in vacuo*. FCC (20% Et_2O/hexane) gave the title compound (16.5 mg, 3%) as a yellow solid. m.p. 179 - 180 °C (CH_2Cl_2/hexane); ν_{max} / cm^{-1}: 2913 (m), 1535 (s), 1341 (s), 975 (s), 891 (s), 725 (s); ^{1}H NMR (400 MHz, $CDCl_3$) δ 9.06 (2H, dd, *J* = 5.5, 2.0 Hz, ArCH), 9.02 (2H, t, *J* = 2.0 Hz, ArCH), 2.04 (1H, dd, *J* = 15.0, 13.5 Hz, C**3**-H), 2.00 (1H, dd, *J* = 29.0, 13.5 Hz, C**3**-H'), 1.62 – 1.51 (2H, m, C**3'**-H_2), 1.51 (3H, d, *J* = 13.0 Hz, C**1**-H_3), 1.46 (3H, s, C**5**-H_3), 1.44 (3H, s, C**5'**-H_3), 1.29 (3H, d, *J* = 13.5 Hz, C**1'**-H_3); ^{13}C NMR (101 MHz, $CDCl_3$) δ 148.5 (d, *J* = 6.0 Hz, ArC), 141.1 (d, *J* = 41.5 Hz, ArC), 134.8 (d, *J* = 20.5 Hz, ArCH), 119.6 (ArC), 97.2 (C**4**), 96.3 (C**4'**), 73.5 (d, *J* = 22.5 Hz, C**2**), 73.3 (d, *J* = 8.5 Hz, C**2'**), 44.8 (d, *J* = 17.5 Hz, C**3**), 36.4 (d, *J* = 17.5 Hz, C**3'**), 28.0 (C**5**), 27.7 (C**5'**), 27.6 (d, *J* = 22.0 Hz, C**1**), 26.9 (d, *J* = 11.0 Hz, C**1**); ^{31}P NMR (162 MHz, $CDCl_3$) δ -25.9; *m/z* (ESI^+) HRMS: Calculated for $C_{16}H_{20}N_2O_7P$: 383.1003. Found $[M+H]^+$: 383.1001.

Carboxylate Salt Additive Synthesis

General Procedure K: Carboxylic acid deprotonation

To a suspension of carboxylic acid (1.05 eq.) in water/MeOH 4:1 (0.4 M), was added NaOH (1 eq.). The reaction was stirred vigorously for 16 hours. Upon completion, the reaction was concentrated *in vacuo*. The resulting solid was triturated with pentane (*approx.* 3 × 1 mL/mmol) and Et_2O (*approx.* 3 × 1 mL/mmol) before drying at 100 °C under high vacuum for 24 hours to obtain the pure sodium salt.

Sodium 2-methoxybenzoate

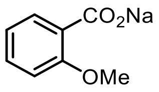

General procedure **K** was followed using 2-methoxybenzoic acid (761 mg, 5.00 mmol) and NaOH (190 mg, 4.75 mmol). The title compound (717 mg, 87%) was obtained as a colorless solid. m.p.: >330 °C (MeOH/H_2O); ^{1}H NMR (400 MHz, DMSO-d^6) δ 7.27 (1H, m, C**6**-H), 7.17 (1H, m, C**5**-H), 6.89 (1H, br d, J = 8.0 Hz, C**3**-H), 6.82 (1H, br t, J = 7.5 Hz, C**4**-H), 3.71 (3H, s, OCH_3); ^{13}C NMR (101 MHz, DMSO-d^6) δ 170.1 (C=O), 156.0 (ArC), 131.9 (ArC), 128.4 (ArCH), 128.1 (ArCH), 119.7 (ArCH), 111.7 (ArCH), 55.3 (OCH_3).

The spectroscopic properties were consistent with the data available in the literature.[8]

Sodium 2-nitrobenzoate

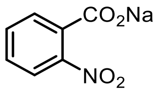

General procedure **K** was followed using 2-nitrobenzoic acid (501 mg, 3.00 mmol) and NaOH (114 mg, 2.86 mmol). The title compound (369 mg, 68%) was obtained as a colorless solid. m.p.: 192 – 193 °C (MeOH/H_2O); ^{1}H NMR (400 MHz, DMSO-d^6) δ 7.63 (1H, br d, J = 7.5 Hz, ArCH), 7.56 (1H, dd, J = 8.0, 1.5 Hz, ArCH), 7.51 (1H, td, J = 7.5, 1.5 Hz, ArCH), 7.38 (1H, m, ArCH); ^{13}C NMR (101 MHz, DMSO-d^6) δ 167.7 (C=O), 149.1 (ArC), 141.7 (ArC), 131.3 (ArCH), 129.7 (ArCH), 128.2 (ArCH), 122.1 (ArCH).

The spectroscopic properties were consistent with the data available in the literature.[9]

Sodium adamantane-1-carboxylate

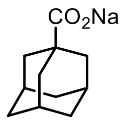

General procedure **K** was followed using 1-adamantanecarboxylic acid (901 mg, 5.00 mmol) and NaOH (190 mg, 4.75 mmol). The title compound (640 mg, 66%) was obtained as a colorless solid. m.p.: >300 °C (MeOH/H_2O).

The spectroscopic properties were consistent with the data available in the literature.[10]

2-Phenoxybenzoic acid

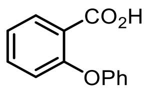

This compound was prepared according to a previously reported literature procedure.[11]

The spectroscopic properties were consistent with the data available in the literature.[12]

Sodium 2-phenoxybenzoate

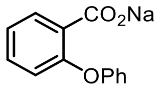

General procedure **K** was followed using 2-phenoxybenzoic acid (885 mg, 4.13 mmol) and NaOH (157 mg, 3.93 mmol). The title compound (730 mg, 77%) was obtained as a colorless solid. m.p.: >330 °C (MeOH/H_2O); ν_{max} / cm^{-1}: 2973 (s), 1607 (s), 1595 (s), 1385 (s), 1225 (s), 747 (s); ^{1}H NMR (400 MHz, DMSO-d^{6}) δ 7.45 (1H, dd, *J* = 7.5, 1.9 Hz, ArCH), 7.28 – 7.23 (2H, m, ArCH), 7.17 (1H, ddd, *J* = 8.0, 7.5, 2.0 Hz, ArCH), 7.05 (1H, td, *J* = 7.5, 1.0 Hz, ArCH), 6.96 (1H, tt, *J* = 7.5, 1.0 Hz, ArCH), 6.85 – 6.82 (2H, m, ArCH), 6.78 (1H, dd, *J* = 8.0, 1.0 Hz, ArCH); ^{13}C NMR (101 MHz, DMSO-d^{6}) δ 169.4 (C=O), 158.7 (ArC), 152.1 (ArC), 137.2 (ArC), 129.4 (ArCH), 129.3 (ArCH), 127.8 (ArCH), 123.4 (ArCH), 121.5 (ArCH), 120.4 (ArCH), 117.4 (ArCH); *m/z* (ES^{-}) HRMS: Calculated for anion $C_{13}H_9O_3$: 213.0552. Found [M-Na]$^{-}$: 213.0558.

2-Isopropoxybenzoic acid

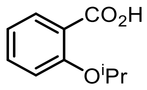

This compound was prepared according to a previously reported literature procedure.[13]

The spectroscopic properties were consistent with the data available in the literature.[13]

Sodium 2-isopropoxybenzoate

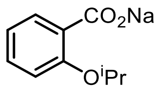

General procedure **K** was followed using 2-isopropoxybenzoic acid (163 mg, 0.90 mmol) and NaOH (34.4 mg, 0.86 mmol). The title compound (97.7 mg, 63%) was obtained as a colorless solid. m.p.: >330 °C (MeOH/H_2O); ν_{max} / cm^{-1}: 2973 (m), 1589 (s), 1599 (s), 1382 (s); ^{1}H NMR (400 MHz, DMSO-d^6) δ 7.23 (1H, dd, *J* = 7.5, 2.0 Hz, ArCH), 7.08 (1H, td, *J* = 7.5, 2.0 Hz, ArCH), 6.84 (1H, d, *J* = 8.0 Hz, ArCH), 6.80 (1H, t, *J* = 7.5 Hz, ArCH), 4.51 (1H, hept, *J* = 6.0 Hz, *i*-Pr CH), 1.20 (6H, d, *J* = 6.0 Hz, 2 × *i*-Pr CH_3); ^{13}C NMR (101 MHz, DMSO-d^6) δ 171.7 (C=O), 153.8 (ArC), 135.6 (ArC), 128.5 (ArCH), 127.1 (ArCH), 120.1 (ArCH), 116.3 (ArCH), 70.6 (*i*-Pr CH), 22.2 (2 × *i*-Pr CH_3); *m/z* (ES$^-$) HRMS: Calculated for anion $C_{10}H_{11}O_3$: 179.0708. Found [M-Na]$^-$: 179.0710.

Sodium 2-(dimethylamino)benzoate

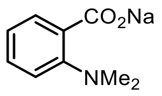

General procedure **K** was followed using 2-dimethylaminobenzoic acid (496 mg, 3.00 mmol) and NaOH (114 mg, 2.86 mmol). The title compound (481 mg, 90%) was obtained as a colorless solid. m.p.: >330 °C (MeOH/H_2O); ν_{max} / cm^{-1}: 2967 (m), 2833 (m), 1602 (s), 1584 (s), 1374 (s); ^{1}H NMR (400 MHz, DMSO-d^6) δ 7.15 (1H, dd, *J* = 7.5, 2.0 Hz, ArCH), 7.01 (1H, ddd, *J* = 8.0, 7.0, 2.0 Hz, ArCH), 6.73 (1H, dd, *J* = 8.0, 1.0, ArCH), 6.68 (1H, td, *J* = 7.5, 1.0 Hz, ArCH), 2.72 (6H, s, 2 × NCH_3); ^{13}C NMR (101 MHz, DMSO-d^6) δ 173.8 (C=O), 148.6 (ArC), 136.2 (ArC), 128.3 (ArCH), 126.5 (ArCH), 118.7 (ArCH), 115.6 (ArCH), 43.1 (2 × NCH_3); *m/z* (ES$^-$) HRMS: Calculated for anion $C_9H_{10}NO_2$: 164.0712. Found [M-Na]$^-$: 164.0718.

Sodium 2-(methylthio)benzoate

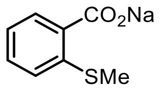

General procedure **K** was followed using 2-(methylthio)benzoic acid (505 mg, 3.00 mmol) and NaOH (114 mg, 2.86 mmol). The title compound (471 mg, 87%) was obtained as a colorless solid. m.p.: 305 – 306 °C (MeOH/H_2O); ν_{max} / cm^{-1}: 2967 (m), 1594 (s), 1576 (s), 1558 (s), 1399 (s), 1055 (s), 1033 (s), 740 (s); ^{1}H NMR (400 MHz, DMSO-d^6) δ 7.75 (1H, dd, *J* = 7.5, 1.5 Hz, ArCH), 7.23 (1H, ddd, *J* = 8.0, 7.0, 1.5 Hz, ArCH), 7.10 (1H, br d, *J* = 7.5 Hz, ArCH), 7.00 (1H, td, 7.5, 1.0 Hz, ArCH), 2.24 (3H, s, SCH_3); ^{13}C NMR (101 MHz, DMSO-d^6) δ 170.5 (C=O), 139.4 (ArC), 138.3 (ArC), 129.8 (ArCH), 128.4 (ArCH), 123.1 (ArCH), 122.5 (ArCH), 15.2 (SCH_3); *m/z* (ES$^-$) HRMS: Calculated for anion $C_8H_7O_2S$: 167.0167. Found [M-Na]$^-$: 167.0164.

Sodium 2,4-dimethoxybenzoate

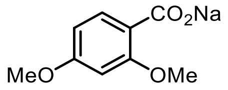

General procedure **K** was followed using 2,4-dimethoxybenzoic acid (547 mg, 3.00 mmol) and NaOH (114 mg, 2.86 mmol). The title compound (550 mg, 94%) was obtained as a colorless solid. m.p.: 279 – 281 °C (MeOH/H_2O); ν_{max} / cm^{-1}: 2967 (m), 1609 (s), 1585 (s),1567 (s), 1392 (s), 1053 (s), 1033 (s), 1020 (s); ^{1}H NMR (400 MHz, DMSO-*d*^{6}) δ 7.36 (1H, d, *J* = 8.0 Hz, ArCH), 6.41 (1H, d, *J* = 2.5 Hz, ArCH), 6.68 (1H, dd, *J* = 8.5, 2.5 Hz, ArCH), 3.73 (3H, s, OCH_3), 3.69 (3H, s, OCH_3); ^{13}C NMR (101 MHz, DMSO-*d*^{6}) δ 170.4 (C=O), 159.5 (ArC), 157.8 (ArC), 130.5 (ArCH), 125.5 (ArC), 103.9 (ArCH), 98.8 (ArCH), 55.4 (OCH_3), 55.0 (OCH_3); *m/z* (ES^-) HRMS: Calculated for anion $C_9H_9O_4$: 181.0501. Found $[M\text{-}Na]^-$: 181.0495.

Sodium 2-oxidobenzoate

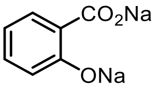

General procedure **K** was followed using salicylic acid (414 mg, 3.00 mmol) and NaOH (240 mg, 6.00 mmol). The title compound (445 mg, 82%) was obtained as a colorless solid. m.p.: >330 °C (MeOH/H_2O); ν_{max} / cm^{-1}: 2973 (m), 1589 (s), 1549 (s), 1530 (s), 1389 (s), 1326 (s), 1033 (s); ^{1}H NMR (400 MHz, D_2O) δ 7.55 (1H, dd, *J* = 7.5, 1.5 Hz, ArCH), 7.30 (1H, ddd, *J* = 8.0, 7.0, 2.0 Hz, ArCH), 6.79 (1H, br d, *J* = 8.0 Hz, ArCH), 6.75 (1H, br t, *J* = 7.5 Hz, ArCH); ^{13}C NMR (101 MHz, D_2O) δ 177.3 (C=O), 161.6 (ArC), 132.3 (ArCH), 129.6 (ArCH), 123.2 (ArC), 118.1 (ArCH), 116.4 (ArCH); *m/z* (ES^-) HRMS: Calculated for anion $C_7H_5O_3$: 137.0239. Found $[M\text{-}Na+H]^-$: 137.0236.

8-Methoxy-1-naphthoic acid

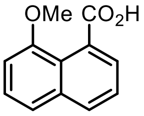

This compound was prepared according to a previously reported literature procedure.[14]

The spectroscopic properties were consistent with the data available in the literature.[14]

Sodium 8-methoxy-1-naphthoate

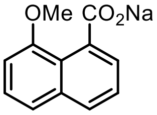

General procedure **K** was followed using the preceding carboxylic acid (244 mg, 1.21 mmol) and NaOH (46 mg, 1.15 mmol). The title compound (216 mg, 80%) was obtained as a colorless solid. m.p.: >330 °C (MeOH/H_2O); ν_{max} / cm^{-1}: 2988 (m), 1561 (s), 1412 (s), 1374 (s), 1254 (s), 1056 (s), 822 (s), 759 (s); ^{1}H NMR (400 MHz, DMSO-*d*^{6}) δ 7.60 (1H, dd, *J* = 8.0, 1.5 Hz, ArCH), 7.39 – 7.31 (3H, m, ArCH), 7.06 (1H, dd, *J* = 7.0, 1.5 Hz, ArCH), 6.84 (1H, dd, *J* = 7.5, 1.5 Hz, ArCH), 3.79 (3H, s, OCH_3); ^{13}C

NMR (101 MHz, DMSO-d^6) δ 174.1 (C=O), 156.1 (ArC), 141.3 (ArC), 134.6 (ArC), 125.7 (ArCH), 125.5 (ArCH), 124.6 (ArCH), 122.1 (ArCH), 121.0 (ArC), 120.0 (ArCH), 105.0 (ArCH), 55.6 (OCH_3); *m/z* (ES^-) HRMS: Calculated for anion $C_{12}H_9O_3$: 201.0552. Found $[M-Na]^-$: 201.0545.

The title compound has limited solubility in DMSO resulting in weak ^{13}C signals. The carbonyl and aromatic quaternary carbons were assigned using the HMBC correlation.

Optimization of the Aza-Heck Cascade

Selected additional optimization experiments are presented below:

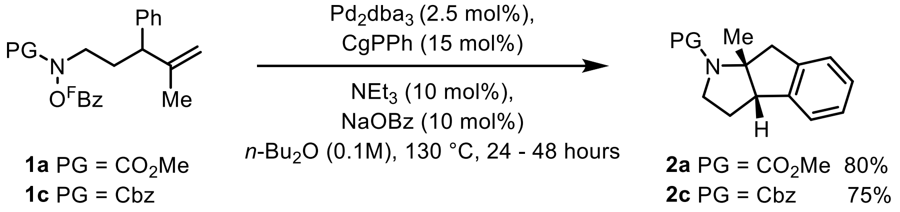

Entry	PG	Variations from standard conditions	NMR Yield
1	Cbz	$P(3,5-(CF_3)_2C_6H_3)_3$ instead of CgPPh	40%
2	Cbz	$P(4-(F)C_6H_4)_3$ instead of CgPPh	16%
3	Cbz	$CgP(4-(CO_2Et)C_6H_4)$ instead of CgPPh	71%
4	Cbz	$CgP(3,5-(CF_3)_2C_6H_3)$ instead of CgPPh	32%
5	Cbz	$CgP(2-(Me)C_6H_4)$ instead of CgPPh	8%
6	CO_2Me	100 °C	8%
7	CO_2Me	110 °C	17%
8	CO_2Me	120 °C	35%
9	Cbz	140 °C	67%
10	CO_2Me	150 °C	70%

Evaluation of Other Carboxylates:

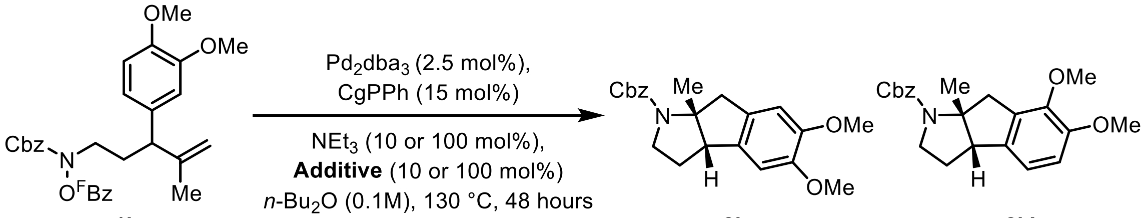

1k **2k** **2k'**

Entry	Additive		NMR Yield	Approx. ratio by NMR (2k:2k')
**1**	NaOBz (10 mol%)		76%	1:1
**2**	AdCO$_2$Na (10 mol%)		68%	1:1
**3**	CsOPiv (100 mol%)		73%[a]	1:1
**4**	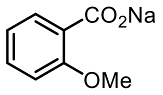	(10 mol%)	78% (isolated)	1.7:1
**5**	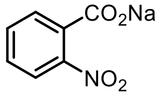	(10 mol%)	54% (isolated)	1:1.5
**6**	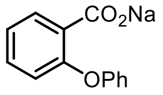	(100 mol%)	84%[a]	1:1
**7**	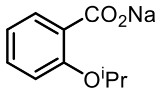	(100 mol%)	77%[a]	1.7:1
**8**	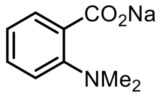	(100 mol%)	75%[a]	1:1
**9**	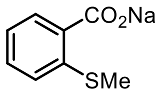	(100 mol%)	86%[a]	1.7:1
**10**	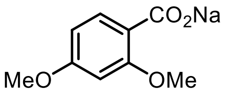	(100 mol%)	82%[a]	1.7:1
**11**	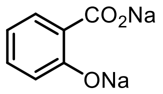	(100 mol%)	42%[a]	1:1.5
**12**	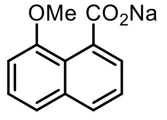	(100 mol%)	76%[a]	1.3:1

[a] $Pd_2(dba)_3$ (5 mol%) and CgPPh (5 mol%) used, resulting in higher yields for some examples.

Substrate Synthesis and Catalysis for Table 1

4-Methyl-3-phenylpent-4-enoic acid

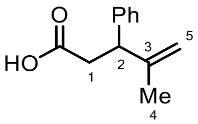

General procedure **E** was followed using *trans*-2-methyl-3-phenyl-2-propen-1-ol (4.45 g, 30.0 mmol), pivalic acid (613 mg, 6.00 mmol) and triethyl orthoacetate (38.5 mL, 210 mmol) for 2 cycles. The subsequent hydrolysis was stirred at 60 °C for 16 hours. The title compound was obtained (2.5 g, 44%) as a light yellow oil. ^{1}H NMR (400 MHz, $CDCl_3$) δ 11.05 (1H, br s, CO_2H), 7.33 – 7.27 (2H, m, ArCH), 7.25 – 7.20 (3H, m, ArCH), 4.94 (1H, m, C**5**-H), 4.91 (1H, m, C**5**-H'), 3.78 (1H, dd, *J* = 8.0, 8.0 Hz, C**2**-H), 2.90 (1H, dd, *J* = 15.5, 8.0 Hz, C**1**-H), 2.75 (1H, dd, *J* = 15.5, 8.0 Hz, C**1**-H'), 1.61 (3H, s, C**4**-H_3); ^{13}C NMR (101 MHz, $CDCl_3$) δ 178.3 (C=O), 146.8 (C**3**), 142.1 (ArC), 128.9 (ArCH), 128.0 (ArCH), 127.1 (ArCH), 111.0 (C**5**), 48.5 (C**2**), 39.3 (C**1**), 22.0 (C**4**).

The spectroscopic properties were consistent with the data available in the literature.[15]

4-Methyl-3-phenylpent-4-en-1-ol

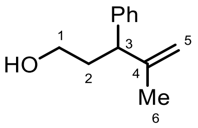

General procedure **F** was followed using the preceding carboxylic acid (2.50 g, 13.1 mmol), $LiAlH_4$ (1M in THF, 26.3 mL) and anhydrous Et_2O at room temperature for 3 hours. The title compound (1.92 g, 83%) was obtained as a colorless oil. ^{1}H NMR (400 MHz, $CDCl_3$) δ 7.36 – 7.18 (5H, m, ArCH), 4.95 (1H, m, C**5**-H), 4.86 (1H, m, C**5**-H'), 3.60 (2H, m, C**1**-H_2), 3.42 (1H, dd, *J* = 7.5, 7.5 Hz, C**3**-H), 2.18 – 2.10 (1H, m, C**2**-H), 2.05 – 1.93 (1H, m, C**2**-H'), 1.59 (3H, s, C**6**-H_3), 1.40 (1H, br s, OH); ^{13}C NMR (101 MHz, $CDCl_3$) δ 147.8 (C**4**), 142.9 (Ar C), 128.3, 127.8, 126.4 (ArCH), 110.5 (C**5**), 61.3 (C**1**), 49.0 (C**3**), 35.7 (C**2**), 21.0 (C**6**).

The spectroscopic properties were consistent with the data available in the literature.[16]

Methyl (4-methyl-3-phenylpent-4-en-1-yl)((perfluorobenzoyl)oxy)carbamate (1a):

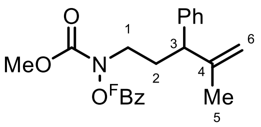

General procedure **I** was followed using MeO_2CNHO^FBz (1.71 g, 6.00 mmol), the preceding alcohol (1.06 g, 6.00 mmol), triphenylphosphine (1.73 g, 6.60 mmol) and diisopropyl azodicarboxylate (1.30

mL, 6.60 mmol). FCC (3% EtOAc/petroleum ether) gave title compound **1a** (1.81 g, 68%) as a light yellow oil. R_f: 0.32 (5% EtOAc/petroleum ether); ν_{max} / cm^{-1}: 2958 (m), 1786 (s), 1736 (s), 1524 (s), 1506 (s), 1327 (s), 1176 (s); ^{1}H NMR (400 MHz, $CDCl_3$) δ 7.32 – 7.26 (2H, m, ArC<u>H</u>), 7.24 – 7.18 (3H, m, ArC<u>H</u>), 4.93 (1H, m, C**6**-<u>H</u>), 4.88 (1H, m, C**6**-<u>H</u>'), 3.79 (3H, s, OC<u>H</u>$_3$), 3.72 (1H, ddd, *J* = 15.0, 9.0, 6.0 Hz, C**1**-<u>H</u>), 3.62 (1H, ddd, *J* = 15.0, 9.0, 6.0 Hz, C**1**-<u>H</u>'), 3.34 (1H, dd, *J* = 7.5, 7.5 Hz, C**3**-<u>H</u>), 2.21 (1H, dddd, *J* = 13.5, 8.5, 7.5, 6.0 Hz, C**2**-<u>H</u>), 2.07 (1H, dtd, *J* = 13.5, 8.0, 6.0 Hz, C**2**-<u>H</u>'), 1.59 (3H, br s, C**5**-<u>H</u>$_3$); ^{13}C NMR (101 MHz, $CDCl_3$) δ 156.2 (carbamate <u>C</u>=O), 146.9 (C**4**), 142.4 (Ph Ar<u>C</u>), 128.6 (Ph ArC<u>H</u>), 127.8 (Ph ArC<u>H</u>), 126.7 (Ph ArC<u>H</u>), 111.0 (C**6**), 54.1 (O<u>C</u>H$_3$), 50.2 (C**1**), 49.7 (C**3**), 30.2 (C**2**), 21.1 (C**5**); ^{19}F NMR (377 MHz, $CDCl_3$) δ -136.0 – -136.2 (2F, m), -146.1 (1F, tt, *J* = 21.0, 5.5 Hz), -159.2 – -159.4 (2F, m); *m/z* (ESI$^+$) HRMS: Calculated for $C_{21}H_{18}F_5NNaO_4$: 466.1048. Found [M+Na]$^+$: 466.1057.

The carbon signals corresponding to the pentafluorobenzoyl group could not be resolved due to their weak intensity.

Methyl (benzoyloxy)(4-methyl-3-phenylpent-4-en-1-yl)carbamate (1a')

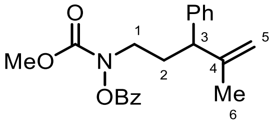

General procedure **I** was followed using $MeO_2CNHOBz$ (195 mg, 1.00 mmol), the preceding alcohol (176 mg, 1.00 mmol), triphenylphosphine (289 mg, 1.10 mmol) and diisopropyl azodicarboxylate (0.216 mL, 1.10 mmol). FCC (10% EtOAc/hexane) gave title compound **1a'** (320 mg, 91%) as a colorless oil. R_f: 0.26 (10% EtOAc/hexane); ν_{max} / cm^{-1}: 2954 (m), 1764 (s), 1717 (s), 1451 (s), 1240 (s); ^{1}H NMR (400 MHz, $CDCl_3$) δ 8.09 – 8.04 (2H, m, Bz ArC<u>H</u>), 7.67 – 7.60 (1H, tt, *J* = 7.5, 1.5 Hz, Bz ArC<u>H</u>), 7.53 – 7.46 (2H, m, Bz ArC<u>H</u>), 7.30 – 7.24 (2H, m, Ph ArC<u>H</u>), 7.23 – 7.17 (3H, m, Ph ArC<u>H</u>), 4.93 (1H, m, C**5**-<u>H</u>), 4.86 (1H, m, C**5**-<u>H</u>'), 3.76 (3H, s, OC<u>H</u>$_3$), 3.75 – 3.60 (2H, m, C**1**-<u>H</u>$_2$), 3.35 (1H, dd, *J* = 7.5, 7.5 Hz, C**3**-<u>H</u>), 2.28 – 2.17 (1H, m, C**2**-<u>H</u>), 2.14 – 2.04 (1H, m, C**2**-<u>H</u>'), 1.58 (3H, br s, C**6**-<u>H</u>$_3$); ^{13}C NMR (101 MHz, $CDCl_3$) δ 164.6 (Bz <u>C</u>=O), 156.4 (carbamate <u>C</u>=O), 147.1 (C**4**), 142.7 (Bz Ar<u>C</u>), 134.2 (Bz ArC<u>H</u>), 130.1 (Bz ArC<u>H</u>), 128.8 (Bz ArC<u>H</u>), 128.5 (Ph Ar<u>C</u>H), 127.9 (Ph Ar<u>C</u>H), 127.4 (Ph Ar<u>C</u>), 126.6 (Ph Ar<u>C</u>H), 111.0 (C**5**), 53.7 (O<u>C</u>H$_3$), 49.9 (C**3**), 49.8 (C**1**), 30.5 (C**2**), 21.1 (C**6**); *m/z* (ESI$^+$) HRMS: Calculated for $C_{21}H_{23}NNaO_4$: 376.1519. Found [M+Na]$^+$: 376.1515.

Some signal broadening was observed due to amide-like resonance.

Methyl (4-methyl-3-phenylpent-4-en-1-yl)(tosyloxy)carbamate (1a'')

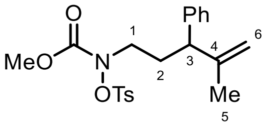

General procedure **I** was followed using $MeO_2CNHOTs$ (736 mg, 3.00 mmol), the preceding alcohol (529 mg, 3.00 mmol), triphenylphosphine (866 mg, 3.30 mmol) and diisopropyl azodicarboxylate (0.649 mL, 3.30 mmol). FCC (15% EtOAc/petroleum ether) gave title compound **1a''** (1.06 g, 87%) as a colorless oil. R_f: 0.19 (toluene); ν_{max} / cm^{-1}: 2956 (m), 1760 (m), 1727 (s), 1382 (s), 1190 (s), 1177 (s); ^1H NMR (400 MHz, $CDCl_3$) δ 7.83 – 7.77 (2H, m, Ts ArCH), 7.35 – 7.13 (7H, m, Ts + Ph ArCH), 4.89 (1H, m, C**6**-H), 4.83 (1H, m, C**6**-H'), 3.63 – 3.31 (2H, m, C**1**-H_2), 3.48 (3H, s, OCH_3), 3.15 (1H, dd, *J* = 8.0, 8.0 Hz, C**3**-H), 2.44 (3H, s, Ts CH_3), 2.19 – 1.96 (2H, m, C**2**-H_2), 1.55 (3H, s, C**5**-H_3); ^{13}C NMR (101 MHz, $CDCl_3$) δ 157.2 (C=O), 146.8 (C**4**), 146.0 (Ts ArC), 142.4 (Ph ArC), 131.2 (Ts ArC), 129.6 (Ts ArCH), 129.6 (Ts ArCH), 128.5 (Ph ArCH), 127.7 (Ph ArCH), 126.7 (Ph ArCH), 111.0 (C**6**), 53.8 (OCH_3), 52.0 (C**1**), 50.2 (**C**3), 28.9 (C**2**), 21.9 (Ts CH_3), 20.8 (C**5**); *m/z* (ESI^+) HRMS: Calculated for $C_{21}H_{25}NNaO_5S$: 426.1346. Found $[M+Na]^+$: 426.1345.

Significant signal broadening was observed due to amide-like resonance.

4-Methyl-*N*-(4-methyl-3-phenylpent-4-en-1-yl)-*N*-((perfluorobenzoyl)oxy)benzenesulfonamide

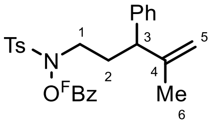

General procedure **I** was followed using $TsNHO^FBz$ (381 mg, 1.00 mmol), the preceding alcohol (159 mg, 0.90 mmol), triphenylphosphine (262 mg, 1.00 mmol) and diisopropyl azodicarboxylate (0.20 mL, 1.00 mmol). FCC (10% EtOAc/hexane) gave the title compound (357 mg, 66%) as a colorless oil. R_f: 0.33 (10% EtOAc/hexane); ν_{max} / cm^{-1}: 2940 (m), 1785 (s), 1497 (s), 1168 (s); ^1H NMR (400 MHz, $CDCl_3$) δ 7.75 (2H, d, *J* = 8.0 Hz, Ts ArCH), 7.35 (2H, d, *J* = 8.0 Hz, Ts ArCH), 7.31 – 7.26 (2H, m, ArCH), 7.23 – 7.18 (3H, m, ArCH), 4.90 – 4.87 (2H, m, C**5**-H_2), 3.51 (1H, dd, *J* = 7.5, 7.5 Hz, C**3**-H), 3.28 – 3.05 (2H, m, C**1**-H_2), 2.46 (3H, s, Ts CH_3), 2.16 – 2.07 (1H, m, C**2**-H), 2.00 – 1.90 (1H, m, C**2**-H'), 1.58 (3H, br s, C**6**-H_3); ^{13}C NMR (101 MHz, $CDCl_3$) δ 146.9 (C**4**), 146.0 (Ts ArC), 142.3 (ArC), 130.2 (Ts ArC), 130.0 (Ts ArCH), 129.7 (Ts ArCH), 128.6 (ArCH), 128.0 (ArCH), 126.8 (ArCH), 110.9 (C**5**), 51.2 (C**1**), 49.1 (C**3**), 30.3 (C**2**), 21.8 (Ts CH_3), 21.5 (C**6**); *m/z* (ESI^+) HRMS: Calculated for $C_{26}H_{22}F_5NNaO_4S$: 562.1082. Found $[M+Na]^+$: 562.1084.

***tert*-Butyl (4-methyl-3-phenylpent-4-en-1-yl)((perfluorobenzoyl)oxy)carbamate (1b)**

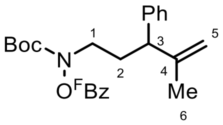

General procedure **I** was followed using $BocNHO^FBz$ (654 mg, 2.00 mmol), the preceding alcohol (353 mg, 2.00 mmol), triphenylphosphine (577 mg, 2.20 mmol) and diisopropyl azodicarboxylate (0.433 mL, 2.20 mmol). FCC (2% EtOAc/hexane) gave title compound **1b** (320 mg, 33%) as a colorless oil. R_f:

0.21 (2% EtOAc/hexane); ν_{max} / cm^{-1}: 2981 (m), 2937 (m), 1784 (s), 1725 (s), 1524 (s), 1506 (s); ^{1}H NMR (400 MHz, $CDCl_3$) δ 7.31 – 7.26 (2H, m, Ph ArC$\underline{H}$), 7.23 – 7.18 (3H, m, Ph ArC$\underline{H}$), 4.93 (1H, m, C**5**-$\underline{H}$), 4.88 (1H, m, C**5**-$\underline{H}$'), 3.72 – 3.52 (2H, m, C**1**-$\underline{H_2}$), 3.33 (1H, dd, *J* = 7.5, 7.5 Hz, C**3**-$\underline{H}$), 2.24 – 2.14 (1H, m, C**2**-$\underline{H}$), 2.10 – 1.98 (1H, m, C**2**-$\underline{H}$'), 1.59 (3H, br s, C**6**-$\underline{H_3}$), 1.47 (9H, s, Boc C(C$\underline{H_3}$)$_3$); ^{13}C NMR (101 MHz, $CDCl_3$) δ 154.6 (Boc $\underline{C}$=O), 147.0 (C**4**), 142.6 (Ar$\underline{C}$), 128.6 (Ar$\underline{C}$H), 127.9 (Ar$\underline{C}$H), 126.7 (Ar$\underline{C}$H), 111.0 (C**5**), 83.5 (Boc $\underline{C}$(CH_3)$_3$), 49.9 (C**1**), 49.7 (C**3**), 30.2 (C**2**), 28.1 (Boc C($\underline{C}H_3$)$_3$), 21.2 (C**6**); ^{19}F NMR (377 MHz, $CDCl_3$) δ -136.4 – -136.6 (2F, m), -146.6 (1F, tt, *J* = 21.0, 5.0 Hz), -159.3 – -159.5 (2F, m); *m/z* (ESI^+) HRMS: Calculated for $C_{24}H_{24}F_5NNaO_4$: 508.1518. Found $[M+Na]^+$: 508.1511.

The carbon signals corresponding to the pentafluorobenzoyl group could not be resolved due to their weak intensity.

Benzyl (4-methyl-3-phenylpent-4-en-1-yl)((perfluorobenzoyl)oxy)carbamate (1c)

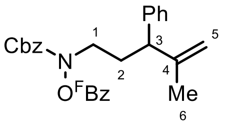

General procedure **I** was followed using CbzNHO^{F}Bz (3.87 g, 10.7 mmol), the preceding alcohol (1.71 g, 9.70 mmol), triphenyl phosphine (2.81 g, 10.7 mmol) and diisopropyl azodicarboxylate (2.11 mL, 10.7 mmol). FCC (2% EtOAc/hexane) gave title compound **1c** (3.80 g, 75%) as a colorless solid. m.p.: 66-68 °C (CH_2Cl_2/hexane); R*f*: 0.21 (2% EtOAc/hexane); ν_{max} / cm^{-1}: 2945 (m), 1784 (s), 1730 (s), 1524 (s), 1498 (s), 1172 (s); ^{1}H NMR (400 MHz, $CDCl_3$) δ 7.38 – 7.29 (5H, m, Cbz ArC$\underline{H}$), 7.29 – 7.24 (2H, m, Ph ArC$\underline{H}$), 7.22 – 7.15 (3H, m, Ph ArC$\underline{H}$), 5.20 (2H, s, Cbz C$\underline{H_2}$), 4.91 (1H, m, C**5**-$\underline{H}$), 4.86 (1H, m, C**5**-$\underline{H}$'), 3.79 – 3.60 (2H, m, C**1**-$\underline{H_2}$), 3.32 (1H, dd, *J* = 7.5, 7.5 Hz, C**3**-$\underline{H}$), 2.25 – 2.14 (1H, m, C**2**-$\underline{H}$), 2.12 – 2.00 (1H, m, C**2**-$\underline{H}$'), 1.56 (3H, br s, C**6**-$\underline{H_3}$); ^{13}C NMR (101 MHz, $CDCl_3$) δ 155.5 (Cbz C=O), 146.9 (C**4**), 142.4 (Ph Ar$\underline{C}$), 135.3 (Cbz Ar$\underline{C}$), 128.7 (Cbz ArC$\underline{H}$), 128.6 (Cbz ArC$\underline{H}$), 128.6 (Ph ArC$\underline{H}$), 128.3 (Cbz ArC$\underline{H}$), 127.8 (Ph ArC$\underline{H}$), 126.7 (Ph ArC$\underline{H}$), 111.0 (C**5**), 68.9 (Cbz C$\underline{H_2}$), 50.2 (C**1**), 49.7 (C**3**), 30.2 (C**2**), 21.1 (C**6**); ^{19}F NMR (377 MHz, $CDCl_3$) δ -135.9 – -136.1 (2F, m), -146.0 (1F, tt, *J* = 20.0, 5.5 Hz), -159.2 – -159.4 (2F, m); *m/z* (ESI^+) HRMS: Calculated for $C_{27}H_{22}F_5NNaO_4$: 542.1361. Found $[M+Na]^+$: 542.1383.

The carbon signals corresponding to the pentafluorobenzoyl group could not be resolved due to their weak intensity.

Methyl (4-methyl-3-phenylpent-4-en-1-yl)carbamate (2a)

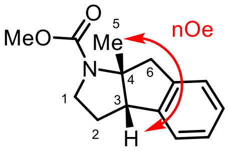

General procedure **J** was followed using cyclization substrate **1a** (44.3 mg, 0.10 mmol), $Pd_2(dba)_3$ (2.29 mg, 0.0025 mmol), CgPPh (4.38 mg, 0.015 mmol), sodium benzoate (1.44 mg, 0.01 mmol), triethylamine (1.40 µL) and dibutyl ether (1 mL). The reaction was stirred at 130 °C for 24 hours. FCC (1% acetone/toluene) gave title compound **2a** (18.5 mg, 80%) as a colorless oil. R_f: 0.38 (5% acetone/toluene); ν_{max} / cm^{-1}: 2957 (m), 1694 (s), 1444 (s), 1372 (s); *mixture of rotamers A + B (2:1)* ^{1}H NMR (400 MHz, $CDCl_3$) δ 7.18 (4H, m, ArC<u>H</u>, A + B), 3.80 (0.66H, d, *J* = 17.0 Hz, C**6**-<u>H</u>, A), 3.76 (1H, s, OC<u>H</u>$_3$, B), 3.62 (2H, s, OC<u>H</u>$_3$, A), 3.66 – 3.45 (1H, m, C**1**-<u>H</u>, A + B), 3.66 – 3.45 (0.33H, m, C**6**-<u>H</u>, B), 3.66 – 3.45 (1H, m, C**3**-<u>H</u>, A + B), 3.19 (0.33H, m, C**1**-<u>H</u>’, B), 3.08 – 2.96 (0.66H, m, C**1**-<u>H</u>’, A), 3.08 – 2.96 (0.66H, m, C**6**-<u>H</u>’, A), 3.08 – 2.96 (0.33H, m, C**6**-<u>H</u>’, B), 2.29 – 2.17 (1H, m, C**2**-<u>H</u>, A + B), 2.12 – 2.00 (1H, m, C**2**-<u>H</u>’, A + B), 1.63 (2H, s, C**5**-<u>H</u>$_3$, A), 1.56 (1H, s, C**5**-<u>H</u>$_3$, B); ^{13}C NMR (101 MHz, $CDCl_3$) δ 155.5 (carbamate <u>C</u>=O, B), 154.5 (carbamate <u>C</u>=O, A), 143.5 (Ar<u>C</u>, A), 143.3 (Ar<u>C</u>, B), 142.9 (Ar<u>C</u>, A), 142.2 (Ar<u>C</u>, B), 127.4, 127.4, 126.9, 126.7, 125.1, 124.9, 123.7, 123.6 (Ar<u>C</u>H, A + B), 71.6 (C**4**, A), 70.8 (C**4**, B), 58.5 (C**3**, B), 57.1 (C**3**, A), 52.2 (O<u>C</u>H$_3$, B), 51.8 (O<u>C</u>H$_3$, A), 48.1 (C**1**, B), 47.0 (C**1**, A), 44.9 (C**6**, B), 43.4 (C**6**, A), 28.3 (C**2**, A), 28.1 (C**2**, B), 24.9 (C**5**, B), 23.6 (C**5**, A); *m/z* (ESI$^+$) HRMS: Calculated for $C_{14}H_{17}NNaO_2$: 254.1151. Found $[M+Na]^+$: 254.1155.

Some signal broadening was observed due to amide-like resonance.

*Note – The relative stereochemistry is confirmed by the observation of an nOe effect between C**5**-H$_3$ and C**3**-H. Catalysis products **2b-n** are assigned by analogy.*

***tert*-Butyl 8a-methyl-3,3a,8,8a-tetrahydroindeno[2,1-*b*]pyrrole-1(2*H*)-carboxylate (2b)**

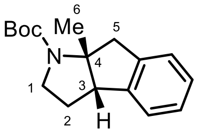

General procedure **J** was followed using cyclization substrate **1b** (48.6 mg, 0.10 mmol), $Pd_2(dba)_3$ (2.29 mg, 0.0025 mmol), CgPPh (4.38 mg, 0.015 mmol), sodium benzoate (1.44 mg, 0.01 mmol), triethylamine (1.40 µL) and dibutyl ether (1 mL). The reaction was stirred at 130 °C for 48 hours. FCC (50/49.5/0.5 toluene/CH_2Cl_2/acetone) gave title compound **2b** (14.8 mg, 54%) as a colorless oil. R_f: 0.26 (1% acetone/toluene); ν_{max} / cm^{-1}: 2971 (s), 1688 (s), 1383 (s), 1365 (s), 1171 (s); *mixture of rotamers A + B (1:1)* ^{1}H NMR (500 MHz, $CDCl_3$) δ 7.22 – 7.12 (4H, m, ArC<u>H</u>, A + B), 3.78 (0.5H, d, *J* = 17.0 Hz, C**5**-<u>H</u>, B), 3.61 – 3.42 (2.5H, m, C**1**-<u>H</u>, A + B, C**5**-<u>H</u>, A, C**3**-<u>H</u>, A + B), 3.19 (0.5H, ddd, *J*

= 11.0, 9.0, 7.0 Hz, C**1**-H’, A), 3.05 – 3.00 (0.5H, m, C**1**-H’, B), 2.99 (1H, d, *J* = 17.0 Hz, C**5**-H’, A + B), 2.24 – 2.15 (1H, m, C**2**-H, A + B), 2.05 – 1.94 (1H, m, C**2**-H’, A + B), 1.61 (1.5H, s, C**6**-H_3, B), 1.55 (1.5H, s, C**6**-H_3, A), 1.52 (4.5H, s, Boc $C(CH_3)_3$, A), 1.41 (4.5H, s, Boc $C(CH_3)_3$, B); ^{13}C NMR (126 MHz, $CDCl_3$) δ 154.3 (C=O, A), 153.6 (C=O, A), 143.8 (ArC, B), 143.6 (ArC, A), 143.2 (ArC, B), 142.4 (ArC, A), 127.4, 127.3, 126.8, 126.6, 125.1, 124.9, 123.8, 123.7 (ArCH, A + B), 79.5 (Boc $C(CH_3)_3$, A), 78.9 (Boc $C(CH_3)_3$, B), 71.0 (C**4**, B), 70.4 (C**4**, A), 58.7 (C**3**, A), 57.4 (C**3**, B), 47.6 (C**1**, A), 47.4 (C**1**, B), 45.2 (C**5**, A), 43.7 (C**5**, B), 28.8 (Boc $C(CH_3)_3$, A), 28.7 (Boc $C(CH_3)_3$, B), 28.4 (C**2**, B), 28.3 (C**2**, A), 25.2 (C**6**, A), 24.0 (C**6**, B); *m/z* (ESI^+) HRMS: Calculated for $C_{17}H_{23}NNaO_2$: 296.1621. Found $[M+Na]^+$: 296.1611.

Some signal broadening was observed due to amide-like resonance.

Benzyl 8a-methyl-3,3a,8,8a-tetrahydroindeno[2,1-*b*]pyrrole-1(2*H*)-carboxylate (2c)

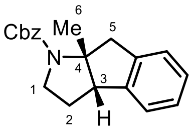

General procedure **J** was followed using cyclization substrate **1c** (52.0 mg, 0.10 mmol), $Pd_2(dba)_3$ (2.29 mg, 0.0025 mmol), CgPPh (4.38 mg, 0.015 mmol), sodium benzoate (1.44 mg, 0.01 mmol), triethylamine (1.40 μL) and dibutyl ether (1 mL). The reaction was stirred at 130°C for 48 hours. FCC (50/49.5/0.5 toluene/CH_2Cl_2/acetone) gave title compound **2c** (22.9 mg, 75%) as a colorless oil. R_f: 0.26 (1% acetone/toluene); ν_{max} / cm^{-1}: 2961 (m), 1698 (s), 1456 (m), 1404 (s), 1354 (s); *mixture of rotamers A + B (2:1)* ^{1}H NMR (500 MHz, $CDCl_3$) δ 7.44 – 7.27 (5H, m, Cbz ArCH, A + B), 7.21 – 7.09 (4H, m, Ph ArCH, A + B), 5.22 (0.33H, d, *J* = 12.5 Hz, Cbz CH, B), 5.18 (0.33H, d, *J* = 12.5 Hz, Cbz CH’, B), 5.11 (0.66H, d, *J* = 12.5 Hz, Cbz CH, A), 5.03 (0.66H, d, *J* = 12.5 Hz, Cbz CH’, A), 3.82 (0.66H, d, *J* = 17.0 Hz, C**5**-H, A), 3.69 – 3.60 (1H, m, C**1**-H, A + B), 3.56 (0.33H, d, *J* = 17.0 Hz, C**5**-H, B), 3.52 – 3.46 (1H, m, C**3**-H, A + B), 3.27 – 3.18 (0.33H, m, C**1**-H’, B), 3.09 (0.66H, ddd, *J* = 10.5, 10.5, 6.5 Hz, C**1**-H’, A), 3.05 – 2.97 (1H, m, C**5**-H’, A + B), 2.28 – 2.18 (1H, m, C**2**-H, A + B), 2.10 – 2.00 (1H, m, C**2**-H’, A + B), 1.65 (2H, s, C**6**-H_3, A), 1.57 (1H, s, C**6**-H_3, **A**); ^{13}C NMR (126 MHz, $CDCl_3$) δ 154.7 (C=O, B), 153.7 (C=O, A), 143.4 (Ph ArC, A), 143.2 (Ph ArC, B), 142.8 (Ph ArC, A), 142.1 (Ph ArC, B), 137.1 (Cbz ArC, A), 136.9 (Cbz ArC, B), 128.5, 128.4, 128.4, 128.1, 128.0, 127.9, 127.8, 127.7, 127.3, 126.8, 126.6, 126.5, 125.0, 124.8, 123.6, 123.5, (ArCH, A + B), 71.5 (C**4**, A), 70.9 (C**4**, B), 66.9 (Cbz CH_2, B), 66.2 (Cbz CH_2, A), 58.5 (C**3**, B), 57.0 (C**3**, A), 48.1 (C**1**, B), 47.0 (C**1**, A), 44.9 (C**5**, B), 43.3 (C**5**, A), 28.2 (C**2**, A), 28.1 (C**2**, B), 25.0 (C**6**, B), 23.6 (C**6**, A); *m/z* (ESI^+) HRMS: Calculated for $C_{20}H_{22}NO_2$: 308.1645. Found $[M+H]^+$: 308.1632.

Some signal broadening was observed due to amide-like resonance.

(*E*)-3-(4-Methoxyphenyl)-2-methylacrylaldehyde

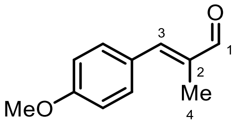

General procedure **A** was followed using *p*-anisaldehyde (5.45 g, 40.0 mmol), propionaldehyde (3.75 mL, 52 mmol) and NaOH (2.5 M, 16.0 mL) in MeOH (30 mL). FCC (8% EtOAc/hexane) gave the title compound (4.56 g, 65%) as a yellow oil – *contains 25% p-anisaldehyde starting material.* ^{1}H NMR (400 MHz, $CDCl_3$) δ 9.54 (1H, s, C**1**-H), 7.55 – 7.50 (2H, m, ArCH), 7.19 (1H, m, C**3**-H), 6.99 – 6.95 (2H, m, ArCH), 3.86 (3H, s, OCH_3), 2.08 (3H, d, J = 1.5 Hz, C**4**-H_3); ^{13}C NMR (101 MHz, $CDCl_3$) δ 195.7 (C**1**), 160.9 (ArC), 150.0 (C**3**), 136.4 (C**2**), 132.2 (ArCH), 128.1 (ArC), 114.4 (ArCH), 55.5 (OCH_3), 11.1 (C**4**).

The spectroscopic properties were consistent with the data available in the literature.[17]

(*E*)-3-(4-Methoxyphenyl)-2-methylprop-2-en-1-ol

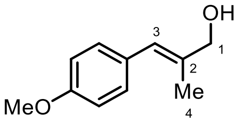

General procedure **C** was followed using the preceding aldehyde (4.56 g, 25.6 mmol), $NaBH_4$ (1.02 g, 26.9 mmol). FCC (20% EtOAc/hexane) gave the title compound (4.48 g, 99%) as a colorless oil – *contains 25% anisyl alcohol impurity*. ^{1}H NMR (400 MHz, $CDCl_3$) δ 7.25 – 7.20 (2H, m, ArCH), 6.90 – 6.85 (2H, m, ArCH), 6.45 (1H, m, C**3**-H), 4.17 (2H, s, C**1**-H_2), 3.81 (3H, s, OCH_3), 1.90 (3H, br s, C**4**-H_3); ^{13}C NMR (101 MHz, $CDCl_3$) δ 158.3 (ArC), 136.2 (ArC), 130.2 (C**2**), 130.2 (ArCH), 124.9 (C**3**), 113.7 (ArCH), 69.5 (C**1**), 55.4 (OCH_3), 15.5 (C**4**).

The spectroscopic properties were consistent with the data available in the literature.[18]

3-(4-Methoxyphenyl)-4-methylpent-4-enoic acid

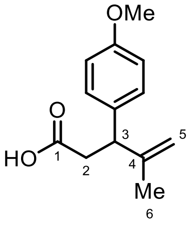

General procedure **E** was followed using the preceding alcohol (4.48 g, 25.4 mmol), pivalic acid (519 mg, 5.08 mmol) and triethyl orthoacetate (32.6 mL, 178 mmol) for a single cycle. The subsequent hydrolysis was stirred at 60 °C for 16 hours. The title compound was obtained (678 mg, 12%) as colorless solid. m.p.: 110-112 °C (CH_2Cl_2/hexane); ν_{max} / cm^{-1}: 2956 (m), 1714 (s), 1691 (s), 1512 (s),

1252 (s); ^{1}H NMR (400 MHz, $CDCl_3$) δ 7.16 – 7.11 (2H, m, ArCH), 6.85 – 6.81 (2H, m, ArCH), 4.90 (1H, m, C**5**-H), 4.87 (1H, m, C**5**-H'), 3.79 (3H, s, OCH_3), 3.72 (1H, dd, *J* = 8.0, 8.0 Hz, C**3**-H), 2.87 (1H, dd, *J* = 15.5, 8.0 Hz, C**2**-H), 2.72 (1H, dd, *J* = 15.5, 8.0 Hz, C**2**-H'), 1.61 (3H, br s, C**6**-H_3); ^{13}C NMR (101 MHz, $CDCl_3$) δ 176.5 (C**1**), 158.6 (ArC), 146.9 (C**4**), 133.9 (ArC), 128.8 (ArCH), 114.0 (ArCH), 110.4 (C**5**), 55.4 (OCH_3), 47.6 (C**3**), 39.0 (C**2**), 21.8 (C**6**); *m/z* (ESI^+) HRMS: Calculated for $C_{13}H_{16}NaO_3$: 243.0992. Found $[M+Na]^+$: 243.0995.

3-(4-Methoxyphenyl)-4-methylpent-4-en-1-ol

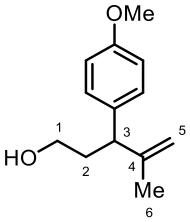

General procedure **F** was followed using the preceding carboxylic acid (678 mg, 3.08 mmol), $LiAlH_4$ (2M in THF, 3.08 mL) and anhydrous THF at room temperature for 4 hours. The title compound (621 mg, 98%) was obtained as a colorless oil. ^{1}H NMR (400 MHz, $CDCl_3$) δ 7.16 – 7.11 (2H, m, ArCH), 6.86 – 6.81 (2H, m, ArCH), 4.92 (1H, m, C**5**-H), 4.83 (1H, m, C**5**-H), 3.79 (3H, s, OCH_3), 3.66 – 3.53 (2H, m, C**1**-H_2), 3.36 (1H, dd, *J* = 7.5, 7.5 Hz, C**3**-H), 2.15 – 2.06 (1H, m, C**2**-H), 1.99 – 1.90 (1H, m, C**2**-H'), 1.58 (1H, br s, C**6**-H_3), 1.27 (1H, t, *J* = 5.5 Hz, OH); ^{13}C NMR (101 MHz, $CDCl_3$) δ 158.3 (ArC), 148.3 (C**4**), 135.1 (ArC), 128.8 (ArCH), 113.9 (ArCH), 110.3 (C**5**), 61.5 (C**1**), 55.3 (OCH_3), 48.3 (C**3**), 36.0 (C**2**), 21.1 (C**6**).

The spectroscopic properties were consistent with the data available in the literature.[16]

Benzyl(3-(4-methoxyphenyl)-4-methylpent-4-en-1-yl)((perfluorobenzoyl)oxy)carbamate (1d)

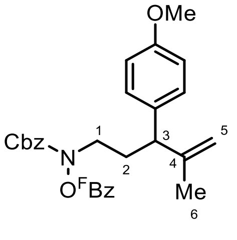

General procedure **I** was followed using CbzNHO^{F}Bz (1.18 g, 3.27 mmol), the preceding alcohol (612 mg, 2.97 mmol), triphenylphosphine (858 mg, 3.27 mmol) and diisopropyl azodicarboxylate (0.644 mL, 3.27 mmol). FCC (5% EtOAc/hexane) gave title compound **1d** (1.45 g, 89%) as a colorless oil. R_f: 0.25 (5% EtOAc/hexane); ν_{max} / cm^{-1}: 2946 (m), 1785 (s), 1737 (m), 1524 (s), 1509 (s), 1175 (s); ^{1}H NMR (400 MHz, $CDCl_3$) δ 7.39 – 7.28 (5H, m, Cbz ArCH), 7.11 – 7.06 (2H, m, ArCH), 6.82 – 6.77 (2H, m, ArCH), 5.20 (2H, s, Cbz CH_2), 4.88 (1H, m, C**5**-H), 4.83 (1H, m, C**5**-H'), 3.78 (3H, s, OCH_3), 3.76 – 3.58 (2H, m, C**1**-H_2), 3.27 (1H, dd, *J* = 7.5, 7.5 Hz, C**3**-H), 2.22 – 2.13 (1H, m, C**2**-H), 2.08 – 1.97 (2H,

m, C**2**-H'), 1.56 (3H, br s, C**6**-H_3); ^{13}C NMR (101 MHz CDCl$_3$) δ 158.4 (ArC), 155.5 (Cbz C=O), 147.3 (C**4**), 135.3 (Cbz ArC), 134.4 (ArC), 128.8 (ArCH), 128.7 (Cbz ArCH), 128.6 (Cbz ArCH), 128.3 (Cbz ArCH), 114.0 (ArCH), 110.6 (C**5**), 68.9 (Cbz CH_2), 55.4 (OCH_3), 50.2 (C**1**), 48.8 (C**3**), 30.4 (C**2**), 21.1 (C**6**); ^{19}F NMR (377 MHz, CDCl$_3$) δ -135.9 – -136.1 (2F, m), -146.1 (1F, tt, *J* = 21.0, 5.5 Hz), -159.2 – -159.4 (2F, m); *m/z* (ESI$^+$) HRMS: Calculated for $C_{28}H_{24}F_5NNaO_5$: 572.1467. Found [M+Na]$^+$: 572.1488.

The carbon signals corresponding to the pentafluorobenzoyl group could not be resolved due to their weak intensity.

Benzyl 6-methoxy-8a-methyl-3,3a,8,8a-tetrahydroindeno[2,1-*b*]pyrrole-1(2*H*)-carboxylate (2d)

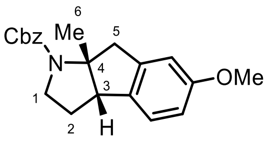

General procedure **J** was followed using cyclization substrate **1d** (55.0 mg, 0.10 mmol), $Pd_2(dba)_3$ (2.29 mg, 0.0025 mmol), CgPPh (4.38 mg, 0.015 mmol), sodium benzoate (1.44 mg, 0.01 mmol), triethylamine (1.40 µL) and dibutyl ether (1 mL). The reaction was stirred at 130 °C for 48 hours. FCC (10% EtOAc/hexane) gave title compound **2d** (26.3 mg, 78%) as a colorless oil. R_f: 0.26 (10% EtOAc/hexane); ν_{max} / cm^{-1}: 2960 (m), 1695 (s), 1493 (s), 1404 (s), 1354 (s); *mixture of rotamers A + B (2:1)* ^{1}H NMR (400 MHz, CDCl$_3$) δ 7.44 – 7.27 (5H, m, Cbz ArCH, A + B), 7.06 (1H, d, *J* = 8.0 Hz, ArCH, A + B), 6.77 – 6.64 (2H, m, ArCH, A + B), 5.24 – 5.15 (0.66H, m, Cbz CH_2, B), 5.12 (0.66H, d, *J* = 12.5 Hz, Cbz CH, A), 5.03 (0.66H, d, *J* = 12.5 Hz, Cbz CH', A), 3.82 – 3.75 (3.66H, m, OCH_3, A + B, C**5**-H, A), 3.69 – 3.59 (1H, m, C**1**-H, A + B), 3.52 (0.33H, d, *J* = 17.0 Hz, C**5**-H, B), 3.43 (1H, m, C**3**-H, A + B), 3.23 (0.33H, ddd, *J* = 10.0, 10.0, 6.5 Hz, C**1**-H', B), 3.10 (0.66H, ddd, *J* = 10.0, 10.0, 6.5 Hz, C**1**-H', A), 2.99 (0.66H, d, *J* = 17.0 Hz, C**5**-H', A), 2.96 (0.33H, d, *J* = 17.0 Hz, C**5**-H', B), 2.27 – 2.13 (1H, m, C**2**-H, A + B), 2.06 – 1.95 (1H, m, C**2**-H', A + B), 1.63 (2H, s, C**6**-H_3, A), 1.56 (1H, s, C**6**-H_3, B); ^{13}C NMR (101 MHz, CDCl$_3$) δ 159.6 (ArC, A + B), 154.8 (C=O, B), 153.9 (C=O, A), 144.4 (ArC, A), 143.7 (ArC, B), 137.2 (Cbz ArC, A), 137.0 (Cbz ArC, B), 135.5 (ArC, A), 135.3 (ArC, B), 128.6, 128.5, 128.1, 127.9, 127.8 (Cbz ArCH, A + B), 124.3 (ArCH, B), 124.2 (ArCH, A), 113.0 (ArCH, A + B), 110.1 (ArCH, A + B), 72.2 (C**4**, A), 71.5 (C**4**, B), 67.0 (Cbz CH_2, B), 66.3 (Cbz CH_2, A), 57.9 (C**3**, B), 56.4 (C**3**, A), 55.5 (OCH_3, A), 55.4 (OCH_3, B), 48.2 (C**1**, B), 47.1 (C**1**, A), 45.1 (C**5**, B), 43.5 (C**5**, A), 28.4 (C**2**, A), 28.3 (C**2**, B), 25.1 (C**6**, A), 23.6 (C**6**, A); *m/z* (ESI$^+$) HRMS: Calculated for $C_{21}H_{24}NO_3$: 338.1751. Found [M+H]$^+$: 338.1763.

Some signal broadening was observed due to amide-like resonance.

(*E*)-3-(4-(*tert*-Butyl)phenyl)-2-methylacrylaldehyde

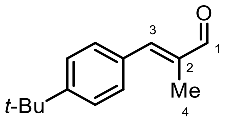

General procedure **A** was followed using 4-*tert*-butyl benzaldehyde (3.09 mL, 18.5 mmol), and propionaldehyde (1.33 mL, 18.5 mmol). FCC (1-2% EtOAc/hexane) gave the title compound (2.62 g, 70%) as a colorless solid. m.p.: 66 - 67 °C (CH_2Cl_2/hexane) [Lit.: 69 °C][19]; ^{1}H NMR (400 MHz, $CDCl_3$) δ 9.58 (1H, s, C**1**-H), 7.52 – 7.46 (4H, m, ArCH), 7.24 (1H, m, C**3**-H), 2.09 (3H, d, *J* = 1.5 Hz, C**4**-H_3), 1.35 (9H, s, *t*-Bu C(CH_3)$_3$); ^{13}C NMR (101 MHz, $CDCl_3$) δ 195.8 (C**1**), 153.3 (ArC), 150.1 (C**3**), 137.8 (C**2**), 132.6 (ArC), 130.2 (ArCH), 125.9 (ArCH), 35.0 (*t*-Bu C(CH_3)$_3$), 31.3 (*t*-Bu C(CH_3)$_3$), 11.1 (C**4**).

The spectroscopic properties were consistent with the data available in literature.[20]

(*E*)-3-(4-(*tert*-Butyl)phenyl)-2-methylprop-2-en-1-ol

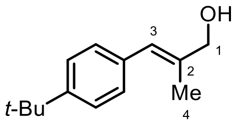

General procedure **C** was followed using the preceding aldehyde (2.62 g, 12.8 mmol) and $NaBH_4$ (509 mg, 13.5 mmol) to give the title compound (2.44 g, 93%) as a colorless oil without purification. ^{1}H NMR (400 MHz, $CDCl_3$) δ 7.38 – 7.34 (2H, m, ArCH), 7.25 – 7.22 (2H, m, ArCH), 6.49 (1H, s, C**3**-H), 4.19 (2H, s, C**1**-H_2), 1.93 (3H, br s, C**4**-H_3), 1.33 (9H, s, *t*-Bu C(CH_3)$_3$); ^{13}C NMR (101 MHz, $CDCl_3$) δ 149.5 (ArC), 137.1 (C**2**), 134.8 (ArC), 128.7 (ArCH), 125.2 (ArCH), 125.1 (C**3**), 69.4 (C**1**), 34.7 (*t*-Bu C(CH_3)$_3$), 31.5 (*t*-Bu C(CH_3)$_3$), 15.5 (C**4**).

The spectroscopic properties were consistent with the data available in literature.[18]

3-(4-(*tert*-Butyl)phenyl)-4-methylpent-4-enoic acid

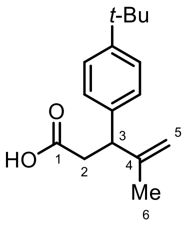

General procedure **E** was followed using the preceding alcohol (548 mg, 2.68 mmol), triethyl orthoacetate (3.45 mL, 18.8 mmol) and pivalic acid (27.4 mg, 0.268 mmol) for 3 cycles. The subsequent hydrolysis was stirred at 60 °C for 16 hours. The title compound (482 mg, 73%) was obtained as an orange oil (*contains AcOH impurity*). ν_{max} / cm^{-1}: 2964 (s), 1708 (s), 1412 (m), 1269 (m), 894 (m); ^{1}H NMR (400 MHz, $CDCl_3$) δ 7.32 - 7.28 (2H, m, ArCH), 7.16 - 7.12 (2H, m, ArCH), 4.93 (1H, m, C**5**-H), 4.88 (1H, m, C**5**-H'), 3.76 (1H, dd, *J* = 8.0, 8.0 Hz, C**3**-H), 2.89 (1H, dd, *J* = 15.5, 8.0 Hz, C**2**-H), 2.75 (1H, dd, *J* = 15.5, 8.0 Hz, C**2**-H'), 1.62 (3H, s, C**6**-H_3), 1.30 (9H, s, *t*-Bu C(CH_3)$_3$); ^{13}C NMR (101

MHz, $CDCl_3$) δ 176.7 (C**1**), 149.6 (ArC), 146.7 (C**4**), 138.6 (ArC), 127.2 (ArCH), 125.4 (ArCH), 110.5 (C**5**), 47.8 (C**3**), 38.7 (C**2**), 34.4 (*t*-Bu C(CH_3)$_3$), 31.4 (*t*-Bu C(CH_3)$_3$), 21.6 (C**6**); m/z (APCI$^+$) HRMS: Calculated for $C_{16}H_{23}O_2$: 247.1693. Found $[M+H]^+$ 247.1696.

3-(4-(*tert*-Butyl)phenyl)-4-methylpent-4-en-1-ol

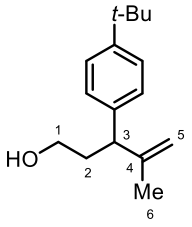

General procedure **F** was followed using the preceding carboxylic acid (879 mg, 3.57 mmol), $LiAlH_4$ (2M in THF, 3.57 mL) and anhydrous THF at 0 °C for 3 hours. FCC (5-15% EtOAc/hexane) gave the title compound (623 mg, 75%) as colorless oil. ν_{max} / cm^{-1}: 3325 (m), 2963 (m), 1645 (m), 1509 (m), 1046 (m), 891 (m); ^{1}H NMR (400 MHz, $CDCl_3$) δ 7.31 - 7.29 (2H, m, ArCH), 7.15 - 7.13 (2H, m, ArCH), 4.94 (1H, m, C**5**-H), 4.84 (1H, m, C**5**-H'), 3.65 - 3.56 (2H, m, C**1**-H_2), 3.39 (1H, dd, *J* = 7.5, 7.5 Hz, C**3**-H), 2.16 - 2.08 (1H, m, C**2**-H), 2.02 - 1.92 (1H, m, C**2**-H'), 1.59 (3H, s, C**6**-H_3), 1.30 (9H, s, *t*-Bu C(CH_3)$_3$); ^{13}C NMR (101 MHz, $CDCl_3$) δ 149.3 (ArC), 148.2 (C**4**), 139.9 (ArC), 127.5 (ArCH), 125.4 (ArCH), 110.6 (C**5**), 61.6 (C**1**), 48.8 (C**3**), 35.8 (C**2**), 34.5 (*t*-Bu C(CH_3)$_3$), 31.5 (*t*-Bu C(CH_3)$_3$), 21.0 (C**6**); m/z (APCI$^+$) HRMS: Calculated for $C_{16}H_{25}O$: 233.1900. Found $[M+H]^+$ 233.1902.

Benzyl (3-(4-(*tert*-butyl)phenyl)-4-methylpent-4-en-1-yl)((perfluorobenzoyl)oxy)carbamate (1e)

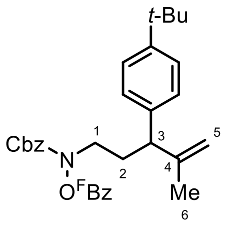

General procedure **I** was followed using the preceding alcohol (159 mg, 0.684 mmol), CbzNHO^{F}Bz (272 mg, 0.753 mmol), triphenylphosphine (198 mg, 0.753 mmol) and diisopropyl azodicarboxylate (135 µL, 0.684 mmol). FCC (3% EtOAc/hexane) gave title compound **1e** (310 mg, 79%) as a colorless oil. ν_{max} / cm^{-1}: 2965 (m), 1733 (m), 1506 (s), 1327 (s), 1172 (s), 1004 (m), 907 (m), 698 (m); ^{1}H NMR (400 MHz, $CDCl_3$) δ 7.37 – 7.32 (5H, m, Cbz ArCH), 7.28 – 7.26 (2H, m, ArCH), 7.10 – 7.08 (2H, m, ArCH), 5.21 (2H, s, Cbz CH_2), 4.90 (1H, m, C**5**-H), 4.84 (1H, m, C**5**-H'), 3.77 – 3.62 (2H, m, C**1**-H_2), 3.30 (1H, dd, *J* = 7.5, 7.5 Hz, C**3**-H), 2.23 - 2.14 (1H, m, C**2**-H), 2.11 - 2.02 (1H, m, C**2**-H'), 1.57 (3H, s, C**6**-H_3), 1.29 (9H, s, *t*-Bu C(CH_3)$_3$); ^{13}C NMR (101 MHz, $CDCl_3$) δ 155.5 (Cbz C=O), 149.5 (ArC), 147.1 (C**4**), 139.2 (ArC), 135.3 (Cbz ArC), 128.7 (Cbz ArCH), 128.6 (Cbz ArCH), 128.3 (Cbz ArCH), 127.4 (ArCH), 125.4 (ArCH), 110.9 (C**5**), 68.9 (Cbz CH_2), 50.3 (C**1**), 49.3 (C**3**), 34.5 (*t*-Bu C(CH_3)$_3$), 31.5 (*t*-Bu C(CH_3)$_3$), 30.2 (C**2**), 21.0 (C**6**); *mixture of rotamers A + B (9:1)* ^{19}F NMR (377 MHz, $CDCl_3$)

δ -135.9 – -136.1 (2F, m, A + B), -146.1 (0.9F, tt, *J* = 21.0, 5.5 Hz, A), -146.3 (0.1F, tt, *J* = 21.0, 5.5 Hz, B), -159.2 – -159.4 (1.8F, m, A), -159.4 – -159.6 (0.2F, m, B).m/z (ESI$^+$) HRMS: Calculated for $C_{31}H_{30}F_5NO_4Na$: 598.1987. Found [M+Na]$^+$ 598.1970.

The ^{13}C signals corresponding to the pentafluorobenzoyl group could not be resolved due to their weak intensity.

Some signal broadening was observed due to amide-like resonance.

Benzyl 6-(*tert*-butyl)-8a-methyl-3,3a,8,8a-tetrahydroindeno[2,1-*b*]pyrrole-1(2*H*)-carboxylate (2e)

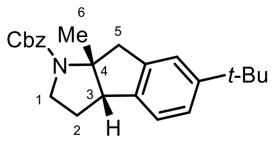

General procedure **J** was followed using cyclization substrate **1e** (57.6 mg, 0.10 mmol), Pd_2dba_3 (2.29 mg, 0.0025 mmol), CgPPh (4.38 mg, 0.015 mmol), sodium benzoate (1.44 mg, 0.01 mmol), triethylamine (1.40 µL) and dibutylether (1 mL). The reaction was stirred at 130 °C for 72 hours. FCC (5% EtOAc/hexane) gave title compound **2e** (23.5 mg, 65%) as a colorless oil. R_f: 0.2 (5% EtOAc/hexane); ν_{max} / cm^{-1}: 2962 (m), 1699 (s), 1404 (s), 1217 (m), 1049 (m), 697 (m); *Mixture of rotamers A + B (3:2)* ^{1}H NMR (500 MHz, $CDCl_3$) δ 7.44 – 7.28 (5H, m, Cbz ArCH, A + B), 7.22 (1H, m, ArCH, A + B), 7.19 (0.6H, br s, ArCH, A), 7.13 (0.4H, br s, ArCH, B), 7.09 (1H, br d, *J* = 8.0 Hz, A + B), 5.23 (0.4H, d, *J* = 12.5 Hz, Cbz CH, B), 5.18 (0.4H, d, *J* = 12.5 Hz, Cbz CH’, B), 5.13 (0.6H, d, *J* = 12.5 Hz, Cbz CH, A), 5.04 (0.6H, d, *J* = 12.5 Hz, Cbz CH’, A), 3.79 (0.6H, d, *J* = 17.0 Hz, C**5**-H, A), 3.68 – 3.61 (1H, m, C**1**-H, A + B), 3.55 (0.4H, d, *J* = 17.0 Hz, C**5**-H, B), 3.48 – 3.44 (1H, m, C**3**-H, A + B), 3.22 (0.4H, ddd, *J* = 10.0, 10.0, 7.0 Hz, C**1**-H’, B), 3.11 (0.6H, ddd, *J* = 10.0, 10.0, 7.0 Hz, C**1**-H’, A), 3.02 (0.6H, d, *J* = 17.0 Hz, C**5**-H’, A), 3.98 (0.4H, d, *J* = 17.0 Hz, C**5**-H’, B), 2.26 - 2.17 (1H, m, C**2**-H, A + B), 2.09 - 2.01 (1H, m, C**2**-H’, A + B), 1.64 (1.8H, s, C**6**-H_3, A), 1.56 (1.2H, s, C**6**-H_3, B), 1.33 – 1.30 (9H, m, *t*-Bu $C(CH_3)_3$, A + B); ^{13}C NMR (126 MHz, $CDCl_3$) δ 154.9 (C=O, B), 153.9 (C=O, A), 150.7 (ArC, B), 150.6 (ArC, A), 142.6 (ArC, A), 142.0 (ArC, B), 140.5 (ArC, A), 140.3 (ArC, B), 137.3 (Cbz ArC, A), 137.0 (Cbz ArC, B), 128.7, 128.5, 128.1, 128.0, 127.9, 127.8 (Cbz ArCH, A + B), 124.1 (ArCH, B), 123.9 (ArCH, A), 123.1 (ArCH, B), 123.1 (ArCH, A), 122.0 (ArCH, A), 121.8 (ArCH, B), 71.9 (C**4**, A), 71.3 (C**4**, B), 67.1 (Cbz CH_2, B), 66.2 (Cbz CH_2, A), 58.3 (C**3**, B), 56.9 (C**3**, A), 48.3 (C**1**, B), 47.1 (C**1**, A), 45.1 (C**5**, B), 43.6 (C**5**, A), 34.7 (*t*-Bu $C(CH_3)_3$, A + B), 31.7 (*t*-Bu $C(CH_3)_3$, A + B), 28.3 (C**2**, A), 28.1 (C**2**, B), 25.20 (C**6**, B), 23.79 (C**6**, A); *m/z* (ESI$^+$) HRMS: Calculated for $C_{24}H_{30}NO_2$: 364.2271. Found [M+H]$^+$ 364.2269.

Some signal broadening was observed due to amide-like resonance.

(*E*)-2-Methyl-3-(4-(trifluoromethyl)phenyl)acrylaldehyde

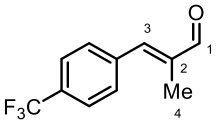

General procedure **A** was followed using 4-(trifluoromethyl)benzaldehyde (4.00 g, 23.0 mmol), propionaldehyde (2.16 mL, 29.9 mmol) and NaOH (2.5 M, 9.20 mL) in MeOH (18 mL). FCC (30% hexane/toluene) gave the title compound (1.53 g, 31%) as a pale yellow oil. R*f*: 0.26 (30% hexane/toluene); ν_{max} / cm^{-1}: 2831 (m), 1684 (s), 1322 (s); ^{1}H NMR (400 MHz, $CDCl_3$) δ 9.63 (1H, s, C**1**-H), 7.71 (2H, d, *J* = 8.0 Hz, ArCH), 7.62 (2H, d, *J* = 8.0 Hz, ArCH), 7.30 (1H, s, C**3**-H), 2.08 (3H, br s, C**4**-H_3); ^{13}C NMR (101 MHz, $CDCl_3$) δ 195.2 (C**1**), 147.5 (C**3**), 140.4 (C**2**), 138.6 (ArC), 131.2 (q, *J* = 32.5 Hz, ArC), 130.1 (ArCH), 125.9 – 125.7 (ArCH), 122.6 (CF_3), 11.1 (C**4**); ^{19}F NMR (377 MHz, $CDCl_3$) δ -62.9 (3F, s); *m/z* (ESI^+) HRMS: Calculated for $C_{11}H_9F_3NaO$: 237.0500. Found $[M+Na]^+$: 237.0490.

The splitting associated with the quaternary CF_3 signal could not be resolved due to its weak intensity.

(*E*)-2-Methyl-3-(4-(trifluoromethyl)phenyl)prop-2-en-1-ol

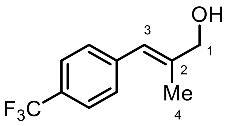

General procedure **C** was followed using the preceding aldehyde (1.89 g, 8.82 mmol) and $NaBH_4$ (351 mg, 9.27 mmol) to give the title compound (1.81 g, 95%) as an off-white solid without purification. R*f*: 0.16 (15% EtOAc/hexane); ^{1}H NMR (400 MHz, $CDCl_3$) δ 7.59 (2H, d, *J* = 8.0 Hz, ArCH), 7.37 (2H, d, *J* = 8.0 Hz, ArCH), 6.57 (1H, br s, C**3**-H), 4.22 (2H, s, C**1**-H_2), 1.89 (3H, br s, C**4**-H_3); ^{13}C NMR (101 MHz, $CDCl_3$) δ 141.4 (ArC), 140.0 (C**2**), 129.2 (ArCH), 125.3 – 125.1 (ArCH), 123.6 (ArC), 123.5 (C**3**), 68.6 (C**1**), 15.4 (C**4**); ^{19}F NMR (377 MHz, $CDCl_3$) δ -62.4 (3F, s).

The signal associated with the quaternary CF_3 could not be resolved due to its weak intensity.

The spectroscopic properties were consistent with the data available in the literature.[18]

4-Methyl-3-(4-(trifluoromethyl)phenyl)pent-4-enoic acid

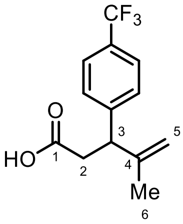

General procedure **E** was followed using the preceding alcohol (1.79 g, 8.28 mmol), pivalic acid (84.6 mg, 0.83 mmol) and triethyl orthoacetate (15.0 mL, 82.8 mmol) for 2 cycles. The subsequent hydrolysis was stirred at 60°C for 5 hours. The title compound was obtained (1.64 g, 77%) as a yellow solid. m.p.: 82 – 84 °C (CH_2Cl_2/hexane); ν_{max} / cm^{-1}: 2974 (m), 1711 (m), 1352 (s), 1124 (s); ^{1}H NMR (500 MHz, $CDCl_3$) δ 7.55 (2H, d, *J* = 8.0 Hz, ArCH), 7.34 (2H, d, *J* = 8.0 Hz, ArCH), 4.95 – 4.93 (2H, m, C**5**-H_2), 3.83 (1H, dd, *J* = 8.0, 8.0 Hz, C**3**-H), 2.91 (1H, dd, *J* = 16.0, 7.5 Hz, C**2**-H), 2.75 (1H, dd, *J* = 16.0, 7.5 Hz, C**2**-H'), 1.60 (3H, br s, C**6**-H_3); ^{13}C NMR (126 MHz, $CDCl_3$) δ 177.4 (C**1**), 145.9 (ArC), 145.7 (C**4**), 129.3 (q, *J* = 32.5 Hz, ArC), 128.2 (ArCH), 125.6 (q, *J* = 4.0 Hz, ArCH), 124.2 (q, *J* = 269.0 Hz, CF_3), 111.6 (C**5**), 48.1 (C**3**), 38.8 (C**2**), 21.8 (C**6**); ^{19}F NMR (377 MHz, $CDCl_3$) δ -62.5 (3F, s); *m/z* (ESI$^+$) HRMS: Calculated for $C_{13}H_{13}F_3NaO_2$: 281.0760. Found $[M+Na]^+$: 281.0752.

4-Methyl-3-(4-(trifluoromethyl)phenyl)pent-4-en-1-ol

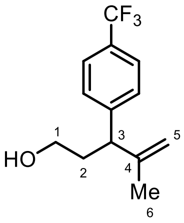

General procedure **F** was followed using the preceding carboxylic acid (678 mg, 3.08 mmol), $LiAlH_4$ (2M in THF, 3.08 mL) and anhydrous THF at room temperature for 4 hours. The title compound (621 mg, 98%) was obtained as a colorless oil. ν_{max} / cm^{-1}: 3328 (br s), 2942 (m), 1325 (s), 1164 (s), 1123 (s), 1068 (s); ^{1}H NMR (500 MHz, $CDCl_3$) δ 7.57 – 7.52 (2H, m, ArCH), 7.36 – 7.32 (2H, m, ArCH), 4.98 (1H, m, C**5**-H), 4.91 (1H, m, C**5**-H'), 3.67 – 3.48 (3H, m, C**1**-H_2 + C**3**-H), 2.16 (1H, dtd, *J* = 14.0, 7.0, 6.5 Hz, C**2**-H'), 1.97 (1H, ddt, *J* = 14.0, 8.0, 6.0 Hz, C**2**-H'), 1.58 (3H, br s, C**6**-H_3); ^{13}C NMR (126 MHz $CDCl_3$) δ 147.3 (ArC), 146.9 (C**4**), 128.9 (q, *J* = 32.5 Hz, ArC), 128.3 (ArCH), 125.5 (q, *J* = 3.5 Hz, ArCH), 123.3 (CF_3), 111.5 (C**5**), 61.0 (C**1**), 48.8 (C**3**), 35.7 (C**2**), 21.1 (C**6**); ^{19}F NMR (377 MHz, $CDCl_3$) δ -62.4 (3F, s); *m/z* (ESI$^+$) HRMS: Calculated for $C_{13}H_{15}F_3NaO$: 267.0967. Found $[M+Na]^+$: 267.0974.

The splitting associated with the quaternary CF_3 signal could not be resolved due to its weak intensity.

Benzyl (4-methyl-3-(4-(trifluoromethyl)phenyl)pent-4-en-1-yl) ((perfluorobenzoyl)oxy) carbamate (1f)

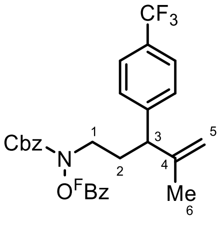

General procedure **I** was followed using CbzNHO^{F}Bz (795 mg, 2.20 mmol), preceding alcohol (489 mg, 2.00 mmol), triphenylphosphine (577 mg, 2.20 mmol) and diisopropyl azodicarboxylate (0.433 mL, 2.20 mmol). FCC (5% EtOAc/hexane) gave title compound **1f** (914 mg, 78%) as a colorless solid. m.p.: 64 – 65 °C (CH_2Cl_2/hexane); R_f: 0.22 (5% EtOAc/hexane); ν_{max} / cm^{-1}: 2972 (m), 1784 (s), 1731 (s), 1524 (s), 1499 (s), 1324 (s); ^{1}H NMR (500 MHz, $CDCl_3$) δ 7.51 (2H, d, *J* = 8.0 Hz, ArCH), 7.38 – 7.27 (7H, m, Cbz 5 × ArCH + 2 × ArCH), 5.20 (2H, s, Cbz CH_2), 4.94 (1H, m, C**5**-H), 4.91 (1H, m, C**5**-H'), 3.75 – 3.63 (2H, m, C**1**-H_2), 3.42 (1H, dd, *J* = 7.5, 7.5 Hz, C**3**-H), 2.21 (1H, dtd, *J* = 14.0, 8.0, 6.5 Hz, C**2**-H), 2.04 (1H, dtd, *J* = 14.0, 8.0, 6.5 Hz, C**2**-H'), 1.55 (3H, br s, C**6**-H_3); ^{13}C NMR (126 MHz $CDCl_3$) δ 155.5 (Cbz C=O), 146.6 (ArC), 146.0 (C**4**), 135.2 (Cbz ArC), 129.1 (q, *J* = 32.5 Hz, ArC), 128.7 (Cbz 4 × ArCH), 128.4 (Cbz ArCH), 128.2 (ArCH), 125.6 (q, *J* = 4.0 Hz, ArCH), 124.3 (q, *J* = 271 Hz, CF_3), 111.8 (C**5**), 69.1 (Cbz CH_2), 49.9 (C**1**), 49.3 (C**3**), 30.2 (C**2**), 21.2 (C**6**); ^{19}F NMR (377 MHz, $CDCl_3$) δ -62.4 (3F, s), -136.0 – -136.2 (2F, m), -145.8 (1F, tt, *J* = 21.0, 5.5 Hz), -159.1 – -159.3 (2F, m); *m/z* (ESI$^+$) HRMS: Calculated for $C_{28}H_{21}F_8NNaO_4$: 610.1235. Found $[M+Na]^+$: 610.1241.

The carbon signals corresponding to the pentafluorobenzoyl group could not be resolved due to their weak intensity.

Benzyl 8a-methyl-6-(trifluoromethyl)-3,3a,8,8a-tetrahydroindeno[2,1-*b*]pyrrole-1(2*H*)-carboxylate (2f)

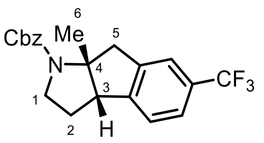

General procedure **J** was followed using cyclization substrate **1f** (58.8 mg, 0.10 mmol), $Pd_2(dba)_3$ (2.29 mg, 0.0025 mmol), CgPPh (4.38 mg, 0.015 mmol), sodium benzoate (1.44 mg, 0.01 mmol), triethylamine (1.40 μL) and dibutyl ether (1 mL). The reaction was stirred at 130 °C for 48 hours. FCC (10% EtOAc/hexane) gave title compound **2f** (27.0 mg, 72%) as a colorless oil. R_f: 0.28 (10% EtOAc/hexane); ν_{max} / cm^{-1}: 2970 (m), 1697 (s), 1405 (s), 1323 (s), 1119 (s); *mixture of rotamers A + B (2:1)* ^{1}H NMR (500 MHz, $CDCl_3$) δ 7.48 – 7.22 (8H, m, ArCH, A + B), 5.23 – 5.16 (0.66H, m, Cbz CH_2, B), 5.12 (0.66H, d, *J* = 12.5 Hz, Cbz CH, A), 5.03 (0.66H, d, *J* = 12.5 Hz, Cbz CH', A), 3.89 (0.66H, d, *J* = 17.3 Hz, C**5**-H, A), 3.71 – 3.58 (1.33H, m, C**5**-H, B, C**1**-H, A + B), 3.54 – 3.49 (1H, m, C**3**-H, A + B), 3.21 (0.33H, ddd, *J* = 11.0, 9.5, 6.5 Hz, C**1**-H', B), 3.10 – 2.99 (1.66H, m, C**5**-H', A + B, C**1**-H', A), 2.33 – 2.22 (1H, m, C**2**-H, A + B), 2.13 – 2.01 (1H, m, C**2**-H', A + B), 1.66 (2H, s, C**6**-H_3, A), 1.58 (1H, s, C**6**-H_3, B); ^{13}C NMR (126 MHz $CDCl_3$) δ 154.7 (C=O, B), 153.8 (C=O, A), 147.7 (ArC, A), 147.4 (ArC, B), 143.7 (ArC, A), 143.1 (ArC, B), 137.0 (Cbz ArC, A), 136.8 (Cbz ArC, B), 130.3 – 129.4 (ArC, A + B), 128.7, 128.6, 128.2, 128.0, 127.9 (ArCH, A + B), 124.5 (q, *J* = 271 Hz, CF_3, A), 124.2, 124.2, 124.1, 124.0, 124.0 (ArCH, A + B), 122.1 – 121.9 (ArCH, A + B), 71.8 (C**4**, A), 71.1 (C**4**, B), 67.2 (Cbz CH_2, B), 66.4 (Cbz CH_2, A), 58.4 (C**3**, B), 56.9 (C**3**, A), 48.2 (C**1**, B), 47.0 (C**1**,

A), 44.7 (C**5**, B), 43.2 (C**5**, A), 28.2 (C**2**, A), 28.0 (C**2**, B), 25.0 (C**6**, B), 23.5 (C**6**, A); ^{19}F NMR (377 MHz, $CDCl_3$) δ -62.1 (2F, s, A), -62.1 (1F, s, B); *m/z* (ESI^+) HRMS: Calculated for $C_{21}H_{21}F_3NO_2$: 376.1519. Found $[M+H]^+$: 376.1523.

The aromatic region in the ^{13}C NMR spectrum is complex due to amide-like resonance and ^{19}F splitting, ranges have been quoted for broad signals split by ^{19}F. Due to its weak signal intensity, the CF_3 quartet was only resolvable for the major rotamer.

(*E*)-2-Methyl-3-(4-nitrophenyl)acrylaldehyde

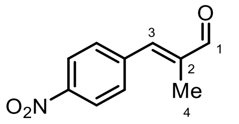

General procedure **A** was followed using 4-nitrobenzaldehyde (4.00 g, 26.5 mmol), and propionaldehyde (1.91 mL, 26.5 mmol) to afford the title compound (3.36 g, 66%) as a yellow solid without purification. m.p.: 113 – 114 °C (CH_2Cl_2/hexane) [Lit.: 117 °C][21]; ^{1}H NMR (400 MHz, $CDCl_3$) δ 9.65 (1H, s, C**1**-H), 8.32 – 8.29 (2H, m, ArCH), 7.68 – 7.65 (2H, m, ArCH), 7.32 (1H, br s, C**3**-H), 2.09 (3H, d, J = 1.5 Hz, C**4**-H_3); ^{13}C NMR (101 MHz, $CDCl_3$) δ 194.8 (C**1**), 147.9 (ArC), 146.2 (C**3**), 141.5 (C**2**), 141.4 (ArC), 130.5 (ArCH), 124.0 (ArCH), 11.2 (C**4**).

The spectroscopic properties were consistent with the data available in literature.[21]

(*E*)-2-Methyl-3-(4-nitrophenyl)prop-2-en-1-ol

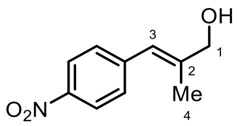

General procedure **C** was followed using the preceding aldehyde (3.36 g, 17.6 mmol) and $NaBH_4$ (700 mg, 18.5 mmol) to give the title compound (3.35 g, 99%) as a yellow oil without purification. ^{1}H NMR (400 MHz, $CDCl_3$) δ 8.21 - 8.17 (2H, m, ArCH), 7.43 – 7.40 (2H, m, ArCH), 6.61 (1H, br s, C**3**-H), 4.24 – 4.22 (2H, m, C**1**-H_2), 1.92 – 1.90 (3H, m, C**4**-H_3), 1.71 (1H, t, J = 5.5 Hz, OH); ^{13}C NMR (101 MHz, $CDCl_3$) δ 146.2 (ArC), 144.6 (ArC), 142.0 (C**2**), 129.6 (ArCH), 123.7 (ArCH), 122.7 (C**3**), 68.3 (C**1**), 15.6 (C**4**).

The spectroscopic properties were consistent with the data available in literature.[22]

Ethyl 4-methyl-3-(4-nitrophenyl)pent-4-enoate

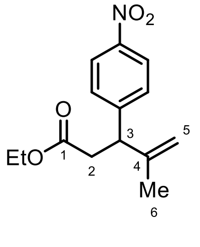

General procedure **D** was followed using the preceding alcohol (3.35 g, 17.3 mmol), triethyl orthoacetate (22.2 mL, 121 mmol) and pivalic acid (176 mg, 1.73 mmol) for 3 cycles with no subsequent hydrolysis step. FCC (3% EtOAc/hexane) gave the title compound (3.39 g, 75%) as a pale yellow oil. ν_{max} / cm^{-1}: 2981 (m), 1733 (s), 1521 (s), 1347 (s), 1156 (m), 1029 (m), 857 (m); ^{1}H NMR (400 MHz, $CDCl_3$) δ 8.17 – 8.13 (2H, m, ArC<u>H</u>), 7.41 - 7.38 (2H, m, ArC<u>H</u>), 4.96 – 4.93 (2H, m, C**5**-<u>H</u>$_2$), 4.10 – 4.02 (2H, m, OC<u>H</u>$_2$$CH_3$), 3.90 (1H, dd, *J* = 8.0, 8.0 Hz, C**3**-<u>H</u>) 2.88 (1H, dd, *J* = 15.5, 8.0 Hz, C**2**-<u>H</u>), 2.72 (1H, dd, *J* = 15.5, 8.0 Hz, C**2**-<u>H</u>'), 1.60 (3H, br s, C**6**-<u>H</u>$_3$), 1.16 (3H, t, *J* = 7.0 Hz, OCH_2C<u>H</u>$_3$); ^{13}C NMR (101 MHz, $CDCl_3$) δ 171.5 (C**1**), 149.9 (Ar<u>C</u>), 147.0 (Ar<u>C</u>), 145.4 (C**4**), 128.8 (Ar<u>C</u>H), 123.8 (Ar<u>C</u>H), 112.0 (C**5**), 60.8 (O<u>C</u>H_2CH_3), 48.5 (C**3**), 38.9 (C**2**), 21.7 (C**6**), 14.2 (OCH_2<u>C</u>H_3); *m/z* (ESI^+) HRMS: Calculated for $C_{14}H_{18}NO_4$: 264.1230. Found $[M+H]^+$ 264.1237.

4-Methyl-3-(4-nitrophenyl)pent-4-en-1-ol

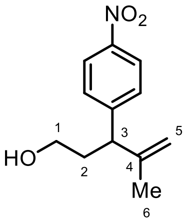

To a solution of the preceding ester (3.39 g, 12.9 mmol) in anhydrous Et_2O (64.5 mL) at 0 °C, was added DIBAL (1M in THF, 28.4 mL) dropwise *via* syringe. The reaction was stirred at 0 °C for 4 hours. Upon completion, the reaction was diluted with Et_2O (40 mL) before the addition of water (1.29 mL), 15 % aqueous solution of NaOH (1.29 mL) and a final portion of water (3.23 mL). The reaction mixture was stirred at room temperature for 15 minutes, dried (Na_2SO_4) and concentrated *in vacuo*. FCC (10% EtOAc/hexane) gave the title compound (2.85 g, 72%) as an orange oil. ^{1}H NMR (400 MHz, $CDCl_3$) δ 8.14 (2H, d, *J* = 8.5 Hz, ArC<u>H</u>), 7.39 (2H, d, *J* = 8.5 Hz, ArC<u>H</u>), 4.99 (1H, m, C**5**-<u>H</u>), 4.93 (1H, m, C**5**-<u>H</u>'), 3.66 – 3.51 (3H, m, C**3**-<u>H</u>, C**1**-<u>H</u>$_2$), 2.20 – 2.12 (1H, m, C**2**-<u>H</u>), 2.00 – 1.91 (1H, m, C**2**-<u>H</u>'), 1.57 (3H, s, C**6**-<u>H</u>$_3$); ^{13}C NMR (101 MHz, $CDCl_3$) δ 151.1 (Ar<u>C</u>), 146.8 (Ar<u>C</u>), 146.2 (C**4**), 128.8 (Ar<u>C</u>H), 123.7 (Ar<u>C</u>H), 112.2 (C**5**), 60.6 (C**1**), 48.7 (C**3**), 35.5 (C**2**), 21.1 (C**6**).

The spectroscopic properties were consistent with the data available in literature.[16]

Benzyl (4-methyl-3-(4-nitrophenyl)pent-4-en-1-yl)((perfluorobenzoyl)oxy)carbamate (1g)

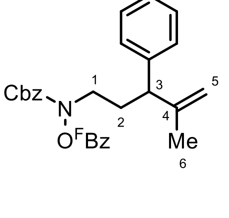

General procedure **I** was followed using the preceding alcohol (443 mg, 2.00 mmol), CbzNHO^{F}Bz (795 mg, 2.20 mmol), triphenylphosphine (577 mg, 2.20 mmol) and diisopropyl azodicarboxylate (436 µL, 2.20 mmol). FCC (5 – 10% EtOAc/hexane) gave title compound **1g** (730 mg, 65%) as a pale-yellow solid. m.p.: 68 - 69 °C (CH_2Cl_2/hexane); ν_{max} / cm^{-1}: 2971 (m), 1784 (m), 1523 (s), 1500 (s), 1347 (s), 1174 (s), 1004 (m), 698 (m); ^{1}H NMR (400 MHz, $CDCl_3$) δ 8.13 – 8.09 (2H, m, ArCH), 7.38 – 7.30 (7H, m, Cbz 5 × ArCH + 2 × ArCH), 5.20 (2H, s, Cbz CH_2), 4.97 – 4.94 (2H, m, C**5**-H_2), 3.74 – 3.66 (2H, m, C**1**-H_2), 3.50 (1H, dd, *J* = 7.5, 7.5 Hz, C**3**-H), 2.27 – 2.18 (1H, m, C**2**-H), 2.08 – 2.00 (1H, m, C**2**-H'), 1.56 (3H, br s, C**1**-H_3); ^{13}C NMR (101 MHz, $CDCl_3$) δ 155.4 (Cbz C=O), 150.2 (ArC), 147.0 (ArC), 145.4 (C**4**), 135.1 (Cbz ArC), 128.8 (Cbz ArCH), 128.8 (Cbz ArCH), 128.7 (Cbz ArCH), 128.4 (ArCH), 123.9 (ArCH), 112.5 (C**5**), 69.1 (Cbz CH_2), 49.7 (C**1**), 49.3 (C**3**), 30.1 (C**2**), 21.19 (C**6**); *mixture of rotamers A + B (9:1)* ^{19}F NMR (377 MHz, $CDCl_3$) δ -135.0 – - 135.2 (0.2F, m, B), -136.1 – -136.2 (1.8F, m, A), -145.0 (0.1F, tt, *J* = 21.0, 6.0 Hz, B) -145.5 – -145.7 (0.9F, tt, *J* = 21.0, 5.5 Hz, A), -158.9 – -159.1 (2F, m, A + B). *m/z* (ESI$^+$) HRMS: Calculated for $C_{27}H_{21}F_5N_2O_6Na$: 587.1217. Found $[M+Na]^+$ 587.1291.

The ^{13}C signals corresponding to the pentafluorobenzoyl group could not be resolved due to their weak intensity.

Some signal broadening was observed due to amide-like resonance.

Benzyl 8a-methyl-6-nitro-3,3a,8,8a-tetrahydroindeno[2,1-*b*]pyrrole-1(2*H*)-carboxylate (2g)

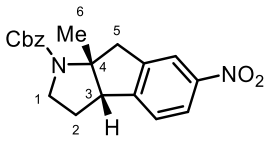

General procedure **J** was followed using cyclization substrate **1g** (56.4 mg, 0.10 mmol), $Pd_2(dba)_3$ (2.29 mg, 0.0025 mmol), CgPPh (4.38 mg, 0.015 mmol), sodium benzoate (1.44 mg, 0.01 mmol), triethylamine (1.40 µL) and dibutyl ether (1 mL). The reaction was stirred at 130°C for 48 hours. FCC (10 – 20% EtOAc/hexane) gave title compound **2g** (22.9 mg, 65%) as a pale yellow oil. R_f: 0.25 (20% EtOAc/hexane); ν_{max} / cm^{-1}: 2928 (m), 1695 (s), 1520 (s), 1404 (s), 1347 (s), 1102 (m), 1051 (m), 742 (m); *mixture of rotamers A + B (2:1)* ^{1}H NMR (500 MHz, $CDCl_3$) δ 8.10 – 8.07 (1H, m, ArCH, A + B), 8.02 (0.66H, br s, ArCH, A), 7.97 (0.33H, br s, ArCH, B), 7.43 – 7.28 (6H, m, 5 × Cbz ArCH + ArCH, A + B), 5.20 (0.66H, s, Cbz CH_2, B), 5.11 (0.66H, d, *J* = 12.5 Hz, Cbz CH, A), 5.04 (0.66H, d, *J* = 12.5 Hz, Cbz CH', A), 3.94 (0.66H, d, *J* = 17.5 Hz, C**5**-H, A), 3.72 – 3.64 (1H, m, C**1**-H, A + B), 3.65 (0.33H, m, C**5**-H, B), 3.56 – 3.52 (1H, m, C**3**-H, A + B), 3.24 – 3.18 (0.33H, m, C**1**-H', B), 3.10 – 3.03 (0.66H, m, C**1**-H', A), 3.10 – 3.03 (1H, m, C**5**-H', A + B), 2.36 – 2.27 (1H, m, C**2**-H, A + B), 2.13 – 2.04 (1H, m, C**2**-H', A + B), 1.67 (2H, s, C**6**-H_3, A), 1.59 (1H, s, C**6**-H_3, B); ^{13}C NMR (126 MHz, $CDCl_3$) δ 154.6 (C=O, B), 153.7 (C=O, A), 151.3 (ArC, A), 151.0 (ArC, B), 148.1 (ArC, A + B), 144.8 (ArC, A), 144.0

(ArC, B), 136.9 (Cbz ArC, A), 136.7 (Cbz ArC, B), 128.8, 128.6, 128.3, 128.3, 128.1, 127.9 (Cbz ArCH, A + B), 124.3 (ArCH, B), 124.2 (ArCH, A), 122.9 (ArCH, B), 122.7 (ArCH, A), 120.4 (ArCH, A), 120.3 (ArCH, B), 72.0 (C**4**, A), 71.3 (C**4**, B), 67.3 (Cbz CH_2, B), 66.6 (Cbz CH_2, A), 58.4 (C**3**, B), 56.9 (C**3**, A), 48.1 (C**1**, B), 47.0 (C**1**, A), 44.6 (C**5**, B), 43.1 (C**5**, A), 28.1 (C**2**, A), 27.9 (C**2**, B), 25.0 (C**6**, B), 23.5 (C**6**, A); *m/z* (ESI^+) HRMS: Calculated for $C_{20}H_{21}N_2O_4$: 353.1496. Found $[M+H]^+$ 353.1494.

Some signal broadening was observed due to amide-like resonance.

(*E*)-2,3-Diphenylacrylaldehyde

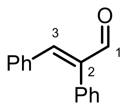

General procedure **A** was followed using benzaldehyde (1.92 mL, 18.8 mmol), and phenylacetaldehyde (2.20 mL, 18.8 mmol). FCC (1 – 5% EtOAc/hexane) gave the title compound (1.70 g, 29%) as a colorless solid. m.p.: 93 – 94 °C (CH_2Cl_2/hexane) [Lit.: 92 – 94 °C][23]; ^{1}H NMR (400 MHz, $CDCl_3$) δ 9.78 (1H, s, C**1**-H), 7.44 - 7.38 (4H, m, C**3**-H + ArCH), 7.32 – 7.28 (1H, m, ArCH), 7.24 - 7.19 (6H, m, ArCH); ^{13}C NMR (101 MHz, $CDCl_3$) δ 194.1 (C**1**), 150.3 (C**3**), 142.0 (C**2**), 134.2 (ArC), 133.5 (ArC), 130.9 (ArCH), 130.4 (ArCH), 129.5 (ArCH), 129.0 (ArCH), 128.7 (ArCH), 128.5 (ArCH).

The spectroscopic properties were consistent with the data available in literature.[24]

(*E*)-2,3-Diphenylprop-2-en-1-ol

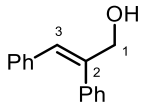

General procedure **C** was followed using the preceding aldehyde (1.70 g, 8.17 mmol) and $NaBH_4$ (320 mg, 8.58 mmol) to give the title compound (1.62 g, 94%) as a colorless solid without purification. m.p.: 71 – 72 °C (CH_2Cl_2/hexane) [Lit.: 70 – 72 °C][25]; ^{1}H NMR (400 MHz, $CDCl_3$) δ 7.36 – 7.30 (3H, m, ArCH), 7.24 – 7.22 (2H, m, ArCH), 7.13 – 7.09 (3H, m, ArCH), 7.01 – 6.99 (2H, m, ArCH), 6.70 (1H, s, C**3**-H), 4.47 (2H, dd, J = 6.0, 1.5 Hz, C**1**-H_2); ^{13}C NMR (101 MHz, $CDCl_3$) δ 141.6 (C**2**) 138.6 (ArC), 136.6 (ArC), 129.4 (ArCH), 129.0 (ArCH), 128.9 (ArCH), 128.1 (ArCH), 127.7 (ArCH), 127.0 (ArCH), 126.7 (C**3**), 68.7 (C**1**).

The spectroscopic properties were consistent with the data available in literature.[26]

3,4-Diphenylpent-4-enoic acid

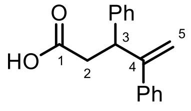

General procedure **E** was followed using the preceding alcohol (1.60 g, 7.60 mmol), triethyl orthoacetate (9.75 mL, 53.2 mmol) and pivalic acid (77.6 mg, 0.760 mmol) for 3 cycles. The subsequent hydrolysis was stirred at 60 °C for 16 hours. The title compound (1.82 g, 95%) was obtained as a yellow oil. ν_{max} / cm^{-1}: 3060 (m), 3029 (m), 1707 (s); ^{1}H NMR (400 MHz, $CDCl_3$) δ 7.32 – 7.19 (10H, m, ArCH), 5.42 (1H, m, C**5**-H), 5.18 (1H, m, C**5**-H'), 4.44 (1H, dd, *J* = 8.0, 8.0 Hz, C**3**-H), 2.99 (1H, dd, *J* = 16.0, 8.0 Hz, C**2**-H), 2.84 (1H, dd, *J* = 16.0, 8.0 Hz, C**2**-H'); ^{13}C NMR (101 MHz, $CDCl_3$) δ 177.6 (C**1**), 150.5 (C**4**), 141.7 (ArC), 141.5 (ArC), 128.7 (ArCH), 128.3 (ArCH), 128.0 (ArCH), 127.6 (ArCH), 127.0 (ArCH), 113.6 (C**5**), 46.2 (C**3**), 40.2 (C**2**); *m/z* (ES^-) HRMS: Calculated for anion $C_{17}H_{15}O_2$: 251.1072. Found $[M-H]^-$ 251.1064.

3,4-Diphenylpent-4-en-1-ol

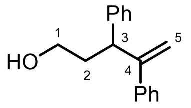

General procedure **F** was followed using the preceding carboxylic acid (1.68 g, 6.66 mmol), $LiAlH_4$ (1M in THF, 6.66 mL) and anhydrous Et_2O at 0 °C for 3 hours. FCC (20% EtOAc/hexane) gave the title compound (789 mg, 50%) as colorless oil. ^{1}H NMR (400 MHz, $CDCl_3$) δ 7.38 – 7.16 (10H, m, ArCH), 5.41 (1H, m, C**5**-H), 5.21 (1H, m, C**5**-H'), 4.03 (1H, m, C**3**-H), 3.68 – 3.55 (2H, m, C**1**-H_2), 2.25 – 2.16 (1H, m, C**2**-H), 2.09 – 2.00 (1H, m, C**2**-H'); ^{13}C NMR (101 MHz, $CDCl_3$), 151.6 (C**4**), 142.8 (ArC), 142.4 (ArC), 128.6 (ArCH), 128.3 (ArCH), 128.2 (ArCH), 127.4 (ArCH), 126.9 (ArCH), 126.5 (ArCH), 113.5 (C**5**), 61.2 (C**1**), 46.6 (C**3**), 37.8 (C**2**).

The spectroscopic properties were consistent with the data available in literature.[16]

Benzyl (3,4-diphenylpent-4-en-1-yl)((perfluorobenzoyl)oxy)carbamate (1h)

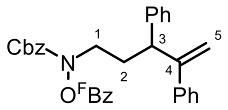

General procedure **I** was followed using the preceding alcohol (318 mg, 1.33 mmol), $CbzNHO^{F}Bz$ (531 mg, 1.47 mmol), triphenylphosphine (396 mg, 1.47 mmol) and diisopropyl azodicarboxylate (264 µL, 1.33 mmol). FCC (5% EtOAc/hexane) gave title compound **1h** (650 mg, 84%) as a colorless solid. m.p.: 72 – 74 °C (CH_2Cl_2/Hexane); ν_{max} / cm^{-1}: 2958 (m), 1785 (m), 1733 (m), 1499 (s), 1327 (s), 1174 (s); ^{1}H NMR (400 MHz, $CDCl_3$) δ 7.38 – 7.15 (15H, m, ArCH), 5.41 (1H, m, C**5**-H), 5.19 – 5.17 (3H, m, Cbz CH_2 + C**5**-H'), 3.96 (1H, dd, *J* = 7.5, 7.5 Hz, C**3**-H), 3.84 – 3.68 (2H, m, C**1**-H_2), 2.32 – 2.23 (1H, m, C**2**-H), 2.17 – 2.08 (1H, m, C**2**-H'); ^{13}C NMR (101 MHz, $CDCl_3$) δ 155.5 (C=O), 150.7 (C**4**), 142.1 (ArC), 142.1 (ArC), 135.3 (Cbz ArC), 128.7 (ArCH), 128.7 (ArCH), 128.6 (ArCH), 128.3 (ArCH), 128.2 (ArCH), 128.1 (ArCH), 127.4 (ArCH), 126.9 (ArCH), 126.8 (ArCH), 113.6 (C**5**), 68.9 (Cbz CH_2), 50.0 (C**1**), 47.3 (C**3**), 32.2 (C**2**); ^{19}F NMR (377 MHz, $CDCl_3$) δ -135.8 – -135.9 (2F, m), -146.1 (1F, tt,

J = 21.0, 5.5 Hz), -159.3 – -159.4 (2F, m); *m/z* (ESI$^+$) HRMS: Calculated for $C_{32}H_{28}F_5N_2O_4$: 599.1964. Found [M+NH$_4$]$^+$ 599.1958.

The ^{13}C signals corresponding to the pentafluorobenzoyl group could not be resolved due to their weak intensity.

Benzyl 8a-phenyl-3,3a,8,8a-tetrahydroindeno[2,1-*b*]pyrrole-1(2*H*)-carboxylate (2h)

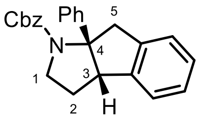

General procedure **J** was followed using cyclization substrate **1h** (27.2 mg, 0.047 mmol), Pd_2dba_3 (1.08 mg, 0.0012 mmol), CgPPh (2.07 mg, 0.0071 mmol), Et_3N (0.66 µL, 0.0047 mmol) and NaOBz (0.68 mg, 0.0047 mmol). The reaction was stirred at 130 °C for 48 hours. FCC (5 – 10% EtOAc/hexane) gave title compound **2h** (10.7 mg, 62%) as a colorless oil. R_f: 0.26 (10% EtOAc/hexane); ν_{max} / cm^{-1}: 2959 (m) 1699 (s), 1403 (s), 1104 (m), 698 (m); *Mixture of rotamers A + B (1:1)* ^{1}H NMR (500 MHz, $CDCl_3$) δ 7.36 – 7.13 (13H, m, Cbz + Ph ArCH, A + B), 6.90 – 6.87 (1H, m, ArCH, B), 5.19 (0.5H, d, *J* = 12.5 Hz, Cbz CH, A), 5.08 (0.5H, d, *J* = 12.5 Hz, Cbz CH, B), 5.04 (0.5H, d, *J* = 12.5 Hz, Cbz CH', A), 4.99 (0.5H, d, *J* = 12.5 Hz, Cbz CH', B), 4.16 (0.5H, d, *J* = 17.0 Hz, C**5**-H, A) 3.90 (0.5H, ddd, *J* = 11.0, 8.0, 4.0 Hz, C**1**-H, B), 3.87 – 3.81 (0.5H, m, C**1**-H, A), 3.81 – 3.74 (1.5H, m, C**5**-H', A, C**5**-H_2, B), 3.75 – 3.69 (1H, C**3**-H, A + B), 3.55 – 3.49 (0.5H, m, C**1**-H', B), 3.33 (0.5H, ddd, *J* = 10.5, 10.5, 7.0 Hz, C**1**-H', A), 2.30 – 2.15 (1H, m, C**2**-H, A + B), 2.12 – 2.03 (1H, m, C**2**-H', A + B); ^{13}C NMR (126 MHz, $CDCl_3$) δ 154.9 (C=O, B), 154.1 (C=O, A), 145.3, 143.8, 142.6, 142.5, 142.4, 142.0 (Ph ArC, A + B), 137.2 (Cbz ArC, A), 136.6 (Cbz ArC, B), 128.6, 128.5, 128.5, 128.3, 128.0, 127.9, 127.7, 127.7, 127.6, 127.5, 127.3, 127.1, 126.8, 126.6, 125.5, 125.2, 125.1, 124.7, 123.8, 123.5 (Cbz + Ph ArCH, A + B), 77.5 (C**4**, A), 76.6 (C**4**, B), 66.9 (Cbz CH_2, B), 66.7 (Cbz CH_2, A), 62.2 (C**3**, B), 60.9 (C**3**, A), 49.0 (C**1**, B), 47.7 (C**1**, A), 45.3 (C**5**, B), 44.0 (C**5**, A), 28.3 (C**2**, B), 27.8 (C**2**, A); *m/z* (ESI$^+$) HRMS: Calculated for $C_{25}H_{24}NO_2$: 370.1802. Found [M+H]$^+$ 370.1797.

Some signal broadening was observed due to amide-like resonance.

(*E*)-2-Benzylidene-3-methylbutan-1-ol

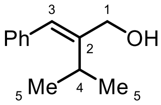

To a stirred solution of 3-phenyl-2-propyn-1-ol (1.06 g, 8.00 mmol) and copper(I) iodide (152 mg, 0.80 mmol) in anhydrous Et_2O (40 mL) at 0 °C, was added isopropylmagnesium bromide (1M in THF, 28.0 mL) dropwise *via* syringe. The reaction was allowed to warm to room temperature and stirred for 24 hours. Upon completion, the reaction was quenched with sat. NH_4Cl solution (50 mL), diluted with water (100 mL) and extracted with Et_2O (3 × 100 mL). The organic phase was washed with brine (100

mL), dried (Na_2SO_4), and concentrated *in vacuo*. FCC (20% EtOAc/hexane) gave the title compound (1.42 g, 100%) as a yellow oil. ^{1}H NMR (400 MHz, $CDCl_3$) δ 7.36 – 7.31 (2H, m, ArCH), 7.25 – 7.20 (3H, m, ArCH), 6.55 (1H, s, C**3**-H), 4.31 (2H, dd, *J* = 6.0, 1.5 Hz, C**1**-H_2), 3.10 (1H, m, C**4**-H), 1.38 (1H, t, *J* = 6.0 Hz, OH), 1.11 (6H, d, *J* = 7.0 Hz, C**5**-H_3); ^{13}C NMR (101 MHz, $CDCl_3$) δ 147.2 (C**2**), 137.9 (ArC), 128.8 (ArCH), 128.3 (ArCH), 126.5 (ArCH), 124.6 (C**3**), 63.2 (C**1**), 28.2 (C**4**), 21.7 (C**5**).

The spectroscopic properties were consistent with the data available in the literature.[27]

5-Methyl-4-methylene-3-phenylhexanoic acid

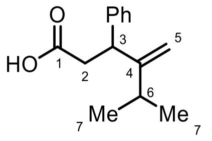

General procedure **E** was followed using the preceding alcohol (1.42 g, 8.00 mmol), triethyl orthoacetate (14.8 mL, 80.0 mmol) and propionic acid (0.06 mL, 0.80 mmol) for 2 cycles. The subsequent hydrolysis was stirred at 60 °C for 16 hours. The title compound (1.21 g, 69%) was obtained as a yellow oil. ν_{max} / cm^{-1}: 2961 (m), 2669 (br m), 1706 (s), 896 (m), 700 (s); ^{1}H NMR (400 MHz, $CDCl_3$) δ 7.30 – 7.26 (2H, m, ArCH), 7.25 – 7.18 (3H, m, ArCH), 5.01 (1H, m, C**5**-H), 4.97 (1H, br d, *J* = 1.0 Hz, C**5**-H'), 3.88 (1H, dd, *J* = 8.0, 8.0 Hz, C**3**-H), 2.88 (1H, dd, *J* = 16.0, 8.0 Hz, C**2**-H), 2.71 (1H, dd, *J* = 16.0, 8.0 Hz, C**2**-H'), 2.10 – 2.00 (1H, m, C**6**-H), 0.98 (3H, d, *J* = 7.0 Hz, C**7**-H_3), 0.94 (3H, d, *J* = 7.0 Hz, C**7'**-H_3); ^{13}C NMR (101 MHz, $CDCl_3$) δ 177.6 (C**1**), 157.1 (C**4**), 142.5 (ArC), 128.6 (ArCH), 128.1 (ArCH), 126.9 (ArCH), 107.1 (C**5**), 46.4 (C**3**), 40.3 (C**2**), 33.2 (C**6**), 22.9 (C**7**), 22.1 (C**7**'); *m/z* (ES$^-$) HRMS: Calculated for anion $C_{14}H_{17}O_2$: 217.1229. Found [M-H]$^-$: 217.1233.

5-Methyl-4-methylene-3-phenylhexan-1-ol

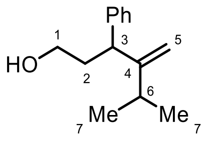

General procedure **F** was followed using the preceding carboxylic acid (1.20 g, 5.50 mmol), $LiAlH_4$ (1M in THF, 11 mL) and anhydrous THF at 0 °C for 3 hours. The title compound (709 mg, 63%) was obtained as colorless oil without purification. ^{1}H NMR (400 MHz, $CDCl_3$) δ 7.30 – 7.26 (2H, m, ArCH), 7.24 – 7.17 (3H, m, ArCH), 5.00 – 4.98 (2H, m, C**5**-H_2), 3.63 – 3.51 (2H, m, C**1**-H_2), 3.48 (1H, m, C**3**-H), 2.16 – 2.01 (2H, m, C**2**-H + C**6**-H), 1.99 – 1.89 (1H, m, C**2**-H'), 0.95 (3H, d, *J* = 7.0 Hz, C**7**-H_3), 0.94 (3H, d, *J* = 7.0 Hz, C**7'**-H_3); ^{13}C NMR (101 MHz, $CDCl_3$) δ 158.5 (C**4**), 143.8 (ArC), 128.5 (ArCH), 128.2 (ArCH), 126.5 (ArCH), 106.9 (C**5**), 61.5 (C**1**), 47.0 (C**3**), 37.8 (C**2**), 33.2 (C**6**), 23.2 (C**7**), 22.3 (C**7**'). *m/z* (APCI$^+$) HRMS: Calculated for $C_{14}H_{21}O$: 205.1587. Found [M+H]$^+$: 205.1579.

Benzyl (5-methyl-4-methylene-3-phenylhexyl)((perfluorobenzoyl)oxy)carbamate (1i)

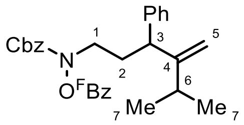

General procedure **I** was followed using the preceding alcohol (532 mg, 2.60 mmol), CbzNHO^{F}Bz (1.19 g, 3.30 mmol), triphenylphosphine (866 mg, 3.30 mmol) and diisopropyl azodicarboxylate (0.650 mL, 3.30 mmol). FCC (0 – 2 % EtOAc/hexane) gave title compound **1i** (1.12 g, 79%) as a colorless oil. ν_{max} / cm^{-1}: 2962 (m), 1786 (s), 1736 (s), 1500 (s), 1327 (s), 1175 (s), 1004 (s); ^{1}H NMR (400 MHz, $CDCl_3$) δ 7.38 – 7.29 (5H, m, Cbz ArCH), 7.26 – 7.22 (2H, m, Ph ArCH), 7.20 – 7.15 (3H, m, Ph ArCH), 5.20 (2H, s, Cbz CH_2), 4.99 (1H, m, C**5**-H), 4.95 (1H, m, C**5**-H’), 3.74 (1H, ddd, *J* = 15.0, 8.5, 6.5 Hz, C**1**-H), 3.63 (1H, ddd, *J* = 14.5, 8.5, 5.5 Hz, C**1**-H’), 3.41 (1H, dd, *J* = 7.5, 7.5 Hz, C**3**-H), 2.22 – 2.13 (1H, m, C**2**-H), 2.07 – 1.97 (2H, m, C**2**-H’ + C**6**-H), 0.93 (3H, d, *J* = 7.0 Hz, C**7**-H_3), 0.92 (3H, d, *J* = 7.0 Hz, C**7’**-H_3); ^{13}C NMR (101 MHz, $CDCl_3$) δ 157.5 (C**4**), 155.6 (C=O), 143.1 (Ph ArC), 135.3 (Cbz ArC), 128.7 (Cbz ArCH), 128.6 (Cbz ArCH), 128.5 (Ph ArCH), 128.3 (Cbz ArCH), 128.1 (Ph ArCH), 126.6 (Ph ArCH), 107.1 (C**5**), 68.9 (Cbz CH_2), 50.2 (C**1**), 47.5 (C**3**), 33.2 (C**6**), 32.0 (C**2**), 23.0 (C**7**), 22.2 (C**7**’); ^{19}F NMR (377 MHz, $CDCl_3$) δ -135.9 - -136.1 (2F, m), -146.1 (1F, tt, *J* = 21.0, 5.5 Hz), -159.2 - -159.4 (2F, m); *m/z* (ESI$^+$) HRMS: Calculated for $C_{29}H_{26}F_5NO_4Na$: 570.1674. Found $[M+Na]^+$ 570.1673.

The ^{13}C signals corresponding to the pentafluorobenzoyl group could not be resolved due to their weak intensity.

Benzyl 8a-isopropyl-3,3a,8,8a-tetrahydroindeno[2,1-*b*]pyrrole-1(2*H*)-carboxylate (2i)

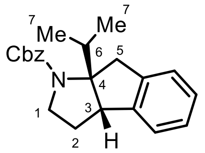

General procedure **J** was followed using cyclization substrate **1i** (54.8 mg, 0.1 mmol), Pd_2dba_3 (2.29 mg, 0.0025 mmol), CgPPh (4.38 mg, 0.015 mmol), Et_3N (1.40 µL, 0.01 mmol) and NaOBz (1.44 mg, 0.01 mmol). The reaction was stirred at 130 °C for 48 hours. FCC (1% acetone/hexane) gave title compound **2i** (21.9 mg, 65%) as a colorless oil. R_f: 0.33 (1% acetone/hexane); ν_{max} / cm^{-1}: 2961 (m), 2873 (m), 1697 (s), 1404 (s), 1356 (s), 1338 (s); *Mixture of rotamers A + B (2:1)* ^{1}H NMR (500 MHz, $CDCl_3$) δ 7.43 – 7.27 (5H, m, Cbz ArCH, A + B), 7.19 – 7.07 (4H, m, ArCH, A + B), 5.23 (0.33H, d, *J* = 12.5 Hz, Cbz CH, B), 5.19 (0.33H, d, *J* = 12.5 Hz, Cbz CH’, B), 5.15 (0.66H, d, *J* = 12.5 Hz, Cbz CH, A), 5.07 (0.66H, d, *J* = 12.5 Hz, Cbz CH’, A), 3.76 – 3.71 (1H, m, C**3**-H, A + B), 3.61 – 3.51 (2H, m, C**5**-H, A, C**1**-H, A, C**1**-H_2, B), 3.42 – 3.34 (1H, m, C**5**-H, B, C**1**-H’, A), 3.31 (0.66H, d, *J* = 18.0 Hz, C**5**-H’, A), 3.21 (0.33H, d, *J* = 18.0 Hz, C**5**-H’, B), 2.92 (0.66H, m, C**6**-H, A), 2.69 (0.33H, m, C**6**-H,

B), 2.28 – 2.19 (1H, m, C**2**-H, A + B), 1.95 – 1.89 (0.66H, m, C**2**-H', A), 1.88 – 1.81 (0.33H, m, C**2**-H', B), 0.91 (2H, d, J = 7.0 Hz, C**7**-H_3, A), 0.88 (1H, d, J = 7.0 Hz, C**7**-H_3, B), 0.85 (2H, d, J = 7.0 Hz, C**7'**-H_3, A), 0.74 (1H, d, J = 7.0 Hz, C**7'**-H_3, B); ^{13}C NMR (126 MHz, $CDCl_3$) δ 154.9 (C=O, B), 153.9 (C=O, A), 144.7 (ArC, B), 144.6 (ArC, B), 142.5 (ArC, A), 141.8 (ArC, B), 137.4 (Cbz ArC, A), 137.1 (Cbz ArC, B), 128.6, 128.6, 128.1, 128.0, 127.9, 127.7 (Cbz ArCH, A + B), 127.3 (ArCH, A + B), 126.9 (ArCH, B), 126.8 (ArCH, A), 124.8 (ArCH, A), 124.5 (ArCH, B), 123.7 (ArCH, A), 123.5 (ArCH, B), 78.4 (C**4**, A), 77.3 (C**4**, B), 67.1 (Cbz CH_2, B), 66.3 (Cbz CH_2, A), 50.9 (C**3**, B), 49.7 (C**3**, A), 49.4 (C**1**, B), 48.1 (C**1**, A), 44.5 (C**5**, B), 42.5 (C**5**, A), 33.7 (C**6**, B), 31.8 (C**6**, A), 31.3 (C**2**, A), 31.1 (C**2**, B), 18.9 (C**7**, A), 18.9 (C**7**, B), 18.4 (C**7**', A), 17.9 (C**7**', B); *m/z* (ESI^+) HRMS: Calculated for $C_{22}H_{26}NO_2$: 336.1958. Found $[M+H]^+$ 336.1960.

Some signal broadening was observed due to amide-like resonance.

(*E*)-3-(3-Chlorophenyl)-2-methylacrylaldehyde

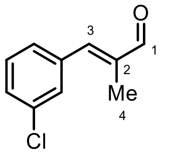

General procedure **A** was followed using 3-chlorobenzaldehyde (3.50 mL, 30.9 mmol) and propionaldehyde (3.12 mL, 43.3 mmol). FCC (1 – 4% EtOAc/hexane) gave the title compound (1.87 g, 33%) as a colorless oil. ^1H NMR (400 MHz, $CDCl_3$) δ 9.59 (1H, s, C**1**-H), 7.50 (1H, m, ArCH), 7.41 – 7.37 (3H, m, ArCH), 7.21 (1H, m, C**3**-H), 2.07 (3H, d, J = 1.5 Hz, C**4**-H_3); ^{13}C NMR (101 MHz, $CDCl_3$) δ 195.3 (C**1**), 147.9 (C**3**), 139.6 (C**2**), 137.0 (ArC), 134.9 (ArC), 130.1 (ArCH), 129.8 (ArCH), 129.6 (ArCH), 128.1 (ArCH), 11.1 (C**4**).

The spectroscopic properties were consistent with the data available in literature.[28]

(*E*)-3-(3-Chlorophenyl)-2-methylprop-2-en-1-ol

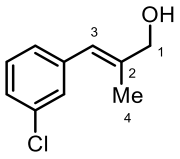

General procedure **C** was followed using the preceding aldehyde 1.79 g, 9.91 mmol) and $NaBH_4$ (394 mg, 10.4 mmol) to give the title compound (1.65 g, 91%) as a colorless oil without purification. ν_{max} / cm^{-1}: 3327 (m), 2913 (m), 1594 (s) 1475 (s), 1078 (s), 890 (s); ^1H NMR (400 MHz, $CDCl_3$) δ 7.28 – 7.23 (2H, m, ArCH), 7.21 – 7.18 (1H, m, ArCH), 7.16 – 7.13 (1H, m, ArCH), 6.47 (1H, s, C**3**-H), 4.19 – 4.17 (2H, m, C**1**-H_2), 1.88 (3H, d, J = 1.5 Hz, C**4**-H_3); ^{13}C NMR (101 MHz, $CDCl_3$) δ 139.5 (ArC), 139.2 (C**2**), 134.1 (ArC), 129.5 (ArCH), 128.9 (ArCH), 127.2 (ArCH), 126.6 (ArCH), 123.6 (C**3**), 68.7 (C**1**), 15.4 (C**4**); *m/z* (ESI^+) HRMS: Calculated for $C_{10}H_{10}Cl$: 165.0446. Found $[M+H-H_2O]^+$ 165.0467.

3-(3-Chlorophenyl)-4-methylpent-4-enoic acid

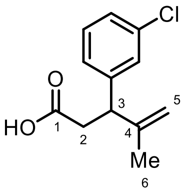

General procedure **E** was followed using the preceding alcohol (1.60 g, 8.76 mmol), triethyl orthoacetate (11.2 mL, 61.3 mmol) and pivalic acid (89.4 mg, 0.876 mmol) for 3 cycles. The subsequent hydrolysis was stirred at 60 °C for 16 hours. The title compound (1.59 g, 81%) was obtained as a yellow oil. ν_{max} / cm^{-1}: 2973 (m), 1709 (s), 1429 (m), 897 (m), 696 (m); ^{1}H NMR (400 MHz, $CDCl_3$) δ ^{1}H NMR (400 MHz, $CDCl_3$) δ 7.24 – 7.18 (3H, m, ArC<u>H</u>), 7.13 – 7.09 (1H, m, ArC<u>H</u>), 4.94 – 4.92 (2H, m, C**5**-<u>H</u>$_2$), 3.74 (1H, dd, *J* = 8.0, 8.0 Hz, C**3**-<u>H</u>), 2.88 (1H, dd, *J* = 16.0, 8.0 Hz, C**2**-<u>H</u>), 2.72 (1H, dd, *J* = 16.0, 8.0 Hz, C**2**-<u>H</u>'), 1.61 (3H, s, C**6**-<u>H</u>$_3$); ^{13}C NMR (101 MHz, $CDCl_3$) δ 177.8 (C**1**), 145.8 (C**4**), 144.0 (Ar<u>C</u>), 134.5 (Ar<u>C</u>), 129.9 (Ar<u>C</u>H), 127.9 (Ar<u>C</u>H), 127.2 (Ar<u>C</u>H), 126.1 (Ar<u>C</u>H), 111.4 (C**5**), 47.9 (C**3**), 38.9 (C**2**), 21.8 (C**6**); *m/z* ($APCI^+$) HRMS: Calculated for $C_{12}H_{14}ClO_2$: 225.0677. Found $[M+H]^+$ 225.0678.

3-(3-Chlorophenyl)-4-methylpent-4-en-1-ol

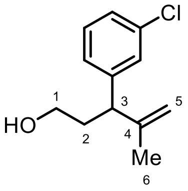

General procedure **F** was followed using the preceding carboxylic acid (1.59 g, 7.08 mmol), $LiAlH_4$ (1M in THF, 7.08 mL) and anhydrous Et_2O at 0 °C for 3 hours. FCC (15% EtOAc/hexane) gave the title compound (757 mg, 51%) as colorless oil. ν_{max} / cm^{-1}: 3322 (m), 2941 (m), 1595 (m), 1428 (m), 1045 (s), 894 (s), 782 (s); ^{1}H NMR (400 MHz, $CDCl_3$) δ 7.24 – 7.17 (3H, m, ArC<u>H</u>), 7.11 (1H, dt, *J* = 7.0, 1.5 Hz, ArC<u>H</u>), 4.95 (1H, m, C**5**-<u>H</u>) 4.89 (1H, m, C**5**-<u>H</u>'), 3.66 – 3.52 (2H, m, C**1**-<u>H</u>$_2$), 3.41 (1H, dd, *J* = 7.5, 7.5 Hz, C**3**-<u>H</u>), 2.17 – 2.07 (1H, m, C**2**-<u>H</u>), 1.98 – 1.89 (1H, m, C**2**-<u>H</u>'), 1.58 (3H, br s, C**6**-<u>H</u>$_3$), 1.31 (1H, t, *J* = 5.5 Hz, O<u>H</u>); ^{13}C NMR (101 MHz, $CDCl_3$) δ 147.1 (C**4**), 145.3 (Ar<u>C</u>), 134.4 (Ar<u>C</u>), 129.7 (Ar<u>C</u>H), 128.0 (Ar<u>C</u>H), 126.8 (Ar<u>C</u>H), 126.3 (Ar<u>C</u>H), 111.3 (C**5**), 61.1 (C**1**), 48.7 (C**3**), 35.7 (C**2**), 21.1 (C**6**); *m/z* (ESI^+) HRMS: Calculated for $C_{12}H_{14}Cl$: 193.0779. Found $[M+H-H_2O]^+$ 193.0780.

Benzyl (3-(3-chlorophenyl)-4-methylpent-4-en-1-yl)((perfluorobenzoyl)oxy)carbamate (1j)

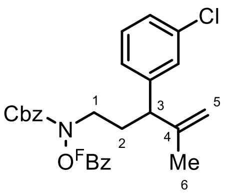

General procedure **I** was followed using the preceding alcohol (450 mg, 2.14 mmol), CbzNHO^{F}Bz (849 mg, 2.35 mmol), triphenylphosphine (616 mg, 2.35 mmol) and diisopropyl azodicarboxylate (424 µL, 2.14 mmol). FCC (1 – 7.5% EtOAc/hexane) gave title compound **1j** (1.08 mg, 91%) as a colorless oil. ν_{max} / cm^{-1}: 2970 (m), 1733 (m), 1500 (s), 1175 (s), 1004 (s), 697 (m); ^{1}H NMR (400 MHz, $CDCl_3$) δ 7.39 – 7.30 (5H, m, Cbz ArC<u>H</u>), 7.22 – 7.16 (3H, m, ArC<u>H</u>), 7.09 – 7.05 (1H, m, ArC<u>H</u>), 5.21 (2H, s, Cbz C<u>H$_2$</u>), 4.91 (1H, m, C**5**-<u>H</u>), 4.89 (1H, m, C**5**-<u>H</u>'), 3.76 - 3.62 (2H, m, C**1**-<u>H$_2$</u>), 3.32 (1H, dd, *J* = 7.5, 7.5 Hz, C**3**-<u>H</u>), 2.23 - 2.14 (1H, m, C**2**-<u>H</u>), 2.07 - 1.98 (1H, m, C**2**-<u>H</u>'), 1.56 (3H, br s, C**6**-<u>H$_3$</u>); ^{13}C NMR (101 MHz, $CDCl_3$) δ 155.5 (<u>C</u>=O), 146.1 (C**4**), 144.6 (Ar<u>C</u>), 135.2 (Cbz Ar<u>C</u>), 134.5 (Ar<u>C</u>), 129.8 (Ar<u>C</u>H), 128.7 (Cbz Ar<u>C</u>H), 128.7 (Cbz Ar<u>C</u>H), 128.3 (Cbz Ar<u>C</u>H), 127.9 (Ar<u>C</u>H), 127.0 (Ar<u>C</u>H), 126.2 (Ar<u>C</u>H), 111.6 (C**5**), 69.0 (Cbz <u>C</u>H$_2$), 49.9 (C**1**), 49.3 (C**3**), 30.1 (C**2**), 21.2 (C**6**); ^{19}F NMR (377 MHz, $CDCl_3$) δ -135.9 – -136.1 (2F, m), -145.9 (1F, tt, *J* = 21.0, 5.5 Hz), -159.1 – -159.3 (2F, m); *m/z* (ESI$^+$) HRMS: Calculated for $C_{27}H_{25}ClF_5N_2O_4$: 571.1418. Found $[M+NH_4]^+$ 571.1422.

The ^{13}C signals corresponding to the pentafluorobenzoyl group could not be resolved due to their weak intensity.

Benzyl 5-chloro-8a-methyl-3,3a,8,8a-tetrahydroindeno[2,1-*b*]pyrrole-1(2*H*)-carboxylate (2j) and benzyl 7-chloro-8a-methyl-3,3a,8,8a-tetrahydroindeno[2,1-*b*]pyrrole-1(2*H*)-carboxylate (2j')

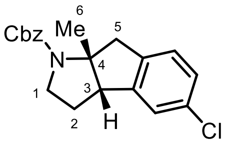

2j

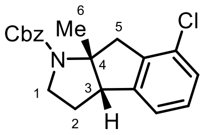

2j'

(<u>Ratio of **2j**:**2j'** = 1.3:1</u>) General procedure **J** was followed using cyclization substrate **1j** (55.4 mg, 0.1 mmol), Pd_2dba_3 (2.29 mg, 0.0025 mmol), CgPPh (4.38 mg, 0.015 mmol), sodium 2-methoxybenzoate (1.74 mg, 0.01 mmol), triethylamine (1.40 µL) and dibutylether (1 mL). The reaction was stirred at 130 °C for 72 hours. FCC (10% EtOAc/hexane) gave title compound **2j** (13.9 mg, 41%) and title compound **2j'** (10.7 mg, 31%) as colorless oils.

(<u>Ratio of **2j**:**2j'** = 1:1.7</u>) General procedure **J** was followed using cyclization substrate **1j** (55.4 mg, 0.1 mmol), Pd_2dba_3 (4.58 mg, 0.005 mmol), CgPPh (8.76 mg, 0.03 mmol), sodium 2-nitrobenzoate (1.89 mg, 0.01 mmol), triethylamine (1.40 µL) and dibutylether (1 mL). The reaction was stirred at 130 °C

for 72 hours. FCC (10% EtOAc/hexane) gave title compound **2j** (8.0 mg, 23%) and title compound **2j’** (13.7 mg, 40%) as colorless oils.

Benzyl 5-chloro-8a-methyl-3,3a,8,8a-tetrahydroindeno[2,1-*b*]pyrrole-1(2*H*)-carboxylate (2j): R_f: 0.43 (15% EtOAc/hexane); ν_{max} / cm^{-1}: 2963 (m), 1697 (s), 1404 (s), 1354 (s), 1100 (m), 4050 (m); *Mixture of rotamers A + B (2:1)* ^{1}H NMR (500 MHz, $CDCl_3$) δ 7.43 – 7.28 (5H, m, Cbz ArCH, A + B), 7.17 – 7.12 (2H, m, ArCH, A + B), 7.10 – 7.00 (1H, m, ArCH, A + B), 5.21 (0.33H, d, *J* = 12.5 Hz, Cbz CH, B), 5.17 (0.33H, d, *J* = 12.5 Hz, Cbz CH’, B), 5.11 (0.66H, d, *J* = 12.5 Hz, Cbz CH, A), 5.03 (0.66H, d, *J* = 12.5 Hz, Cbz CH’, A), 3.78 (0.66H, d, *J* = 17.0 Hz, C**5**-H, A), 3.69 – 3.61 (1H, m, C**1**-H, A + B), 3.51 (0.33H, d, *J* = 17.0 Hz, C**5**-H, B), 3.49 – 3.43 (1H, m, C**3**-H, A + B), 3.26 – 3.20 (0.33H, m, C**1**-H’, B), 3.13 – 3.07 (0.66H, m, C**1**-H’, A), 2.96 (0.66H, d, *J* = 17.0 Hz, C**5**-H’, A), 2.93 (0.33H, d, *J* = 17.0 Hz, C**5**-H’, B), 2.28 – 2.19 (1H, m, C**2**-H, A + B), 2.07 – 1.97 (1H, m, C**2**-H’, A + B), 1.63 (2H, s, C**6**-H_3, A), 1.56 (1H, s, C**6**-H_3, B); ^{13}C NMR (126 MHz, $CDCl_3$) δ 154.7 (C=O, B), 153.8 (C=O, A), 145.6 (ArC, A), 145.4 (ArC, B), 141.4 (ArC, A), 140.7 (ArC, B), 137.1(Cbz ArC, A), 136.9 (Cbz ArC, B), 132.6 (ArC, B), 132.4 (ArC, A), 128.7, 128.6, 128.1, 128.0, 127.8 (Cbz ArCH, A + B), 127.7 (ArCH, B), 127.6 (ArCH, A), 126.3 (ArCH, A), 126.1 (ArCH, B), 124.0 (ArCH, B), 123.9 (ArCH, A), 72.1 (C**4**, A), 71.3 (C**4**, B), 67.1 (Cbz CH_2, B), 66.4 (Cbz CH_2, A), 58.5 (C**3**, B), 57.0 (C**3**, A), 48.2 (C**1**, B), 47.0 (C**1**, A), 44.5 (C**5**, B), 42.9 (C**5**, A), 28.2 (C**2**, A), 28.1 (C**2**, B), 25.1 (C**6**, B), 23.6 (C**6**, A); *m/z* (ESI^+) HRMS: Calculated for $C_{20}H_{21}NO_2Cl$: 342.1255. Found $[M+H]^+$ 342.1262.

Benzyl 7-chloro-8a-methyl-3,3a,8,8a-tetrahydroindeno[2,1-*b*]pyrrole-1(2*H*)-carboxylate (2j’): R_f: 0.39 (15% EtOAc/hexane); ν_{max} / cm^{-1}: 2963 (m), 1697 (s), 1404 (s), 1354 (s), 1052 (m); *Mixture of rotamers A + B (3:2)* ^{1}H NMR (500 MHz, $CDCl_3$) δ 7.44 – 7.28 (5H, Cbz ArCH, A + B), 7.20 – 7.16 (1H, m, ArCH, A + B), 7.16 – 7.12 (1H, m, ArCH, A + B), 7.06 (1H, br d, *J* = 7.5 Hz, ArCH, A + B), 5.22 (0.8H, s, Cbz CH_2, B), 5.13 (0.6H, d, *J* = 12.5 Hz, Cbz CH, A), 5.05 (0.6H, d, *J* = 12.5 Hz, Cbz CH’, A), 3.87 (0.6H, d, *J* = 18.0 Hz, C**5**-H, A), 3.69 – 3.61 (1.4H, m, C**5**-H, B, C**1**-H, A + B), 3.56 – 3.51 (1H, m, C**3**-H, A + B), 3.25 – 3.19 (0.4H, m, C**1**-H’, B), 3.16 – 3.09 (0.6H, m, C**1**-H’, A), 3.03 (0.6H, d, *J* = 18.0 Hz, C**5**-H’, A), 2.98 (0.4H, d, *J* = 18.0 Hz, C**5**-H’, B), 2.28 – 2.20 (1H, m, C**2**-H, A + B), 2.07 – 2.00 (1H, m, C**2**-H’, A + B), 1.66 (1.8H, s, C**6**-H_3, A), 1.57 (1.2H, s, C**6**-H_3, B); ^{13}C NMR (126 MHz, $CDCl_3$) δ 154.5 (C=O, B), 153.8 (C=O, A), 145.6 (ArC, A + B), 141.1 (ArC, A), 140.5 (ArC, B), 137.1 (Cbz ArC, A + B), 131.3 (ArC, A + B), 128.7, 128.6, 128.4, 128.2, 128.1, 128.0, 127.9, 127.6 (5 × Cbz ArCH + 2 × ArCH, A + B), 122.0 (ArCH, A), 121.9 (ArCH, B), 70.9 (C**4**, A), 70.3 (C**4**, B), 67.2 (Cbz CH_2, B), 66.4 (Cbz CH_2, A), 59.4 (C**3**, B), 58.0 (C**3**, A), 48.1 (C**1**, B), 47.0 (C**1**, A), 44.3 (C**5**, B), 43.1 (C**5**, A), 28.6 (C**2**, A), 28.1 (C**2**, B), 25.2 (C**6**, B), 23.8 (C**6**, A); *m/z* (ESI^+) HRMS: Calculated for $C_{20}H_{21}NO_2Cl$: 342.1255. Found $[M+H]^+$ 342.1262.

*Note – the regioisomer assignment of **2j** and **2j’** is based upon the splitting pattern of the signals corresponding to the chloro-substituted aryl group. See the zoomed in sections on the relevant spectra.*

Ethyl (*E*)-3-(3,4-dimethoxyphenyl)-2-methylacrylate

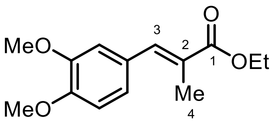

To a stirred solution of 3,4-dimethoxybenzaldehyde (1.66 g, 10.0 mmol) and triethyl 2-phosphonopropionate (2.79 g, 11.7 mmol) in anhydrous toluene (20 mL) at 0 °C was added NaO^tBu (1.40 g, 14.6 mmol) portionwise over 30 minutes. The reaction was allowed to warm to room temperature and stirred for 30 minutes. Upon completion, the reaction was quenched with 2M aq. HCl solution (10 mL) and diluted with water (20 mL). The aqueous layer was extracted with toluene (3 × 50 mL), and the combined organic layers washed with brine (50 mL), dried (Na_2SO_4), concentrated *in vacuo*. The title compound (2.52 g, 100%) was obtained as a colorless oil without purification. ν_{max} / cm^{-1}: 2959 (m), 1698 (s), 1514 (s), 1232 (s), 1107 (s); ^1H NMR (400 MHz, $CDCl_3$) δ 7.63 (1H, m, C**3**-H), 7.03 (1H ,dd, *J* = 8.5, 2.0 Hz, ArCH), 6.95 (1H, d, *J* = 2.0 Hz, ArCH), 6.89 (1H, d, *J* = 8.5 Hz, ArCH), 4.27 (2H, q, *J* = 7.0 Hz, OCH_2CH_3), 3.91 (3H, s, OCH_3), 3.90 (3H, s, OCH_3), 2.15 (3H, d, *J* = 1.5 Hz, C**4**-H_3), 1.35 (3H, t, *J* = 7.0 Hz, OCH_2CH_3); ^{13}C NMR (101 MHz, $CDCl_3$) δ 169.0 (C**1**), 149.4 (ArC), 148.8 (ArC), 138.7 (C**3**), 129.0 (ArC), 126.9 (C**2**), 123.2 (ArCH), 113.1 (ArCH), 111.1 (ArCH), 60.9 (OCH_2CH_3), 56.0 (2x OCH_3), 14.5 (OCH_2CH_3), 14.3 (C**4**); *m/z* ($APCI^+$) HRMS: Calculated for $C_{14}H_9O_4$: 251.1278. Found $[M+H]^+$ 251.1270.

(*E*)-3-(3,4-Dimethoxyphenyl)-2-methylprop-2-en-1-ol

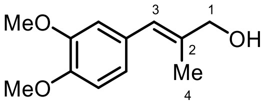

General procedure **F** was followed using the preceding ester (2.52 g, 10.1 mmol), $LiAlH_4$ (2M in THF, 10.1 mL) and anhydrous THF at 0 °C for 3 hours. The title compound (2.00 g, 95%) was obtained as colorless oil without purification. ν_{max} / cm^{-1}: 3387 (br s), 2935 (m), 2836 (m), 1513 (s), 1256 (s), 1239 (s), 1139 (s), 1025 (s); ^1H NMR (400 MHz, $CDCl_3$) δ 6.87 – 6.84 (2H, m, ArCH), 6.83 – 6.81 (1H, m, ArCH), 6.46 (1H, m, C**3**-H), 4.19 – 4.17 (2H, m, C**1**-H_2), 3.89 (3H, s, OCH_3), 3.88 (3H, s, OCH_3), 1.92 (3H, d, *J* = 1.5 Hz, C**4**-H_3); ^{13}C NMR (101 MHz, $CDCl_3$) δ 148.7 (ArC), 147.9 (ArC), 136.5 (C**2**), 130.6 (ArC), 125.1 (C**3**), 121.5 (ArCH), 112.4 (ArCH), 111.1 (ArCH), 69.4 (C**1**), 56.0 (OCH_3), 56.0 (OCH_3), 15.5 (C**4**); *m/z* ($APCI^+$) HRMS: Calculated for $C_{12}H_{15}O_2$: 191.1067. Found $[M+H-H_2O]^+$ 191.1059.

3-(3,4-Dimethoxyphenyl)-4-methylpent-4-enoic acid

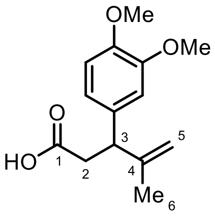

General procedure **E** was followed using the preceding alcohol (2.00 g, 9.60 mmol), triethyl orthoacetate (12.3 mL, 67.2 mmol) and pivalic acid (98.1 mg, 0.96 mmol) for 2 cycles. The subsequent hydrolysis was stirred at 60 °C for 16 hours. The title compound (1.85 g, 77%) was obtained as a yellow oil. ν_{max} / cm^{-1}: 2937 (br s), 1706 (s), 1515 (s), 1257 (s), 1236 (s), 1140 (s), 1027 (s); ^{1}H NMR (400 MHz, $CDCl_3$) δ 6.81 – 6.74 (2H, m, ArCH), 6.73 (1H, m, ArCH), 4.91 (1H, m, C**5**-H), 4.89 (1H, m, C**5**-H’), 3.85 (6H, s, 2 × OCH_3), 3.71 (1H, dd, *J* = 8.0, 8.0 Hz, C**3**-H), 2.87 (1H, dd, *J* = 15.5, 8.0 Hz, C**2**-H), 2.72 (1H, dd, *J* = 15.5, 8.0 Hz, C**2**-H’), 1.62 (3H, br s, C**6**-H_3); ^{13}C NMR (101 MHz, $CDCl_3$) δ 177.9 (C**1**), 149.1 (ArC), 148.0 (ArC), 146.8 (C**4**), 134.4 (ArC), 119.7 (ArCH), 111.3 (ArCH), 111.1 (ArCH), 110.5 (C**5**), 56.0 (OCH_3), 56.0 (OCH_3), 47.9 (C**3**), 39.3 (C**2**), 21.8 (C**6**); *m/z* (ESI^+) HRMS: Calculated for $C_{14}H_{19}O_4$: 251.1278. Found $[M+H]^+$ 251.1271.

3-(3,4-Dimethoxyphenyl)-4-methylpent-4-en-1-ol

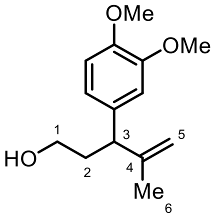

General procedure **F** was followed using the preceding ester (1.74 g, 6.95 mmol), $LiAlH_4$ (2M in THF, 6.95 mL) and anhydrous Et_2O at 0 °C for 3 hours. The title compound (916 mg, 56%) was obtained as a colorless oil without purification. ν_{max} / cm^{-1}: 3399 (br s), 2937 (s), 1515 (s), 1257 (s), 1141 (s), 1028 (s); ^{1}H NMR (400 MHz, $CDCl_3$) δ 6.81 – 6.74 (2H, m, ArCH), 6.73 (1H, m, ArCH), 4.93 (1H, m, C**5**-H), 4.85 (1H, m, C**5**-H’), 3.86 (6H, s, 2 × OCH_3), 3.66 – 3.54 (2H, m, C**1**-H_2), 3.35 (1H, dd, *J* = 7.5, 7.5 Hz, C**3**-H), 2.15 – 2.06 (1H, m, C**2**-H), 1.99 – 1.90 (1H, m, C**2**-H’), 1.59 (3H, br s, C**6**-CH_3); ^{13}C NMR (101 MHz, $CDCl_3$) δ 149.0 (ArC), 148.2 (C**4**), 147.7 (ArC), 135.7 (ArC), 119.9 (ArCH), 111.2 (ArCH), 111.1 (ArCH), 110.4 (C**5**), 61.5 (C**1**), 56.0 (OCH_3), 56.0 (OCH_3), 48.8 (C**3**), 36.0 (C**2**), 21.1 (C**6**); *m/z* (ESI^+) HRMS: Calculated for $C_{14}H_{21}O_3$: 237.1485. Found $[M+H]^+$ 237.1477.

Benzyl (3-(3,4-dimethoxyphenyl)-4-methylpent-4-en-1-yl)((perfluorobenzoyl)oxy)carbamate (1k)

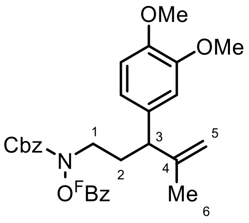

General procedure **I** was followed using the preceding alcohol (709 mg, 3.0 mmol), CbzNHO^{F}Bz (1.19 g, 3.30 mmol), triphenylphosphine (866 mg, 3.30 mmol) and diisopropyl azodicarboxylate (0.650 mL, 3.30 mmol). FCC (15 % EtOAc/hexane) gave title compound **1k** (1.23 g, 71%) as a colorless oil. ν_{max} / cm^{-1}: 2940 (m), 1782 (s), 1729 (s), 1498 (s), 1173 (s), 998 (s); ^{1}H NMR (400 MHz, $CDCl_3$) δ 7.38 – 7.29 (5H, m Cbz ArC<u>H</u>), 6.77 – 6.69 (3H, m, ArCH), 5.21 (2H, s, Cbz C<u>H$_2$</u>), 4.89 (1H, m, C**5**-<u>H</u>), 4.86 (1H, m, C**5**-<u>H</u>'), 3.85 (3H, s, OC<u>H$_3$</u>), 3.83 (3H, s, OC<u>H$_3$</u>), 3.75 (1H, ddd, *J* = 15.0, 8.5, 6.0 Hz, C**1**-<u>H</u>), 3.64 (1H, ddd, *J* = 15.0, 8.5, 6.0 Hz, C**1**-<u>H</u>'), 3.26 (1H, dd, *J* = 7.5, 7.5 Hz, C**3**-<u>H</u>), 2.22 – 2.12 (1H, m, C**2**-<u>H</u>), 2.08 – 1.98 (1H, m, C**2**-<u>H</u>'), 1.57 (3H, br s, C**6**-<u>H$_3$</u>); ^{13}C NMR (101 MHz, $CDCl_3$) δ 155.5 (Cbz <u>C</u>=O), 149.1 (Ar<u>C</u>), 147.9 (Ar<u>C</u>), 147.2 (C**4**), 135.3 (Cbz Ar<u>C</u>), 134.9 (Ar<u>C</u>), 128.7 (Cbz Ar<u>C</u>H), 128.6 (Cbz Ar<u>C</u>H), 128.2 (Cbz Ar<u>C</u>H), 119.9 (Ar<u>C</u>H), 111.2 (Ar<u>C</u>H), 111.0 (Ar<u>C</u>H), 110.7 (C**5**), 68.9 (Cbz <u>C</u>H$_2$), 56.0 (O<u>C</u>H$_3$), 55.9 (O<u>C</u>H$_3$), 50.2 (C**1**), 49.2 (C**3**), 30.4 (C**2**), 21.3 (C**6**); ^{19}F NMR (377 MHz, $CDCl_3$) δ -136.0 – 136.1 (2F, m), -146.0 (1F, tt, *J* = 21.0, 5.5 Hz), -159.2 – -159.3 (2F, m); *m/z* (ESI$^+$) HRMS: Calculated for $C_{29}H_{30}F_5N_2O_6$: 597.2019. Found $[M+NH_4]^+$ 597.1997.

The ^{13}C signals corresponding to the pentafluorobenzoyl group could not be resolved due to their weak intensity.

Benzyl 5,6-dimethoxy-8a-methyl-3,3a,8,8a-tetrahydroindeno[2,1-*b*]pyrrole-1(2*H*)-carboxylate (2k) and benzyl 6,7-dimethoxy-8a-methyl-3,3a,8,8a-tetrahydroindeno[2,1-*b*]pyrrole-1(2*H*)-carboxylate (2k')

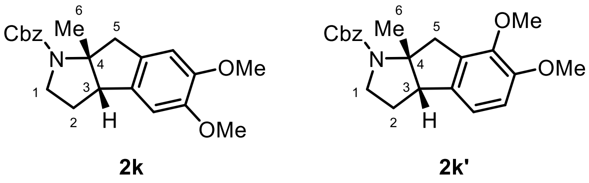

(<u>Ratio of **2k**:**2k'** = 1.7:1</u>) General procedure **J** was followed using cyclization substrate **1k** (58.0 mg, 0.1 mmol), Pd_2dba_3 (2.29 mg, 0.0025 mmol), CgPPh (4.38 mg, 0.015 mmol), sodium 2-methoxybenzoate (1.74 mg, 0.01 mmol), triethylamine (1.40 μL) and dibutylether (1 mL). The reaction was stirred at 130 °C for 48 hours. FCC (75/15/10 hexane/CH_2Cl_2/acetone) gave title compound **2k** (18.0 mg, 49%) and title compound **2k'** (10.6 mg, 29%) as colorless oils.

(Ratio of **2k**:**2k'** = 1:1.5) General procedure **J** was followed using cyclization substrate **1k** (58.0 mg, 0.1 mmol), Pd_2dba_3 (2.29 mg, 0.0025 mmol), CgPPh (4.38 mg, 0.015 mmol), sodium 2-nitrobenzoate (1.89 mg, 0.01 mmol), triethylamine (1.40 µL) and dibutylether (1 mL). The reaction was stirred at 130 °C for 48 hours. FCC (75/15/10 hexane/CH_2Cl_2/acetone) gave title compound **2k** (8.0 mg, 22%) and title compound **2k'** (11.6 mg, 32%) as colorless oils.

Benzyl 5,6-dimethoxy-8a-methyl-3,3a,8,8a-tetrahydroindeno[2,1-*b*]pyrrole-1(2*H*)-carboxylate (2k): R_f: 0.22 (25% EtOAc/hexane); ν_{max} / cm^{-1}: 2960 (m), 1696 (s), 1507 (s), 1405 (s), 1102 (s); *Mixture of rotamers A + B (2:1)* ^1H NMR (500 MHz, $CDCl_3$) δ 7.43 – 7.27 (5H, m, Cbz ArCH, A + B), 6.69 (0.66H, s, ArCH, A), 6.68 (1H, s, ArCH, A + B), 6.63 (0.33H, s, ArCH, B), 5.22 (0.33H, d, *J* = 12.5 Hz, Cbz CH, B), 5.18 (0.33H, d, *J* = 12.5 Hz, Cbz CH', B), 5.12 (0.66H, d, *J* = 12.5 Hz, Cbz CH, A), 5.04 (0.66H, d, *J* = 12.5 Hz, Cbz CH', A), 3.87 – 3.82 (6H, m, 2 × OCH_3, A + B), 3.72 (0.66H, d, *J* = 17.0 Hz, C**5**-H, A), 3.68 – 3.61 (1H, m, C**1**-H, A + B), 3.50 – 3.42 (1.33H, m, C**5**-H, B, C**3**-H, A + B), 3.26 (0.33H, ddd, *J* = 11.0, 9.5, 6.5 Hz, C**1**-H', B), 3.12 (0.66H, ddd, *J* = 10.5, 10.5, 6.5 Hz, C**1**-H', A), 2.96 (0.66H, d, *J* = 17.0 Hz, C**5**-H', A), 2.93 (0.33H, d, *J* = 16.5 Hz, C**5**-H', B), 2.24 – 2.15 (1H, m, C**2**-H, A + B), 2.05 – 1.95 (1H, m, C**2**-H', A + B), 1.63 (2H, s, C**6**-H_3, A), 1.56 (1H, s, C**6**-H_3, B); ^{13}C NMR (126 MHz, $CDCl_3$) δ 154.8 (Cbz C=O, B), 153.8 (Cbz C=O, A), 149.0 (ArC, A + B), 148.6 (ArC, B), 148.5 (ArC, A), 137.2 (Cbz ArC, A), 137.0 (Cbz ArC, B), 134.8 (ArC, A), 134.7 (ArC, B), 134.5 (ArC, A), 133.7 (ArC, B), 128.6, 128.5, 128.1, 128.0, 127.9, 127.8 (Cbz ArCH, A + B), 108.1 (ArCH, A), 106.8 (ArCH, B), 106.8 (ArCH, A + B), 72.2 (C**4**, A), 71.5 (C**4**, B), 67.0 (Cbz CH_2, B), 66.3 (Cbz CH_2, A), 58.8 (C**3**, B), 57.3 (C**3**, B), 56.3 (OCH_3, A), 56.3 (OCH_3, B), 56.1 (OCH_3, B), 56.1 (OCH_3, A), 48.2 (C**1**, B), 47.1 (C**1**, A), 45.1 (C**5**, B), 43.5 (C**5**, A), 28.4 (C**2**, A), 28.4 (C**2**, B), 25.3 (C**6**, B), 23.8 (C**6**, A); *m/z* (ESI^+) HRMS: Calculated for $C_{22}H_{26}NO_4$: 368.1856. Found $[M+H]^+$ 368.1851.

Some signal broadening was observed due to amide-like resonance.

Benzyl 6,7-dimethoxy-8a-methyl-3,3a,8,8a-tetrahydroindeno[2,1-*b*]pyrrole-1(2*H*)-carboxylate (2k'): R_f: 0.41 (25% EtOAc/hexane); ν_{max} / cm^{-1}: 2960 (m), 2937 (m), 1697 (s), 1489 (s), 1404 (s), 1266 (s), 1081 (s); *Mixture of rotamers A + B (2:1)* ^1H NMR (500 MHz, $CDCl_3$) δ 7.44 – 7.27 (5H, m, Cbz ArCH, A + B), 6.83 (1H, br d, *J* = 8.0 Hz, ArCH, A + B), 6.77 (1H, br d, *J* = 8.0 Hz, ArCH, A + B), 5.21 (0.66H, br s, Cbz CH_2, B), 5.10 (0.66H, d, *J* = 12.5 Hz, Cbz CH, A), 5.05 (0.66H, d, *J* = 12.5 Hz, Cbz CH', A), 3.89 (0.66H, d, *J* = 17.5 Hz, C**5**-H, A), 3.83 (3H, s, OCH_3, A + B), 3.80 (2H, s, OCH_3, A), 3.75 (1H, s, OCH_3, B), 3.67 – 3.58 (1.33H, m, C**1**-H, A + B, C**5**-H, B), 3.45 – 3.40 (1H, m, C**3**-H, A + B), 3.25 (0.33H, ddd, *J* = 11.0, 9.5, 6.5 Hz, C**1**-H', B), 3.12 (0.66H, ddd, *J* = 10.5, 10.5, 6.5 Hz, C**1**-H', A), 2.98 (0.66H, d, *J* = 17.5 Hz, C**5**-H', A), 2.95 (0.33H, d, *J* = 17.5 Hz, C**5**-H', B), 2.23 – 2.14 (1H, m, C**2**-H, A + B), 2.04 – 1.96 (1H, m, C**2**-H', A + B), 1.64 (2H, s, C**6**-H_3, A), 1.55 (1H, s, C**6**-H_3, B); ^{13}C NMR (126 MHz, $CDCl_3$) δ 154.8 (Cbz C=O, B), 153.8 (Cbz C=O, A), 151.8 (ArC, A + B), 145.7 (ArC, A), 145.5 (ArC, B), 137.4 (ArC, A), 137.3 (Cbz ArC, A), 137.2 (ArC, B), 137.1 (Cbz ArC, B), 135.6 (ArC, A), 135.1 (ArC, B), 128.7, 128.5, 128.2, 128.1, 127.9, 127.9 (Cbz ArCH), 118.6 (ArCH,

B), 118.5 (ArC̲H, A), 111.9 (ArC̲H, B), 111.7 (ArC̲H, A), 72.2 (C**4**, A), 71.5 (C**4**, B), 67.0 (Cbz, C̲H_2, B), 66.3 (Cbz, C̲H_2, A), 60.3 (OC̲H_3, A + B), 58.1 (C**3**, B), 56.7 (C**3**, A), 56.3 (OC̲H_3, A + B), 48.2 (C**1**, B), 47.1 (C**1**, A), 41.9 (C**5**, B), 40.7 (C**5**, A), 28.5 (C**2**, A), 28.2 (C**2**, B), 25.2 (C**6**, B), 23.8 (C**6**, A); *m/z* (ESI^+) HRMS: Calculated for $C_{22}H_{26}NO_4$: 368.1856. Found $[M+H]^+$ 368.1856.

Some signal broadening was observed due to amide-like resonance.

*Note – the regioisomer assignment of **2k** and **2k'** is based upon NMR analysis after Cbz deprotection. The hydrogenative removal of the Cbz group on **2k** was carried out using the following procedure:*

5,6-Dimethoxy-8a-methyl-1,2,3,3a,8,8a-hexahydroindeno[2,1-*b*]pyrrole

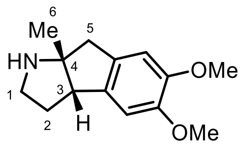

To a solution of precursor **2k** (24.5 mg, 0.067 mmol) in MeOH (1 mL) was added 5% Pd/C (5 mg). Hydrogen gas was bubbled through the solution for 2 minutes. The reaction was stirred vigorously under a balloon of hydrogen for 20 hours. Upon completion, the reaction was filtered through celite, washing with EtOAc. The filtrate was concentrated *in vacuo* to give the title compound (9.50 mg, 61%) as a colorless oil.

^{1}H NMR analysis of the crude material showed 2 characteristic singlets in the aromatic region, consistent with the regioisomer assignment of the title compound drawn above. ^{1}H NMR (400 MHz, $CDCl_3$) δ 6.67 (1H, s, ArCH̲), 6.64 (1H, s, ArCH̲), 3.85 (3H, s, OC$\underline{H_3}$), 3.84 (3H, s, OC$\underline{H_3}$), 3.63 (1H, d, J = 17.5 Hz, C**5**-H̲), 3.51 (1H, m, C**3**-H̲), 3.42 (1H, ddd, J = 11.0, 7.0, 2.5 Hz, C**1**-H̲), 3.11 (1H, d, J = 17.5 Hz, C**5**-H̲'), 2.93 (1H, ddd, J = 11.0, 11.0, 6.2 Hz, C**1**-H̲'), 2.53 – 2.42 (1H, m, C**2**-H̲), 2.07 – 2.00 (1H, m, C**2**-H̲'), 1.76 (3H, s, C**6**-$\underline{H_3}$).

(*E*)-3-(Furan-3-yl)-2-methylacrylaldehyde

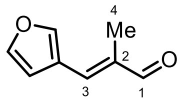

An adapted general procedure **A** was followed using 3-furancarboxaldehyde (2.00 g, 20.8 mmol), propionaldehyde (3.00 mL, 41.6 mmol) and KOH (1.17 g, 20.8 mmol) in EtOH (52 mL). FCC (5 – 10% EtOAc/hexane) gave the title compound (1.85 g, 65%) as a yellow oil (*contains 10% aldehyde impurity*). R*f*: 0.21 (5% EtOAc/hexane); ν_{max} / cm^{-1}: 3133 (m), 2926 (m), 1678 (s), 1627 (s); ^{1}H NMR (400 MHz, $CDCl_3$) δ 9.51 (1H, s, C**1**-H̲), 7.77 (1H, br s, ArCH̲), 7.51 (1H, m, ArCH̲), 7.10 (1H, br s, ArCH̲), 6.69 (1H, m, C**3**-H̲), 2.01 – 2.00 (3H, m, C**4**-$\underline{H_3}$); ^{13}C NMR (101 MHz, $CDCl_3$) δ 194.7 (C**1**), 144.9 (ArC̲H), 144.3 (ArC̲H), 140.1 (ArC̲H), 137.2 (C**2**), 122.3 (ArC̲), 110.7 (C**3**), 10.9 (C**4**); *m/z* ($APCI^+$) HRMS: Calculated for $C_8H_9O_2$: 137.0597. Found $[M+H]^+$: 137.0596.

(*E*)-3-(Furan-3-yl)-2-methylprop-2-en-1-ol

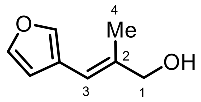

General procedure **C** was followed using the preceding aldehyde (1.74 g, 12.8 mmol) and $NaBH_4$ (507 mg, 13.4 mmol). FCC (15% EtOAc/toluene) gave the title compound (1.55 g, 88%) as a pale yellow oil. R*f*: 0.26 (15% EtOAc/toluene); ν_{max} / cm^{-1}: 3404 (br s), 2921 (s), 1754 (s), 1024 (s); ^1H NMR (400 MHz, $CDCl_3$) δ 7.45 (1H, m, ArC<u>H</u>), 7.39 (1H, m, ArC<u>H</u>), 6.47 (1H, m, ArC<u>H</u>), 6.25 (1H, m, C**3**-<u>H</u>), 4.15 (2H, br s, C**1**-$\underline{H_2}$), 1.89 (3H, br s, C**4**-$\underline{H_3}$); ^{13}C NMR (101 MHz, $CDCl_3$) δ 142.8 (Ar<u>C</u>H), 140.9 (Ar<u>C</u>H), 136.9 (C**2**), 122.6 (Ar<u>C</u>), 115.6 (C**3**), 111.1 (Ar<u>C</u>H), 69.1 (C**1**), 15.9 (C**4**); *m/z* ($APCI^+$) HRMS: Calculated for C_8H_9O: 121.0648. Found $[M+H-H_2O]^+$: 121.0644.

3-(Furan-3-yl)-4-methylpent-4-enoic acid

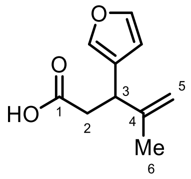

General procedure **E** was followed using the preceding alcohol (1.39 g, 10.0 mmol), pivalic acid (102 mg, 1.00 mmol) and triethyl orthoacetate (12.8 mL, 70.0 mmol) for 1 cycle. The subsequent hydrolysis was stirred at 60 °C for 5 hours. The title compound was obtained (1.30 g, 72%) as a dark orange oil. ν_{max} / cm^{-1}: 2975 (br s), 1709 (s); ^1H NMR (400 MHz, $CDCl_3$) δ 7.35 (1H, m, ArC<u>H</u>), 7.26 (1H, m, ArC<u>H</u>), 6.24 (1H, m, ArC<u>H</u>), 4.89 (1H, m, C**5**-<u>H</u>), 4.86 (1H, m, C**5**-<u>H</u>’), 3.73 (1H, dd, *J* = 8.0, 8.0 Hz, C**3**-<u>H</u>), 2.78 (1H, dd, *J* = 15.5, 7.5 Hz, C**2**-<u>H</u>), 2.70 (1H, dd, *J* = 15.5, 7.5 Hz, C**2**-<u>H</u>’); ^{13}C NMR (101 MHz, $CDCl_3$) δ 177.6 (C**1**), 145.9 (C**4**), 143.2 (Ar<u>C</u>H), 139.3 (Ar<u>C</u>H), 126.1 (Ar<u>C</u>), 111.6 (C**5**), 110.1 (Ar<u>C</u>H), 39.7 (C**3**), 38.5 (C**2**), 20.6 (C**6**); *m/z* ($APCI^+$) HRMS: Calculated for $C_{10}H_{13}O_3$: 181.0859. Found $[M+H]^+$: 181.0856.

3-(Furan-3-yl)-4-methylpent-4-en-1-ol

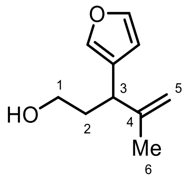

General procedure **F** was followed using the preceding carboxylic acid (1.30 g, 7.21 mmol), $LiAlH_4$ (2M in THF, 7.2 mL) and anhydrous THF at 0 °C for 3 hours. The title compound (1.19 g, 99%) was obtained as a colorless oil. ν_{max} / cm^{-1}: 3346 (br s), 2941 (s), 1501 (s), 1048 (s); ^1H NMR (400 MHz, $CDCl_3$) δ 7.35 (1H, m, ArC<u>H</u>), 7.25 (1H, m, ArC<u>H</u>), 6.25 (1H, m, ArC<u>H</u>), 4.91 (1H, m, C**5**-<u>H</u>), 4.83

(1H, m, C**5**-H’), 3.68 – 3.62 (2H, m, C**1**-$\underline{H_2}$), 3.39 (1H, dd, *J* = 7.5, 7.5 Hz, C**3**-H), 2.03 – 1.87 (2H, m, C**2**-$\underline{H_2}$), 1.63 (3H, br s, C**6**-$\underline{H_3}$), 1.37 (1H, t, *J* = 5.5 Hz, OH); ^{13}C NMR (101 MHz, $CDCl_3$) δ 147.2 (C**4**), 143.0 (ArCH), 139.2 (ArCH), 127.2 (ArC), 111.6 (C**5**), 110.3 (ArCH), 61.3 (C**1**), 40.5 (C**3**), 35.1 (C**2**), 19.6 (C**6**); *m/z* (APCI$^+$) HRMS: Calculated for $C_{10}H_{15}O_2$: 167.1067. Found $[M+H]^+$: 167.1062.

Benzyl (3-(furan-3-yl)-4-methylpent-4-en-1-yl)((perfluorobenzoyl)oxy)carbamate (1l)

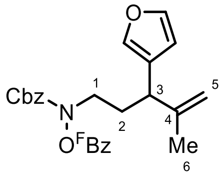

General procedure **I** was followed using CbzNHO^{F}Bz (1.38 g, 3.82 mmol), the preceding alcohol (577 mg, 3.47 mmol), triphenylphosphine (1.00 g, 3.82 mmol) and diisopropyl azodicarboxylate (0.752 mL, 3.82 mmol). FCC (3 – 5% EtOAc/hexane) gave title compound **1l** (1.59 mg, 90%) as a colorless oil. R_f: 0.17 (3% EtOAc/hexane); ν_{max} / cm^{-1}: 2946 (m), 1783 (s), 1730 (s), 1524 (s), 1500 (s), 1175 (s); ^{1}H NMR (400 MHz, $CDCl_3$) δ 7.39 – 7.30 (6H, m, Cbz 5 × ArCH + furan ArCH), 7.21 (1H, m, furan ArCH), 6.21 (1H, m, furan ArCH), 4.86 (1H, m, C**5**-H), 4.83 (1H, m, C**5**-H’), 3.80 – 3.65 (2H, m, C**1**-$\underline{H_2}$), 3.30 (1H, dd, *J* = 7.5, 7.5 Hz, C**3**-H), 2.11 – 1.95 (2H, m, C**2**-$\underline{H_2}$), 1.61 (3H, br s, C**6**-$\underline{H_3}$); ^{13}C NMR (101 MHz, $CDCl_3$) δ 155.5 (C=O), 146.2 (C**4**), 143.2 (furan ArCH), 139.3 (furan ArCH), 135.3 (Cbz ArC), 128.7 (Cbz ArCH), 128.7 (Cbz ArCH), 128.3 (Cbz ArCH), 126.6 (furan ArC), 111.9 (C**5**), 110.1 (furan ArCH), 69.0 (Cbz CH_2), 50.0 (C**1**), 40.9 (C**3**), 29.5 (C**2**), 19.7 (C**6**); ^{19}F NMR (377 MHz, $CDCl_3$) δ -136.0 – -136.2 (2F, m), -146.0 (1F, tt, *J* = 21.0, 5.5 Hz), -159.1 – -159.3 (2F, m); *m/z* (ESI$^+$) HRMS: Calculated for $C_{25}H_{20}F_5NNaO_5$: 532.1154. Found $[M+Na]^+$: 532.1155.

The carbon signals corresponding to the pentafluorobenzoyl group could not be resolved due to their weak intensity.

Benzyl 6a-methyl-4,5,6a,7-tetrahydrofuro[3',2':3,4]cyclopenta[1,2-*b*]pyrrole-6(3b*H*)-carboxylate (2l)

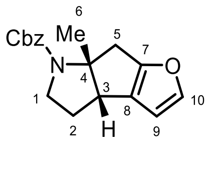

General procedure **J** was followed using cyclization substrate **1l** (50.9 mg, 0.10 mmol), $Pd_2(dba)_3$ (2.29 mg, 0.0025 mmol), CgPPh (4.38 mg, 0.015 mmol), sodium benzoate (1.44 mg, 0.01 mmol), triethylamine (1.40 μL) and dibutyl ether (1 mL). The reaction was stirred at 130 °C for 48 hours. FCC (5 – 10% EtOAc/hexane) gave title compound **2l** (18.4 mg, 62%) as a colorless oil. R_f: 0.35 (10% EtOAc/hexane); ν_{max} / cm^{-1}: 2963 (m), 1692 (s), 1402 (s), 1349 (s); *mixture of rotamers A + B (2:1)* ^{1}H NMR (400 MHz, $CDCl_3$) δ 7.44 – 7.27 (6H, m, Cbz 5 × ArCH + C**10**-H, A + B), 6.24 – 6.19 (1H, m,

C**9**-H, A + B), 5.22 – 5.16 (0.7H, m, Cbz CH_2, B), 5.15 – 5.05 (1.3H, m, Cbz CH_2, A), 3.71 – 3.61 (1H, m, C**1**-H, A + B), 3.50 – 3.37 (1H, m, C**1**-H', B, C**5**-H, A), 3.36 – 3.27 (0.66H, m, C**1**-H', A), 3.26 – 3.16 (1.33H, m, C**5**-H, B, C**3**-H, A + B), 2.87 – 2.74 (1H, m, C**5**-H', A + B), 2.15 – 2.01 (1H, m, C**2**-H, A + B), 1.88 – 1.73 (1H, m, C**2**-H', A + B), 1.66 (2H, s, C**6**-H_3, A), 1.58 (1H, s, C**6**-H_3, B); ^{13}C NMR (101 MHz, $CDCl_3$) δ 157.0 (C**7**, A), 156.1 (C**7**, B), 154.6 (C=O, B), 153.8 (C=O, A), 146.5 (C**10**-H, B), 146.4 (C**10**-H, A), 137.2 (Cbz ArC, A), 136.8 (Cbz ArC, B), 128.7, 128.6, 128.2, 128.1, 128.0, 127.9 (Cbz ArCH, A + B), 124.7 (C**8**-H, B), 124.6 (C**8**-H, A), 107.3 (C**9**-H, B), 107.2 (C**9**-H, A), 76.8 (C**4**, A), 75.9 (C**4**, B), 67.2 (Cbz CH_2, B), 66.4 (Cbz CH_2, A), 51.9 (C**3**, B), 50.4 (C**3**, A), 48.4 (C**1**, B), 47.4 (C**1**, A), 39.6 (C**5**, B), 38.1 (C**5**, A), 28.2 (C**2**, A + B), 26.4 (C**6**, B), 25.0 (C**6**, A); *m/z* (ESI^+) HRMS: Calculated for $C_{18}H_{20}NO_3$: 298.1438. Found $[M+H]^+$: 298.1445.

*Note - the regioisomer assignment is based upon the retention of the signal corresponding to C**9** on **2l** (^{1}H δ 6.21 and ^{13}C δ 110.1 in starting material **1l**, translating to ^{1}H δ 6.21 and ^{13}C δ 107.3 in product **2l**.) This is further backed up by the disappearance of a ^{1}H signal at C**7**.*

(*E*)-2-Methyl-3-(pyridin-3-yl)acrylaldehyde

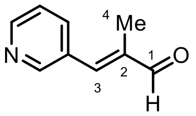

3-Pyridinecarboxaldehyde (4.8 mL, 50.0 mmol) was added to a suspension of KOH (0.50 g, 8.90 mmol) in DMF (65 mL). A solution of propionaldehyde (4.4 mL, 60.0 mmol) in DMF (19 mL) was then added dropwise over the course of 1 hour. The reaction was stirred for a further 3 hours. Upon completion, the reaction was diluted with EtOAc (200 mL), and the organic phase was washed with brine/water 1:1 (5 × 100 mL). The organic layer was dried (Na_2SO_4) and concentrated *in vacuo* to give the title compound (5.94 g, 81%), which was used in the next step without further purification. ^{1}H NMR (400 MHz, $CDCl_3$) δ 9.63 (1H, s, C**1**-H), 8.77 (1H, d, J = 2.0 Hz, ArCH), 8.62 (1H, dd, J = 5.0, 2.0 Hz, ArCH), 7.85 (1H, ddd, J = 8.0, 2.0, 2.0 Hz, ArCH), 7.40 (1H, dd, J = 8.0, 5.0 Hz, ArCH), 7.25 (1H, s, C**3**-H), 2.09 (3H, d, J = 1.0 Hz, C**4**-H_3); ^{13}C NMR (101 MHz, $CDCl_3$) δ 194.9 (C**1**), 151.0 (ArCH), 150.3 (ArCH), 145.6 (C**3**), 140.4 (C**2**), 136.6 (ArCH), 131.1 (ArC), 123.7 (ArCH), 11.1 (C**4**).

The spectroscopic properties were consistent with the data available in the literature.[29]

(*E*)-2-Methyl-3-(pyridin-3-yl)prop-2-en-1-ol

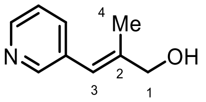

General procedure **C** was followed using the preceding aldehyde (5.94 g, 40.7 mmol) and $NaBH_4$ (1.65 g, 42.7 mmol). FCC (0 – 10% MeOH/CH_2Cl_2) gave the title compound (3.70 g, 60%) as a colorless oil.

^{1}H NMR (400 MHz, $CDCl_3$) δ 8.54 (1H, s, ArC<u>H</u>), 8.45 (1H, d, *J* = 4.0 Hz, ArC<u>H</u>), 7.59 (1H, d, *J* = 8.0 Hz, ArC<u>H</u>), 7.28 – 7.26 (1H, m, ArC<u>H</u>), 6.50 (1H, s, C**3**-<u>H</u>), 4.22 (2H, d, *J* = 5.0 Hz, C**1**-<u>H$_2$</u>), 1.89 (3H, s, C**4**-<u>H$_3$</u>), 1.75 (1H, br t, *J* = 5.0 Hz, O<u>H</u>); ^{13}C NMR (101 MHz, $CDCl_3$) δ 150.0 (Ar<u>C</u>H), 147.3 (Ar<u>C</u>H), 140.8 (C**2**), 136.1 (Ar<u>C</u>H), 133.6 (Ar<u>C</u>), 123.3 (Ar<u>C</u>H), 120.8 (C**3**), 68.3 (C**1**), 15.4 (C**4**).

The spectroscopic properties were consistent with the data available in the literature.[29]

Ethyl 4-methyl-3-(pyridin-3-yl)pent-4-enoate

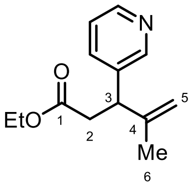

General procedure **D** was followed using the preceding alcohol (1.33 g, 8.92 mmol), triethyl orthoacetate (16.7 mL, 89.2 mmol) and propionic acid (135 μL, 1.78 mmol) to give the title compound (1.72 g, 88%), which was used in the next step without further purification. R$_f$: 0.22 (30% EtOAc/hexane); ν_{max} / cm^{-1}: 2979 (m), 1734 (s), 1424 (m), 1155 (m); ^{1}H NMR (400 MHz, $CDCl_3$) δ 8.49 (1H, s, ArC<u>H</u>), 8.47 (1H, d, *J* = 5.0 Hz, ArC<u>H</u>), 7.53 (1H, d, *J* = 7.0 Hz, ArC<u>H</u>), 7.22 (1H, dd, *J* = 7.0, 5.0 Hz, ArC<u>H</u>), 4.94 – 4.92 (2H, m, C**5**-<u>H$_2$</u>), 4.06 (2H, q, *J* = 7.0 Hz, OC<u>H$_2$</u>CH$_3$), 3.80 (1H, dd, *J* = 8.0, 8.0 Hz, C**3**-<u>H</u>), 2.89 – 2.84 (1H, m, C**2**-<u>H</u>), 2.73 – 2.67 (1H, m, C**2**-<u>H</u>’), 1.62 (3H, s, C**6**-<u>H$_3$</u>), 1.16 (3H, t, *J* = 7.0 Hz, OCH$_2$C<u>H$_3$</u>); ^{13}C NMR (101 MHz, $CDCl_3$) δ 171.7 (C**1**), 149.8 (Ar<u>C</u>H), 148.4 (Ar<u>C</u>H), 145.8 (C**4**), 137.5 (Ar<u>C</u>), 135.2 (Ar<u>C</u>H), 123.5 (Ar<u>C</u>H), 111.5 (C**5**), 60.7 (O<u>C</u>H$_2$CH$_3$), 46.2 (C**3**), 39.0 (C**2**), 21.7 (C**6**), 14.2 (OCH$_2$<u>C</u>H$_3$); *m/z* (ESI$^+$) HRMS: Calculated for $C_{13}H_{17}NO_2$: 220.1332. Found [M+H]$^+$: 220.1328.

4-Methyl-3-(pyridin-3-yl)pent-4-en-1-ol

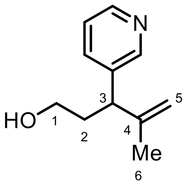

General procedure **F** was followed using the preceding ester (1.72 g, 7.84 mmol), $LiAlH_4$ (1M in THF, 11.8 mL) and anhydrous THF at 0 °C for 1.5 hours. FCC (0 – 4% MeOH/CH_2Cl_2) gave the title compound (882 mg, 63%) as a colorless oil. R$_f$: 0.43 (5% MeOH/CH_2Cl_2); ν_{max} / cm^{-1}: 3286 (br s), 2938 (m), 1646 (m), 1423 (s), 1128 (s); ^{1}H NMR (400 MHz, $CDCl_3$) δ 8.49 (1H, s, ArC<u>H</u>), 8.46 (1H, d, *J* = 5.0 Hz, ArC<u>H</u>), 7.54 (1H, d, *J* = 8.0 Hz, ArC<u>H</u>), 7.23 (1H, dd, *J* = 8.0, 5.0 Hz, ArC<u>H</u>), 4.98 (1H, m, C**5**-<u>H</u>), 4.92 (1H, m, C**5**-<u>H</u>’), 3.67 – 3.53 (2H, m, C**1**), 3.48 (1H, dd, *J* = 8.0, 8.0 Hz, C**3**-<u>H</u>), 2.21 – 2.13 (1H, m, C**2**-<u>H</u>), 2.00 – 1.92 (1H, m, C**2**-<u>H</u>’), 1.59 (3H, s, C**6**-<u>H$_3$</u>); ^{13}C NMR (101 MHz, $CDCl_3$) δ 149.8

(ArCH), 147.9 (ArCH), 146.7 (C**4**), 138.6 (ArC), 135.3 (ArCH), 123.6 (ArCH), 111.7 (C**5**), 60.6 (C**1**), 46.3 (C**3**), 35.5 (C**2**), 21.1 (C**6**); *m/z* (ESI$^+$) HRMS: Calculated for $C_{11}H_{16}NO$: 178.1226. Found $[M+H]^+$: 178.1222.

Benzyl (4-methyl-3-(pyridin-3-yl)pent-4-en-1-yl)((perfluorobenzoyl)oxy)carbamate (1m)

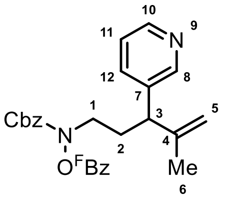

General procedure **I** was followed using CbzNHO^{F}Bz (797 mg, 2.21 mmol), the preceding alcohol (391 mg, 2.21 mmol), triphenylphosphine (643 mg, 2.43 mmol) and diisopropyl azodicarboxylate (0.48 mL, 2.43 mmol). FCC (2 – 5% acetone/toluene) gave title compound **1m** (584 mg, 51%) as a colorless oil. R_f: 0.26 (5% acetone/toluene); ν_{max} / cm^{-1}: 1786 (m), 1737 (m), 1524 (m), 1500 (s), 1327 (m), 1174 (s); ^{1}H NMR (400 MHz, $CDCl_3$) δ 8.47 – 8.46 (2H, m, C**8**-H + C**10**-H), 7.50 (1H, ddd, *J* = 8.0, 2.0, 2.0 Hz, C**12**-H), 7.38 – 7.30 (5H, m, Cbz ArCH), 7.21 – 7.18 (1H, m, C**11**-H), 5.20 (2H, s, Cbz CH_2), 4.94 (1H, m, C**5**-H), 4.92 (1H, m, C**5**-H'), 3.78 – 3.64 (2H, m, C**1**-H_2), 3.39 (1H, dd, *J* = 8.0, 8.0 Hz, C**3**-H_2), 2.27 – 2.18 (1H, m, C**2**-H), 2.09 – 2.00 (1H, m, C**2**-H'); ^{13}C NMR (101 MHz, $CDCl_3$) δ 155.4 (Cbz C=O), 149.7 (C**8**), 148.3 (C**10**), 145.6 (C**4**), 137.8 (C**7**), 135.2 (Cbz ArC), 135.1 (C**12**), 128.7, 128.3 (5 × Cbz ArCH), 123.6 (C**11**), 112.1 (C**5**), 69.0 (Cbz CH_2), 49.8 (C**1**), 47.1 (C**3**), 29.9 (C**2**), 21.0 (C**6**); ^{19}F NMR (400 MHz, $CDCl_3$) δ -135.9 – -136.0 (2F, m), -145.6 (1F, tt, *J* = 21.0, 5.0 Hz), -158.9 – -159.0 (2F, m); *m/z* (ESI$^+$) HRMS: Calculated for $C_{26}H_{22}F_5N_2O_4$: 521.1494. Found $[M+H]^+$: 521.1479.

The ^{13}C NMR signals corresponding to the pentafluorobenzoyl group could not be resolved due to their weak intensity.

Benzyl 8a-methyl-3,3a,8,8a-tetrahydropyrrolo[3',2':3,4]cyclopenta[1,2-*c*]pyridine-1(2*H*)-carboxylate (2m)

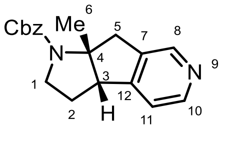

General procedure **J** was followed using cyclization substrate **1m** (33.20 mg, 0.064 mmol), $Pd_2(dba)_3$ (2.93 mg, 0.0032 mmol), CgPPh (9.64 mg, 0.032 mmol), sodium benzoate (0.92 mg, 0.0064 mmol), triethylamine (0.90 μL) and dibutyl ether (0.64 mL). The reaction was stirred at 150 °C for 48 hours. FCC (40 – 50% EtOAc/hexane) gave title compound **2m** (6.8 mg, 34%) as a colorless oil. R_f: 0.27 (50% EtOAc/hexane); ν_{max} / cm^{-1}: 1693 (s), 1403 (s), 1354 (m), 1102 (m), 1052 (m); *mixture of rotamers A + B (2:1)* ^{1}H NMR (500 MHz, $CDCl_3$) δ 8.45 – 8.42 (2H, m, C**8**-H + C**10**-H, A + B), 7.41 – 7.27 (5H, m,

Cbz ArCH, A + B), 7.14 (0.66H, d, *J* = 5.0 Hz, C**11**-H, A), 7.08 (0.33H, d, *J* = 5.0 Hz, C**11**-H, B), 5.23 – 5.17 (0.66H, m, Cbz CH_2, B), 5.11 (0.66H, d, *J* = 12.5 Hz, Cbz CH, A), 5.04 (0.66H, d, *J* = 12.5 Hz, Cbz CH', A), 3.86 (0.66H, d, *J* = 18.0 Hz, C**5**-H, A), 3.72 – 3.64 (1H, m, C**1**-H, A + B), 3.59 – 3.54 (1.33H, m, C**3**-H, A + B, C**5**-H, B), 3.27 – 3.21 (0.33H, m, C**1**-H', B), 3.13 – 3.08 (0.66H, m, C**1**-H', A), 3.00 (0.66H, d, *J* = 18.0 Hz, C**5**-H', A), 2.97 (0.33H, d, *J* = 17.0 Hz, C**5**-H', B), 2.34 – 2.25 (1H, m, C**2**-H, A + B), 2.17 – 2.05 (1H, m, C**2**-H', A + B), 1.66 (2H, s, C**6**-H_3, A), 1.58 (1H, s, C**6**-H_3, B); ^{13}C NMR (125 MHz, $CDCl_3$) δ 154.6 (Cbz C=O, B), 153.8 (Cbz C=O, A), 152.5 (C**12**, A), 151.8 (C**12**, B), 148.6 (C**8**, B), 148.5 (C**8**, A), 145.7 (C**10**, B), 145.6 (C**10**, A), 139.7 (C**7**, A), 139.5 (C**7**, B), 137.0 (Cbz ArC, A), 136.8 (Cbz ArC, B), 128.7, 128.6, 128.2, 128.0, 127.8 (Cbz ArCH, A + B), 120.6 (C**11**, A), 120.4 (C**11**, B), 71.7 (C**4**, A), 71.0 (C**4**, B), 67.3 (Cbz CH_2, B), 66.5 (Cbz CH_2, A), 56.8 (C**3**, B), 55.4 (C**3**, A), 48.3 (C**1**, B), 47.1 (C**1**, A), 44.9 (C**5**, B), 43.3 (C**5**, A), 28.1 (C**2**, A), 28.0 (C**2**, B), 25.0 (C**6**, B), 23.4 (C**6**, A); *m/z* (ESI^+) HRMS: Calculated for $C_{19}H_{21}N_2O_2$: 309.1597. Found $[M+H]^+$: 309.1593.

*Note - the regioisomer assignment is based upon the retention of the signals corresponding to C**8** and C**10** on **2m** (^{1}H δ 8.47 – 8.46 for C**8** and C**10**, ^{13}C δ 149.7 for C**8** and 148.3 for C**10** in starting material **1m**, translating to ^{1}H δ 8.45 – 8.42 for C**8** and C**10**, ^{13}C δ 148.5 for C**8** and 145.6 for C**10** in product **2m**.) This is further backed up by the disappearance of a ^{1}H signal at C**12**.*

1-Tosyl-1*H*-indole-3-carbaldehyde

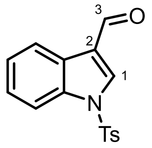

To a solution of 1*H*-indole-3-carboxaldehyde (1.45g, 10.0 mmol) in CH_2Cl_2 (20 mL) at 0 °C was added triethylamine (2.79 mL, 20 mmol) *via* syringe. After stirring for 15 minutes, *p*-toluenesulfonyl chloride (2.10 g, 11.0 mmol) was added, and the reaction was stirred at room temperature for 16 hours. Upon completion, the reaction was diluted with CH_2Cl_2 (20 mL) and washed with sat. NH_4Cl solution (50 mL), sat. $NaHCO_3$ solution (50 mL), brine (50 mL), dried (Na_2SO_4) and concentrated *in vacuo* to give the title compound (3.00 g, 100%) as a purple solid. m.p.: 137 °C (EtOAc/hexane) [lit: 138 – 140 °C][30]; ^{1}H NMR (400 MHz, $CDCl_3$) δ 10.1 (1H, s, C**3**-H), 8.27 – 8.24 (1H, m, ArCH), 8.23 (1H, s, C**1**-H), 7.96 – 7.93 (1H, m, ArCH), 7.87 – 7.83 (2H, m, Ts ArCH), 7.43 – 7.38 (1H, m, ArCH), 7.36 (1H, ddd, *J* = 7.5, 7.5, 1.0 Hz, ArCH), 7.31 – 7.28 (2H, m, Ts ArCH), 2.38 (3H, s, Ts CH_3); ^{13}C NMR (101 MHz, $CDCl_3$) δ 185.4 (C**3**), 146.3 (Ts ArC), 136.3 (C**1**), 135.4 (ArC), 134.5 (Ts ArC), 130.5 (Ts ArCH), 127.4 (Ts ArCH), 126.4 (ArCH + ArC), 125.2 (ArCH), 122.7 (ArCH), 122.5 (C**2**), 113.4 (ArCH), 21.8 (Ts CH_3).

The spectroscopic properties were consistent with the data available in the literature.[30]

Ethyl (*E*)-2-methyl-3-(1-tosyl-1*H*-indol-3-yl)acrylate

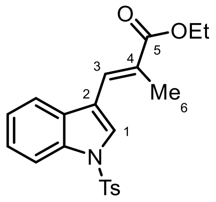

A solution of the preceding aldehyde (3.00 g, 10.0 mmol) and triphenyl(1-(ethoxycarbonyl)ethylidene)phosphorane (5.44 g, 15.0 mmol) in anhydrous toluene (80 mL) was heated to reflux (120 °C) for 16 hours. Upon completion, the reaction was quenched with sat. NH_4Cl solution (100 mL) and the aqueous layer was extracted with EtOAc (3 × 50 mL). The organic phase was washed with brine (100 mL), dried (Na_2SO_4), and concentrated *in vacuo*. FCC (15 – 20% EtOAc/hexane) gave the title compound (3.00 g, 78%) as an off-white solid. m.p.: 136 – 137 °C (EtOAc/hexane); R_f: 0.43 (15% EtOAc/hexane); ν_{max} / cm^{-1}: 2981 (m), 1702 (s), 1373 (s), 1242 (s), 1220 (s), 1174 (s); ^{1}H NMR (400 MHz, $CDCl_3$) δ 8.01 – 7.98 (1H, m, ArC$\underline{H}$), 7.81 – 7.76 (3H, m, Ts ArC$\underline{H}$ + C**3**-$\underline{H}$), 7.74 (1H, s, C**1**-$\underline{H}$), 7.68 – 7.64 (1H, m, ArC$\underline{H}$), 7.39 – 7.35 (1H, m, ArC$\underline{H}$), 7.32 – 7.28 (1H, m, ArC$\underline{H}$), 7.25 – 7.22 (2H, m, Ts ArC$\underline{H}$), 4.30 (2H, q, *J* = 7.0 Hz, OC$\underline{H_2}$CH$_3$), 2.35 (3H, s, Ts C$\underline{H_3}$), 2.20 (3H, d, *J* = 1.5 Hz, C**6**-$\underline{H_3}$), 1.36 (3H, t, *J* = 7.0 Hz, OCH$_2$C$\underline{H_3}$); ^{13}C NMR (101 MHz, $CDCl_3$) δ 168.3 (C**5**), 145.5 (Ts Ar$\underline{C}$), 135.1 (Ts Ar$\underline{C}$), 134.6 (Ar$\underline{C}$), 130.6 (Ar$\underline{C}$), 130.2 (Ts Ar$\underline{C}$H), 129.0 (C**4**), 127.7 (C**3**), 127.0 (Ts Ar$\underline{C}$H), 126.0 (C**1**), 125.5 (Ar$\underline{C}$H), 123.8 (Ar$\underline{C}$H), 119.7 (Ar$\underline{C}$H), 118.2 (C**2**), 113.7 (Ar$\underline{C}$H), 61.1 (O$\underline{C}$H$_2$CH$_3$), 21.7 (Ts $\underline{C}$H$_3$), 15.4 (C**6**), 14.5 (OCH$_2$$\underline{C}H_3$); *m/z* (ESI$^+$) HRMS: Calculated for $C_{21}H_{22}NO_4S$: 384.1264. Found $[M+H]^+$ 384.1279.

(*E*)-2-Methyl-3-(1-tosyl-1*H*-indol-3-yl)prop-2-en-1-ol

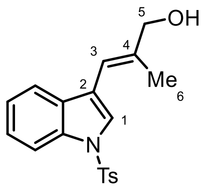

General procedure **F** was followed using preceding ester (2.81 g, 7.33 mmol), $LiAlH_4$ (1M in THF, 14.7 mL) and anhydrous Et_2O at 0 °C for 1 hour. The title compound (2.46 g, 98%) was obtained as colorless oil without purification. ν_{max} / cm^{-1}: 3387 (br s), 2921 (m), 1446 (s), 1366 (s), 1170 (s), 1122 (s); ^{1}H NMR (400 MHz, $CDCl_3$) δ 8.01 – 7.97 (1H, m, ArC$\underline{H}$), 7.77 – 7.73 (2H, m, Ts ArC$\underline{H}$), 7.55 – 7.51 (2H, m, ArC$\underline{H}$ + C**1**-$\underline{H}$), 7.35 – 7.30 (1H, m, ArC$\underline{H}$), 7.26 7.21 (1H, m, ArC$\underline{H}$), 7.20 – 7.17 (2H, m, Ts ArC$\underline{H}$), 6.52 (1H, m, C**3**-$\underline{H}$), 4.26 – 4.24 (2H, m, C**5**-$\underline{H_2}$), 2.31 (3H, s, Ts C$\underline{H_3}$), 1.94 (3H, d, *J* = 1.5 Hz, C**6**-$\underline{H_3}$); ^{13}C NMR (101 MHz, $CDCl_3$) δ 145.1 (Ts Ar$\underline{C}$), 139.6 (C**4**), 135.2 (Ts Ar$\underline{C}$), 134.7 (Ar$\underline{C}$), 131.0 (Ar$\underline{C}$), 130.0 (Ts Ar$\underline{C}$H), 126.9 (Ts Ar$\underline{C}$H), 125.0 (Ar$\underline{C}$H), 123.5 (C**1**), 123.4 (Ar$\underline{C}$H), 119.7 (Ar$\underline{C}$H), 119.4

(C**2**), 113.8 (C**3**), 113.7 (ArCH), 68.7 (C**5**), 21.6 (Ts CH_3), 16.6 (C**6**); *m/z* ($APCI^+$) HRMS: Calculated for $C_{19}H_{18}NO_2S$: 324.1053. Found $[M+H-H_2O]^+$ 324.1041.

3-(1*H*-Indol-3-yl)-4-methylpent-4-enoic acid

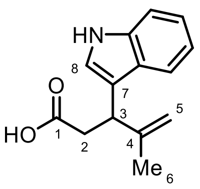

An adapted version of general procedure **E** was followed using the preceding alcohol (2.15 g, 6.30 mmol), triethyl orthoacetate (5.77 mL, 31.5 mmol), *N*,*N*-diisopropylethylamine (5.39 mL, 31.5 mmol) and 4Å molecular sieves (2.15 g) for 2 cycles (16 hours each). The subsequent hydrolysis was stirred at 60 °C for 16 hours. The title compound (201 mg, 14%) was obtained as an orange oil. ν_{max} / cm^{-1}: 3414 (br s), 3057 (s), 2971 (s), 2671 (br s), 1705 (s), 743 (s); ^1H NMR (400 MHz, $CDCl_3$) δ 7.98 (1H, br s, NH), 7.67 – 7.63 (1H, m, ArCH), 7.36 – 7.33 (1H, m, ArCH), 7.21 – 7.16 (1H, m, ArCH), 7.12 – 7.07 (1H, m, ArCH), 7.02 (1H, d, *J* = 2.0 Hz, C**8**-H), 5.04 (1H, m, C**5**-H), 4.92 (1H, m, C**5**-H’), 4.15 (1H, dd, *J* = 8.0, 8.0 Hz, C**3**-H), 2.93 (2H, d, *J* = 8.0 Hz, C**2**-H_2), 1.67 (3H, br s, C**6**-H_3); ^{13}C NMR (101 MHz, $CDCl_3$) δ 177.5 (C**1**), 146.4 (C**4**), 136.7 (ArC), 126.9 (ArC), 122.3 (ArCH), 121.6 (C**8**), 119.6 (ArCH), 119.5 (ArCH), 116.8 (C**7**), 111.3 (ArCH), 111.2 (C**5**), 40.5 (C**3**), 38.6 (C**2**), 20.9 (C**6**); *m/z* (ESI^-) HRMS: Calculated for $C_{14}H_{14}NO_2$: 228.1025. Found $[M-H]^-$ 228.1017.

3-(1*H*-Indol-3-yl)-4-methylpent-4-en-1-ol

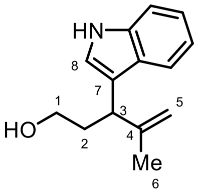

General procedure **F** was followed using the preceding carboxylic acid (200 mg, 0.87 mmol), $LiAlH_4$ (1M in THF, 1.74 mL) and anhydrous Et_2O at 0 °C for 2 hours. FCC (30 – 40% EtOAc/hexane) gave the title compound (71 mg, 38%) as a colorless oil. R_f: 0.45 (40% EtOAc/hexane); ν_{max} / cm^{-1}: 3411 (br s), 2932 (s), 1643 (m), 1456 (s), 740 (s); ^1H NMR (400 MHz, $CDCl_3$) δ 8.01 (1H, br s, NH), 7.69 – 7.65 (1H, m, ArCH), 7.37 – 7.34 (1H, m, ArCH), 7.18 (1H, ddd, *J* = 8.0, 7.0, 1.0 Hz, ArCH), 7.09 (1H, ddd, *J* = 8.0, 7.0, 1.0 Hz, ArCH), 7.02 (1H, d, *J* = 2.5 Hz, C**8**-H), 5.07 (1H, m, C**5**-H), 4.90 (1H, m, C**5**-H’), 3.80 (1H, dd, *J* = 7.5, 7.5 Hz, C**3**-H), 3.72 (2H, br t, *J* = 6.5 Hz, C**1**-H_2), 2.19 – 2.13 (2H, m, C**2**-H_2), 1.62 (3H, br s, C**6**-H_3), 1.42 (1H, br s, OH); ^{13}C NMR (101 MHz, $CDCl_3$) δ 147.8 (C**4**), 136.7 (ArC), 127.2 (ArC), 122.2 (ArCH), 121.3 (C**8**), 119.7 (ArCH), 119.4 (ArCH), 117.9 (C**7**), 111.2 (ArCH), 111.1 (C**5**), 61.8 (C**1**), 41.2 (C**3**), 35.4 (C**2**), 20.0 (C**6**); *m/z* (ESI^+) HRMS: Calculated for $C_{14}H_{18}NO$: 216.1383. Found $[M+H]^+$ 216.1390.

Benzyl (3-(1*H*-indol-3-yl)-4-methylpent-4-en-1-yl)((perfluorobenzoyl)oxy)carbamate (1n)

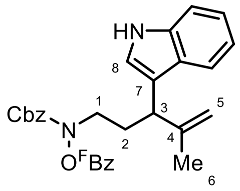

General procedure **I** was followed using the preceding alcohol (92 mg, 0.43 mmol), CbzNHO^{F}Bz (170 mg, 0.47 mmol), triphenylphosphine (123 mg, 0.47 mmol) and diisopropyl azodicarboxylate (93 μL, 0.47 mmol). FCC (10 – 15% EtOAc/hexane) gave title compound **1n** (174 mg, 72%) as a colorless oil. R_f: 0.40 (20% EtOAc/hexane); ν_{max} / cm^{-1}: 3415 (br s), 2945 (m), 1780 (s), 1722 (s), 1497 (s), 1326 (s), 1173 (s); ^{1}H NMR (400 MHz, $CDCl_3$) δ 7.93 (1H, br s, NH), 7.62 – 7.58 (1H, m, ArCH), 7.38 – 7.29 (6H, m, Cbz ArCH + ArCH), 7.16 (1H, ddd, *J* = 8.0, 7.0, 1.0 Hz, ArCH), 7.04 (1H, ddd, *J* = 8.0, 7.0, 1.0 Hz, ArCH), 6.97 (1H, br d, *J* = 2.5 Hz, C**8**-H), 5.19 (2H, s, Cbz CH_2), 5.01 (1H, m, C**5**-H), 4.88 (1H, m, C**5**-H’), 3.83 – 3.78 (2H, m, C**1**-H_2), 3.69 (1H, dd, *J* = 7.5, 7.5 Hz, C**3**-H), 2.27 – 2.21 (2H, m, C**2**-H_2), 1.60 (3H, br s, C**6**-H_3); ^{13}C NMR (101 MHz, $CDCl_3$) δ 155.5 (Cbz C=O), 146.7 (C**4**), 136.7 (ArC), 135.4 (Cbz ArC), 128.7 (Cbz ArCH), 128.6 (Cbz ArCH), 128.3 (Cbz ArCH), 127.0 (ArC), 122.2 (ArCH), 121.5 (C**8**), 119.6 (ArCH), 119.3 (ArCH), 117.0 (C**7**), 111.4 (C**5**), 111.2 (ArCH), 68.9 (Cbz CH_2), 50.3 (C**1**), 41.6 (C**3**), 29.7 (C**2**), 20.1 (C**6**); ^{19}F NMR (377 MHz, $CDCl_3$) δ -135.9 – -136.1 (2F, m), -146.1 (1F, tt, *J* = 21.0, 5.5 Hz), -159.3 – -159.4 (2F, m); *m/z* (ESI^+) HRMS: Calculated for $C_{29}H_{24}F_5N_2O_4$: 559.1651. Found $[M+H]^+$ 559.1650.

The ^{13}C signals corresponding to the pentafluorobenzoyl group could not be resolved due to their weak intensity.

Benzyl 9a-methyl-2,3,3a,8,9,9a-hexahydro-1*H*-pyrrolo[3',2':3,4]cyclopenta[1,2-*b*]indole-1-carboxylate (2n)

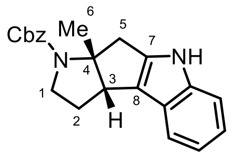

General procedure **J** was followed using cyclization substrate **1n** (55.9 mg, 0.1 mmol), Pd_2dba_3 (4.58 mg, 0.005 mmol), CgPPh (8.77 mg, 0.03 mmol), NaOBz (1.44 mg, 0.01 mmol), triethylamine (27.9 μL) and dibutylether (1 mL). The reaction was stirred at 130 °C for 48 hours. FCC (3% acetone/toluene) gave title compound **2n** (20.1 mg, 58%) as a colorless oil. R_f: 0.20 (3% acetone/toluene); ν_{max} / cm^{-1}: 3320 (br s), 2963 (s), 1669 (s), 1405 (s), 1353 (s), 735 (s); *mixture of rotamers A + B (2:1)* ^{1}H NMR (500 MHz, $CDCl_3$) δ 7.95 (0.66H, br s, NH, A), 7.80 (0.33H, br s, NH, B), 7.46 – 7.27 (7H, m, ArCH, A + B), 7.14 – 7.07 (2H, m, ArCH, A + B), 5.24 – 5.19 (0.66H, m, Cbz CH_2, B), 5.13 (0.66H, d, *J* = 12.5 Hz, Cbz CH, A), 5.09 (0.66H, d, *J* = 12.5 Hz, Cbz CH’, A), 3.75 – 3.68 (1H, m, C**1**-H, A + B),

3.64 (0.66H, d, J = 17.0 Hz, C**5**-H, A), 3.61 – 3.58 (1H, m, C**3**-H, A + B), 3.47 – 3.42 (0.33H, m, C**1**-H', B), 3.40 (0.33H, d, J = 17.0 Hz, C**5**-H, B), 3.30 (0.66H, ddd, J = 11.0, 9.5, 7.0 Hz, C**1**-H', A), 3.00 (0.66H, d, J = 17.0 Hz, C**5**-H', A), 2.95 (0.33H, d, J = 17.0 Hz, C**5**-H', B), 2.23 – 2.14 (1.66H, m, C**2**-H, A + B, C**2**-H', A), 2.09 – 2.04 (0.33H, m, C**2**-H', B), 1.73 (2H, s, C**6**-H_3, A), 1.66 (1H, s, C**6**-H_3, B); ^{13}C NMR (126 MHz, $CDCl_3$) δ 154.7 (Cbz C=O, B), 154.0 (Cbz C=O, A), 141.4 (ArC, A), 141.0 (C**7**, A + B), 140.4 (ArC, B), 137.2 (Cbz ArC, A), 137.0 (Cbz ArC, B), 128.7, 128.6, 128.2, 128.1, 127.9, 127.8 (Cbz ArCH + ArC, A + B), 124.2 (C**8**, A), 124.2 (C**8**, B), 121.1 (ArCH, B), 120.9 (ArCH, A), 119.9 (ArCH, B), 119.8 (ArCH, A), 118.1 (ArCH, B), 118.0 (ArCH, A), 111.7 (ArCH, A), 111.7 (ArCH, B), 77.3 (C**4**, A), 76.3 (C**4**, B), 67.1 (Cbz CH_2, B), 66.3, (Cbz CH_2, A) 53.3 (C**3**, B), 51.8 (C**3**, A), 48.8 (C**1**, B), 47.6 (C**1**, A), 40.2 (C**5**, B), 38.5 (C**5**, A), 27.9 (C**2**, B), 27.7 (C**2**, A), 26.4 (C**6**, B), 24.7 (C**6**, A); *m/z* (ESI^+) HRMS: Calculated for $C_{22}H_{23}N_2O_2$: 347.1754. Found $[M+H]^+$ 347.1752.

Some signal broadening was observed due to amide-like resonance.

Substrate Synthesis and Catalysis for Table 2

2-Benzylacrylaldehyde

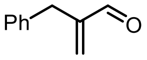

This compound was prepared according to our previously reported literature procedure.[31]

The spectroscopic properties were consistent with the data available in the literature.[31]

2-Benzylprop-2-en-1-ol

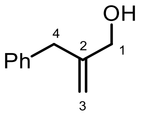

General procedure **C** was followed using 2-benzylacrylaldehyde (4.00 g, 27.4 mmol), $NaBH_4$ (1.09 g, 28.7 mmol). FCC (10% EtOAc/petroleum ether) gave the title compound (3.99 g, 98%) as a colorless oil. ^{1}H NMR (400 MHz, $CDCl_3$) δ 7.34 – 7.18 (5H, m, Ph ArCH), 5.13 (1H, m, C**3**-H), 4.91 (1H, m, C**3**-H'), 4.04 (2H, br s, C**1**-H_2), 3.41 (2H, br s, C**4**-H_2); ^{13}C NMR (101 MHz, $CDCl_3$) δ 148.3 (C**2**), 139.1 (ArC), 129.0 (ArCH), 128.5 (ArCH), 126.4 (ArCH), 111.6 (C**3**), 65.4 (C**1**), 40.0 (C**4**).

The spectroscopic properties are consistent with the data available in the literature.[32]

Ethyl 4-benzylpent-4-enoate

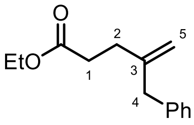

General procedure **D** was followed using the preceding alcohol (3.99 g, 26.9 mmol), propionic acid (0.40 mL, 5.38 mmol) and triethyl orthoacetate (49 mL, 269 mmol) for 16 hours. FCC (2% EtOAc/petroleum ether) gave the title compound (3.57 g, 61%) as a colorless oil. R*f*: 0.20 (2% EtOAc/petroleum ether); ν_{max} / cm^{-1}: 2981 (m), 1732 (s), 1495 (m), 1370 (m), 1236 (s), 1155 (s); ^{1}H NMR (400 MHz, $CDCl_3$) δ 7.32 – 7.25 (2H, m, ArCH), 7.23 – 7.16 (3H, m, ArCH), 4.83 (1H, m, C**5**-H), 4.80 (1H, m, C**5**-H'), 4.11 (2H, q, *J* = 7.0 Hz, OCH_2CH_3), 3.36 (2H, br s, C**4**-H_2), 2.49 – 2.41 (2H, m, C**1**-H_2), 2.30 (2H, m, C**2**-H_2), 1.24 (2H, t, *J* = 7.0 Hz, OCH_2CH_3); ^{13}C NMR (101 MHz, $CDCl_3$) δ 173.3 (C=O), 147.4 (C**3**), 139.4 (ArC), 129.1 (ArCH), 128.5 (ArCH), 126.3 (ArCH), 111.7 (C**5**), 60.5 (OCH_2CH_3), 43.3 (C**4**), 32.7 (C**1**), 30.4 (C**2**), 14.4 (OCH_2CH_3); *m/z* (ESI^+) HRMS: Calculated for $C_{14}H_{18}NaO_5$: 241.1199. Found $[M+Na]^+$: 241.1199.

4-Benzylpent-4-en-1-ol

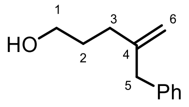

General procedure **F** was followed using the preceding ester (3.57 g, 16.4 mmol), $LiAlH_4$ (1M in THF, 19.6 mL) in anhydrous THF at room temperature for 4 hours. FCC (15% EtOAc/petroleum ether) gave the title compound (2.26 g, 78%) as a colorless oil. R*f*: 0.32 (20% EtOAc/petroleum ether); ν_{max} / cm^{-1}: 3333 (br s), 3083 (m), 3027 (m), 2938 (m), 1055 (s), 893 (s), 733 (s), 698 (s); ^{1}H NMR (400 MHz, $CDCl_3$) δ 7.32 – 7.26 (2H, m, ArCH), 7.24 – 7.17 (3H, m, ArCH), 4.92 (1H, m, C**6**-H), 4.80 (1H, m, C**6**-H'), 3.62 (2H, t, *J* = 6.5 Hz, C**1**-H_2), 3.36 (2H, br s, C**5**-H_2), 2.06 (2H, br t, *J* = 7.5 Hz, C**3**-H_2), 1.77 – 1.67 (2H, m, C**2**-H_2), 1.39 (1H, br s, OH); ^{13}C NMR (101 MHz, $CDCl_3$) δ 148.6 (C**4**), 139.7 (ArC), 129.1 (ArCH), 128.4 (ArCH), 126.3 (ArCH), 111.6 (C**4**), 62.8 (C**1**), 43.2 (C**5**), 31.7 (C**3**), 30.7 (C**2**); *m/z* (ESI^+) HRMS: Calculated for $C_{12}H_{16}NaO$: 199.1093. Found $[M+Na]^+$: 199.1097.

Benzyl (4-benzylpent-4-en-1-yl)((perfluorobenzoyl)oxy)carbamate (3a)

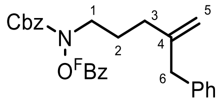

General procedure **I** was followed using $CbzNHO^{F}Bz$ (2.25 g, 6.24 mmol), the preceding alcohol (1.00 g, 5.67 mmol), triphenylphosphine (1.64 g, 6.24 mmol) and diisopropyl azodicarboxylate (1.23 mL, 6.24 mmol). FCC (3% EtOAc/hexane) gave title compound **3a** (2.15 g, 73%) as a colorless oil. R*f*: 0.17 (3% EtOAc/hexane); ν_{max} / cm^{-1}: 2946 (m), 1785 (s), 1730 (s), 1524 (s), 1499 (s), 1172 (s); ^{1}H NMR (400 MHz, $CDCl_3$) δ 7.39 – 7.30 (5H, m, Cbz ArCH), 7.29 – 7.24 (2H, m, Ph ArCH), 7.21 – 7.13 (3H, m, Ph ArCH), 5.21 (2H, s, Cbz CH_2), 4.84 (1H, m, C**5**-H), 4.79 (1H, m, C**5**-H'), 3.72 (2H, dd, *J* = 7.0, 7.0 Hz, C**1**-H_2), 3.32 (2H, s, C**6**-H_2), 2.05 (2H, br t, *J* = 7.5 Hz, C**3**-H_2), 1.84 – 1.74 (2H, m, C**2**-H_2); ^{13}C NMR (101 MHz, $CDCl_3$) δ 155.5 (Cbz C=O), 147.5 (C**4**), 139.5 (Ph ArC), 135.3 (Cbz ArC), 129.1 (Ph ArCH), 128.7 (Cbz ArCH), 128.6 (Cbz ArCH), 128.5 (Ph ArCH), 128.3 (Cbz ArCH), 126.3 (Ph ArCH),

112.1 (C**5**), 68.9 (Cbz C̲H₂), 51.0 (C**1**), 43.1 (C**6**), 32.2 (C**3**), 24.9 (C**2**); ^{19}F NMR (377 MHz, $CDCl_3$) δ -135.9 – -136.1 (2F, m), -146.0 (1F, tt, *J* = 21.0, 5.5 Hz), -159.2 – -159.4 (2F, m); *m/z* (ESI^+) HRMS: Calculated for $C_{27}H_{22}F_5NNaO_4$: 542.1361. Found $[M+Na]^+$: 542.1354.

The carbon signals corresponding to the pentafluorobenzoyl group could not be resolved due to their weak intensity.

Benzyl 1,3-dihydrospiro[indene-2,2'-pyrrolidine]-1'-carboxylate (4a)

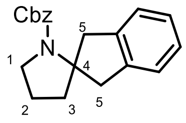

General procedure **J** was followed using cyclization substrate **3a** (52.0 mg, 0.10 mmol), $Pd_2(dba)_3$ (2.29 mg, 0.0025 mmol), CgPPh (4.38 mg, 0.015 mmol), sodium benzoate (1.44 mg, 0.01 mmol), triethylamine (1.40 μL) and dibutyl ether (1 mL). The reaction was stirred at 130 °C for 48 hours. FCC (8% EtOAc/hexane) gave title compound **4a** (23.7 mg, 77%) as a colorless oil. R_f: 0.25 (8% EtOAc/hexane); ν_{max} / cm^{-1}: 2965 (s), 1693 (s), 1404 (s), 1355 (s); *mixture of rotamers A + B (2:1)* ^{1}H NMR (500 MHz, $CDCl_3$) δ 7.42 – 7.28 (3H, m, Cbz ArCH̲), 7.23 – 7.01 (6H, m, 2 × Cbz ArCH̲ + 4 × ArCH̲), 5.11 (1.3H, br s, Cbz CH̲₂, A), 5.08 (0.7H, br s, Cbz CH̲₂, B), 3.93 (1.3H, d, *J* = 15.5 Hz, C**5**-H̲, A), 3.68 (0.7H, d, *J* = 15.5 Hz, C**5**-H̲, B), 3.63 – 3.56 (2H, m, C**1**-H̲₂, A + B), 2.82 (0.7H, d, *J* = 15.5 Hz, C**5**-H̲', B), 2.67 (1.3H, d, *J* = 15.5 Hz, C**5**-H̲', A), 2.04 – 2.00 (0.7H, m, C**3**-H̲₂, B), 1.97 – 1.93 (1.3H, m, C**3**-H̲₂, A), 1.88 – 1.82 (2H, m, C**2**-H̲₂, A + B); ^{13}C NMR (126 MHz $CDCl_3$) δ 154.9 (C̲=O, B), 153.9 (C̲=O, A), 141.7 (2 × ArC̲, A), 141.5 (2 × ArC̲, B), 137.3 (Cbz ArC̲, A), 136.7 (Cbz ArC̲, B), 128.6, 128.6, 128.4, 128.3, 128.0, 128.0, 127.7, 127.7, 126.5, 126.3, 124.5, 124.5 (ArC̲H, A + B), 70.3 (C**4**, A), 69.1 (C**4**, B), 66.8 (Cbz C̲H₂, B), 66.3 (Cbz C̲H₂, A), 49.0 (C**1**, B), 48.2 (C**1**, A), 45.3 (C**5**, B), 43.9 (C**3**, B), 43.0 (C**5**, A), 42.0 (C**3**, A), 22.8 (C**2**, A), 22.6 (C**2**, B); *m/z* (ESI^+) HRMS: Calculated for $C_{20}H_{22}NO_2$: 308.1645. Found $[M+H]^+$: 308.1648.

Some signal broadening was observed due to amide-like resonance.

3-(4-Methoxyphenyl)propanal

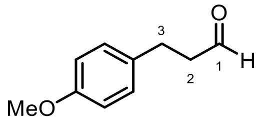

A round bottom flask was charged with $NaHCO_3$ (9.20 g, 125.6 mmol), tetrabutylammonium chloride (15.58 g, 62.8 mmol) and $Pd(OAc)_2$ (122 mg, 0.628 mmol) under Ar. A solution of 4-iodoanisole (12.98 g, 62.8 mmol) in DMF (54 mL) and allyl alcohol (5.6 mL, 94.2 mmol) were added sequentially *via* syringe, and the mixture was stirred at 50 ºC for 16 hours. Upon completion, the reaction was filtered through celite, washing with EtOAc. The filtrate was washed with water (3 × 100 mL), brine (100 mL),

dried (Na_2SO_4) and concentrated *in vacuo*. FCC (5 – 8% EtOAc/hexane) gave the title compound (7.01 g, 68%) as a colorless oil. ^{1}H NMR (400 MHz, $CDCl_3$) δ 9.81 (1H, br t, *J* = 1.0 Hz, C**1**-H), 7.11 (2H, d, *J* = 8.5 Hz, ArCH), 6.84 (2H, d, *J* = 8.5 Hz, ArCH), 3.79 (3H, s, OCH_3), 2.91 (2H, t, *J* = 7.5 Hz, C**3**-H_2), 2.75 (2H, m, C**2**-H_2); *m/z* (ESI^+) HRMS: Calculated for $C_{10}H_{12}NaO_2$: 187.0730. Found $[M+Na]^+$: 187.0737.

The spectroscopic properties were consistent with the data available in the literature.[33]

2-(4-Methoxybenzyl)acrylaldehyde

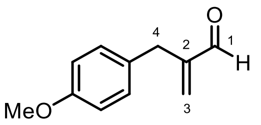

General procedure **B** was followed using of 4-(dimethylamino)benzoic acid (0.60 g, 3.59 mmol), pyrrolidine (0.3 mL, 3.59 mmol), formaldehyde (2.7 mL, 35.9 mmol) and the preceding aldehyde (5.90 g, 35.9 mmol). FCC (0 – 10% EtOAc/hexane) gave the title compound (5.14 g, 81%) as a colorless oil. ^{1}H NMR (400 MHz, $CDCl_3$) δ 9.60 (1H, s, C**1**-H), 7.10 (2H, d, *J* = 8.5 Hz, ArCH), 6.84 (2H, d, *J* = 8.5 Hz, ArCH), 6.09 (1H, m, C**3**-H), 6.04 (1H, m, C**3**-H'), 3.79 (3H, s, OCH_3), 3.51 (2H, s, C**4**-H_2); *m/z* (ESI^+) HRMS: Calculated for $C_{11}H_{12}NaO_2$: 199.0730. Found $[M+Na]^+$: 199.0728.

The spectroscopic properties were consistent with the data available in the literature.[34]

2-(4-Methoxybenzyl)prop-2-en-1-ol

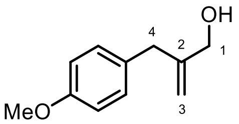

General procedure **C** was followed using the preceding aldehyde (1.77 g, 10.05 mmol) and $NaBH_4$ (0.41 g, 10.55 mmol). FCC (20% EtOAc/petroleum ether) gave the title compound (1.63 g, 91%) as a colorless oil. ^{1}H NMR (400 MHz, $CDCl_3$) δ 7.12 (2H, d, *J* = 8.5 Hz, ArCH), 6.84 (2H, d, *J* = 8.5 Hz, ArCH), 5.10 (1H, m, C**3**-H), 4.90 (1H, m, C**3**-H'), 4.04 (2H, s, C**1**-H_2), 3.79 (3H, s, OCH_3), 3.35 (2H, s, C**4**-H_2); *m/z* (ESI^+) HRMS: Calculated for $C_{11}H_{14}NaO_2$: 201.0886. Found $[M+Na]^+$: 201.0881.

The spectroscopic properties were consistent with the data available in the literature.[35]

Ethyl 4-(4-methoxybenzyl)pent-4-enoate

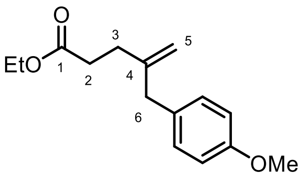

General procedure **D** was followed using the preceding alcohol 1.63 g, 9.145 mmol), triethyl orthoacetate (17.1 mL, 94.4 mmol) and propionic acid (0.14 mL, 1.829 mmol). The title compound (2.38 g, quantitative) was obtained as a colorless oil and used in the next step without further purification. R$_f$: 0.48 (10% EtOAc/hexane); ν_{max} / cm^{-1}: 1732 (s), 1510 (s), 1245 (s); ^{1}H NMR (400 MHz, $CDCl_3$) δ 7.09 (2H, d, *J* = 8.5 Hz, ArC<u>H</u>), 6.83 (2H, d, *J* = 8.5 Hz, ArC<u>H</u>), 4.80 (1H, m, C**5**-<u>H</u>), 4.75 (1H, m, C**5**-<u>H</u>'), 4.11 (2H, q, *J* = 7.0 Hz, OC<u>H$_2$</u>CH$_3$), 3.79 (3H, s, OC<u>H$_3$</u>), 3.30 (2H, s, C**6**-<u>H$_2$</u>), 2.45 – 2.42 (2H, m, C**2**-<u>H$_2$</u>), 2.30 – 2.27 (2H, m, C**3**-<u>H$_2$</u>), 1.24 (3H, t, *J* = 7.0 Hz, OCH$_2$C<u>H$_3$</u>); ^{13}C NMR (101 MHz, $CDCl_3$) δ 173.4 (C**1**), 158.2 (Ar<u>C</u>), 147.8 (C**4**), 131.5 (Ar<u>C</u>), 130.0 (Ar<u>C</u>H), 113.9 (Ar<u>C</u>H), 111.4 (C**5**), 60.5 (O<u>C</u>H$_2$CH$_3$), 55.4 (O<u>C</u>H$_3$), 42.5 (C**6**), 32.8 (C**2**), 30.4 (C**3**), 14.4 (OCH$_2$<u>C</u>H$_3$); *m/z* (ESI$^+$) HRMS: Calculated for $C_{15}H_{20}NaO_3$: 271.1305. Found [M+Na]$^+$: 271.1313.

4-(4-Methoxybenzyl)pent-4-en-1-ol

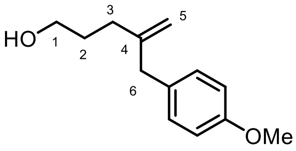

General procedure **F** was followed using the preceding ester (6.52 g, 26.3 mmol), $LiAlH_4$ (1.50 g, 39.4 mmol, 1.5 eq.) and anhydrous THF at 0 °C for 50 minutes. FCC (5 – 20% EtOAc/hexane) gave the title compound (3.23 g, 60%) as a colorless oil. R$_f$: 0.30 (20% EtOAc/hexane); ν_{max} / cm^{-1}: 3308 (br s), 2935 (m), 1510 (s), 1246 (s); ^{1}H NMR (400 MHz, $CDCl_3$) δ 7.10 (2H, d, *J* = 8.5 Hz, ArC<u>H</u>), 6.83 (2H, d, *J* = 8.5 Hz, ArC<u>H</u>), 4.84 (1H, m, C**5**-<u>H</u>), 4.76 (1H, m, C**5**-<u>H</u>'), 3.79 (3H, s, OC<u>H$_3$</u>), 3.64 – 3.61 (2H, m, C**1**-<u>H$_2$</u>), 3.30 (2H, s, C**6**-<u>H$_2$</u>), 2.04 (2H, m, C**3**-<u>H$_2$</u>), 1.74 – 1.67 (2H, m, C**2**-<u>H$_2$</u>); ^{13}C NMR (101 MHz, $CDCl_3$) δ 158.1 (Ar<u>C</u>), 149.0 (C**4**), 131.8 (Ar<u>C</u>), 130.0 (Ar<u>C</u>H), 113.9 (Ar<u>C</u>H), 111.3 (C**5**), 62.8 (C**1**), 55.4 (O<u>C</u>H$_3$), 42.3 (C**6**), 31.6 (C**3**), 30.7 (C**2**); *m/z* (ESI$^+$) HRMS: Calculated for $C_{13}H_{19}O_2$: 207.1380. Found [M+H]$^+$: 207.1383.

Benzyl (4-(4-methoxybenzyl)pent-4-en-1-yl)((perfluorobenzoyl)oxy)carbamate (3b)

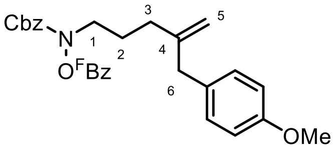

General procedure **I** was followed using CbzNHO^{F}Bz (1.22 g, 3.388 mmol), the preceding alcohol (0.70 g, 3.388 mmol), triphenylphosphine (0.99 g, 3.727 mmol) and diisopropyl azodicarboxylate (0.78 mL, 3.727 mmol). FCC (3 – 6% EtOAc/hexane) gave title compound **3b** (2.86 g, 72%) as a colorless oil. R$_f$: 0.31 (10% EtOAc/hexane); ν_{max} / cm^{-1}: 1785 (m), 1730 (m), 1524 (m), 1509 (s), 1326 (m), 1247 (m), 1175 (s); ^{1}H NMR (400 MHz, $CDCl_3$) δ 7.38 – 7.31 (5H, m, Cbz ArC<u>H</u>), 7.06 (2H, d, *J* = 8.0 Hz, Ph ArC<u>H</u>), 6.80 (2H, d, *J* = 8.0 Hz, Ph ArC<u>H</u>), 5.21 (2H, s, Cbz C<u>H$_2$</u>), 4.81 (1H, m, C**5**-<u>H</u>), 4.77 (1H, m,

C**5**-H'), 3.78 (3H, s, OCH3), 3.71 (2H, t, *J* = 7.5 Hz, C**1**-H2), 3.25 (2H, s, C**6**-H2), 2.03 (2H, t, *J* = 7.5 Hz, C**3**-H2), 1.78 (2H, tt, *J* = 7.5, 7.5 Hz, C**2**-H2); ^{13}C NMR (101 MHz, $CDCl_3$) δ 158.2 (Ph ArC), 155.5 (Cbz C=O), 147.9 (C**4**), 135.3 (Cbz ArC), 131.6 (Ph ArC), 130.0 (Ph ArCH), 128.7 (Cbz ArCH), 128.6 (Cbz ArCH), 128.3 (Cbz ArCH), 113.9 (Ph ArCH), 111.8 (C**5**), 68.9 (Cbz CH_2), 55.4 (OCH_3), 51.0 (C**1**), 42.2 (C**6**), 32.1 (C**3**), 24.9 (C**2**); ^{19}F NMR (400 MHz, $CDCl_3$) δ -136.0 – -136.1 (2F, m), -146.0 (1F, tt, *J* = 20.9, 5.3 Hz), -159.2 – -159.4 (2F, m); *m/z* (ESI$^+$) HRMS: Calculated for $C_{28}H_{24}F_5NNaO_5$: 572.1467. Found $[M+Na]^+$: 572.1444.

The ^{13}C NMR signals corresponding to the pentafluorobenzoyl group could not be resolved due to their weak intensity.

Benzyl 5-methoxy-1,3-dihydrospiro[indene-2,2'-pyrrolidine]-1'-carboxylate (4b)

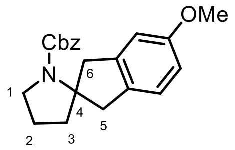

General procedure **J** was followed using cyclization substrate **3b** (54.8 mg, 0.10 mmol), $Pd_2(dba)_3$ (2.30 mg, 0.0025 mmol), CgPPh (4.5 mg, 0.015 mmol), sodium benzoate (1.44 mg, 0.01 mmol), triethylamine (1.40 μL) and dibutyl ether (1 mL). The reaction was stirred at 130 °C for 48 hours. FCC (0 – 6% EtOAc/hexane) gave title compound **4b** (27 mg, 76%) as a colorless oil. R_f: 0.36 (10% EtOAc/hexane); ν_{max} / cm^{-1}: 1694 (s), 1493 (m), 1404 (s), 1355 (m), 1111 (m); *mixture of rotamers A + B (55:45)* ^{1}H NMR (500 MHz, $CDCl_3$) δ 7.39 – 7.30 (3.1H, m, Cbz ArCH, A + B), 7.21 – 7.17 (1H, m, Cbz ArCH, A + B), 7.07 – 7.01 (1.9H, m, Ph ArCH, A + B, Cbz ArCH, B), 6.78 – 6.67 (2H, m, Ph ArCH, A + B), 5.18 – 5.15 (0.45H, m, Cbz CH2, B), 5.11 – 5.09 (1.55H, m, Cbz CH2, A + B), 3.92 (0.55H, d, *J* = 15.0 Hz, C**5**-H, A), 3.81 (0.55H, d, *J* = 14.5 Hz, C**6**-H, A), 3.78 (1.65H, s, OCH3, A), 3.77 (1.35H, s, OCH3, B), 3.66 (0.45H, d, *J* = 15.5 Hz, C**6**-H, B), 3.61 – 3.56 (2.45H, m, C**1**-H2, A + B, C**5**-H, B), 2.78 (0.45H, d, *J* = 15.5 Hz, C**6**-H', B), 2.75 (0.45H, d, *J* = 14.6 Hz, C**5**-H', B), 2.64 (0.55H, d, *J* = 15.0 Hz, C**5**-H', A), 2.61 (0.55H, d, *J* = 14.5 Hz, C**6**-H', A), 2.04 – 2.00 (0.9H, m, C**3**-H2, B), 1.96 – 1.93 (1.1H, m, C**3**-H2, A), 1.87 – 1.82 (2H, m, C**2**-H2, A + B); ^{13}C NMR (125 MHz, $CDCl_3$) δ 158.8 (Ph ArC, B), 158.6 (Ph ArC, A), 154.9 (Cbz C=O, B), 153.8 (Cbz C=O, A), 143.0 (Ph ArC, A), 142.8 (Ph ArC, B), 137.3 (Cbz ArC, A), 136.7 (Cbz ArC, B), 133.7 (Ph ArC, A), 133.4 (Ph ArC, B), 130.3 (Ph ArCH, B), 128.6, 128.5, 128.4, 128.0, 127.9, 127.7 (Cbz ArCH, A + B), 125.03 (Ph ArCH, A), 112.6 (Ph ArCH, B), 112.2 (Ph ArCH, A), 110.0 (Ph ArCH, A), 109.7 (Ph ArCH, B), 70.6 (C**4**, A), 69.5 (C**4**, B), 66.8 (Cbz CH_2, B), 66.3 (Cbz CH_2, A), 55.5 (OCH_3, A), 55.3 (OCH_3, B), 49.0 (C**1**, B), 48.2 (C**1**, A), 45.6 (C**6**, B), 44.6 (C**5**, B), 43.9 (C**3**, B), 43.4 (C**5**, A), 42.3 (C**6**, A), 42.1 (C**3**, A), 22.8 (C**2**, A), 22.6 (C**2**, B); *m/z* (ESI$^+$) HRMS: Calculated for $C_{21}H_{24}NO_3$: 338.1751. Found $[M+H]^+$: 338.1760.

Some signal broadening was observed due to amide-like resonance.

5-(4-Nitrobenzylidene)-2,2-dimethyl-[1,3]dioxane-4,6-dione

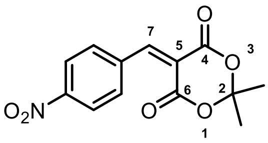

4-Nitrobenzaldehyde (2.39 g, 15.82 mmol) was added to a suspension of Meldrum's acid (2.11 g, 14.38 mmol) in water (26 mL). The mixture was stirred at 75 °C for 2 hours. Upon completion, the resulting solid was filtered, washing with water (50 mL) and petroleum ether (50 mL), before recrystallisation from ethanol to give the title compound (3.23 g, 81%) as a colorless solid. ν_{max} / cm^{-1}: 1760 (m), 1728 (s), 1527 (m), 1306 (m), 1291 (s); ^{1}H NMR (400 MHz, $CDCl_3$) δ 8.46 (1H, s, C**7**-H), 8.30 (2H, d, *J* = 8.5 Hz, ArCH), 8.06 (2H, d, *J* = 8.5 Hz, ArCH), 1.84 (6H, s, 2 × CH_3).

The spectroscopic properties were consistent with the data available in the literature.[36]

5-(4-Nitrobenzylidene)-2,2-dimethyl-[1,3]dioxane-4,6-dione

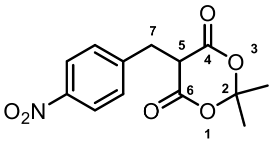

To a solution of the preceding dione (3.23 g, 11.65 mmol) in acetic acid (13 mL) and CH_2Cl_2 (80 mL) at 0 °C, was added $NaBH_4$ (1.44 g, 37.28 mmol) portionwise. The reaction was stirred at room temperature for 1 hour. Upon completion, the reaction was quenched with water (50 mL), and the aqueous phase was extracted with CH_2Cl_2 (3 × 50 mL). The combined organic layers were washed with brine (100 mL), dried (Na_2SO_4) and concentrated *in vacuo*. The title compound (3.20 g, quantitative) was obtained as a colorless solid and used in the next step without further purification. ν_{max} / cm^{-1}: 1779 (m), 1736 (s), 1518 (s), 1337 (s); ^{1}H NMR (400 MHz, $CDCl_3$) δ 8.15 (2H, d, *J* = 9.0 Hz, ArCH), 7.53 (2H, d, *J* = 9.0 Hz, ArCH), 3.81 (1H, t, *J* = 5.0 Hz, C**5**-H), 3.58 (2H, d, *J* = 5.0 Hz, C**7**-H_2), 1.79 (3H, s, CH_3), 1.67 (3H, s, CH_3).

The spectroscopic properties were consistent with the data available in the literature.[36]

3-(4-Nitrophenyl)-propanal

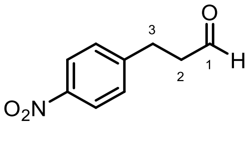

To a solution of the preceding dione (3.05 g, 10.92 mmol) in THF (65 mL), was added triethylamine (3.0 mL, 21.84 mmol) followed by phenylsilane (4.2 mL, 32.76 mmol) *via* syringe. The reaction was stirred at room temperature for 2 hours. Upon completion, water (11 mL) was added, and the mixture

was stirred for 15 minutes before further dilution with Et_2O (20 mL). The organic phase was washed with water (20 mL), dried (Na_2SO_4) and concentrated *in vacuo*. FCC (10 – 20% EtOAc/hexane) gave the title compound (668 mg, 34%) as a colorless solid. ν_{max} / cm^{-1}: 1723 (m), 1517 (s), 1346 (s), 1133 (s); ^{1}H NMR (400 MHz, $CDCl_3$) δ 9.83 (1H, s, C**1**-H), 8.16 (2H, d, J = 9.0 Hz, ArCH), 7.37 (2H, d, J = 9.0 Hz, ArCH), 3.06 (2H, t, J = 7.0 Hz, C**3**-H_2), 2.86 (2H, t, J = 7.0 Hz, C**2**-H_2).

The spectroscopic properties were consistent with the data available in the literature.[36]

2-(4-Nitrobenzyl)acrylaldehyde

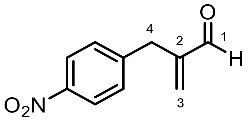

General procedure **B** was followed using 4-(dimethylamino)benzoic acid (62 mg, 0.373 mmol), pyrrolidine (30 μL, 0.373 mmol), formaldehyde (0.28 mL, 3.73 mmol) and the preceding aldehyde (668 mg, 3.73 mmol). FCC (0 – 10% EtOAc/petroleum ether) gave the title compound (390 mg, 55%) as a colorless oil. ^{1}H NMR (400 MHz, $CDCl_3$) δ 9.60 (1H, s, C**1**-H), 8.16 (2H, d, J = 8.5 Hz, ArCH), 7.36 (2H, d, J = 8.5 Hz, ArCH), 6.21 (1H, m, C**3**-H), 6.16 (1H, m, C**3**-H'), 3.68 (2H, s, C**4**-H_2); *m/z* (ESI^+) HRMS: Calculated for $C_{10}H_9NNaO_3$: 214.0475. Found $[M+Na]^+$: 214.0471.

The spectroscopic properties were consistent with the data available in the literature.[37]

2-(4-Nitrobenzyl)prop-2-en-1-ol

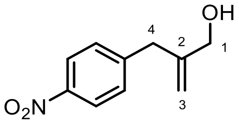

General procedure **C** was followed using the preceding aldehyde (390 mg, 2.04 mmol) and $NaBH_4$ (83 mg, 2.14 mmol). FCC (10 – 20% EtOAc/hexane) gave the title compound (281 mg, 76%) as a colorless oil. ^{1}H NMR (400 MHz, $CDCl_3$) δ 8.14 (2H, d, J = 8.5 Hz, ArCH), 7.36 (2H, d, J = 8.5 Hz, ArCH), 5.18 (1H, m, C**3**-H), 4.88 (1H, m, C**3**-H'), 4.03 (1H, d, J = 4.0 Hz, C**1**-H_2), 3.50 (2H, s, C**4**-H_2); *m/z* (ESI^+) HRMS: Calculated for $C_{10}H_{11}NNaO_3$: 216.0631. Found $[M+Na]^+$: 216.0637.

The spectroscopic properties were consistent with the data available in the literature.[38]

Ethyl 4-(4-nitrobenzyl)pent-4-enoate

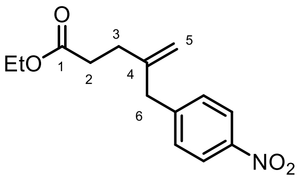

General procedure **D** was followed using the preceding alcohol (270 mg, 1.49 mmol), triethyl orthoacetate (2.8 mL, 14.9 mmol) and propionic acid (20 μL, 0.30 mmol). The title compound (348 mg, 90%) was obtained as a colorless oil and used in the next step without further purification. R*f*: 0.61 (20% EtOAc/hexane); ν_{max} / cm^{-1}: 1731 (s), 1517 (s), 1344 (s); ^{1}H NMR (400 MHz, $CDCl_3$) δ 8.15 (2H, d, *J* = 8.0 Hz, ArCH), 7.35 (2H, d, *J* = 8.0 Hz, ArCH), 4.91 (1H, m, C**5**-H), 4.81 (1H, m, C**5**-H'), 4.12 (2H, q, *J* = 7.0 Hz, OCH_2CH_3), 3.46 (2H, s, C**6**-H_2), 2.47 – 2.44 (2H, m, C**2**-H_2), 2.31 – 2.27 (2H, m, C**3**-H_2), 1.24 (3H, t, *J* = 7.0 Hz, OCH_2CH_3); ^{13}C NMR (101 MHz, $CDCl_3$) δ 173.0 (C**1**), 147.3 (ArC), 146.9 (ArC), 145.9 (C**4**), 129.9 (ArCH), 123.8 (ArCH), 113.0 (C**5**), 60.6 (OCH_2CH_3), 43.1 (C**6**), 32.6 (C**2**), 30.5 (C**3**), 14.4 (OCH_2CH_3); *m/z* (ESI^+) HRMS: Calculated for $C_{14}H_{17}NNaO_4$: 286.1050. Found $[M+Na]^+$: 286.1061.

4-(4-Nitrobenzyl)pent-4-en-1-ol

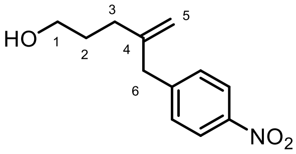

To a solution of the preceding ester (337 mg, 1.28 mmol) in anhydrous Et_2O (6 mL) at 0 °C, was added DIBAL (1.0 M in THF, 3.2 mL) dropwise *via* syringe. The reaction was stirred at 0 °C for 2 hours. Upon completion, the reaction was quenched with sat. solution of Rochelle's salt (20 mL) and allowed to stir at room temperature overnight. The aqueous phase was extracted with Et_2O (3 × 20 mL), and the combined organic layers were washed with brine (50 mL), dried (Na_2SO_4), and concentrated *in vacuo*. FCC (20 – 30% EtOAc/hexane) gave the title compound (184 mg, 83%) as a colorless oil. R*f*: 0.13 (20% EtOAc/hexane); ν_{max} / cm^{-1}: 3365 (br s), 2937 (m), 1517 (s), 1345 (s); ^{1}H NMR (400 MHz, $CDCl_3$) δ 8.15 (2H, d, *J* = 9.0 Hz, ArCH), 7.35 (2H, d, *J* = 9.0 Hz, ArCH), 4.94 (1H, m, C**5**-H), 4.78 (1H, s, C**5**-H'), 3.65 – 3.63 (2H, m, C**1**-H_2), 3.45 (2H, s, C**6**-H_2), 2.07 – 2.03 (2H, m, C**3**-H_2), 1.75 – 1.68 (2H, m, C**2**-H_2); ^{13}C NMR (101 MHz, $CDCl_3$) δ 147.6 (ArC), 147.1 (C**4**), 146.8 (ArC), 129.9 (ArCH), 123.8 (ArCH), 112.9 (C**5**), 62.6 (C**1**), 43.0 (C**6**), 31.8 (C**3**), 30.6 (C**2**); *m/z* (ESI^+) HRMS: Calculated for $C_{12}H_{15}NNaO_3$: 244.0944. Found $[M+Na]^+$: 244.0950.

Benzyl (4-(4-nitrobenzyl)pent-4-en-1-yl)((perfluorobenzoyl)oxy)carbamate (3c)

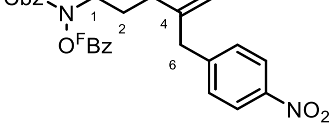

General procedure **I** was followed using CbzNHO^{F}Bz (277 mg, 0.77 mmol), the preceding alcohol (170 mg, 0.77 mmol), triphenylphosphine (222 mg, 0.85 mmol) and diisopropyl azodicarboxylate (0.18 mL, 0.85 mmol). FCC (0 – 10% EtOAc/hexane) gave title compound **3c** (279 mg, 64%) as a colorless oil.

R_f: 0.22 (10% EtOAc/hexane); ν_{max} / cm^{-1}: 1784 (m), 1729 (m), 1521 (m), 1346 (s), 1173 (m); ^{1}H NMR (400 MHz, $CDCl_3$) δ 8.13 (2H, d, *J* = 9.0 Hz, Ph ArCH), 7.36 – 7.30 (7H, m, 5 × Cbz ArCH, 2 × Ph ArCH), 5.21 (2H, s, Cbz CH_2), 4.92 (1H, m, C**5**-H), 4.79 (1H, m, C**5**-H'), 3.73 (2H, t, *J* = 7.5 Hz, C**1**-H_2), 3.41 (2H, s, C**6**-H_2), 2.05 (2H, t, *J* = 7.5 Hz, C**3**-H_2), 1.80 (2H, tt, *J* = 7.5, 7.5 Hz, C**2**-H_2); ^{13}C NMR (101 MHz, $CDCl_3$) δ 155.5 (Cbz C=O), 147.4 (ArC), 146.8 (ArC), 146.1 (C**4**), 135.2 (Cbz ArC), 130.0 (ArCH), 128.7 (Cbz ArCH), 128.6 (Cbz ArCH), 128.3 (Cbz ArCH), 123.8 (Ph ArCH), 113.4 (C**5**), 69.0 (Cbz CH_2), 50.8 (C**1**), 42.8 (C**6**), 32.2 (C**3**), 24.9 (C**2**); ^{19}F NMR (400 MHz, $CDCl_3$) δ -136.0 – -136.0 (2F, m), -145.5 (1F, tt, *J* = 21.0, 5.0 Hz), -158.9 – -159.0 (2F, m); *m/z* (ESI$^+$) HRMS: Calculated for $C_{27}H_{21}F_5N_2NaO_6$: 587.1212. Found $[M+Na]^+$: 587.1196.

The ^{13}C NMR signals corresponding to the pentafluorobenzoyl group could not be resolved due to their weak intensity.

Benzyl 5-nitro-1,3-dihydrospiro[indene-2,2'-pyrrolidine]-1'-carboxylate (4c)

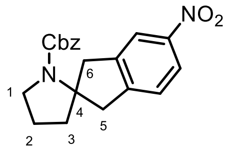

General procedure **J** was followed using cyclization substrate **3c** (56.5 mg, 0.10 mmol), $Pd_2(dba)_3$ (2.30 mg, 0.0025 mmol), CgPPh (4.5 mg, 0.015 mmol), sodium benzoate (1.44 mg, 0.0055 mmol), triethylamine (1.40 µL) and dibutyl ether (1 mL). The reaction was stirred at 130 °C for 48 hours. FCC (2 – 6% EtOAc/hexane) gave title compound **4c** (31 mg, 86%) as a colorless oil. R_f: 0.41 (10% EtOAc/hexane); ν_{max} / cm^{-1}: 1693 (s), 1518 (s), 1406 (s), 1346 (s); *mixture of rotamers A + B (2:1)* ^{1}H NMR (500 MHz, $CDCl_3$) δ 8.07 – 7.93 (1.66H, m, ArCH, A + B), 7.84 (0.33H, s, ArCH, B), 7.37 – 7.26 (4.33H, m, Cbz ArCH A + B, ArCH, A), 7.19 – 7.14 (1H, m, Cbz ArCH A + B, ArCH, B), 6.99 – 6.98 (0.66H, m, Cbz ArCH, A), 5.11 (1.33H, s, Cbz CH_2, A), 5.03 – 4.94 (0.66H, m, Cbz CH_2, B), 3.97 (1.33H, m, C**5**-H_2, A), 3.63 – 3.58 (2.66H, m, C**1**-H_2, A + B, C**5**-H_2, B), 2.95 (0.66H, m, C**6**-H_2, B), 2.80 (0.66H, d, *J* = 16.0 Hz, C**6**-H, A), 2.77 (0.66H, d, *J* = 16.0 Hz, C**6**-H', A), 2.06 – 2.04 (0.66H, m, C**3**-H_2, B), 1.98 – 1.93 (1.33H, m, C**3**-H_2, A), 1.90 – 1.85 (2H, m, C**2**-H_2, A + B); ^{13}C NMR (125 MHz, $CDCl_3$) δ 154.6 (Cbz C=O, B), 154.0 (Cbz C=O, A), 149.8 (ArC, A), 149.4 (ArC, B), 147.2 (ArC, A + B), 143.4 (ArC, A), 143.1 (ArC, B), 137.0 (Cbz ArC, A), 135.9 (Cbz ArC, B), 128.6, 128.4, 128.2, 128.1, 128.0 (Cbz ArCH, A + B), 124.9 (ArCH, A), 124.5 (ArCH, B), 122.2 (ArCH, A + B), 119.7 (ArCH, A), 119.4 (ArCH, B), 70.3 (C**4**, A), 68.9 (C**4**, B), 67.3 (Cbz CH_2, B), 66.6 (Cbz CH_2, A), 48.8 (C**1**, B), 48.1 (C**1**, A), 45.9 (C**6**, B), 45.6 (C**5**, B), 44.0 (C**3**, B), 43.2 (C**6**, A), 43.0 (C**5**, A), 42.0 (C**3**, A), 22.7 (C**2**, A + B); *m/z* (Nanospray) HRMS: Calculated for $C_{20}H_{20}N_2NaO_4$: 375.1328. Found $[M+Na]^+$: 375.1321.

Some signal broadening was observed due to amide-like resonance.

4-Benzylpent-4-enal

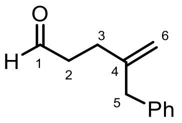

General procedure **G** was followed using 4-benzylpent-4-en-1-ol (755 mg, 4.28 mmol), DMSO (0.91 mL, 12.85 mmol), oxalyl chloride (0.44 mL, 5.14 mmol) and triethylamine (3.0 mL, 21.4 mmol) in CH_2Cl_2. FCC (0 – 10% Et_2O/hexane) gave the title compound (656 mg, 88%) as a colorless oil. ^{1}H NMR (400 MHz, $CDCl_3$) δ 9.72 (1H, t, *J* = 2.0 Hz, C**1**-H), 7.32 – 7.27 (2H, m, ArCH), 7.24 – 7.16 (3H, m, ArCH), 4.86 – 4.81 (2H, m, C**6**-H_2), 3.37 (2H, s, C**5**-H_2), 2.55 (2H, td, *J* = 7.5, 2.0 Hz, C**2**-H_2), 2.31 (2H, br t, *J* = 7.5 Hz, C**3**-H_2); *m/z* (ESI$^+$) HRMS: Calculated for $C_{12}H_{15}O$: 175.1123. Found $[M+H]^+$: 175.1142.

The spectroscopic properties were consistent with the data available in the literature.[39]

4-Benzyl-1-phenylpent-4-en-1-ol

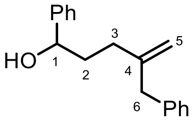

General procedure **H** was followed using the preceding aldehyde (365 mg, 2.10 mmol) and PhMgBr (1.0 M in THF, 3.35 mL). FCC (0 – 10% EtOAc/hexane) gave the title compound (428 mg, 81%) as a colorless oil. R_f: 0.40 (20% EtOAc/hexane); ν_{max} / cm^{-1}: 3358 (br s), 3026 (m), 1644 (m), 1452 (s), 1058 (s); ^{1}H NMR (400 MHz, $CDCl_3$) δ 7.37 – 7.26 (7H, m, Ph ArCH), 7.22 – 7.15 (3H, m, Ph ArCH), 4.86 (1H, m, C**5**-H), 4.78 (1H, m, C**5**-H’), 4.66 – 4.62 (1H, m, C**1**-H), 3.34 (2H, s, C**6**-H_2), 2.17 – 1.84 (4H, m, C**2**-H_2, C**3**-H_2), 1.82 (1H, d, *J* = 3.0 Hz, OH); ^{13}C NMR (101 MHz, $CDCl_3$) δ 148.6 (C**4**), 144.8 (Ph ArC), 139.7 (Ph ArC), 129.2, 128.6, 128.5, 127.7, 126.3, 126.0 (Ph ArCH), 111.6 (C**5**), 74.3 (C**1**), 43.3 (C**6**), 37.0 (C**2**), 31.8 (C**3**); *m/z* (APCI$^+$) HRMS: Calculated for $C_{18}H_{19}$: 235.1481. Found $[M+H–H_2O]^+$: 235.1474.

Benzyl (4-benzyl-1-phenylpent-4-en-1-yl)((perfluorobenzoyl)oxy)carbamate (3d)

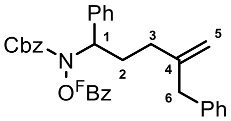

General procedure **I** was followed using CbzNHO^{F}Bz (613 mg, 1.70 mmol), the preceding alcohol (428 mg, 1.70 mmol), triphenylphosphine (494 mg, 1.87 mmol) and diisopropyl azodicarboxylate (0.39 mL, 1.87 mmol). FCC (0 – 5% EtOAc/petroleum ether) gave title compound **3d** (423 mg, 44%) as a colorless oil. R_f: 0.18 (5% EtOAc/ petroleum ether); ν_{max} / cm^{-1}: 1786 (m), 1729 (m), 1651 (m), 1524 (s), 1497 (s), 1325 (s), 1178 (s), 1003 (s); ^{1}H NMR (400 MHz, $CDCl_3$) δ 7.47 – 7.43 (1H, m, Ph ArCH), 7.37 –

7.13 (14H, m, 5 × Cbz ArCH̲, 9 × Ph ArCH̲), 5.34 – 5.06 (3H, m, Cbz CH̲$_2$ + C**1**-H̲), 4.84 – 4.79 (2H, m, C**5**-H̲$_2$), 3.32 (2H, s, C**6**-H̲$_2$), 2.28 – 1.99 (4H, m, C**2**-H̲$_2$ + C**3**-H̲$_2$); ^{13}C NMR (101 MHz, $CDCl_3$) δ 156.9 (Cbz C̲=O), 147.5 (C**4**), 139.4 (Ph ArC̲), 135.2 (Cbz ArC̲), 132.7 (Ph ArC̲), 130.6, 129.1, 128.8, 128.6, 128.5, 128.4, 128.2, 127.8, 127.6, 126.2 (Ph ArC̲H), 112.4 (C**5**), 69.0 (Cbz C̲H$_2$), 63.6 (C**1**), 43.0 (C**6**), 32.0, 29.6 (C**2**, C**3**); *mixture of rotamers A + B (9:1)* ^{19}F NMR (400 MHz, $CDCl_3$) δ -135.5 – -135.9 (1.8F, m, A), -137.3 (0.2F, d, *J* = 18.0 Hz, B), -146.2 (0.9F, br t, *J* = 22.0 Hz, A), -148.8 (0.1F, t, *J* = 20.0 Hz, B), -159.1 – -159.3 (1.8F, m, A), -160.1 – -160.3 (0.2F, m, B); *m/z* (ESI$^+$) HRMS: Calculated for $C_{33}H_{30}F_5N_2O_4$: 613.2120. Found $[M+NH_4]^+$: 613.2125.

The ^{13}C NMR signals corresponding to the pentafluorobenzoyl group could not be resolved due to their weak intensity.

Signal broadening was observed due to amide-like resonance resulting in weak signal intensities in the ^{13}C NMR spectrum.

Benzyl 5'-phenyl-1,3-dihydrospiro[indene-2,2'-pyrrolidine]-1'-carboxylate (4d)

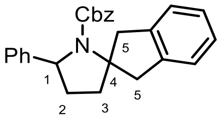

General procedure **J** was followed using cyclization substrate **3d** (32.7 mg, 0.055 mmol), $Pd_2(dba)_3$ (1.92 mg, 0.0021 mmol), CgPPh (4.97 mg, 0.016 mmol), sodium benzoate (0.79 mg, 0.01 mmol), triethylamine (0.7 µL) and dibutyl ether (0.55 mL). The reaction was stirred at 150 °C for 48 hours. FCC (toluene) gave title compound **4d** (11.3 mg, 53%) as a colorless oil. R_f: 0.28 (toluene); ν_{max} / cm^{-1}: 2926 (m), 1697 (s), 1399 (s), 1343 (s); *mixture of rotamers A + B (2:1)* ^{1}H NMR (500 MHz, $CDCl_3$) δ 7.35 – 7.32 (2H, m, ArCH̲, A + B), 7.28 – 7.14 (10H, m, 5 × Cbz ArCH̲, A + B, 5 × ArCH̲, A + B), 7.05 – 7.01 (0.66H, ArCH̲, A), 6.91 – 6.89 (1.33H, m, ArCH̲, A + B), 5.23 – 5.22 (0.33H, m, C**1**-H̲, B), 5.20 – 5.15 (0.66H, m, C**1**-H̲, A), 5.12 – 5.04 (0.66H, m, Cbz CH̲$_2$, B), 5.02 – 4.93 (1.33H, m, Cbz CH̲$_2$, A), 4.39 (0.66H, d, *J* = 15.2 Hz, C**5**-H̲, A), 4.16 (0.33H, d, *J* = 14.0 Hz, C**5**-H̲, B), 3.84 (0.66H, d, *J* = 15.2 Hz, C**5**-H̲, A), 3.59 (0.33H, d, *J* = 15.0 Hz, C**5**-H̲, B), 3.07 (0.33H, d, *J* = 14.0 Hz, C**5**-H̲', B), 2.93 (0.66H, d, *J* = 15.2 Hz, C**5**-H̲', A), 2.89 (0.33H, d, *J* = 15.0 Hz, C**5**-H̲', B), 2.76 (0.66H, d, *J* = 15.2 Hz, C**5**-H̲', A), 2.42 – 2.34 (1H, m, C**2**-H̲, A + B), 2.04 – 1.92 (2H, m, C**3**-H̲$_2$, A + B), 1.85 – 1.82 (1H, m, C**2**-H̲', A + B); ^{13}C NMR (125 MHz, $CDCl_3$) δ 154.0 (Cbz C̲=O, A + B), 144.3 (ArC̲, A), 143.4 (ArC̲, B), 141.9 (ArC̲, A), 141.6 (ArC̲, B), 141.4 (ArC̲, A), 141.1 (ArC̲, B), 137.0 (Cbz ArC̲, A), 136.5 (Cbz ArC̲, B), 128.5, 128.4, 128.3, 127.8, 127.6, 127.3, 126.8, 126.6, 126.4, 126.3, 125.6, 124.7, 124.5 (5 × Cbz ArC̲H A + B, 9 × Ph ArC̲H A + B), 71.4 (C**4**, A + B), 67.0 (Cbz C̲H$_2$, B), 66.2 (Cbz C̲H$_2$, A), 63.6 (C**1**, B), 63.0 (C**1**, A), 45.4 (C**5**, A), 43.5 (C**5'**, A), 42.8 (2 × C**5** B), 40.8 (C**3**, B), 39.0 (C**3**, A), 32.3

(C**2**, A), 32.0 (C**2**, B); *m/z* (ESI$^+$) HRMS: Calculated for $C_{26}H_{26}NO_2$: 384.1958. Found $[M+H]^+$: 384.1944.

Signal broadening was observed due to amide-like resonance.

5-Benzylhex-5-en-2-ol

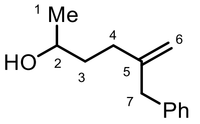

General procedure **H** was followed using 4-benzylpent-4-enal (294 mg, 1.69 mmol) and MeMgBr (3.0 M in Et_2O, 0.9 mL, 2.70 mmol). FCC (10% EtOAc/hexane) gave the title compound (218 mg, 68%) as a colorless oil. R_f: 0.16 (10% EtOAc/hexane); ν_{max} / cm^{-1}: 3349 (br s), 3026 (m), 1644 (m), 1453 (m), 891 (s), 736 (s); ^{1}H NMR (400 MHz, $CDCl_3$) δ 7.31 – 7.27 (2H, m, ArC<u>H</u>), 7.22 – 7.18 (3H, m, ArC<u>H</u>), 4.86 (1H, m, C**6**-<u>H</u>), 4.77 (1H, m, C**6**-<u>H</u>’), 3.78 (1H, tq, *J* = 6.0, 6.0 Hz, C**2**-<u>H</u>), 3.36 (2H, s, C**7**-<u>H$_2$</u>), 2.14 – 1.98 (2H, m, C**4**-<u>H$_2$</u>), 1.66 – 1.56 (2H, m, C**3**-<u>H$_2$</u>), 1.37 (1H, br s, O<u>H</u>), 1.17 (3H, d, *J* = 6.0 Hz, C**1**-<u>H$_3$</u>); ^{13}C NMR (101 MHz, $CDCl_3$) δ 148.9 (C**5**), 139.7 (Ar<u>C</u>), 129.1 (Ar<u>C</u>H), 128.5 (Ar<u>C</u>H), 126.3 (Ar<u>C</u>H), 111.6 (C**6**), 68.0 (C**2**), 43.2 (C**7**), 37.2 (C**3**), 31.8 (C**4**), 23.6 (C**1**); *m/z* (ESI$^+$) HRMS: Calculated for $C_{13}H_{19}O$: 191.1430. Found $[M+H]^+$: 191.1427.

Benzyl (5-benzylhex-5-en-2-yl)((perfluorobenzoyl)oxy)carbamate (3e)

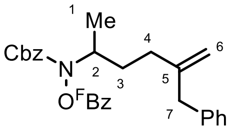

General procedure **I** was followed using CbzNHO^{F}Bz (387 mg, 1.07 mmol), the preceding alcohol (204 mg, 1.07 mmol), triphenylphosphine (312 mg, 1.18 mmol) and diisopropyl azodicarboxylate (0.25 mL, 1.18 mmol). FCC (0 – 1% EtOAc/hexane) gave title compound **3e** (222 mg, 40%) as a colorless oil. R_f: 0.29 (4% EtOAc/hexane); ν_{max} / cm^{-1}: 1787 (m), 1731 (m), 1507 (m), 1276 (m), 764 (s); ^{1}H NMR (400 MHz, $CDCl_3$) δ 7.38 – 7.31 (5H, m, Cbz ArC<u>H</u>), 7.27 – 7.24 (2H, m, Ph ArC<u>H</u>), 7.19 – 7.14 (3H, m, Ph ArC<u>H</u>), 5.21 (2H, br s, Cbz C<u>H$_2$</u>), 4.82 (1H, m, C**6**-<u>H</u>), 4.75 (1H, m, C**6**-<u>H</u>’), 4.35 (1H, m, C**2**-<u>H</u>), 3.30 (2H, br s, C**7**-<u>H$_2$</u>), 2.27 – 1.58 (4H, m, C**3**-<u>H$_2$</u>, C**4**-<u>H$_2$</u>), 1.19 (3H, d, *J* = 7.0 Hz, C**1**-<u>H$_3$</u>); ^{13}C NMR (101 MHz, $CDCl_3$) δ 147.9 (C**5**), 139.6 (Ph Ar<u>C</u>), 135.4 (Cbz Ar<u>C</u>), 129.1, 128.7, 128.6, 128.4, 128.2, 126.2 (Ar<u>C</u>H), 112.0 (C**6**), 68.9 (Cbz <u>C</u>H$_2$), 56.6 (C**2**), 43.1 (C**7**), 32.0, 31.6 (C**3**, C**4**), 17.5 (C**1**); ^{19}F NMR (400 MHz, $CDCl_3$) δ -136.0 – -136.1 (2F, m), -146.3 (1F, m), -159.2 – -159.3 (2F, m); *m/z* (ESI$^+$) HRMS: Calculated for $C_{28}H_{28}F_5N_2O_4$: 551.1964. Found $[M+NH_4]^+$: 551.1949.

The ^{13}C NMR signals corresponding to the pentafluorobenzoyl group could not be resolved due to their weak intensity.

Signal broadening was observed due to amide-like resonance. The ^{13}C signal of the Cbz group could not be resolved due to its weak intensity.

Benzyl 5'-methyl-1,3-dihydrospiro[indene-2,2'-pyrrolidine]-1'-carboxylate (4e)

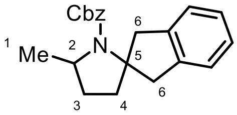

General procedure **J** was followed using cyclization substrate **3e** (53.4 mg, 0.100 mmol), $Pd_2(dba)_3$ (2.29 mg, 0.0025 mmol), CgPPh (4.54 mg, 0.015 mmol), sodium benzoate (1.44 mg, 0.01 mmol), triethylamine (1.40 µL) and dibutyl ether (1 mL). The reaction was stirred at 130 °C for 48 hours. FCC (toluene) gave title compound **4e** (21 mg, 65%) as a colorless solid. R_f: 0.23 (toluene); m.p.: 78 – 80 °C (CH_2Cl_2); ν_{max} / cm^{-1}: 2965 (m), 1694 (s), 1401 (s), 1347 (s); *mixture of rotamers A + B (3:2)* ^{1}H NMR (500 MHz, $CDCl_3$) δ 7.42 – 7.31 (3H, m, ArC<u>H</u>, A + B), 7.22 – 7.03 (6H, m, 5 × Cbz ArC<u>H</u> A + B, 1 × Ph ArC<u>H</u> A + B), 5.17 – 5.06 (2H, m, Cbz C<u>H</u>$_2$, A + B), 4.19 (1.6H, m, C**2**-<u>H</u>, A + B, C**6**-<u>H</u>, A), 3.93 (0.4H, d, J = 16.0 Hz, C**6**-<u>H</u>, B), 3.72 (0.6H, d, J = 15.5 Hz, C**6**-<u>H</u>, A), 3.48 (0.4H, d, J = 16.0 Hz, C**6**-<u>H</u>, B), 2.88 (0.4H, d, J = 16.0 Hz, C**6**-<u>H</u>', B), 2.80 (0.4H, d, J = 16.0 Hz, C**6**-<u>H</u>', B), 2.72 (0.6H, d, J = 15.5 Hz, C**6**-<u>H</u>', A), 2.65 (0.6H, d, J = 15.5 Hz, C**6**-<u>H</u>', A), 2.13 – 2.01 (3H, m, C**3**-<u>H</u> A + B, C**4**-<u>H</u>$_2$ A + B), 1.58 – 1.57 (1H, m, C**3**-<u>H</u>', A + B), 1.30 – 1.24 (3H, m, C**1**-<u>H</u>$_3$, A + B); ^{13}C NMR (125 MHz, $CDCl_3$) δ 154.5 (Cbz <u>C</u>=O, B), 153.8 (Cbz <u>C</u>=O, A), 142.0 (Ph Ar<u>C</u>, A), 141.7 (Ph Ar<u>C</u>, B), 141.3 (Ph Ar<u>C</u>, A), 141.0 (Ph Ar<u>C</u>, B), 137.2 (Cbz Ar<u>C</u>, A), 136.6 (Cbz Ar<u>C</u>, B), 128.4, 128.3, 128.2, 127.8, 127.6, 126.4, 126.2, 126.0, 124.5, 124.3 (5 × Cbz Ar<u>C</u>H A + B, 4 × Ph Ar<u>C</u>H A + B), 70.7 (C**5**, A), 69.5 (C**5**, B), 66.4 (Cbz <u>C</u>H$_2$, B), 66.2 (Cbz <u>C</u>H$_2$, A), 55.8 (C**2**, B), 54.9 (C**2**, A), 46.1, 45.2 (C**6**, B), 44.1, 42.5 (C**6**, A), 41.4 (C**4**, B), 39.6 (C**4**, A), 29.6 (C**3**, A), 29.3 (C**3**, B), 21.1 (C**1**, A), 20.2 (C**1**, B); *m/z* (ESI$^+$) HRMS: Calculated for $C_{21}H_{24}NO_2$: 322.1802. Found $[M+H]^+$: 322.1795.

The structure of this compound was confirmed by single crystal X-ray analysis after recrystallization (CH_2Cl_2).

Single Crystal X-ray Analysis of 4e

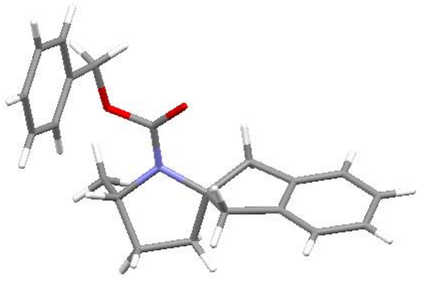

3-Iodoquinoline

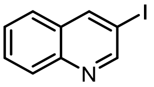

A flame-dried sealed tube was charged with 3-bromoquinoline (2.0 mL, 14.4 mmol), dried NaI (4.33 g, 28.9 mmol) and CuI (280 mg, 1.44 mmol), and the system was purged with argon. Anhydrous 1,4-dioxane (14.0 mL) and *N,N'*-dimethylethylenediamine (0.33 mL, 2.89 mmol) were added *via* syringe. The tube was then sealed, and the mixture was heated at 110 ºC for 22 hours. Upon completion, the reaction was cooled to room temperature, diluted with saturated aq. NH_4Cl (50 mL) and extracted with CH_2Cl_2 (3 × 50 mL). The combined organic layers were washed with brine (50 mL), dried (Na_2SO_4) and concentrated *in vacuo*. The title compound (3.50 g, 95%) was obtained as a pale yellow solid and used in the next step without further purification. ^{1}H NMR (400 MHz, $CDCl_3$) δ 9.04 (1H, d, *J* = 2.0 Hz, ArCH), 8.55 (1H, d, *J* = 2.0 Hz, ArCH), 8.07 (1H, d, *J* = 8.5 Hz, ArCH), 7.76 – 7.71 (2H, m, ArCH), 7.59 – 7.55 (1H, m, ArCH); *m/z* (ESI$^+$) HRMS: Calculated for C_9H_7IN: 255.9618. Found $[M+H]^+$: 255.9620.

The spectroscopic properties were consistent with the data available in the literature.[40]

3-(Quinolin-3-yl)propanal

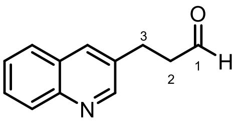

A flask was charged with 3-iodoquinoline (3.50 g, 13.7 mmol), benzyltriethylammonium chloride (3.16 g, 13.7 mmol), $Pd(OAc)_2$ (31 mg, 0.14 mmol) and $NaHCO_3$ (2.88 g, 34.3 mmol), and the system was purged with argon. Allyl alcohol (1.41 mL, 20.58 mmol) and DMF (55 mL) were then added sequentially *via* syringe. The mixture was heated at 110 ºC for 5 hours. Upon completion, the reaction was cooled to room temperature, filtered through a pad of silica gel, diluted with a mixture of brine/water 1:1 (50 mL) and extracted with EtOAc (3 × 50 mL). The combined organic layers were dried (Na_2SO_4) and concentrated *in vacuo*. FCC (10 – 30% EtOAc/CH_2Cl_2) gave the title compound (709 mg, 28%) as a colorless oil. R_f: 0.27 (30% EtOAc/CH_2Cl_2); ν_{max} / cm^{-1}: 2926 (m), 1721 (s), 1573 (m), 1496 (s); ^{1}H NMR (400 MHz, $CDCl_3$) δ 9.86 (1H, s, C**1**-H), 8.80 (1H, d, *J* = 2.0 Hz, ArCH), 8.07 (1H, d, *J* = 8.5 Hz, ArCH), 7.95 (1H, d, *J* = 1.0 Hz, ArCH), 7.76 (1H, d, *J* = 8.5 Hz, ArCH), 7.69 – 7.65 (1H, m, ArCH), 7.55 – 7.51 (1H, m, ArCH), 3.14 (2H, t, *J* = 7.5 Hz, C**3**-H_2), 2.92 (2H, t, *J* = 7.5 Hz, C**2**-H_2); ^{13}C NMR (101 MHz, $CDCl_3$) δ 200.6 (C**1**), 151.7 (ArCH), 147.1 (ArC), 134.6 (ArCH), 133.2 (ArC), 129.3 (ArCH), 129.1 (ArCH), 128.1 (ArC), 127.5 (ArCH), 127.0 (ArCH), 44.9 (C**2**), 25.4 (C**3**); *m/z* (ESI$^+$) HRMS: Calculated for $C_{12}H_{12}NO$: 186.0913. Found $[M+H]^+$: 186.0908.

2-(Quinolin-3-ylmethyl)acrylaldehyde

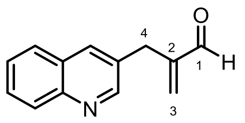

General procedure **B** was followed using 4-(dimethylamino)benzoic acid (55 mg, 0.33 mmol), pyrrolidine (30 μL, 0.33 mmol), formaldehyde (0.24 mL, 3.30 mmol) and the preceding aldehyde (612 mg, 3.30 mmol). FCC (0 – 20% EtOAc/hexane) gave the title compound (379 mg, 58%) as a colorless oil. R*f*: 0.27 (30% EtOAc/hexane); ν_{max} / cm^{-1}: 2823 (m), 1683 (s), 1495 (s), 1326 (m); ^{1}H NMR (400 MHz, $CDCl_3$) δ 9.63 (1H, s, C**1**-H), 8.76 (1H, d, *J* = 2.0 Hz, ArCH), 8.08 (1H, d, *J* = 8.5 Hz, ArCH), 7.96 (1H, s, ArCH), 7.77 (1H, d, *J* = 8.5 Hz, ArCH), 7.70 – 7.66 (1H, m, ArCH), 7.55 – 7.51 (1H, m, ArCH), 6.21 (1H, m, C**3**-H), 6.16 (1H, m, C**3**-H'), 3.76 (2H, s, C**4**-H_2); ^{13}C NMR (101 MHz, $CDCl_3$) δ 193.7 (C**1**), 152.0 (ArCH), 148.8 (C**2**), 147.2 (ArC), 135.7 (C**3**), 135.6 (ArCH), 131.0 (ArC), 129.3 (ArCH), 129.2 (ArCH), 128.1 (ArC), 127.6 (ArCH), 127.0 (ArCH), 31.8 (C**4**); *m/z* (ESI^+) HRMS: Calculated for $C_{13}H_{12}NO$: 198.0913. Found $[M+H]^+$: 198.0908.

2-(Quinolin-3-ylmethyl)prop-2-en-1-ol

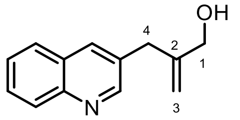

General procedure **C** was followed using the preceding aldehyde (369 mg, 1.87 mmol) and $NaBH_4$ (76 mg, 1.97 mmol). FCC (40% EtOAc/hexane) gave the title compound (290 mg, 78%) as a colorless oil. R*f*: 0.14 (40% EtOAc/hexane); ν_{max} / cm^{-1}: 3240 (br s), 2908 (m), 1653 (m), 1497 (s); ^{1}H NMR (400 MHz, $CDCl_3$) δ 8.78 (1H, d, *J* = 2.0 Hz, ArCH), 8.09 (1H, d, *J* = 8.5 Hz, ArCH), 7.97 (1H, s, ArCH), 7.78 (1H, d, *J* = 8.5 Hz, ArCH), 7.70 – 7.66 (1H, m, ArCH), 7.55 – 7.52 (1H, m, ArCH), 5.22 (1H, m, C**3**-H), 4.94 (1H, m, C**3**-H'), 4.11 (2H, d, *J* = 5.0 Hz, C**1**-H_2), 3.61 (2H, s, C**4**-H_2); ^{13}C NMR (101 MHz, $CDCl_3$) δ 152.1 (ArCH), 147.6 (C**2**), 146.9 (ArC), 135.4 (ArCH), 132.0 (ArC), 129.1 (ArCH), 128.2 (ArC), 127.5 (ArCH), 126.9 (ArCH), 112.7 (C**3**), 65.3 (C**1**), 37.4 (C**4**); *m/z* (ESI^+) HRMS: Calculated for $C_{13}H_{14}NO$: 200.1070. Found $[M+H]^+$: 200.1061.

Ethyl 4-(quinolin-3-ylmethyl)pent-4-enoate

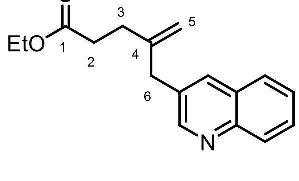

General procedure **D** was followed using the preceding alcohol (244 mg, 1.23 mmol), triethyl orthoacetate (2.3 mL, 12.3 mmol) and propionic acid (20 μL, 0.25 mmol). The title compound (326 mg,

quantitative) was obtained as a colorless oil and used in the next step without further purification. R_f: 0.56 (40% EtOAc/hexane); ν_{max} / cm^{-1}: 2980 (m), 1731 (s), 1646 (m), 1495 (m), 1176 (s); ^1H NMR (400 MHz, $CDCl_3$) δ 8.76 (1H, d, *J* = 2.0 Hz, ArCH), 8.08 (1H, d, *J* = 8.5 Hz, ArCH), 7.93 (1H, s, ArCH), 7.77 (1H, d, *J* = 8.5 Hz, ArCH), 7.69 – 7.65 (1H, m, ArCH), 7.55 – 7.51 (1H, m, ArCH), 4.93 (1H, m, C**5**-H), 4.84 (1H, s, C**5**-H’), 4.11 (2H, q, *J* = 7.0 Hz, OCH_2CH_3), 3.54 (2H, s, C**6**-H_2), 2.51 – 2.47 (2H, m, C**2**-H_2), 2.37 – 2.33 (2H, m, C**3**-H_2), 1.22 (3H, t, *J* = 7.0 Hz, OCH_2CH_3); ^{13}C NMR (101 MHz, $CDCl_3$) δ 173.4 (C**1**), 152.3 (ArCH), 147.2 (ArC), 146.4 (C**4**), 135.2 (ArCH), 132.1 (ArC), 129.3 (ArCH), 129.0 (ArCH), 128.2 (ArC), 127.6 (ArCH), 126.8 (ArCH), 112.7 (C**5**), 60.6 (OCH_2CH_3), 40.5 (C**6**), 32.6 (C**2**), 30.5 (C**3**), 14.3 (OCH_2CH_3); *m/z* (ESI^+) HRMS: Calculated for $C_{17}H_{20}NO_2$: 270.1489. Found $[M+H]^+$: 270.1477.

4-(Quinolin-3-ylmethyl)pent-4-en-1-ol

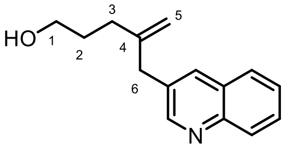

To a solution of the preceding ester (675 mg, 2.51 mmol) in anhydrous Et_2O (12 mL) at 0 °C, was added DIBAL (1.0 M in THF, 5.6 mL) dropwise *via* syringe. The reaction was stirred at -40 °C for 1 hour. Upon completion, the reaction was quenched with sat. solution of Rochelle’s salt (30 mL) and allowed to stir at room temperature overnight. The aqueous phase was extracted with Et_2O (3 × 30 mL), and the combined organic layers were washed with brine (50 mL), dried (Na_2SO_4), and concentrated *in vacuo*. FCC (0 – 2% MeOH/CH_2Cl_2) gave the title compound (308 mg, 54%) as a colorless oil. R_f: 0.48 (10% MeOH/CH_2Cl_2); ν_{max} / cm^{-1}: 3295 (br s), 2924 (m), 1497 (s), 1265 (s); ^1H NMR (500 MHz, $CDCl_3$) δ 8.76 (1H, d, *J* = 2.0 Hz, ArCH), 8.08 (1H, d, *J* = 8.5 Hz, ArCH), 7.93 (1H, d, *J* = 1.5 Hz, ArCH), 7.77 (1H, dd, *J* = 8.0, 1.5 Hz, ArCH), 7.67 (1H, ddd, *J* = 8.5, 7.0, 1.5 Hz, ArCH), 7.53 (1H, ddd, *J* = 8.0, 7.0, 1.5 Hz, ArCH), 4.95 (1H, m, C**5**-H), 4.82 (1H, m, C**5**-H’), 3.65 (2H, t, *J* = 6.5 Hz, C**1**-H_2), 3.54 (2H, s, C**6**-H_2), 2.12 – 2.09 (2H, m, C**3**-H_2), 1.78 – 1.73 (3H, m, C**2**-H_2, OH); ^{13}C NMR (125 MHz, $CDCl_3$) δ 154.3 (ArCH), 147.5 (C**4**), 147.1 (ArC), 135.2 (ArCH), 132.4 (ArC), 129.3 (ArCH), 129.0 (ArCH), 128.2 (ArC), 127.6 (ArCH), 126.8 (ArCH), 112.6 (C**5**), 62.6 (C**1**), 40.4 (C**6**), 31.9 (C**3**), 30.7 (C**2**); *m/z* (ESI^+) HRMS: Calculated for $C_{15}H_{18}NO$: 228.1383. Found $[M+H]^+$: 228.1378.

Benzyl ((perfluorobenzoyl)oxy)(4-(quinolin-3-ylmethyl)pent-4-en-1-yl)carbamate (3g)

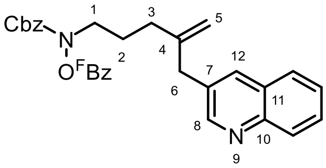

General procedure **I** was followed using CbzNHO^{F}Bz (473 mg, 1.31 mmol), the preceding alcohol (297 mg, 1.31 mmol), triphenylphosphine (381 mg, 1.44 mmol) and diisopropyl azodicarboxylate (0.30 mL, 1.44 mmol). FCC (0 – 1% acetone/toluene, SiO_2 particle size 19 – 37 μm) gave title compound **3g** (432 mg, 58%) as a colorless oil. R$_f$: 0.24 (5% acetone/toluene); ν_{max} / cm^{-1}: 1782 (m), 1726 (m), 1523 (s), 1497 (s), 1326 (m), 1172 (s); ^{1}H NMR (400 MHz, $CDCl_3$) δ 8.74 (1H, d, *J* = 2.0 Hz, C**8**-H), 8.07 (1H, d, *J* = 8.5 Hz, quinol. ArCH), 7.90 (1H, br s, C**12**-H), 7.76 (1H, d, *J* = 8.0 Hz, quinol. ArCH), 7.69 – 7.65 (1H, m, quinol. ArCH), 7.54 – 7.50 (1H, m, quinol. ArCH), 7.36 – 7.30 (5H, m, Cbz ArCH), 5.20 (2H, s, Cbz CH_2), 4.94 (1H, m, C**5**-H), 4.83 (1H, m, C**5**-H'), 3.74 (2H, t, *J* = 7.5 Hz, C**1**-H_2), 3.50 (2H, s, C**6**-H_2), 2.11 (2H, t, *J* = 7.5 Hz, C**3**-H_2), 1.83 (2H, tt, *J* = 7.5, 7.5 Hz, C**2**-H_2); ^{13}C NMR (101 MHz, $CDCl_3$) δ 155.5 (Cbz C=O), 152.2 (C**8**), 147.1 (C**10**), 146.5 (C**4**), 135.3 (Cbz ArC), 135.2 (C**12**), 132.2 (C**7**), 129.3 (quinol. ArCH), 129.0 (quinol. ArCH), 128.7 (Cbz ArCH), 128.6 (Cbz ArCH), 128.3 (Cbz ArCH), 128.2 (C**11**), 127.5 (quinol. ArCH), 126.8 (quinol. ArCH), 113.2 (C**5**), 68.9 (Cbz CH_2), 50.9 (C**1**), 40.3 (C**6**), 32.3 (C**3**), 25.0 (C**2**); ^{19}F NMR (400 MHz, $CDCl_3$) δ -135.9 – -136.0 (2F, m), -145.7 (1F, tt, *J* = 21.0, 5.2 Hz), -159.0 – -159.1 (2F, m); *m/z* (ESI$^+$) HRMS: Calculated for $C_{30}H_{24}F_5N_2O_4$: 571.1651. Found [M+H]$^+$: 571.1652.

The ^{13}C NMR signals corresponding to the pentafluorobenzoyl group could not be resolved due to their weak intensity.

Benzyl 1,3-dihydrospiro[cyclopenta[*c*]quinoline-2,2'-pyrrolidine]-1'-carboxylate (4g)

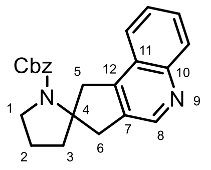

General procedure **J** was followed using cyclization substrate **3g** (57.1 mg, 0.10 mmol), $Pd_2(dba)_3$ (4.67 mg, 0.005 mmol), CgPPh (15.4 mg, 0.05 mmol), sodium benzoate (1.44 mg, 0.01 mmol), triethylamine (1.40 μL) and dibutyl ether (1 mL). The reaction was stirred at 150 °C for 48 hours. FCC (2 – 5% acetone/toluene) gave title compound **4g** (14.5 mg, 40%) as a colorless oil. R$_f$: 0.22 (10% acetone/toluene); ν_{max} / cm^{-1}: 2923 (m), 1695 (s), 1407 (s), 1356 (s); *mixture of rotamers A + B (3:2)* ^{1}H NMR (500 MHz, $CDCl_3$) δ 8.80 (0.6H, s, C**8**-H, A), 8.66 (0.4H, s, C**8**-H, B), 8.28 – 8.10 (1H, m, quinol. ArCH, A +B), 7.78 – 7.58 (3H, m, quinol. ArCH, A + B), 7.38 – 7.32 (3.4H, m, Cbz ArCH, A + B), 7.05 (0.4H, t, *J* = 7.0 Hz, Cbz ArCH, B), 6.91 (0.6H, t, *J* = 7.5 Hz, Cbz ArCH, A), 6.77 (0.6H, d, *J* = 7.5 Hz, Cbz ArCH, A), 5.11 (1.2H, s, Cbz CH_2, A), 4.95 – 4.87 (0.8H, m, Cbz CH_2, B), 4.24 (0.6H, d, *J* = 17.0 Hz, C**5**-H, A), 4.05 (0.6H, d, *J* = 16.0 Hz, C**6**-H, A), 3.85 (0.4H, d, *J* = 18.0 Hz, C**5**-H, B), 3.73 – 3.56 (2.4H, m, C**1**-H_2 A + B, C**6**-H B), 3.31 (0.4H, d, *J* = 18.0 Hz, C**5**-H', B), 3.21 (0.6H, d, *J* = 17.0 Hz, C**5**-H', A), 3.15 (0.4H, d, *J* = 16.6 Hz, C**6**-H', B), 3.00 (0.6H, d, *J* = 16.0 Hz, C**6**-H', A), 2.16 – 1.92 (4H, m, C**2**-H_2 A + B, C**3**-H_2 A + B); ^{13}C NMR (125 MHz, $CDCl_3$) δ 154.6 (Cbz C=O, B), 154.1

(Cbz C=O, A), 150.6 (C**10**, A + B), 145.3 (C**8**, A), 144.7 (C**8**, B), 136.9 (Cbz ArC, A), 135.5 (Cbz ArC, B), 134.8 (C**7**, A), 134.4 (C**7**, B), 129.8 (quinol. ArCH, A), 129.6 (quinol. ArCH, B), 128.7, 128.5, 128.3, 128.2, 128.1, 128.0, 127.4 (5 × Cbz ArCH A + B, 2 × quinol. ArCH A + B, C**11**, A + B), 125.9 (C**12**, A), 125.6 (C**12**, B), 124.4 (quinol. ArCH, A), 124.2 (quinol. ArCH, B), 70.2 (C**4**, A), 68.6 (C**4**, B), 67.3 (Cbz CH_2, B), 66.7 (Cbz CH_2, A), 48.8 (C**1**, B), 48.1 (C**1**, A), 45.1, 45.0 (C**5** B, C**6** B), 44.6 (C**3**, B), 42.7 (C**3**, A), 42.3, 42.2 (C**5** A, C**6** A), 23.0 (C**2**, A + B); *m/z* (ESI^+) HRMS: Calculated for $C_{23}H_{23}N_2O_2$: 359.1754. Found $[M+H]^+$: 359.1757.

*Significant signal broadening was observed in the ^{13}C NMR spectrum resulting in weak signal intensities. The chemical shift for C**10** was assigned using the HMBC correlation.*

*Note - the regioisomer assignment is based upon the retention of the signal corresponding to C**8** on **4g** (^{1}H δ 8.74 and ^{13}C δ 152.2 for C**8** in starting material **3g**, translating to ^{1}H δ 8.80 and ^{13}C δ 145.3 for C**8** in product **4g**.) This is further backed up by the disappearance of a ^{1}H signal at C**12**.*

*Additional evidence is obtained through the observation of an nOe effect between C**6**-H and C**8**-H, as well as C**5**-H with another quinoline ArCH.*

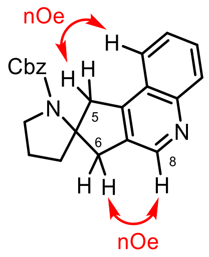

3-(Furan-3-yl)propan-1-ol

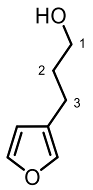

To a solution of 3-bromofuran (3.23 g, 21.5 mmol) in THF (21.5 mL) at -78 °C, was added *n*-butyllithium (1.6 M in hexane, 14.8 mL) using a syringe pump at a rate of 24.6 mL/h. The reaction was then stirred at -78 °C for 45 minutes before adding oxetane (1.6 mL, 23.7 mmol) *via* syringe. After stirring for 5 minutes, $BF_3 \cdot OEt_2$ (2.9 mL, 23.7 mmol) was added using a syringe pump at a rate of 8.2 mL/h, maintaining the temperature at -78 °C. The reaction was then stirred at that temperature for 5 h. Upon completion, the reaction was slowly quenched with sat. $NaHCO_3$ solution (70 mL) and allowed to warm to room temperature overnight. The aqueous layer was extracted with Et_2O (3 × 50 mL), and the combined organic layers were washed with sat. $NaHCO_3$ (50 mL), water (50 mL), brine (50 mL), dried (Na_2SO_4) and concentrated *in vacuo*. Due to high volatility, the title compound (2.50 g, 92%) was

used directly in the next step without further purification. ^{1}H NMR (400 MHz, $CDCl_3$) δ 7.36 (1H, t, *J* = 2.0 Hz, ArCH), 7.24 (1H, br s, ArCH), 6.28 (1H, br s, ArCH), 3.69 (2H, td, *J* = 6.0, 6.0 Hz, C**1**-H_2), 2.53 (2H, t, *J* = 8.0 Hz, C**3**-H_2), 1.87 – 1.80 (2H, m, C**2**-H_2); *m/z* (APCI) HRMS: Calculated for $C_7H_{11}O_2$: 127.0754. Found $[M+H]^+$: 127.0751.

The spectroscopic properties were consistent with the data available in the literature.[41]

3-(Furan-3-yl)propanal

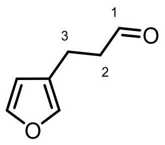

General procedure **G** was followed using the preceding alcohol (2.50 g, 19.8 mmol), DMSO (4.2 mL, 59.5 mmol), oxalyl chloride (2.1 mL, 23.8 mmol) and triethylamine (13.8 mL). Due to high volatility, the title compound (2.50 g, quantitative) was used directly in the next step without further purification. ^{1}H NMR (400 MHz, $CDCl_3$) δ 9.81 (1H, t, *J* = 1.0 Hz, C**1**-H), 7.35 (1H, t, *J* = 1.5 Hz, ArCH), 7.24 (1H, s, ArCH), 6.27 (1H, s, ArCH), 2.82 – 2.69 (4H, m, C**2**-H_2, C**3**-H_2); *m/z* ($APCI^+$) HRMS: Calculated for $C_7H_9O_2$: 125.0597. Found $[M+H]^+$: 125.0591.

The spectroscopic properties were consistent with the data available in the literature.[42]

2-(Furan-3-ylmethyl)acrylaldehyde

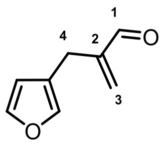

General procedure **B** was followed using 4-(dimethylamino)benzoic acid (334 mg, 1.98 mmol), pyrrolidine (170 μL, 1.98 mmol), formaldehyde (1.5 mL, 19.8 mmol) and the preceding aldehyde (2.46 g, 19.8 mmol). Due to high volatility, the title compound (2.75 g, quantitative) was used directly in the next step without further purification. R_f: 0.32 (30% Et_2O/hexane); ν_{max} / cm^{-1}: 1688 (s), 1606 (s), 1276 (m), 1023 (s); ^{1}H NMR (400 MHz, $CDCl_3$) δ 9.61 (C**1**-H), 7.37 (1H, s, ArCH), 7.26 (1H, m, ArCH), 6.24 (1H, s, ArCH), 6.22 (1H, m, C**3**-H), 6.07 (1H, m, C**3**-H'), 3.38 (2H, s, C**4**-H_2); ^{13}C NMR (101 MHz, $CDCl_3$) δ 194.1 (C**1**), 149.0 (C**2**), 143.2 (ArCH), 140.2 (ArCH), 135.0 (C**3**), 121.1 (ArC), 111.4 (ArCH), 23.5 (C**4**); *m/z* ($APCI^+$) HRMS: Calculated for $C_8H_9O_2$: 137.0597. Found $[M+H]^+$: 137.0590.

2-(Furan-3-ylmethyl)prop-2-en-1-ol

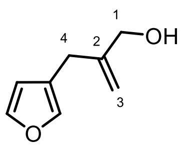

General procedure **C** was followed using the preceding aldehyde (112 mg, 0.82 mmol) and $NaBH_4$ (32.5 mg, 0.86 mmol). FCC (20 – 30% Et_2O/hexane) gave the title compound (53.0 mg, 53%) as a colorless oil. R_f: 0.20 (30% Et_2O/hexane); ν_{max} / cm^{-1}: 3338 (br s), 1654 (m), 1501 (m), 1021 (s); ^1H NMR (400 MHz, $CDCl_3$) δ 7.37 (1H, t, *J* = 1.5 Hz, ArCH), 7.26 (1H, s, ArCH), 6.27 (1H, s, ArCH), 5.10 (1H, m, C**3**-H), 4.95 (1H, m, C**3**-H'), 4.09 (2H, d, *J* = 6.0 Hz, C**1**-H_2), 3.22 (2H, s, C**4**-H_2), 1.39 (1H, t, *J* = 6.0 Hz, OH); ^{13}C NMR (101 MHz, $CDCl_3$) δ 147.5 (C**2**), 143.1 (ArCH), 139.9 (ArCH), 122.2 (ArC), 111.4 (ArCH), 111.2 (C**3**), 65.5 (C**1**), 28.9 (C**4**); *m/z* ($APCI^+$) HRMS: Calculated for C_8H_9O: 121.0648. Found $[M+H–H_2O]^+$: 121.0643.

Ethyl 4-(furan-3-ylmethyl)pent-4-enoate

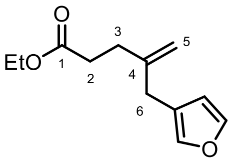

General procedure **D** was followed using the preceding alcohol 593 mg, 4.29 mmol), triethyl orthoacetate (8.05 mL, 42.9 mmol) and propionic acid (60 μL, 0.86 mmol). FCC (0 – 10% EtOAc/hexane) gave the title compound (619 mg, 69%) as a colorless oil. R_f: 0.56 (10% EtOAc/hexane); ν_{max} / cm^{-1}: 1732 (s), 1276 (m), 1260 (m), 1157 (m); ^1H NMR (400 MHz, $CDCl_3$) δ 7.35 (1H, t, *J* = 1.5 Hz, ArCH), 7.24 (1H, s, ArCH), 6.24 (1H, s, ArCH), 4.84 (1H, m, C**5**-H), 4.80 (1H, m, C**5**-H'), 4.12 (2H, q, *J* = 7.0 Hz, OCH_2CH_3), 3.15 (2H, s, C**6**-H_2), 2.47 – 2.43 (2H, m, C**3**-H_2), 2.35 – 2.32 (2H, m, C**2**-H_2), 1.24 (3H, t, *J* = 7.0 Hz, OCH_2CH_3); ^{13}C NMR (101 MHz, $CDCl_3$) δ 173.3 (C**1**), 146.6 (C**4**), 143.0 (ArCH), 139.9 (ArCH), 122.5 (ArC), 111.5 (ArCH), 111.1 (C**5**), 60.5 (OCH_2CH_3), 32.7 (C**3**), 32.3 (C**6**), 30.5 (C**2**), 14.4 (OCH_2CH_3); *m/z* (ESI^+) HRMS: Calculated for $C_{12}H_{16}NaO_3$: 231.0992. Found $[M+Na]^+$: 231.0996.

4-(Furan-3-ylmethyl)pent-4-en-1-ol

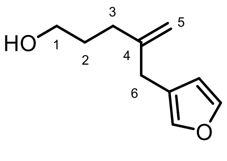

General procedure **F** was followed using the preceding ester (619 mg, 2.97 mmol), $LiAlH_4$ (169 mg, 4.46 mmol) and anhydrous THF at 0 °C for 1.5 hours. FCC (0 – 20% EtOAc/hexane) gave the title compound (390 mg, 79%) as a colorless oil. R_f: 0.20 (20% EtOAc/hexane); ν_{max} / cm^{-1}: 3349 (br s), 1647 (m), 1501 (m), 1439 (m), 1062 (s), 1022 (s); ^1H NMR (400 MHz, $CDCl_3$) δ 7.35 (1H, t, *J* = 1.0 Hz, ArCH), 7.24 (1H, s, ArCH), 6.24 (1H, s, ArCH), 4.84 (1H, m, C**5**-H), 4.82 (1H, m, C**5**-H'), 3.64 (2H, td, *J* = 6.5, 6.5 Hz, C**1**-H_2), 3.15 (2H, s, C**6**-H_2), 2.11 – 2.07 (2H, m, C**3**-H_2), 1.75 – 1.68 (2H, m,

C**2**-H$_2$); ^{13}C NMR (101 MHz, $CDCl_3$) δ 147.8 (C**4**), 143.0 (ArCH), 139.8 (ArCH), 122.7 (ArC), 111.5 (ArCH), 111.0 (C**5**), 62.7 (C**1**), 32.0 (C**6**), 31.8 (C**3**), 30.6 (C**2**); *m/z* (ESI$^+$) HRMS: *not observed*.

Benzyl (4-(furan-3-ylmethyl)pent-4-en-1-yl)((perfluorobenzoyl)oxy)carbamate (3f)

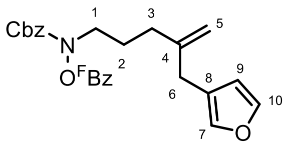

General procedure **I** was followed using CbzNHO^{F}Bz (652 mg, 1.81 mmol), the preceding alcohol (316 mg, 1.81 mmol), triphenylphosphine (521 mg, 1.99 mmol) and diisopropyl azodicarboxylate (0.42 mL, 1.99 mmol). FCC (0 – 4% EtOAc/hexane) gave title compound **3f** (475 mg, 52%) as a colorless oil. R_f: 0.26 (5% EtOAc/hexane); ν_{max} / cm^{-1}: 1784 (m), 1729 (m), 1524 (m), 1499 (s), 132 (m), 1170 (s); ^{1}H NMR (400 MHz, $CDCl_3$) δ 7.38 – 7.30 (6H, m, 5 × Cbz ArCH, C**10**-H), 7.20 (1H, s, C**7**-H), 6.22 (1H, s, C**9**-H), 5.22 (2H, s, Cbz CH$_2$), 4.82 (1H, m, C**5**-H), 4.80 (1H, m, C**5**-H'), 3.74 (2H, t, *J* = 7.5 Hz, C**1**-H$_2$), 3.11 (2H, s, C**6**-H$_2$), 2.08 (2H, t, *J* = 7.5 Hz, C**3**-H$_2$), 1.79 (2H, tt, *J* = 7.5, 7.5 Hz, C**2**-H$_2$); ^{13}C NMR (101 MHz, $CDCl_3$) δ 155.5 (Cbz C=O), 146.7 (C**4**), 143.0 (C**10**), 139.9 (C**7**), 135.3 (Cbz ArC), 128.7 (Cbz ArCH), 128.3 (Cbz ArCH), 122.6 (C**8**), 111.5 (C**5**), 111.4 (C**9**), 68.9 (Cbz CH$_2$), 51.0 (C**1**), 32.2 (C**3**), 32.0 (C**6**), 24.9 (C**2**); ^{19}F NMR (400 MHz, $CDCl_3$) δ -135.8 – -135.9 (2F, m), -145.8 (1F, tt, *J* = 21.0, 5.0 Hz), -159.0 – -159.2 (2F, m); *m/z* (ESI$^+$) HRMS: Calculated for $C_{25}H_{21}F_5NO_5$: 510.1334. Found [M+H]$^+$: 510.1327.

The ^{13}C NMR signals corresponding to the pentafluorobenzoyl group could not be resolved due to their weak intensity.

Benzyl 4,6-dihydrospiro[cyclopenta[*b*]furan-5,2'-pyrrolidine]-1'-carboxylate (4f)

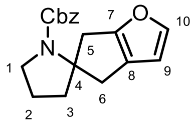

General procedure **J** was followed using cyclization substrate **3f** (51.0 mg, 0.10 mmol), $Pd_2(dba)_3$ (2.29 mg, 0.0025 mmol), CgPPh (4.54 mg, 0.015 mmol), sodium benzoate (1.44 mg, 0.01 mmol), triethylamine (1.40 μL) and dibutyl ether (1 mL). The reaction was stirred at 130°C for 48 hours. FCC (toluene) gave title compound **4f** (20 mg, 70%) as a colorless oil. R_f: 0.20 (10% EtOAc/hexane); ν_{max} / cm^{-1}: 2927 (m), 1693 (s), 1403 (s), 1335 (s); *mixture of rotamers A + B (55:45)* ^{1}H NMR (500 MHz, $CDCl_3$) δ 7.37 – 7.30 (4H, m, Cbz ArCH, A + B, C**10**-H, A + B), 7.23 – 7.22 (1H, m, Cbz ArCH, A + B), 7.04 (1H, m, Cbz ArCH, A + B), 6.22 – 6.20 (1H, m, C**9**-H, A + B), 5.13 – 5.09 (2H, m, Cbz CH$_2$, A + B), 3.60 – 3.49 (2.55H, m, C**1**-H$_2$, A + B, C**6**-H, A), 3.34 – 3.27 (1H, m, C**5**-H, A, C**6**-H, B), 3.12 (0.45H, d, *J* = 14.5 Hz, C**5**-H, B), 2.65 (0.45H, d, *J* = 16.0 Hz, C**6**-H', B), 2.57 – 2.51 (1H, m, C**6**-H', A, C**5**-H', B), 2.43 (0.55H, d, *J* = 14.2 Hz, C**5**-H', A), 2.12 (0.9H, t, *J* = 7.0 Hz, C**3**-H$_2$, B), 2.07 (1.1H,

t, J = 7.0 Hz, C**3**-H2, A), 1.90 – 1.83 (2H, m, C**2**-H2, A + B); ^{13}C NMR (125 MHz, $CDCl_3$) δ 155.2 (C**7**, A), 154.9 (Cbz C=O, B), 154.8 (C**7**, B), 154.0 (Cbz C=O, A), 145.4 (C**10**, B), 145.0 (C**10**, A), 137.2 (Cbz ArC, A), 136.4 (Cbz ArC, B), 128.6, 128.5, 128.4, 128.0, 127.8, 127.5 (Cbz ArCH, A + B), 122.1 (C**8**, A), 122.0 (C**8**, B), 108.2 (C**9**, A), 108.1 (C**9**, B), 74.0 (C**4**, A), 73.2 (C**4**, B), 66.9 (Cbz CH_2, B), 66.5 (Cbz CH_2, A), 48.8 (C**1**, B), 48.0 (C**1**, A), 45.1 (C**3**, B), 43.7 (C**3**, A), 39.7 (C**6**, B), 39.4 (C**5**, B), 37.8 (C**6**, A), 37.4 (C**5**, A), 22.9 (C**2**, A), 22.7 (C**2**, B); *m/z* (ESI^+) HRMS: Calculated for $C_{18}H_{20}NO_3$: 298.1438. Found $[M+H]^+$: 298.1428.

Signal broadening was observed due to amide-like resonance.

*Note - the regioisomer assignment is based upon the retention of the signal corresponding to C**9** on **4f** (^{1}H δ 6.22 and ^{13}C δ 111.4 in starting material **3f**, translating to ^{1}H δ 6.22 – 6.20 and ^{13}C δ 108.2 in product **4f**.) This is further backed up by the disappearance of a ^{1}H signal at C7.*

4-(4-Methoxybenzyl)pent-4-enal

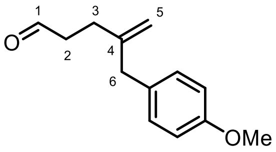

General procedure **G** was followed using 4-(4-methoxybenzyl)pent-4-en-1-ol (911 mg, 4.42 mmol), DMSO (0.94 mL, 13.3 mmol), oxalyl chloride (0.46 mL, 5.30 mmol) and triethylamine (3.10 mL). FCC (10% EtOAc/hexane) gave the title compound (612 mg, 68%) as a colorless oil. ^{1}H NMR (400 MHz, $CDCl_3$) δ 9.71 (1H, t, J = 1.5 Hz, C**1**-H), 7.11 – 7.07 (2H, m, ArCH), 6.85 – 6.81 (2H, m, ArCH), 4.83 (1H, m, C**5**-H), 4.80 (1H, m, C**5**-H'), 3.79 (3H, s, OCH_3), 3.30 (2H, s, C**6**-H), 2.54 (2H, ddd, J = 8.0, 7.0, 2.0 Hz, C**2**-H2), 2.33 – 2.27 (2H, m, C**3**-H2); ^{13}C NMR (101 MHz, $CDCl_3$) δ 202.3 (C**1**), 158.3 (ArC), 147.4 (C**4**), 131.3 (ArC), 130.0 (ArCH), 113.9 (ArCH), 111.7 (C**5**), 55.4 (OCH_3), 42.6 (C**6**), 41.9 (C**2**), 27.5 (C**3**); *m/z* (ESI^+) HRMS: Calculated for $C_{13}H_{17}O_2$: 205.1223. Found $[M+H]^+$: 205.1214.

5-(4-Methoxybenzyl)hex-5-en-2-ol

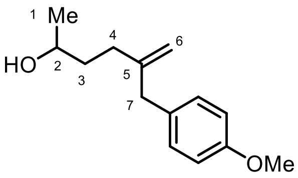

General procedure **H** was followed using the preceding aldehyde (301 mg, 1.47 mmol) and MeMgBr (3.0 M in Et_2O, 0.8 mL). The title compound (284 mg, 87%) was used directly in the next step without further purification. R_f: 0.26 (20% EtOAc/hexane); ν_{max} / cm^{-1}: 3359 (br s), 1610 (m), 1509 (s), 1244 (s); ^{1}H NMR (400 MHz, $CDCl_3$) δ 7.09 (2H, d, J = 8.5 Hz, ArCH), 6.83 (2H, d, J = 8.5 Hz, ArCH), 4.83 (1H, m, C**6**-H), 4.75 (1H, m, C**6**-H'), 3.79 – 3.75 (4H, m, C**2**-H, OCH_3), 3.29 (2H, s, C**7**-H2), 2.13 –

1.97 (2H, m, C**4**-H$_2$), 1.66 – 1.54 (2H, m, C**3**-H$_2$), 1.39 (1H, br s, OH), 1.17 (3H, d, J = 6.0 Hz, C**1**-H$_3$); ^{13}C NMR (101 MHz, $CDCl_3$) δ 158.1 (ArC), 149.3 (C**5**), 131.9 (ArC), 130.0 (ArCH), 113.8 (ArCH), 111.1 (C**6**), 68.0 (C**2**), 55.4 (OCH_3), 42.3 (C**7**), 37.2 (C**3**), 31.7 (C**4**), 23.6 (C**1**); *m/z* (ESI$^+$) HRMS: Calculated for $C_{14}H_{21}O_2$: 221.1536. Found $[M+H]^+$: 221.1525.

Benzyl (5-(4-methoxybenzyl)hex-5-en-2-yl)((perfluorobenzoyl)oxy)carbamate (3h)

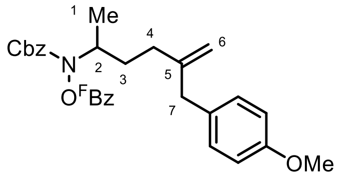

General procedure **I** was followed using CbzNHO^{F}Bz (444 mg, 1.23 mmol), the preceding alcohol (271 mg, 1.23 mmol), triphenylphosphine (358 mg, 1.35 mmol) and diisopropyl azodicarboxylate (0.28 mL, 1.35 mmol). FCC (0 – 2% EtOAc/hexane) gave title compound **3h** (320 mg, 46%) as a colorless oil. R_f: 0.21 (5% EtOAc/hexane); ν_{max} / cm^{-1}: 1785 (m), 1727 (m), 1509 (s), 1326 (m), 764 (s); ^{1}H NMR (400 MHz, $CDCl_3$) δ 7.38 – 7.32 (5H, m, Cbz ArCH), 7.07 – 7.04 (2H, m, ArCH), 6.78 (2H, d, J = 8.5 Hz, ArCH), 5.20 (2H, br s, Cbz CH_2), 4.79 (1H, m, C**6**-H), 4.75 (1H, m, C**6**-H'), 4.34 (1H, m, C**2**-H), 3.77 (3H, s, OCH_3), 3.24 (2H, br s, C**7**-H$_2$), 2.21 – 1.56 (4H, m, C**3**-H$_2$, C**4**-H$_2$), 1.19 (3H, d, J = 7.0 Hz, C**1**-H$_3$); ^{13}C NMR (101 MHz, $CDCl_3$) δ 158.1 (ArC), 148.3 (C**5**), 139.6 (ArC), 135.4 (Cbz ArC), 131.6 (ArC), 130.0, 128.7, 128.6, 128.4, 128.2, 113.8 (5 × Cbz ArCH, 4 × Ph ArCH), 111.7 (C**6**), 68.9 (Cbz CH_2), 56.5 (C**2**), 55.3 (OCH_3), 42.2 (C**7**), 31.8, 31.6 (C**3**, C**4**), 17.5 (C**1**); *mixture of rotamers A + B (96:4)* ^{19}F NMR (400 MHz, $CDCl_3$) δ -135.9 – -136.0 (1.92F, m, A), -137.3 – -137.4 (0.08F, m, B), -146.3 (0.96F, m, A), -148.7 (0.04F, m, B), -159.3 – 159.4 (1.92F, m, A), -160.1 – -160.2 (0.08F, m, B); *m/z* (ESI$^+$) HRMS: Calculated for $C_{29}H_{30}F_5N_2O_5$: 581.2069. Found $[M+NH_4]^+$: 581.2056.

The ^{13}C NMR signals corresponding to the pentafluorobenzoyl group could not be resolved due to their weak intensity.

*Signal broadening was observed due to amide-like resonance resulting in weak signal intensities. The chemical shifts for C**2** and C**6** were assigned using their HMBC correlations.*

Benzyl 5-methoxy-5'-methyl-1,3-dihydrospiro[indene-2,2'-pyrrolidine]-1'-carboxylate (4h)

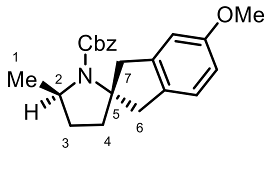

General procedure **J** was followed using cyclization substrate **3h** (56.4 mg, 0.10 mmol), $Pd_2(dba)_3$ (2.29 mg, 0.0025 mmol), CgPPh (4.54 mg, 0.015 mmol), sodium benzoate (1.44 mg, 0.01 mmol), triethylamine (1.40 μL) and dibutyl ether (1 mL). The reaction was stirred at 150 °C for 48 hours. FCC

(0 – 30% CH_2Cl_2/hexane) gave title compound **4h** (19 mg, 53%) as a colorless solid. R_f: 0.20 (30% CH_2Cl_2/hexane); m.p.: 101 – 102 °C; ν_{max} / cm^{-1}: 2924 (m), 1694 (s), 1493(m), 1401 (s), 1347 (s); *mixture of rotamers A + B (2:1)* ^{1}H NMR (500 MHz, $CDCl_3$) δ 7.39 – 7.29 (3H, m, Cbz ArCH, A + B), 7.22 – 7.00 (3H, m, 2 × Cbz ArCH, A + B, 1 × ArCH, A + B), 6.72 – 6.66 (3H, m, ArCH, A + B), 5.14 – 5.04 (2H, m, Cbz CH_2, A + B), 4.16 – 4.13 (1.66H, m, C**2**-H, A + B, C**7**-H A), 3.88 (0.33H, d, *J* = 17.0 Hz, C**7**-H, B), 3.77 (3H, s, OCH_3, A + B), 3.59 (0.66H, d, *J* = 15.0 Hz, C**6**-H, A), 3.36 (0.33H, d, *J* = 15.5 Hz, C**6**-H, B), 2.82 – 2.55 (2H, m, C**6**-H', A, C**6**-H', B, C**7**-H', A, C**7**-H' B), 2.06 – 1.97 (3H, m, C**3**-H, A + B, C**4**-H_2, A + B), 1.54 – 1.53 (1H, m, C**3**-H', A + B), 1.27 – 1.21 (3H, m, C**1**-H_3, A + B); ^{13}C NMR (125 MHz, $CDCl_3$) δ 158.7 (ArC, A + B), 154.7 (Cbz C=O, B), 153.9 (Cbz C=O, A), 143.5 (ArC, A), 143.2 (ArC, B), 137.4 (Cbz ArC, A), 136.7 (Cbz ArC, B), 133.5 (ArC, A), 133.2 (ArC, B), 128.6, 128.4, 127.9, 127.7 (5 × Cbz ArCH, A + B), 125.2 (ArCH, A), 124.9 (ArCH, B), 112.5 (ArCH, B), 112.2 (ArCH, A), 109.9 (ArCH, A), 109.6 (ArCH, B), 71.1 (C**5**, A), 70.0 (C**5**, B), 66.6 (Cbz CH_2, B), 66.3 (Cbz CH_2, A), 55.9 (C**2**, B), 55.4 (OCH_3, A + B), 55.0 (C**2**, A), 46.4 (C**7**, B), 44.6 (C**7**, A), 41.9 (C**6**, A + B), 41.6 (C**4**, B), 39.7 (C**4**, A), 29.7 (C**3**, A), 29.4 (C**3**, B), 21.2 (C**1**, A), 20.3 (C**1**, B); *m/z* (ESI^+) HRMS: Calculated for $C_{22}H_{26}NO_3$: 352.1907. Found $[M+H]^+$: 352.1901.

Significant signal broadening was observed due to amide-like resonance.

Note – the relative stereochemistry of ***4h*** *is assigned using nOe analysis after Cbz deprotection. The hydrogenative removal of the Cbz group on* ***4h*** *was carried out using the following procedure:*

5-Methoxy-5'-methyl-1,3-dihydrospiro[indene-2,2'-pyrrolidine]

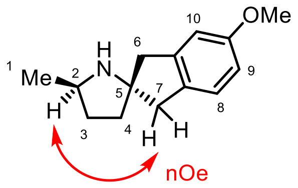

To a solution of **4h** (7.1 mg, 0.02 mmol) in EtOAc (1 mL) was added 5% Pd/C (2 mg). Hydrogen gas was bubbled through the solution for 2 minutes. The reaction was stirred vigorously under a balloon of hydrogen for 4 hours. Upon completion, the reaction was filtered through celite, washing with CH_2Cl_2/MeOH 4:1 (20 mL). The filtrate was concentrated thoroughly *in vacuo* to give the title compound (3.8 mg, 87%) as a colorless oil. ν_{max} / cm^{-1}: 3316 (br s), 2954 (s), 2928 (s), 1610 (s), 1491 (s), 1263 (s), 1034 (s); ^{1}H NMR (400 MHz, $CDCl_3$) δ 7.05 (1H, d, *J* = 8.0 Hz, C**8**-H), 6.73 (1H, m, C**10**-H), 6.68 (1H, dd, *J* = 8.0, 2.0 Hz, C**9**-H), 3.76 (3H, s, OCH_3), 3.41 (1H, m, C**2**-H), 3.11 – 2.97 (2H, m, C**6**-H_2), 2.99 – 2.86 (2H, m, C**7**-H_2), 2.05 (1H, m, C**3**-H), 1.91 – 1.82 (2H, m, C**4**-H_2), 1.50 (1H, m, C**3**-H'), 1.23 (3H, d, *J* = 6.5 Hz, C**1**-H_3); ^{13}C NMR (101 MHz, $CDCl_3$) δ 158.9 (ArC OCH_3), 143.4 (ArC), 133.8 (ArC), 125.4 (ArCH), 112.4 (ArCH), 110.2 (ArCH), 71.9 (C**5**), 55.5 (OCH_3), 53.8 (C**2**), 46.9

(C**6**), 45.6 (C**7**), 38.1 (C**4**), 33.2 (C**3**), 21.7 (C**1**); *m/z* (ESI^+) HRMS: Calculated for $C_{14}H_{20}NO$: 218.1539. Found $[M+H]^+$: 218.1549.

*The relative stereochemistry is confirmed by the observation of an nOe effect between C**2**-H and C**7**-H_2.*

4-(4-Methoxybenzyl)-1-phenylpent-4-en-1-ol

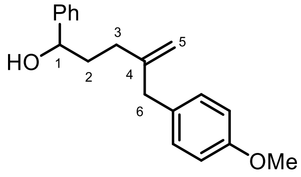

General procedure **H** was followed using 4-(4-methoxybenzyl)pent-4-enal (301 mg, 1.47 mmol) and PhMgBr (1.0 M in THF, 2.4 mL). The title compound (446 mg, quantitative) was used directly in the next step without further purification. R_f: 0.31 (20% EtOAc/hexane); ν_{max} / cm^{-1}: 3381 (br s), 1643 (m), 1509 (s), 1260 (s); ^1H NMR (400 MHz, $CDCl_3$) δ 7.37 – 7.28 (5H, m, ArCH), 7.07 (2H, d, *J* = 9.0 Hz, ArCH), 6.82 (2H, d, *J* = 9.0 Hz, ArCH), 4.83 (1H, m, C**5**-H), 4.76 (1H, m, C**5**-H'), 4.66 – 4.62 (1H, m, C**1**-H), 3.79 (3H, s, OCH_3), 3.28 (2H, s, C**6**-H_2), 2.16 – 1.80 (5H, m, C**2**-H_2, C**3**-H_2, OH); ^{13}C NMR (101 MHz, $CDCl_3$) δ 158.1 (ArC), 149.0 (C**4**), 144.8 (ArC), 131.8 (ArC), 130.0, 128.6, 127.7, 126.0, 113.8 (ArCH), 111.3 (C**5**), 74.3 (C**1**), 55.3 (OCH_3), 42.3 (C**6**), 37.0 (C**2**), 31.7 (C**3**); *m/z* ($APCI^+$) HRMS: Calculated for $C_{19}H_{22}O$: 265.1587. Found $[M+H–H_2O]^+$: 265.1579.

Benzyl (4-(4-methoxybenzyl)-1-phenylpent-4-en-1-yl)((perfluorobenzoyl)oxy)carbamate (3i)

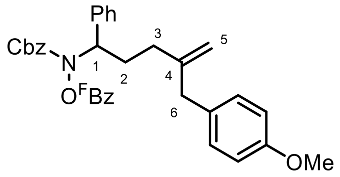

General procedure **I** was followed using $CbzNHO^FBz$ (554 mg, 1.53 mmol), the preceding alcohol (433 mg, 1.53 mmol), triphenylphosphine (447 mg, 1.69 mmol) and diisopropyl azodicarboxylate (0.35 mL, 1.69 mmol). FCC (0 – 2% EtOAc/hexane) gave title compound **3i** (525 mg, 55%) as a colorless oil. R_f: 0.21 (5% EtOAc/hexane); ν_{max} / cm^{-1}: 1786 (m), 1729 (m), 1652 (m), 1524 (m), 1509 (s), 1276 (s), 764 (s); ^1H NMR (400 MHz, $CDCl_3$) δ 7.36 – 7.18 (10H, m, 5 × Cbz ArCH + 5 × ArCH), 7.04 (2H, d, *J* = 8.0 Hz, ArCH), 6.82 – 6.76 (2H, m, ArCH), 5.31 – 5.07 (3H, m, Cbz CH_2, C**1**-H), 4.81 (1H, m, C**5**-H), 4.78 (1H, m, C**5**-H'), 3.76 (3H, s, OCH_3), 3.25 (2H, s, C**6**-H_2), 2.24 – 1.91 (4H, m, C**2**-H_2, C**3**-H_2); ^{13}C NMR (101 MHz, $CDCl_3$) δ 158.1 (ArC), 148.0 (C**4**), 138.1 (ArC), 135.2 (Cbz ArC), 131.5 (ArC), 130.0, 129.2, 128.7, 128.6, 128.4, 128.2, 113.8 (ArCH), 112.1 (C**5**), 69.0 (Cbz CH_2), 63.6 (C**1**), 55.3 (OCH_3), 42.2 (C**6**), 31.9 (C**2**, C**3**); *mixture of rotamers A + B (9:1)* ^{19}F NMR (400 MHz, $CDCl_3$) δ -135.6 – -136.0 (1.8F, m, A), -137.3 (0.2F, d, *J* = 19.0 Hz, B), -146.2 (0.9F, br t, *J* = 21.0 Hz, A), -148.8 (0.1F, t,

J = 21.0 Hz, B), -159.1 – -159.3 (1.8F, m, A), -160.1 – -160.3 (0.2F, m, B); *m/z* (ESI^+) HRMS: Calculated for $C_{34}H_{32}F_5N_2O_5$: 643.2226. Found $[M+NH_4]^+$: 643.2209.

The ^{13}C NMR signals corresponding to the pentafluorobenzoyl group and the carbonyl of the Cbz could not be resolved due to their weak intensity.

*Signal broadening was observed due to amide-like resonance resulting in weak signal intensities. The chemical shifts for C**1**, C**5** and the ArC̲ signal at 138.1 ppm were assigned using their HSQC and HMBC correlations.*

Benzyl 5-methoxy-5'-phenyl-1,3-dihydrospiro[indene-2,2'-pyrrolidine]-1'-carboxylate (4i)

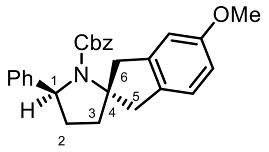

General procedure **J** was followed using cyclization substrate **3i** (62.6 mg, 0.100 mmol), $Pd_2(dba)_3$ (4.62 mg, 0.005 mmol), CgPPh (12.2 mg, 0.040 mmol), sodium benzoate (1.44 mg, 0.01 mmol), triethylamine (1.40 μL) and dibutyl ether (1 mL). The reaction was stirred at 150 °C for 48 hours. FCC (toluene) gave title compound **4i** (18.2 mg, 44%) as a colorless solid. R_f: 0.18 (toluene); m.p.: 128 – 130 °C (CH_2Cl_2/hexane); ν_{max} / cm^{-1}: 2925 (m), 1698 (s), 1493 (m), 1401 (m), 1343 (s); *mixture of rotamers A + B (2:1)* ^{1}H NMR (500 MHz, $CDCl_3$) δ 7.35 – 7.16 (8H, m, 4 × Cbz ArCH̲, A + B, 4 × ArCH̲, A + B), 7.06 – 7.05 (2H, m, ArCH̲, A + B), 6.93 – 6.88 (1H, m, Cbz ArCH̲, A + B), 6.77 – 6.69 (2H, m, ArCH̲, A + B), 5.23 – 4.94 (3H, m, Cbz CH̲$_2$, A + B, C**1**-H̲, A + B), 4.38 (0.66H, d, *J* = 15.0 Hz, C**6**-H̲, A), 4.14 (0.33H, d, *J* = 15.5 Hz, C**6**-H̲, B), 3.78 – 3.72 (3.66H, m, OCH_3, A + B, C**5**-H̲, A), 3.50 (0.33H, d, *J* = 15.0 Hz, C**5**-H̲, B), 3.04 (0.33H, d, *J* = 15.5 Hz, C**6**-H̲', B), 2.90 (0.66H, d, *J* = 15.0 Hz, C**6**-H̲', A), 2.83 (0.33H, d, *J* = 15.0 Hz, C**5**-H̲', B), 2.70 (0.66H, d, *J* = 15.0 Hz, C**5**-H̲', A), 2.41 – 2.34 (1H, m, C**2**-H̲, A + B), 2.04 – 1.93 (2H, m, C**3**-H̲$_2$, A + B), 1.84 – 1.81 (1H, m, C**2**-H̲', A + B); ^{13}C NMR (125 MHz, $CDCl_3$) δ 158.9 (ArC̲, B), 158.8 (ArC̲, A), 154.0 (Cbz C̲=O, A + B), 144.3 (ArC̲, A), 143.4 (ArC̲, B), 143.2 (ArC̲, A), 143.0 (ArC̲, B), 137.0 (Cbz ArC̲, A), 136.6 (Cbz ArC̲, B), 133.4 (ArC̲, A), 133.1 (ArC̲, B), 128.8, 128.5, 128.3, 127.8, 127.6, 127.3, 126.8, 126.5, 125.6, 125.2 (5 × Cbz ArC̲H A + B, 6 × ArC̲H A + B), 112.6 (ArC̲H, B), 112.3 (ArC̲H, A), 110.1 (ArC̲H, A), 109.8 (ArC̲H, B), 71.7 (C**4**, A), 70.6 (C**4**, B), 66.9 (Cbz C̲H$_2$, B), 66.2 (Cbz C̲H$_2$, A), 63.5 (C**1**, B), 63.0 (C**1**, A), 55.5 (OC̲H$_3$, A), 55.4 (OC̲H$_3$, B), 45.7 (C**6**, B), 44.7 (C**5**, B), 43.7 (C**6**, A), 42.0 (C**5**, A), 40.8 (C**3**, B), 39.0 (C**3**, A), 32.3 (C**2**, A), 31.9 (C**2**, B); *m/z* (ESI^+) HRMS: Calculated for $C_{27}H_{28}NO_3$: 414.2064. Found $[M+H]^+$: 414.2064.

Significant signal broadening was observed due to amide-like resonance.

*Note – the relative stereochemistry was assigned by analogy to **4h**.*

Total Synthesis of (+)-Pileamartine A

Experimental Procedures and Data

((Hex-5-en-1-yloxy)methyl)benzene

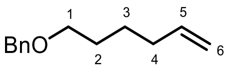

To a suspension of NaH 60% dispersion in mineral oil (1.37 g, 57.0 mmol) in anhydrous THF (60 mL) at 0 °C, was added 5-hexen-1-ol (3.00 g, 30.0 mmol) as a solution in anhydrous THF (5 mL). Benzyl bromide (4.28 mL, 36.0 mmol) was then added *via* syringe, and the reaction was stirred at room temperature for 16 hours. Upon completion, the reaction was quenched with sat. NH_4Cl solution (150 mL), and the aqueous phase extracted with Et_2O (3 × 100 mL). The combined organic extracts were washed with brine (100 mL), dried (Na_2SO_4) and concentrated *in vacuo*. FCC (0 – 5% EtOAc/hexane gave the title compound (4.90 g, 86%) as a colorless oil. ^{1}H NMR (400 MHz, $CDCl_3$) δ 7.38 – 7.27 (5H, m, ArC<u>H</u>), 5.81 (1H, ddt, *J* = 17.0, 10.0, 6.5 Hz, C**5**-<u>H</u>), 5.00 (1H, m, C**6**-<u>H</u>), 4.95 (1H, m, C**6**-<u>H</u>'), 4.51 (2H, s, Bn C<u>H$_2$</u>), 3.48 (2H, t, *J* = 6.5 Hz, C**1**-<u>H$_2$</u>), 2.11 – 2.04 (2H, m, C**4**-<u>H$_2$</u>), 1.69 – 1.59 (2H, m, C**2**-<u>H$_2$</u>), 1.53 – 1.44 (2H, m, C**3**-<u>H$_2$</u>); ^{13}C NMR (101 MHz, $CDCl_3$) δ 138.9 (C**5**), 138.8 (Ar<u>C</u>), 128.5 (Ar<u>C</u>H), 127.8 (Ar<u>C</u>H), 127.6 (Ar<u>C</u>H), 114.6 (C**6**), 73.0 (Cbz <u>C</u>H$_2$), 70.4 (C**1**), 33.7 (C**4**), 29.4 (C**2**), 25.7 (C**3**).

The spectroscopic properties were consistent with the data available in the literature.[43]

2-(4-(Benzyloxy)butyl)oxirane (*rac*-5)

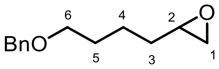

To a solution of the preceding alkene (4.88 g, 25.6 mmol) in anhydrous CH_2Cl_2 (73 mL) at 0 °C was added *m*-CPBA (7.51 g, 43.5 mmol). The solution was stirred at room temperature for 16 hours. Upon completion, the reaction was quenched with sat. $NaHCO_3$ solution (40 mL) and sat. Na_2SO_3 solution (100 mL). The aqueous phase was extracted with CH_2Cl_2 (3 × 100 mL), and the combined extracts were washed with brine (100 mL), dried (Na_2SO_4) and concentrated *in vacuo*. FCC (5 – 10% EtOAc/hexane) gave title compound ***rac*-5** (4.95 g, 94%) as a colorless oil. ^{1}H NMR (400 MHz, $CDCl_3$) δ 7.38 – 7.27 (5H, m, ArC<u>H</u>), 4.51 (2H, s, Bn C<u>H$_2$</u>), 3.49 (2H, t, *J* = 6.5 Hz, C**6**-<u>H$_2$</u>), 2.92 (1H, m, C**2**-<u>H</u>), 2.74 (1H, dd, *J* = 5.0, 4.0 Hz, C**1**-<u>H</u>), 2.46 (1H, dd, *J* = 5.0, 2.7 Hz, C**1**-<u>H</u>'), 1.72 – 1.63 (2H, m, C**5**-<u>H$_2$</u>), 1.58 – 1.53 (4H, m, C**3**-<u>H$_2$</u> + C**4**-<u>H$_2$</u>); ^{13}C NMR (101 MHz, $CDCl_3$) δ 138.7 (Ar<u>C</u>), 128.5 (Ar<u>C</u>H), 127.8 (Ar<u>C</u>H), 127.7 (Ar<u>C</u>H), 73.1 (Bn <u>C</u>H$_2$), 70.3 (C**6**), 52.4 (C**2**), 47.2 (C**1**), 32.4 (C**3**), 29.7 (C**5**), 22.9 (C**4**).

The spectroscopic properties were consistent with the data available in the literature.[44]

(*R*)-2-(4-(Benzyloxy)butyl)oxirane (5)

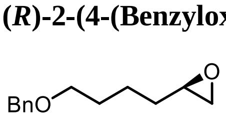

To a solution of ***rac*-5** (3.15 g, 15.3 mmol) and (*R*,*R*)-*N*,*N'*-bis(3,5-di-tert-butylsalicylidene)-1,2-cyclohexanediaminocobalt(II) (45.9 mg, 0.076 mmol) in THF (0.5 mL) open to air, was added acetic acid (17.5 μL, 0.31 mmol). The mixture was subsequently cooled to 0 °C, and water (151 μL, 8.42 mmol) was added slowly. The reaction was stirred at room temperature for 48 hours. Upon completion, the reaction was concentrated *in vacuo*. FCC (5 – 10% EtOAc/hexane) gave title compound **5** (1.56 g, 49%, e.e. > 99%) as a red oil. $[\alpha]^{24}_{D}$ = + 4.4 (c = 1.025, $CHCl_3$).

The spectroscopic properties were consistent with the data available in the literature.[44]

Chiral SFC: (DAICEL CHIRALPAK-IC column (25 cm), CO_2:IPA 97.5:2.5, 1.5 mL/min, 140 bars, 40 °C). Retention times: 15.7 minutes (major), 16.5 minutes (minor), e.e. > 99%.

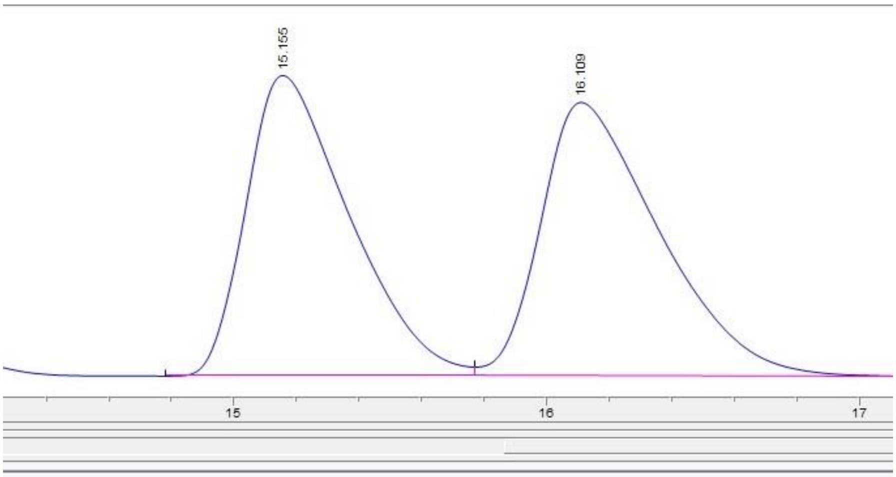

#	Time	Area	Height	Width	Area%	Symmetry
1	15.155	17867.2	777.4	0.3401	48.840	0.527
2	16.109	18715.8	708.4	0.4002	51.160	0.497

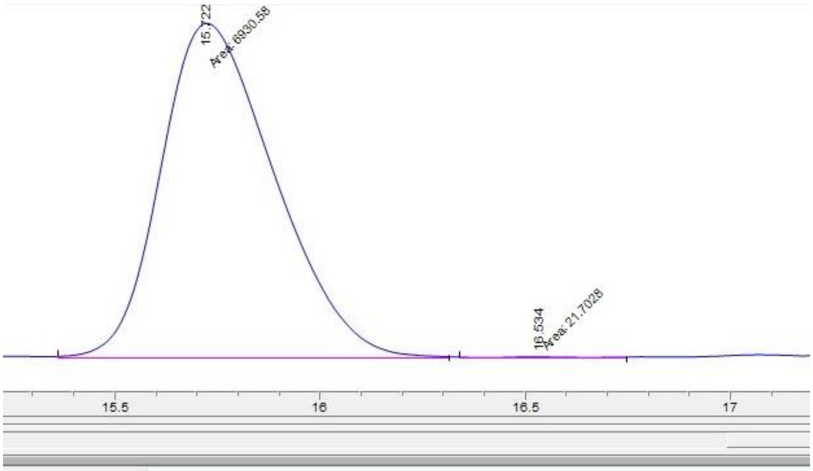

#	Time	Area	Height	Width	Area%	Symmetry
1	15.722	6930.6	358.9	0.3218	99.688	0.671
2	16.534	21.7	1.3	0.2886	0.312	0.884

Isopropyl 2-(3,4-dimethoxyphenyl)acetate (6)

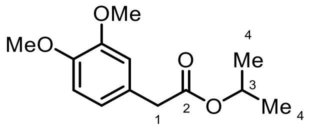

A solution of 3,4-dimethoxyphenylacetic acid (3.24 g, 20 mmol) and H_2SO_4 (0.213 mL, 4.00 mmol) in MeOH (50 mL) was heated at reflux (80 °C) for 24 hours. Upon completion, the reaction was cooled to room temperature, and the solvent was removed *in vacuo*. The residue was diluted with water (100 mL) and extracted with CH_2Cl_2 (3 × 50 mL). The combined organic layers were washed with brine (50 mL), dried (Na_2SO_4) and concentrated *in vacuo*. The crude residue was redissolved in IPA (80 mL), KOH (223 mg, 4.00 mmol) was added, and the reaction was stirred at 80 °C for 5 hours. Upon completion, sat. NH_4Cl solution (100 mL) was added, and the mixture was extracted with EtOAc (3 × 50 mL). The combined organic layers were washed with brine (100 mL), dried (Na_2SO_4) and concentrated *in vacuo*. FCC (20% EtOAc/hexane) gave title compound **6** (4.16 g, 88%) as a colorless oil. ^{1}H NMR (400 MHz, $CDCl_3$) δ 6.83 – 6.80 (3H, m, ArCH), 5.01 (1H, hept, C**3**-H), 3.88 (3H, s, OCH$_3$), 3.86 (3H, s, OCH$_3$), 3.51 (1H, s, C**1**-H$_2$), 1.23 (6H, d, *J* = 6.0 Hz, 2 × C**4**-H$_3$); ^{13}C NMR (101 MHz, $CDCl_3$) δ 171.5 (C**1**), 149.0 (ArC), 148.2 (ArC), 127.0 (ArC), 121.5 (ArCH), 112.5 (ArCH), 111.4 (ArCH), 68.3 (C**3**), 56.0 (OCH$_3$), 56.0 (OCH$_3$), 41.4 (C**1**), 21.9 (C**4**).

The spectroscopic properties were consistent with the data available in the literature.[45]

(3*R*,5*R*)-5-(4-(Benzyloxy)butyl)-3-(3,4-dimethoxyphenyl)dihydrofuran-2(3*H*)-one (*cis*-7)

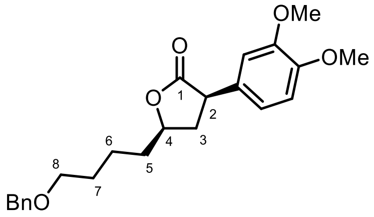

To a solution of NaHMDS (2M in THF, 0.30 mL) in a flame-dried Schlenk tube cooled to 0 °C, was added DMPU (0.37 mL, 3.00 mmol) *via* syringe. Next, a solution of **6** (131 mg, 0.55 mmol) in anhydrous DMF (10 mL) was added dropwise *via* syringe, and the reaction was stirred for 15 minutes. A solution of **5** (103 mg, 0.50 mmol) in anhydrous DMF (3 mL) was then added *via* syringe. The reaction was sealed and stirred at 60 °C for 24 hours. Upon completion, the reaction was quenched with sat. NH_4Cl solution (20 mL) and the aqueous layer was extracted with EtOAc (3 × 20 mL). The combined organic layers were washed with water (5 × 20 mL), brine (50 mL), dried (Na_2SO_4) and concentrated *in vacuo*. FCC (80% Et_2O/ hexane) gave title compound ***cis*-7** (117 mg, 61%) and ***trans*-7** (42 mg, 22%) as pale yellow solids. *Data for **cis-7**:* R_f: 0.38 (80% Et_2O/hexane); m.p.: 71 – 73 °C (CH_2Cl_2/hexane); ν_{max} / cm^{-1}: 2937 (s), 2863 (s), 1766 (s), 1518 (s), 1263 (s), 1174 (s), 1144 (s), 1027 (s); ^{1}H NMR (400 MHz, $CDCl_3$) δ 7.35 – 7.27 (5H, m, Bn ArCH), 6.86 – 6.79 (3H, m, ArCH), 4.51

(2H, s, Bn C<u>H</u>$_2$), 4.50 – 4.43 (1H, m, C**4**-<u>H</u>), 3.88 (3H, s, OC<u>H</u>$_3$), 3.87 (3H, s, OC<u>H</u>$_3$), 3.86 – 3.79 (1H, m, C**2**-<u>H</u>), 3.50 (2H, t, *J* = 6.0 Hz, C**8**-<u>H</u>$_2$), 2.73 (1H, ddd, *J* = 12.5, 8.5, 5.5 Hz, C**3**-<u>H</u>), 2.01 (1H, ddd, 12.5, 12.5, 10.5 Hz, C**3**-<u>H</u>'), 1.91 – 1.81 (1H, m, C**5**-<u>H</u>), 1.76 – 1.65 (3H, m, C**5**-<u>H</u>', C**7**-<u>H</u>$_2$), 1.63 – 1.54 (2H, m, C**6**-<u>H</u>$_2$); ^{13}C NMR (101 MHz, $CDCl_3$) δ 177.1 (C**1**), 149.3 (Ar<u>C</u>OCH$_3$), 148.7 (Ar<u>C</u>OCH$_3$), 138.6 (Bn Ar<u>C</u>), 129.1 (Ar<u>C</u>), 128.5 (Bn Ar<u>C</u>H), 127.8 (Bn Ar<u>C</u>H), 127.7 (Bn Ar<u>C</u>H), 120.4 (Ar<u>C</u>H), 111.6 (Ar<u>C</u>H), 111.4 (Ar<u>C</u>H), 78.6 (C**4**), 73.1 (Bn <u>C</u>H$_2$), 70.1 (C**8**), 56.1 (2 × O<u>C</u>H$_3$), 47.0 (C**2**), 38.2 (C**3**), 35.3 (C**5**), 29.6 (C**7**), 22.3 (C**6**); *m/z* (ESI$^+$) HRMS: Calculated for $C_{23}H_{29}O_5$: 385.2032. Found [M+H]$^+$: 385.2030; $[\alpha]^{25}_D$ = + 6.42 (c = 0.55, $CHCl_3$).

*Data for **trans-7**:* R$_f$: 0.42 (80% Et_2O/hexane); m.p.: 41 – 42 °C (CH_2Cl_2/hexane); ν_{max} / cm^{-1}: 2938 (m), 2863 (m), 1767 (s), 1517 (s), 1252 (s), 1145 (s), 1027 (s); ^{1}H NMR (400 MHz, $CDCl_3$) δ 7.36 – 7.26 (5H, m, Bn ArC<u>H</u>), 7.86 – 7.78 (3H, m, ArC<u>H</u>), 4.62 (1H, m, C**4**-<u>H</u>), 4.51 (2H, s, Bn C<u>H</u>$_2$), 3.88 (3H, s, OC<u>H</u>$_3$), 3.87 (3H, s, OC<u>H</u>$_3$), 3.84 (1H, dd, *J* = 9.5, 7.0 Hz, C**2**-<u>H</u>), 3.50 (2H, t, *J* = 6.0 Hz, C**8**-<u>H</u>$_2$), 2.48 (1H, m, C**3**-<u>H</u>), 2.36 (1H, ddd, *J* = 13.0, 9.5, 5.5 Hz, C**3**-<u>H</u>'), 1.86 – 1.78 (1H, m, C**5**-<u>H</u>), 1.72 – 1.51 (5H, m, C**5**-<u>H</u>', C**6**-<u>H</u>$_2$, C**7**-<u>H</u>$_2$); ^{13}C NMR (101 MHz, $CDCl_3$) δ 177.5 (C**1**), 149.5 (Ar<u>C</u>OCH$_3$), 148.7 (Ar<u>C</u>OCH$_3$), 138.6 (Bn Ar<u>C</u>), 129.6 (Ar<u>C</u>), 128.5 (Bn Ar<u>C</u>H), 127.8 (Bn Ar<u>C</u>H), 127.7 (Bn Ar<u>C</u>H), 119.7 (Ar<u>C</u>H), 111.6 (Ar<u>C</u>H), 111.2 (Ar<u>C</u>H), 79.0 (C**4**), 73.1 (Bn <u>C</u>H$_2$), 70.1 (C**8**), 56.1 (2 × O<u>C</u>H$_3$), 45.3 (C**2**), 36.5 (C**3**), 35.4 (C**5**), 29.6 (C**7**), 22.4 (C**6**); *m/z* (ESI$^+$) HRMS: Calculated for $C_{23}H_{29}O_5$: 385.2032. Found [M+H]$^+$: 385.2030; $[\alpha]^{25}_D$ = + 22.4 (c = 0.39, $CHCl_3$).

*The relative and absolute stereochemistry were confirmed by single crystal X-ray analysis of derivative **8**.*

(3*R*,5*R*)-3-(3,4-Dimethoxyphenyl)-5-(4-hydroxybutyl)dihydrofuran-2(3H)-one

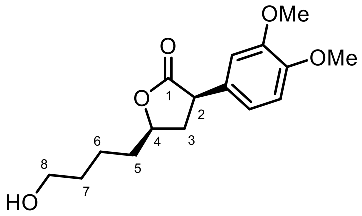

To a solution of ***cis*-7** (385 mg, 1.00 mmol) in EtOAc (10 mL) was added 5% Pd/C (38.5 mg). Hydrogen gas was bubbled through the solution for 2 minutes and the reaction was stirred vigorously under the balloon of hydrogen for 20 hours. Upon completion, the reaction was filtered through celite, washing with EtOAc (30 mL), and the filtrate was concentrated *in vacuo*. FCC (90% EtOAc/hexane) gave the title compound (233 mg, 79%) as a colorless solid. R$_f$: 0.33 (90% EtOAc/hexane); m.p.: 114 – 115 °C (EtOAc/hexane); ν_{max} / cm^{-1}: 3508 (br s), 2937 (s), 1762 (s), 1518 (s), 1269 (s), 1175 (s), 1144 (s), 1025 (s); ^{1}H NMR (400 MHz, $CDCl_3$) δ 6.87 – 6.79 (3H, m, ArC<u>H</u>), 4.48 (1H, dddd, *J* = 10.5, 7.5, 5.5, 5.5 Hz, C**4**-<u>H</u>), 3.90 – 3.80 (1H, m, C**2**-<u>H</u>), 3.89 (3H, s, OC<u>H</u>$_3$), 3.87 (3H, s, OC<u>H</u>$_3$), 3.71 – 3.66 (2H, m,

C**8**-H_2), 2.75 (1H, ddd, *J* = 12.5, 8.5, 5.5 Hz, C**3**-H), 2.03 (1H, ddd, *J* = 12.5, 12.5, 10.5 Hz, C**3**-H'), 1.92 – 1.83 (1H, m, C**5**-H), 1.80 – 1.70 (1H, m, C**5**-H'), 1.68 – 1.57 (4H, m, C**6**-H_2, C**7**-H_2); ^{13}C NMR (101 MHz, $CDCl_3$) δ 177.1 (C**1**), 149.4 ($ArCOCH_3$), 148.7 ($ArCOCH_3$), 129.1 (ArC), 120.4 (ArCH), 111.6 (ArCH), 111.5 (ArCH), 78.6 (C**4**), 62.7 (C**8**), 56.1 (2 × OCH_3), 46.9 (C**2**), 38.3 (C**3**), 35.3 (C**5**), 32.5 (C**7**), 21.9 (C**6**); *m/z* (ESI^+) HRMS: Calculated for $C_{16}H_{22}NaO_5$: 317.1359. Found $[M+Na]^+$: 317.1373; $[\alpha]^{21}_D$ = + 6.73 (c = 0.45, $CHCl_3$).

4-((2*R*,4*R*)-4-(3,4-Dimethoxyphenyl)-5-oxotetrahydrofuran-2-yl)butyl 4-bromobenzoate (8)

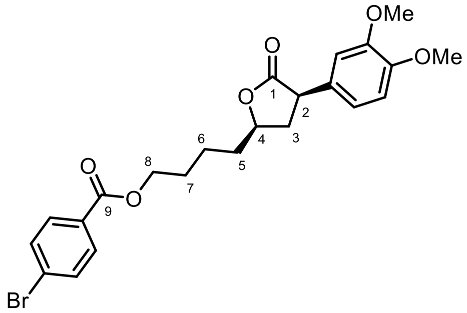

To a solution of the preceding alcohol (118 mg, 0.40 mmol) and 4-bromobenzoyl chloride (105 mg, 0.48 mmol) in CH_2Cl_2 (3 mL) at 0 °C, was added NEt_3 (84 µL). The reaction was allowed to warm to room temperature and stirred for 16 hours. Upon completion, sat. $NaHCO_3$ solution (10 mL) and water (10 mL) were added and the aqueous phase was extracted with CH_2Cl_2 (3 × 20 mL). The combined organic layers were washed with brine (20 mL), dried (Na_2SO_4) and concentrated *in vacuo*. FCC (40% EtOAc/hexane) gave title compound **8** (117 mg, 61%) as a colorless solid. R_f: 0.41 (50% EtOAc/hexane); m.p.: 81 – 82 °C (Et_2O); ν_{max} / cm^{-1}: 2938 (m), 1765 (s), 1715 (s), 1518 (s), 1265 (s); ^{1}H NMR (400 MHz, $CDCl_3$) δ 7.90 – 7.87 (2H, m, ArCH), 7.59 – 7.55 (2H, m, ArCH), 6.85 – 6.78 (3H, m, ArCH), 4.48 (1H, dddd, *J* = 10.5, 8.0, 5.0, 5.0 Hz, C**4**-H), 4.34 (2H, t, *J* = 6.5 Hz, C**8**-H_2), 3.87 (3H, s, OCH_3), 3.86 (3H, s, OCH_3), 3.82 (1H, dd, *J* = 13.0, 9.0 Hz, C**2**-H), 2.74 (1H, ddd, *J* = 12.5, 8.5, 5.5 Hz, C**3**-H), 2.02 (1H, ddd, *J* = 12.5, 12.5, 10.5 Hz, C**3**-H'), 1.90 – 1.56 (6H, m, C**5**-H_2, C**6**-H_2, C**7**-H_2); ^{13}C NMR (101 MHz, $CDCl_3$) δ 176.9 (C**1**), 166.0 (C**9**), 149.3 ($ArCOCH_3$), 148.7 ($ArCOCH_3$), 131.8 (ArCH), 131.2 (ArCH), 129.3 (ArC), 129.0 (ArC), 128.2 (ArC), 120.3 (ArCH), 111.6 (ArCH), 111.4 (ArCH), 78.3 (C**4**), 64.9 (C**8**), 56.1 (2 × OCH_3), 46.8 (C**2**), 38.2 (C**3**), 35.2 (C**5**), 28.6 (C**7**), 22.2 (C**6**); *m/z* (ESI^+) HRMS: Calculated for $C_{23}H_{25}BrNaO_6$: 499.0727. Found $[M+Na]^+$: 499.0708; $[\alpha]^{21}_D$ = – 5.53 (c = 0.72, $CHCl_3$).

The relative and absolute stereochemistry were confirmed by single crystal X-ray analysis after recrystallization (benzene/Et_2O).

Single Crystal X-ray Analysis of 8

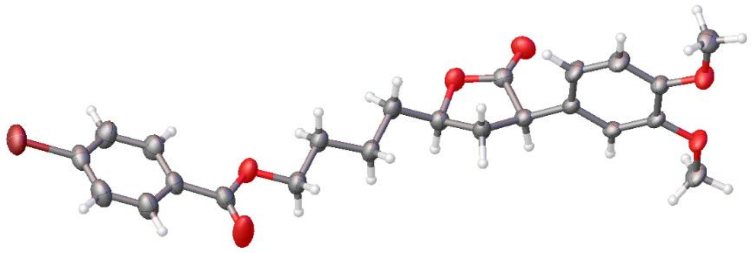

(3*R*,5*R*)-9-(Benzyloxy)-3-(3,4-dimethoxyphenyl)-2-(4-methoxyphenyl)non-1-en-5-ol (11)

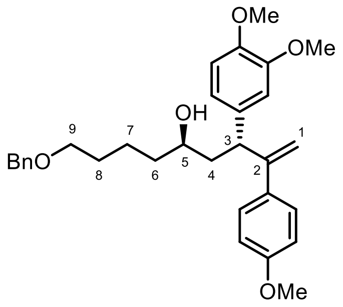

A solution of ***cis*-7** (76.8 mg, 0.20 mmol) and $LaCl_3{\cdot}2LiCl$ (0.6M in THF, 0.36 mL) in anhydrous THF (2 mL) under argon was stirred at room temperature for 1 hour. The solution was then cooled to -15 °C (acetone/dry ice bath), and 4-methoxyphenylmagnesium bromide (0.5M in THF, 0.48 mL) was added dropwise *via* syringe. The reaction as stirred at -15 °C for 1 hour until complete consumption of ***cis*-7** was observed by TLC. The Tebbe reagent (0.5M in toluene, 1.6 mL) was then added *via* syringe. The reaction was allowed to warm to room temperature and subsequently heated to 50 °C for 1 hour. Upon completion, the reaction was cooled to 0 °C and quenched sequentially with 2M aq. NaOH solution (3 drops), sat. $NaHCO_3$ solution (10 drops), water (1 mL), diluted with toluene (5 mL), and stirred vigorously at 0 °C for 10 minutes. The mixture was then filtered through celite whilst cold, rinsing with toluene, and concentrated *in vacuo*. FCC (40 – 50% EtOAc/hexane) gave title compound **11** (59.1 mg, 60%) as a colorless oil. *Note – $CDCl_3$ must be filtered through a plug of K_2CO_3 directly before use as an NMR solvent to avoid acid mediated cyclization of the OH group onto the electron rich styrene.* R_f: 0.13 (30% EtOAc/hexane); ν_{max} / cm^{-1}: 3434 (br s), 2936 (s), 2862 (s), 1510 (s), 1248 (s), 1032 (s); ^1H NMR (400 MHz, $CDCl_3$) δ 7.35 – 7.27 (5H, m, Bn ArCH), 7.25 – 7.22 (2H, m, ArCH), 6.81 – 6.74 (5H, m, ArCH), 5.28 (1H, m, C**1**-H), 5.07 (1H, m, C**1**-H'), 4.47 (2H, s, Bn CH_2), 4.07 (1H, dd *J* = 10.0, 5.0 Hz, C**3**-H), 3.84 (3H, s, OCH_3), 3.82 (3H, s, OCH_3), 3.76 (3H, s, OCH_3), 3.45 – 3.40 (2H, m, C**9**-H_2, C**5**-H), 1.97 – 1.83 (2H, m, C**4**-H_2), 1.61 – 1.52 (2H, m, C**8**-H_2), 1.48 – 1.34 (4H, m, C**6**-H_2, C**7**-H_2); ^{13}C NMR (101 MHz, $CDCl_3$) δ 159.0 ($ArCOCH_3$), 152.2 (C**2**), 149.0 ($ArCOCH_3$), 147.6 ($ArCOCH_3$), 138.7 (Bn ArC), 135.4 (ArC), 134.9 (ArC), 128.5 (ArCH), 128.0 (ArCH), 127.8 (ArCH), 127.7 (ArCH), 120.6 (ArCH), 113.6 (ArCH), 111.7 (ArCH), 111.6 (C**1**), 111.1 (ArCH), 73.0 (Bn CH_2), 70.3 (C**9**), 69.6 (C**5**), 56.0 (OCH_3), 55.9 (OCH_3), 55.3 (OCH_3), 46.0 (C**3**), 42.6 (C**4**), 38.2 (C**7**), 29.8 (C**8**), 22.4 (C**6**);

m/z (ESI^+) HRMS: Calculated for $C_{31}H_{39}O_5$: 491.2792. Found $[M+H]^+$: 491.2772; $[\alpha]^{27}_D$ = – 24.4 (c = 0.43, $CHCl_3$).

Tris(3,4,5-trifluorophenyl)phosphane

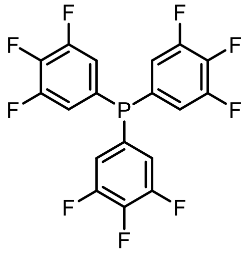

This compound was prepared according to a previously reported literature procedure.[46]

The spectroscopic properties were consistent with the data available in the literature.[46]

Benzyl ((3*R*,5*S*)-9-(benzyloxy)-3-(3,4-dimethoxyphenyl)-2-(4-methoxyphenyl)non-1-en-5-yl)((perfluorobenzoyl)oxy)carbamate (13)

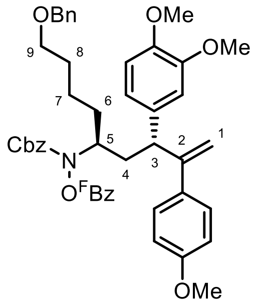

General procedure **I** was followed using CbzNHO^{F}Bz (217 mg, 0.60 mmol), **11** (243 mg, 0.50 mmol), P(3,4,5-(F)$_3C_6H_2)_3$ (255 mg, 0.60 mmol) and diisopropyl azodicarboxylate (0.118 mL, 0.60 mmol). FCC (20% EtOAc/hexane) gave title compound **13** (280 mg, 67%) as a colorless oil. R_f: 0.28 (20% EtOAc/hexane); ν_{max} / cm^{-1}: 2934 (m), 2859 (m), 1781 (s), 1729 (s), 1509 (s), 1246 (s), 1178 (s); *NMR spectra run at 90 °C, mixture of rotamers A + B (9:1)* ^{1}H NMR (500 MHz, *toluene-d^8*) δ 7.31 – 6.93 (15H, m, ArC<u>H</u>, A + B), 6.66 – 6.61 (2H, m, ArC<u>H</u>, A + B), 5.40 (0.9H, s, C**1**-<u>H</u>, A), 5.31 (0.1H, s, C**1**-<u>H</u>, B), 5.21 (0.9H, s, C**1**-<u>H</u>’, A), 5.10 (0.1H, s, C**1**-<u>H</u>’, B), 5.04 (1H, d, *J* = 12.0 Hz, Cbz C<u>H</u>, A + B), 4.96 (1H, d, *J* = 12.0 Hz, Cbz C<u>H</u>’, A + B), 4.74 – 4.66 (1H, m, C**5**-<u>H</u>, A + B), 4.41 – 4.34 (0.9H, m, C**3**-<u>H</u>, A), 4.34 (0.2H, s, Bn C<u>H$_2$</u>, B), 4.31 (1.8H, s, Bn C<u>H$_2$</u>, A), 4.05 – 4.02 (0.1H, m, C**3**-<u>H</u>, B), 3.66 (0.3H, s, OC<u>H$_3$</u>, B), 3.59 (2.7H, s, OC<u>H$_3$</u>, A), 3.48 (0.3H, s, OC<u>H$_3$</u>, B), 3.44 (2.7H, s, OC<u>H$_3$</u>, A), 3.35 (0.3H, s, OC<u>H$_3$</u>, B), 3.33 (2.7H, s, OC<u>H$_3$</u>, A), 3.33 – 3.29 (2H, m, C**9**-<u>H$_2$</u>, A + B), 2.27 – 2.20 (2H, m, C**4**-<u>H$_2$</u>, A + B), 1.80 – 1.90 (1H, m, C6-H, A + B), 1.67 – 1.48 (5H, m, C**6**-<u>H</u>’, C**7**-<u>H$_2$</u>, C**8**-<u>H$_2$</u>, A + B); ^{13}C NMR (126 MHz, *toluene-d^8*) δ 160.0 (Ar<u>C</u>OCH$_3$, A), 155.9 (Cbz <u>C</u>=O, A), 151.3 (C**2**, A), 151.1 (Ar<u>C</u>OCH$_3$, A), 149.7 (Ar<u>C</u>OCH$_3$, A), 139.8 (Bn Ar<u>C</u>, A), 136.9 (Ar<u>C</u>), 136.3 (Cbz Ar<u>C</u>), 135.6 (Ar<u>C</u>), 129.4, 128.9, 128.7, 128.6, 128.5, 127.7, 127.6 (Ar<u>C</u>H, A), 120.9 (Ar<u>C</u>H, A), 114.3 (Ar<u>C</u>H, A), 114.2

(ArCH, A), 114.0 (ArCH, A), 112.1 (C**1**, A), 73.2 (Bn CH_2), 70.5 (C**9**, A), 69.0 (Cbz CH_2, A), 60.3 (C**5**, A), 56.4 (OCH_3, A), 56.2 (OCH_3, A), 54.9 (OCH_3, A), 48.2 (C**3**, B), 47.5 (C**3**, A), 39.3 (C**4**, B), 38.7 (C**4**, A), 34.1 (C**6**, B), 33.1 (C**6**, A), 30.1 (C**8**, A), 23.5 (C**7**, A); *mixture of rotamers A + B (85:15)* ^{19}F NMR (470 MHz, *toluene-d^8*) δ -136.8 – -137.0 (1.7F, m, A), -138.8 – -138-.9 (0.3F, m, B), -148.1 – -148.3 (0.85F, m, A), -151.0 – -151.1 (0.15F, m, B), -160.5 – -160.7 (1.7F, m, A), -161.6 – -161.7 (0.3F, m, B); *m/z* (MALDI$^+$) HRMS: Calculated for $C_{46}H_{44}NNaO_8F_5$: 856.2879. Found $[M+Na]^+$: 856.2870; $[\alpha]^{27}_D$ = – 17.5 (c = 0.36, $CHCl_3$).

The ^{13}C NMR signals corresponding to the pentafluorobenzoyl group could not be resolved due to their weak intensity.

Even at high temperature (90 °C), significant broadening was observed due to amide-like resonance resulting in weak signal intensities. The ^{13}C signals of the minor rotamer could not be resolved in most cases.

Benzyl (2*S*,3a*R*,8a*S*)-2-(4-(benzyloxy)butyl)-5,6-dimethoxy-8a-(4-methoxyphenyl)-3,3a,8,8a-tetrahydroindeno[2,1-*b*]pyrrole-1(2*H*)-carboxylate (15a) and benzyl 2-(4-(benzyloxy)butyl)-6,7-dimethoxy-8a-(4-methoxyphenyl)-3,3a,8,8a-tetrahydroindeno[2,1-*b*]pyrrole-1(2*H*)-carboxylate (15b)

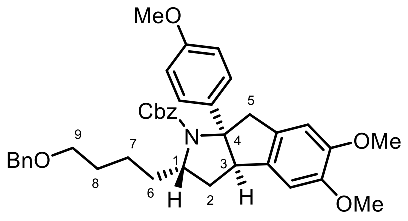

15a

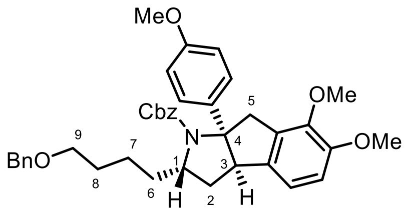

15b

General procedure **J** was followed using **13** (41.7 mg, 0.05 mmol), $Pd_2(dba)_3$ (2.29 mg, 0.0025 mmol), CgP(4-$NO_2C_6H_4$) (5.06 mg, 0.015 mmol), CsOPiv (1.44 mg, 0.01 mmol, *dried at 100 °C under high vacuum and stored in a glovebox*), triethylamine (1.40 µL) and dibutyl ether (0.5 mL). The reaction was stirred at 130 °C for 48 hours. FCC (20 – 30% EtOAc/hexane) gave title compound **15a** (6.0 mg, 19%) and regioisomer **15b** (6.5 mg, 21%) as colorless oils.

*Data for **15a**:* R_f: 0.27 (25% EtOAc/hexane); ν_{max} / cm^{-1}: 2934 (m), 2858 (m), 1694 (s), 1511 (s), 1250 (s), 1110 (s); *NMR spectra run at -35 °C, mixture of rotamers A + B (60:40)* ^{1}H NMR (500 MHz, $CDCl_3$) δ 7.37 – 7.28 (8H, m, ArCH, A + B), 7.22 – 7.16 (2H, m, ArCH), 7.13 – 7.09 (2H, m, ArCH), 6.81 – 6.76 (3H, m, ArCH), 6.74 (0.4H, s, ArCH, B), 6.71 (0.6H, s, ArCH, A), 6.62 (0.4H, s, ArCH, B), 6.60 (0.6H, s, ArCH, A), 5.15 (0.4H, d, *J* = 13.0 Hz, Cbz CH, B), 5.09 (0.4H, d, *J* = 13.0 Hz, Cbz CH',B), 5.04 (0.6H, d, *J* = 13.0 Hz, Cbz CH, A), 4.95 (0.6H, d, *J* = 13.0 Hz, Cbz CH', A), 4.51 (1.2H, s, Bn CH_2, A), 4.47 (0.8H, s, Bn CH_2, B), 4.28 – 4.22 (0.6H, m, C**1**-H, A), 4.17 – 4.12 (0.4H, m, C**1**-H, B),

3.90 (1.2H, s, OC$\underline{H_3}$, B), 3.87 (1.8H, s, OC$\underline{H_3}$, A), 3.83 (1.2H, s, OC$\underline{H_3}$, B), 3.81 (1.8H, s, OC$\underline{H_3}$, A), 3.78 (1.8H, s, OC$\underline{H_3}$, A), 3.75 (1.2H, s, OC$\underline{H_3}$, B), 7.72 – 7.39 (5H, m, C**5**-$\underline{H_2}$, C**3**-$\underline{H}$, C**9**-$\underline{H_2}$, A + B), 2.21 – 2.15 (0.6H, m, C**2**-$\underline{H}$, A), 2.13 – 2.06 (1H, m, C**6**-$\underline{H}$, A, C**2**-$\underline{H}$, B), 2.04 – 1.95 (0.8H, m, C**6**-$\underline{H}$, B, C**2**-$\underline{H}$’, B), 1.94 – 1.86 (0.6H, m, C**2**-$\underline{H}$’, A), 1.69 – 1.33 (5H, m, C**6**-$\underline{H}$’, A + B, C**7**-$\underline{H_2}$, A + B, C**8**-$\underline{H_2}$, A + B); ^{13}C NMR (126 MHz, $CDCl_3$) δ 157.9 (Ar$\underline{C}$OCH$_3$, A + B), 154.8 (Cbz C=O, A), 154.3 (Cbz C=O, B), 148.2 (Ar$\underline{C}$OCH$_3$, B), 148.1 (Ar$\underline{C}$OCH$_3$, A), 147.8 (Ar$\underline{C}$OCH$_3$, A + B), 139.3 (Bn Ar$\underline{C}$, A + B), 138.2 (Ar$\underline{C}$, A), 138.2 (Ar$\underline{C}$, B), 136.7 (Cbz Ar$\underline{C}$, B), 136.5 (Cbz Ar$\underline{C}$, A), 134.2 (Ar$\underline{C}$, B), 134.0 (Ar$\underline{C}$, A), 133.0 (Ar$\underline{C}$, B), 132.7 (Ar$\underline{C}$, A), 128.8, 128.7, 128.5, 128.5, 128.5, 128.2, 128.0, 127.9, 127.9, 127.8, 127.8, 127.7, 127.5, 127.0 (Ar$\underline{C}$H, A + B), 125.3 (Ar$\underline{C}$H, A), 125.1 (Ar$\underline{C}$H, B), 113.4 (Ar$\underline{C}$H, B), 113.3 (Ar$\underline{C}$H, A), 106.7 (Ar$\underline{C}$H, B), 106.5 (Ar$\underline{C}$H, A), 76.6 (C**4**, A + B), 72.9 (Bn $\underline{C}$H$_2$, A + B), 70.3 (C**9**, A), 70.1 (C**9**, B), 66.6 (Cbz $\underline{C}$H$_2$, B), 66.5 (Cbz $\underline{C}$H$_2$, B), 62.3 (C**1**, A), 61.1 (C**1**, B), 59.9 (C**3**, A), 59.0 (C**3**, B), 56.0, 55.9, 55.8, 55.4, 55.3 (O$\underline{C}$H$_3$, A + B), 47.4 (C**5**, A), 46.6 (C**5**, B), 36.5 (C**2**, B), 36.0 (C**2**, A), 34.8 (C**6**, B), 33.9 (C**6**, A), 29.8 (C**8**, A), 29.6 (C**8**, B), 23.9 (C**7**, A), 23.7 (C**7**, B); *m/z* (ESI$^+$) HRMS: Calculated for $C_{39}H_{44}NO_6$: 622.3163. Found [M+H]$^+$: 622.3173; $[\alpha]^{27}_D$ = + 5.40 (c = 0.19, $CHCl_3$).

Data for ***15b****:* R$_f$: 0.23 (20% EtOAc/hexane); ν_{max} / cm^{-1}: 2935 (m), 2859 (m), 1696 (s), 1512 (s), 1489 (s), 1402 (s), 1250 (s), 1033 (s); *NMR spectra run at 100 °C*, ^{1}H NMR (500 MHz, $CDCl_3$) δ 7.35 – 7.14 (10H, m, Bn + Cbz ArC$\underline{H}$), 7.11 (2H, d, J = 8.5 Hz, ArC$\underline{H}$), 6.86 (1H, d, J = 8.0 Hz, ArC$\underline{H}$), 6.83 (1H, d, J = 8.0 Hz, ArC$\underline{H}$), 6.80 (2H, d, J = 8.5 Hz, ArC$\underline{H}$), 5.06 – 5.00 (2H, m, Cbz C$\underline{H_2}$), 4.45 (2H, s, Bn C$\underline{H_2}$), 4.02 (1H, m, C**1**-$\underline{H}$), 3.80 (3H, s, OC$\underline{H_3}$), 3.75 (3H, s, OC$\underline{H_3}$), 3.72 (3H, s, OC$\underline{H_3}$), 3.60 – 3.54 (3H, m, C**3**-$\underline{H}$, C**5**-$\underline{H_2}$), 3.45 (2H, t, J = 6.5 Hz, C**9**-$\underline{H_2}$), 2.06 – 1.95 (3H, m, C**2**-$\underline{H_2}$, C**6**-$\underline{H}$), 1.62 – 1.32 (5H, m, C**6**-$\underline{H}$’, C**7**-$\underline{H_2}$, C**8**-$\underline{H_2}$); ^{13}C NMR (126 MHz, $CDCl_3$) δ 157.5 (Ar$\underline{C}$OCH$_3$), 153.4 (Cbz $\underline{C}$=O), 151.0 (Ar$\underline{C}$OCH$_3$), 144.3 (Ar$\underline{C}$OCH$_3$), 138.5 (Bn Ar$\underline{C}$), 137.8 (Ar$\underline{C}$), 136.5 (Cbz Ar$\underline{C}$), 136.3 (Ar$\underline{C}$), 133.5 (Ar$\underline{C}$), 127.6, 127.1, 126.8, 126.7 (Bn + Cbz Ar$\underline{C}$H), 125.0 (Ar$\underline{C}$H), 118.4 (Ar$\underline{C}$H), 113.2 (Ar$\underline{C}$H), 112.6 (Ar$\underline{C}$H), 76.8 (C**4**), 71.5 (Bn $\underline{C}$H$_2$), 69.2 (C**9**), 65.4 (Cbz $\underline{C}$H$_2$), 60.5 (C**1**), 59.1 (O$\underline{C}$H$_3$), 58.1 (C**3**), 55.8 (O$\underline{C}$H$_3$), 54.7 (O$\underline{C}$H$_3$), 42.3 (C**5**), 35.4 (C**2**), 33.9 (C**6**), 28.6 (C**8**), 22.5 (C**7**); *m/z* (ESI$^+$) HRMS: Calculated for $C_{39}H_{44}NO_6$: 622.3163. Found [M+H]$^+$: 622.3168; $[\alpha]^{27}_D$ = + 7.90 (c = 0.045, $CHCl_3$).

Significant broadening was observed due to amide resonance resulting in weak signal intensities.

Note- the regioisomer assignment of ***15a*** *is based upon the observation of 2 distinctive aromatic singlets in the* ^{1}*H NMR of compound* ***16*** *after removal of the Cbz group. Further evidence is obtained through the observation of 2 aromatic doublets in the high temperature* ^{1}*H NMR of regioisomer* ***15b****.*

4-((2*S*,3a*R*,8a*S*)-5,6-Dimethoxy-8a-(4-methoxyphenyl)-1,2,3,3a,8,8a-hexahydroindeno[2,1-*b*]pyrrol-2-yl)butan-1-ol (16)

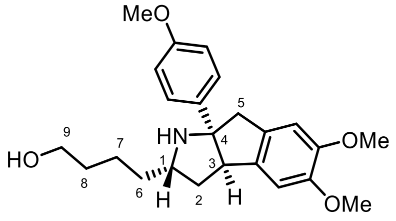

A catalysis tube fitted with a septum was charged with **15a** (19.8 mg, 0.032 mmol) and 5% Pd/C (19.8 mg). Acetic acid (1 mL) was added *via* syringe, and hydrogen gas was bubbled through the solution for 2 minutes. The reaction was stirred vigorously under the balloon of hydrogen for 20 hours. Upon completion, the reaction was filtered through celite, washing with CH_2Cl_2/MeOH 4:1 (20 mL). The filtrate was concentrated thoroughly *in vacuo.* The residue was dissolved in $CHCl_3$ (3mL) and washed with sat. Na_2CO_3 solution (4 × 2 mL). The organic layer was dried (Na_2SO_4) and concentrated *in vacuo.* FCC (10% MeOH/CH_2Cl_2) gave title compound **16** (12.2 mg, 96%) as a colorless oil. R_f: 0.53 (10% MeOH/ CH_2Cl_2); ν_{max} / cm^{-1}: 3370 (br s), 2927 (s), 2855 (s), 1506 (s), 1464 (s), 1247 (s); ^{1}H NMR (400 MHz, $CDCl_3$) δ 7.45 – 7.41 (2H, m, ArCH), 7.87 – 7.83 (2H, m, ArCH), 6.71 (1H, s, ArCH), 6.88 (1H, s, ArCH), 3.89 (3H, s, OCH_3), 3.85 (3H, s, OCH_3), 3.79 (3H, s, OCH_3), 3.73 (1H, br d, *J* = 8.0 Hz, C**3**-H), 3.63 (2H, t, *J* = 6.5 Hz, C**9**-H_2), 3.39 (1H, d, *J* = 17.0 Hz, C**5**-H), 3.18 (1H, d, *J* = 17.0 Hz, C**5**-H’), 3.11 – 3.03 (1H, m, C**1**-H), 2.06 (1H, ddd, *J* = 12.0, 5.5, 1.0 Hz, C**2**-H), 1.68 – 1.53 (4H, m, C**2**-H’, C**6**-H, C**8**-H_2), 1.49 – 1.37 (3H, m, C**6**-H’, C**7**-H_2); ^{13}C NMR (101 MHz, $CDCl_3$) δ 158.1 (ArCOCH$_3$), 148.9 (ArCOCH$_3$), 148.8 (ArCOCH$_3$), 141.7 (ArC), 137.5 (ArC), 133.7 (ArC), 126.4 (ArCH), 113.6 (ArCH), 107.4 (ArCH), 107.3 (ArCH), 75.3 (C**4**), 62.9 (C**9**), 58.6 (C**3**), 58.3 (C**1**), 56.2 (OCH_3), 56.1 (OCH_3), 55.4 (OCH_3), 51.4 (C**5**), 40.9 (C**2**), 36.1 (C**6**), 32.9 (C**8**), 23.7 (C**7**); *m/z* (ESI^+) HRMS: Calculated for $C_{24}H_{32}NO_4$: 398.2326. Found $[M+H]^+$: 398.2329; $[\alpha]^{27}_D$ = – 7.15 (c = 0.61, $CHCl_3$).

(+)-Pileamartine A (17)

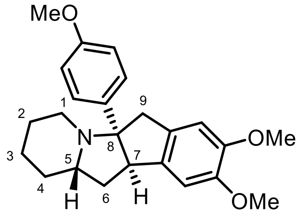

To a solution of CBr_4 (24.9 mg, 0.075 mmol) and triphenylphosphine (19.7 mg, 0.075 mmol) in CH_2Cl_2 (0.5 mL), was added NEt_3 (20.9 µL, 0.15 mmol) *via* syringe. The resulting mixture was added dropwise to a separate solution of precursor **16** (9.80 mg, 0.025 mmol) in CH_2Cl_2 (0.5 mL) at 0 °C *via* syringe. The reaction was allowed to warm to room temperature and stirred for 16 hours. Upon completion, the reaction mixture was concentrated *in vacuo* and the residue was purified by FCC (15% EtOAc/hexane)

to give (+)-Pileamartine A (6.30 mg, 66%) as a colorless solid. m.p.: = 180 °C (CH_2Cl_2/hexane); R_f: 0.36 (15% EtOAc/hexane); ν_{max} / cm^{-1}: 2928 (s), 2856 (m), 1508 (s), 1454 (m), 1246 (s); ^{1}H NMR (500 MHz, $CDCl_3$) δ 7.39 – 7.36 (2H, m, ArCH), 6.84 – 6.81 (2H, m, ArCH), 6.76 (1H, s, ArCH), 6.63 (1H, s, ArCH), 3.89 (3H, s, OCH_3), 3.84 (3H, s, OCH_3), 3.78 (3H, s, OCH_3), 3.50 (1H, d, *J* = 17.0 Hz, C**9**-H), 3.27 (1H, dd, *J* = 8.5, 3.0 Hz, C**7**-H), 2.94 (1H, d, *J* = 17.0 Hz, C**9**-H'), 2.77 (1H, m, C**1**-H), 2.39 (1H, m, C**5**-H), 2.19 (1H, m, C**1**-H'), 1.85 – 1.75 (4H, m, C**6**-H_2, C**3**-H, C**4**-H), 1.67 – 1.58 (2H, m, C**2**-H_2), 1.31 – 1.15 (2H, m, C**3**-H', C**4**-H'); ^{13}C NMR (125 MHz, $CDCl_3$) δ 158.0 (ArCOCH₃), 148.9 (ArCOCH₃), 148.6 (ArC OCH₃), 141.2 (ArC), 137.4 (ArC), 134.8 (ArC), 126.6 (2 × ArCH), 113.5 (2 × ArCH), 107.6 (ArCH), 106.8 (ArCH), 77.5 (C**8**), 59.7 (C**5**), 59.2 (C**7**), 56.1 (2 × OCH_3), 55.4 (OCH_3), 46.7 (C**1**), 40.2 (C**6**), 38.0 (C**9**), 32.5 (C**4**), 26.1 (C**2**), 24.6 (C**3**); *m/z* (ESI$^+$) HRMS: Calculated for $C_{24}H_{30}NO_3$: 380.2220. Found $[M+H]^+$: 380.2217; $[\alpha]^{25}_D$ = + 144.1 (c = 0.32, $CHCl_3$).

The spectroscopic properties match the data available in the isolation literature.[47]

The relative stereochemistry was also confirmed by nOe analysis:

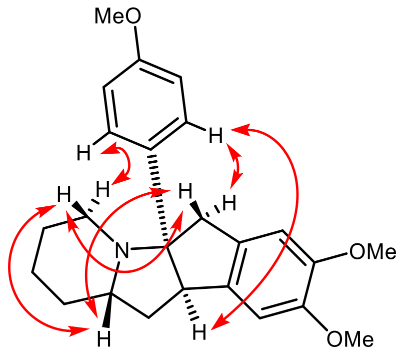

Optimization of the Key Catalysis Cyclization

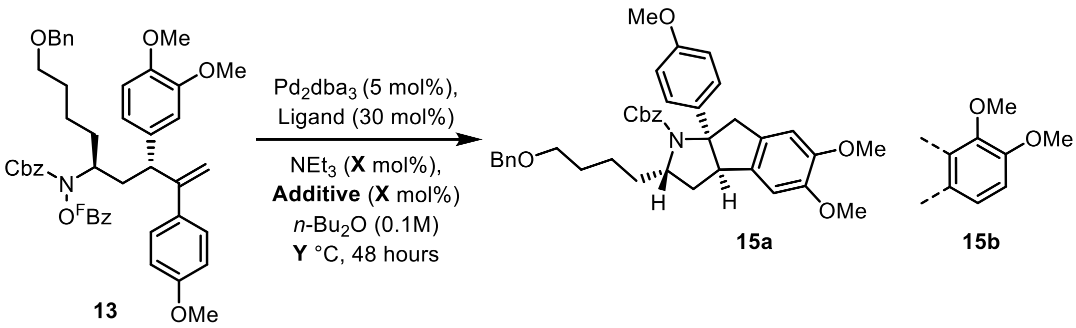

Entry	Ligand	Additive	X	Y	Isolated Yield	Ratio (15a:15b)
**1**	CgPPh	NaOBz	10	130	19%	1.3:1
**2**	CgP(3,5-$(CF_3)_2C_6H_3$)	NaOBz	10	130	24%	1.2:1
**3**	CgP(4-(CO_2Et)C_6H_4)	NaOBz	10	130	23%	1:1
**4**	CgP(4-(NO_2)C_6H_4)	NaOBz	20	130	32%	1:1
**5**	P(3,5$(CF_3)_2C_6H_3)_3$	NaOBz	20	130	0%	–
**6**	CgP(4-(NO_2)C_6H_4)	NaOAc	20	130	29%	1:1
**7**	CgP(4-(NO_2)C_6H_4)	CsOPiv	20	130	**40%**	1:1
**8**	CgP(4-(NO_2)C_6H_4)	CsOPiv	20	120	25%	1:1
**9**	CgP(4-(NO_2)C_6H_4)	CsOPiv	20	140	32%	1:1
**10**	CgP(4-(NO_2)C_6H_4)	CsOPiv	20	150	21%	1:1
**11**	CgP(3,5-$(F)_2$-4-(NO_2)C_6H_2)	CsOPiv	20	130	47%	1:1.4
**12**	CgP(3,5-$(NO_2)_2C_6H_3$)	CsOPiv	20	130	44%	1:1.7
**13**	CgP(4-(NO_2)C_6H_4)	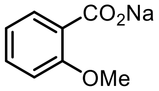 CO_2Na, OMe	20	130	35%	1:1
**14**	CgP(3,5-$(F)_2$-4-(NO_2)C_6H_2)	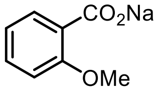 CO_2Na, OMe	20	130	25%	1:1

Synthesis of (-)-Pileamartine A

The following (non-optimized) route was used to prepare (-)-pileamartine A:

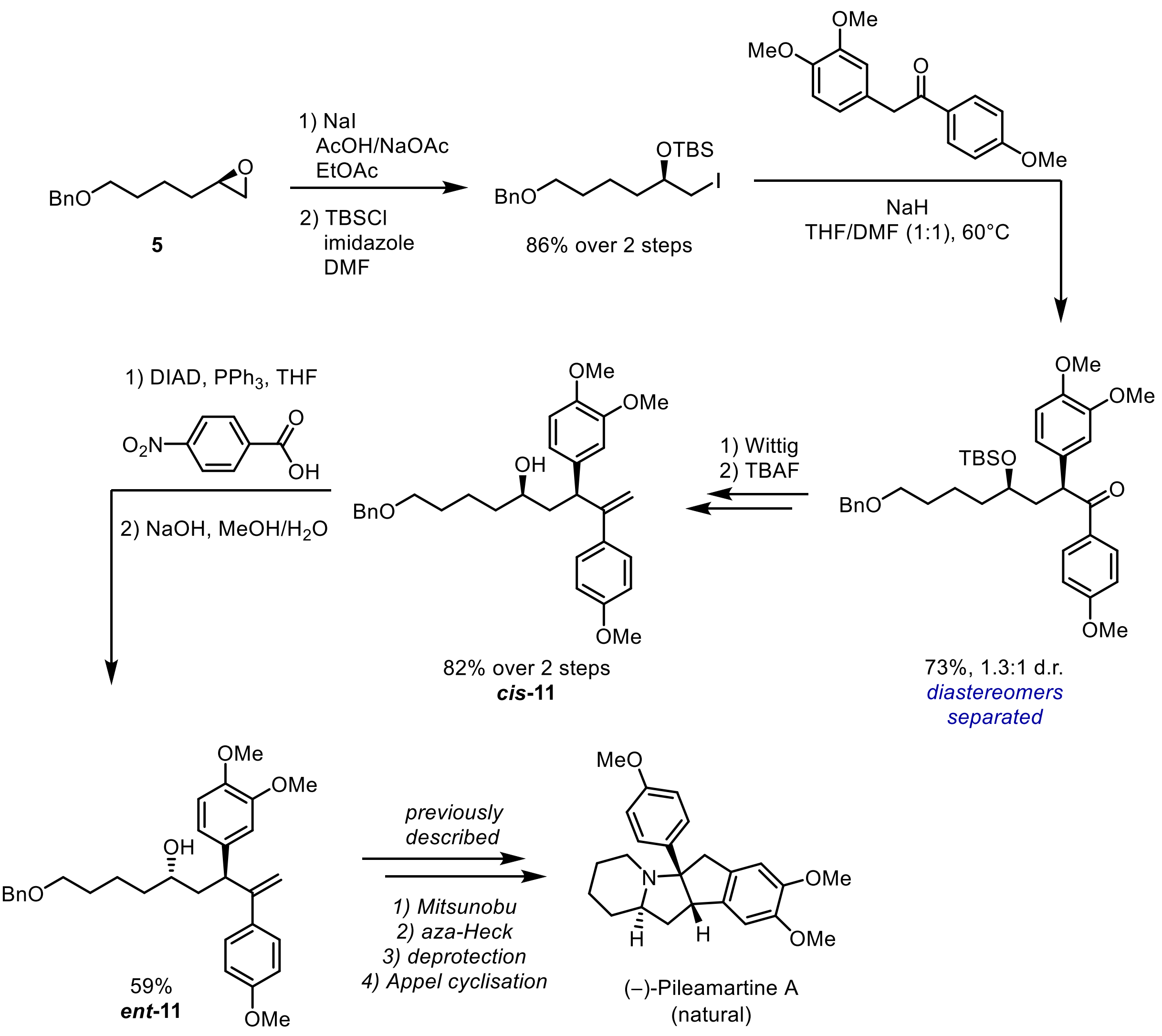

Experimental Procedures and Data

2-(3,4-Dimethoxyphenyl)-1-(4-methoxyphenyl)ethan-1-one

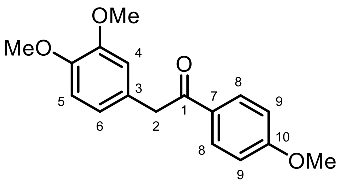

A solution of 3,4-dimethoxyphenylacetic acid (3.92 g, 20.0 mmol) in $SOCl_2$ (29.2 mL, 400 mmol) was heated at reflux (85 °C) for 3 hours. Upon completion, the reaction was cooled and concentrated *in vacuo*. The residue was dissolved in anhydrous CH_2Cl_2 (7 mL) and added to a separate solution of anisole (4.35 mL, 40.0 mmol) and aluminium trichloride (8.00 g, 60.0 mmol) in anhydrous CH_2Cl_2 (80

mL) *via* syringe. The reaction was stirred at room temperature for 2 hours. Upon completion, the reaction was cooled to 0 °C and quenched with sat. Na_2CO_3 solution (200 mL), diluted with water (100 mL), and the aqueous layer extracted with CH_2Cl_2 (3 × 100 mL). The combined organic extracts were washed with brine (100 mL), dried (Na_2SO_4) and concentrated *in vacuo*. FCC (20 – 50% EtOAc/hexane) gave the title compound (4.91 g, 86%) as a colorless solid. m.p.: 136 °C (CH_2Cl_2/hexane) [Lit.: 136 °C][48]; ^{1}H NMR (400 MHz, $CDCl_3$) δ 8.02 – 7.97 (2H, m, C**8**-H), 6.95 – 6.89 (2H, m, C**9**-H), 6.83 – 6.80 (2H, m, C**5**-H + C**6**-H), 6.80 – 6.78 (1H, m, C**4**-H), 4.17 (2H, s, C**2**-H_2), 3.89 – 3.81 (9H, m, 3 × OCH_3); ^{13}C NMR (101 MHz, $CDCl_3$) δ 196.6 (C**1**), 163.7 (C**10**), 149.2 (Ar$COCH_3$), 148.1 (Ar$COCH_3$), 131.1 (C**9**), 129.8 (C**3**), 127.6 (C**7**), 121.6 (C**6**), 113.9 (C**8**), 112.6 (C**4**), 111.5 (C**5**), 56.0 (OCH_3), 56.0 (OCH_3), 55.6 (OCH_3), 45.0 (C**2**).

The spectroscopic properties were consistent with the data available in the literature.[49]

(*R*)-((6-(Benzyloxy)-1-iodohexan-2-yl)oxy)(*tert*-butyl)dimethylsilane

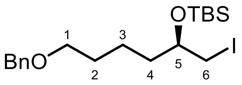

To a solution of **5** (800 mg, 3.88 mmol) in EtOAc (39 mL), was added NaI (755 mg, 5.04 mmol), NaOAc (350 mg, 4.27 mmol) and acetic acid (0.244 mL, 4.27 mmol) sequentially. The flask was wrapped in foil, and the reaction was stirred at room temperature for 16 hours. Upon completion, the reaction was diluted with water (50 mL) and the aqueous layer was extracted with EtOAc (3 × 30 mL). The combined organic layers were washed with sat. aq. $Na_2S_2O_3$ solution (50 mL), brine (50 mL), dried (Na_2SO_4) and concentrated *in vacuo*. The crude residue was dissolved in DMF (20 mL) before the addition of *tert*-butyldimethylsilyl chloride (1.17 g, 7.76 mmol) and imidazole (1.06 g, 15.5 mmol). The reaction was stirred at room temperature for 7 hours. Upon completion, the reaction was diluted with water (50 mL) and the aqueous layer was extracted with Et_2O (3 × 50 mL). The combined organic layers were washed with sat. NH_4Cl solution (50 mL), sat. $NaHCO_3$ solution (50 mL), dried (Na_2SO_4) and concentrated *in vacuo*. FCC (1% EtOAc/hexane) gave the title compound (1.49 g, 86% over 2 steps) as a colorless oil. R_f: 0.21 (1% EtOAc/hexane); ν_{max} / cm^{-1}: 2928 (s), 2855 (s), 1254 (s), 1099 (s), 835 (s); ^{1}H NMR (400 MHz, $CDCl_3$) δ 7.36 – 7.31 (4H, m, ArCH), 7.31 – 7.26 (1H, m, ArCH), 4.50 (2H, s, Bn CH_2), 3.54 (1H, m, C**5**-H), 3.48 (2H, t, J = 6.5 Hz, C**1**-H_2), 3.19 (2H, d, J = 5.0 Hz, C**6**-H_2), 1.68 – 1.56 (4H, m, C**4**-H_2, C**2**-H_2), 1.49 – 1.33 (2H, m, C**3**-H_2), 0.90 (9H, s, TBS $C(CH_3)_3$), 0.10 (3H, s, TBS CH_3), 0.06 (3H, s, TBS CH_3); ^{13}C NMR (101 MHz, $CDCl_3$) δ 138.8 (ArC), 128.5 (ArCH), 127.8 (ArCH), 127.6 (ArCH), 73.1 (Bn CH_2), 71.5 (C**5**), 70.3 (C**1**), 36.9 (C**4**), 29.8 (C**2**), 26.0 (TBS $C(CH_3)_3$), 21.9 (C**3**), 18.2 (TBS $C(CH_3)_3$), 14.1 (C**6**), -4.2 (TBS CH_3), -4.4 (TBS CH_3); $[\alpha]^{27}_D$ = + 8.57 (c = 0.62, $CHCl_3$).

The spectroscopic properties were consistent with the data available in the literature.[50]

(2*S*,4*R*)-8-(Benzyloxy)-4-((*tert*-butyldimethylsilyl)oxy)-2-(3,4-dimethoxyphenyl)-1-(4-methoxyphenyl)octan-1-one

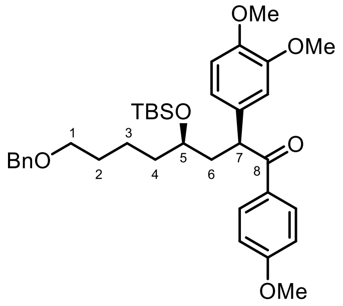

To a suspension of NaH 60% dispersion in mineral oil (200 mg, 5.00 mmol) in anhydrous THF (10 mL) cooled to 0 °C, was added 2-(3,4-dimethoxyphenyl)-1-(4-methoxyphenyl)ethan-1-one (1.43 g, 5.00 mmol) as a solution in anhydrous DMF (25 mL) dropwise *via* syringe. The reaction was stirred at room temperature for 1 hour, before the addition of a solution of the preceding alkyl iodide (4.48 g, 10.0 mmol) in anhydrous THF (15 mL) *via* syringe. The reaction was stirred at 60 °C for 16 hours. Upon completion, the reaction was quenched with sat. NH_4Cl solution (100 mL) and the aqueous layer was extracted with EtOAc (3 × 100 mL). The combined organic layers were washed with water (50 mL), brine (50 mL), dried (Na_2SO_4) and concentrated *in vacuo*. FCC (10-20% EtOAc/hexane) gave the title compound (2.22 g, 73%, *1.3:1 cis:trans mixture of diastereomers*). *Separation of diastereomers:* FCC (5% EtOAc/toluene, SiO_2 particle size 19 – 37 μm) gave the *cis* diastereomer (1.24 g, 41%) and *trans* diastereomer (0.98 g, 32%) as colorless oils. *Data for the cis material:* R_f: 0.19 (5% EtOAc/toluene); ν_{max} / cm^{-1}: 2932 (s), 2856 (s), 1672 (s), 1600 (s), 1513 (s), 1259 (s); ^1H NMR (400 MHz, $CDCl_3$) δ 7.96 – 7.92 (2H, m, ArCH), 7.35 – 7.27 (5H, m, ArCH), 6.87 – 6.83 (2H, m, ArCH), 6.82 – 6.73 (3H, m, ArCH), 4.68 (1H, dd, *J* = 9.5, 4.5 Hz, C**7**-H), 4.50 (2H, s, Bn CH_2), 3.83 (3H, s, OCH_3), 3.82 (3H, s, OCH_3), 3.81 (3H, s, OCH_3), 3.63 (1H, m, C**5**-H), 3.46 (2H, t, *J* = 6.5 Hz, C**1**-H_2), 2.44 (1H, ddd, *J* = 13.5, 9.5, 4.0 Hz, C**6**-H), 1.74 (1H, m, C**6**-H'), 1.65 – 1.58 (2H, m, C**2**-H_2), 1.55 – 1.39 (4H, m, C**3**-H_2, C**4**-H_2), 0.87 (9H, s, TBS $C(CH_3)_3$), -0.06 (3H, s, TBS CH_3), -0.18 (3H, s, TBS CH_3); ^{13}C NMR (101 MHz, $CDCl_3$) δ 198.2 (C**8**), 163.3 (ArC), 149.5 (ArC), 148.0 (ArC), 138.8 (Bn ArC), 133.6 (ArC), 131.2 (ArCH), 130.0 (ArC), 128.5 (Bn ArCH), 127.8 (Bn ArCH), 127.6 (Bn ArCH), 120.6 (ArCH), 113.7 (ArCH), 111.7 (ArCH), 110.9 (ArCH), 73.0 (Bn CH_2), 70.6 (C**1**), 70.5 (C**5**), 56.0 (OCH_3), 56.0 (OCH_3), 55.5 (OCH_3), 49.2 (C**7**), 41.6 (C**6**), 37.7 (C**4**), 30.1 (C**2**), 26.1 (TBS $C(CH_3)_3$), 21.6 (C**3**), 18.2 (TBS $C(CH_3)_3$), -4.0 (TBS CH_3), -4.5 (TBS CH_3); *m/z* (ESI^+) HRMS: Calculated for $C_{36}H_{51}O_6Si$: 607.3449. Found $[M+H]^+$: 607.3455; $[\alpha]^{27}_D$ = + 72.7 (c = 0.66, $CHCl_3$).

*Note – the relative stereochemistry was assigned by analysis of derivative **cis-11**. The stereochemistry of **trans-11** was ultimately confirmed by single crystal X-ray analysis of lactone **8**. Comparison of the NMR data for **cis-11** and **trans-11** was used to infer the stereochemistry of the title compound.*

(((3*S*,5*R*)-9-(Benzyloxy)-3-(3,4-dimethoxyphenyl)-2-(4-methoxyphenyl)non-1-en-5-yl)oxy)(*tert*-butyl)dimethylsilane

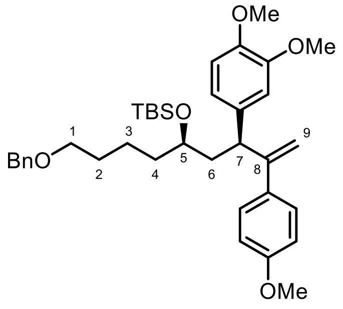

To a solution of methyltriphenylphosphonium bromide (2.00 g, 5.59 mmol) in anhydrous THF (25 mL) at -78 °C, was added *n*-BuLi (1.5 M in hexane, 3.73 mL) dropwise *via* syringe. The solution was allowed to warm to 0 °C and stirred for 1 hour. The reaction was then cooled to -78 °C, and the preceding ketone (1.13 g, 1.86 mmol) in anhydrous THF (5 mL) was added *via* syringe. The reaction was stirred at this temperature for 30 minutes, then warmed to 0 °C for 2 hours, and finally allowed to stir at room temperature for 16 hours. Upon completion, the reaction was quenched with sat. NH_4Cl solution (50 mL), and the aqueous layer was extracted with EtOAc (3 × 30 mL). The combined organic layers were washed with brine (50 mL), dried (Na_2SO_4), and concentrated *in vacuo*. FCC (10 – 20% EtOAc/hexane) gave the title compound (1.03 g, 92%) as a colorless oil. R_f: 0.44 (15% EtOAc/hexane); ν_{max} / cm^{-1}: 2931 (s), 2855 (s), 1511 (s), 1463 (s), 1248 (s), 1031 (s), 834 (s); ^1H NMR (400 MHz, $CDCl_3$) δ 7.36 – 7.26 (5H, s, ArC<u>H</u>), 7.21 – 7.18 (2H, m, ArC<u>H</u>), 6.79 – 6.73 (5H, m, ArC<u>H</u>), 5.40 (1H, m, C**9**-<u>H</u>), 5.11 (1H, m, C**9**-<u>H</u>’), 4.50 (2H, s, Bn C<u>H</u>$_2$), 3.88 – 3.80 (1H, m, C**7**-<u>H</u>), 3.84 (3H, s, OC<u>H</u>$_3$), 3.84 (3H, s, OC<u>H</u>$_3$), 3.76 (3H, s, OC<u>H</u>$_3$), 3.65 (1H, m, C**5**-<u>H</u>), 3.46 (2H, t, *J* = 6.5 Hz, C**1**-<u>H</u>$_2$), 2.03 (1H, ddd, *J* = 13.5, 8.0, 5.5 Hz, C**6**-<u>H</u>), 1.86 (1H, m, C**6**-<u>H</u>’), 1.63 – 1.34 (6H, m, C**2**-<u>H</u>$_2$, C**3**-<u>H</u>$_2$, C**4**-<u>H</u>$_2$), 0.86 (9H, s, TBS C(C<u>H</u>$_3$)$_3$), -0.06 (3H, s, TBS C<u>H</u>$_3$), -0.10 (3H, s, TBS C<u>H</u>$_3$); ^{13}C NMR (101 MHz, $CDCl_3$) δ 158.9 (Ar<u>C</u>), 150.4 (C**8**), 149.0 (Ar<u>C</u>), 147.5 (Ar<u>C</u>), 138.8 (Bn Ar<u>C</u>), 136.7 (Ar<u>C</u>), 135.0 (Ar<u>C</u>), 128.5 (Ar<u>C</u>H), 128.0 (Ar<u>C</u>H), 127.7 (Ar<u>C</u>H), 127.6 (Ar<u>C</u>H), 120.3 (Ar<u>C</u>H), 113.5 (Ar<u>C</u>H), 111.9 (C**9**), 111.2 (Ar<u>C</u>H), 111.2 (Ar<u>C</u>H), 73.0 (Bn <u>C</u>H$_2$), 70.5 (C**1**), 70.3 (C**5**), 56.0 (O<u>C</u>H$_3$), 55.9 (O<u>C</u>H$_3$), 55.3 (O<u>C</u>H$_3$), 46.2 (C**7**), 43.3 (C**6**), 37.2 (C**4**), 30.1 (C**2**), 26.1 (TBS C(<u>C</u>H$_3$)$_3$), 21.6 (C**3**), 18.2 (TBS <u>C</u>(CH$_3$)$_3$), -4.1 (TBS <u>C</u>H$_3$), -4.4 (TBS <u>C</u>H$_3$); *m/z* (ESI^+) HRMS: Calculated for $C_{37}H_{56}NO_5Si$: 622.3922. Found $[M+NH_4]^+$: 622.3919; $[\alpha]^{27}_D$ = + 46.3 (c = 0.23, $CHCl_3$).

(3*S*,5*R*)-9-(Benzyloxy)-3-(3,4-dimethoxyphenyl)-2-(4-methoxyphenyl)non-1-en-5-ol (*cis*-11)

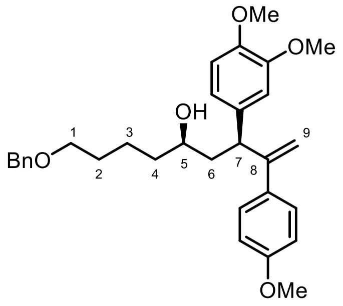

To a solution of the preceding protected alcohol (1.03 g, 1.70 mmol) in anhydrous THF (17 mL), was added TBAF (1M in THF, 5.12 mL) *via* syringe. The reaction was stirred at room temperature for 72 hours. Upon completion, the reaction was diluted with water (40 mL) and sat. $NaHCO_3$ solution (40 mL), and the aqueous layer was extracted with EtOAc (3 × 40 mL). The combined organic layers were washed with brine (100 mL), dried (Na_2SO_4), and concentrated *in vacuo*. FCC (30 – 50% EtOAc/hexane) gave the title compound (746 mg, 89%) as a colorless oil. R_f: 0.18 (30% EtOAc/hexane); ν_{max} / cm^{-1}: 3440 (br s), 2934 (s), 2858 (m), 1606 (m), 1510 (s), 1245 (s), 1220 (s); ^{1}H NMR (400 MHz, $CDCl_3$) δ 7.35 – 7.26 (5H, m, Bn ArCH), 7.21 – 7.17 (2H, m, ArCH), 6.83 – 6.74 (5H, m, ArCH), 5.37 (1H, m, C**9**-H), 5.13 (1H, m, C**9**-H'), 4.49 (2H, s, Bn CH_2), 4.00 (1H, dd, *J* = 9.0, 6.5 Hz, C**7**-H), 3.82 (3H, s, OCH_3), 3.82 (3H, s, OCH_3), 3.76 (3H, s, OCH_3), 3.72 (1H, m, C**5**-H), 3.46 (2H, t, *J* = 6.5 Hz, C**1**-H_2), 2.04 (1H, ddd, *J* = 14.0, 9.0, 3.5 Hz, C**6**-H), 1.86 (1H, ddd, *J* = 14.0, 9.0, 6.5 Hz, C**6**-H'), 1.65 – 1.39 (6H, m, C**2**-H_2, C**3**-H_2, C**4**-H_2); ^{13}C NMR (101 MHz, $CDCl_3$) δ 159.0 (ArC), 150.5 (C**8**), 149.1 (ArC), 147.7 (ArC), 138.7 (Bn ArC), 136.4 (ArC), 135.0 (ArC), 128.5 (ArCH), 128.1 (ArCH), 127.8 (ArCH), 127.7 (ArCH), 119.9 (ArCH), 113.6 (ArCH), 112.3 (C**9**), 111.3 (ArCH), 111.3 (ArCH), 73.1 (Bn CH_2), 70.4 (C**1**-H), 70.2 (C**5**), 56.0 (2 × OCH_3), 55.3 (OCH_3), 46.9 (C**7**), 43.2 (C**6**), 37.8 (C**4**), 29.8 (C**2**), 22.4 (C**3**); *m/z* (ESI^+) HRMS: Calculated for $C_{31}H_{39}O_5$: 491.2792. Found $[M+H]^+$: 491.2802; $[\alpha]^{27}_D$ = + 31.3 (c = 0.29, $CHCl_3$).

(3*S*,5*S*)-9-(Benzyloxy)-3-(3,4-dimethoxyphenyl)-2-(4-methoxyphenyl)non-1-en-5-ol (*ent*-11)

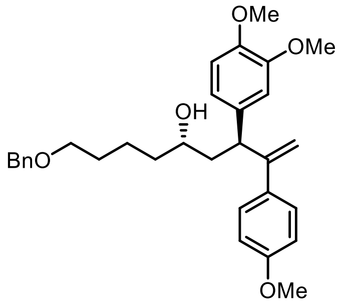

To a solution of ***cis*-11** (1.88 g, 3.83 mmol), triphenylphosphine (2.01 g, 7.66 mmol) and 4-nitrobenzoic acid (1.28 g, 7.66 mmol) in anhydrous THF (38 mL) at 0 °C, was added diisopropyl azodicarboxylate (1.51 mL, 7.66 mmol). The reaction was stirred at room temperature for 7 hours. NaOH (1.22 g, 30.6

mmol) was then added, along with MeOH (30 mL) and water (15 mL) *via* syringe. The reaction was stirred vigorously for 16 hours. Upon completion, the reaction was poured into sat. NH_4Cl solution (100 mL) and the aqueous layer was extracted with EtOAc (3 × 50 mL). The combined organic layers were washed with brine (100 mL), dried (Na_2SO_4), and concentrated in *vacuo*. FCC (25% EtOAc/toluene gave the title compound (1.11 g, 59%) as a colorless oil.

The data for this compound is given previously for compound **11**.

$[\alpha]^{27}_{D}$ = + 19.6 (c = 0.56, $CHCl_3$).

Benzyl ((3*S*,5*R*)-9-(benzyloxy)-3-(3,4-dimethoxyphenyl)-2-(4-methoxyphenyl)non-1-en-5-yl)((perfluorobenzoyl)oxy)carbamate (*ent*-13)

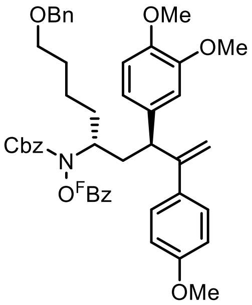

The procedure and data for this compound are given previously for compound **13**.

$[\alpha]^{27}_{D}$ = + 18.6 (c = 0.32, $CHCl_3$).

Benzyl (2*R*,3a*S*,8a*R*)-2-(4-(benzyloxy)butyl)-5,6-dimethoxy-8a-(4-methoxyphenyl)-3,3a,8,8a-tetrahydroindeno[2,1-*b*]pyrrole-1(2*H*)-carboxylate (*ent*-15a)

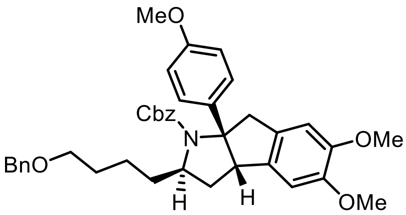

The procedure and data for this compound are given previously for compound **15a**.

$[\alpha]^{27}_{D}$ = – 67.2 (c = 0.97, $CHCl_3$).

4-((2*R*,3a*S*,8a*R*)-5,6-dimethoxy-8a-(4-methoxyphenyl)-1,2,3,3a,8,8a-hexahydroindeno[2,1-*b*]pyrrol-2-yl)butan-1-ol (*ent*-16)

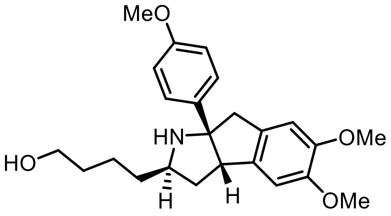

The procedure and data for this compound are given previously for compound **16**.

$[\alpha]^{25}_{D}$ = + 5.20 (c = 0.25, $CHCl_3$).

(–)-Pileamartine A

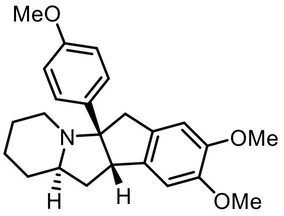

The procedure and data for this compound are given previously for compound **17**.

$[\alpha]^{25}_{D}$ = – 127.1 (c = 0.20, $CHCl_3$) [lit: $[\alpha]^{25}_{D}$ = – 141.2 (c = 0.33, $CHCl_3$)][47]

Vibrational Circular Dichroism (VCD) and Electronic Circular Dichroism (ECD)

Experimental ECD: Data was acquired on samples at a concentration of 0.13 mg/ml in MeCN in a 1 mm path length quartz cuvette using a JASCO J-815 CD spectrometer at 25 °C.

Experimental VCD: (+)-Pileamartine A (**17**) was dissolved at a concentration of 0.105 M in $CDCl_3$. The solution was transferred to a 0.0995 mm BaF_2 cell and VCD spectra acquired for 12 hours in a Biotools ChiralIR2X instrument at a resolution of 4 cm^{-1} and PEM setting of 1400 cm^{-1}. A blank spectrum of the solvent was also acquired. Infra-red spectra were acquired on the samples concomitantly. The experimental infra-red and VCD spectra are shown below.

Experimental VCD (green) and infra-red (red) spectra for (+)-pileamartine A (17) and spectra from $CDCl_3$ solvent alone (blue).

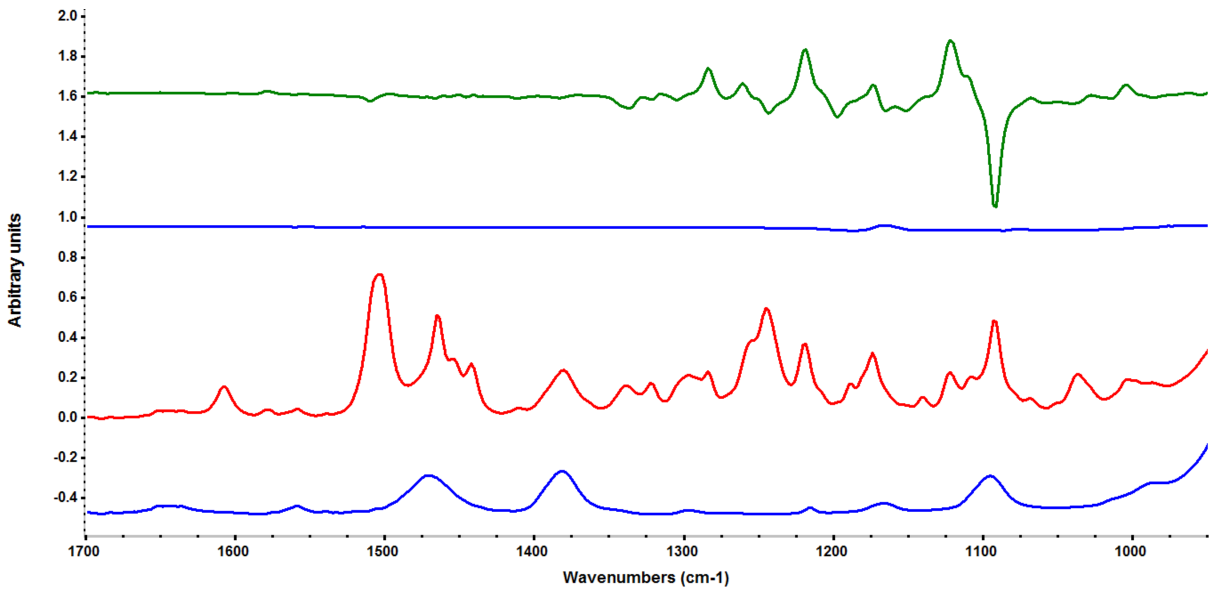

Computational Spectral Simulations: A Monte Carlo molecular mechanics search for low energy geometries was conducted for the 6*S*,8*R*,9*S* stereoisomer of pilemartine A. *MacroModel* within the *Maestro* graphical interface (Schrödinger Inc.) was used to generate 38 starting coordinates for conformers within 21 $kJmol^{-1}$ of the lowest energy conformer. These were used as starting points for density functional theory (DFT) minimizations within *Gaussian16*.[51] Optimized structures, harmonic vibrational frequencies/intensities, VCD rotational strengths, and free energies at STP (including zero-point energies) were determined at the B3PW91/cc-pVTZ level of theory using the default polarizable

continuum model (PCM) for chloroform (VCD) or acetonitrile (ECD). TD-DFT calculations of 30 excited states were made on those conformations within 5 kJmol^{-1} of the lowest energy conformation, also using a PCM model for acetonitrile.

Only two conformations were found within 5 kJmol^{-1} of the minimum, and these are shown overlaid below. They differ only in the orientation of the mono-methoxy aromatic ring. The coordinates of the minimum energy conformation are shown below. The conformations found in both chloroform and acetonitrile PCM models were identical, though the relative energy differed slightly.

Overlay of the two lowest energy conformations (within 5 kJmol^{-1} of the minimum) used in the calculation of the Boltzmann average IR, VCD and ECD spectra.

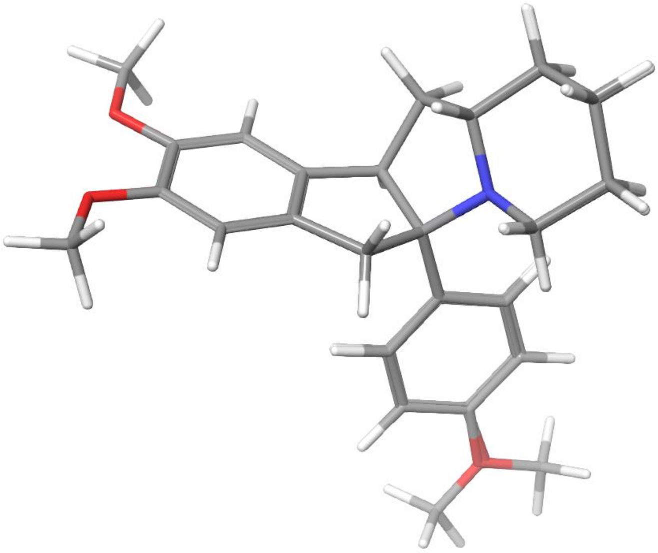

Coordinates for the minimum energy conformation.

C	-1.45890	0.16680	-0.57910
C	-2.59370	-0.15520	-1.32430
C	-3.73640	-0.60250	-0.67970
C	-3.74450	-0.73000	0.73080
C	-2.61010	-0.40540	1.45890
C	-1.46740	0.04240	0.79580
C	-0.13890	0.66230	-1.08990
H	-2.58030	-0.06230	-2.40190
O	-4.89050	-0.94870	-1.30400
O	-4.90550	-1.17970	1.26990
H	-2.61090	-0.50050	2.53640
C	-0.15940	0.47000	1.38890
C	-4.93420	-0.83970	-2.71250
C	-4.96620	-1.32360	2.67460
H	-4.77290	0.19150	-3.04060

H	-4.19290	-1.48820	-3.18880
H	-5.93110	-1.15780	-3.00900
H	-4.81030	-0.36750	3.18330
H	-5.96610	-1.68880	2.89760
H	-4.22970	-2.04720	3.03650
H	0.22550	-0.22810	2.13460
C	0.80890	0.63290	0.17100
H	-0.27330	1.42910	1.90360
C	1.81160	-0.50860	0.07290
N	1.47400	1.93400	0.10690
C	-0.11200	2.12690	-1.57780
H	0.24670	0.00540	-1.87120
C	0.52080	2.89670	-0.42620
H	0.52100	2.21790	-2.46330
H	-1.10500	2.49760	-1.83370
H	-0.25930	3.13210	0.32450
C	1.22050	4.19580	-0.79010
C	2.18260	2.39300	1.28380
C	2.93400	3.68170	0.97180
H	1.49700	2.57950	2.13010
H	2.87790	1.61340	1.60370
H	3.42290	4.04520	1.87910
H	3.72190	3.46590	0.24390
C	1.99310	4.74210	0.40770
H	0.48670	4.92530	-1.14320
H	1.91070	4.00000	-1.61740
H	2.55120	5.63830	0.12610
H	1.28440	5.04710	1.18630
C	3.06110	-0.31720	-0.52250
C	1.49720	-1.79840	0.48880
C	2.38170	-2.86130	0.33110
C	3.95720	-1.35810	-0.67970
C	3.62550	-2.64400	-0.25270
H	0.53380	-2.00480	0.93860
H	2.08850	-3.84570	0.66820
O	4.56650	-3.60450	-0.44600
H	4.92620	-1.19330	-1.13480
H	3.32500	0.67500	-0.86490
C	4.26370	-4.92290	-0.03160
H	3.39390	-5.32020	-0.56260
H	5.13650	-5.52480	-0.27440
H	4.08140	-4.97240	1.04580

Fit between calculated and experimental ECD spectra

Analysis of the ECD results was performed using Gaussview version 6.1.1 and a Boltzmann weighted average spectrum computed from the two conformations within the 5 $kJmol^{-1}$ limit. After applying a UV shift correction of +4 nm, the calculated spectrum was compared to the experimental data (Figure S1). There is a good match between the calculated spectrum for 6*S*,8*R*,9*S* and (+)-pileamartine A (**17**).

Figure S1. Comparison of experimental and calculated ECD spectra. The calculated spectrum for the 6*S*,8*R*,9*S* stereoisomer of pilemartine A (blue) is compared to the experimental spectra for (+)-pileamartine A (**17**) (grey) and (–)-pileamartine A (orange).

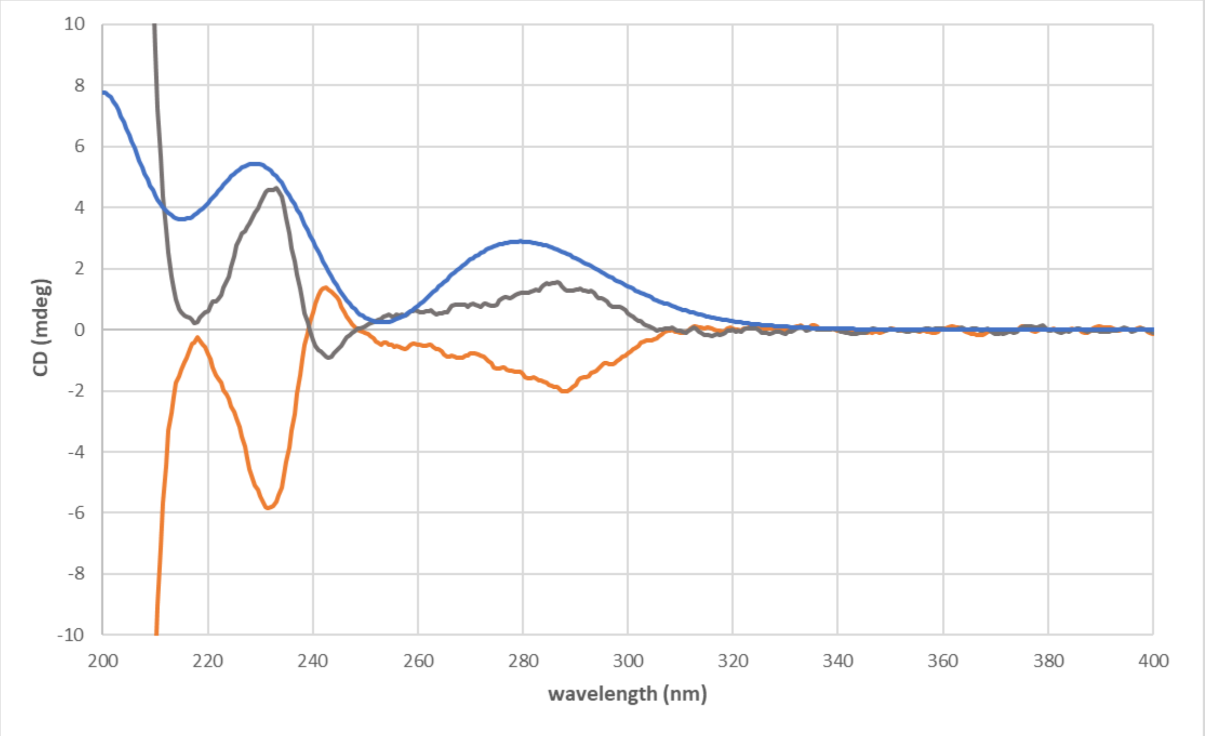

Fit between calculated and experimental IR and VCD spectra

An in-house program was used to generate a Boltzmann weighted average spectrum for the two conformations within the 5 $kJmol^{-1}$ limit and to fit Lorentzian line shapes (12 cm^{-1} line width) to the computed spectra applying a linear scaling factor of 0.98. The fit between calculated and experimental IR and VCD data is shown in Figure S2. There is excellent agreement between calculated and experimental spectra, with only small differences between calculated and experimental frequencies noted for a few peaks. Compound **17** can therefore be equated to the 6*S*,8*R*,9*S* stereoisomer with very high confidence.

Figure S2. Comparison of experimental (green) and calculated (red) VCD (top) and infra-red spectra (bottom) for compound **17** and the 6*S*,8*R*,9*S* stereoisomer of pilemartine A. The blank spectra recorded for $CDCl_3$ have been subtracted from both experimental spectra.

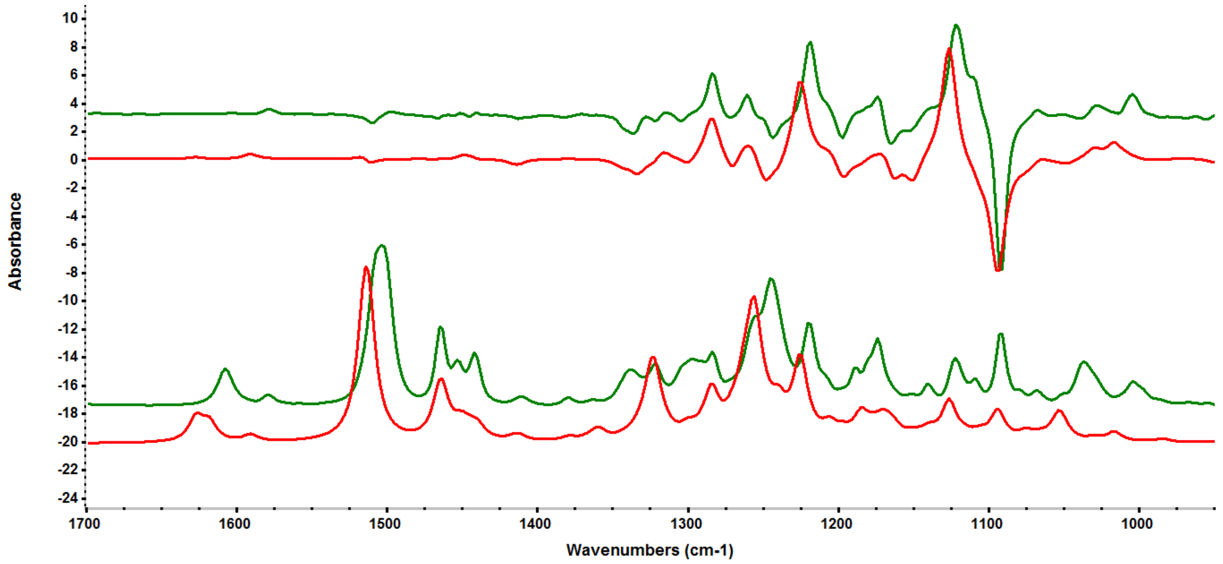

^{1}H and ^{13}C NMR Spectra of Novel Compounds

8-(3,5-Bis(trifluoromethyl)phenyl)-1,3,5,7-tetramethyl-2,4,6-trioxa-8-phosphaadamantane

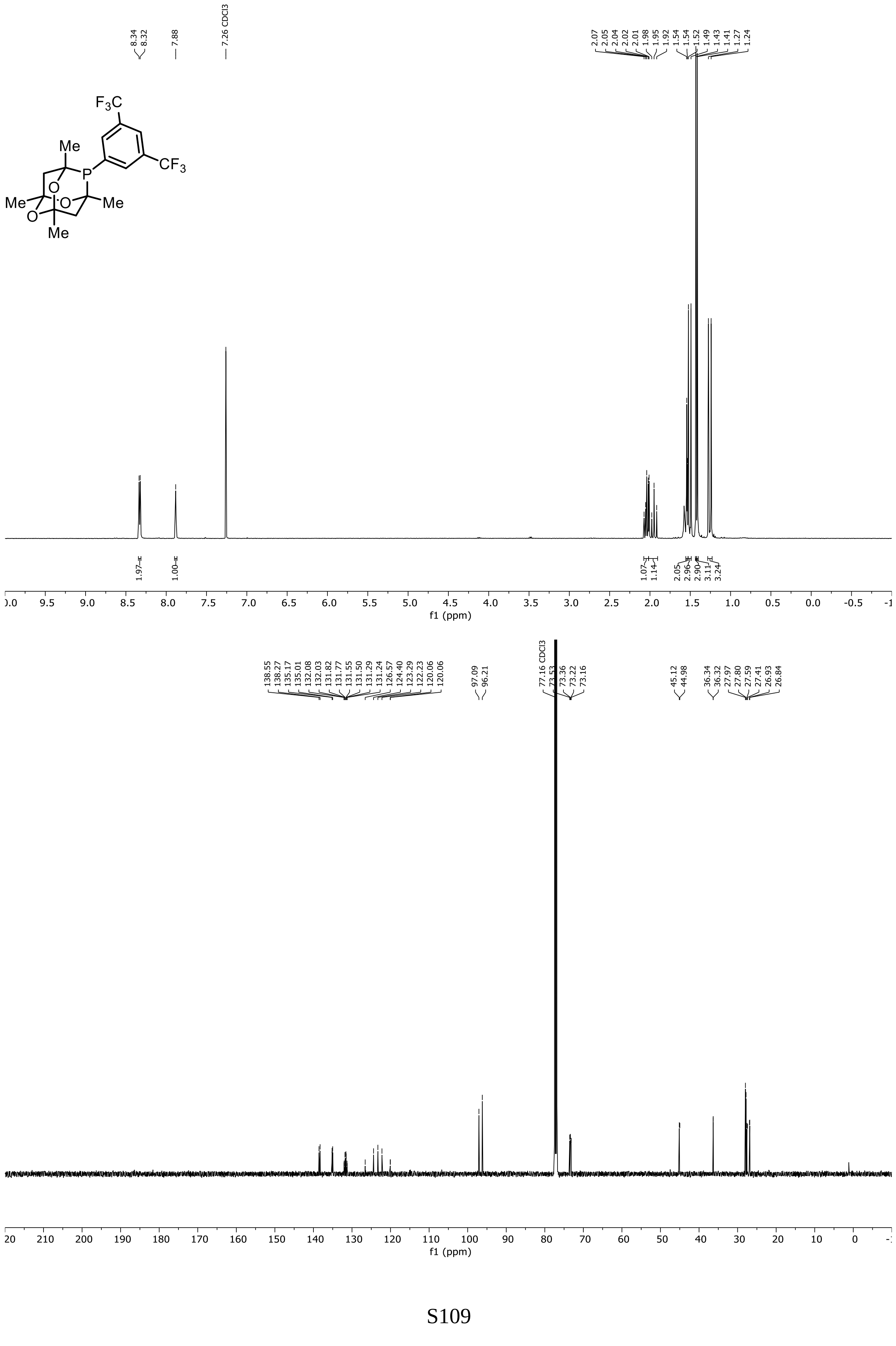

8-(3,5-Difluoro-4-nitrophenyl)-1,3,5,7-tetramethyl-2,4,6-trioxa-8-phosphaadamantane

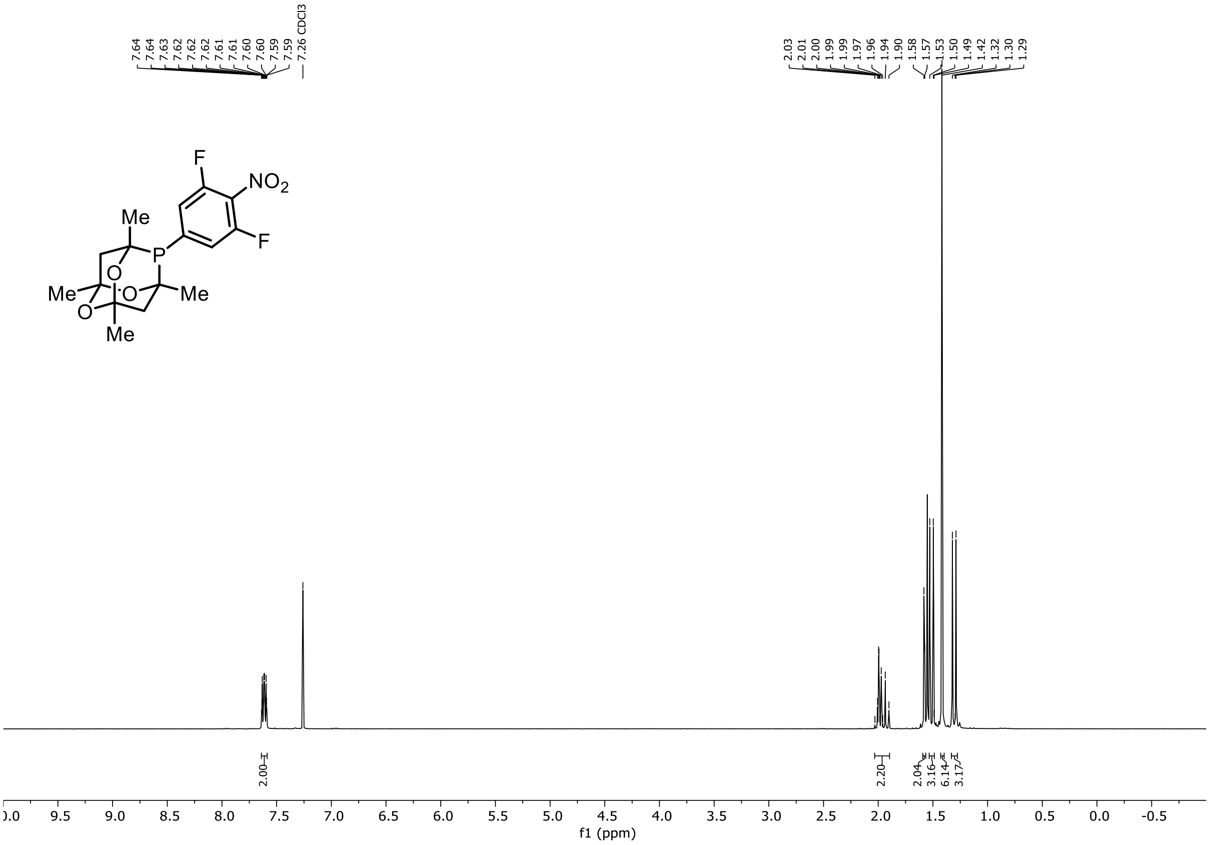

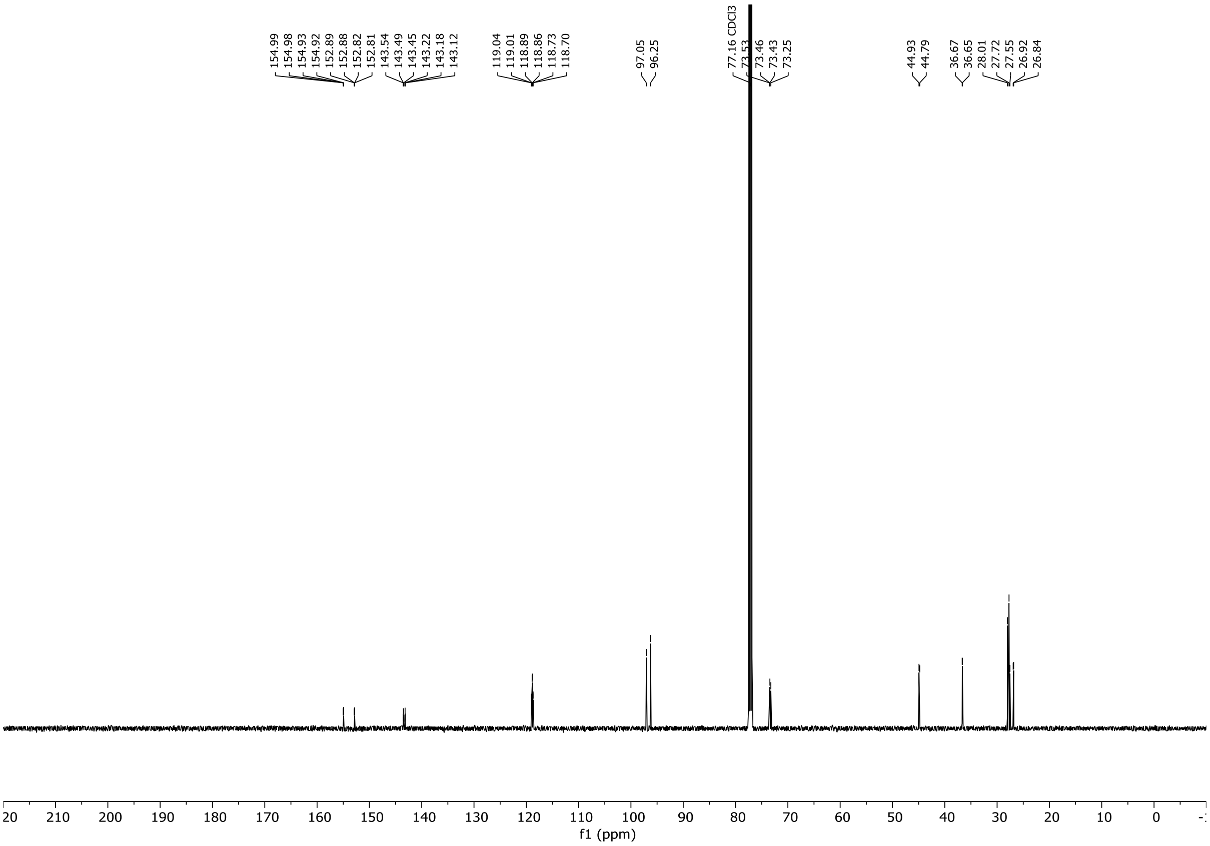

8-(3,5-Dinitrophenyl)-1,3,5,7-tetramethyl-2,4,6-trioxa-8-phosphaadamantane

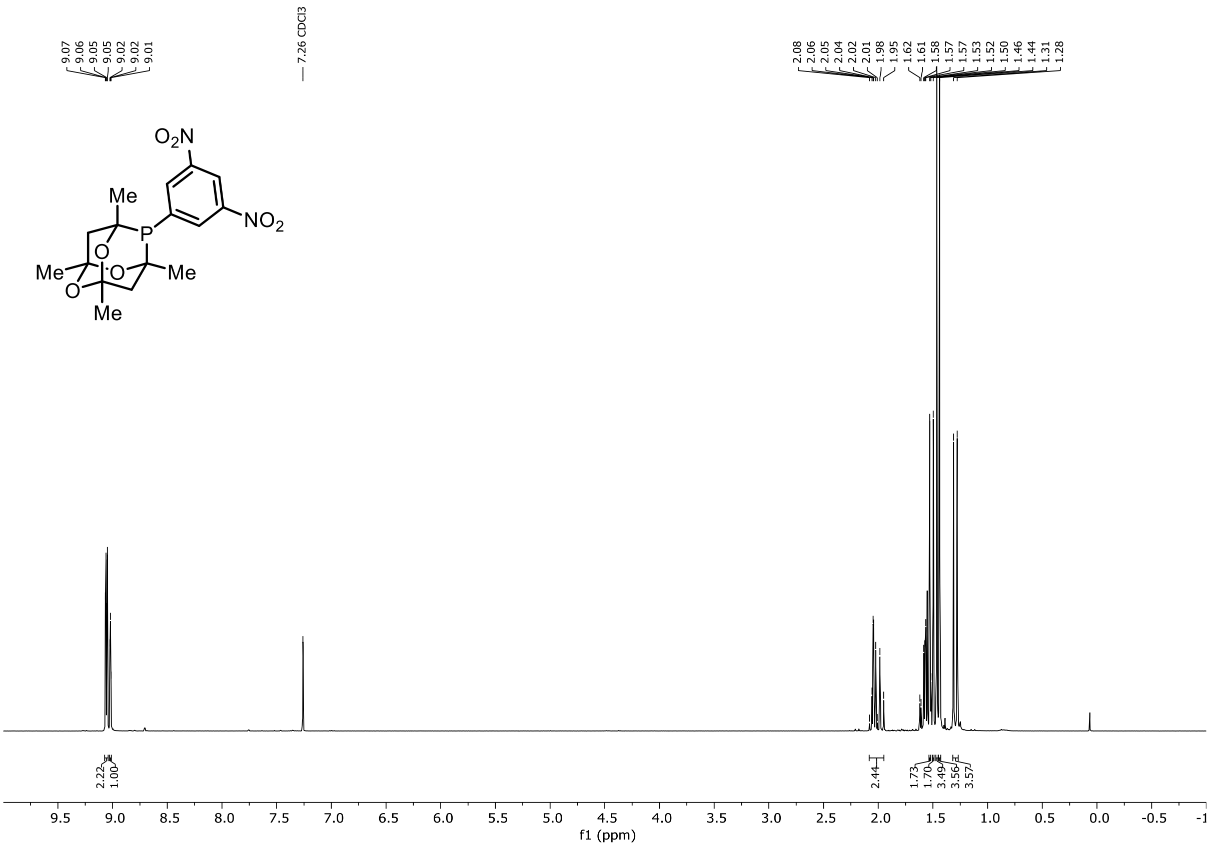

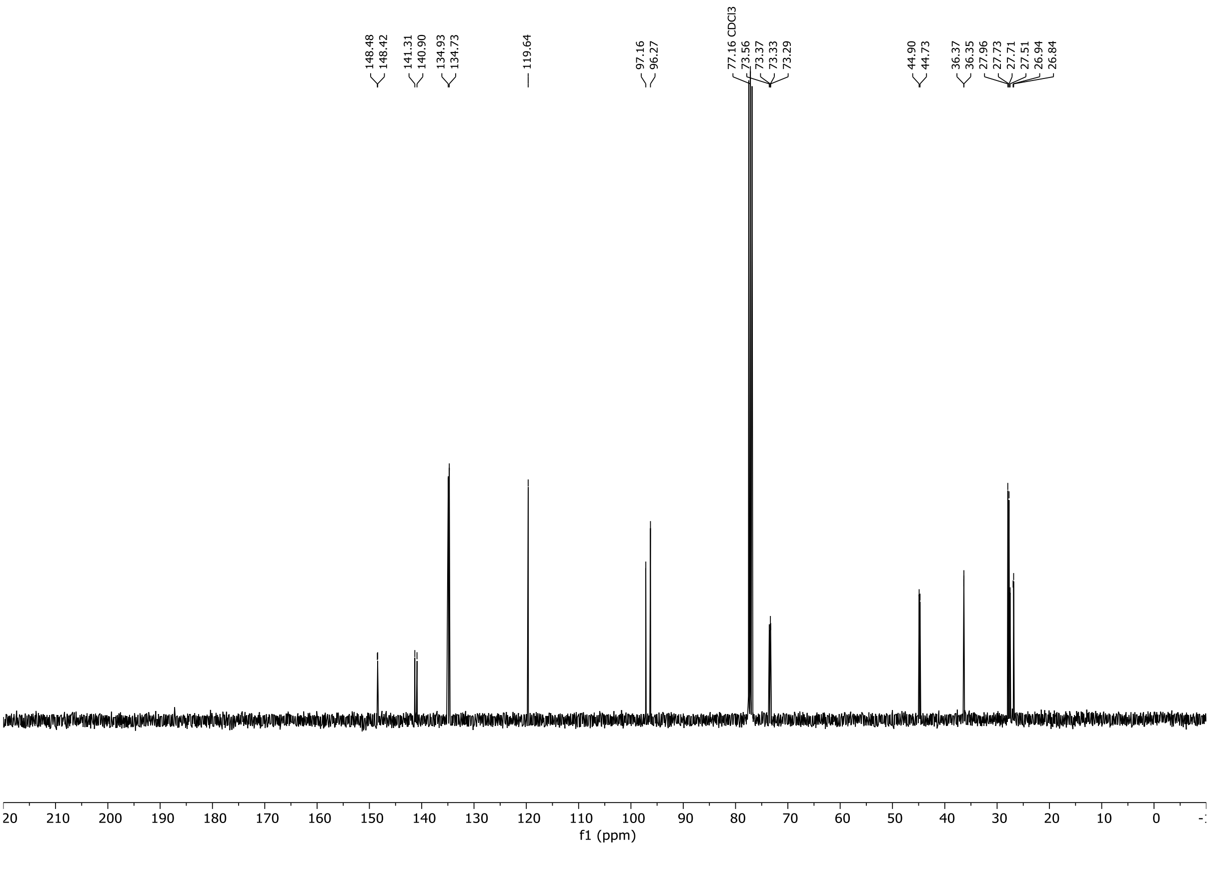

Sodium 2-phenoxybenzoate

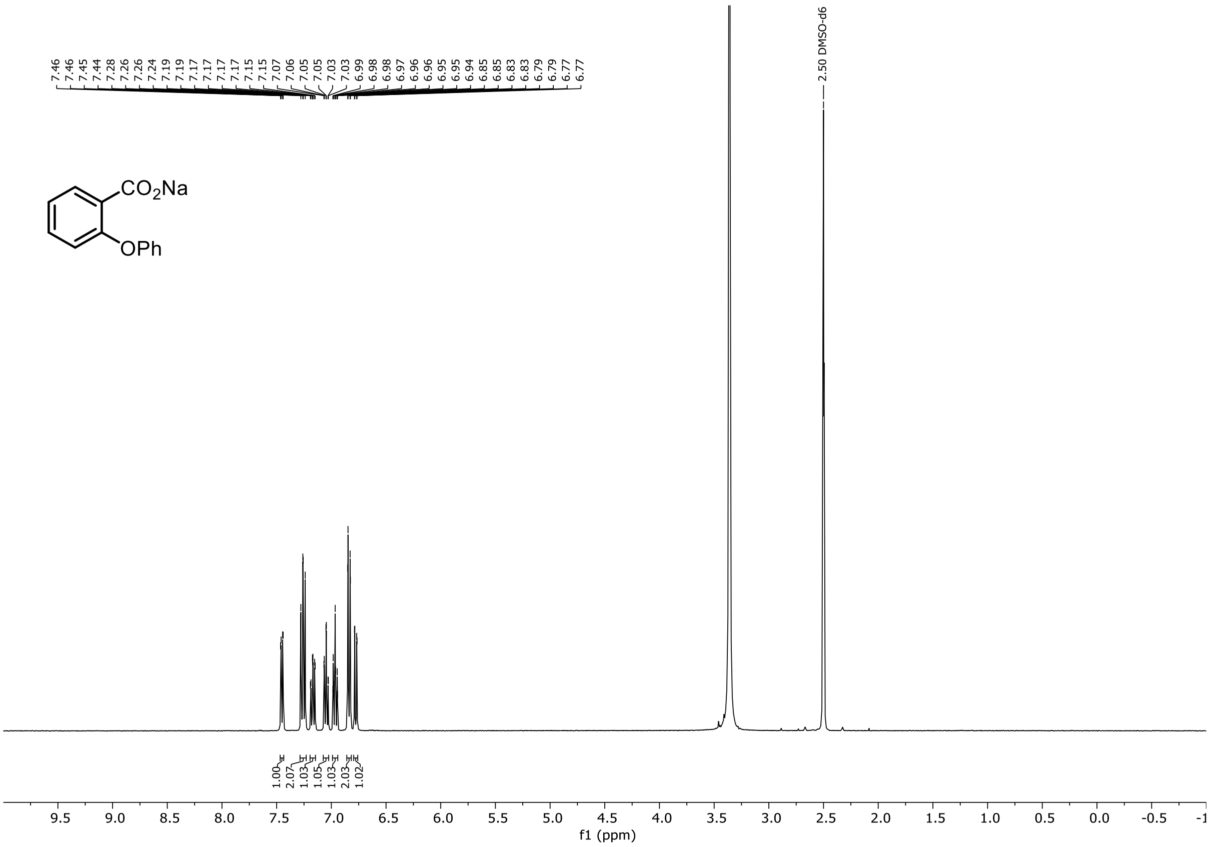

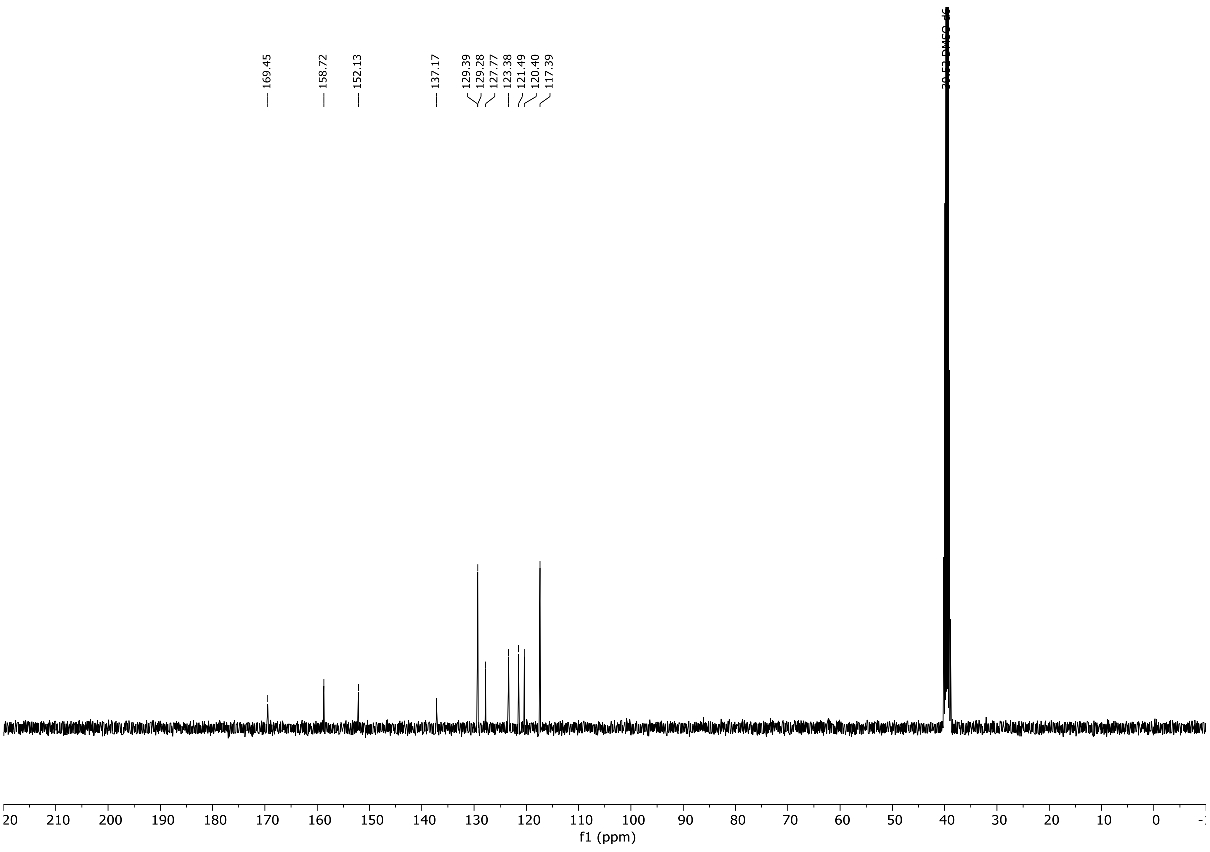

Sodium 2-isopropoxybenzoate

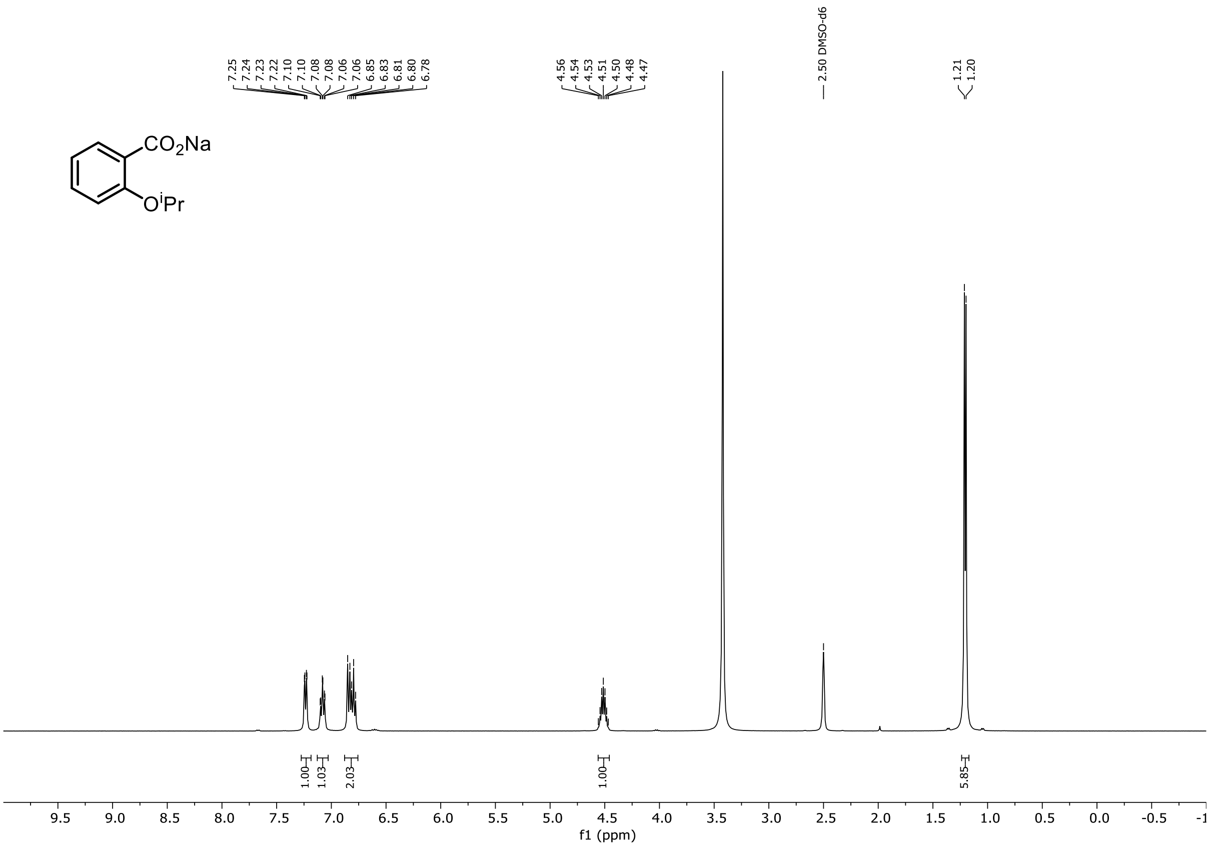

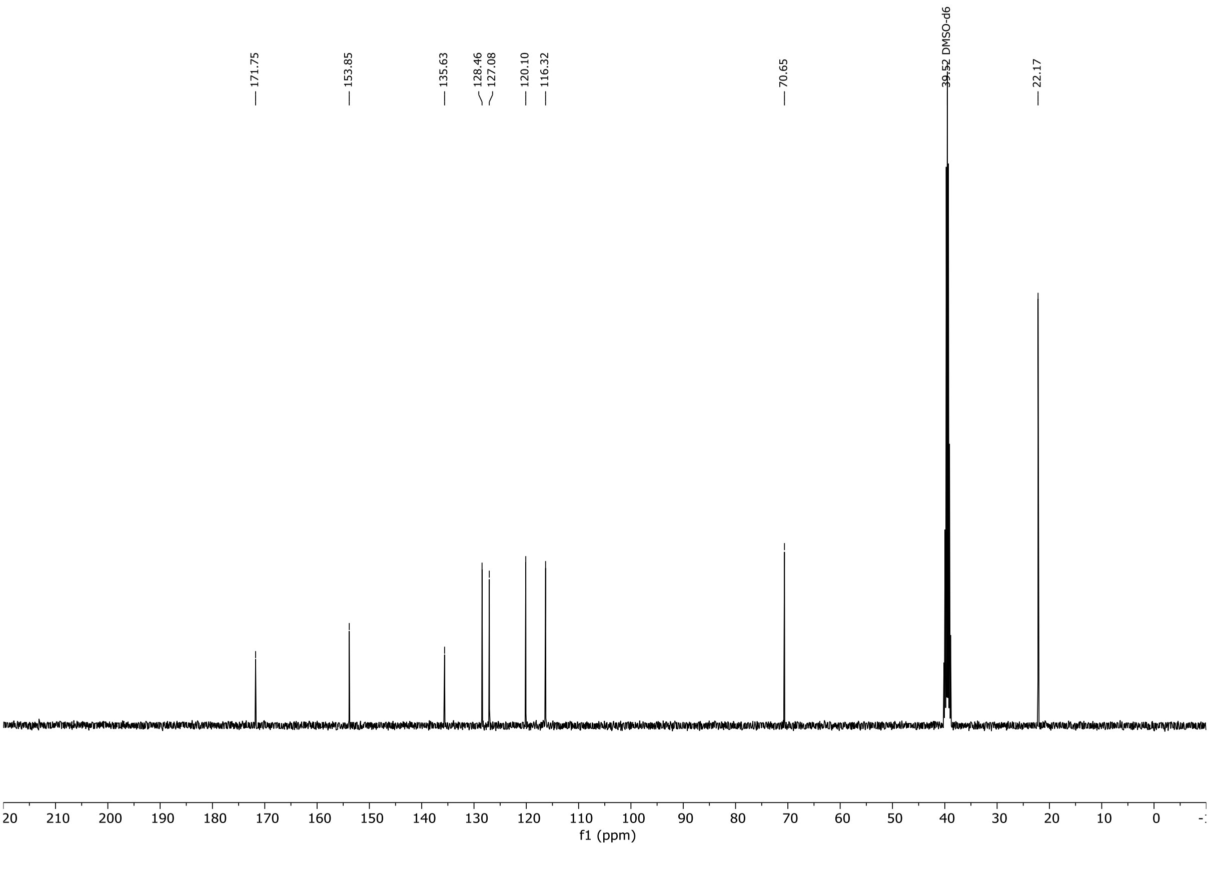

Sodium 2-(dimethylamino)benzoate

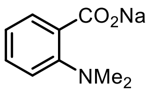

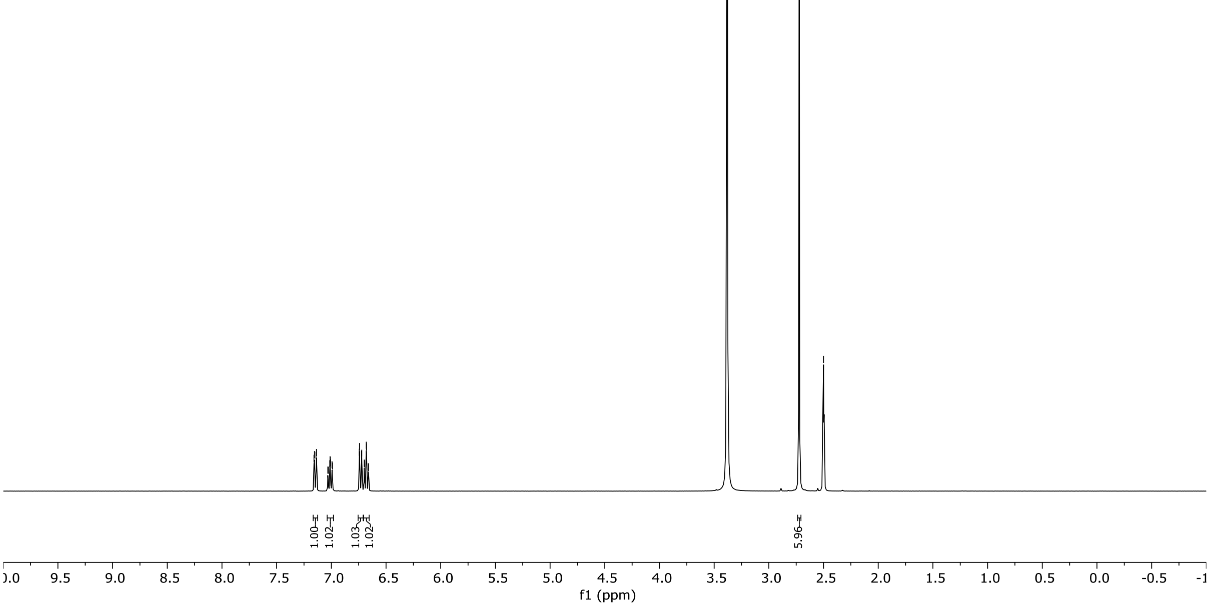

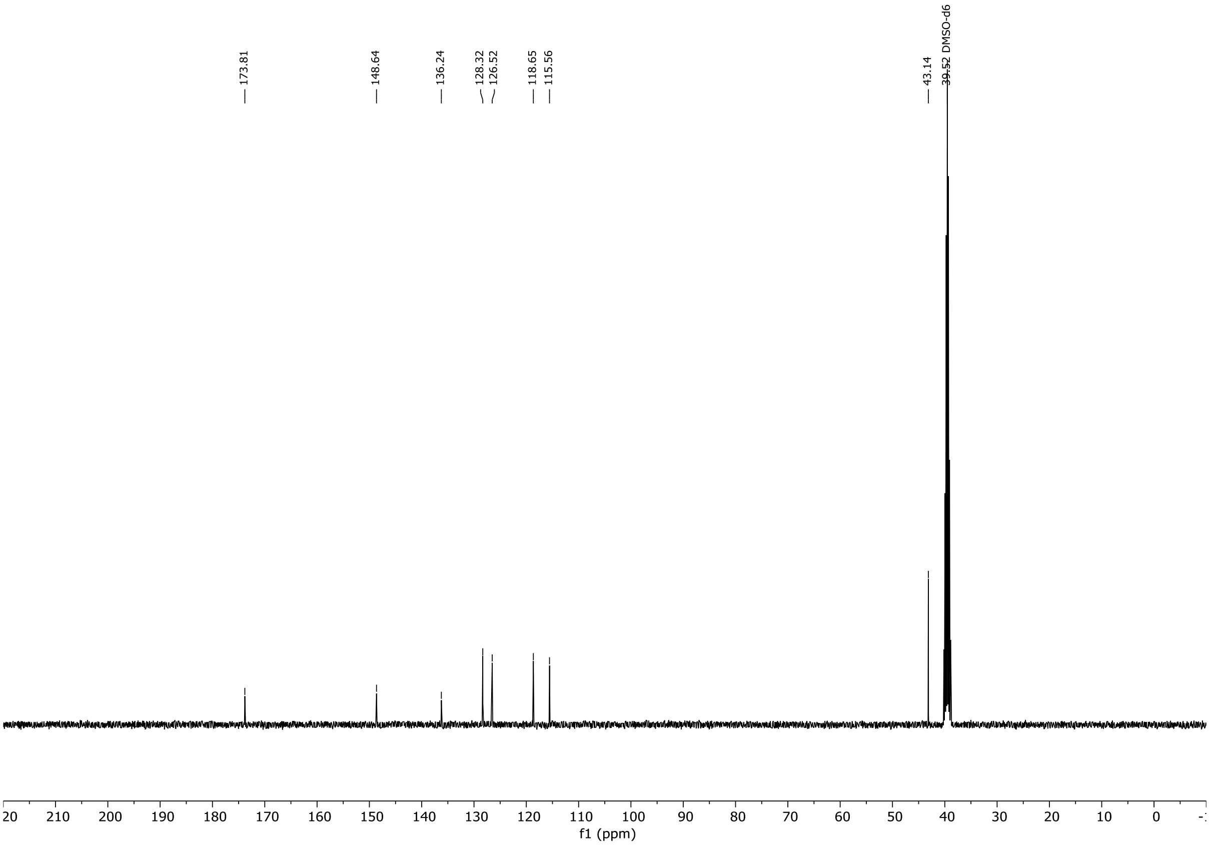

Sodium 2-(methylthio)benzoate

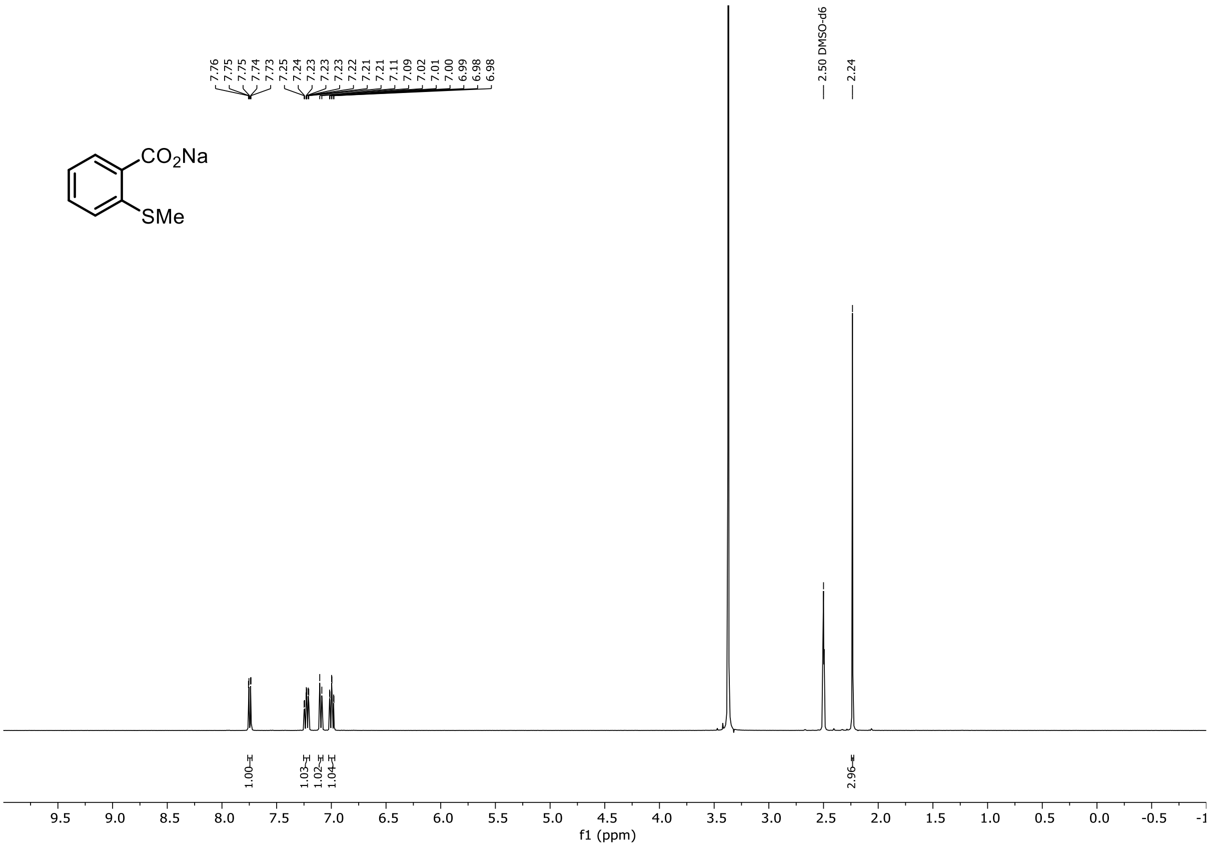

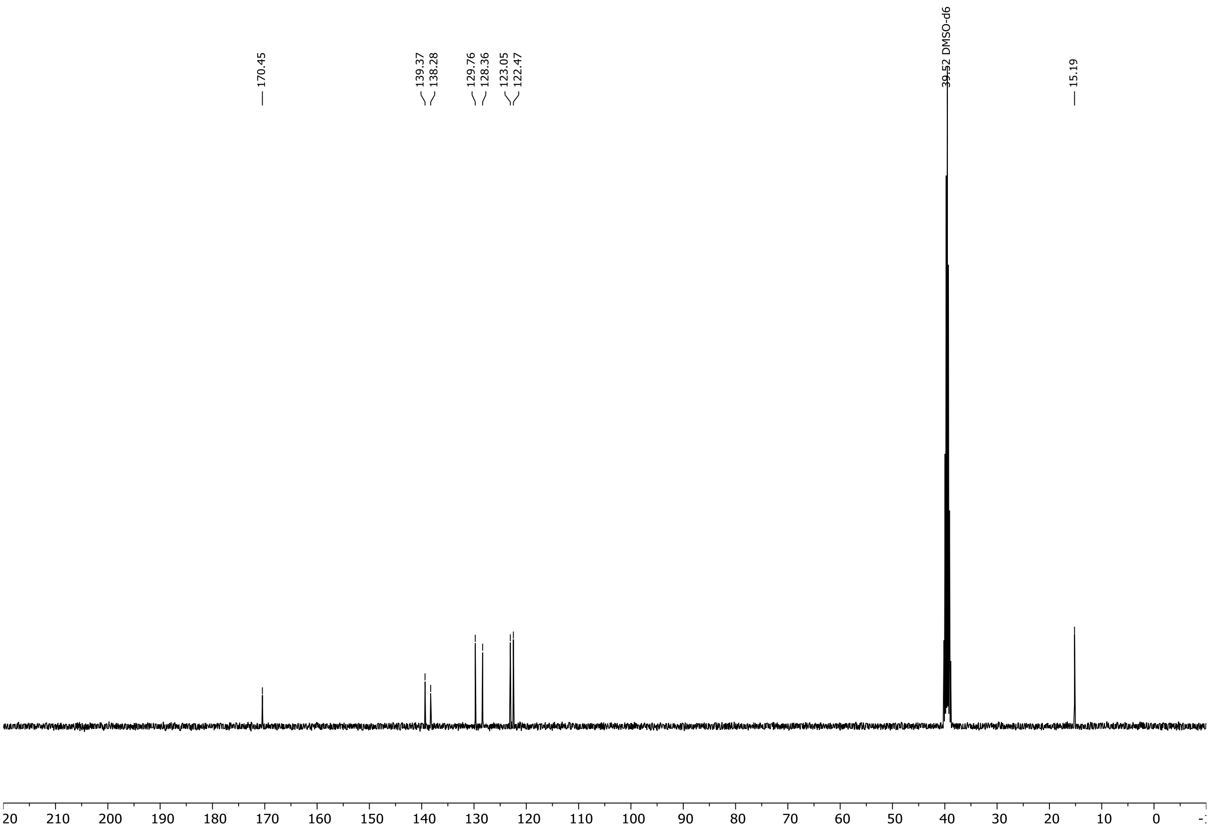

Sodium 2,4-dimethoxybenzoate

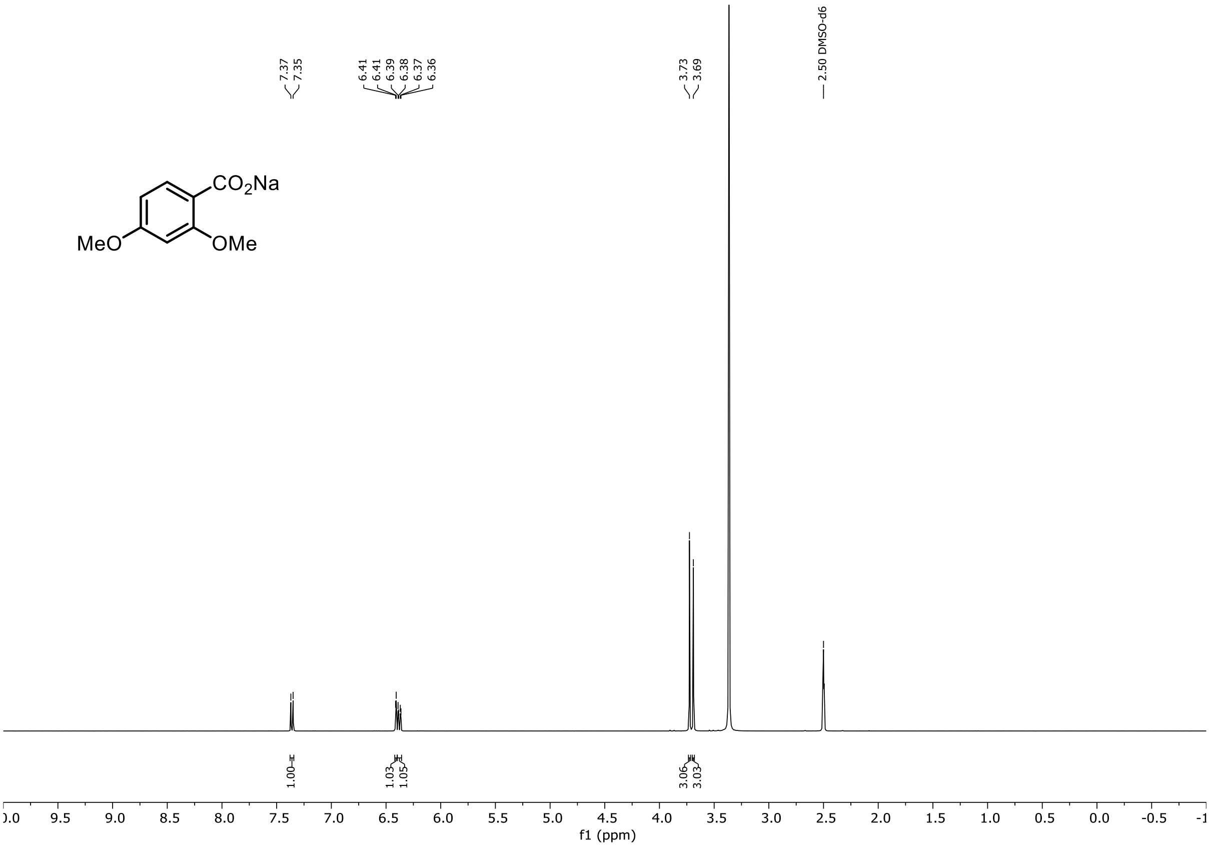

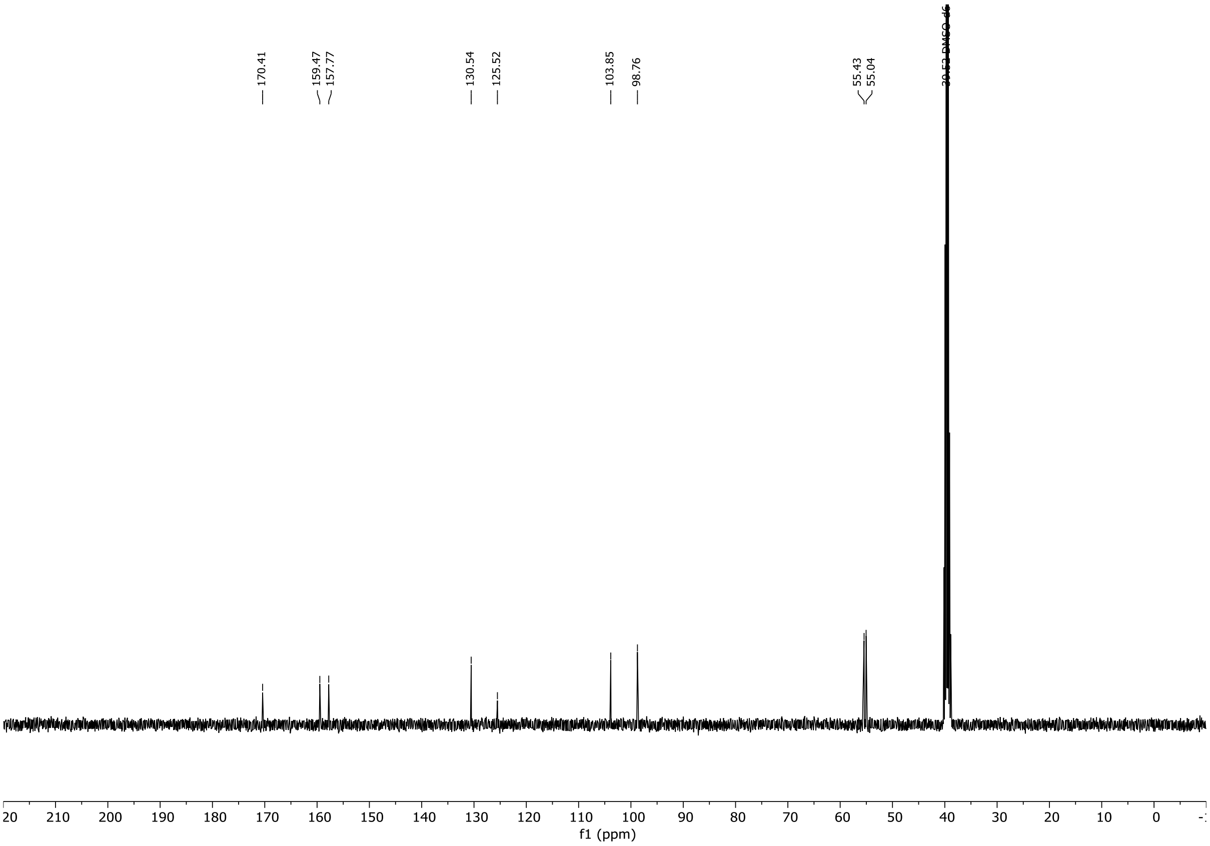

Sodium 2-oxidobenzoate

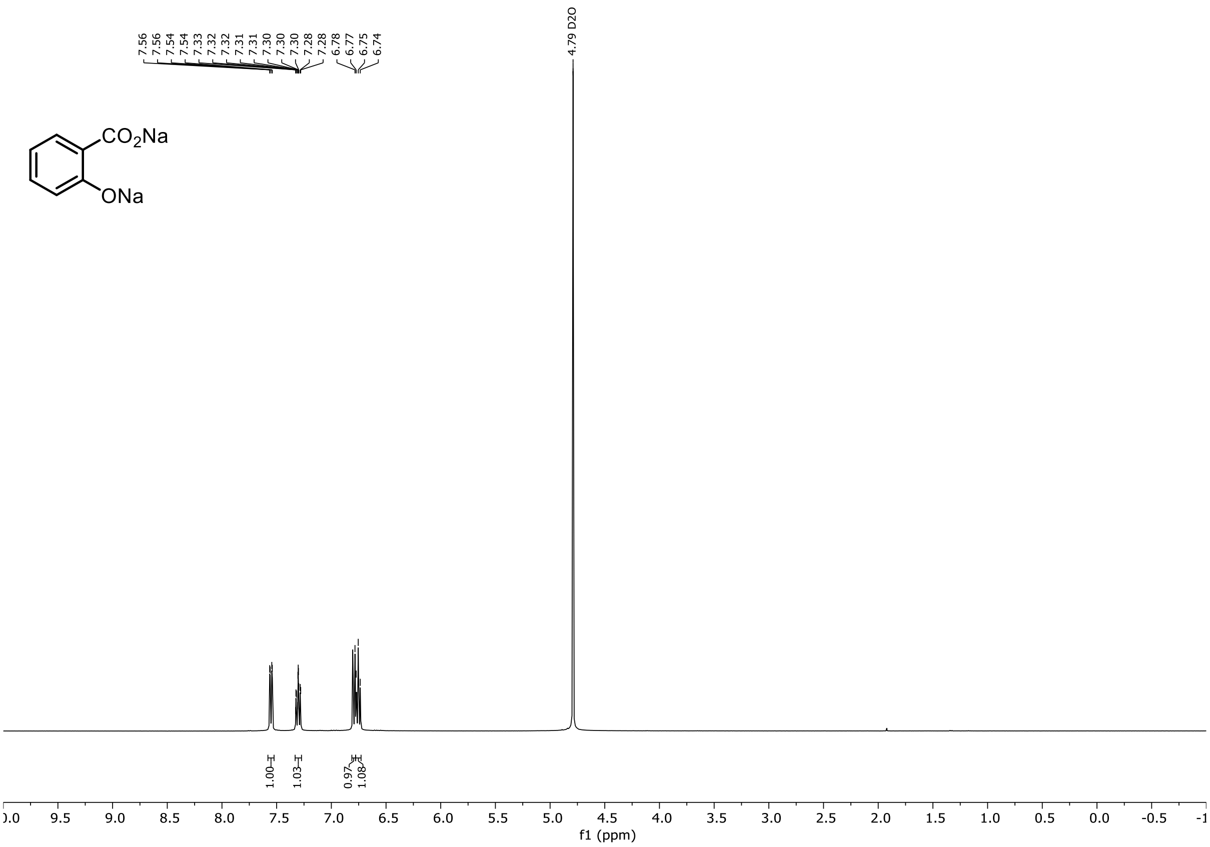

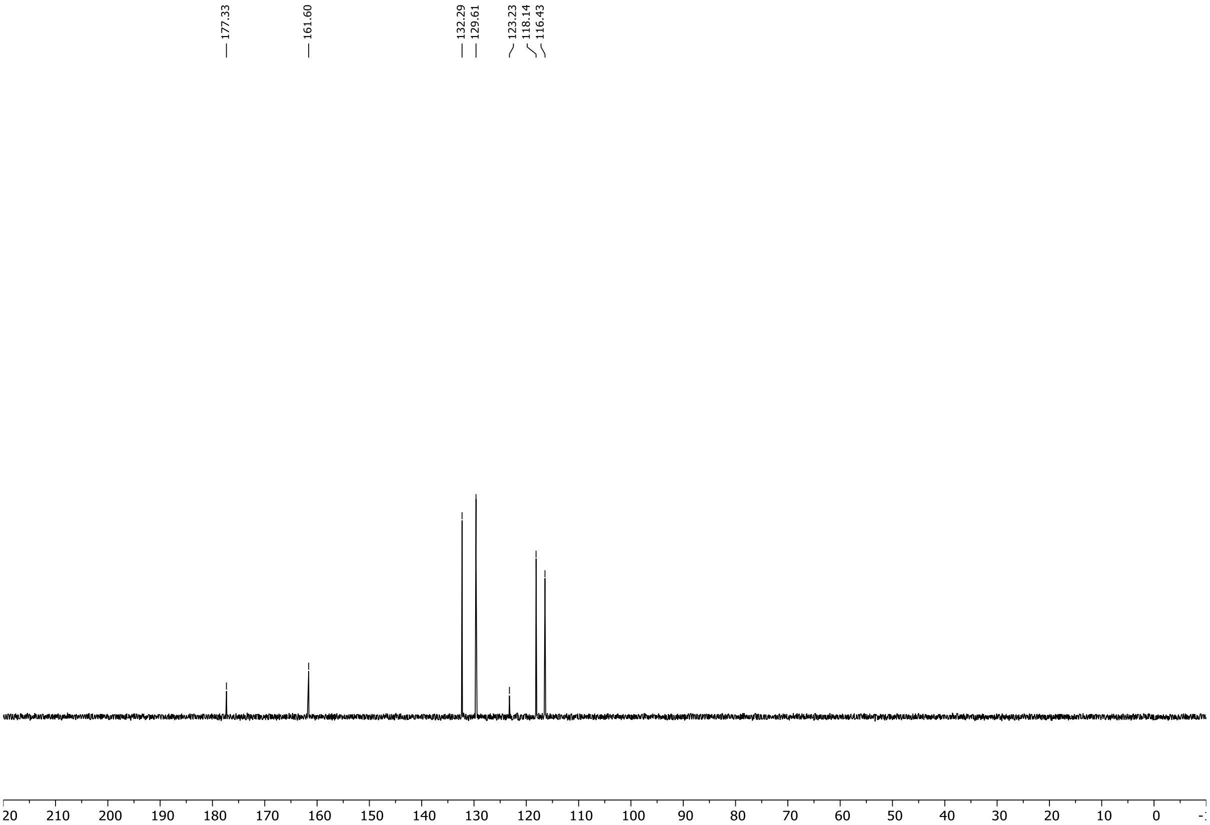

Sodium 8-methoxy-1-naphthoate

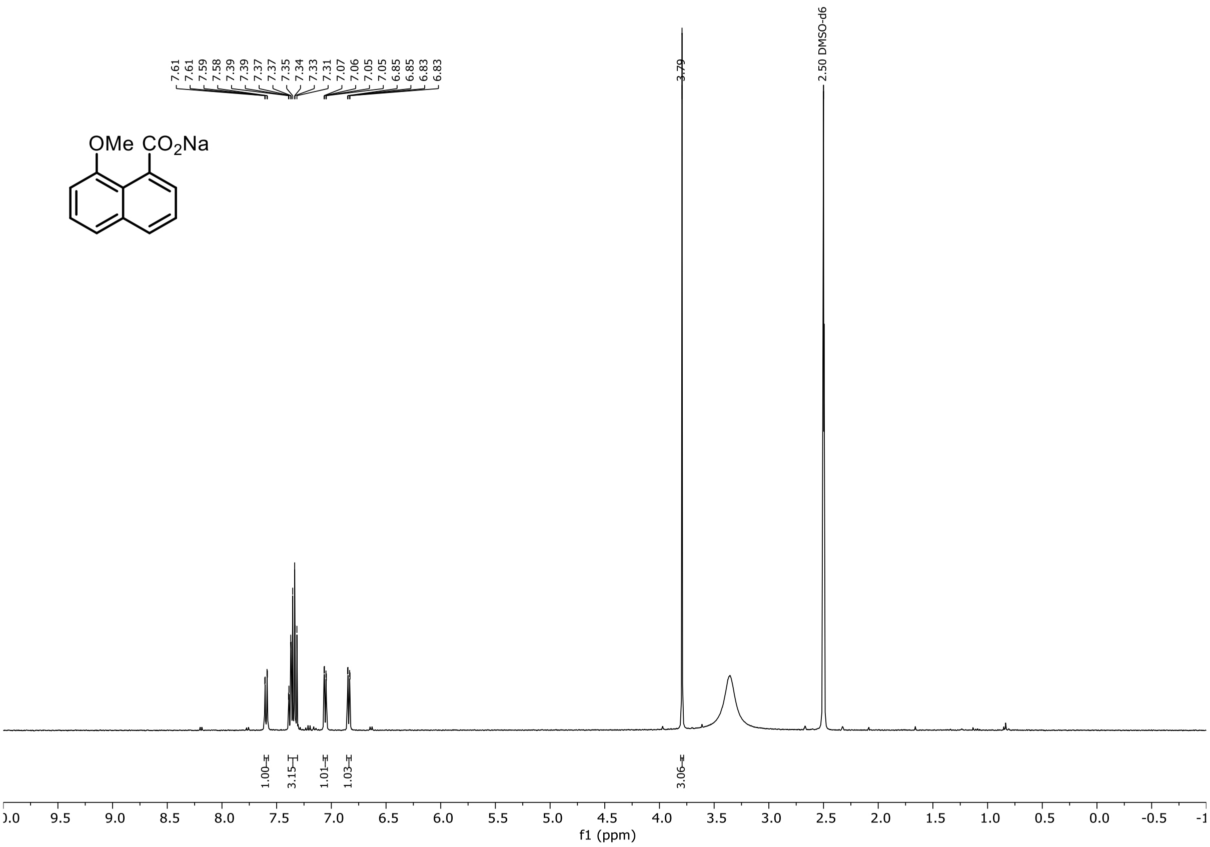

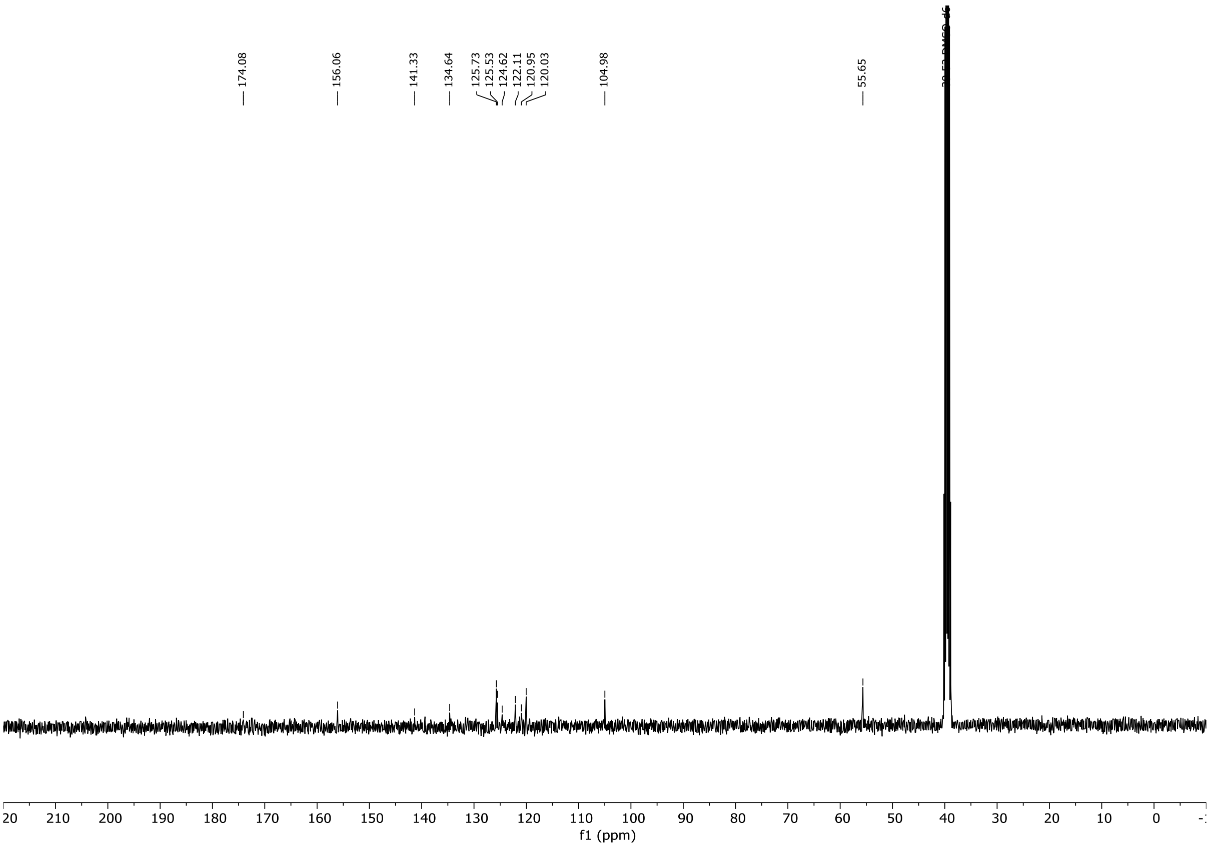

Methyl (4-methyl-3-phenylpent-4-en-1-yl)((perfluorobenzoyl)oxy)carbamate (1a)

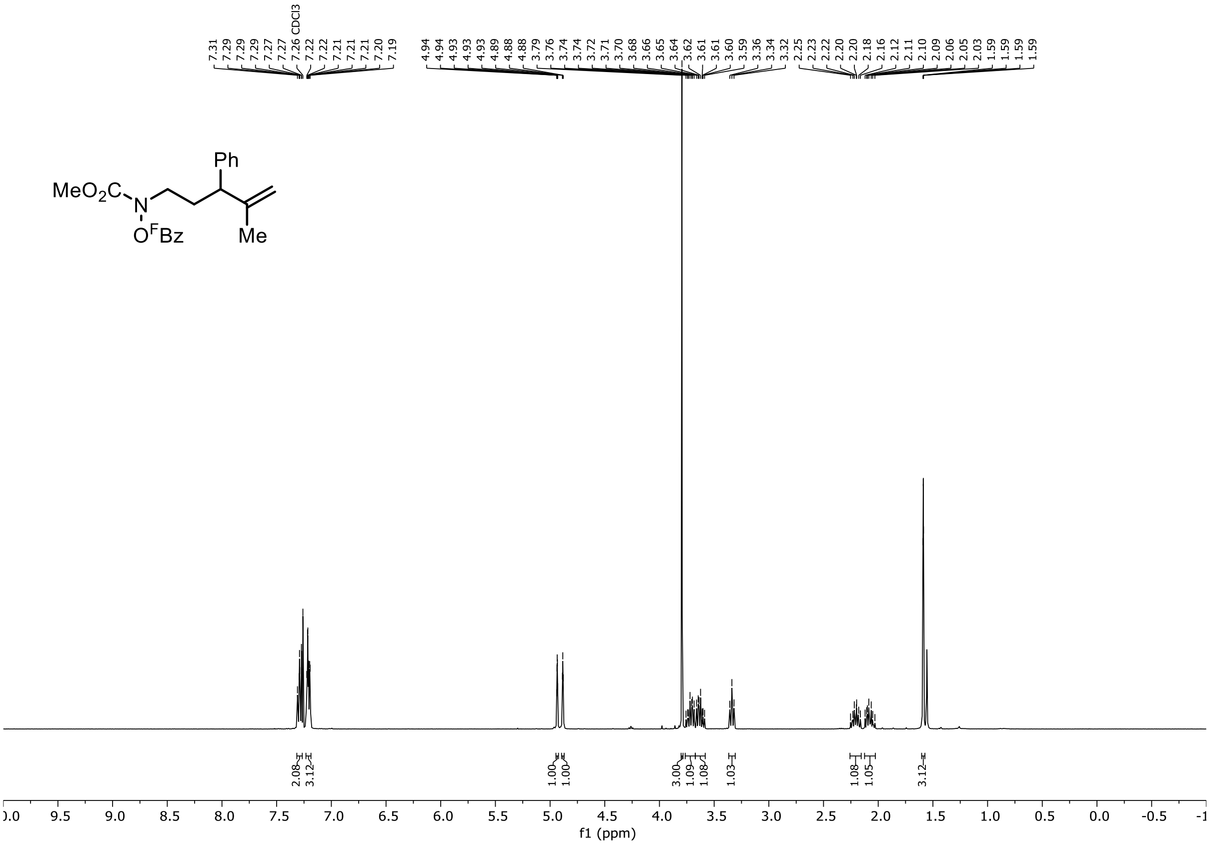

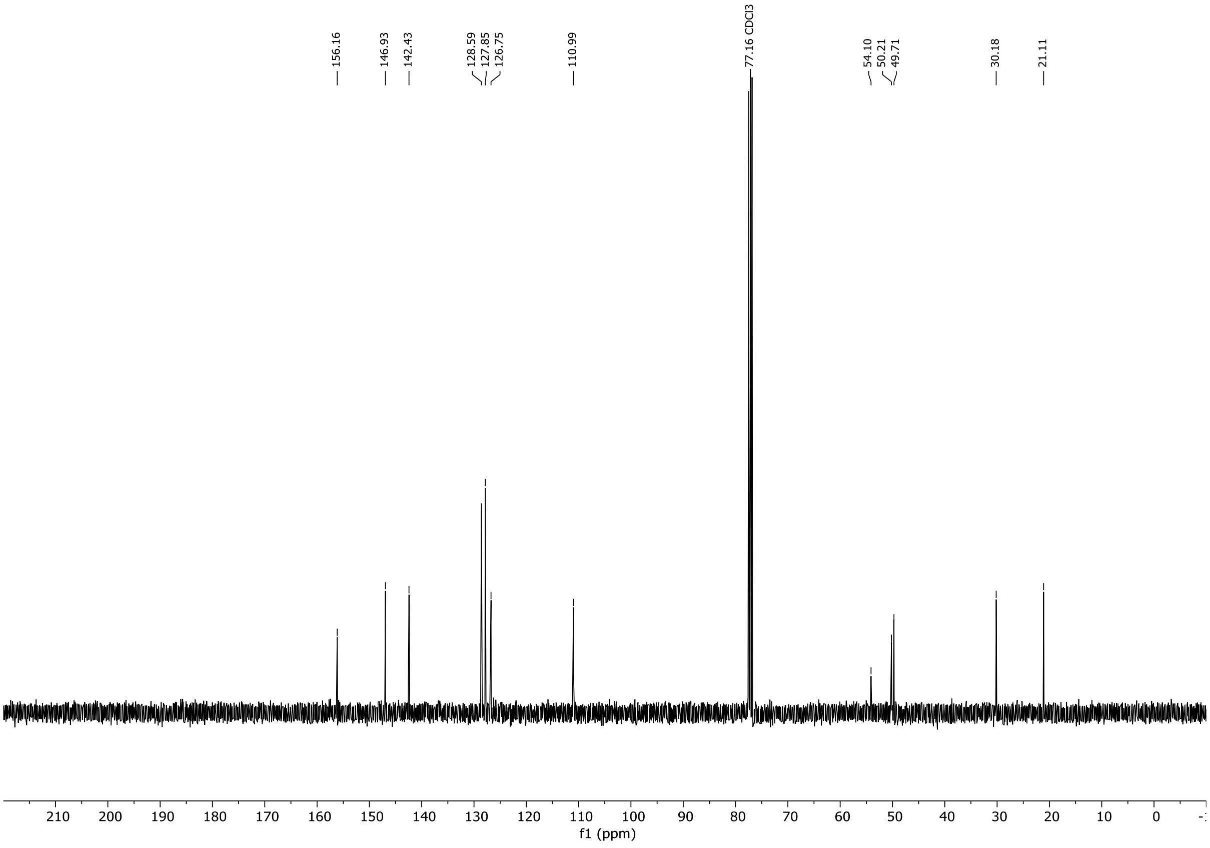

Methyl (benzoyloxy)carbamate

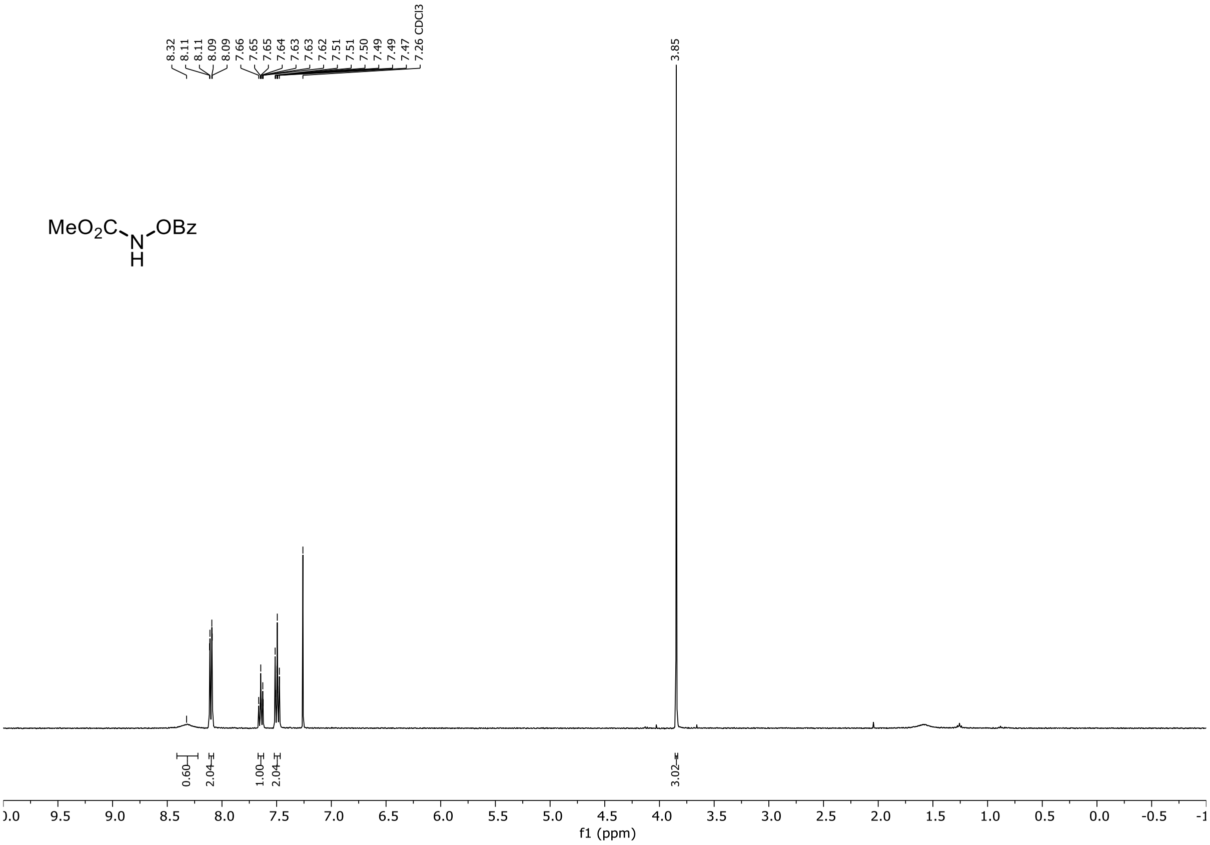

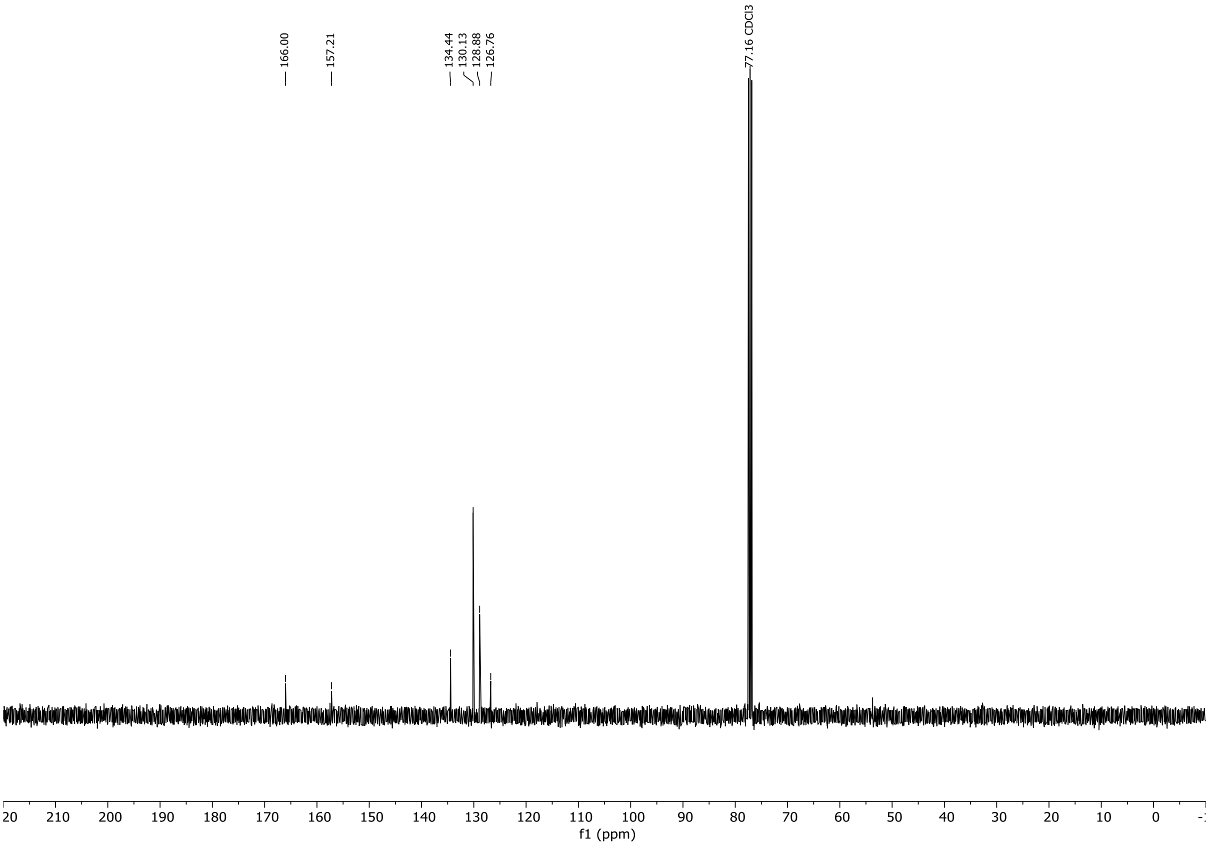

Methyl (benzoyloxy)(4-methyl-3-phenylpent-4-en-1-yl)carbamate (1a')

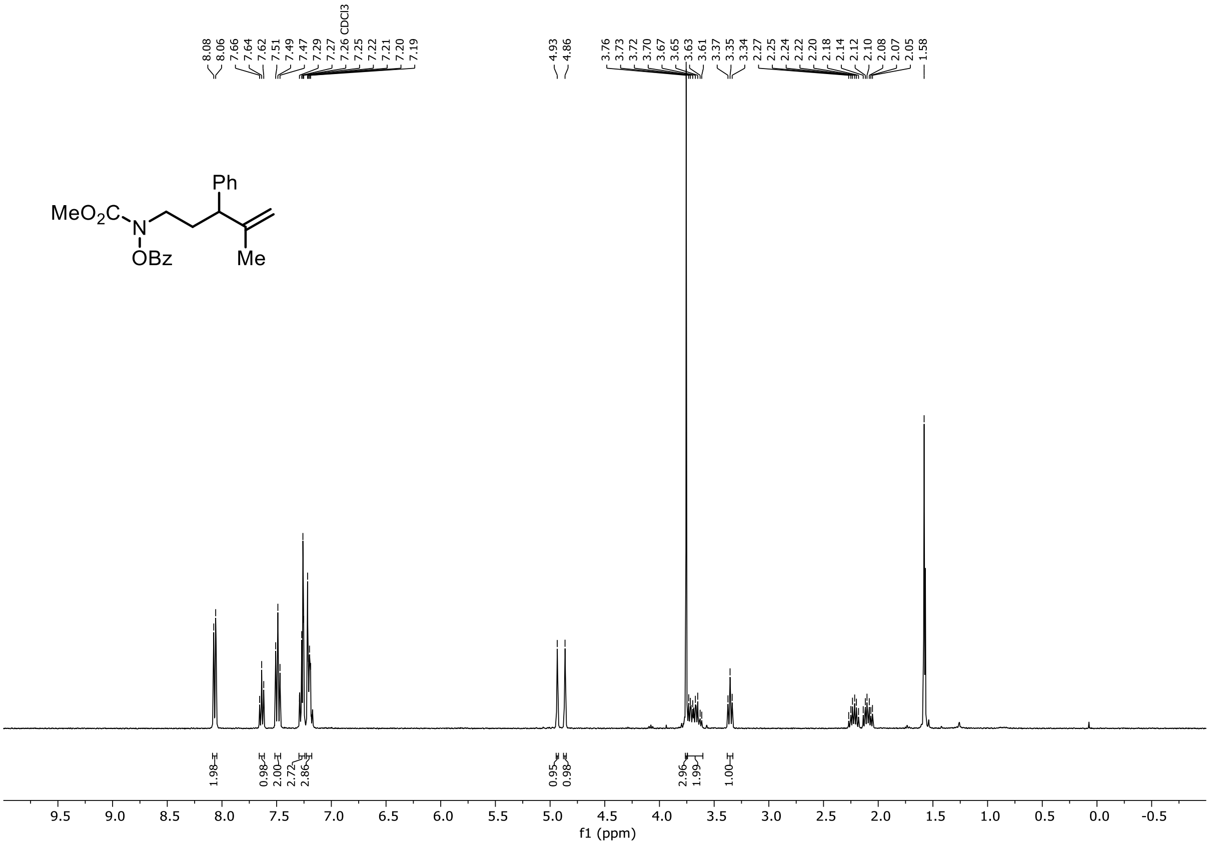

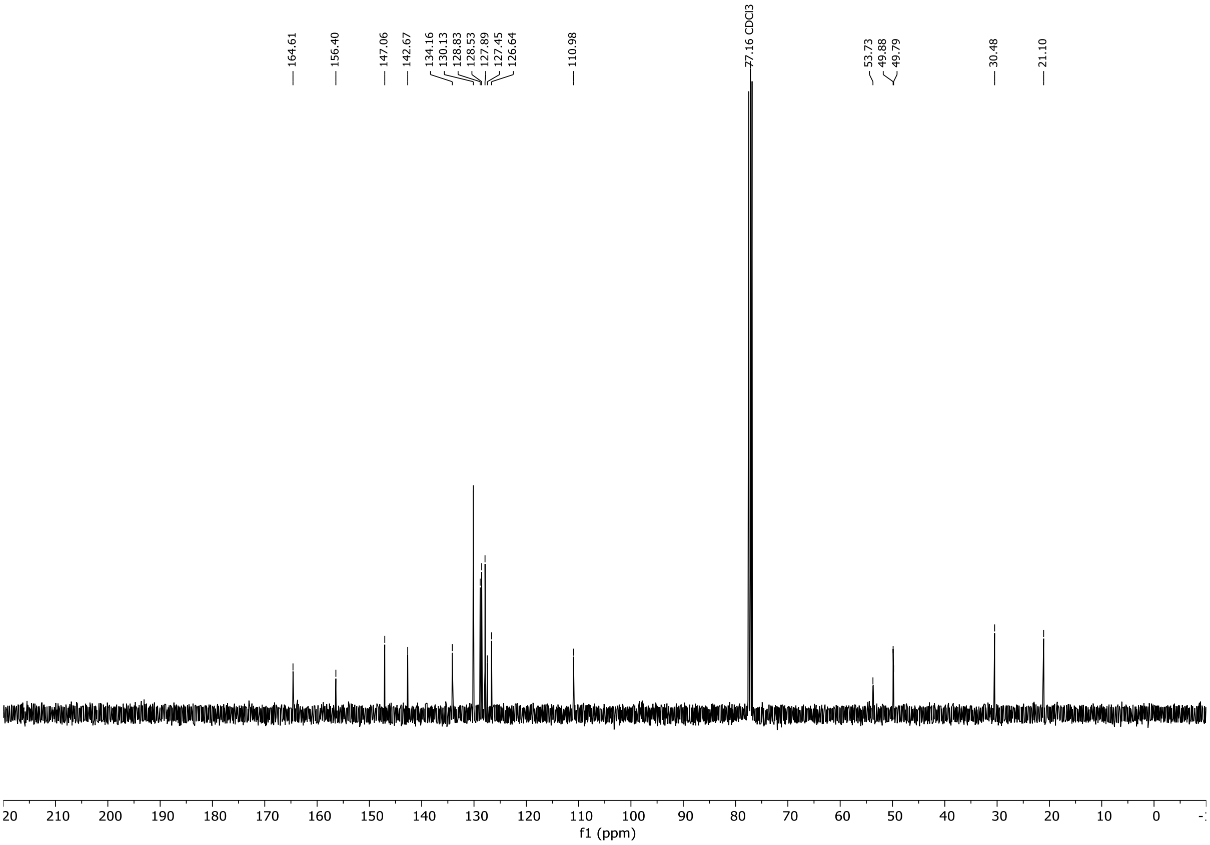

Benzyl (4-methyl-3-phenylpent-4-en-1-yl)(tosyloxy)carbamate (1a'')

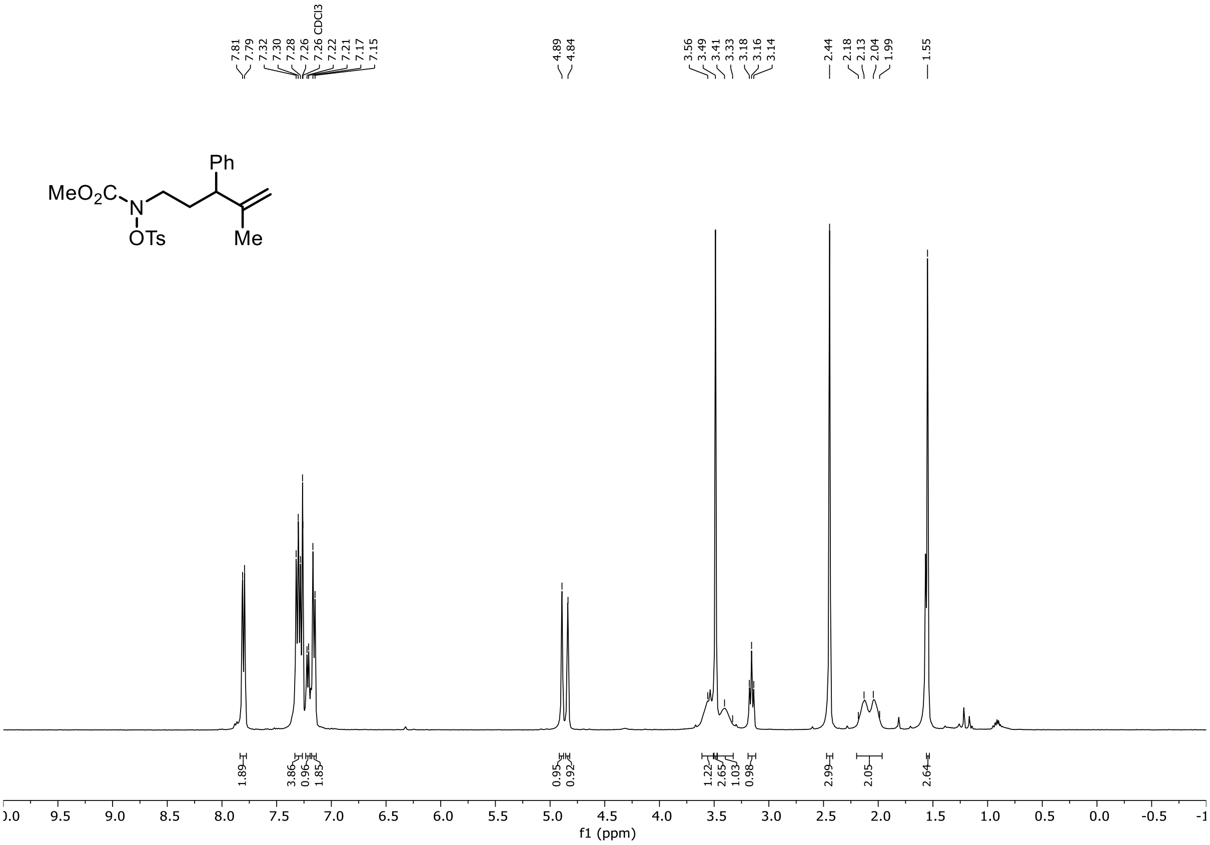

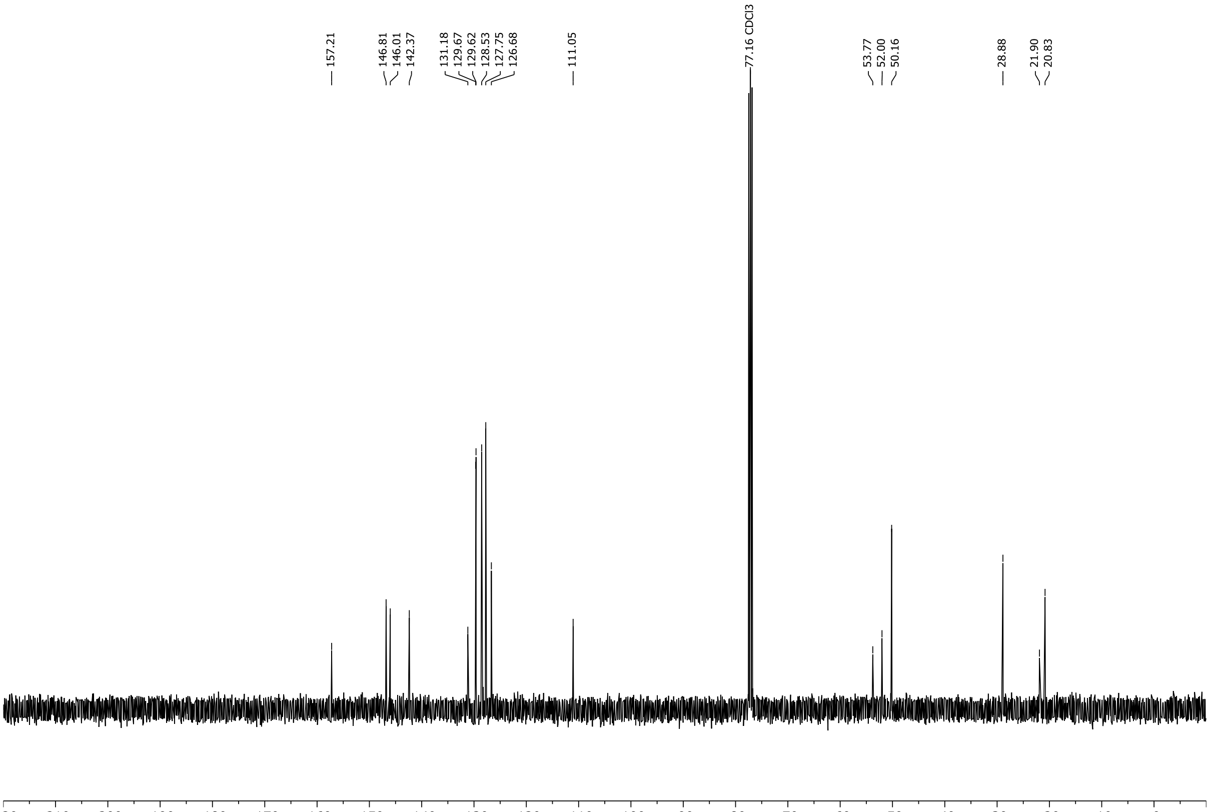

4-Methyl-*N*-(4-methyl-3-phenylpent-4-en-1-yl)-*N*-((perfluorobenzoyl)oxy)benzenesulfonamide

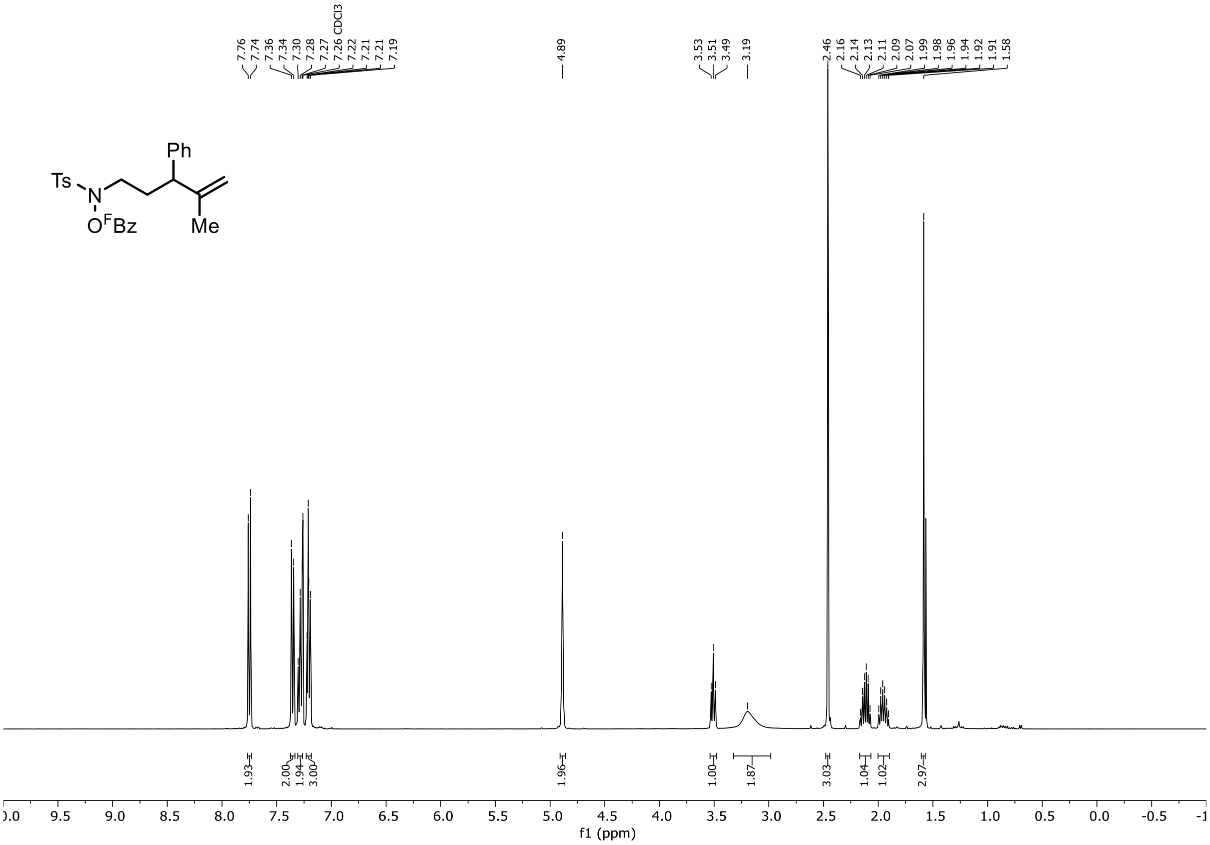

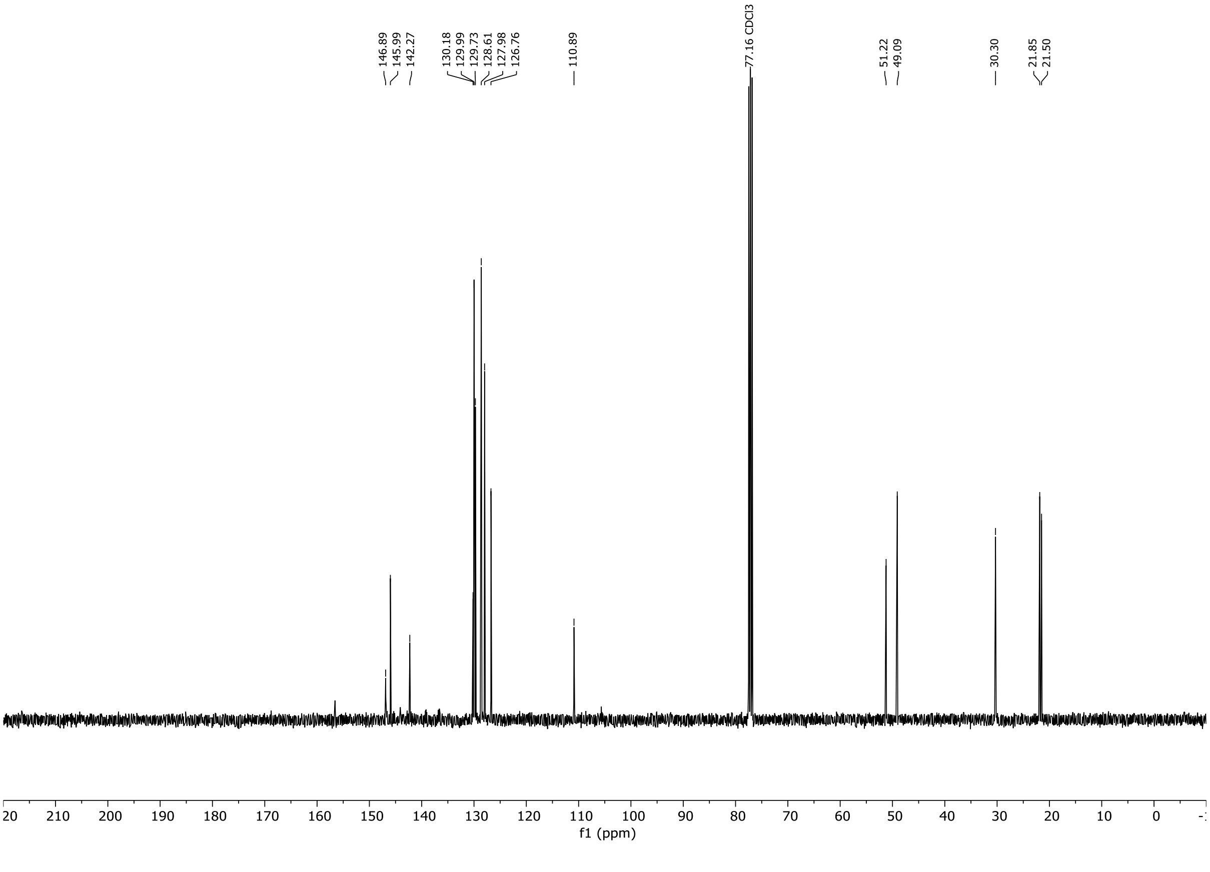

Methyl (4-methyl-3-phenylpent-4-en-1-yl)carbamate (2a)

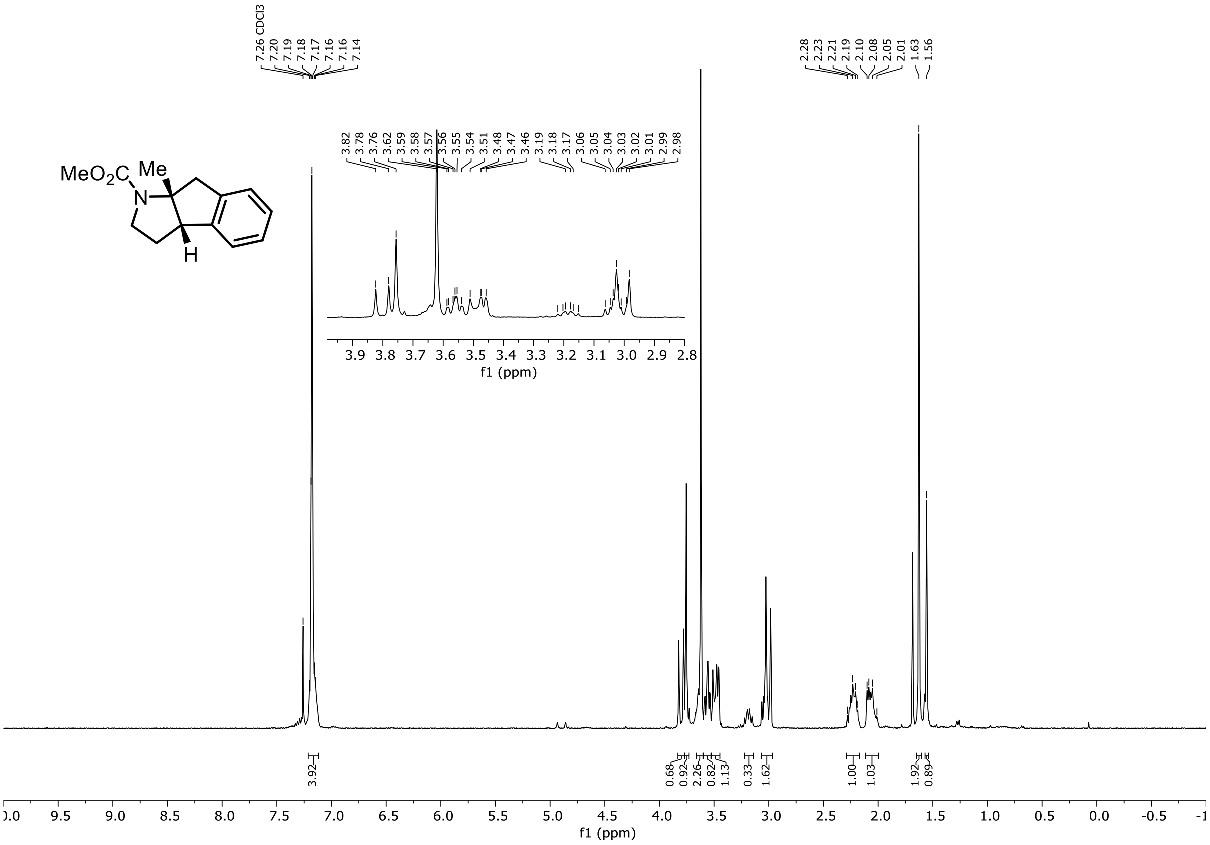

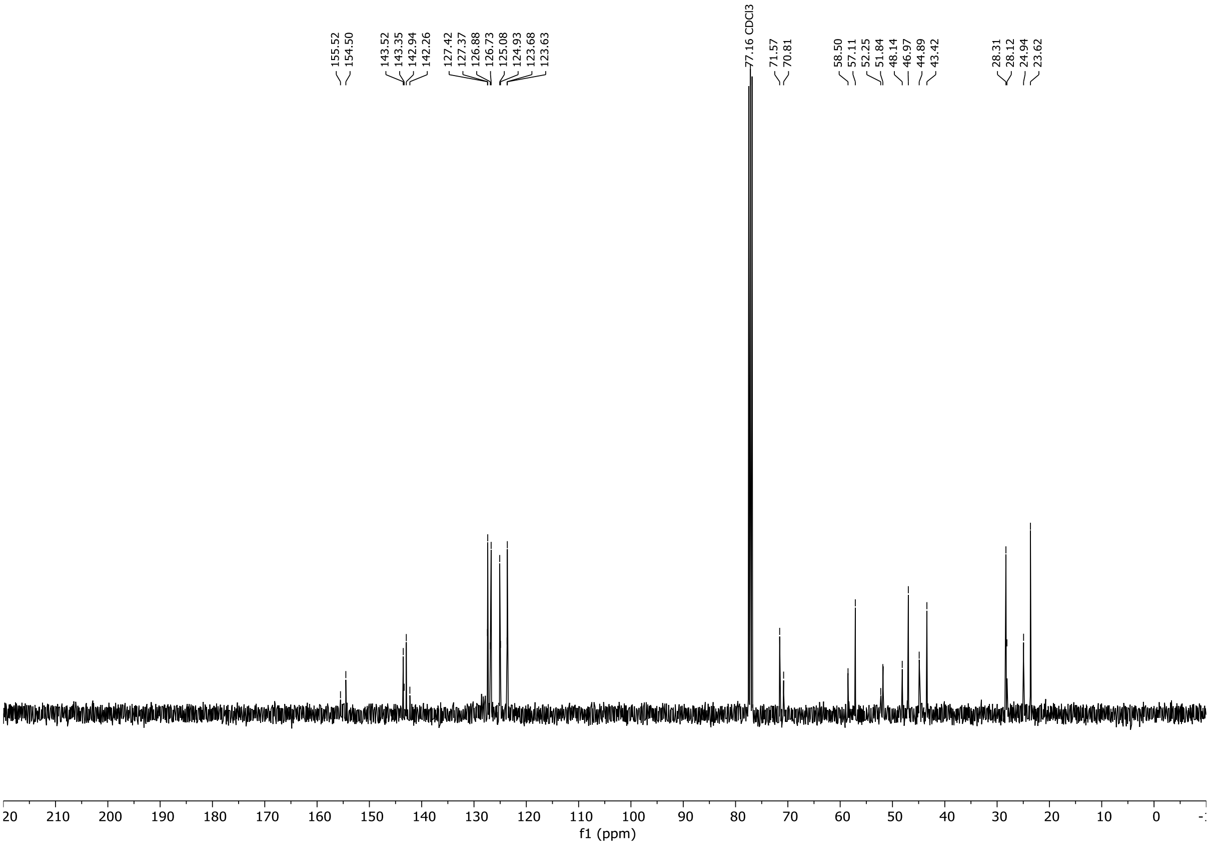

***tert*-Butyl (4-methyl-3-phenylpent-4-en-1-yl)((perfluorobenzoyl)oxy)carbamate (1b)**

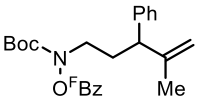

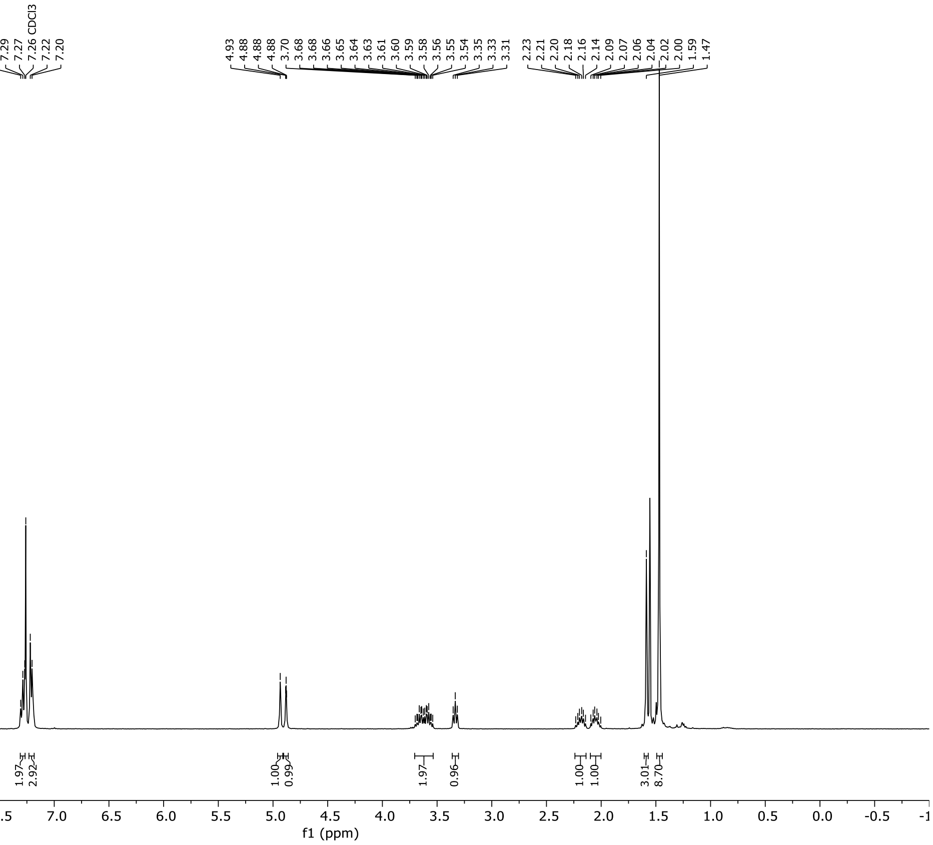

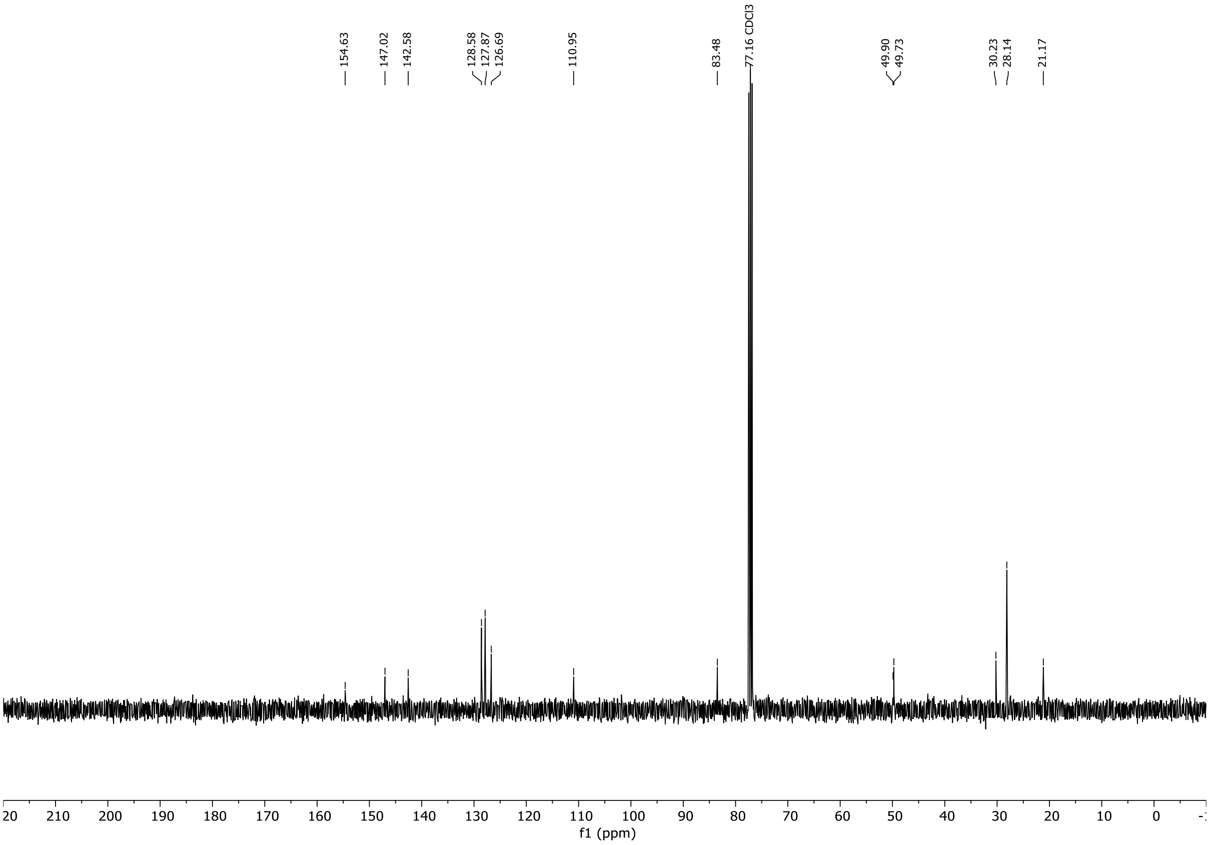

***tert*-Butyl 8a-methyl-3,3a,8,8a-tetrahydroindeno[2,1-*b*]pyrrole-1(2*H*)-carboxylate (2b)**

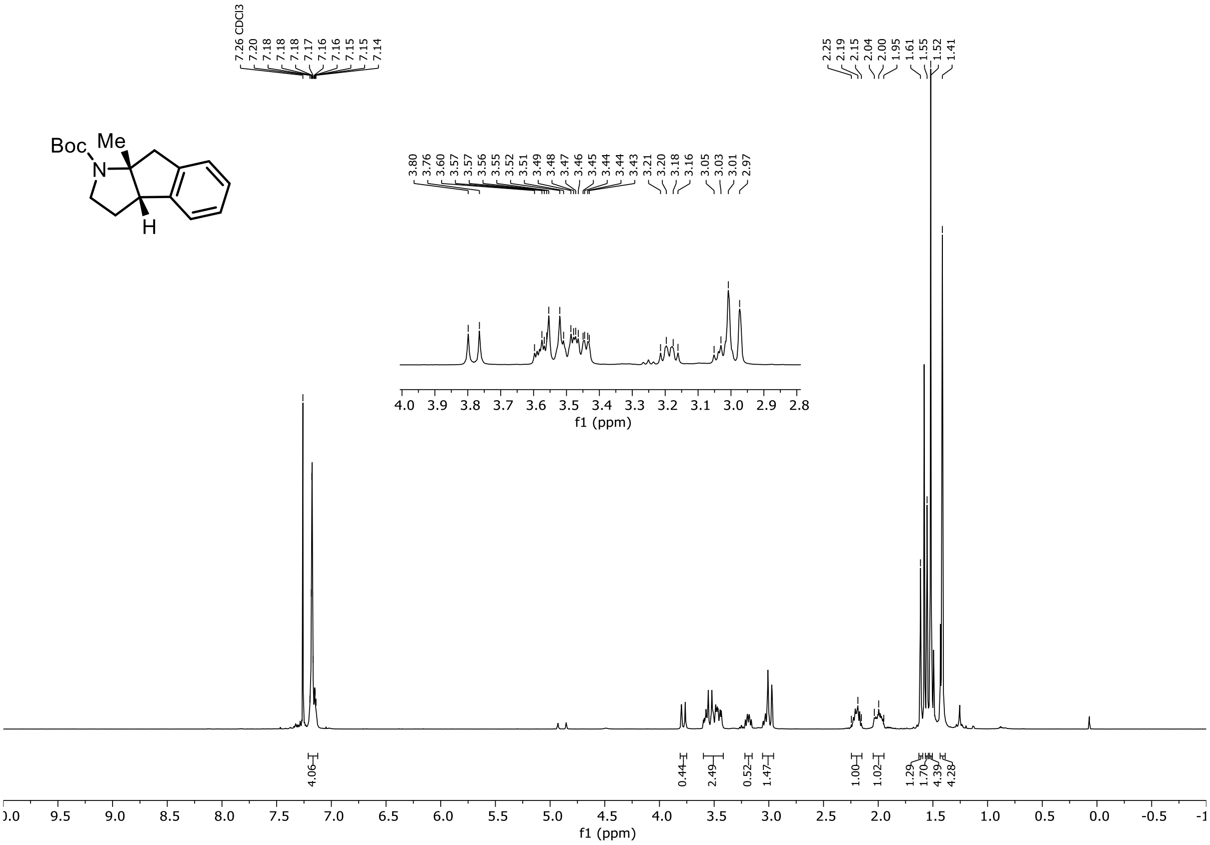

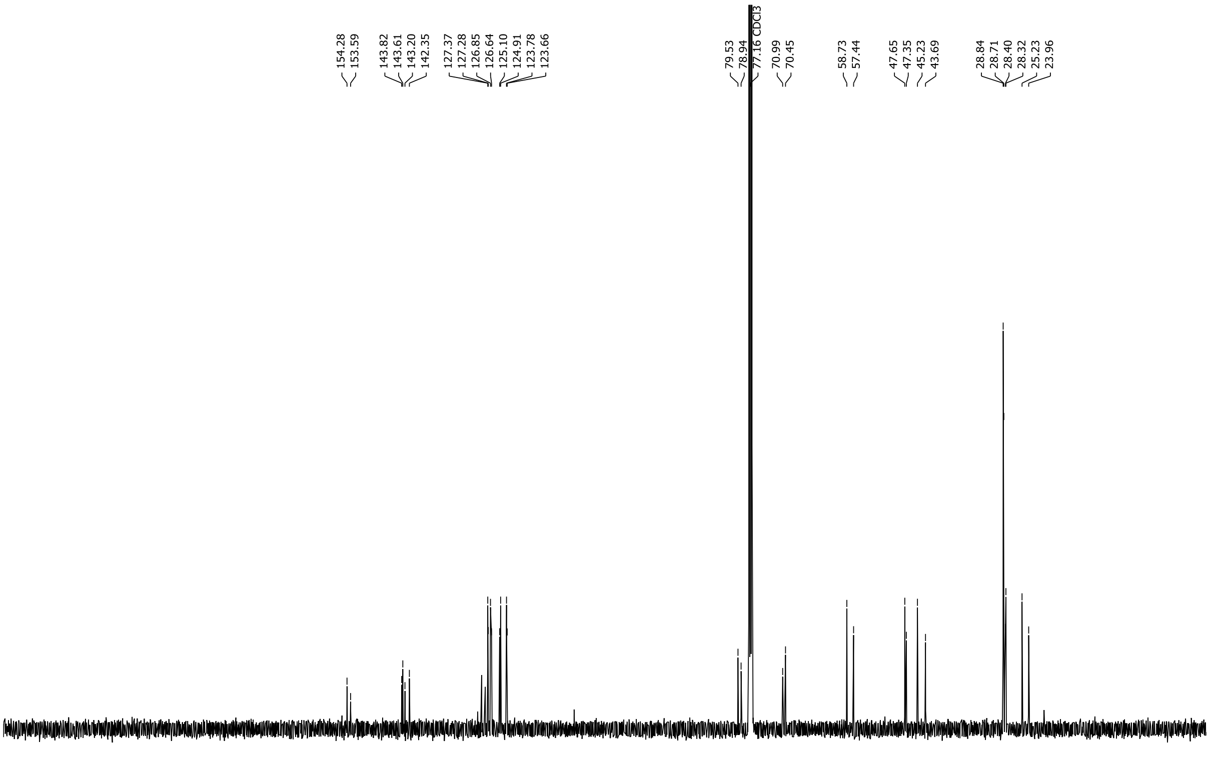

Benzyl (4-methyl-3-phenylpent-4-en-1-yl)((perfluorobenzoyl)oxy)carbamate (1c)

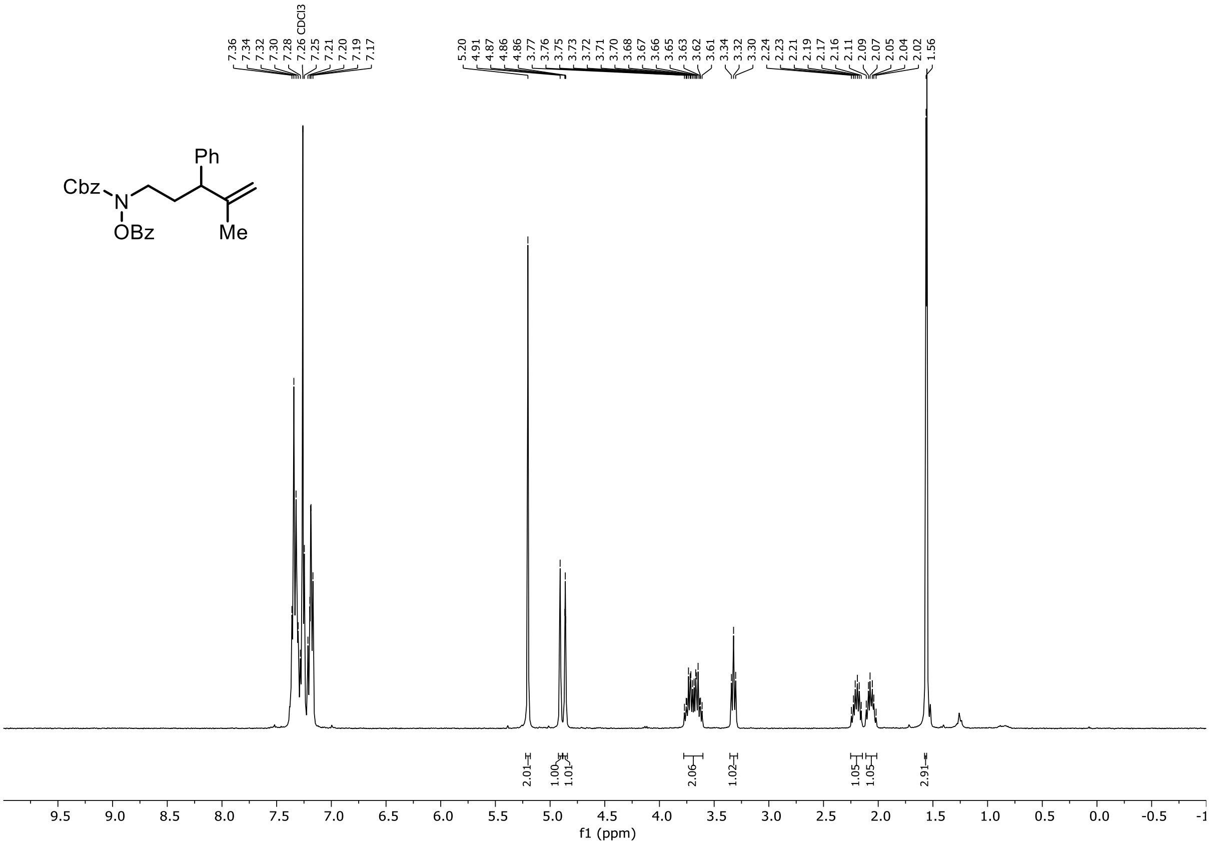

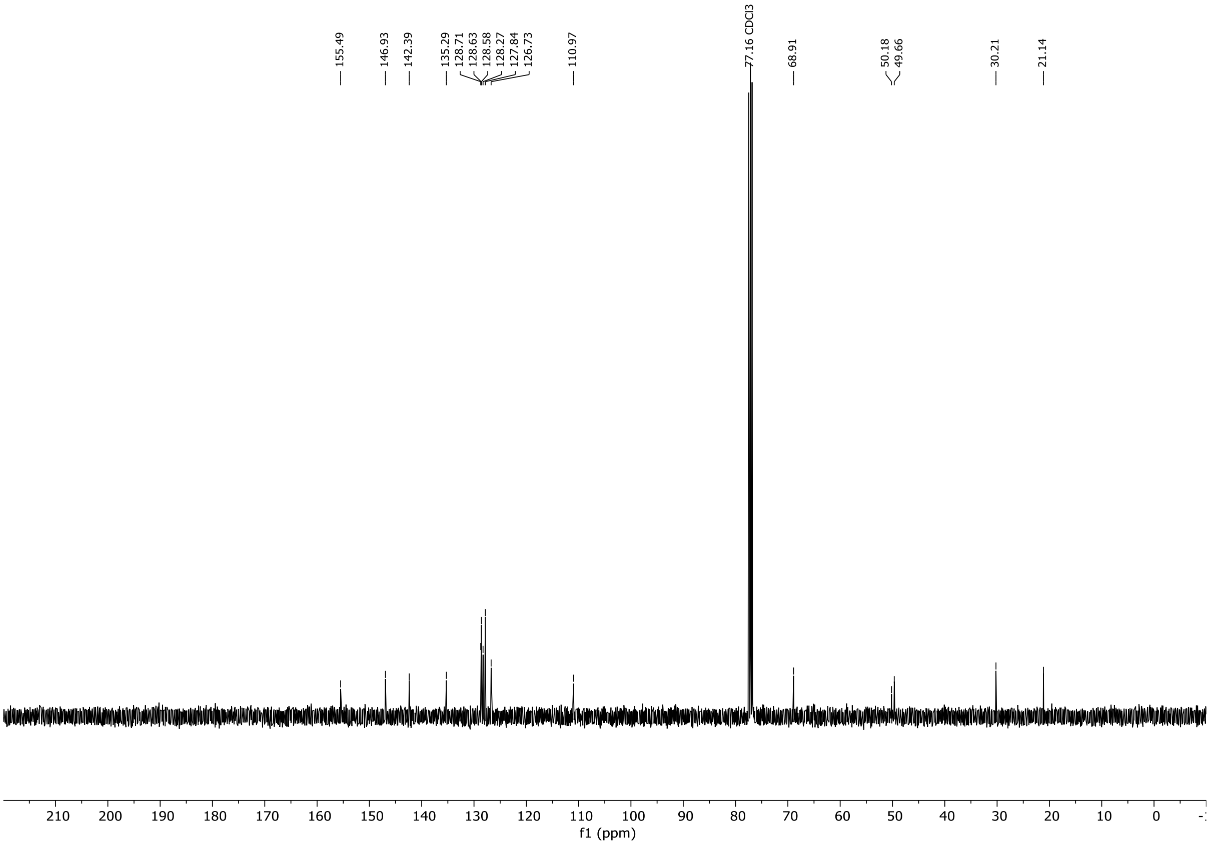

Benzyl 8a-methyl-3,3a,8,8a-tetrahydroindeno[2,1-*b*]pyrrole-1(2*H*)-carboxylate (2c)

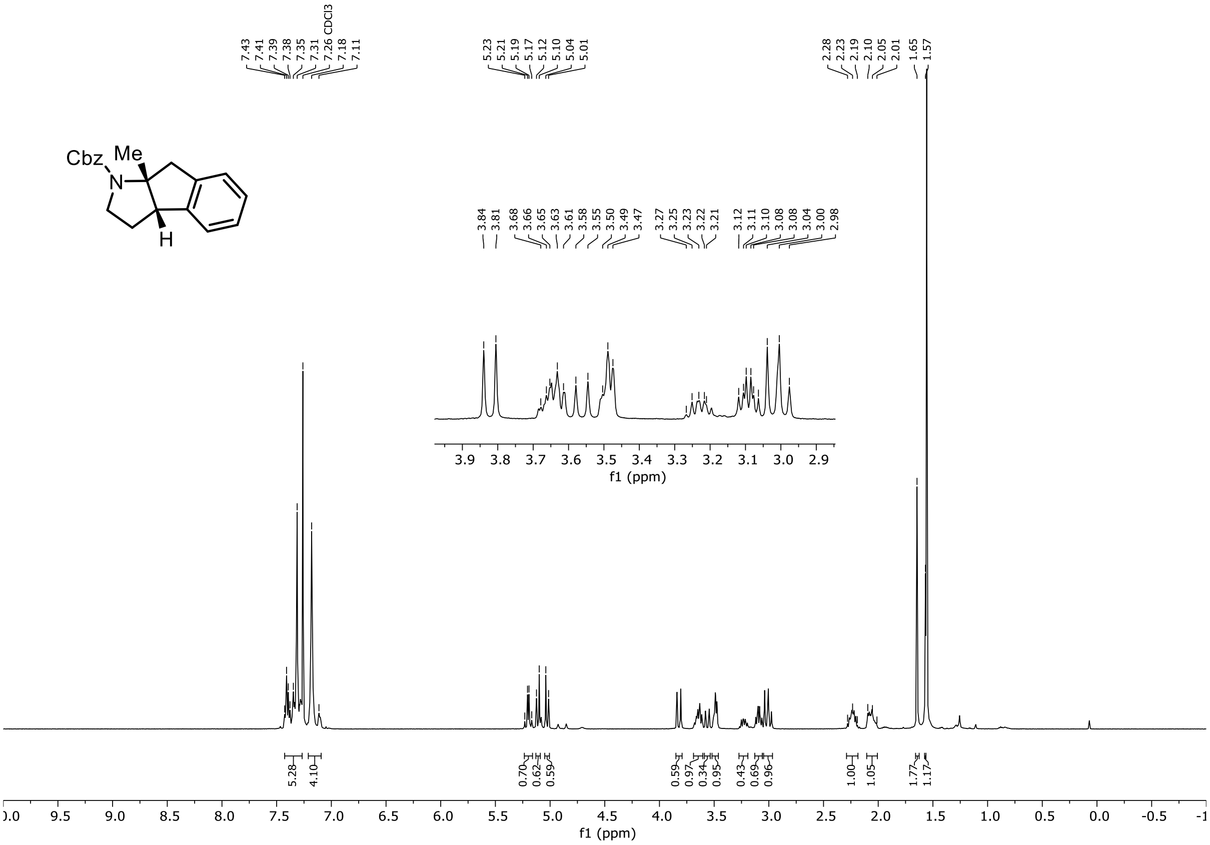

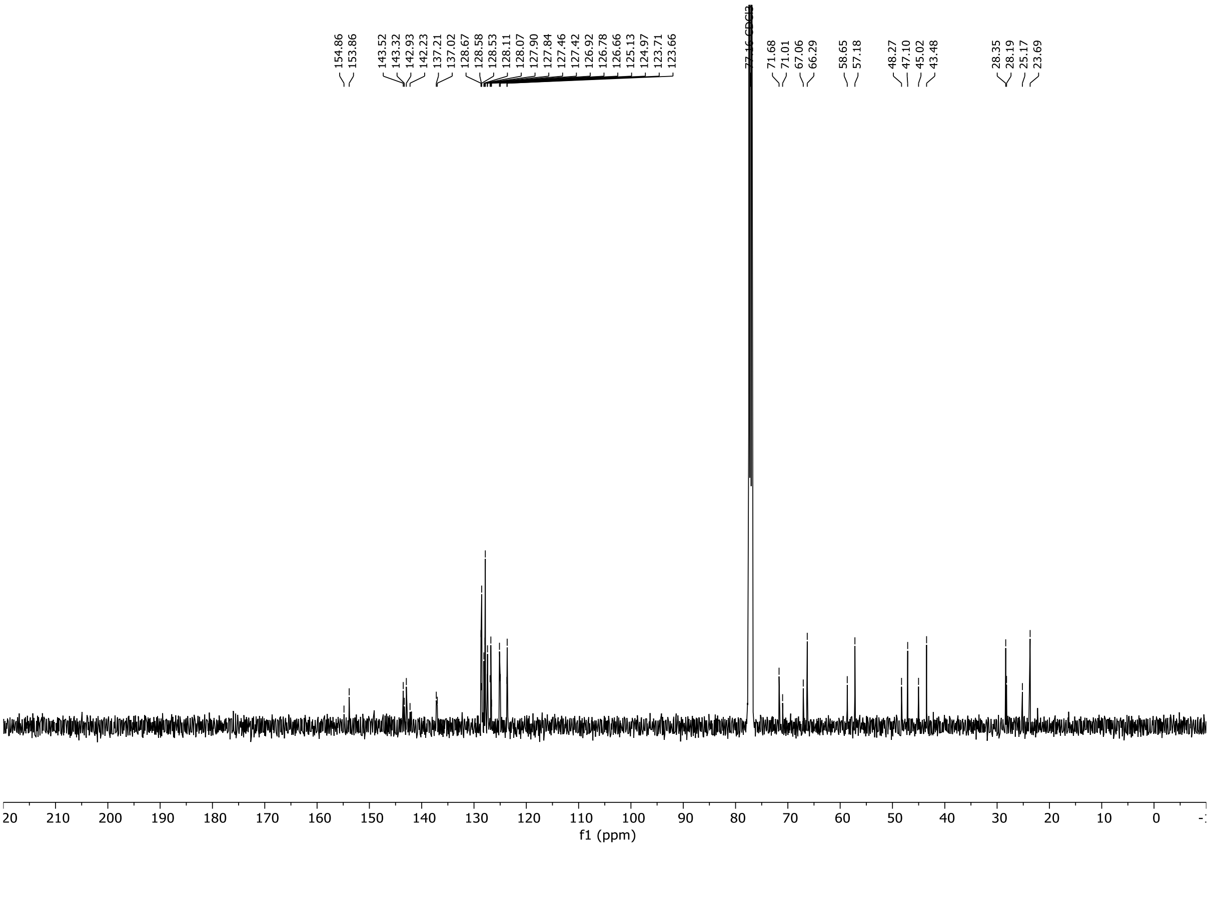

3-(4-Methoxyphenyl)-4-methylpent-4-enoic acid

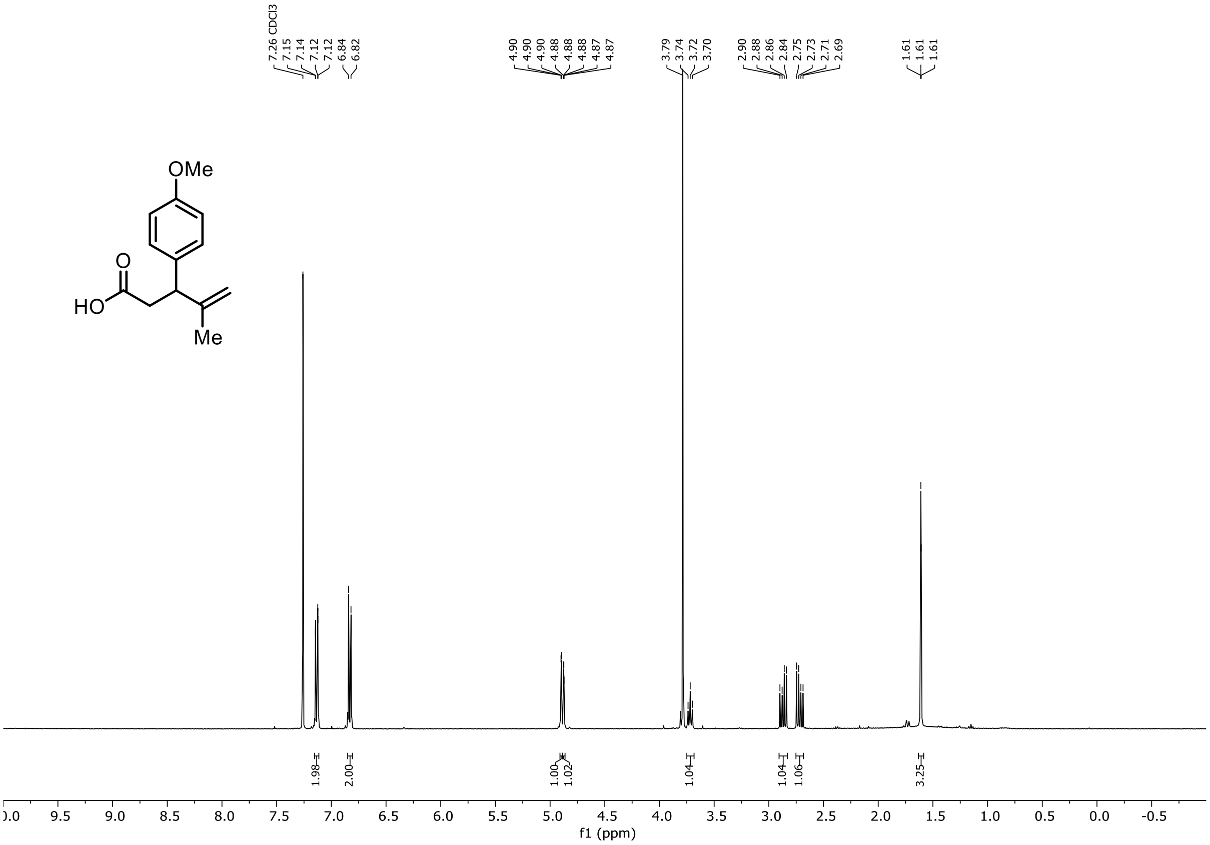

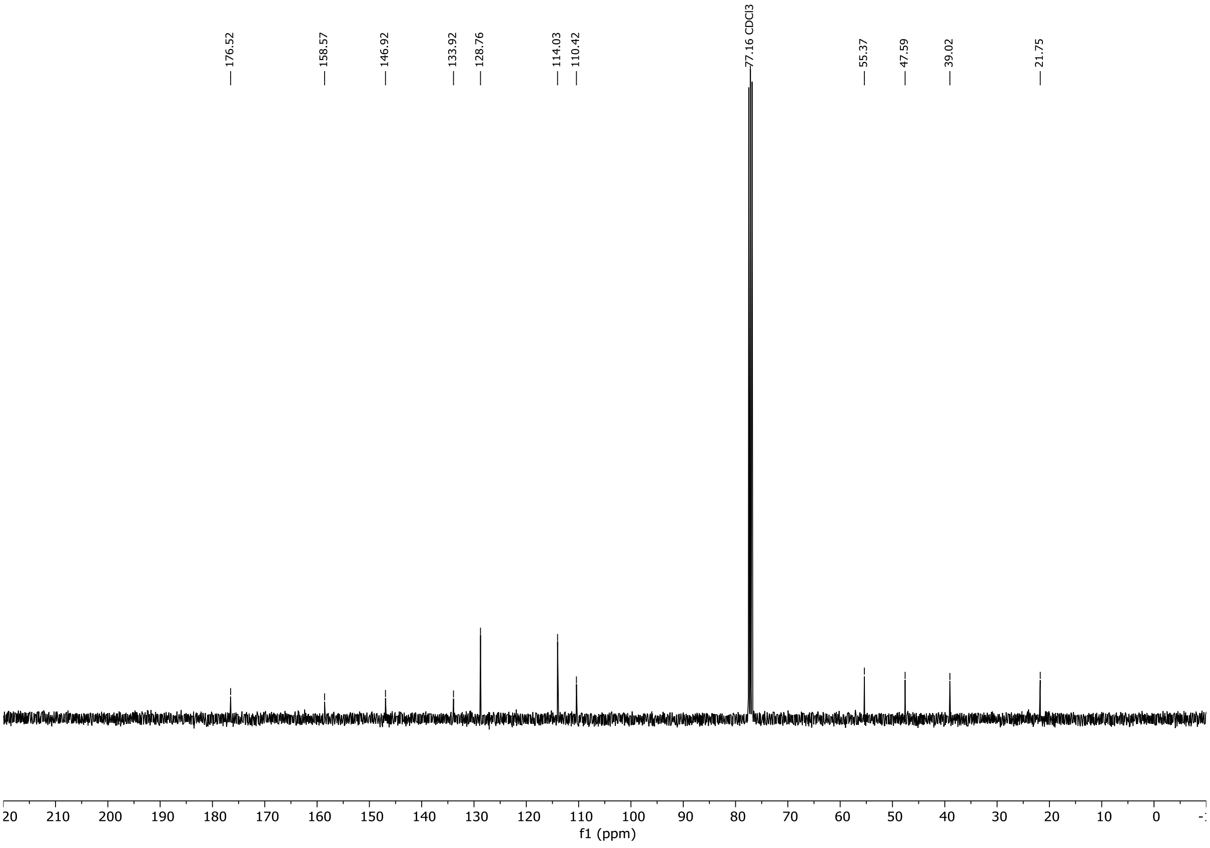

3-(4-Methoxyphenyl)-4-methylpent-4-en-1-ol

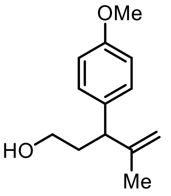

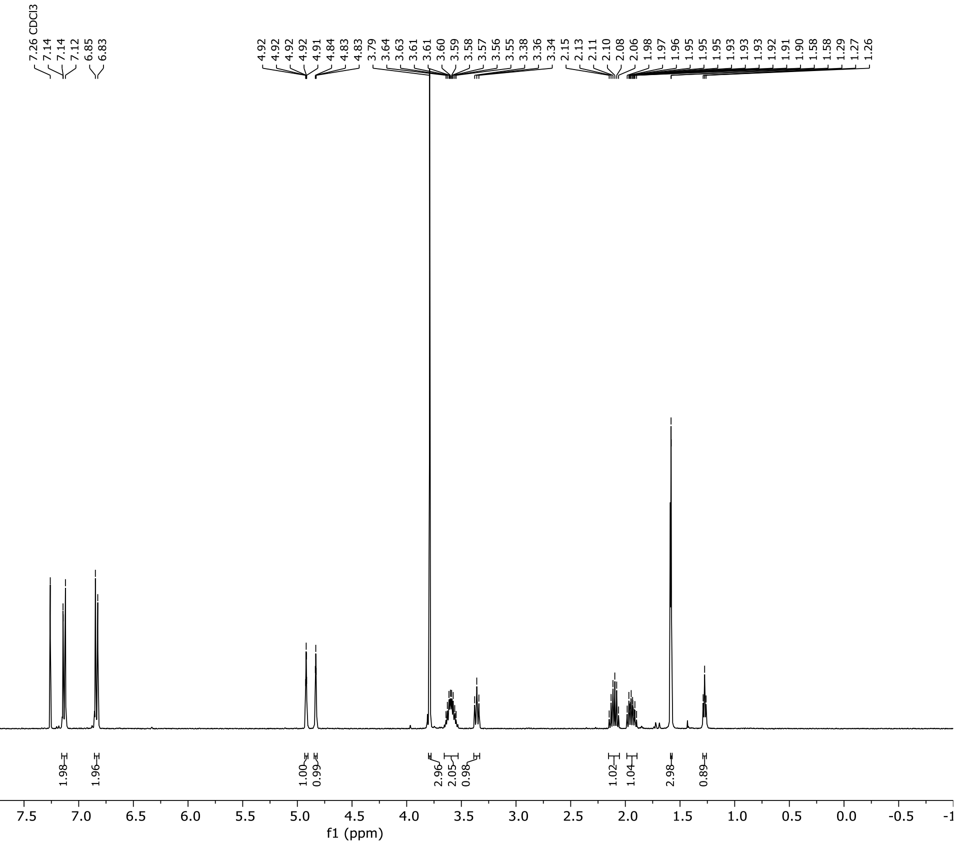

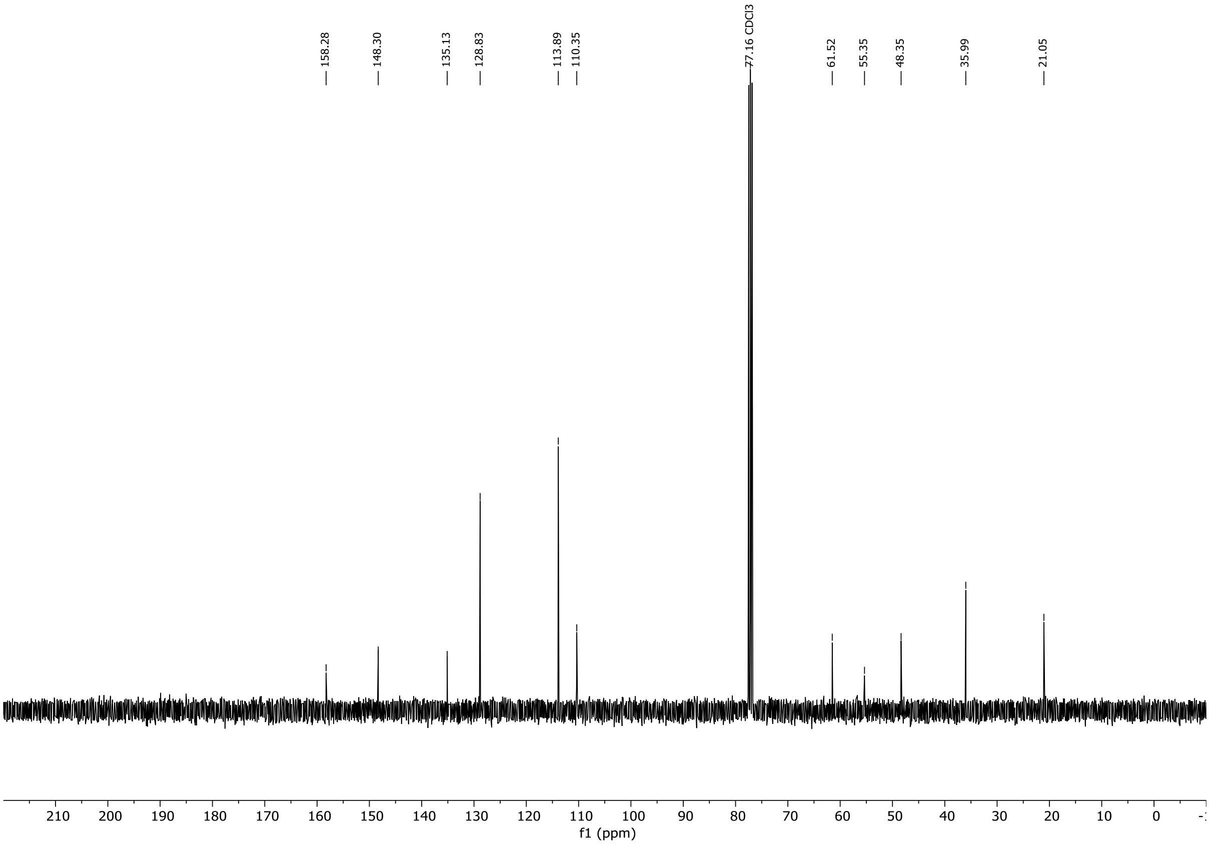

Benzyl(3-(4-methoxyphenyl)-4-methylpent-4-en-1-yl)((perfluorobenzoyl)oxy)carbamate (1d)

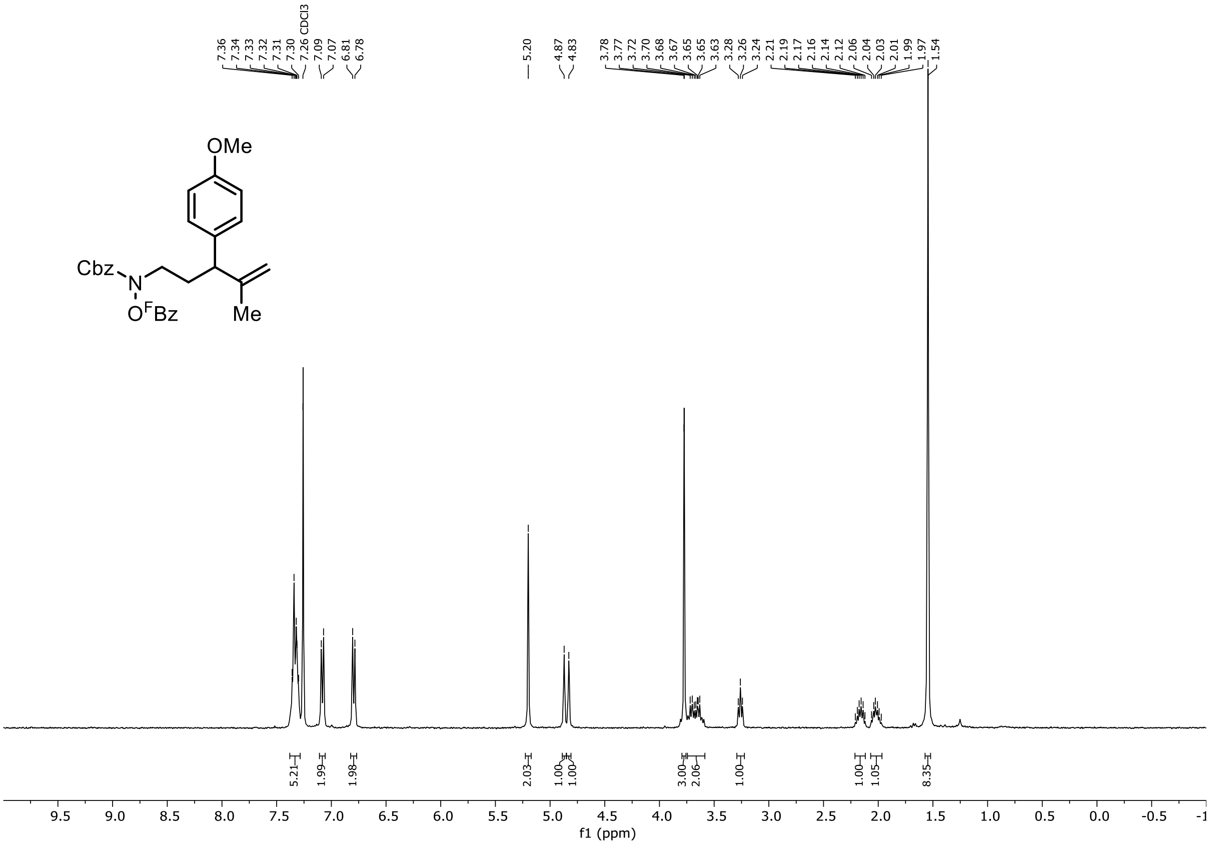

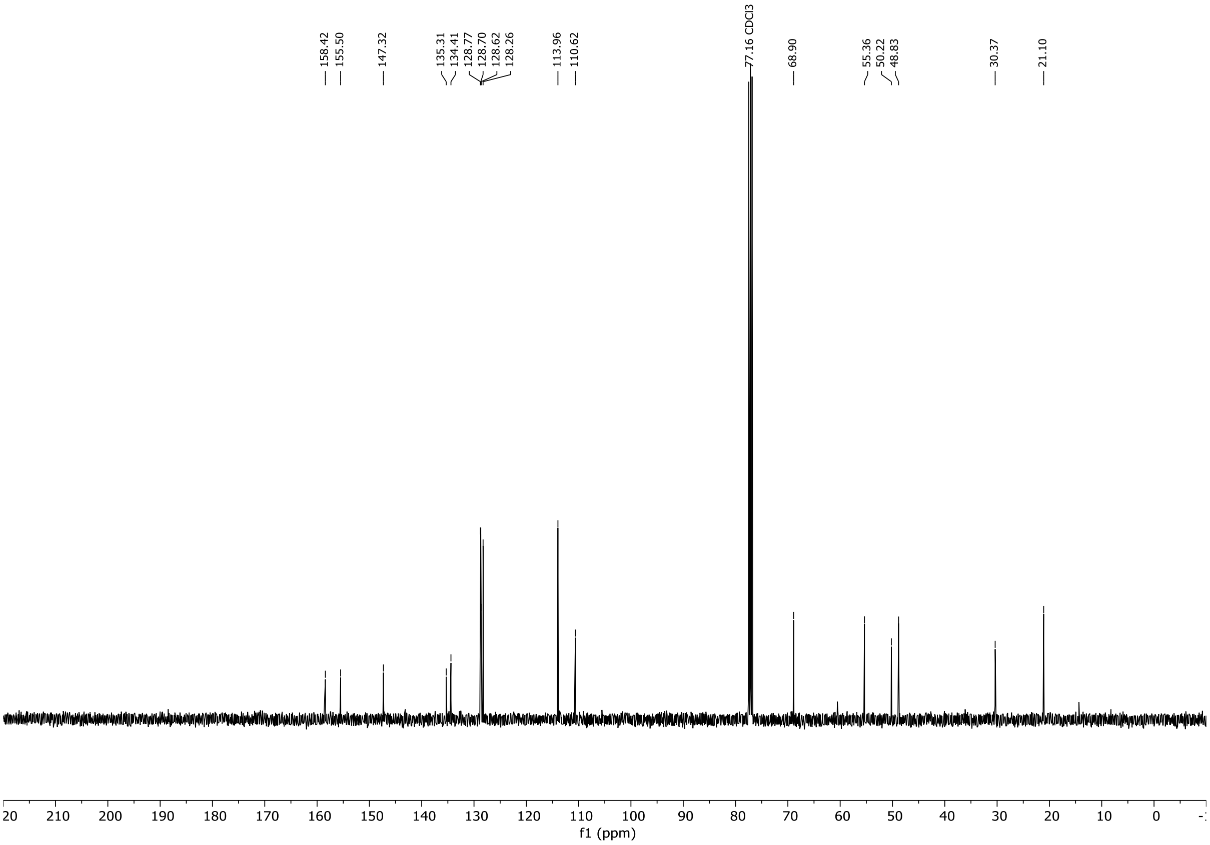

Benzyl 6-methoxy-8a-methyl-3,3a,8,8a-tetrahydroindeno[2,1-*b*]pyrrole-1(2*H*)-carboxylate (2d)

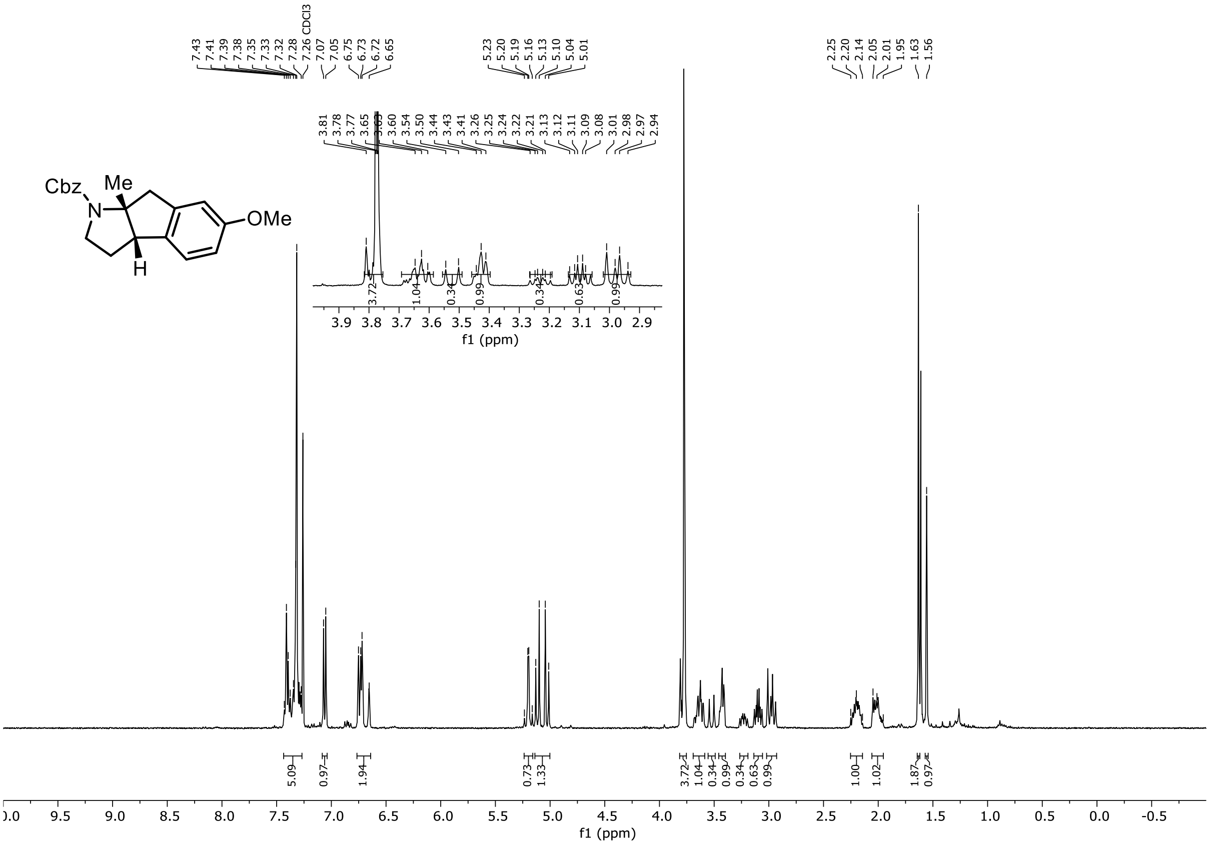

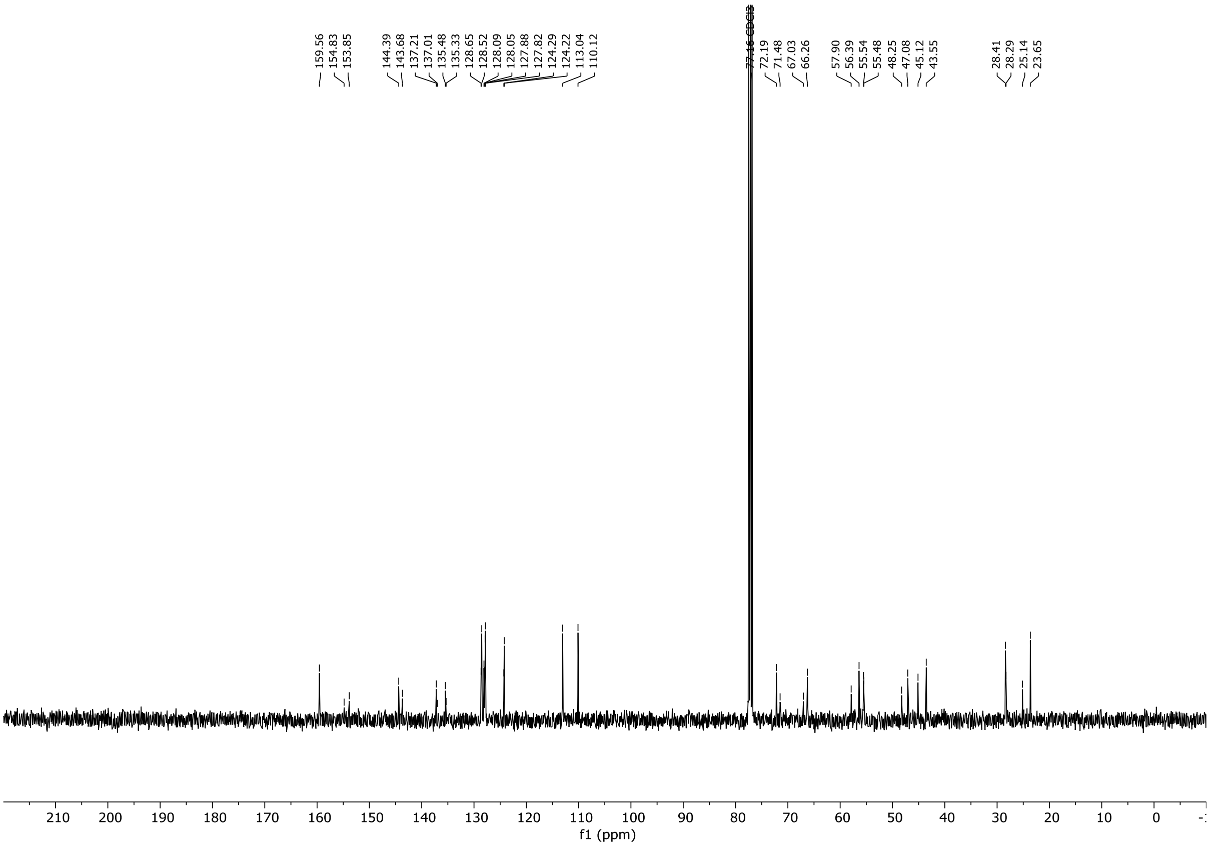

3-(4-(*tert*-Butyl)phenyl)-4-methylpent-4-enoic acid

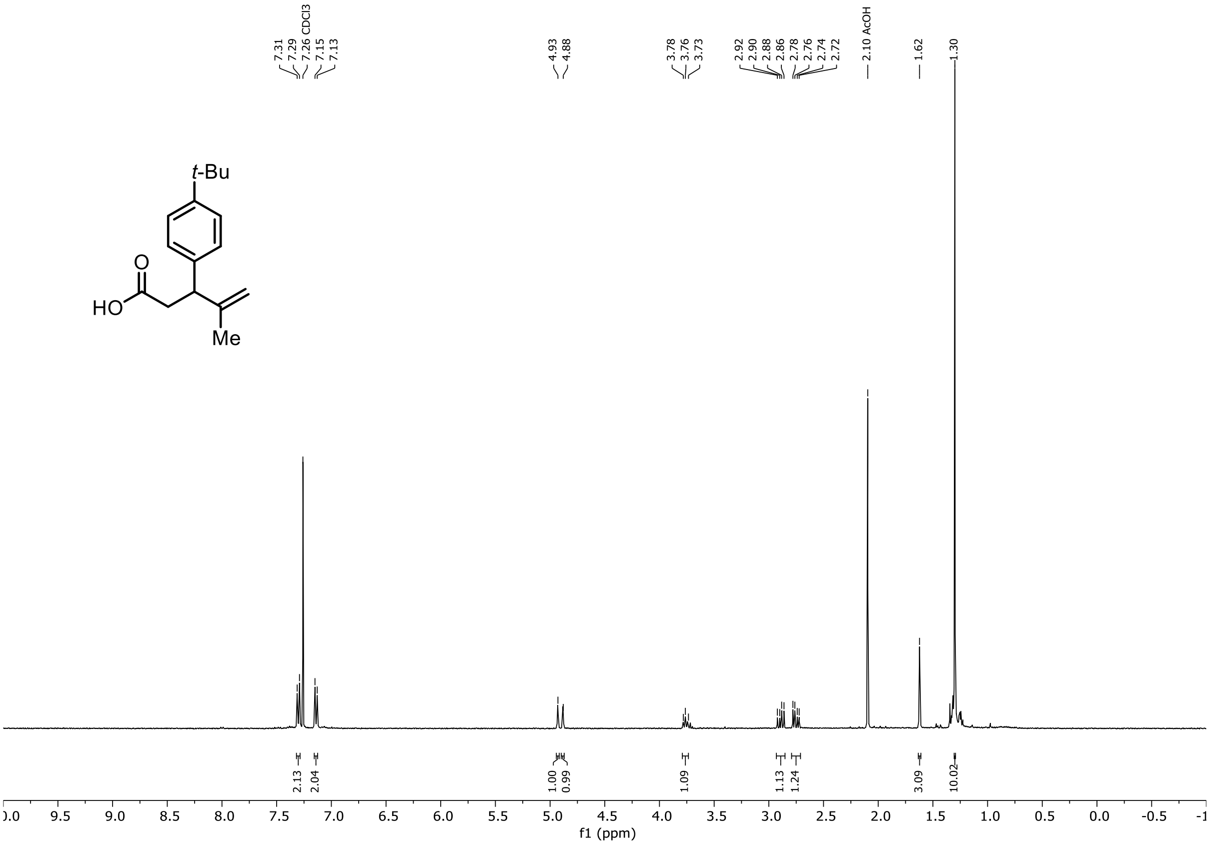

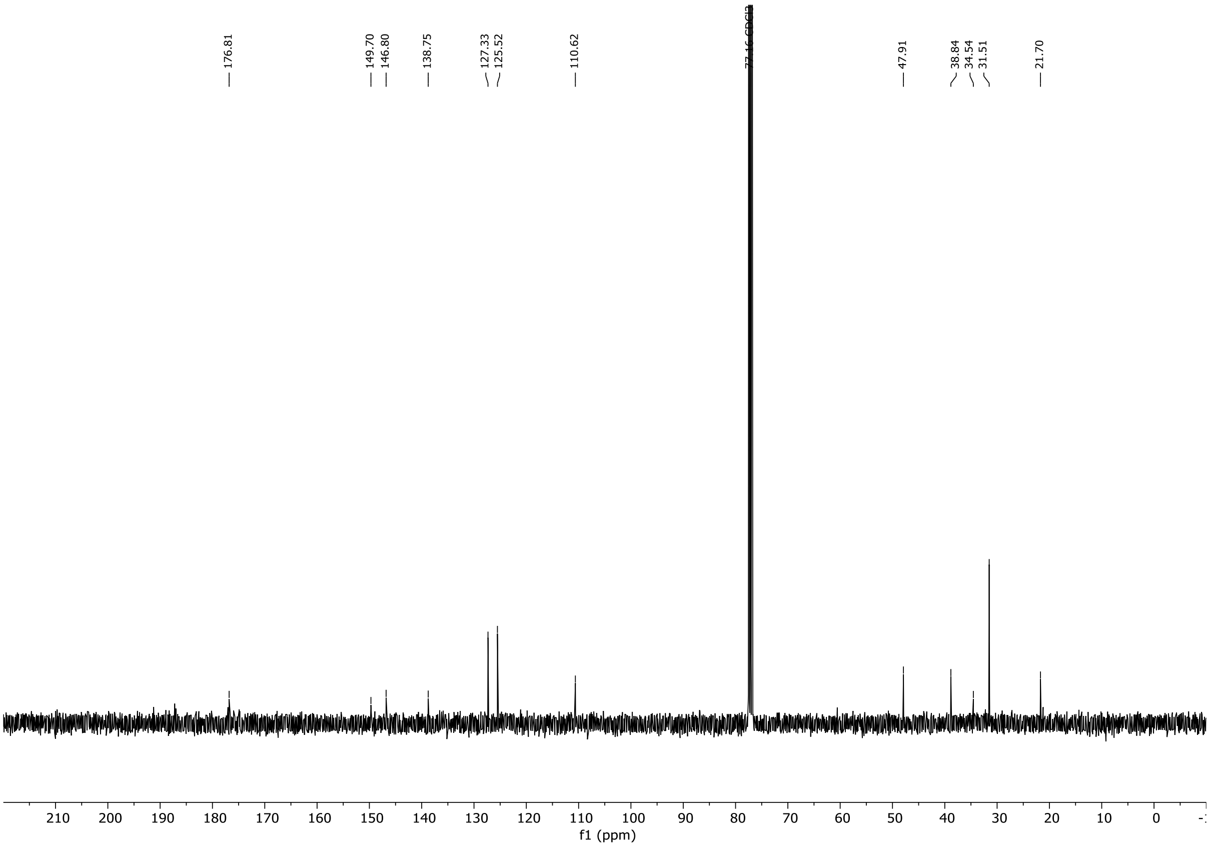

3-(4-(*tert*-Butyl)phenyl)-4-methylpent-4-en-1-ol

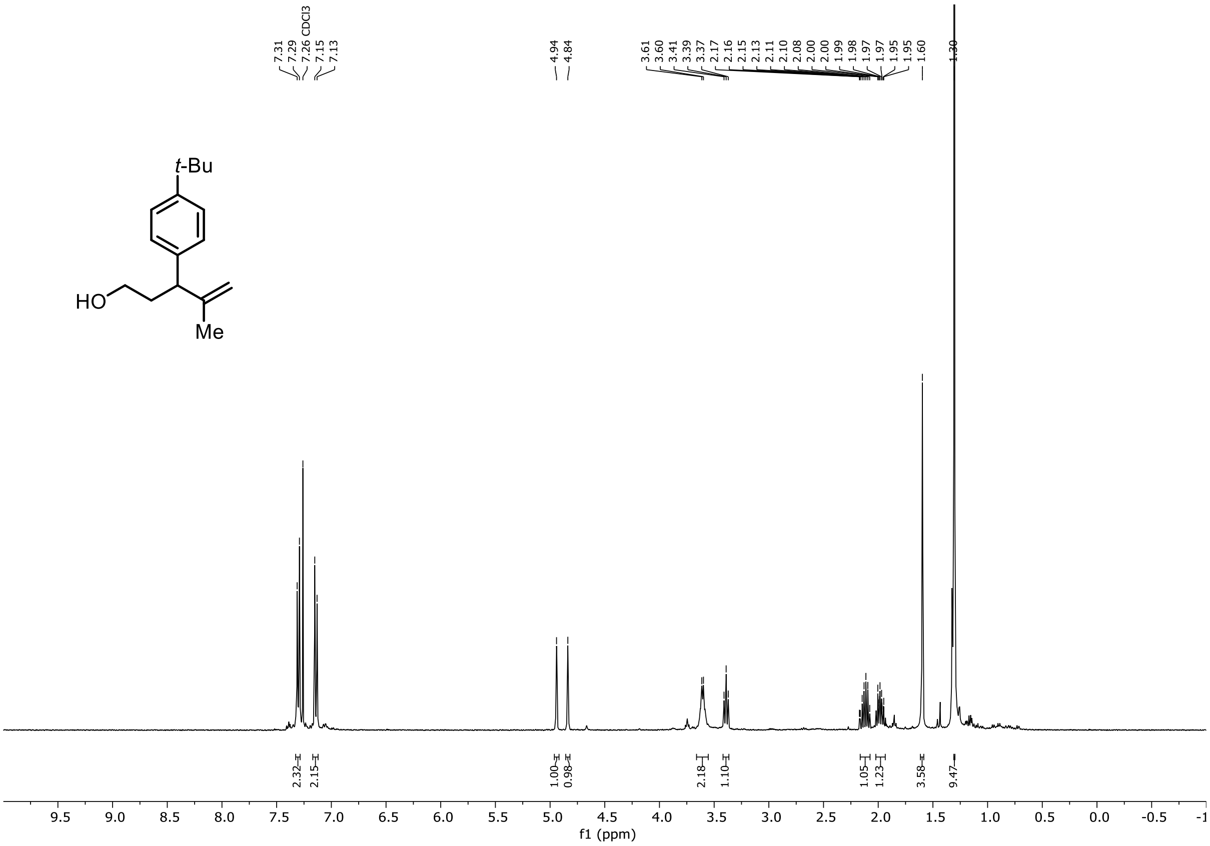

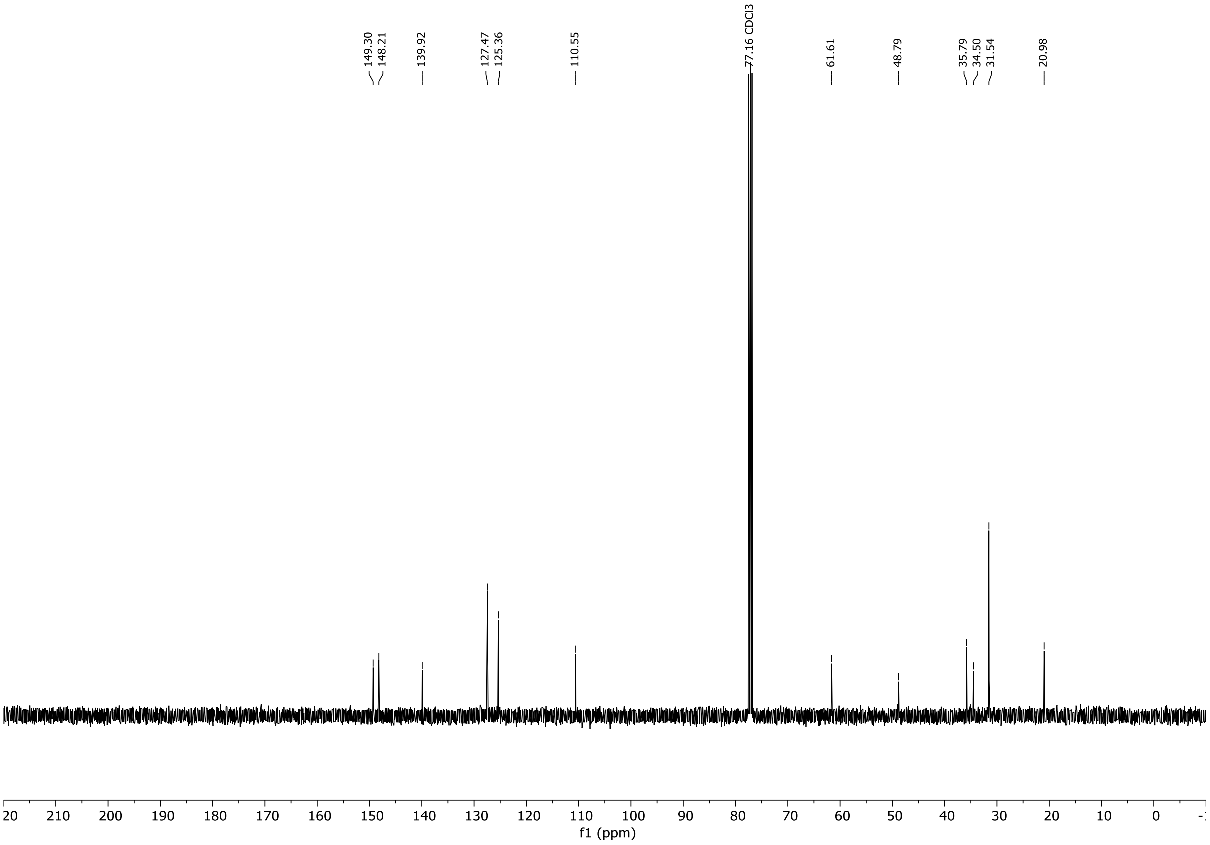

Benzyl (3-(4-(tert-butyl)phenyl)-4-methylpent-4-en-1-yl)((perfluorobenzoyl)oxy)carbamate (1e)

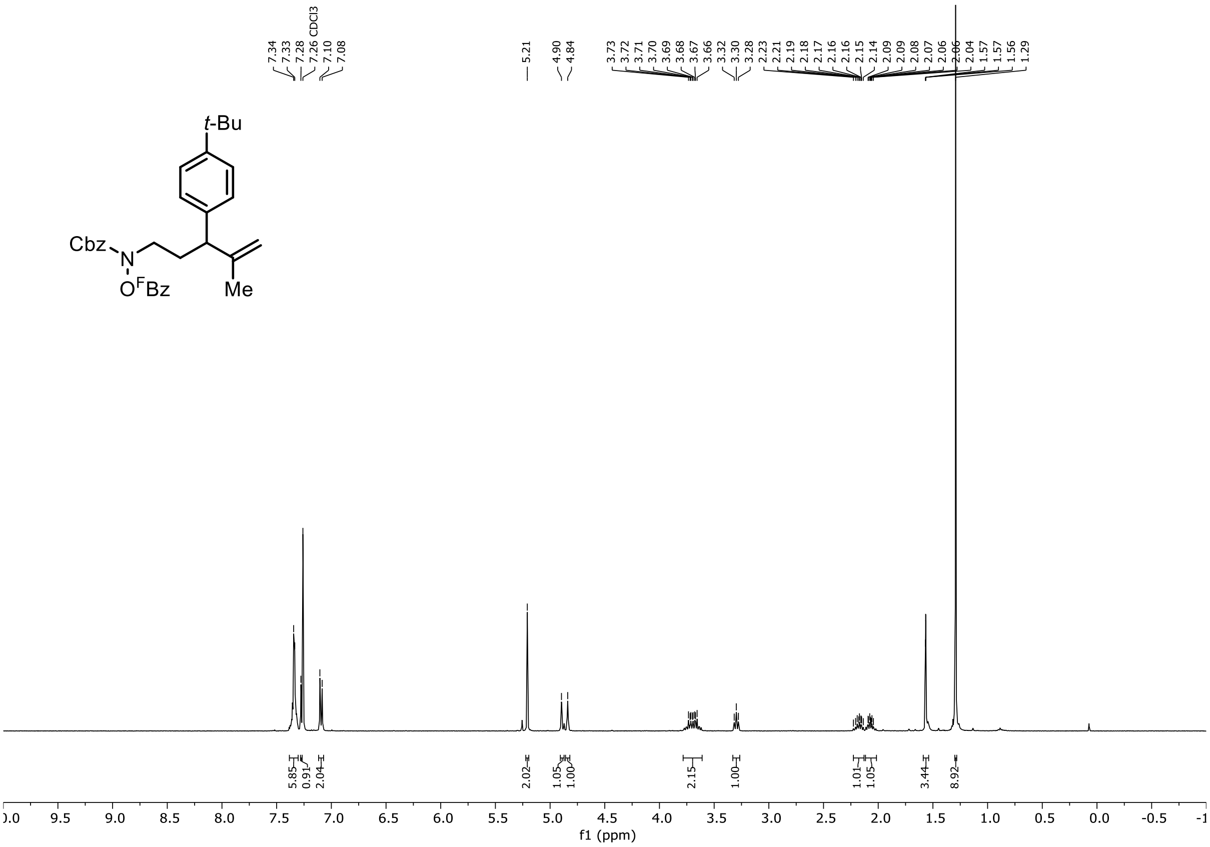

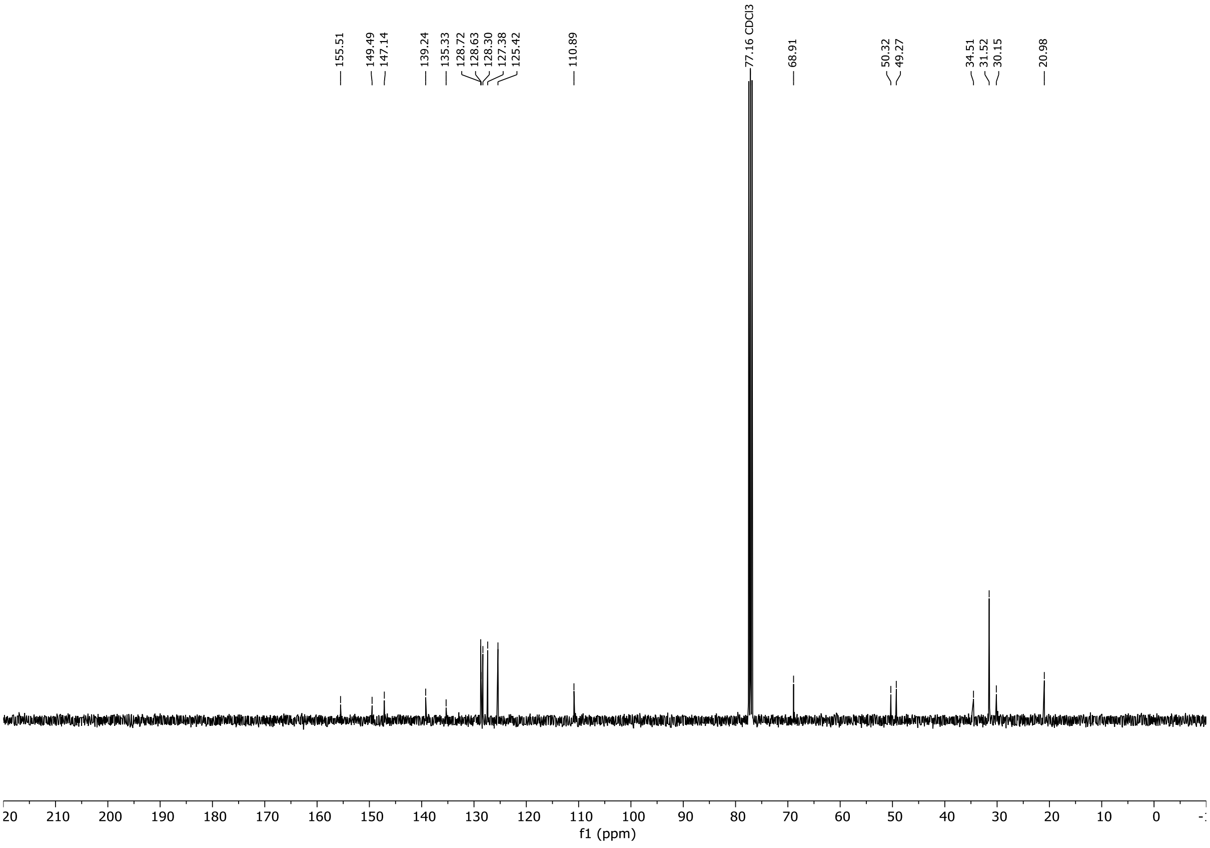

Benzyl 6-(tert-butyl)-8a-methyl-3,3a,8,8a-tetrahydroindeno[2,1-*b*]pyrrole-1(2*H*)-carboxylate (2e)

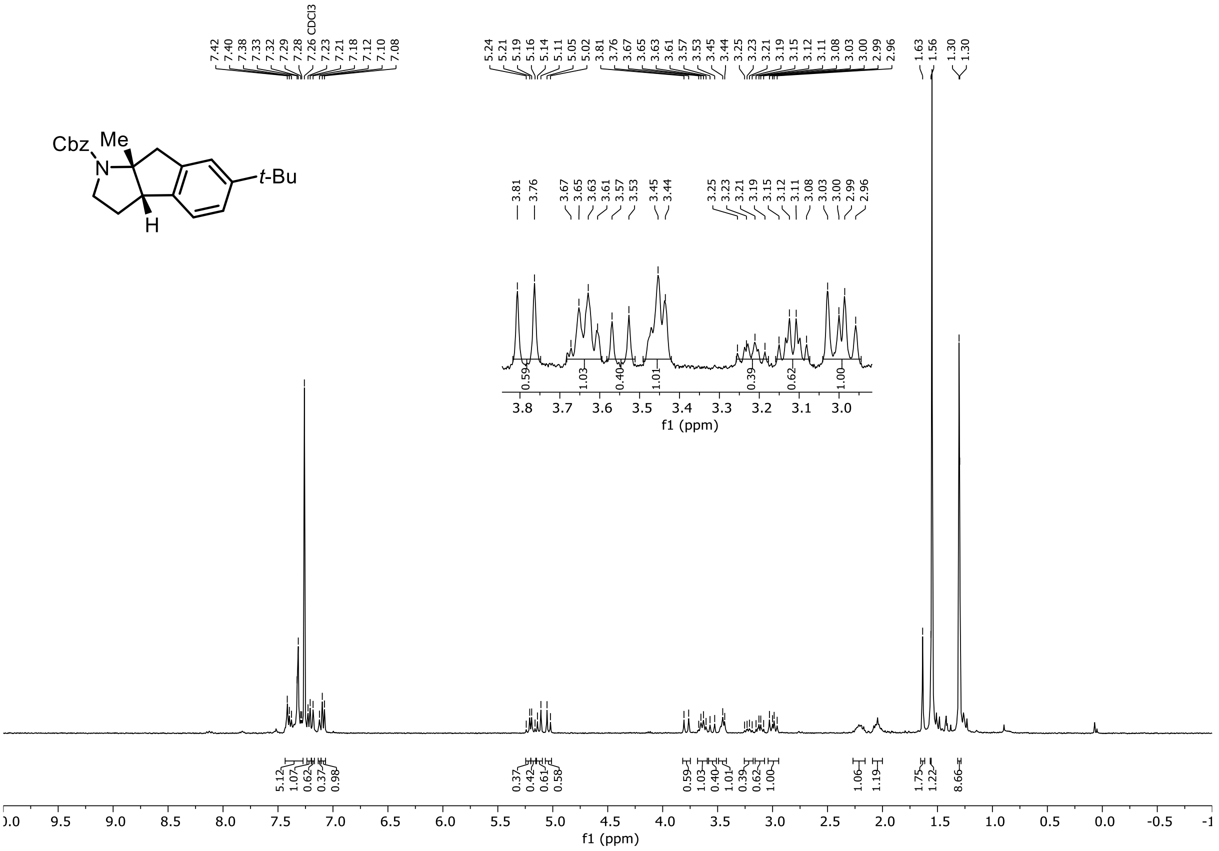

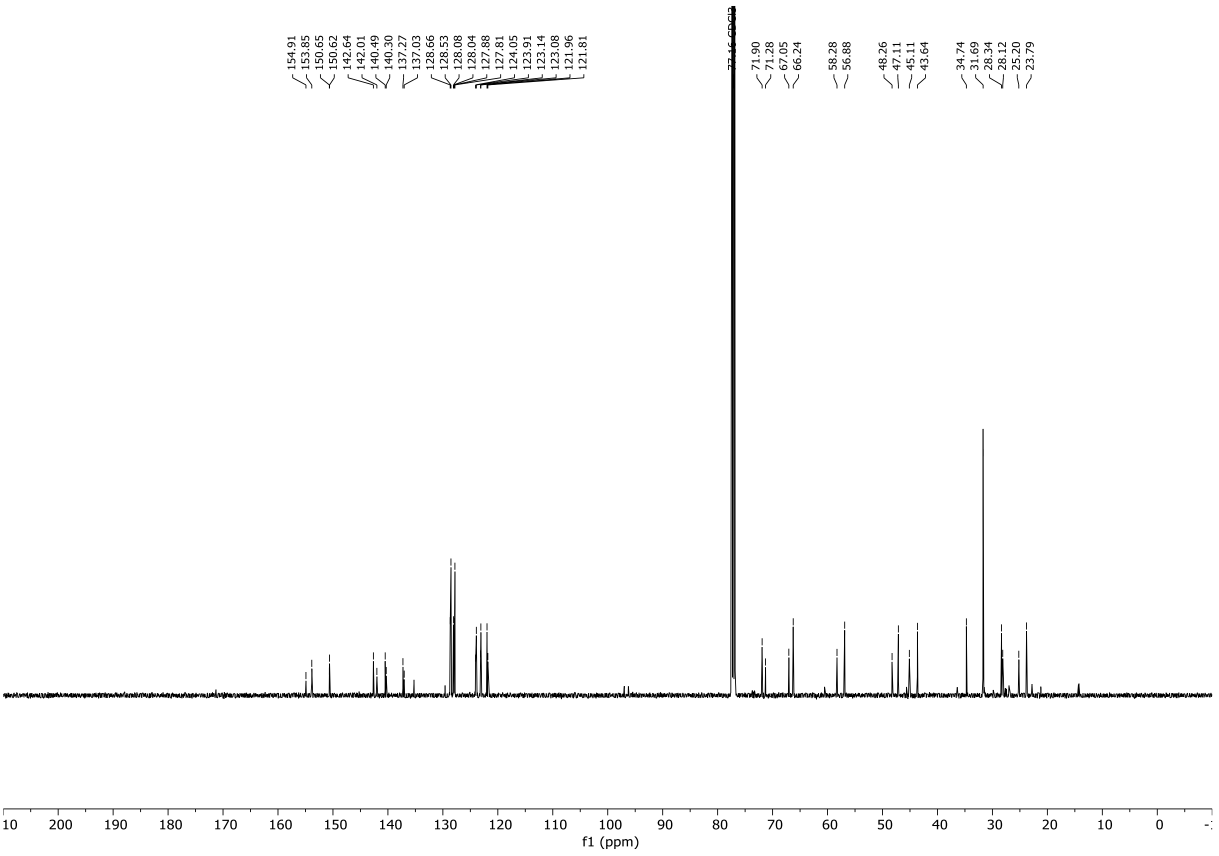

4-Methyl-3-(4-(trifluoromethyl)phenyl)pent-4-enoic acid

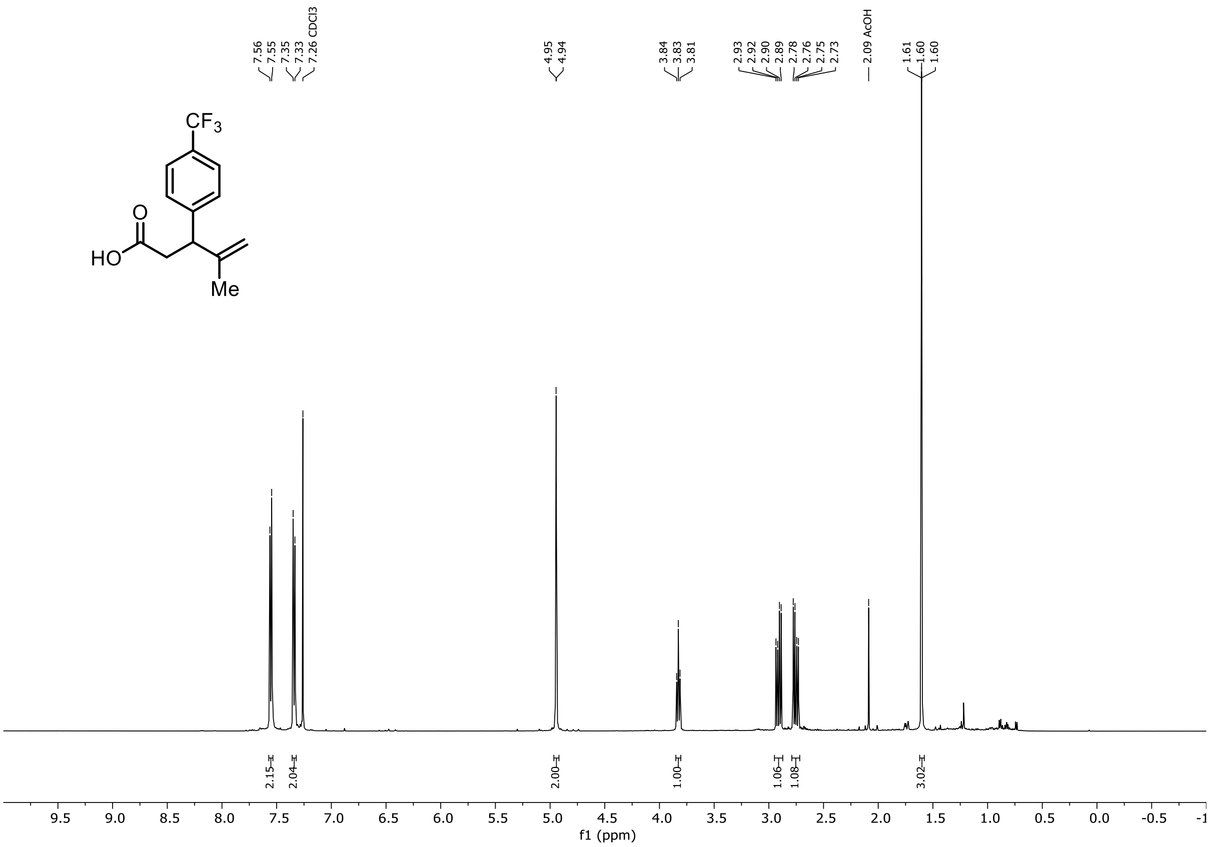

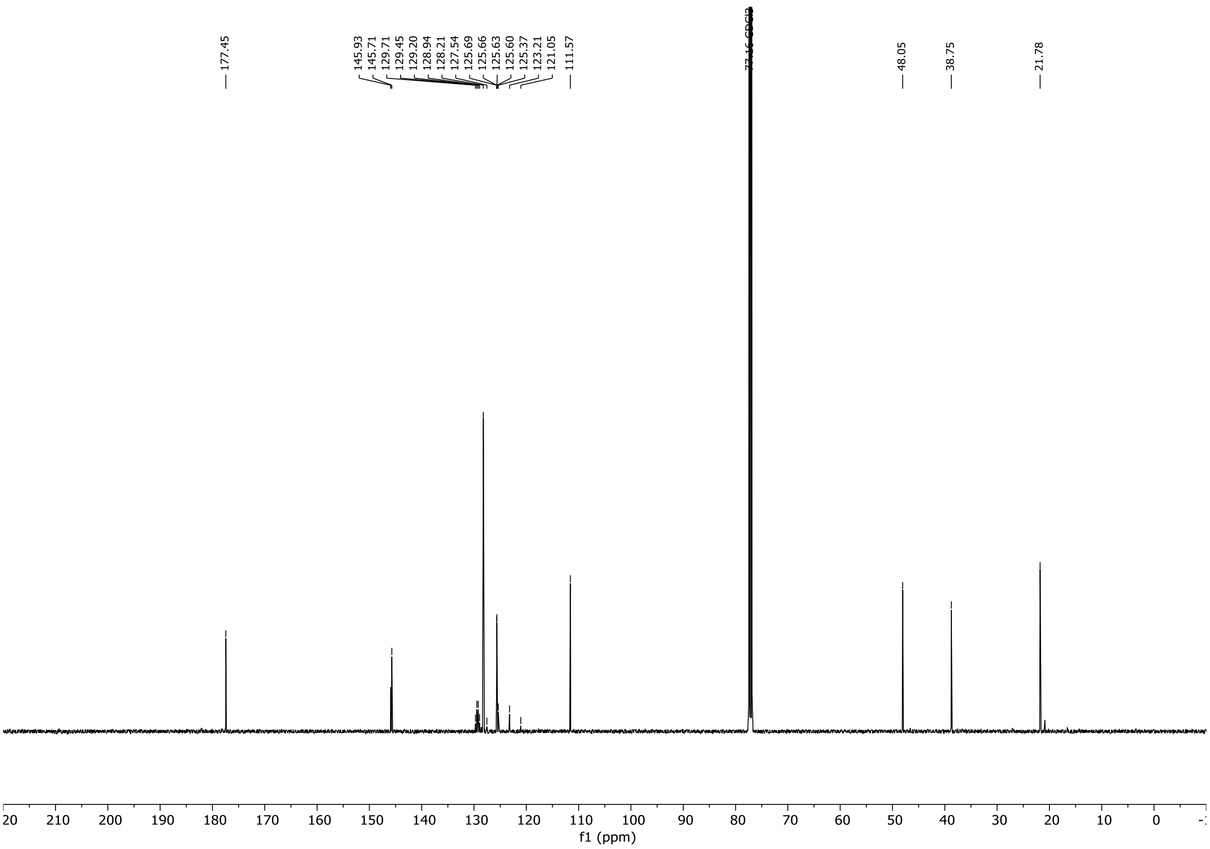

4-Methyl-3-(4-(trifluoromethyl)phenyl)pent-4-en-1-ol

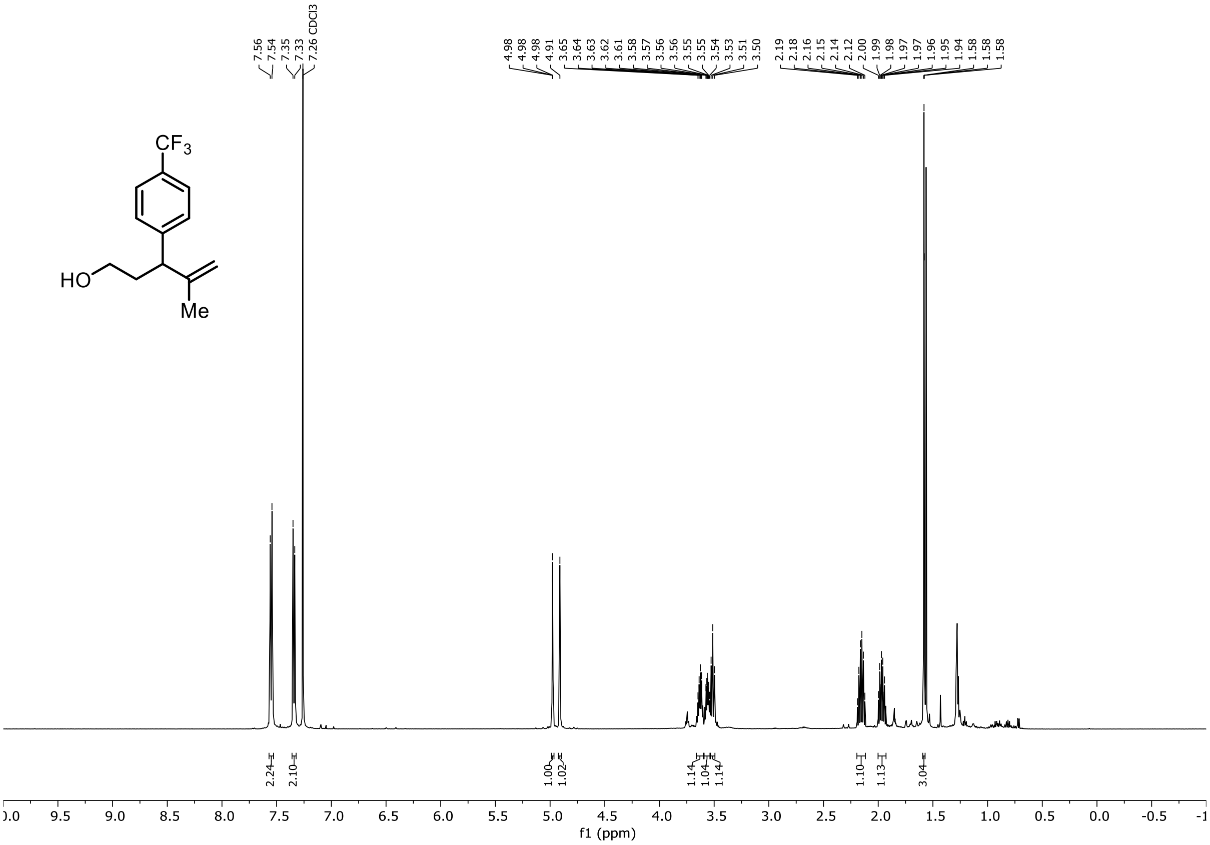

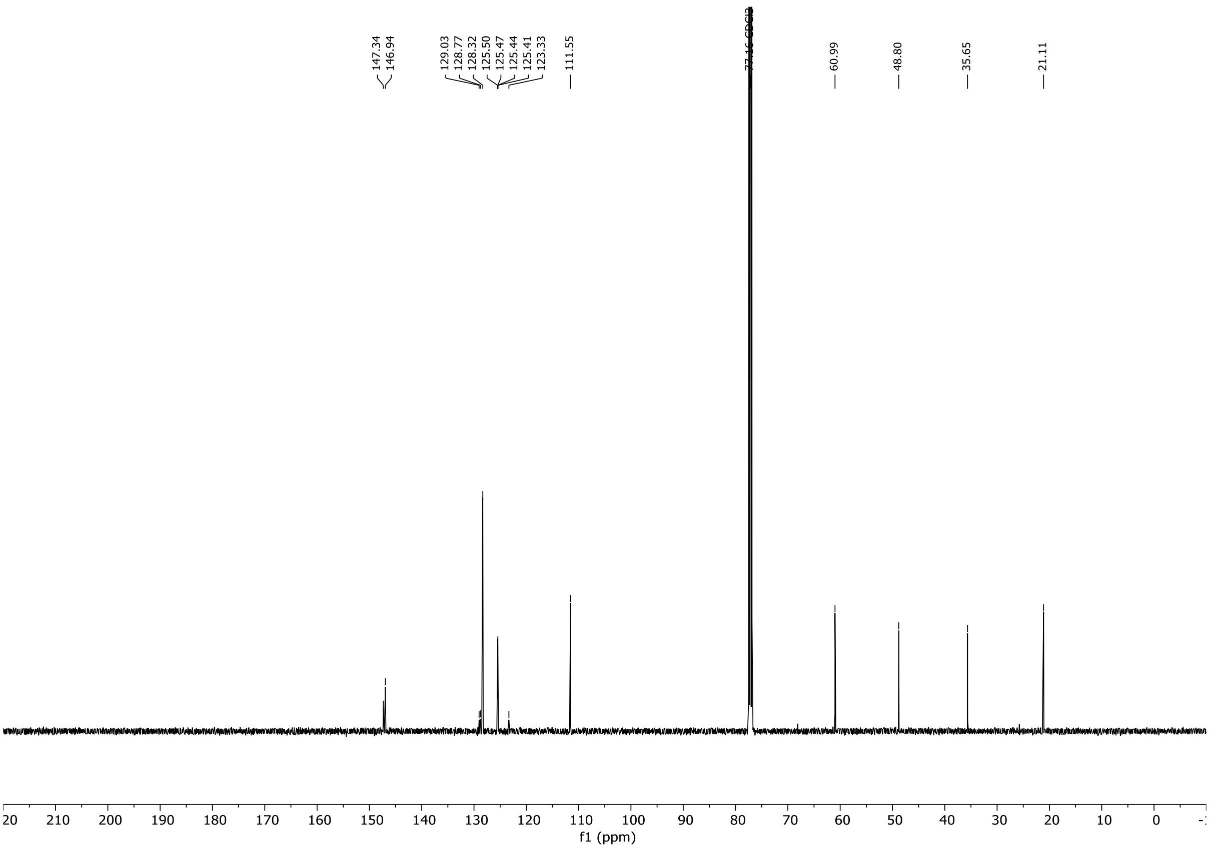

Benzyl (4-methyl-3-(4-(trifluoromethyl)phenyl)pent-4-en-1-yl) ((perfluorobenzoyl)oxy) carbamate (1f)

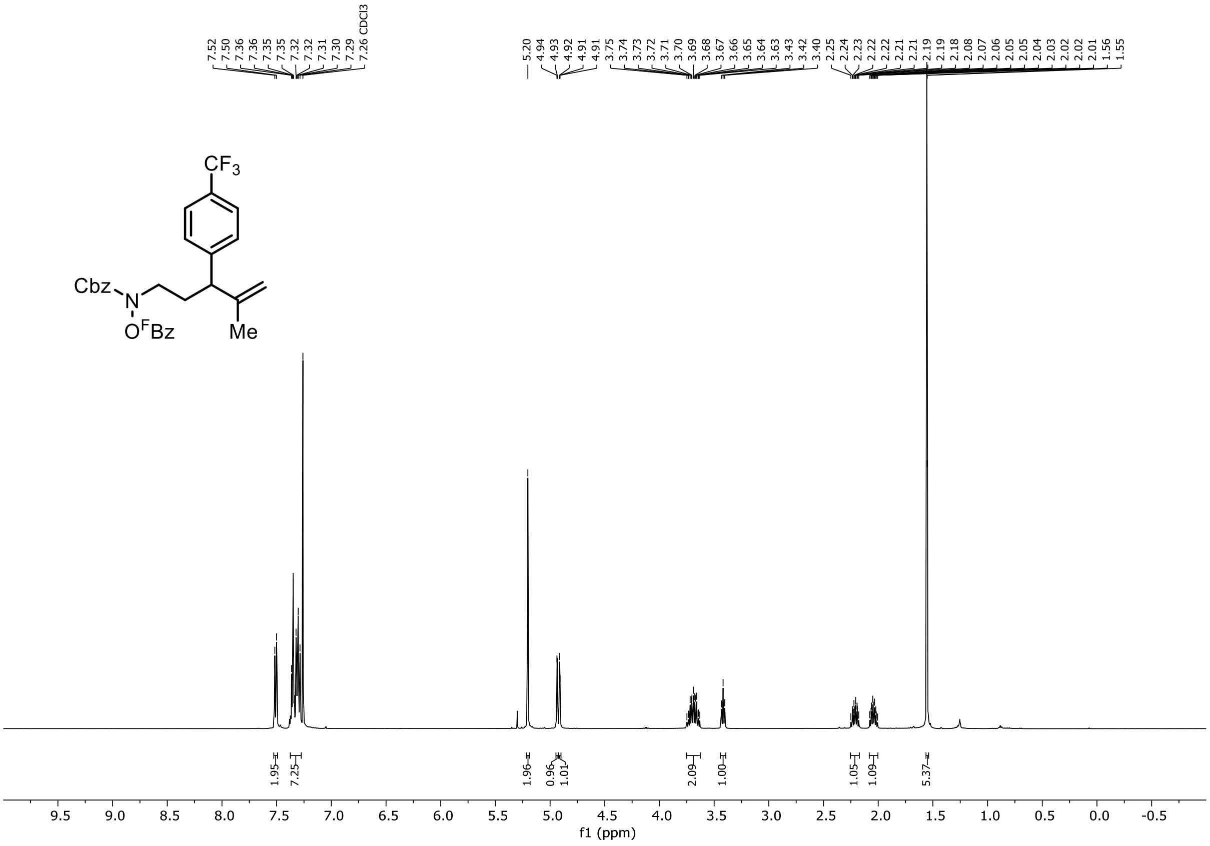

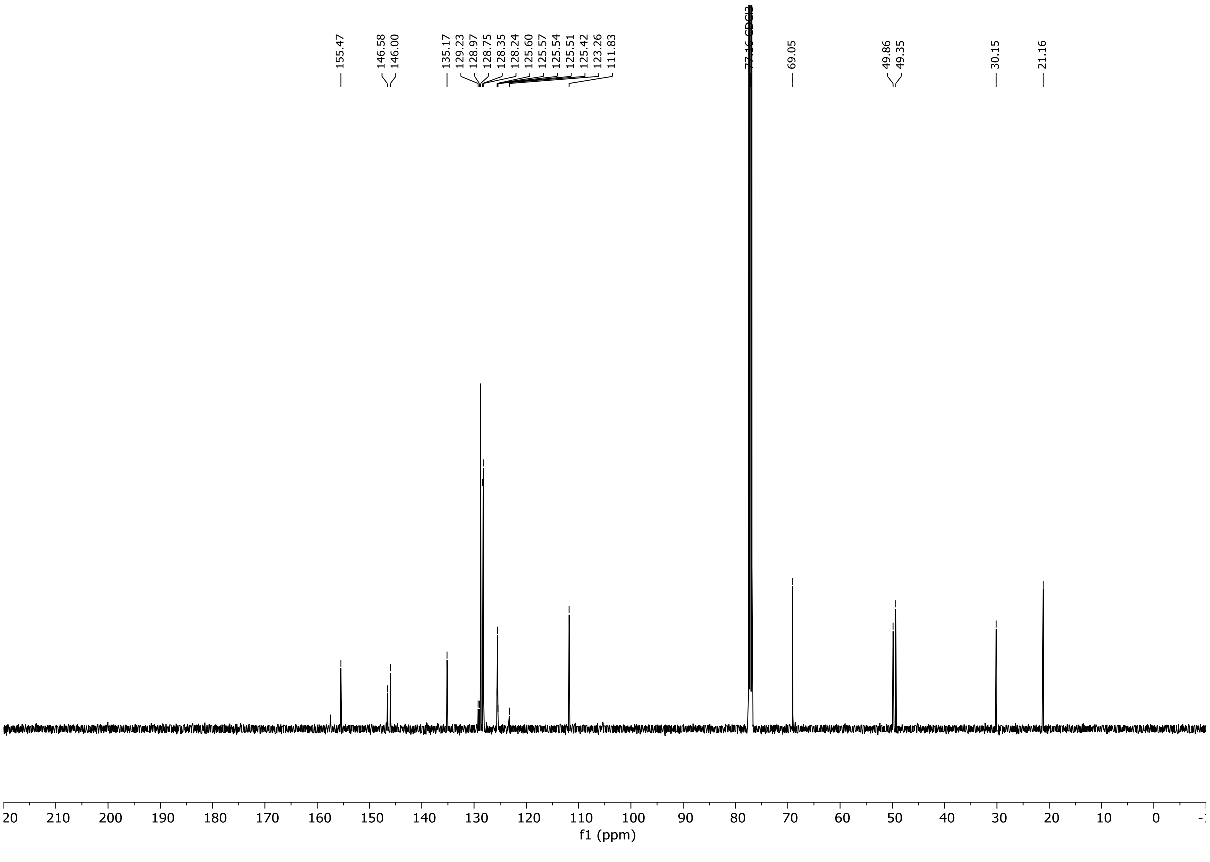

Benzyl 8a-methyl-6-(trifluoromethyl)-3,3a,8,8a-tetrahydroindeno[2,1-*b*]pyrrole-1(2*H*)-carboxylate (2f)

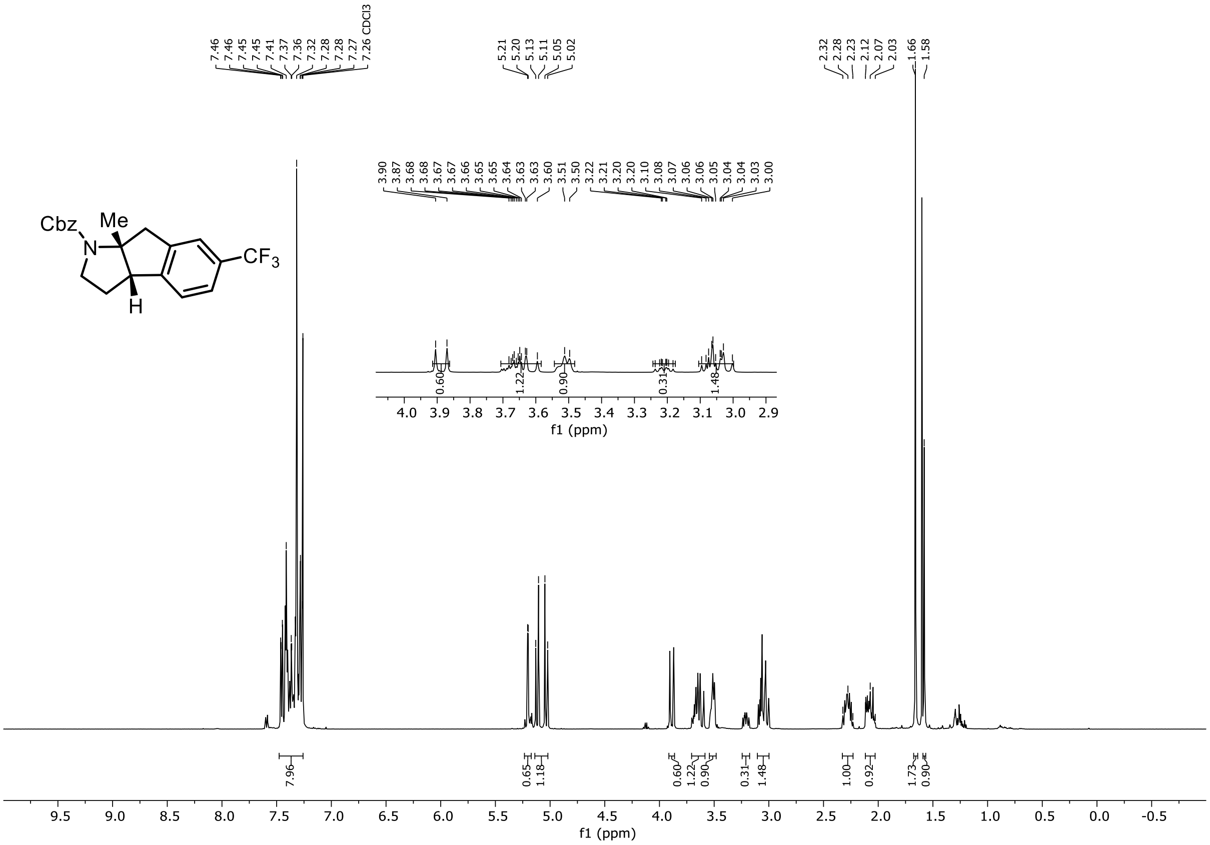

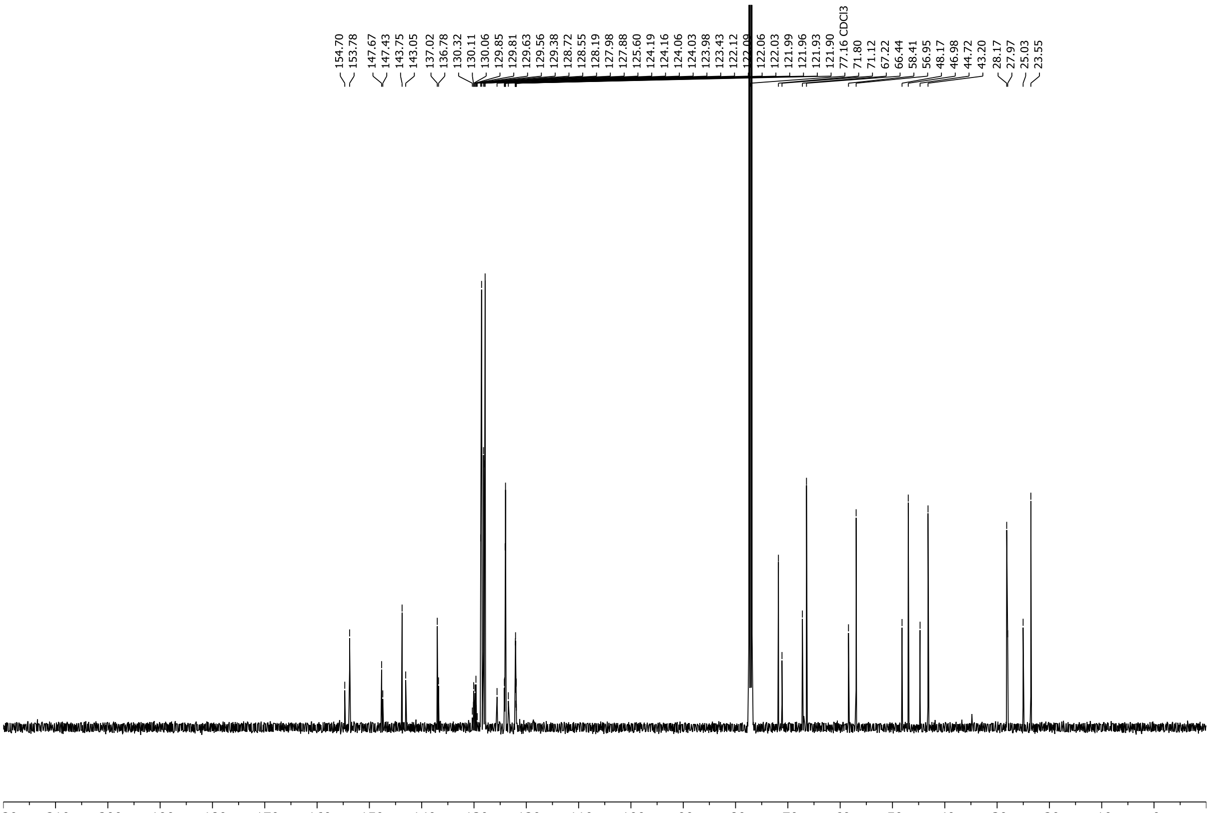

Ethyl 4-methyl-3-(4-nitrophenyl)pent-4-enoate

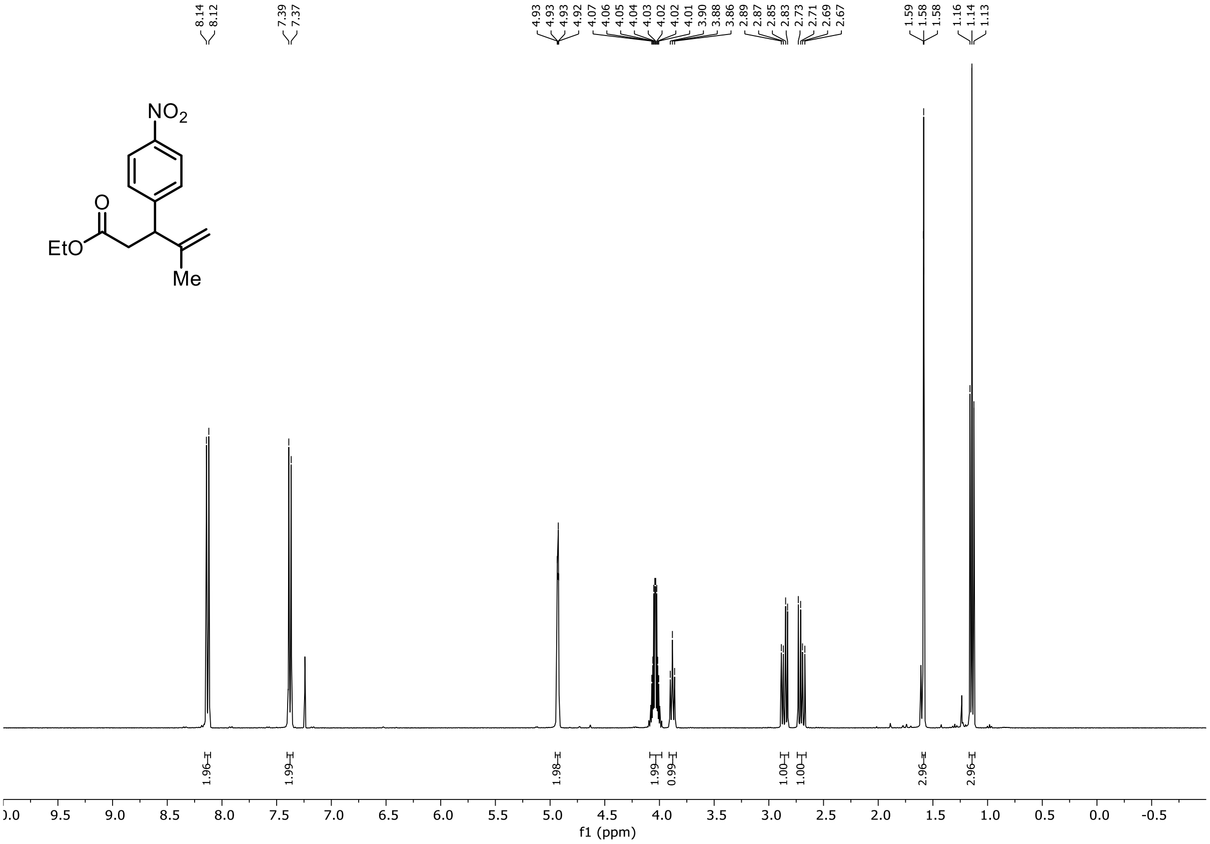

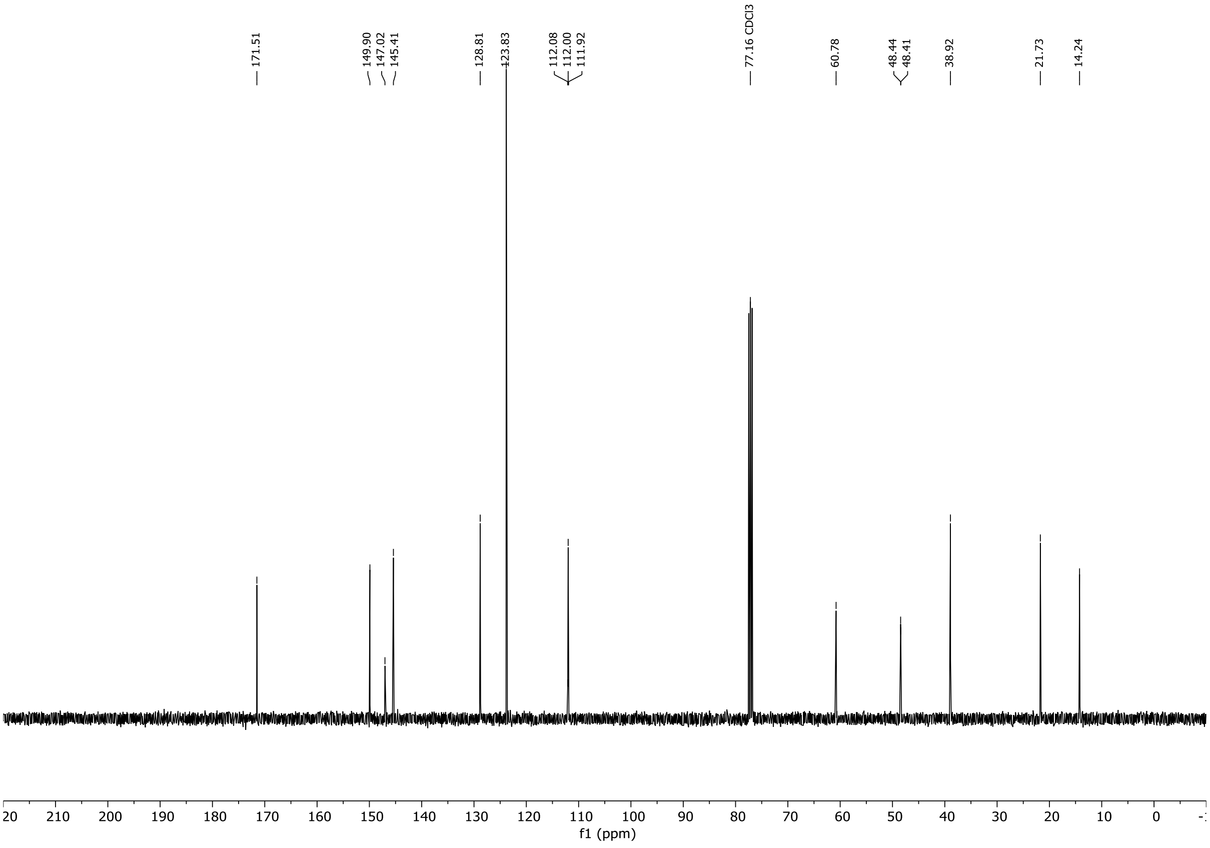

Benzyl (4-methyl-3-(4-nitrophenyl)pent-4-en-1-yl)((perfluorobenzoyl)oxy)carbamate (1g)

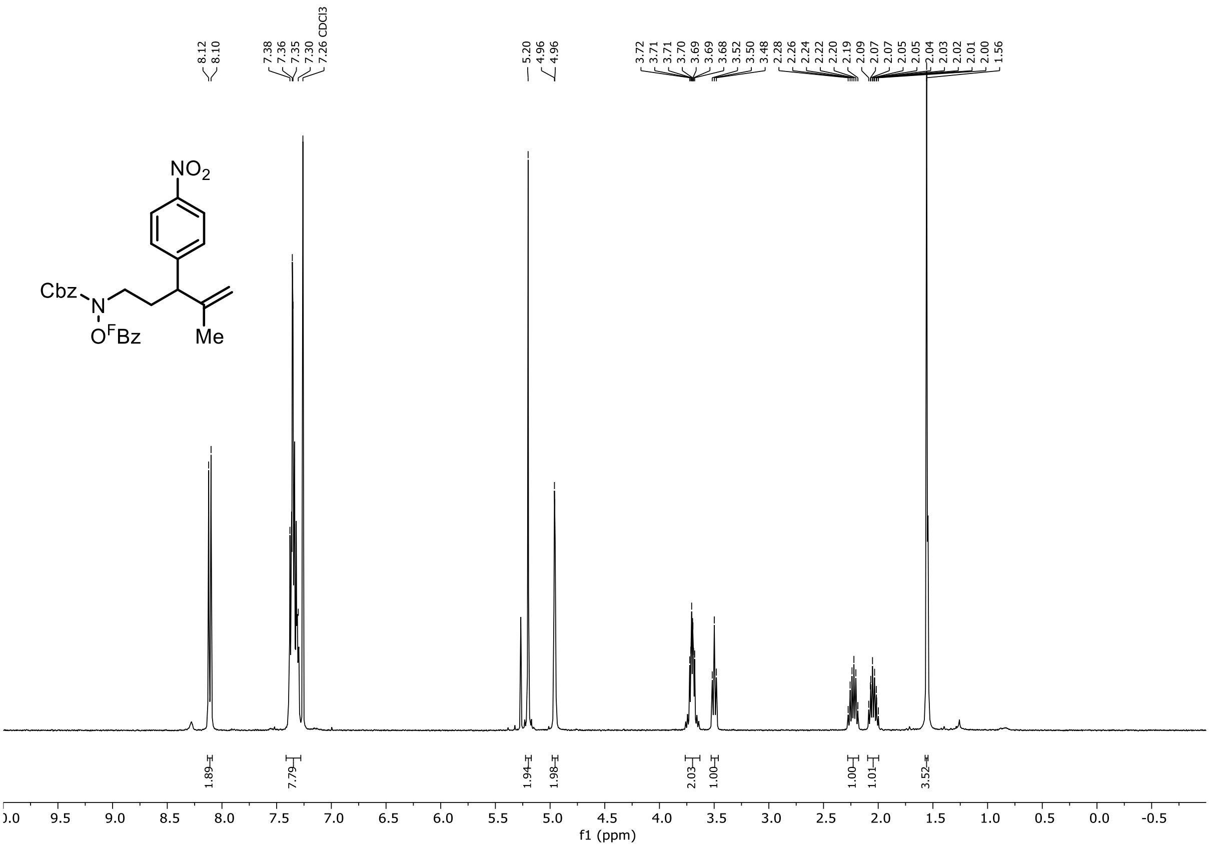

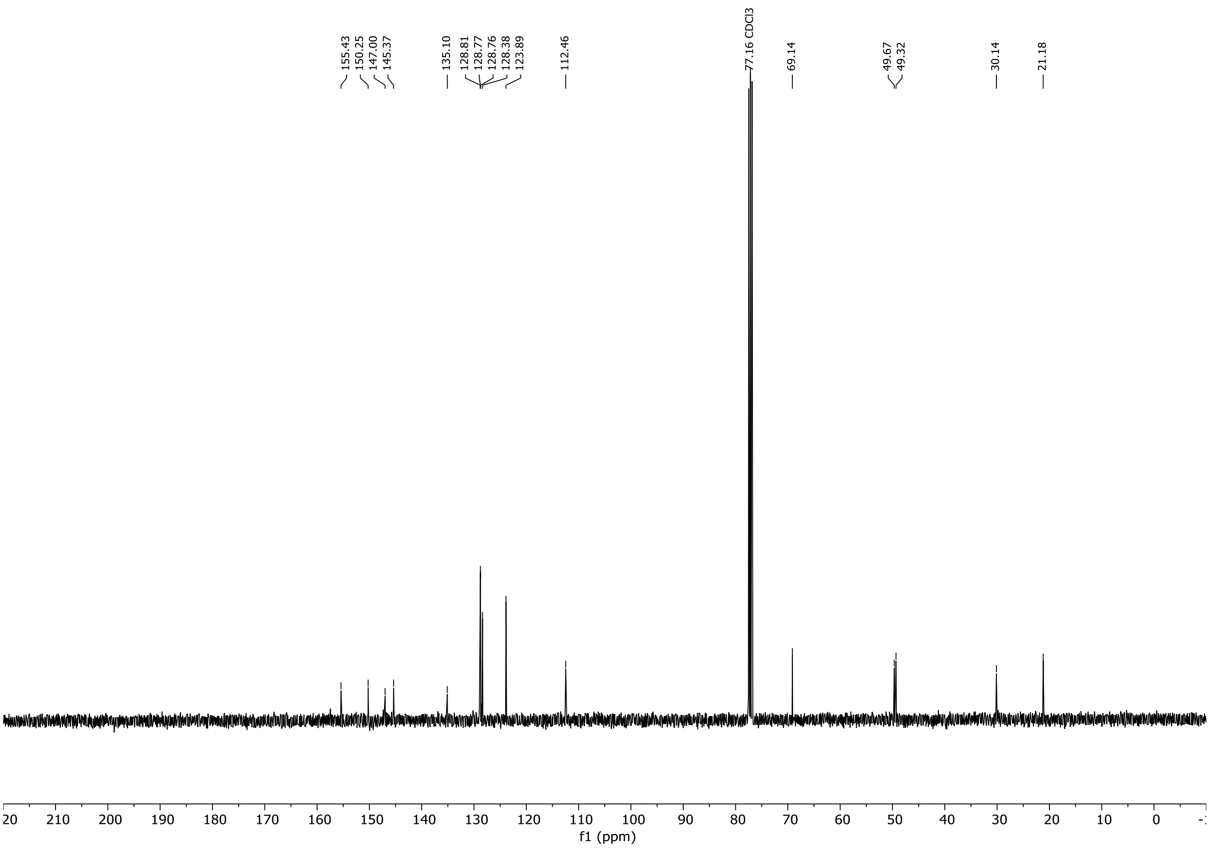

Benzyl 8a-methyl-6-nitro-3,3a,8,8a-tetrahydroindeno[2,1-*b*]pyrrole-1(2*H*)-carboxylate (2g)

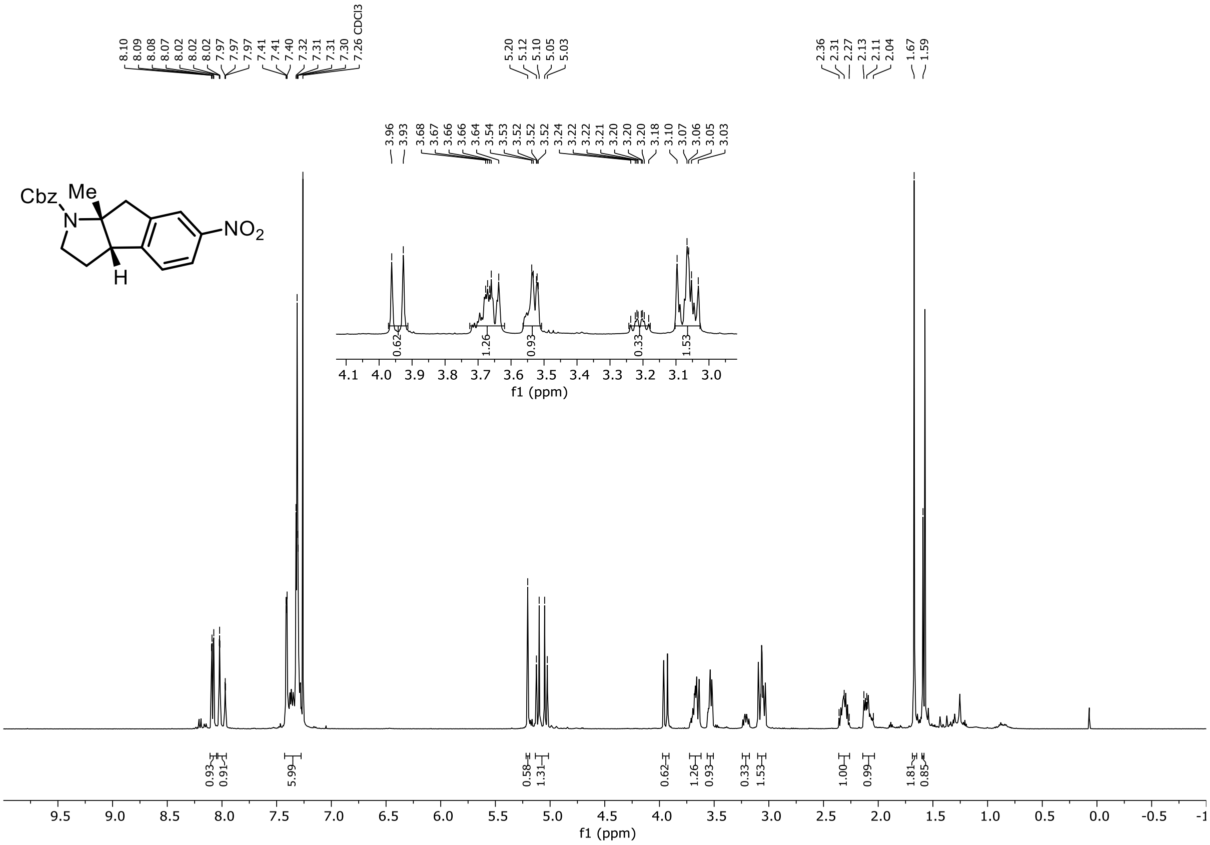

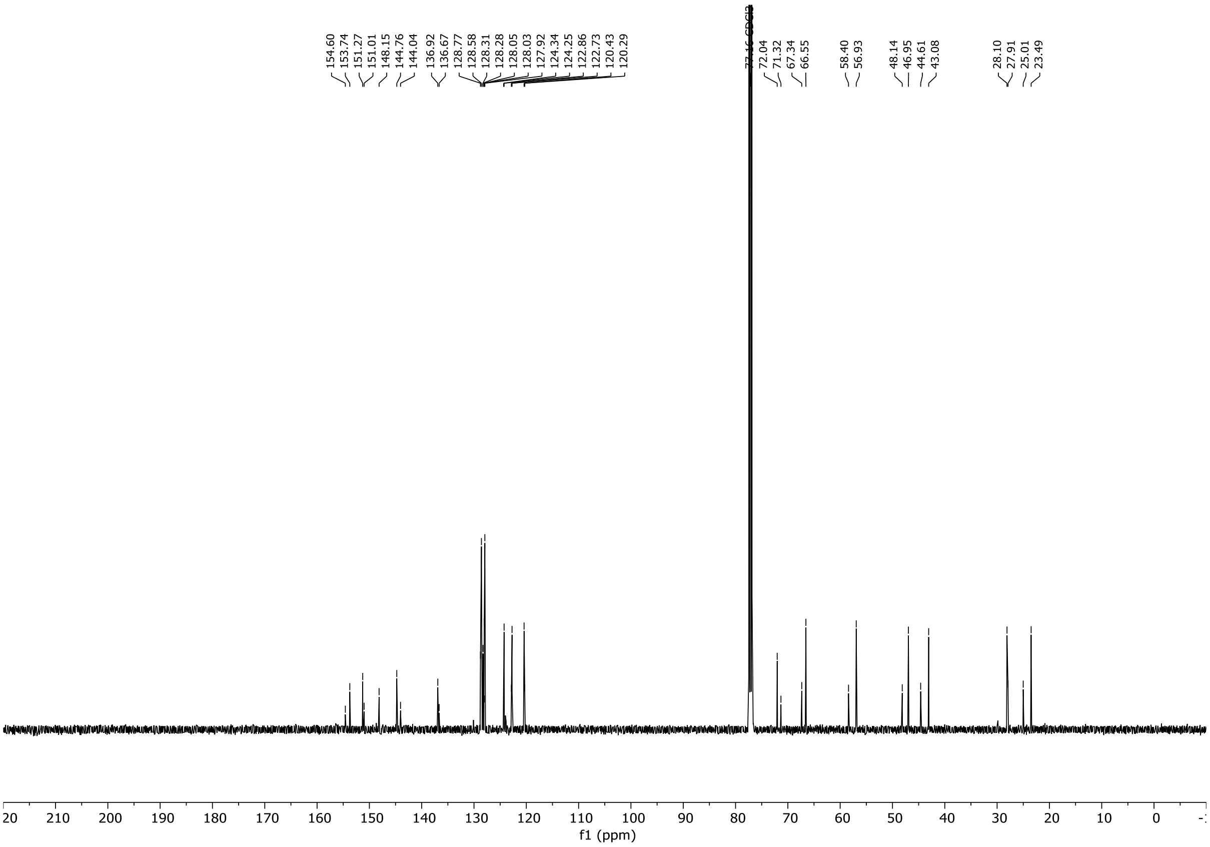

3,4-Diphenylpent-4-enoic acid

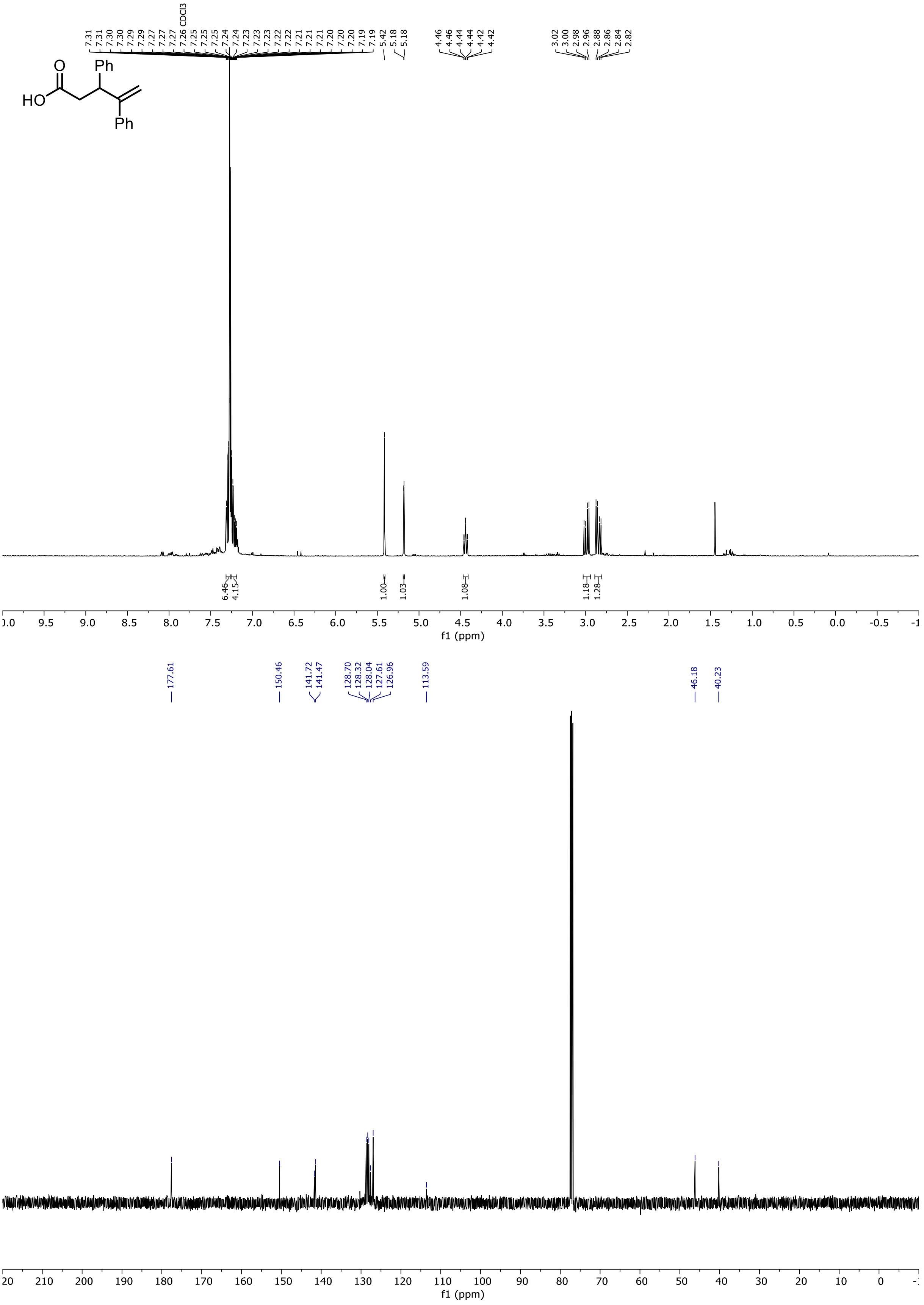

Benzyl (3,4-diphenylpent-4-en-1-yl)((perfluorobenzoyl)oxy)carbamate (1h)

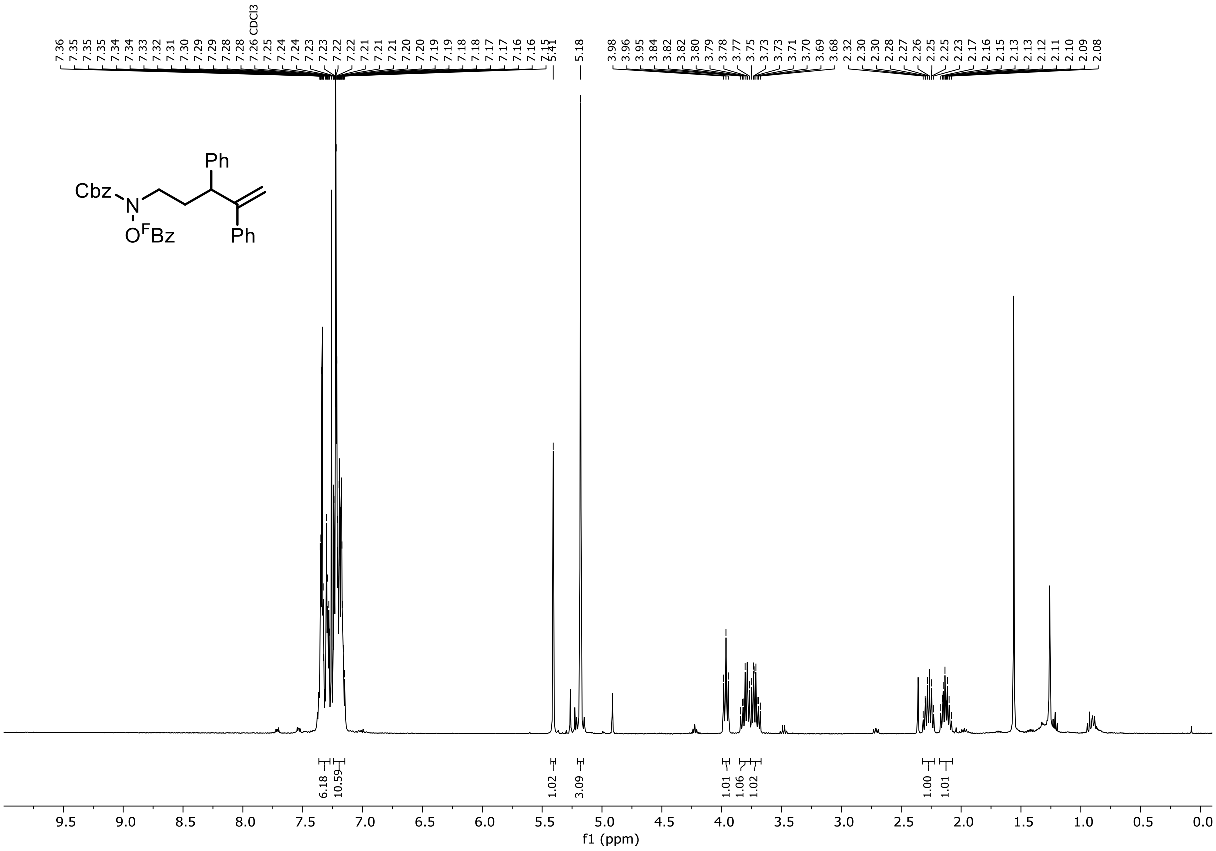

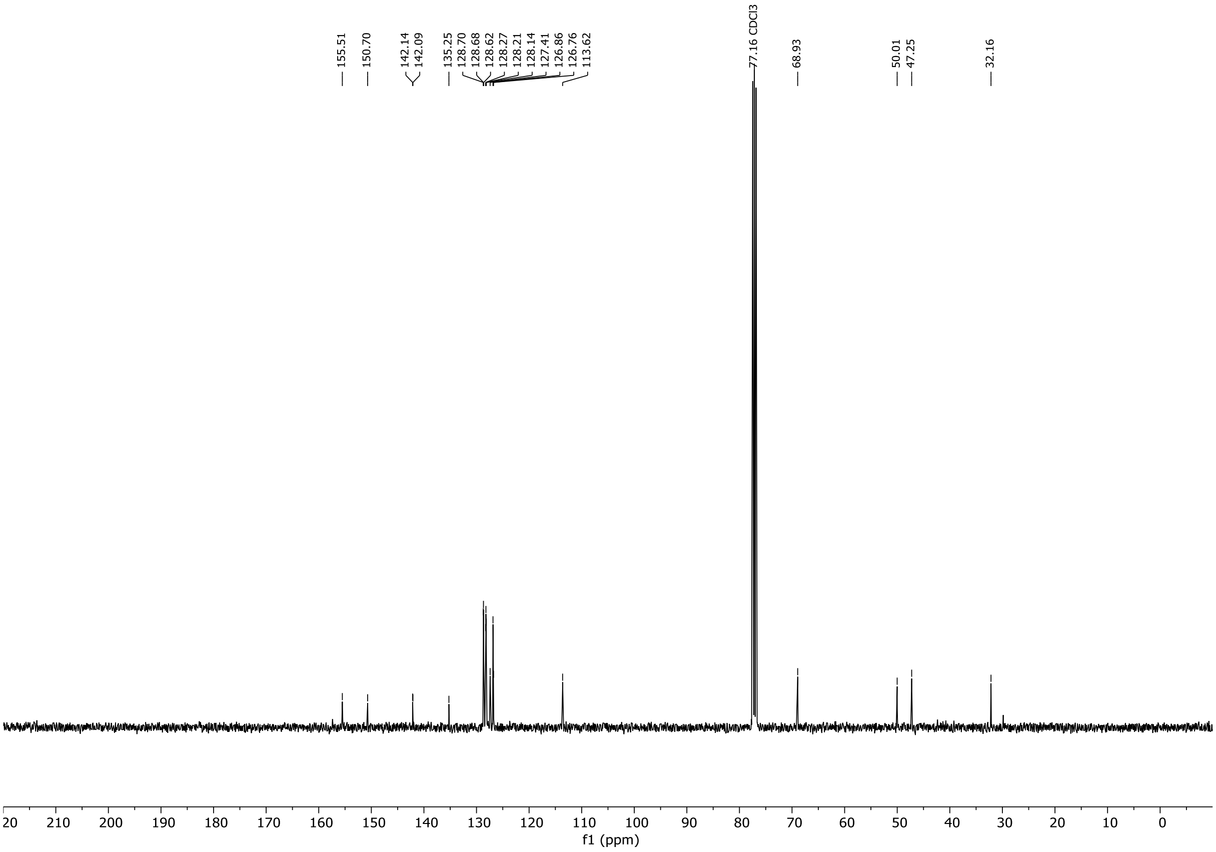

Benzyl 8a-phenyl-3,3a,8,8a-tetrahydroindeno[2,1-*b*]pyrrole-1(2*H*)-carboxylate (2h)

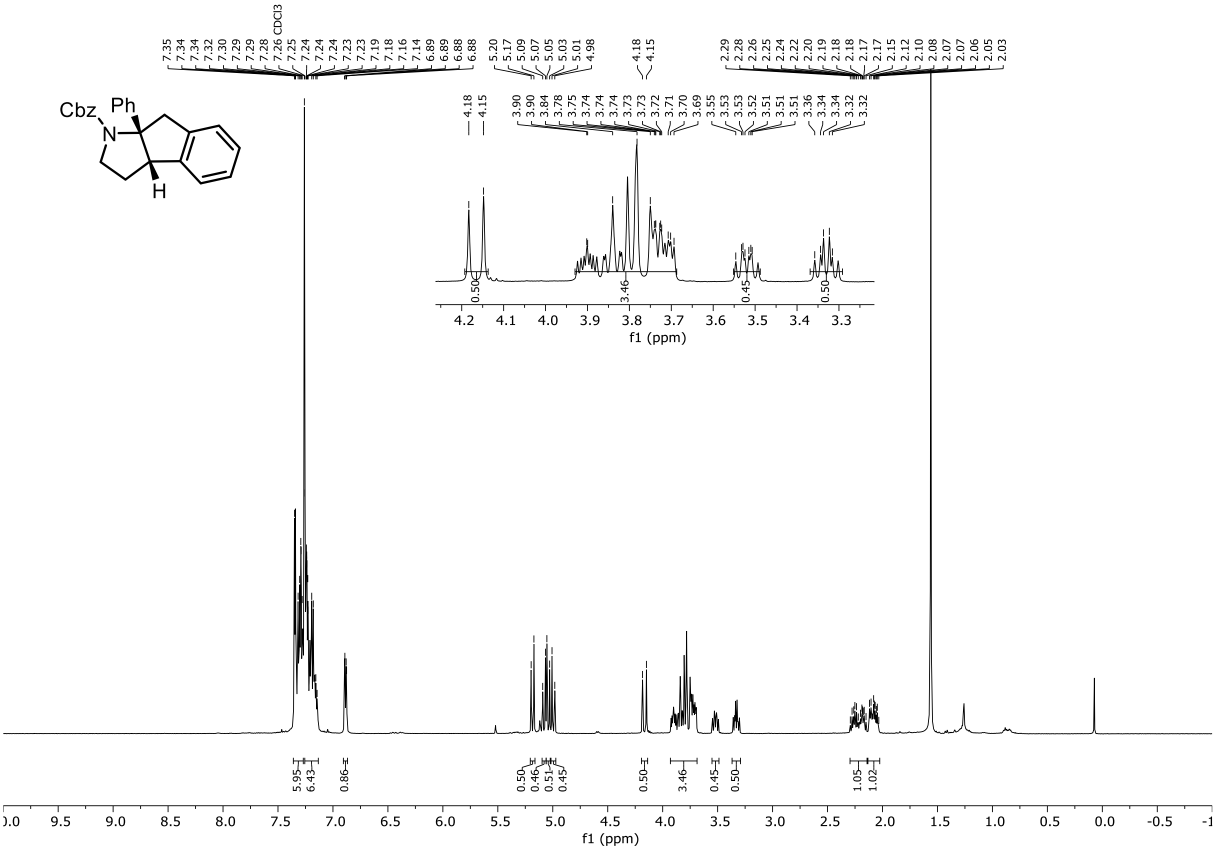

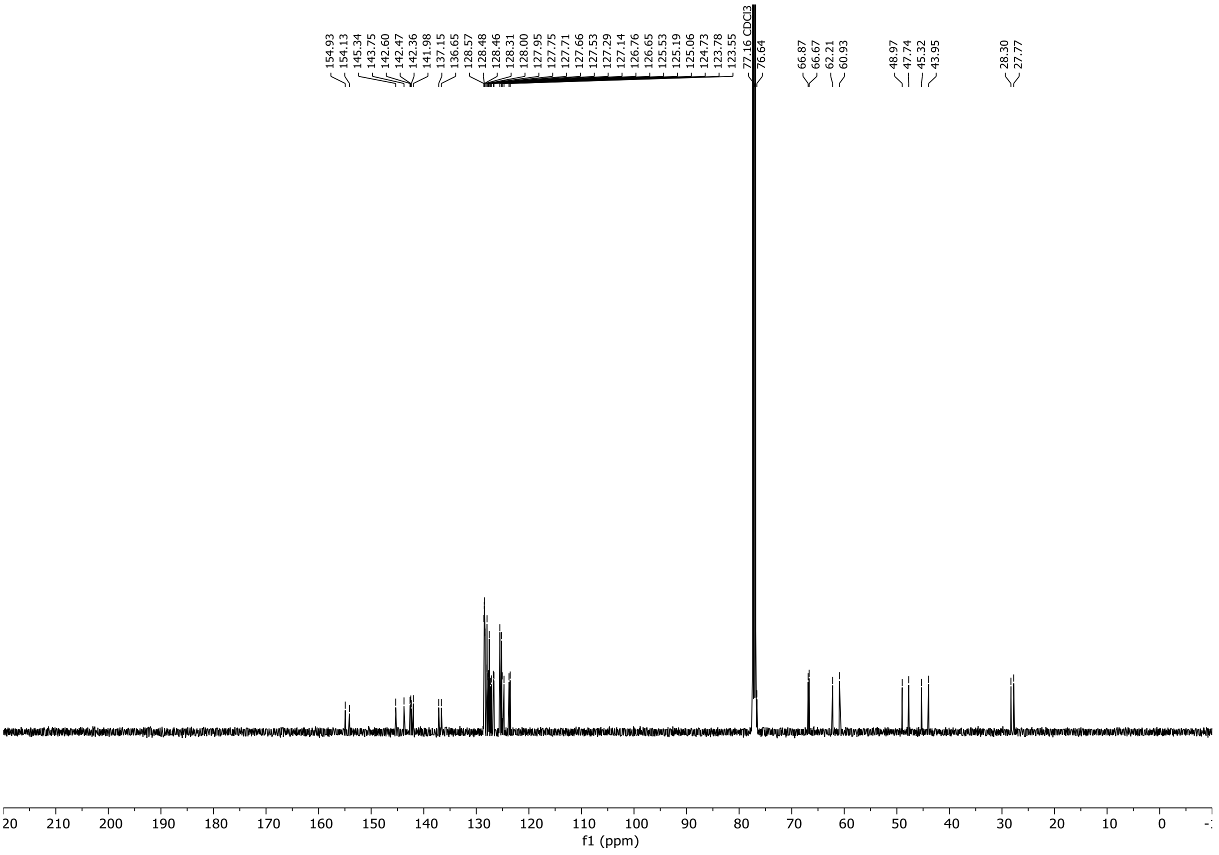

5-Methyl-4-methylene-3-phenylhexanoic acid

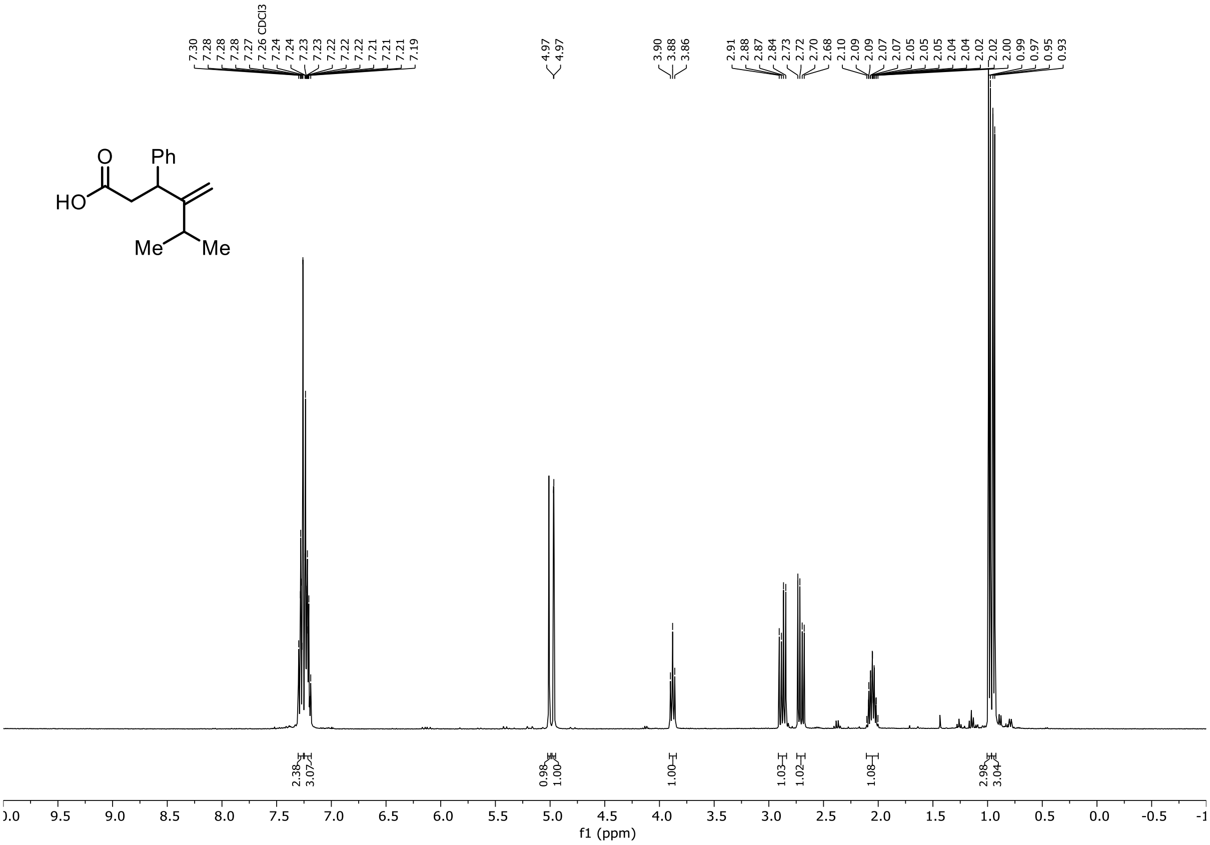

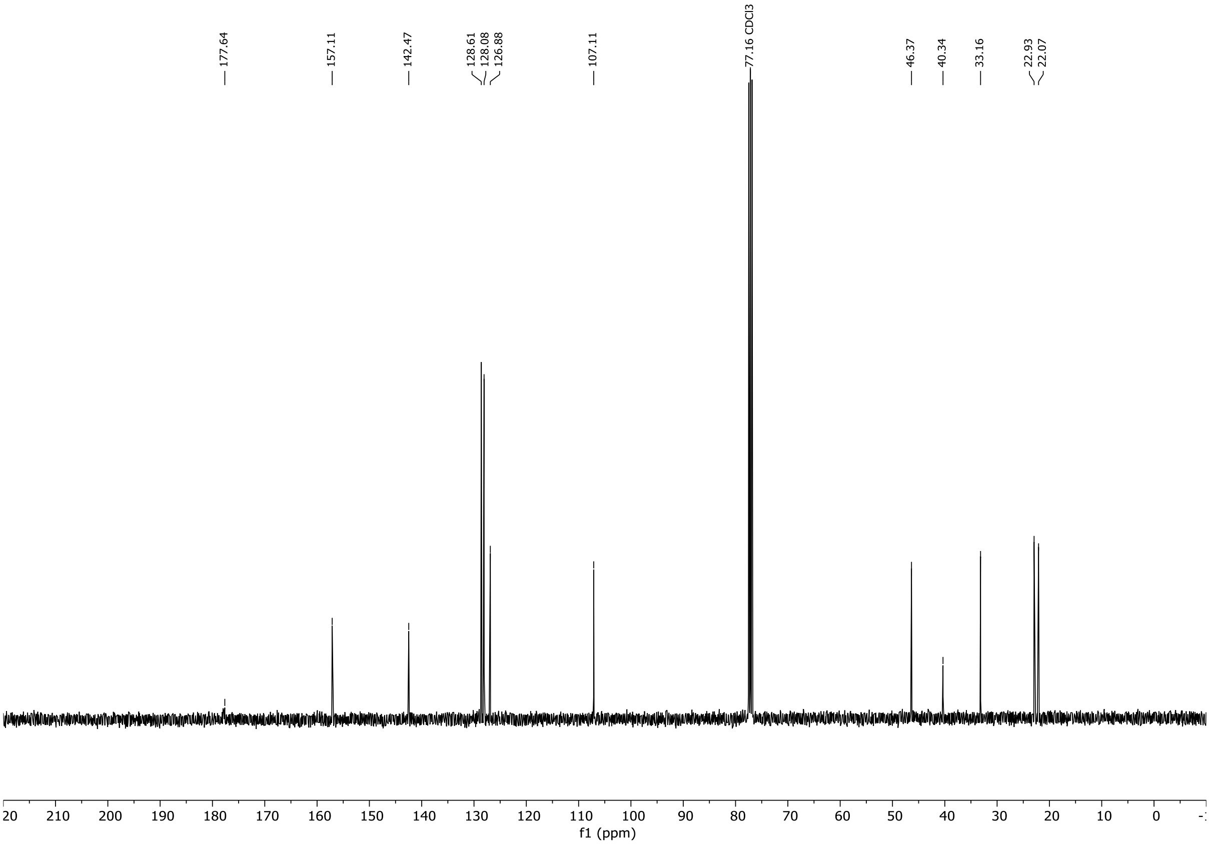

5-Methyl-4-methylene-3-phenylhexan-1-ol

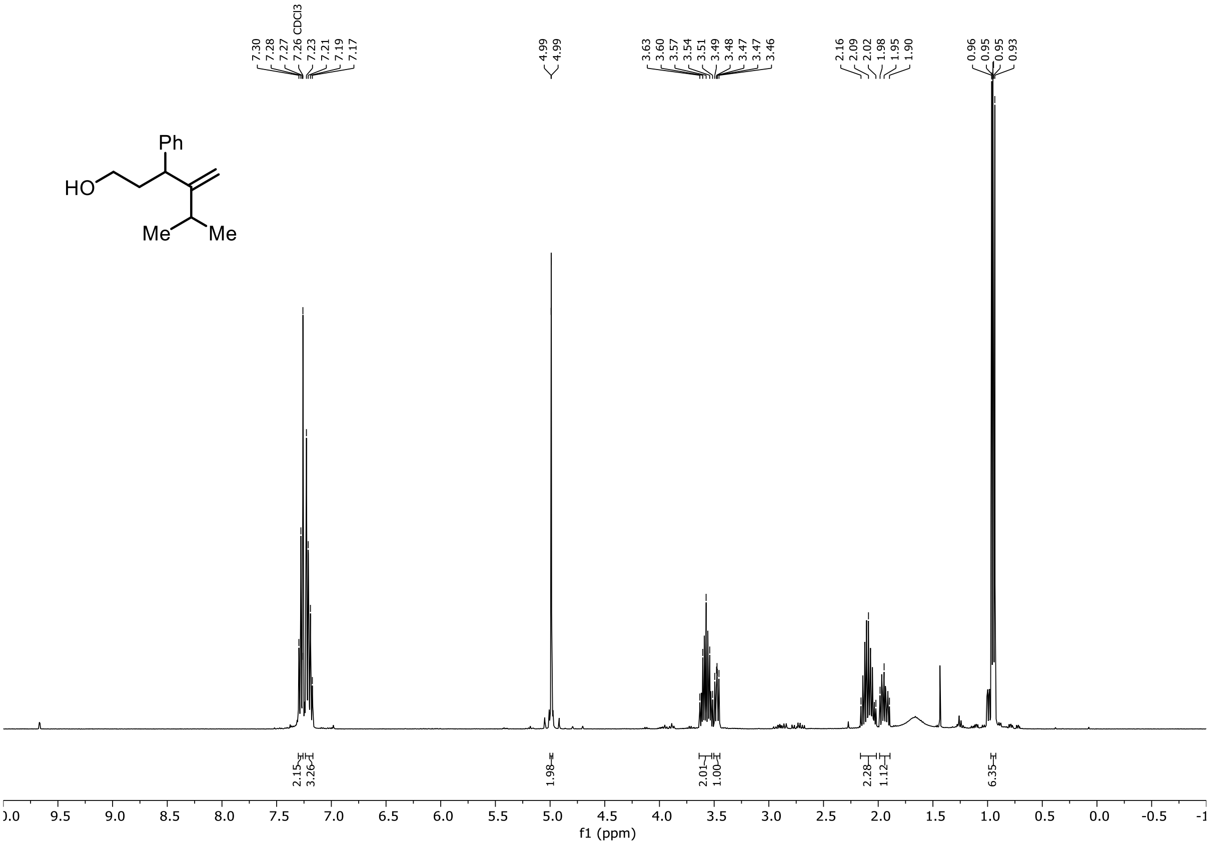

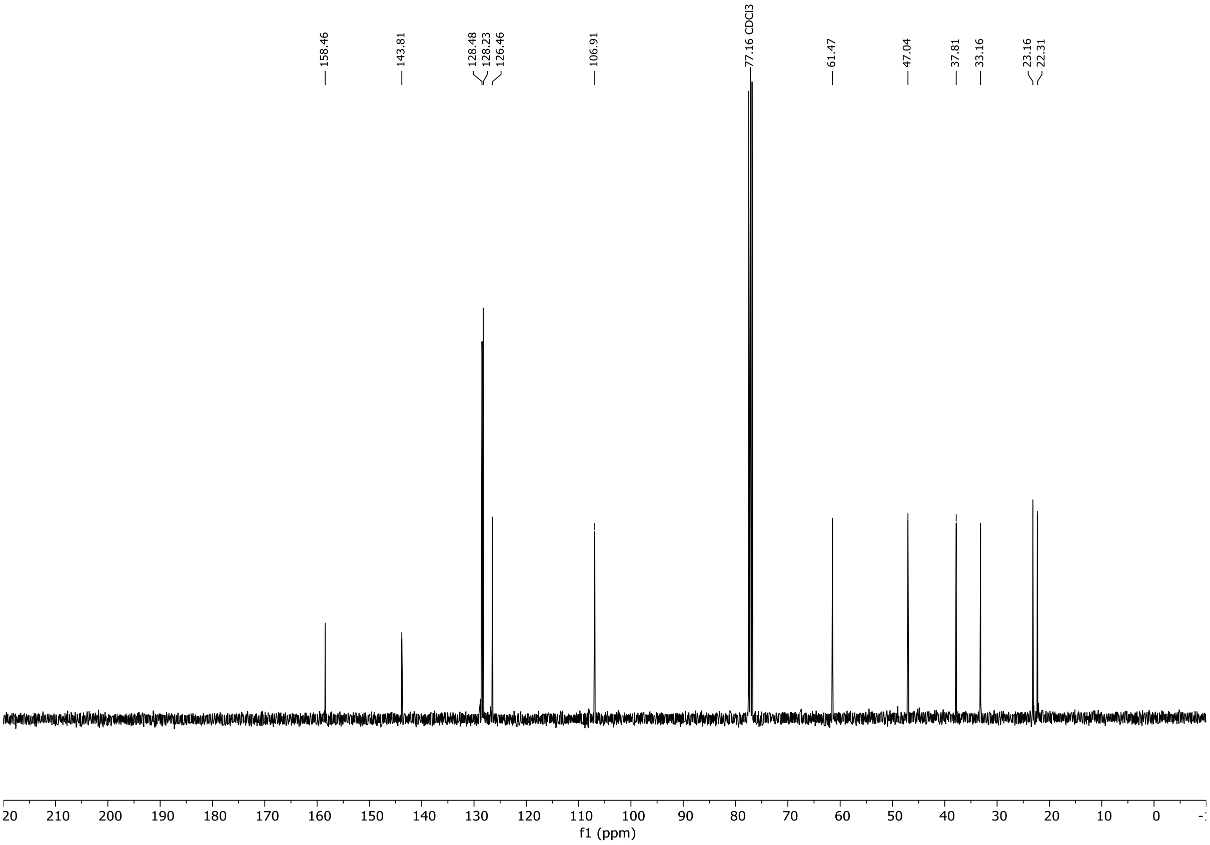

Benzyl (5-methyl-4-methylene-3-phenylhexyl)((perfluorobenzoyl)oxy)carbamate (1i)

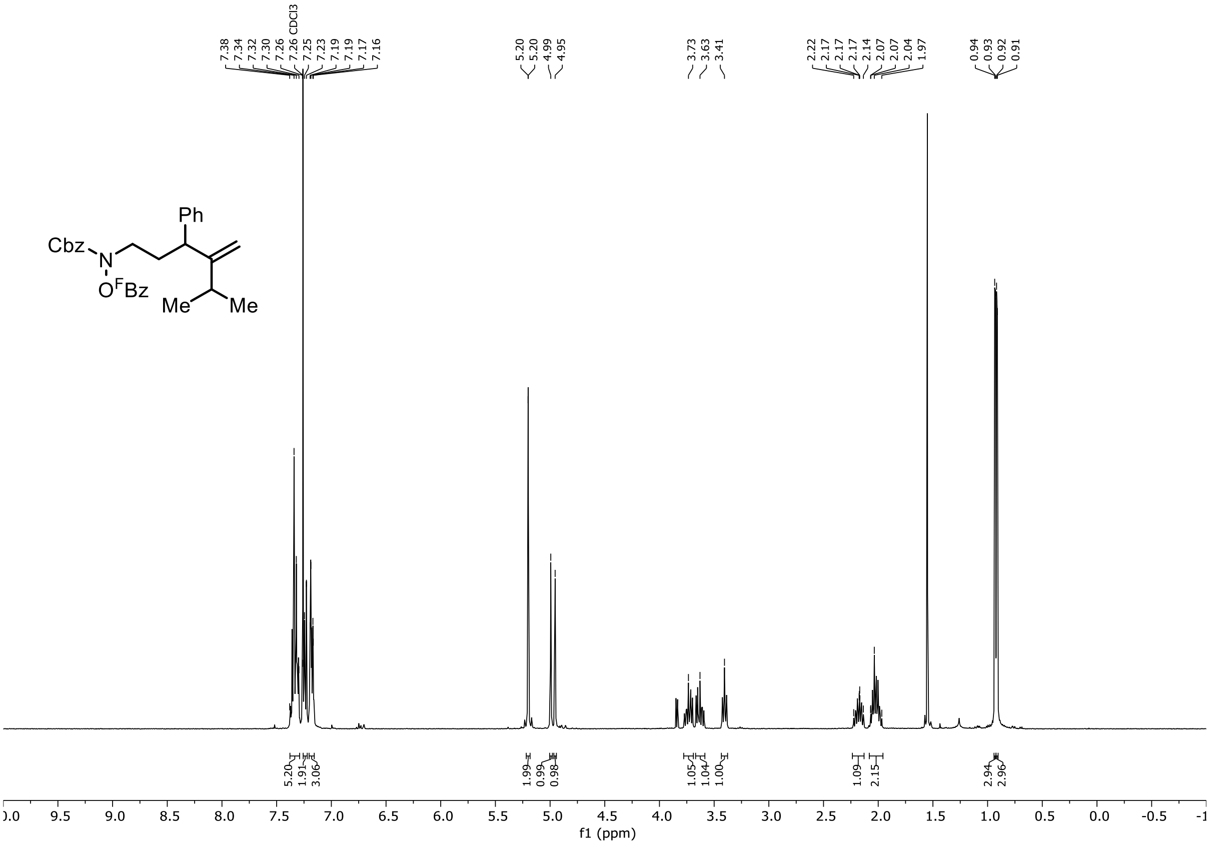

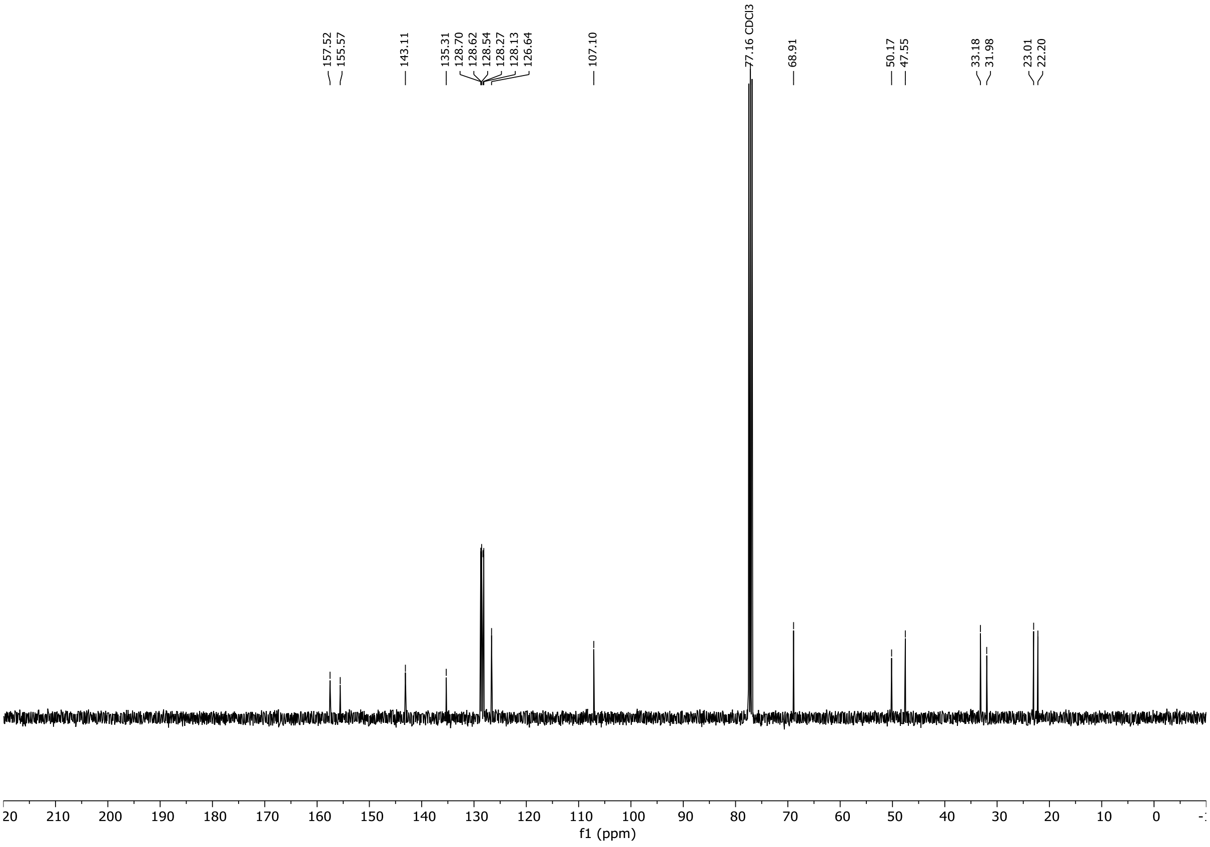

Benzyl 8a-isopropyl-3,3a,8,8a-tetrahydroindeno[2,1-*b*]pyrrole-1(2*H*)-carboxylate (2i)

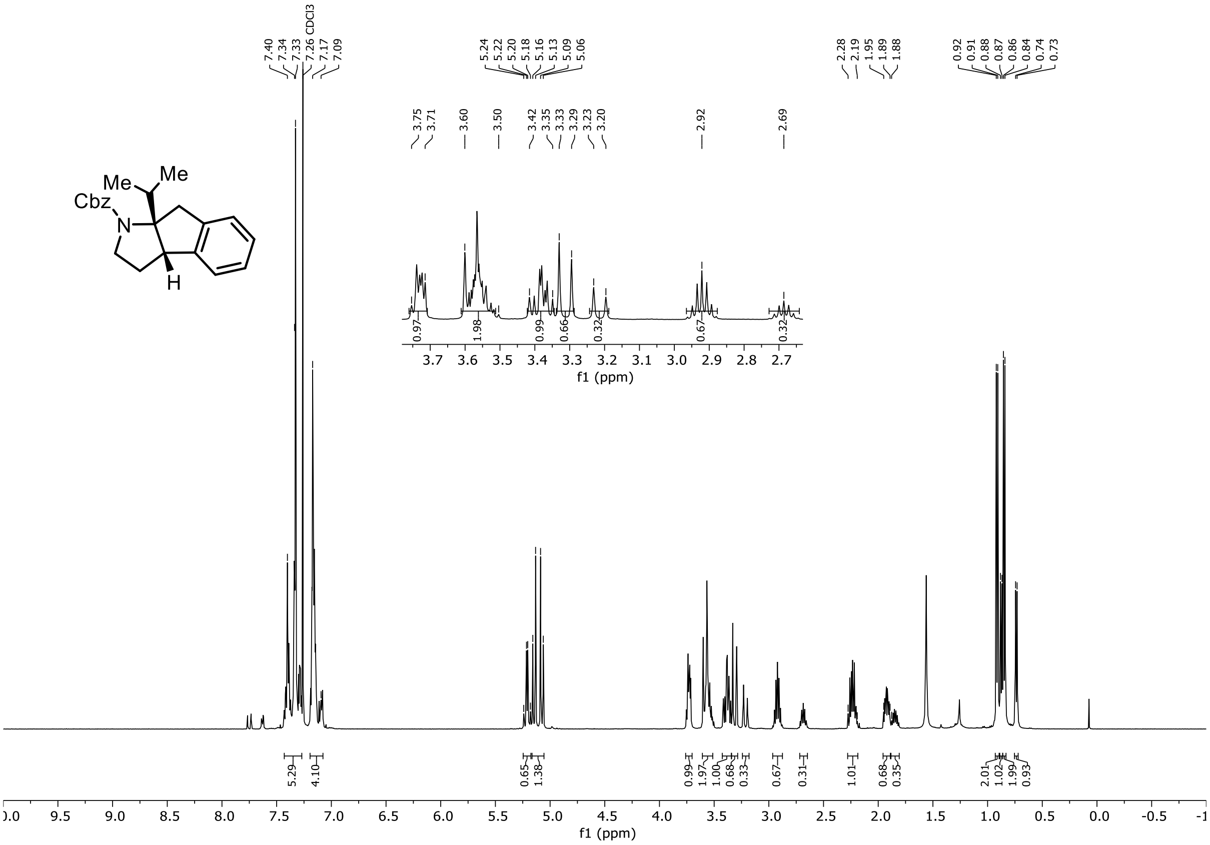

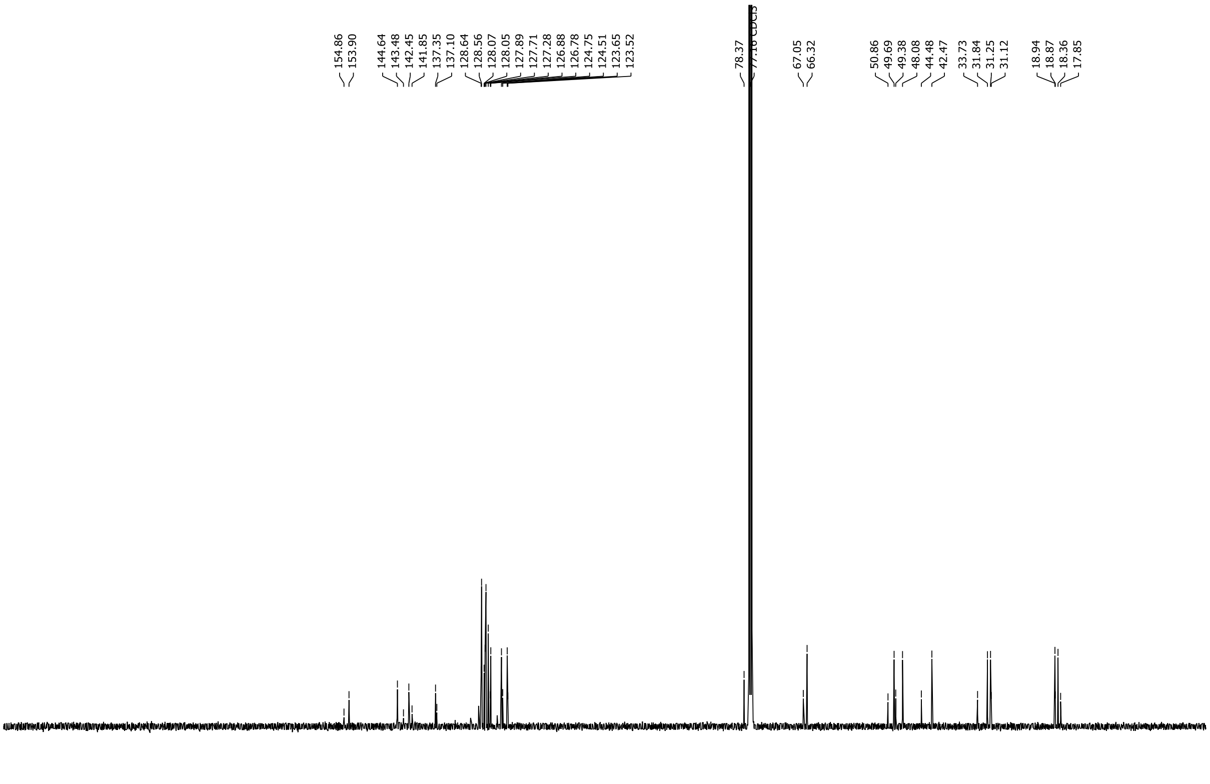

(*E*)-3-(3-Chlorophenyl)-2-methylprop-2-en-1-ol

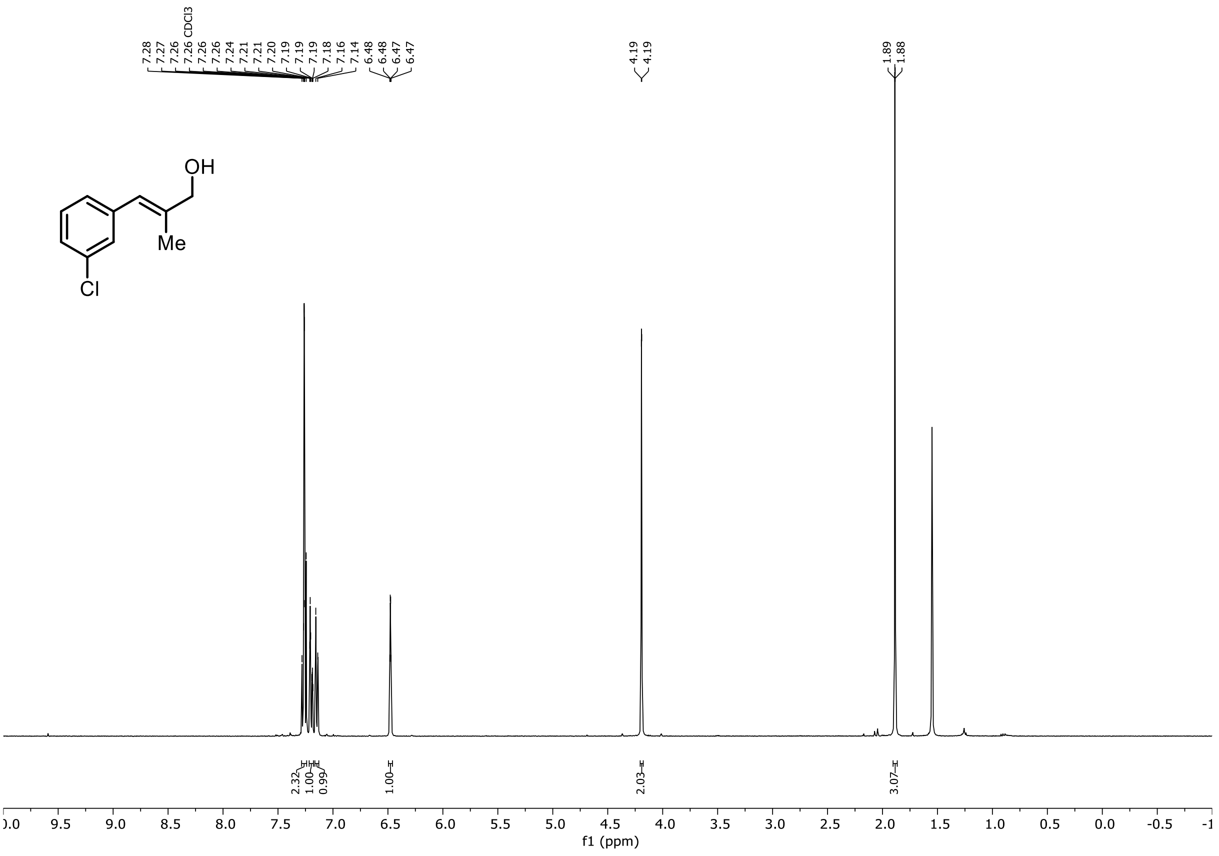

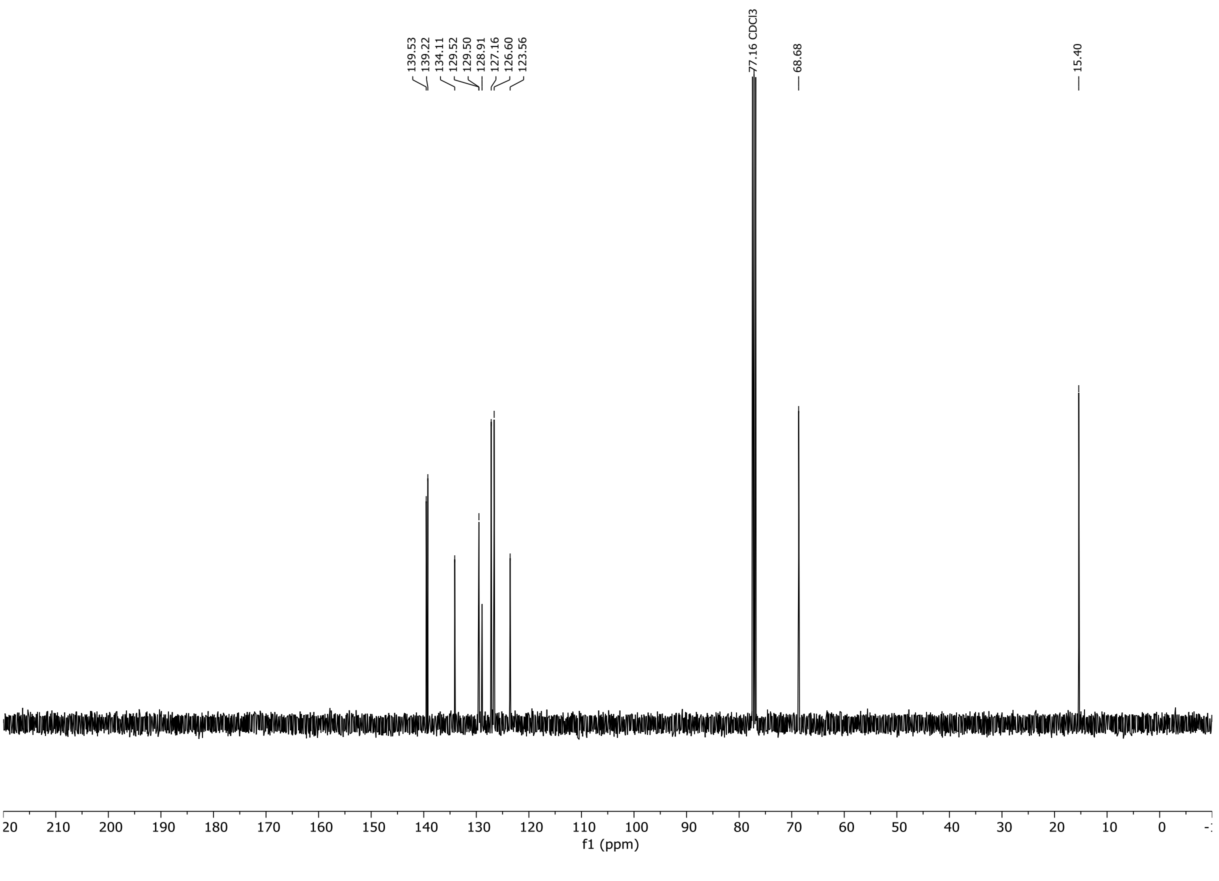

3-(3-Chlorophenyl)-4-methylpent-4-enoic acid

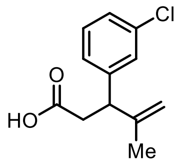

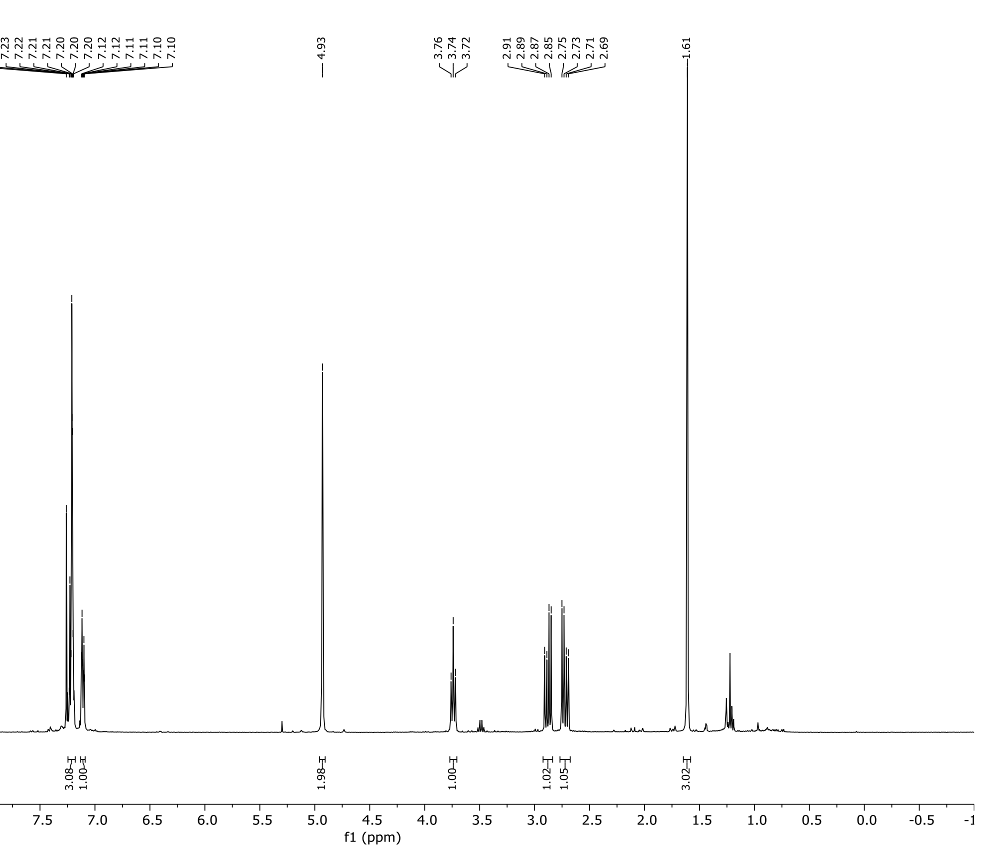

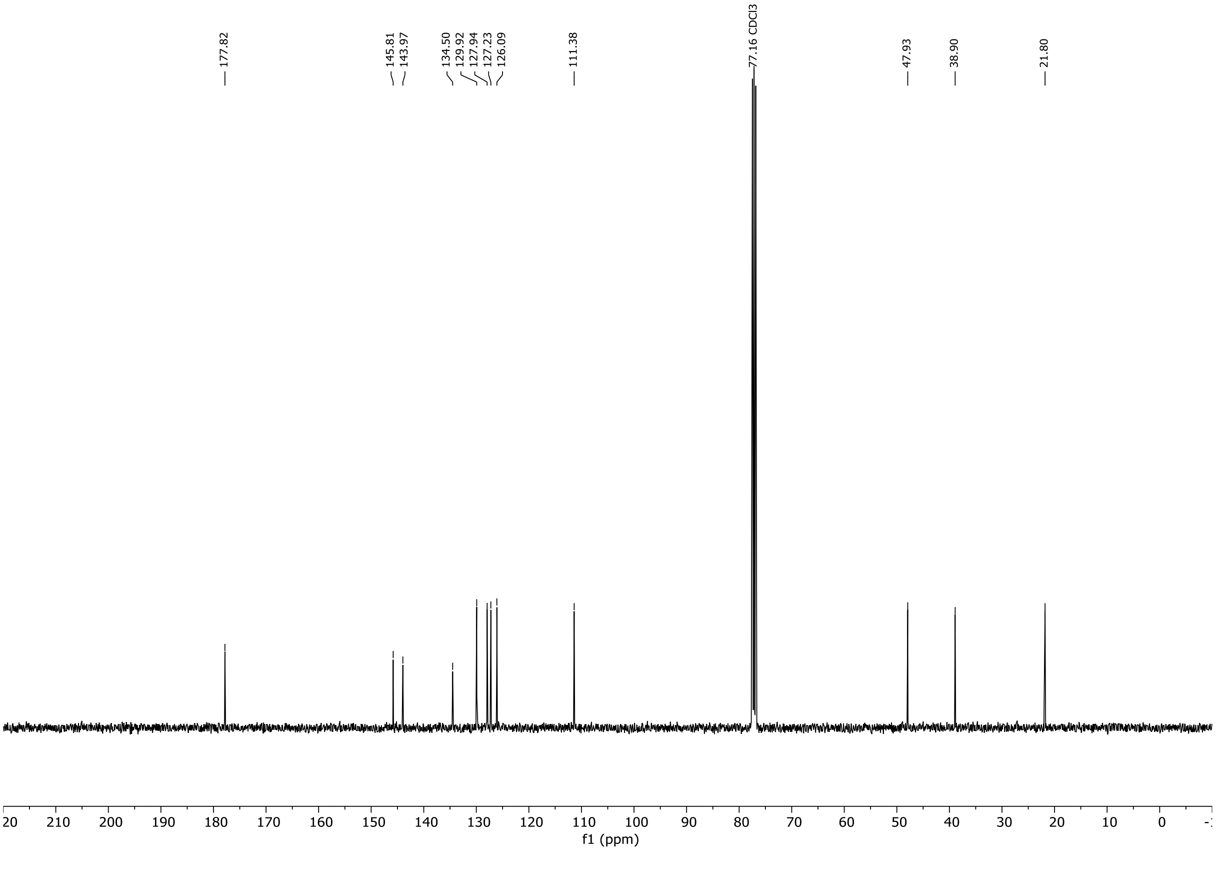

3-(3-Chlorophenyl)-4-methylpent-4-en-1-ol

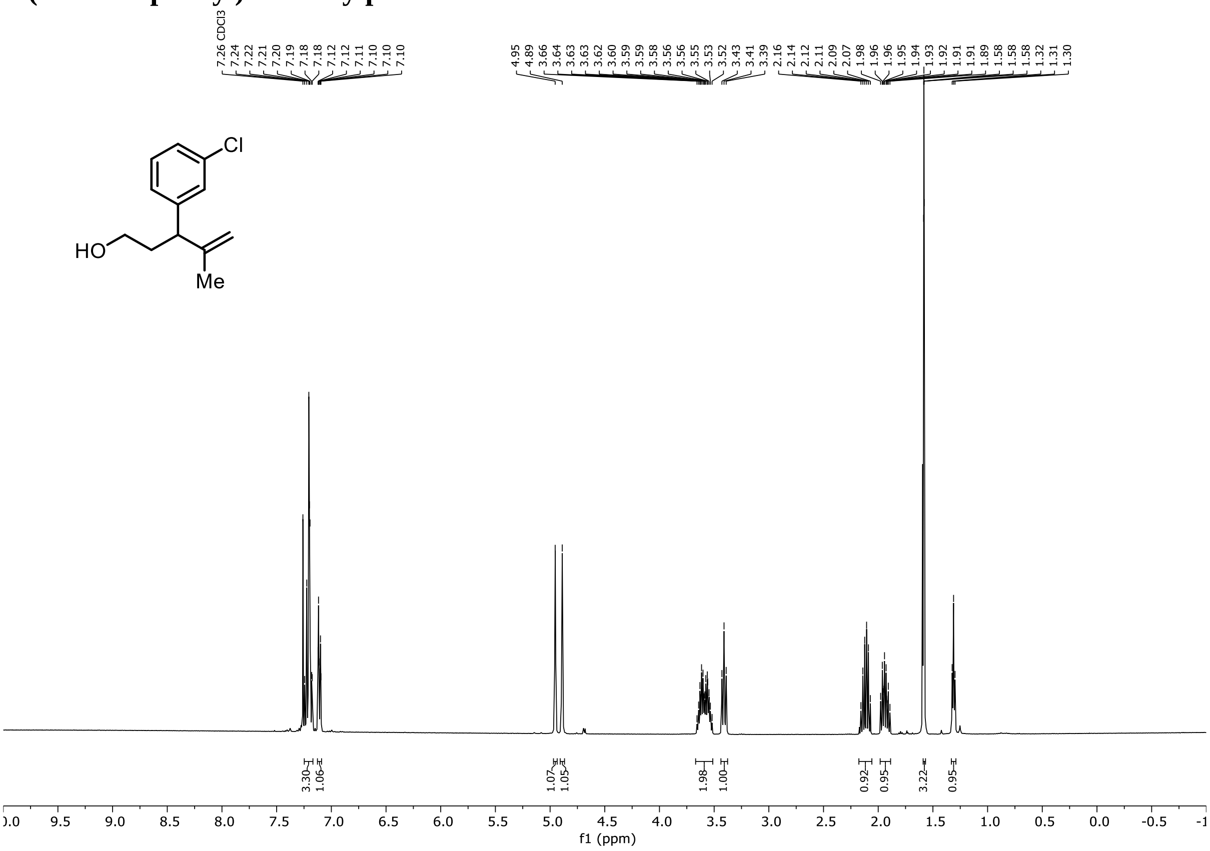

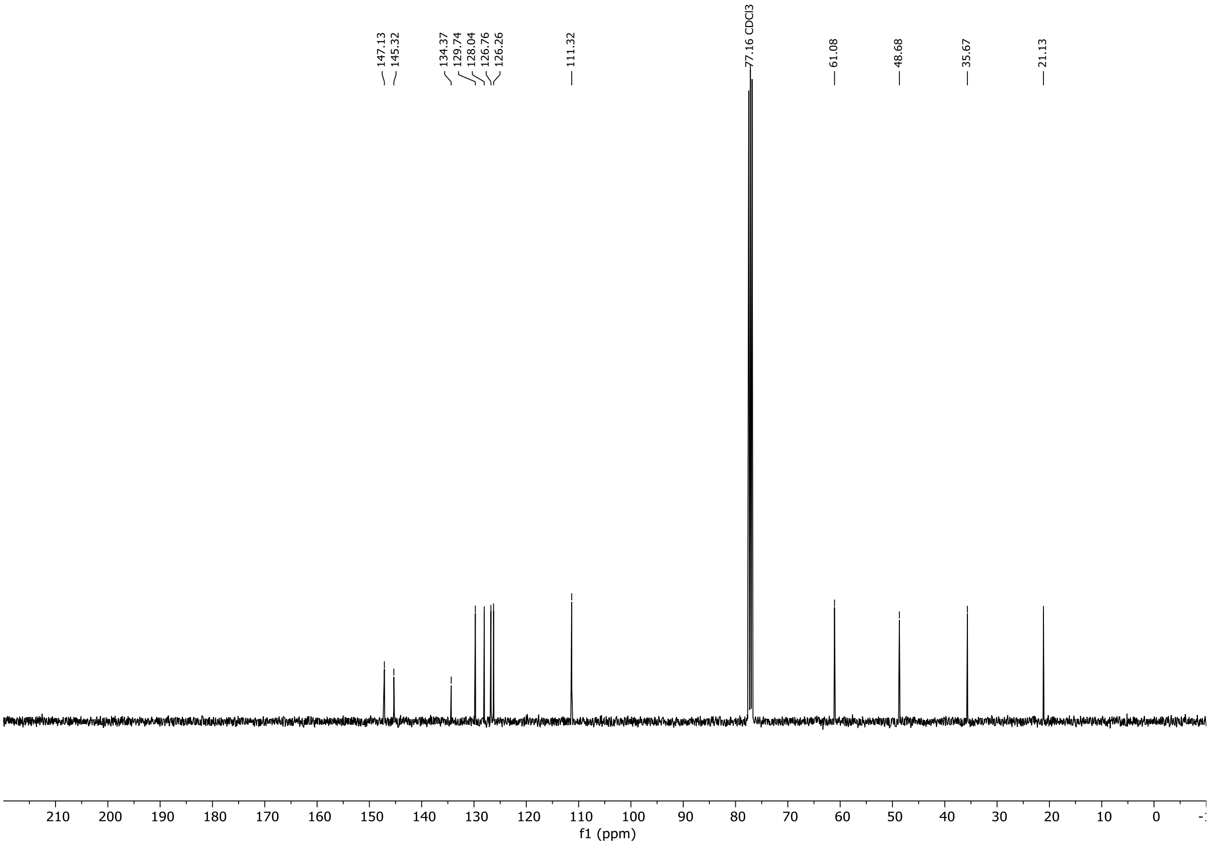

Benzyl (3-(3-chlorophenyl)-4-methylpent-4-en-1-yl)((perfluorobenzoyl)oxy)carbamate (1j)

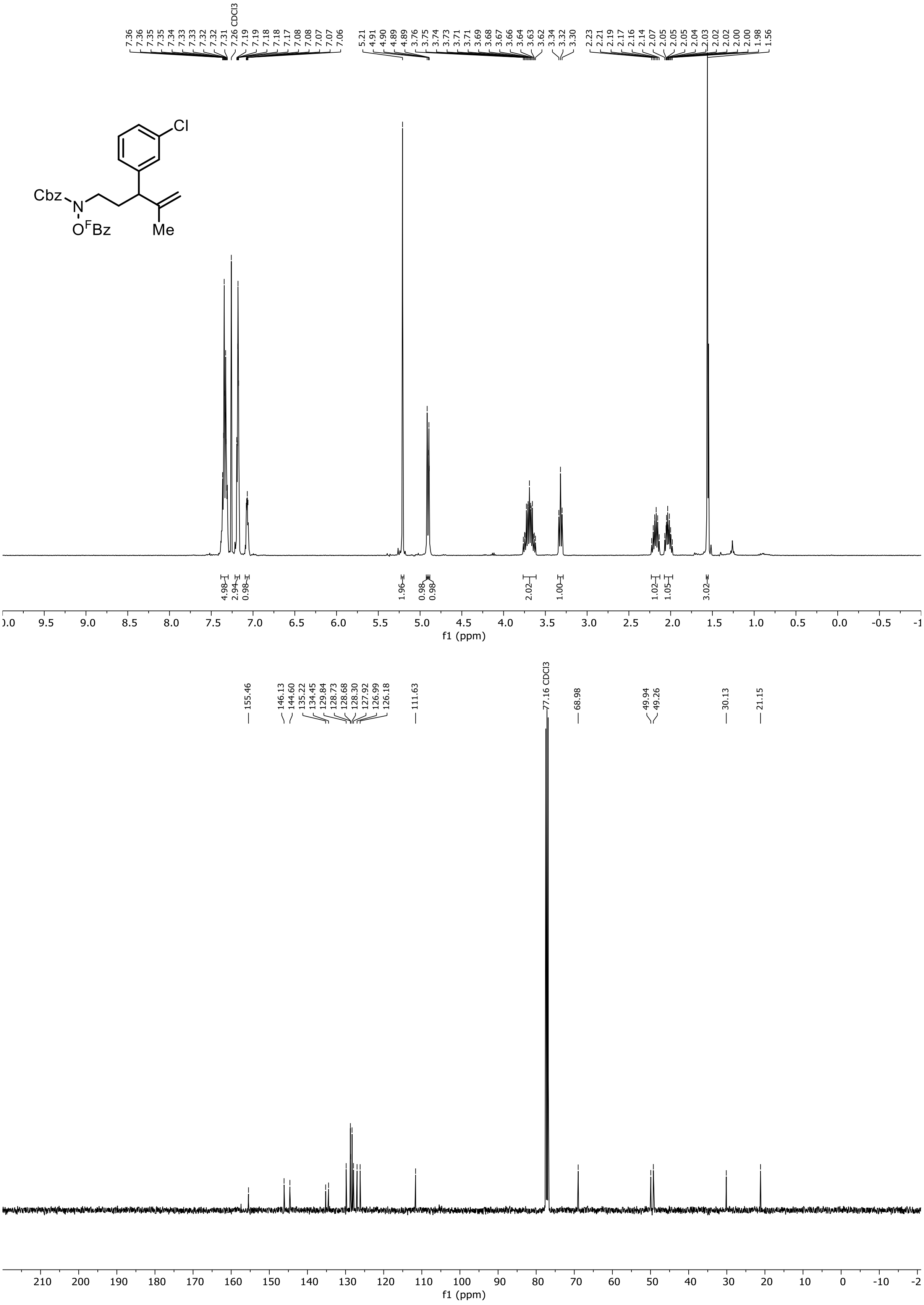

Benzyl 5-chloro-8a-methyl-3,3a,8,8a-tetrahydroindeno[2,1-*b*]pyrrole-1(2*H*)-carboxylate (2j)

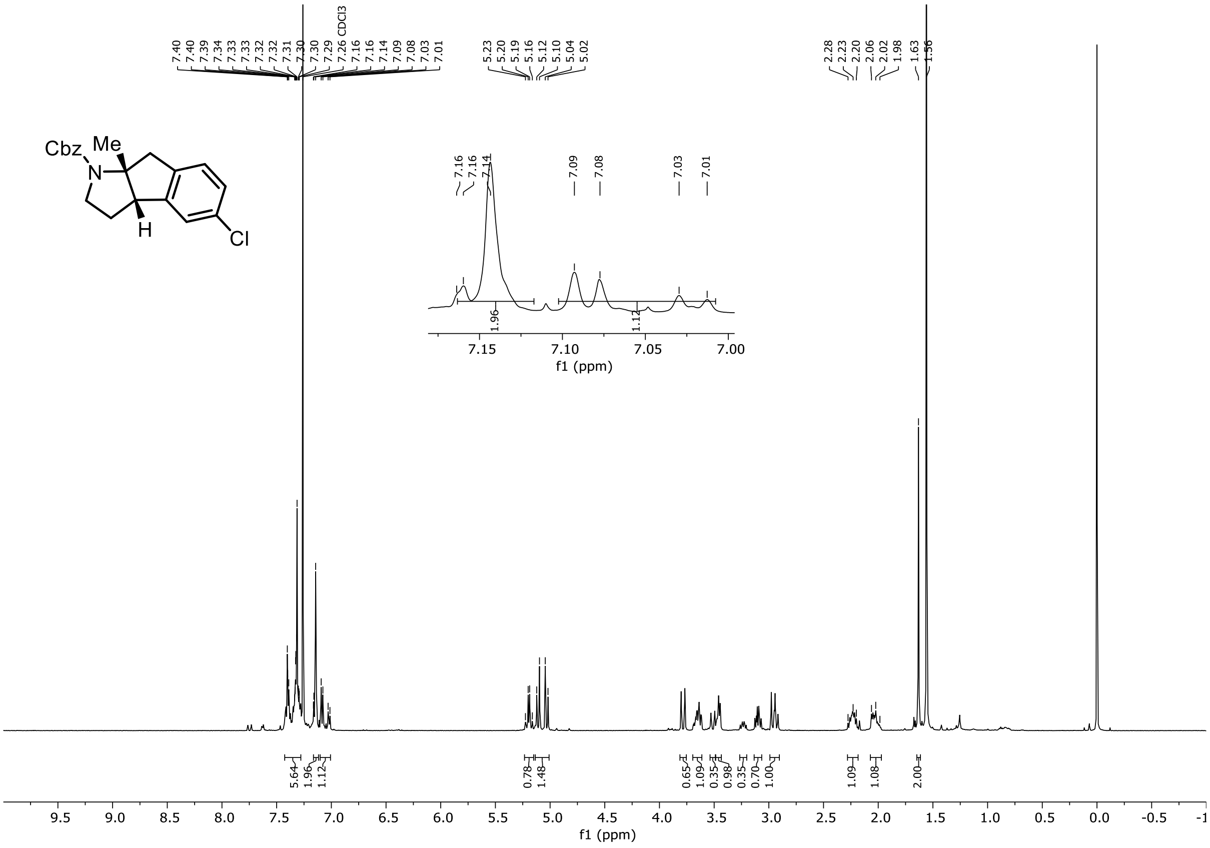

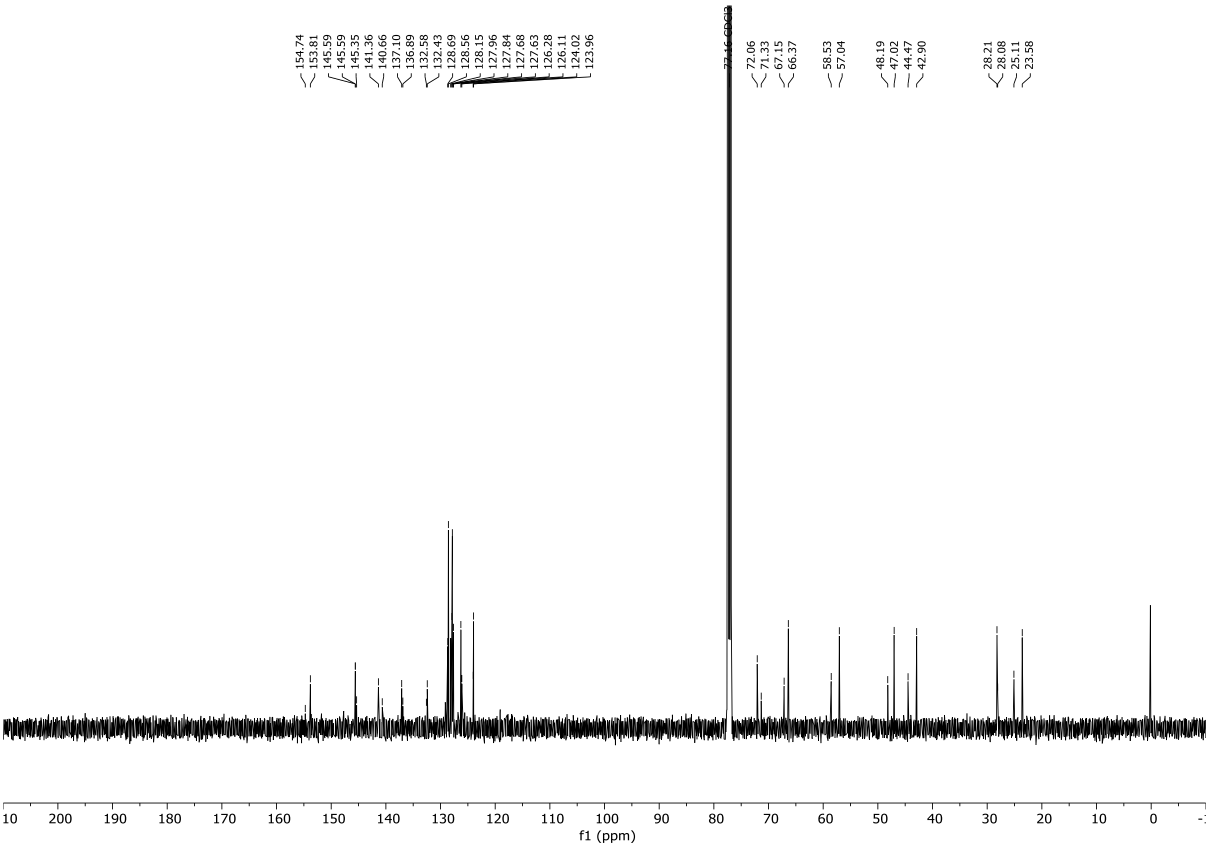

Benzyl 7-chloro-8a-methyl-3,3a,8,8a-tetrahydroindeno[2,1-*b*]pyrrole-1(2*H*)-carboxylate (2j’)

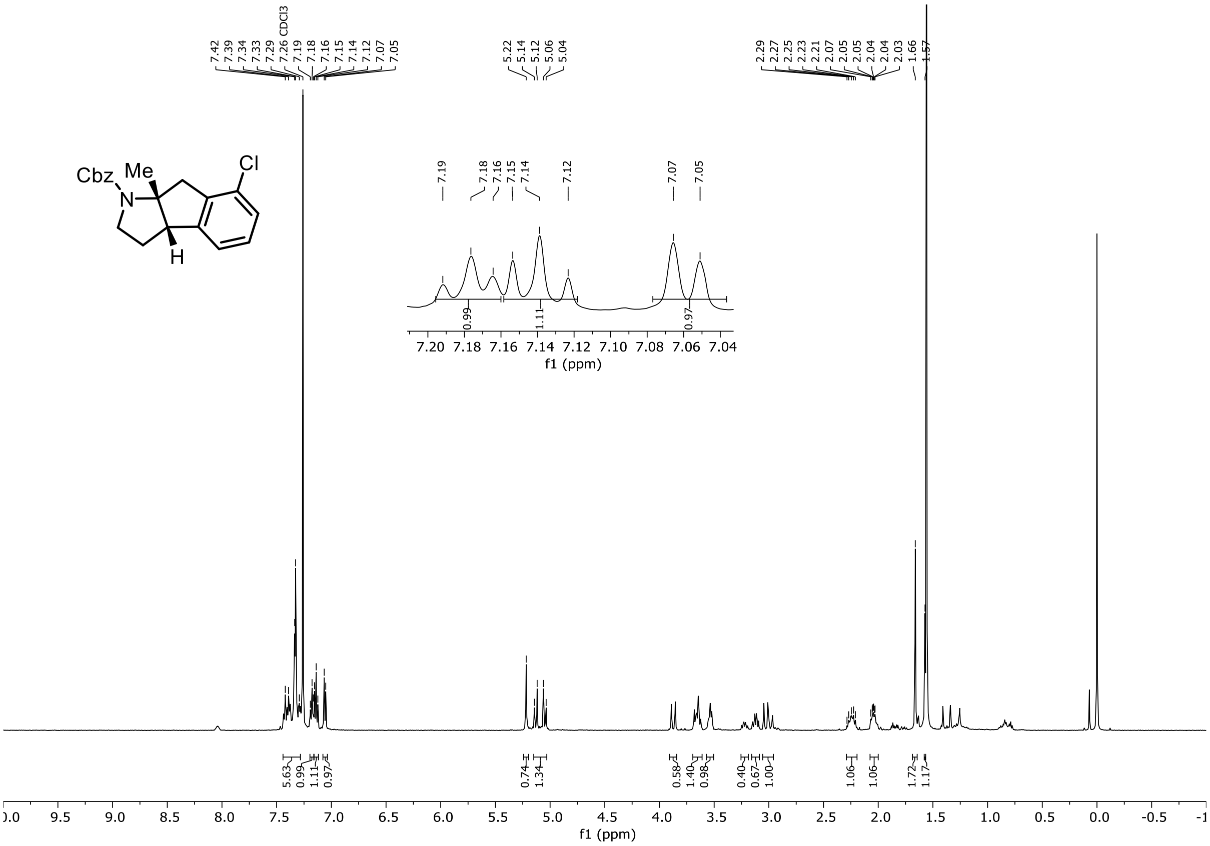

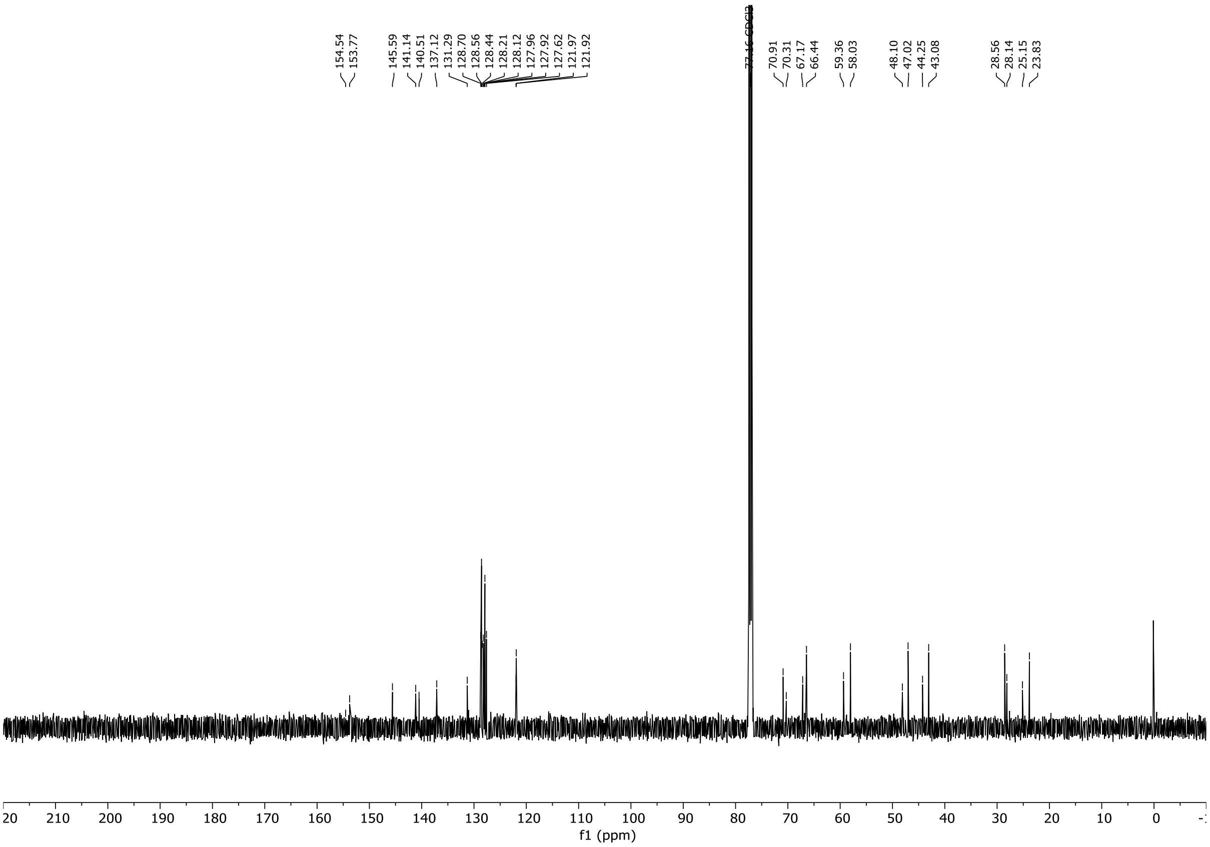

Ethyl (*E*)-3-(3,4-dimethoxyphenyl)-2-methylacrylate

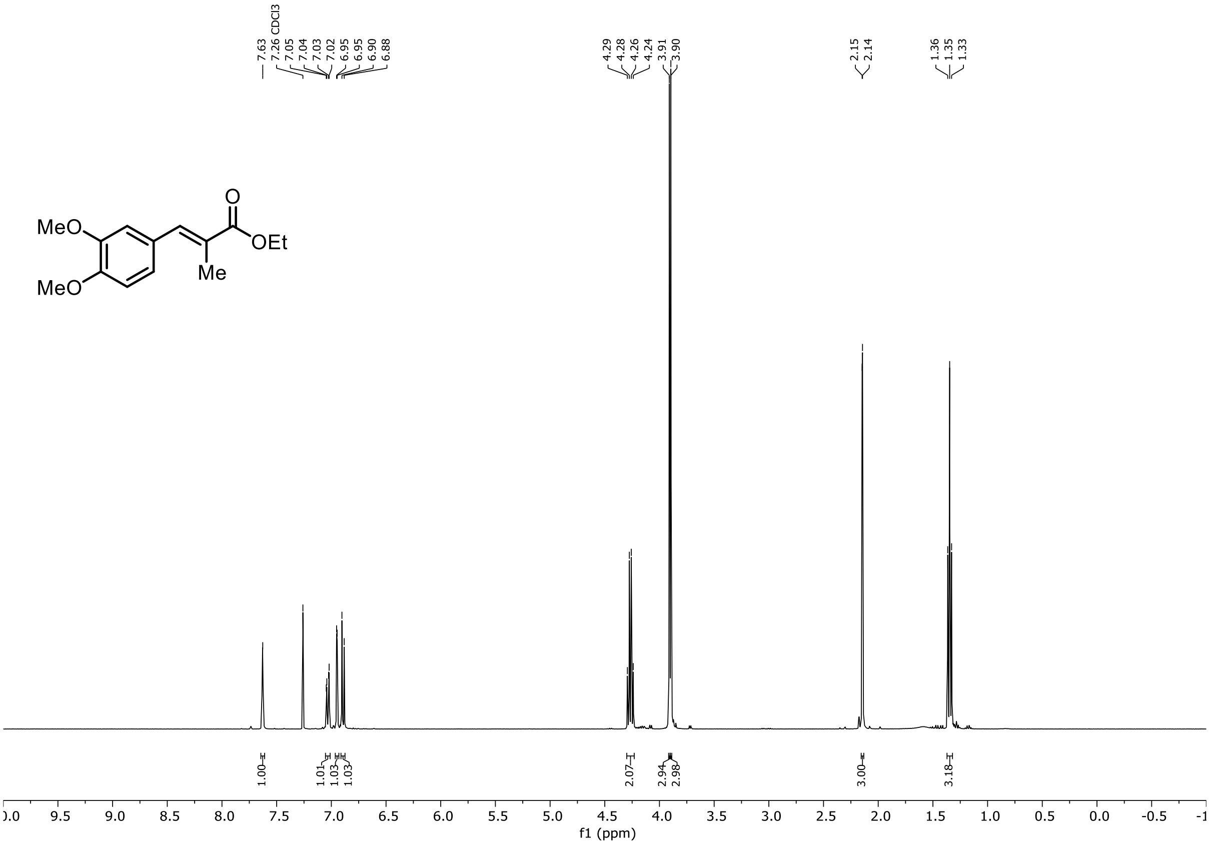

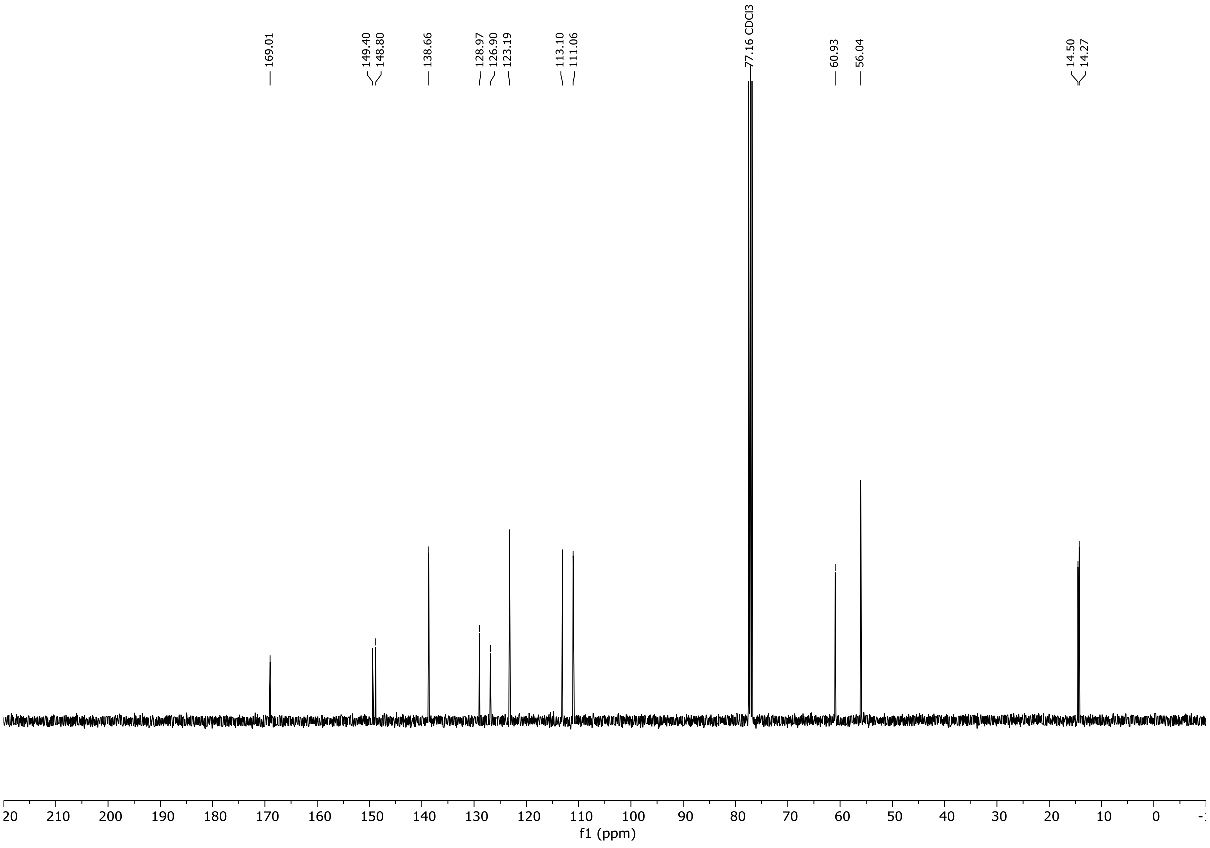

(*E*)-3-(3,4-Dimethoxyphenyl)-2-methylprop-2-en-1-ol

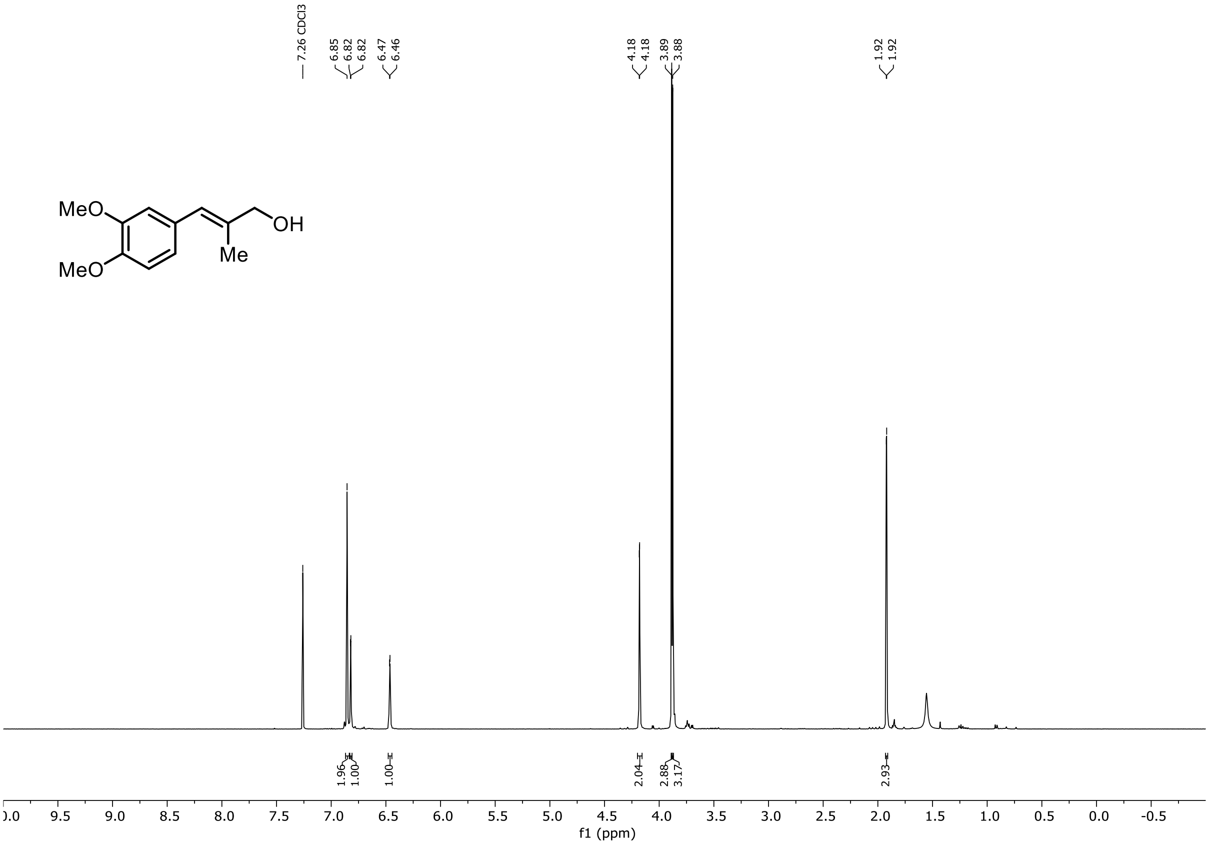

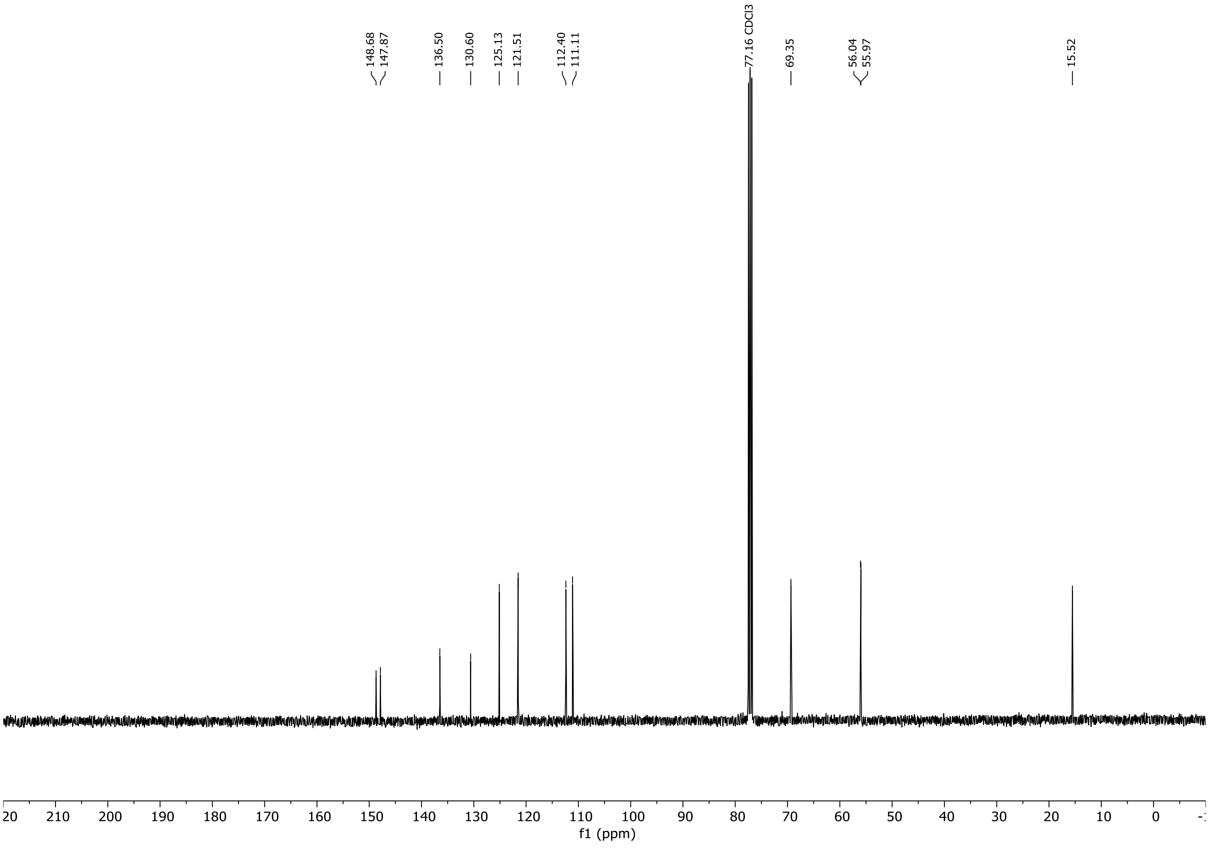

3-(3,4-Dimethoxyphenyl)-4-methylpent-4-enoic acid

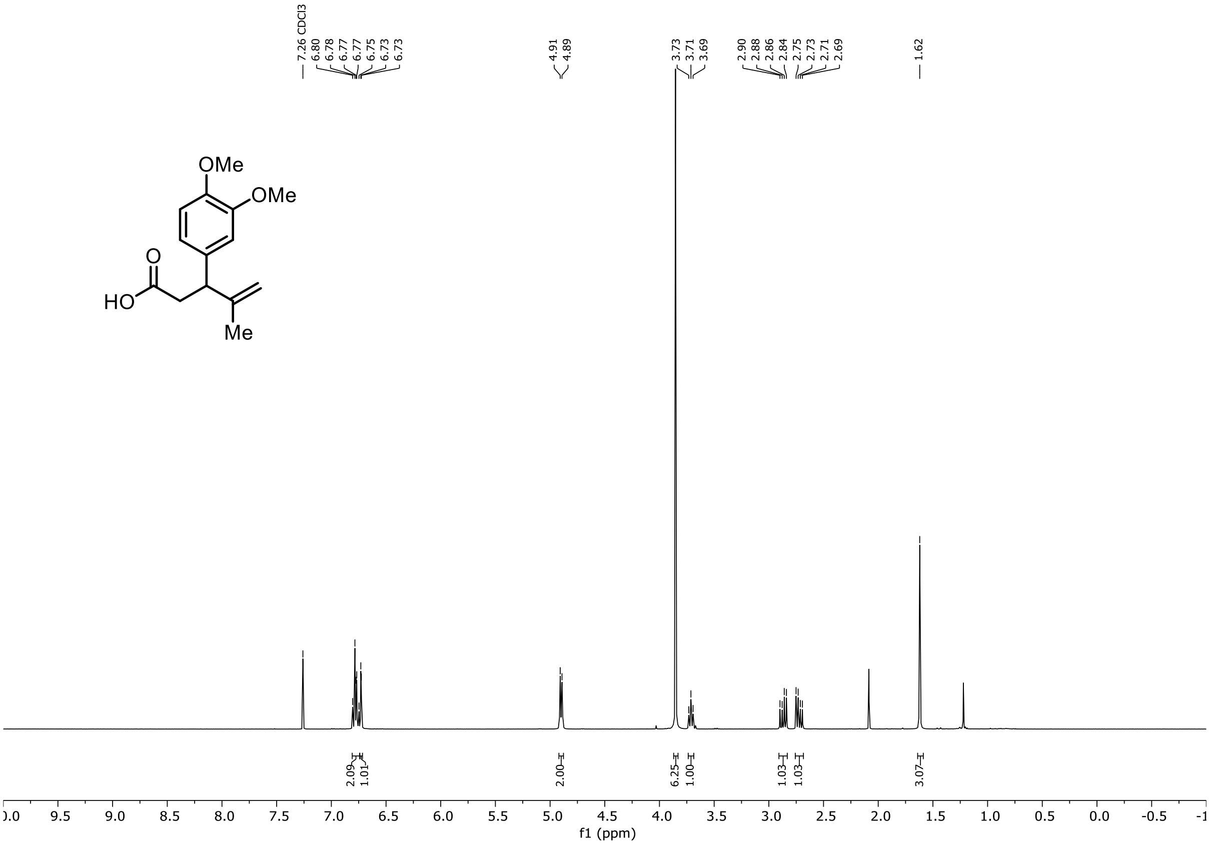

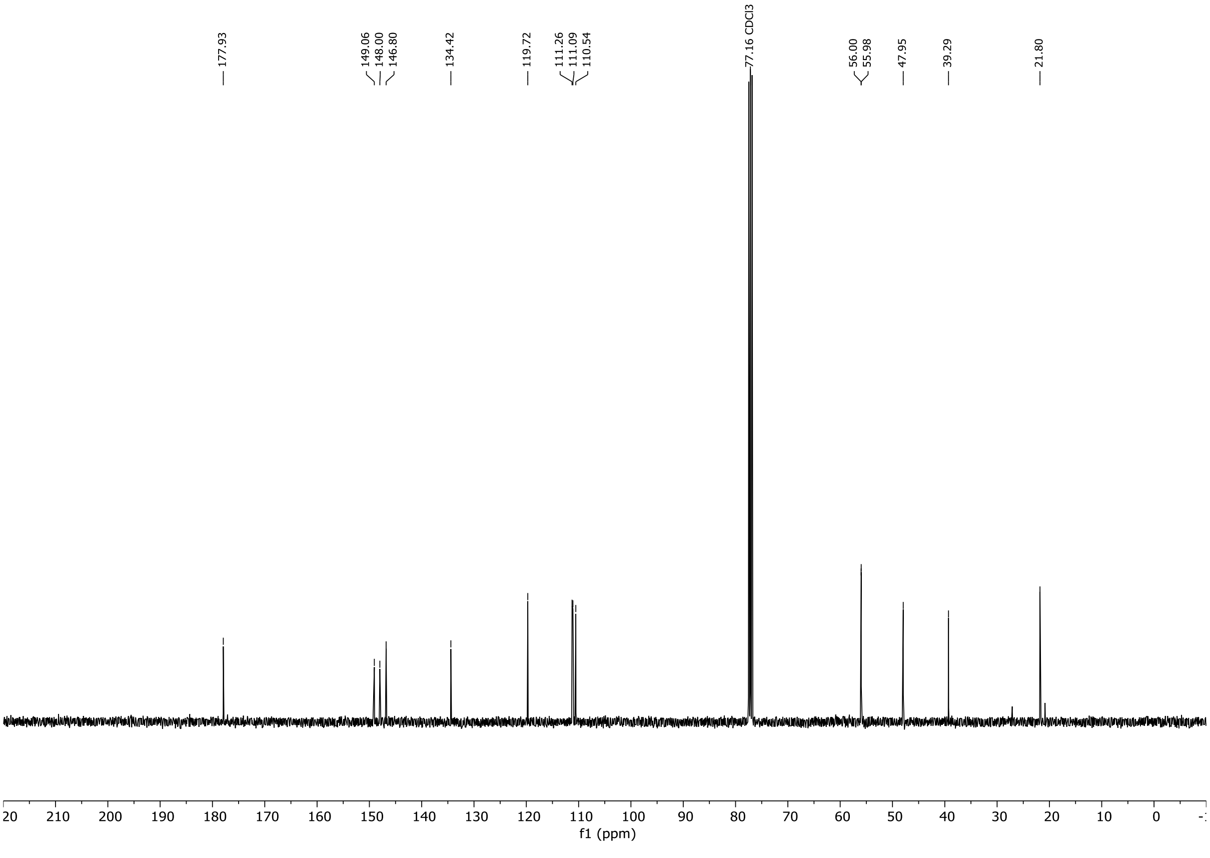

3-(3,4-Dimethoxyphenyl)-4-methylpent-4-en-1-ol

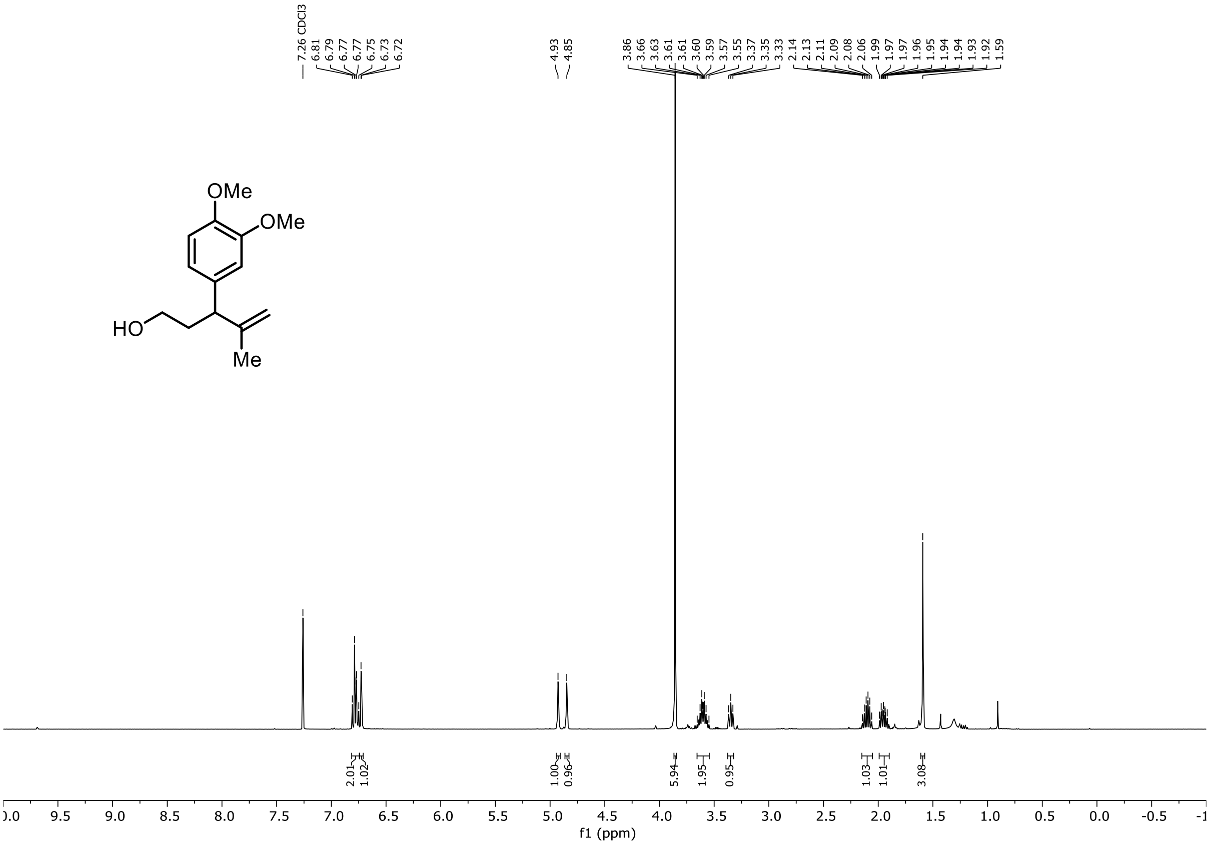

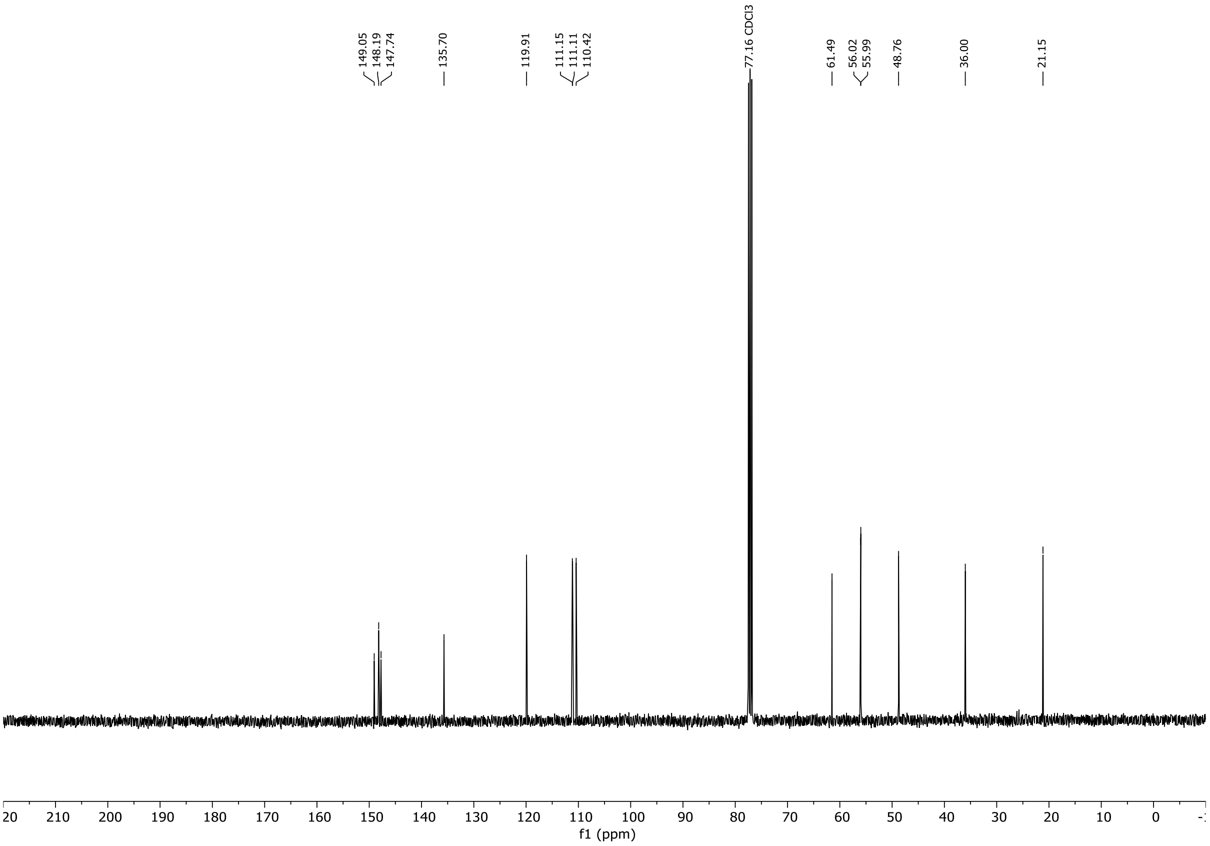

Benzyl (3-(3,4-dimethoxyphenyl)-4-methylpent-4-en-1-yl)((perfluorobenzoyl)oxy)carbamate (1k)

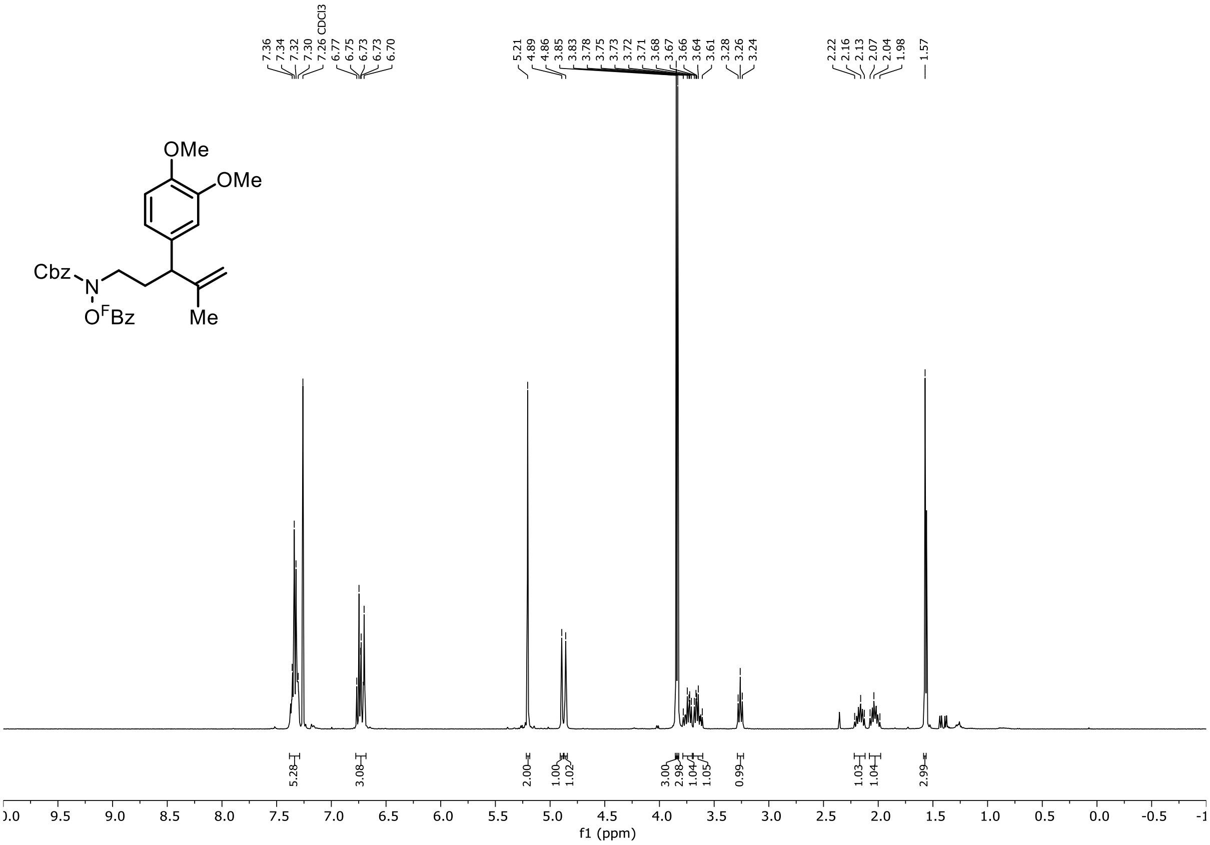

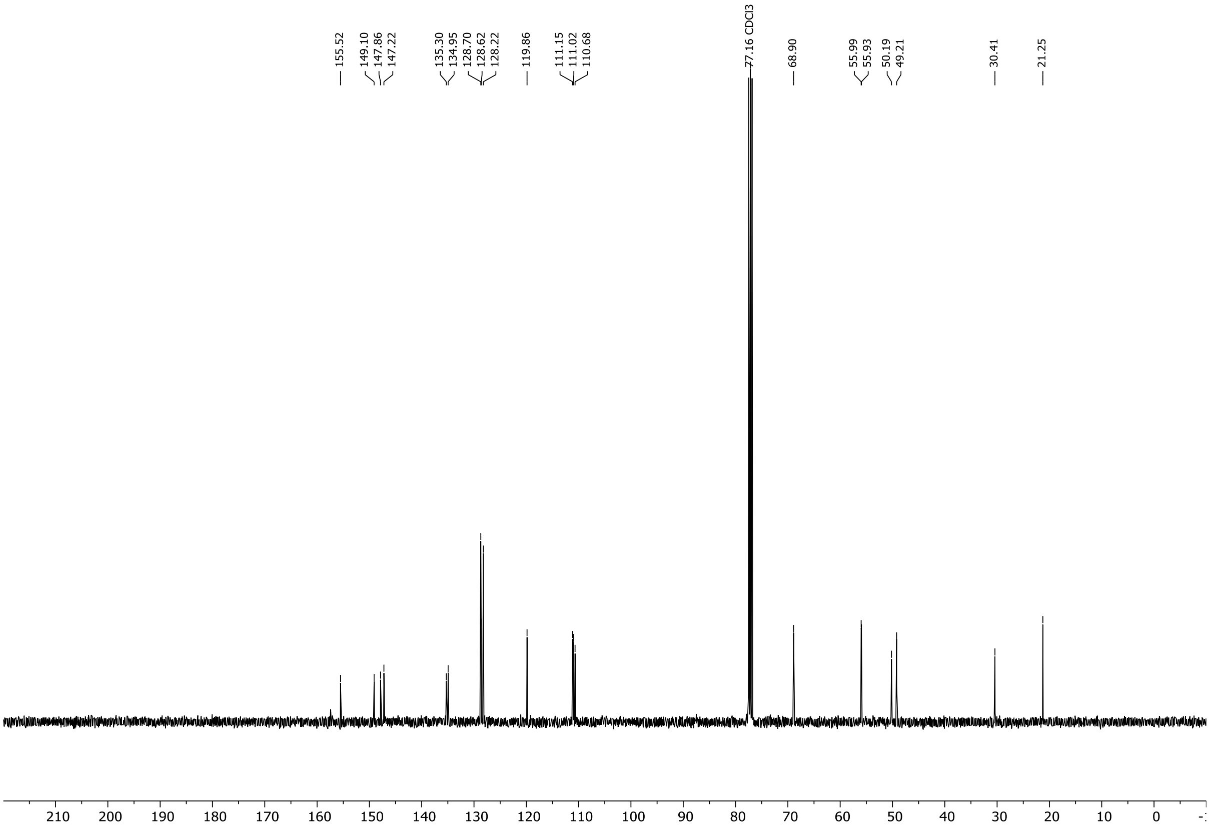

Benzyl 5,6-dimethoxy-8a-methyl-3,3a,8,8a-tetrahydroindeno[2,1-*b*]pyrrole-1(2*H*)-carboxylate (2k)

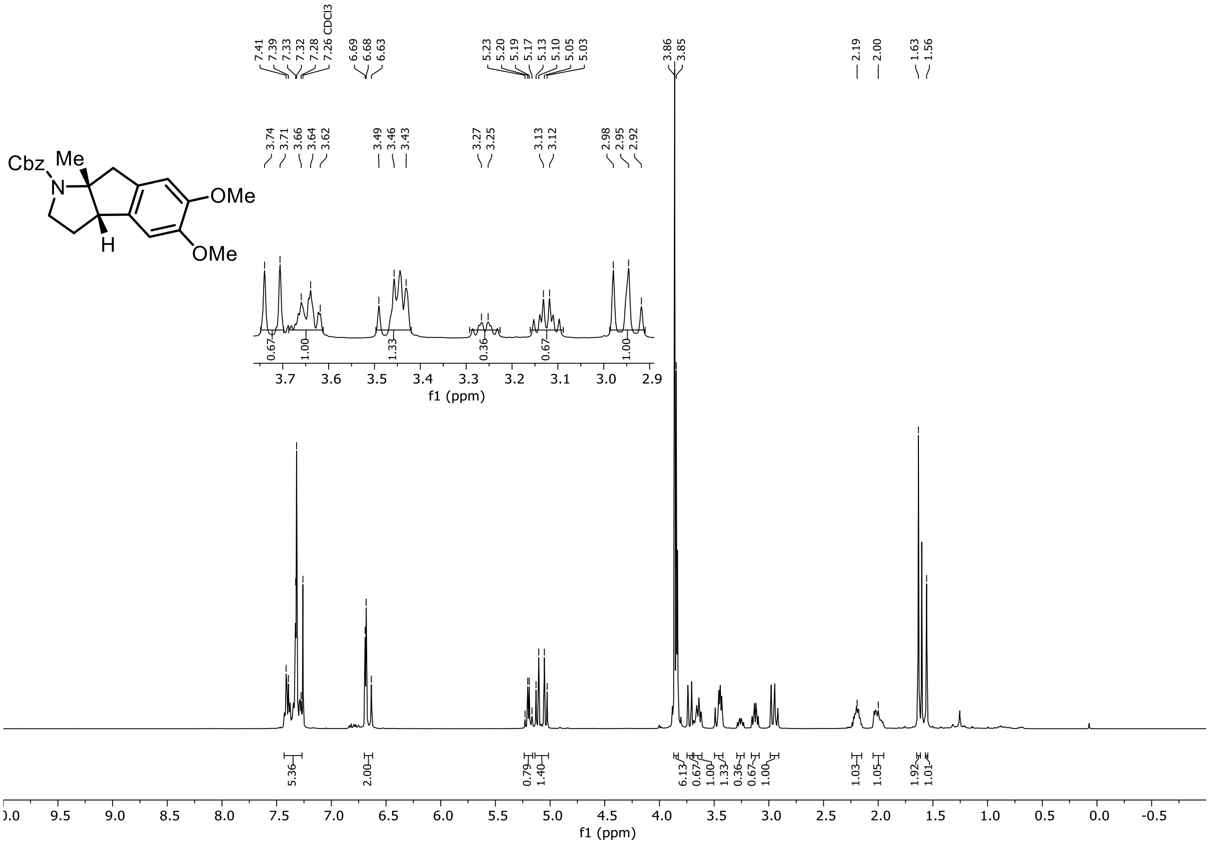

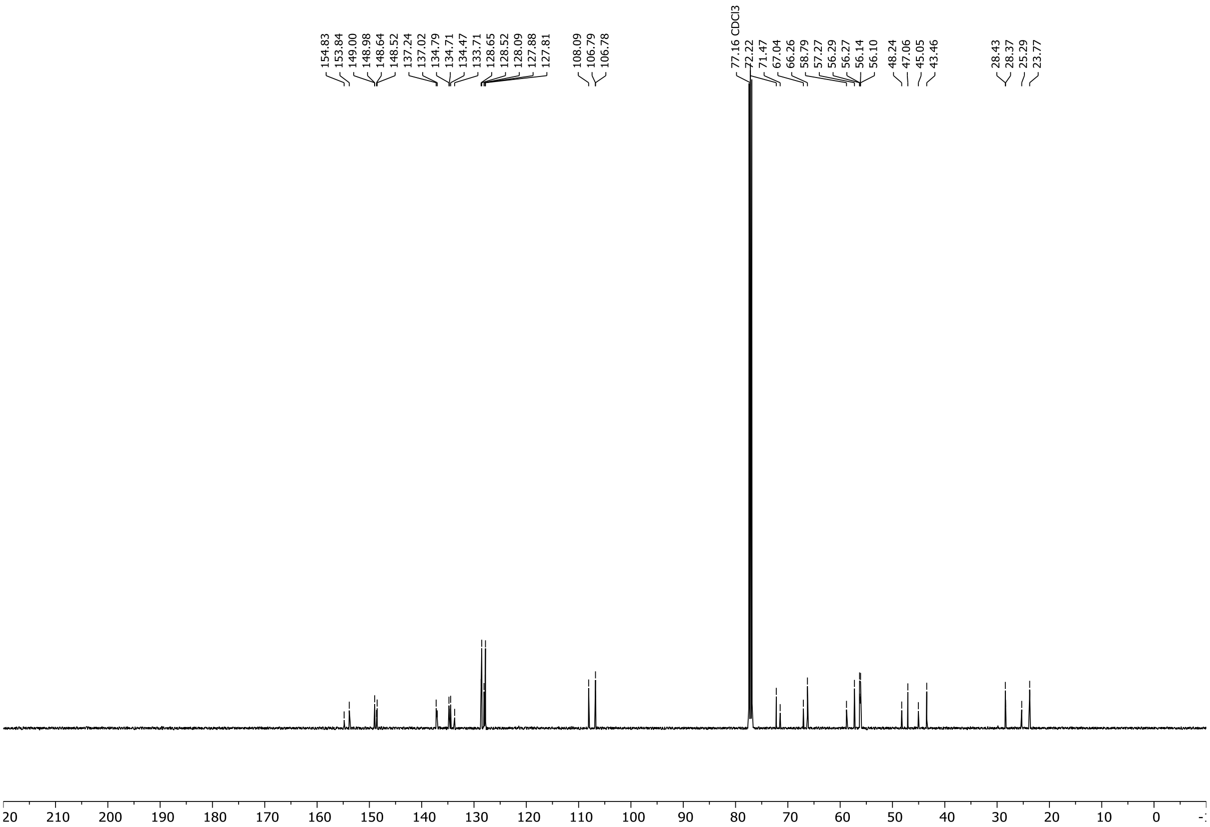

Benzyl 6,7-dimethoxy-8a-methyl-3,3a,8,8a-tetrahydroindeno[2,1-*b*]pyrrole-1(2*H*)-carboxylate (2k’)

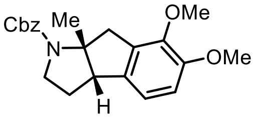

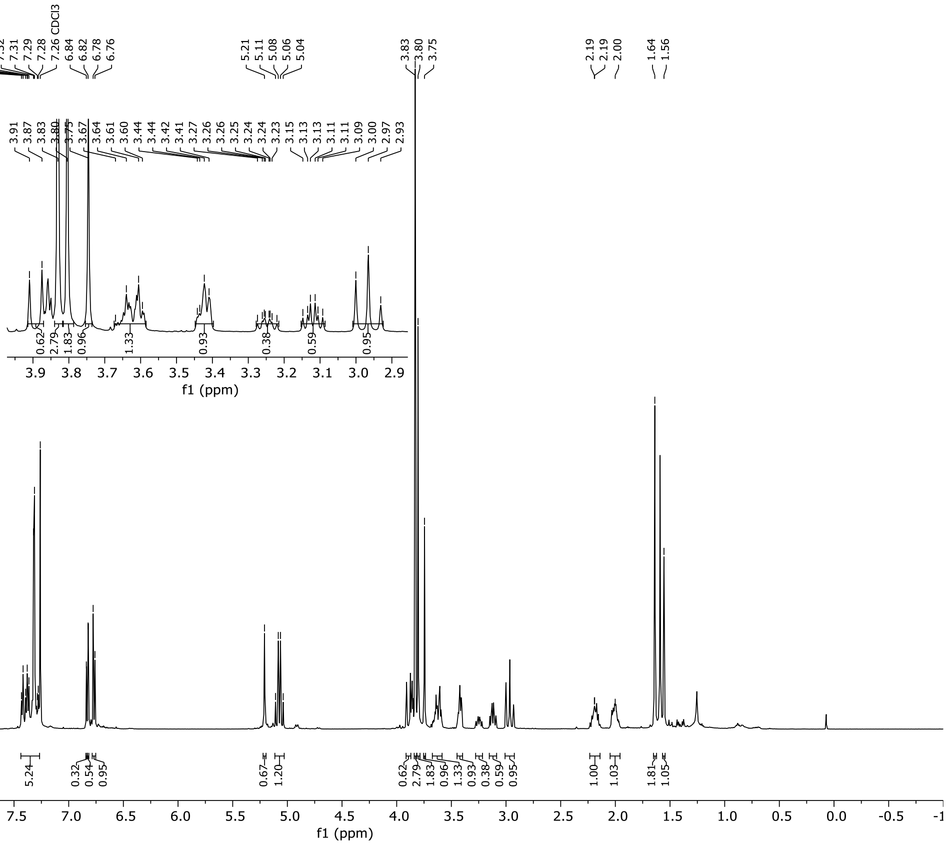

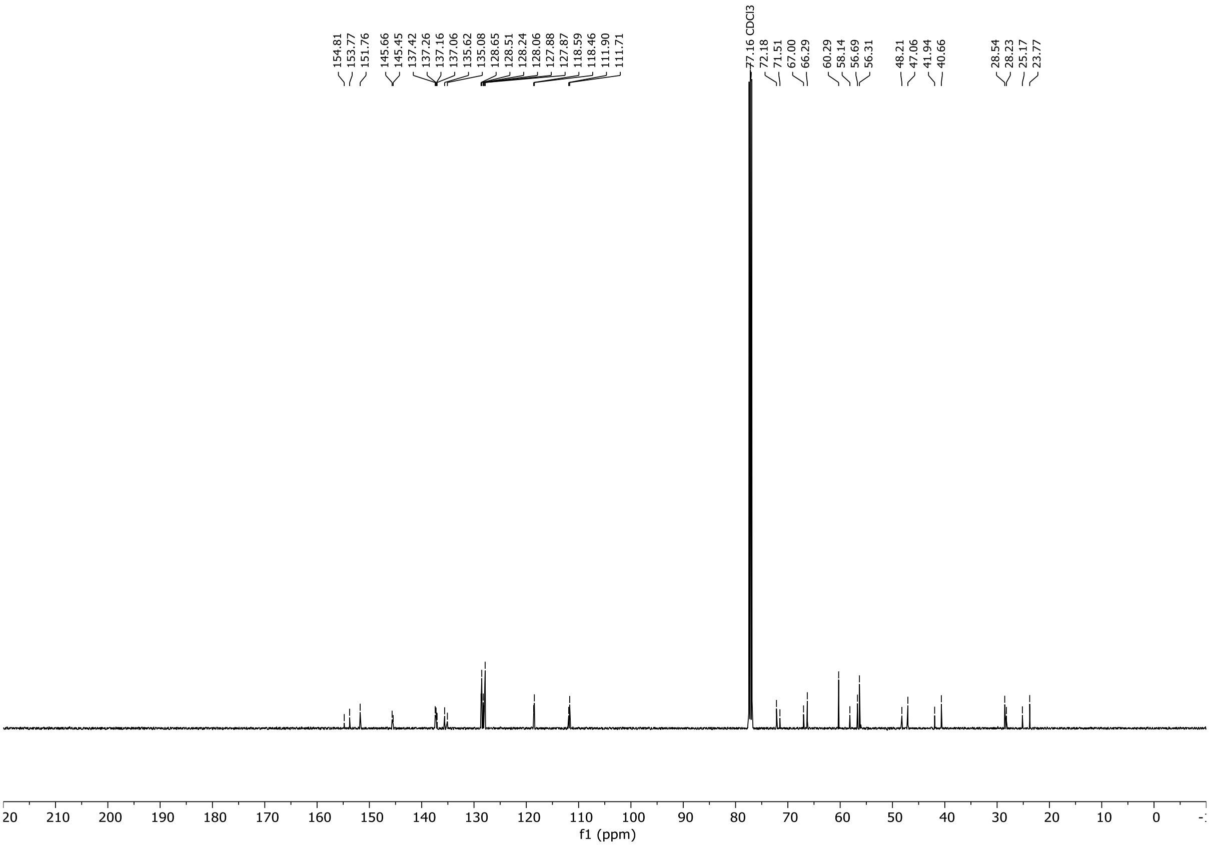

5,6-Dimethoxy-8a-methyl-1,2,3,3a,8,8a-hexahydroindeno[2,1-*b*]pyrrole

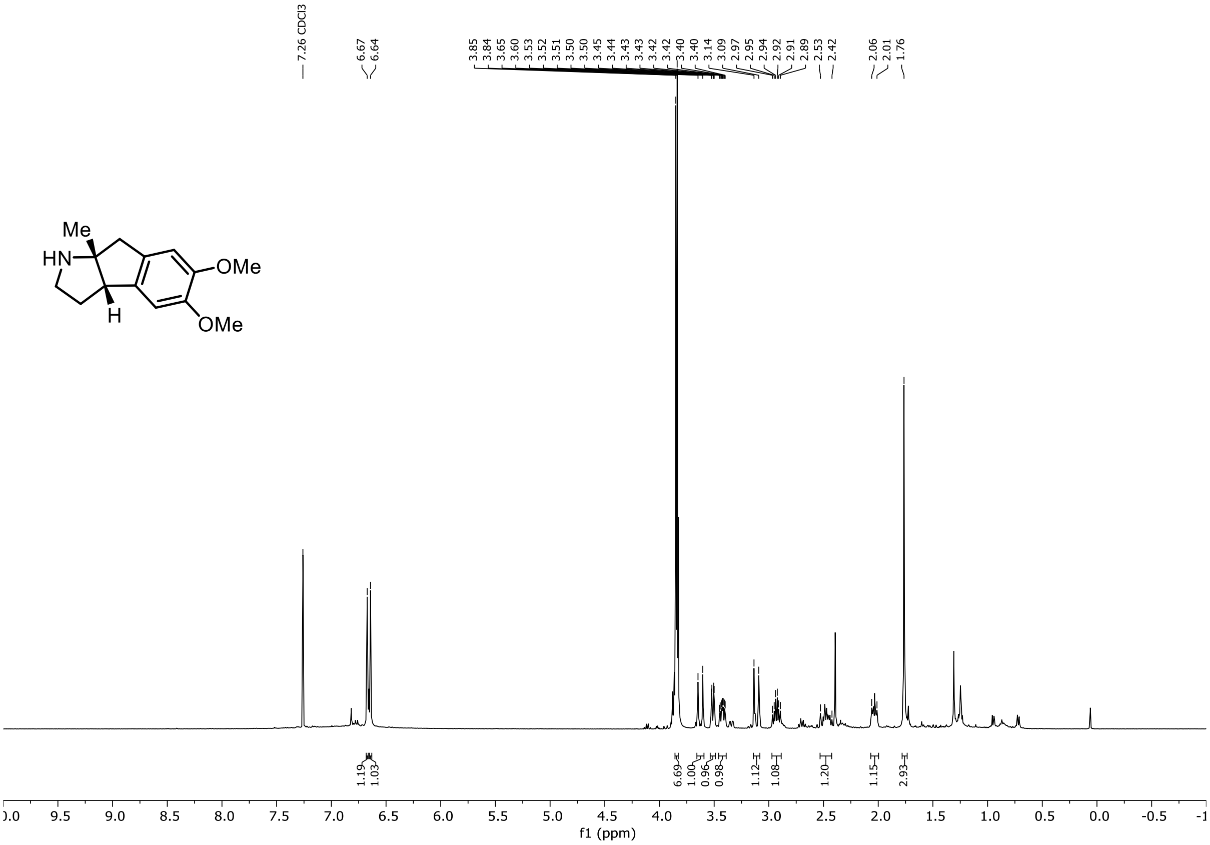

(*E*)-3-(Furan-3-yl)-2-methylacrylaldehyde

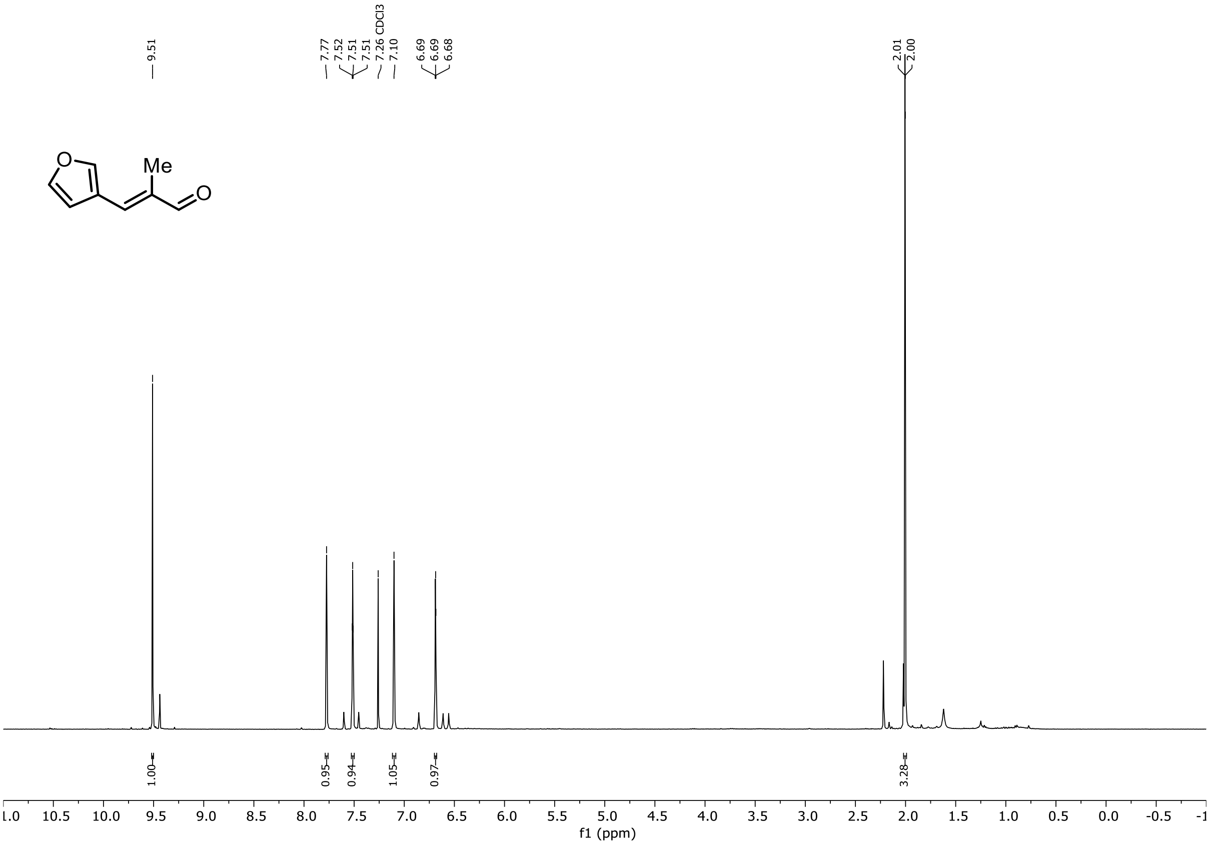

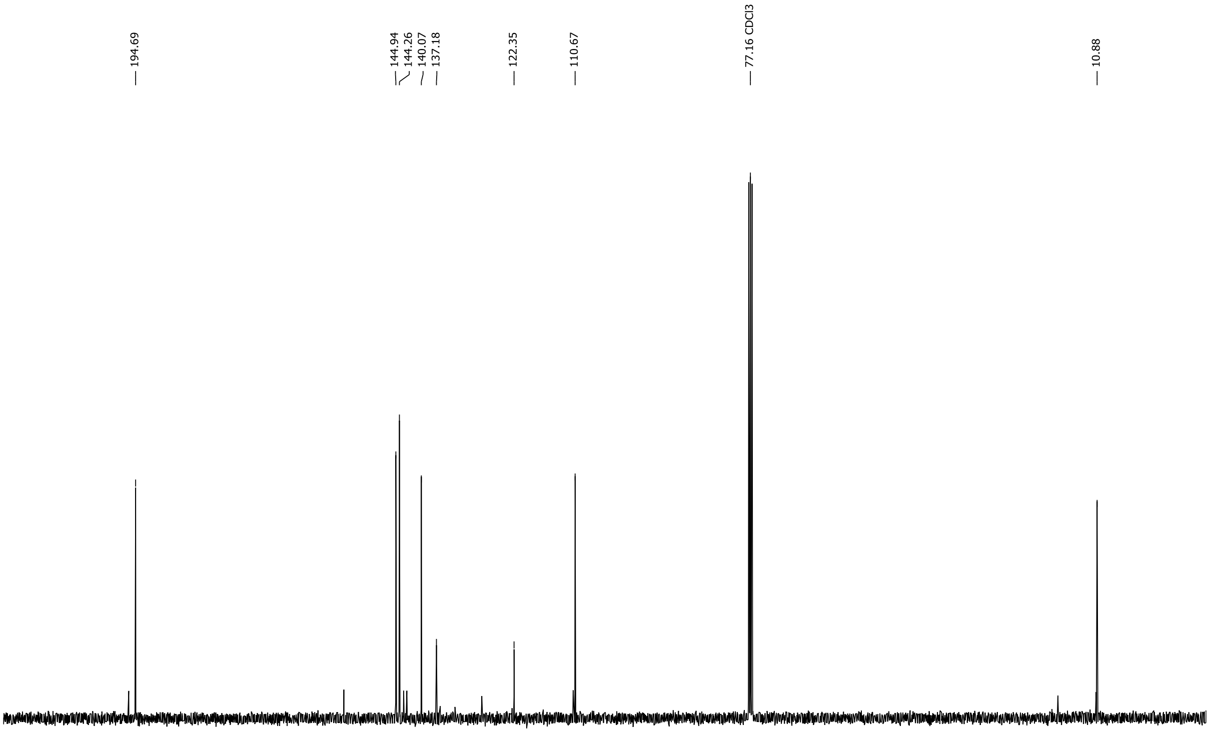

(*E*)-3-(Furan-3-yl)-2-methylprop-2-en-1-ol

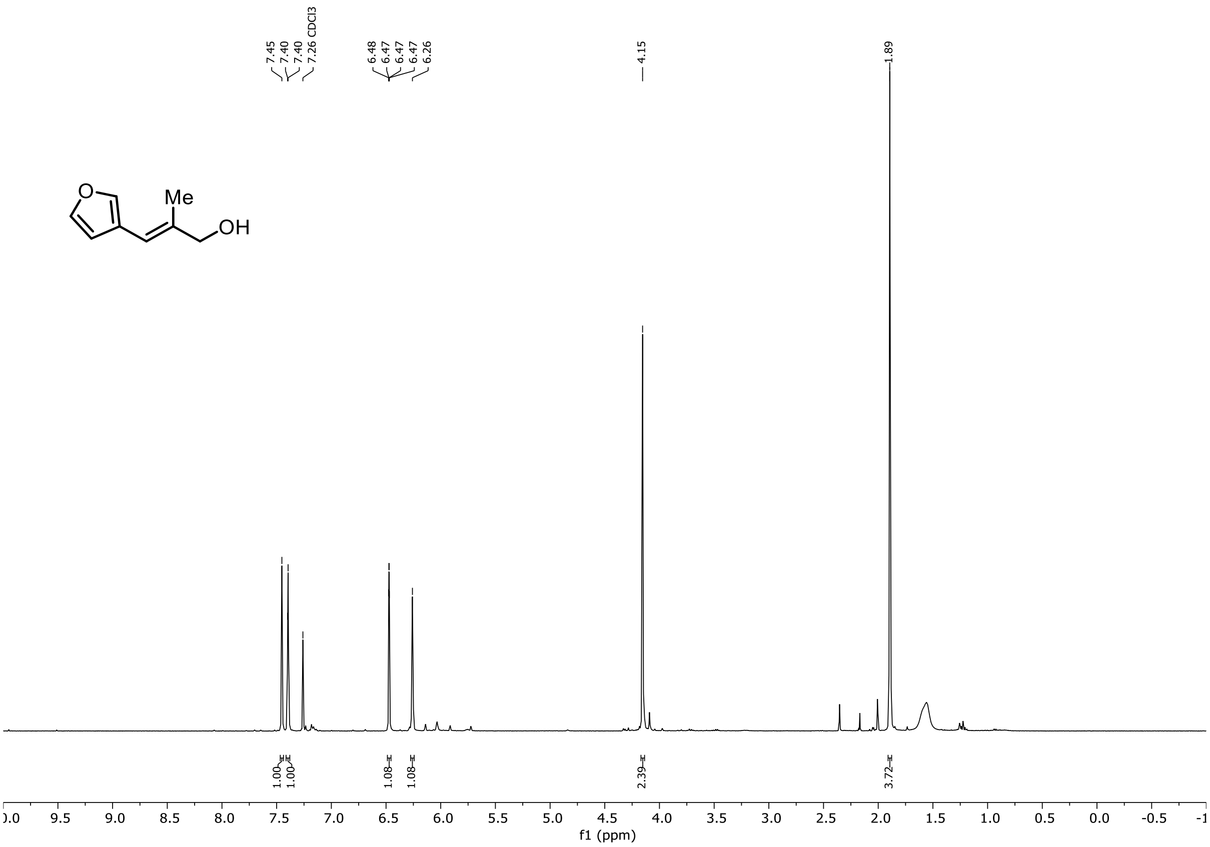

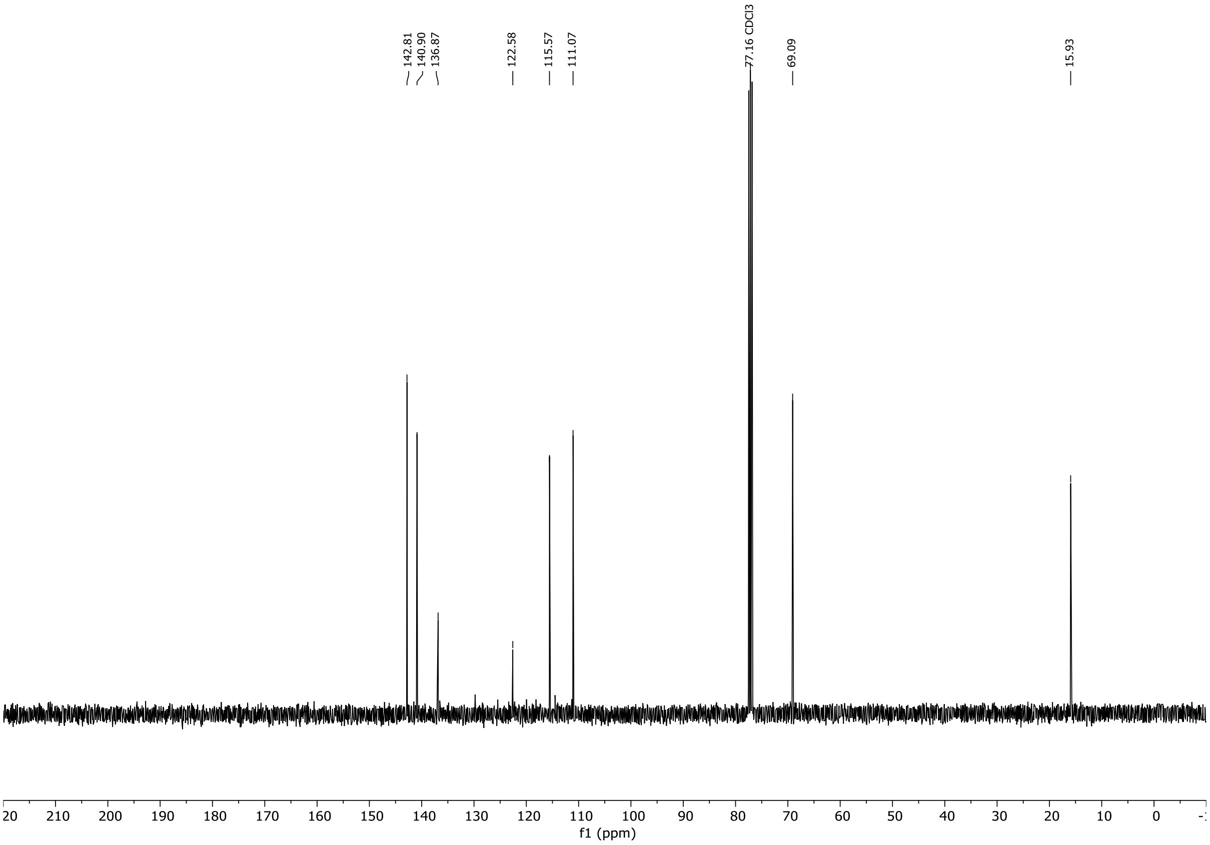

3-(Furan-3-yl)-4-methylpent-4-enoic acid

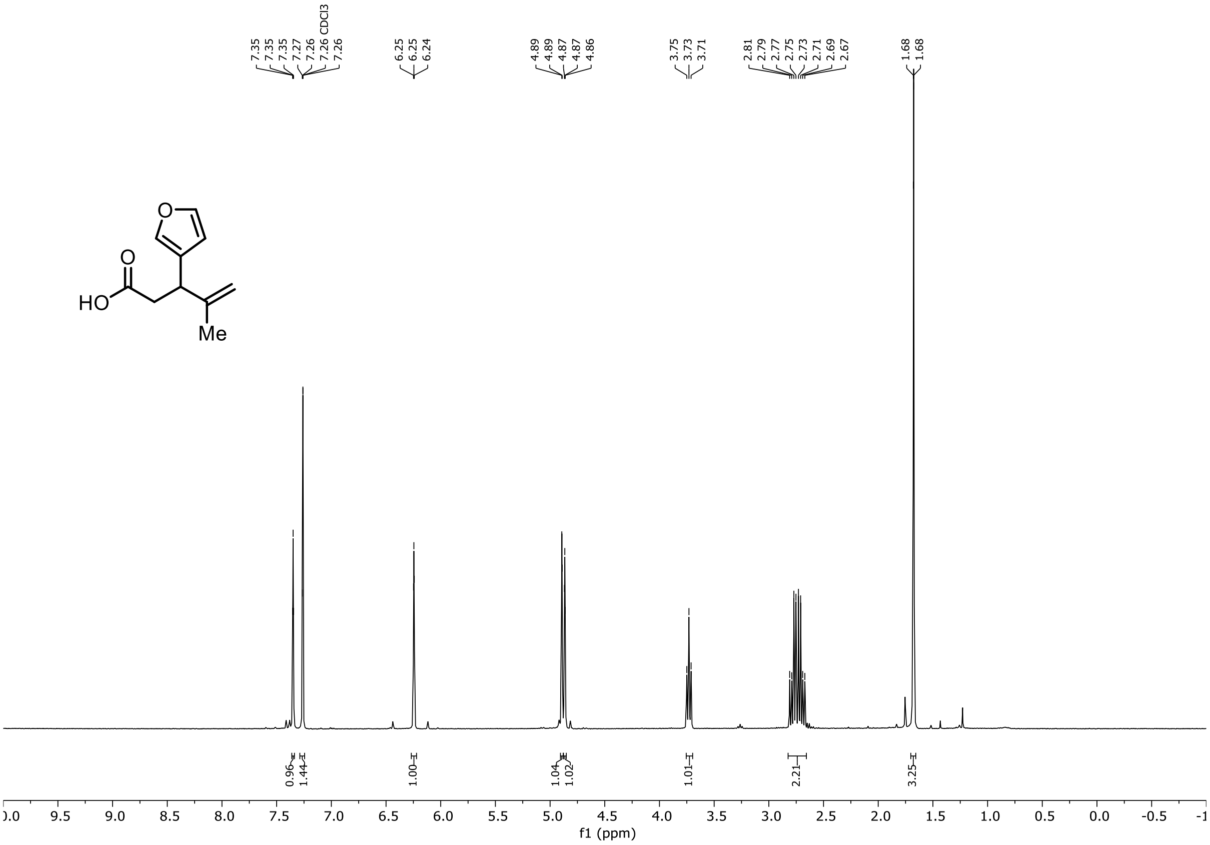

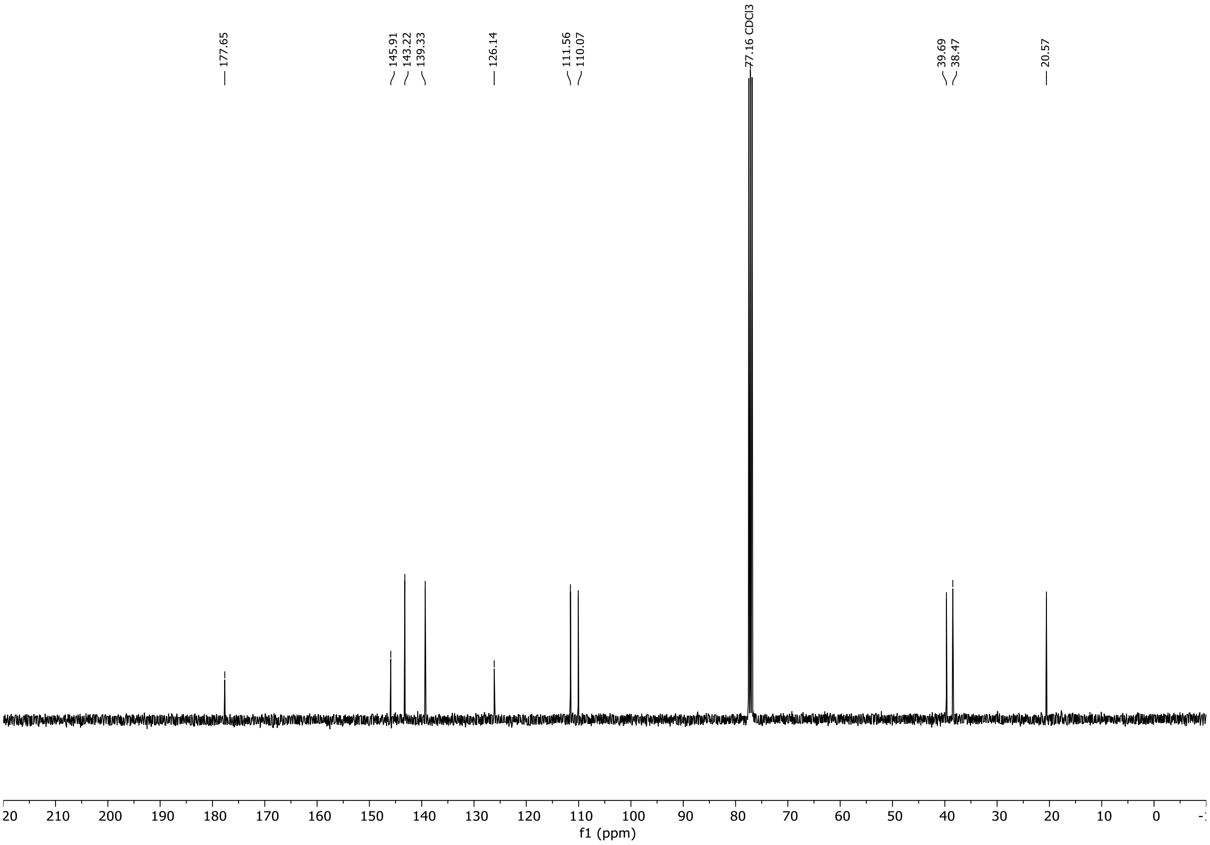

3-(Furan-3-yl)-4-methylpent-4-en-1-ol

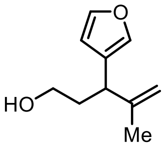

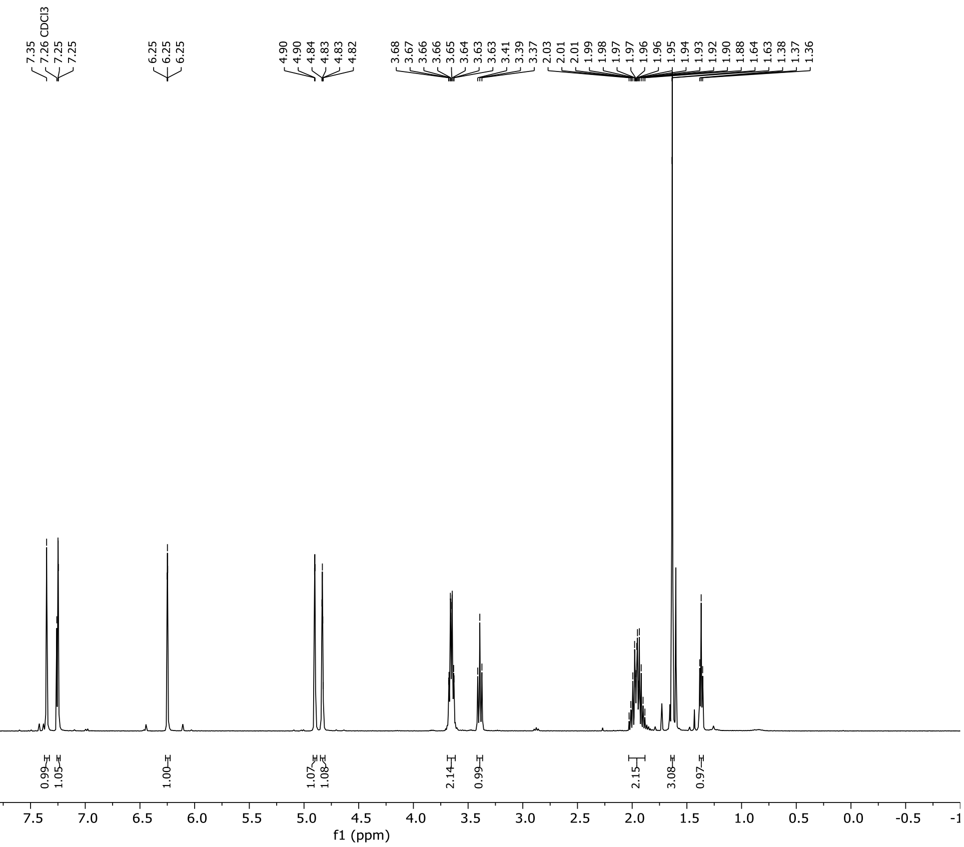

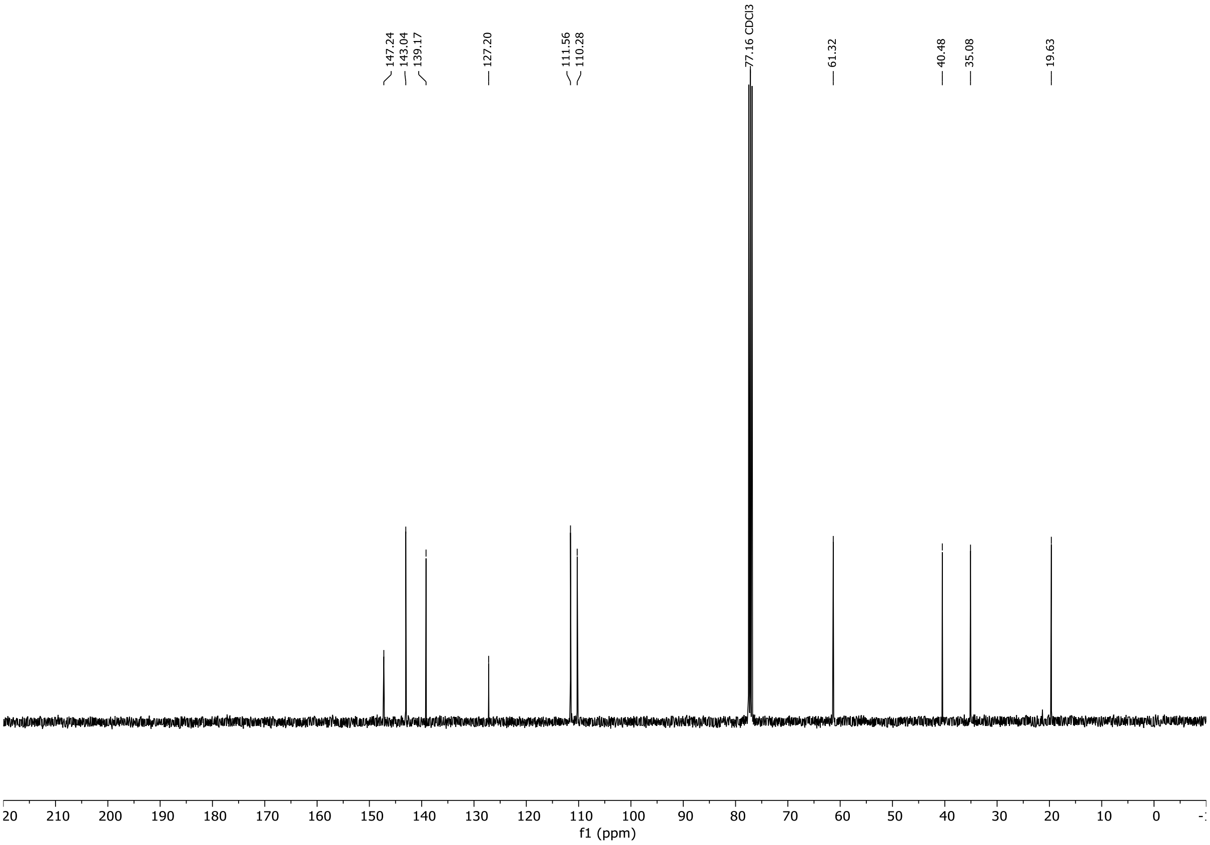

Benzyl (3-(furan-3-yl)-4-methylpent-4-en-1-yl)((perfluorobenzoyl)oxy)carbamate (1l)

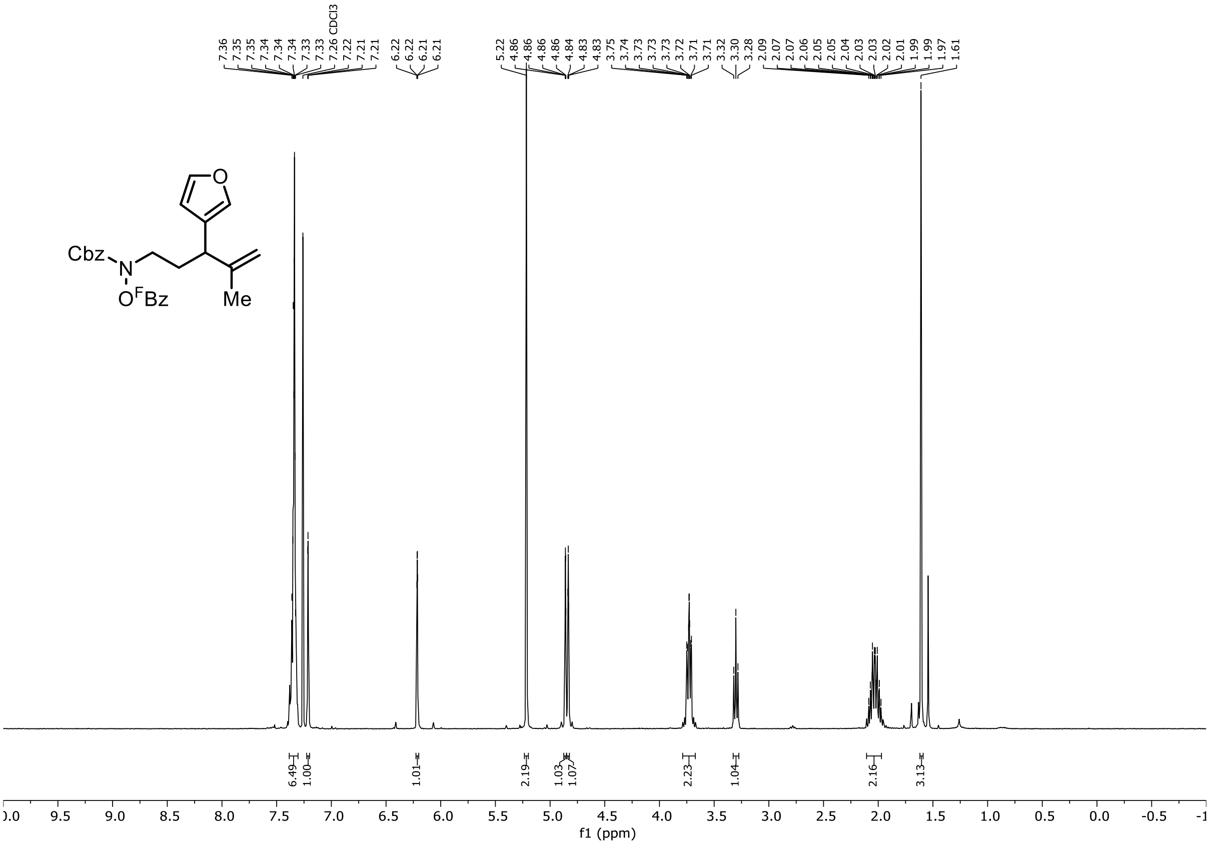

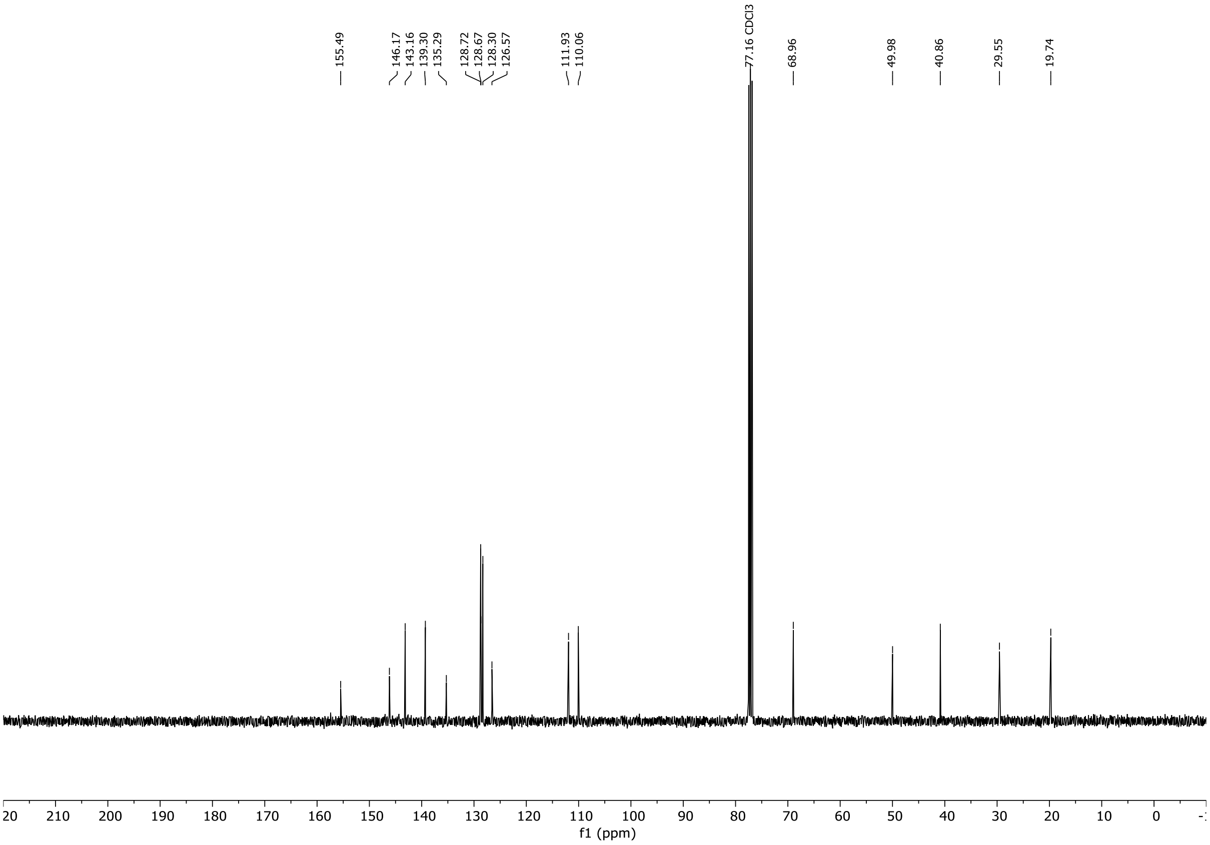

Benzyl 6a-methyl-4,5,6a,7-tetrahydrofuro[3',2':3,4]cyclopenta[1,2-*b*]pyrrole-6(3b*H*)-carboxylate (2l)

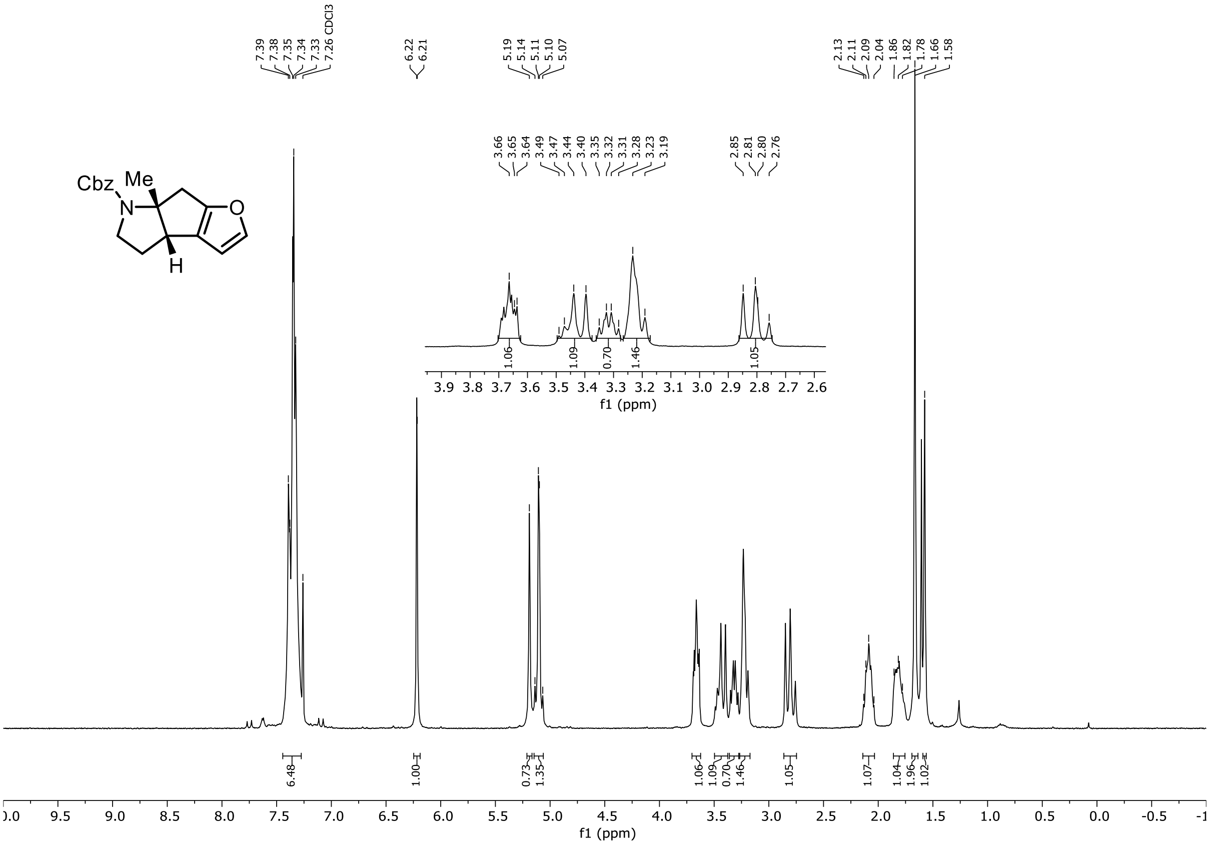

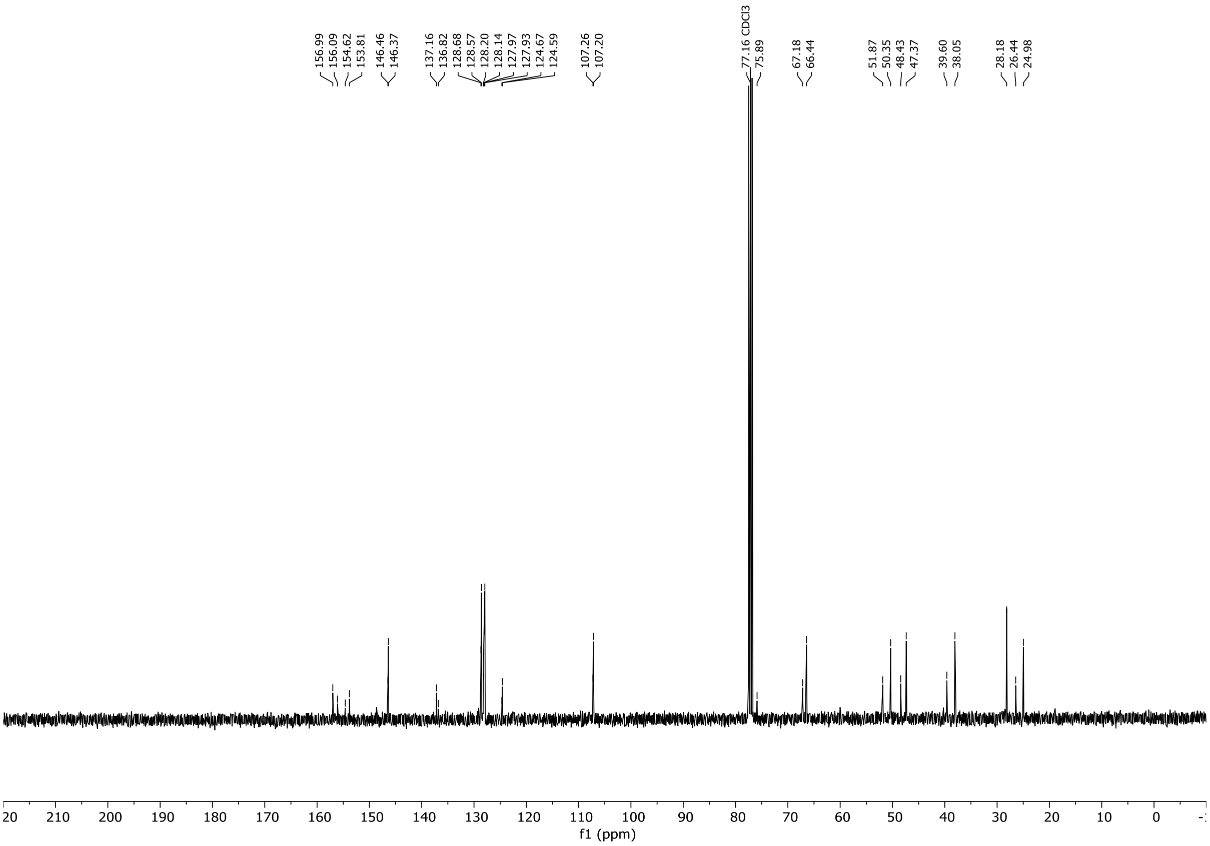

Ethyl 4-methyl-3-(pyridin-3-yl)pent-4-enoate

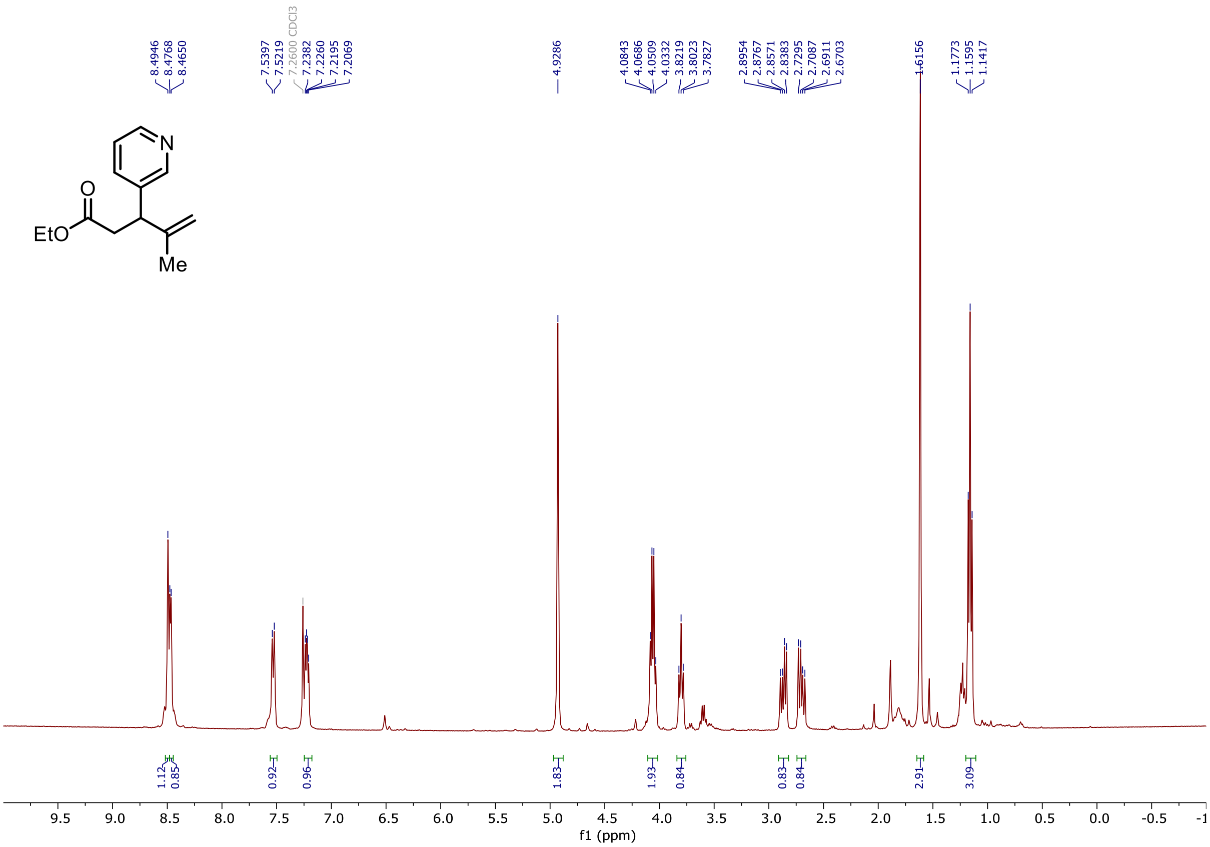

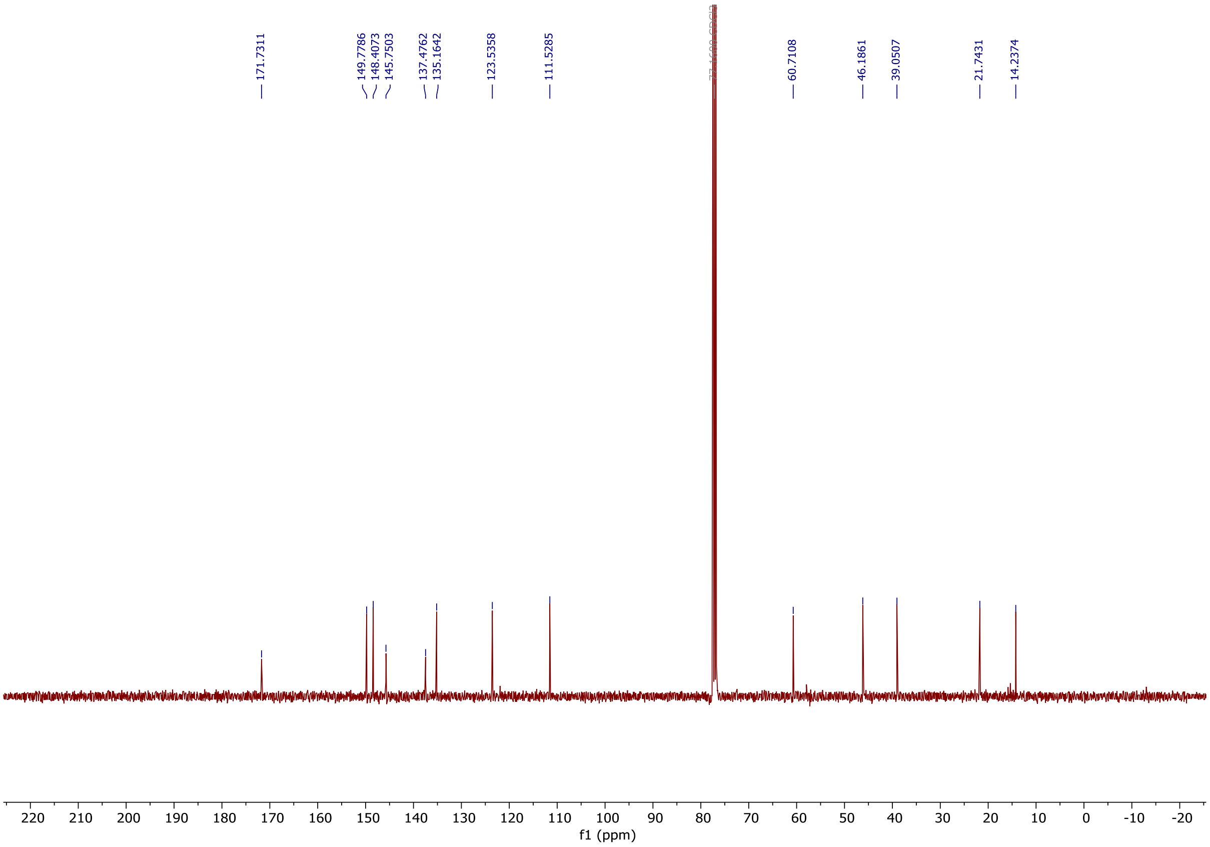

4-Methyl-3-(pyridin-3-yl)pent-4-en-1-ol

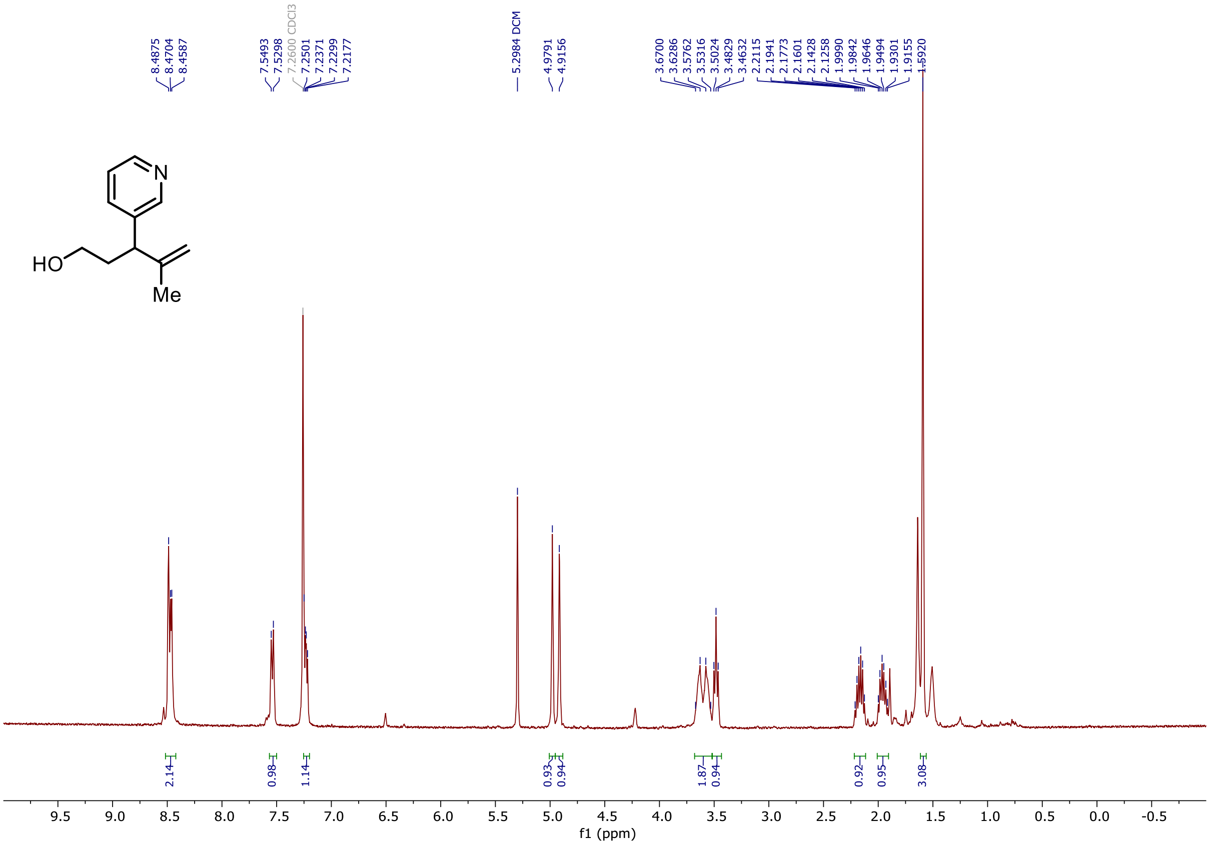

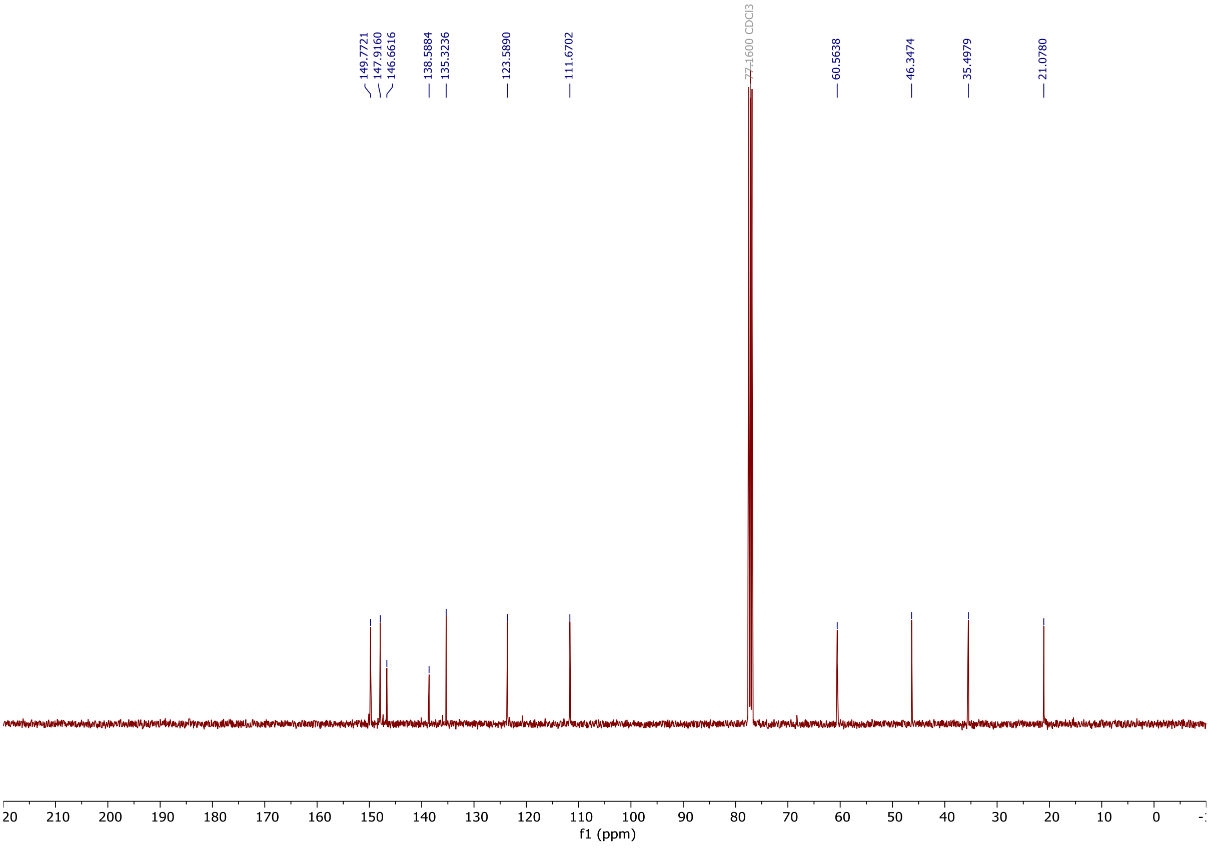

Benzyl (4-methyl-3-(pyridin-3-yl)pent-4-en-1-yl)((perfluorobenzoyl)oxy)carbamate (1m)

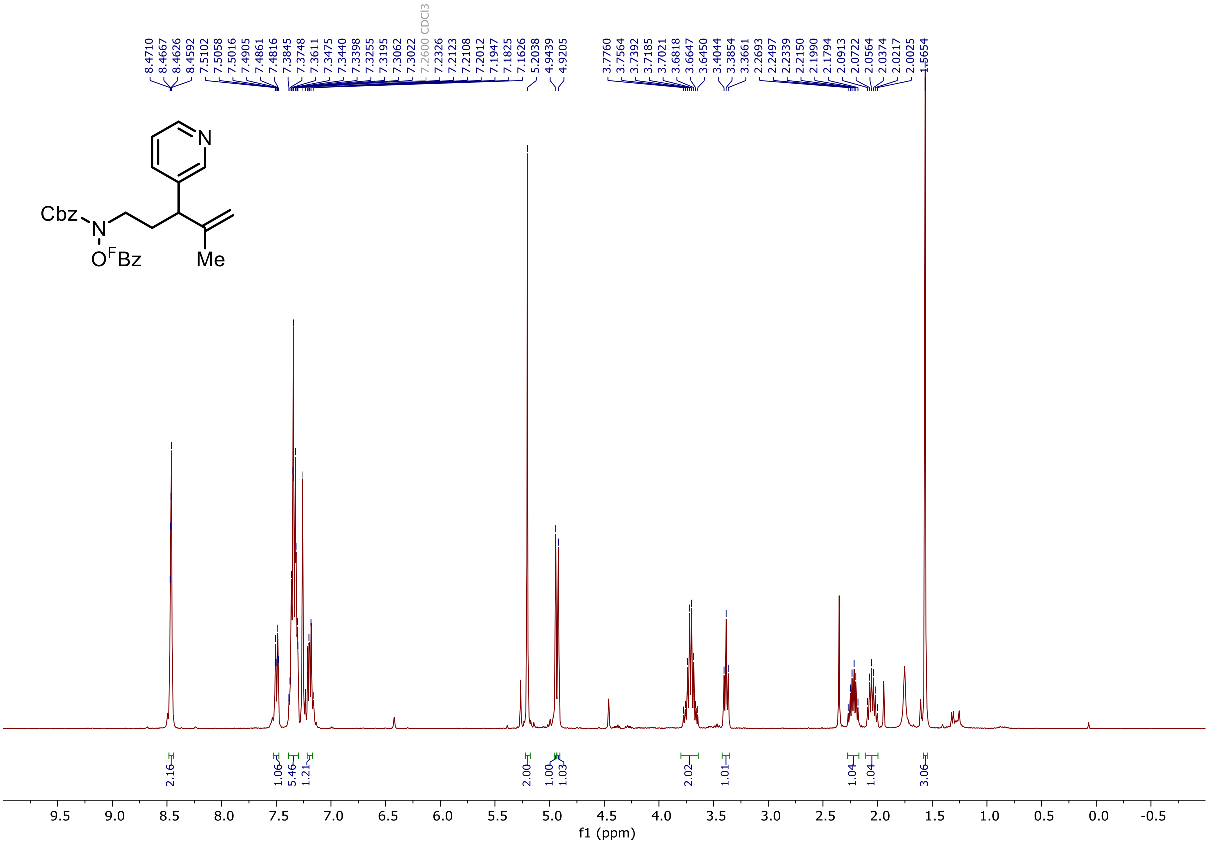

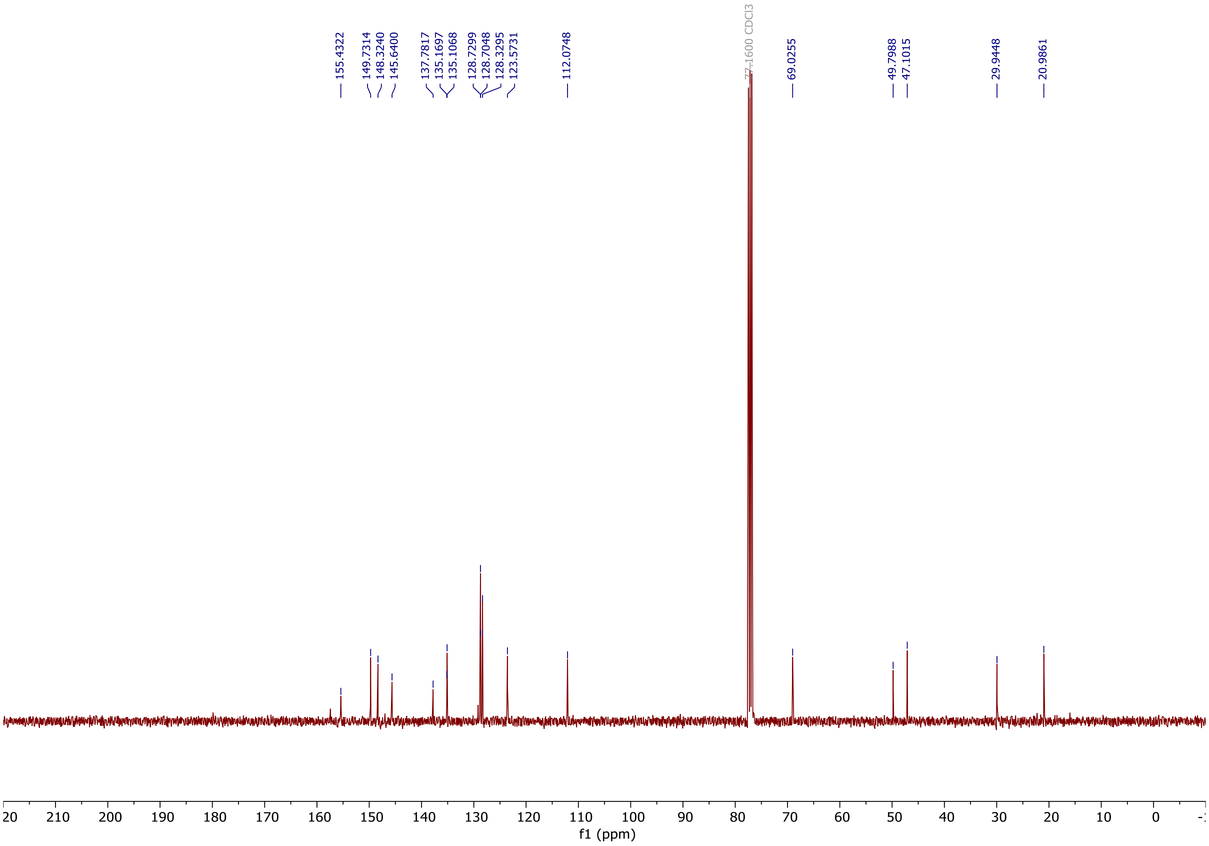

Benzyl 8a-methyl-3,3a,8,8a-tetrahydropyrrolo[3',2':3,4]cyclopenta[1,2-c]pyridine-1(2*H*)-carboxylate (2m)

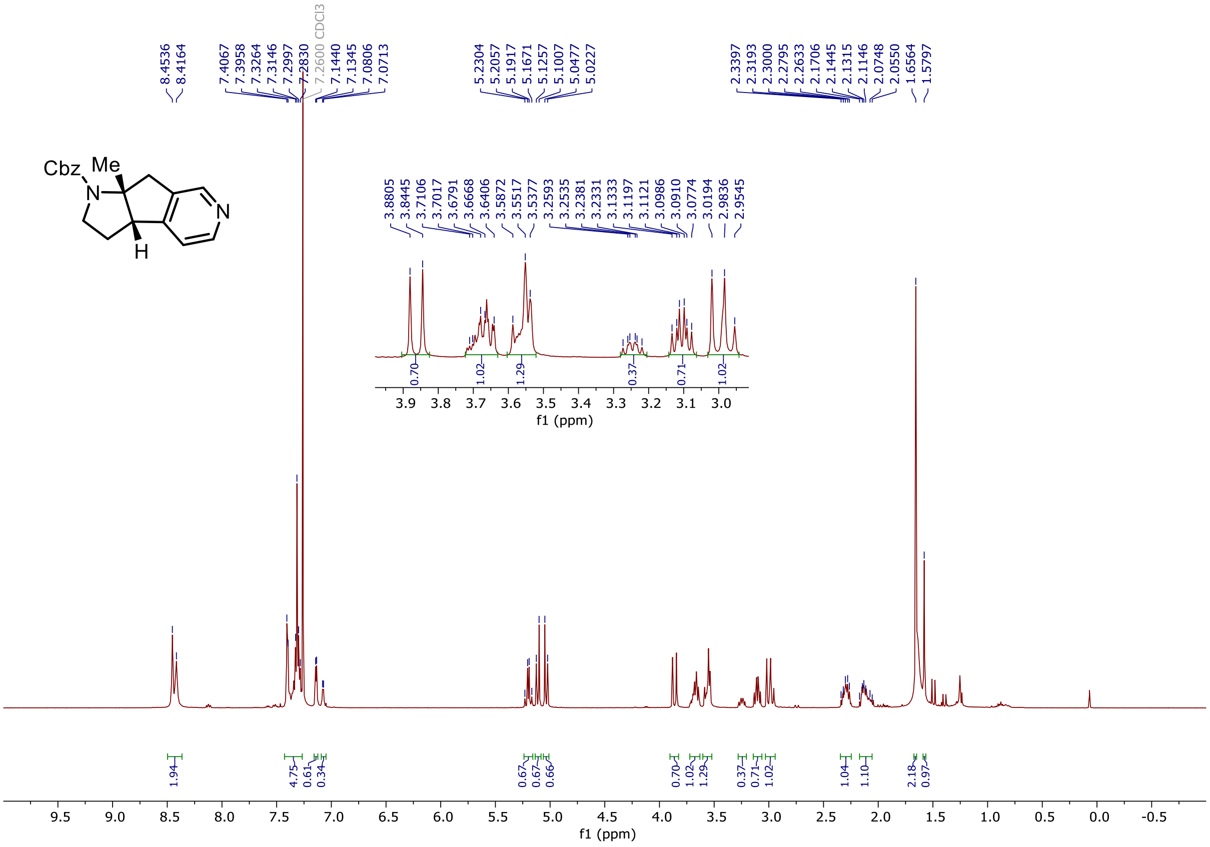

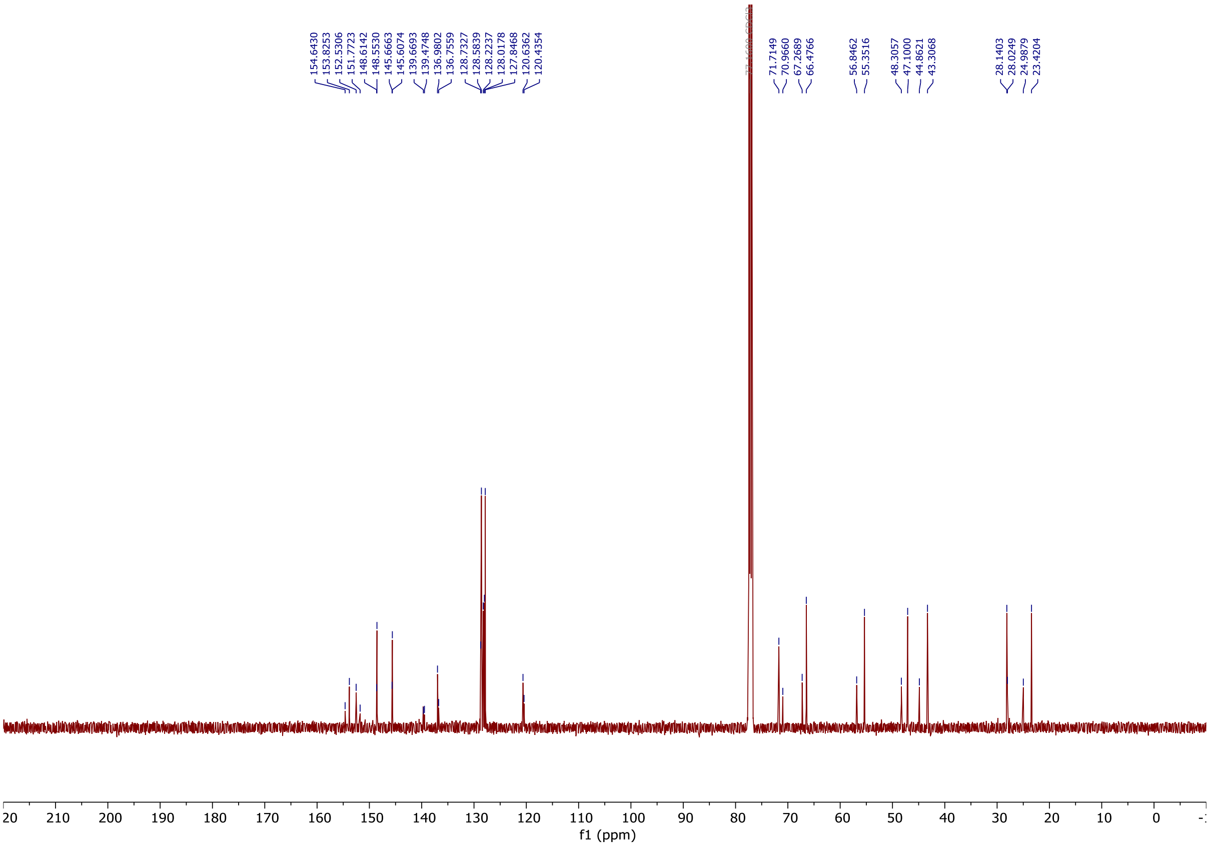

Ethyl (*E*)-2-methyl-3-(1-tosyl-1*H*-indol-3-yl)acrylate

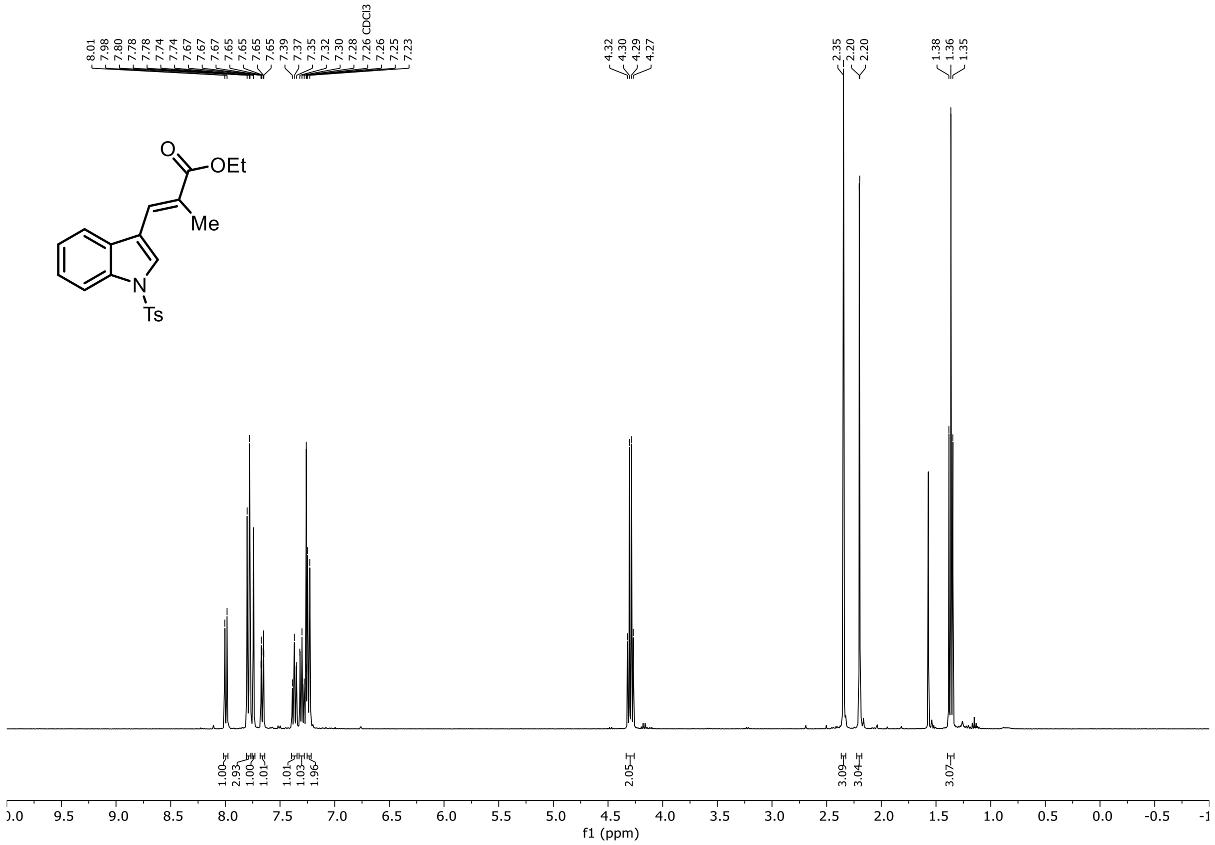

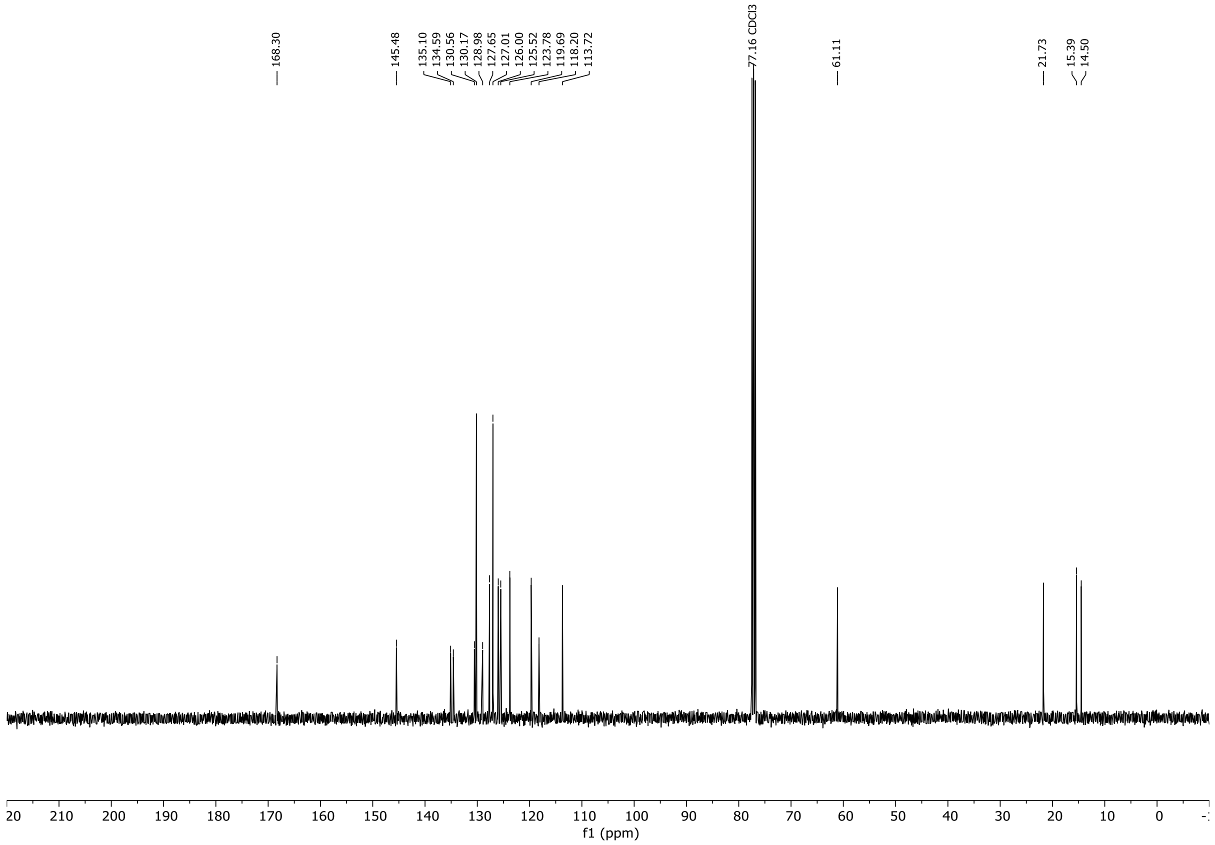

(*E*)-2-Methyl-3-(1-tosyl-1*H*-indol-3-yl)prop-2-en-1-ol

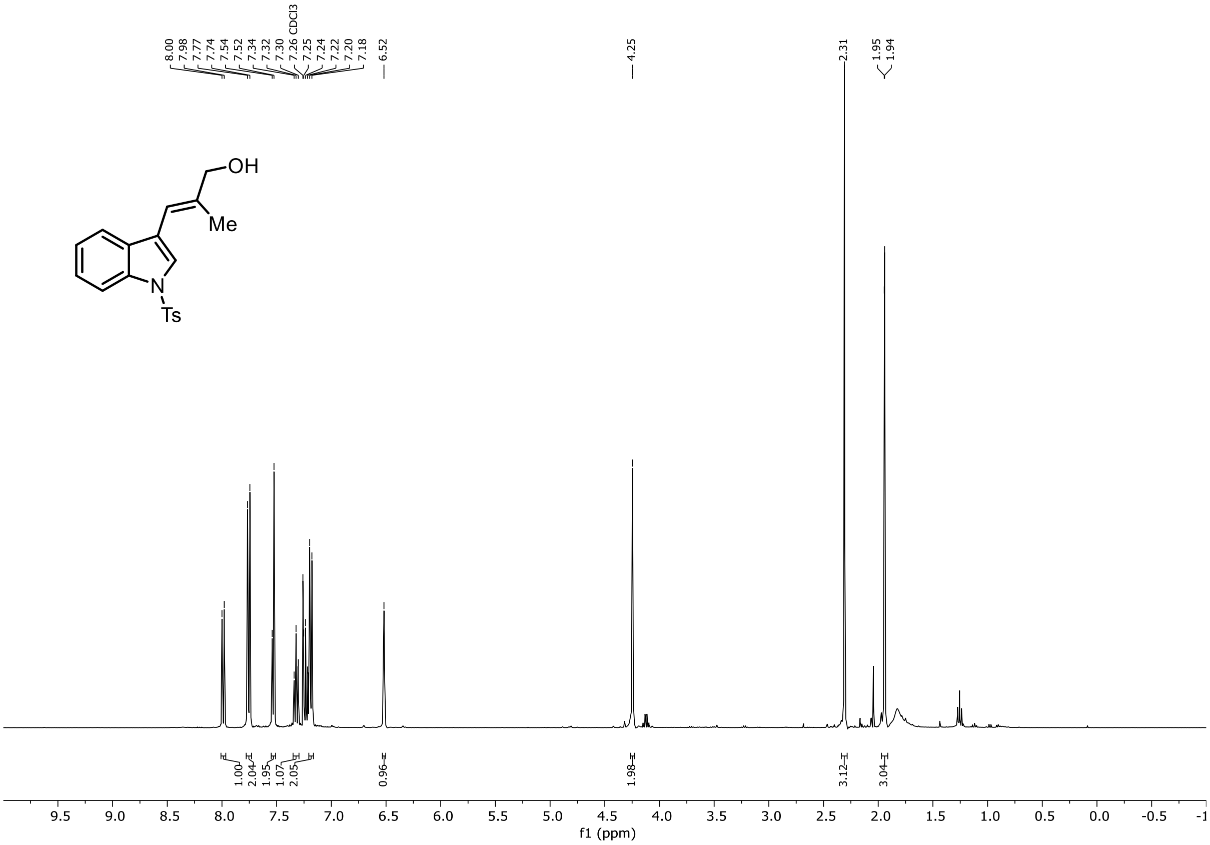

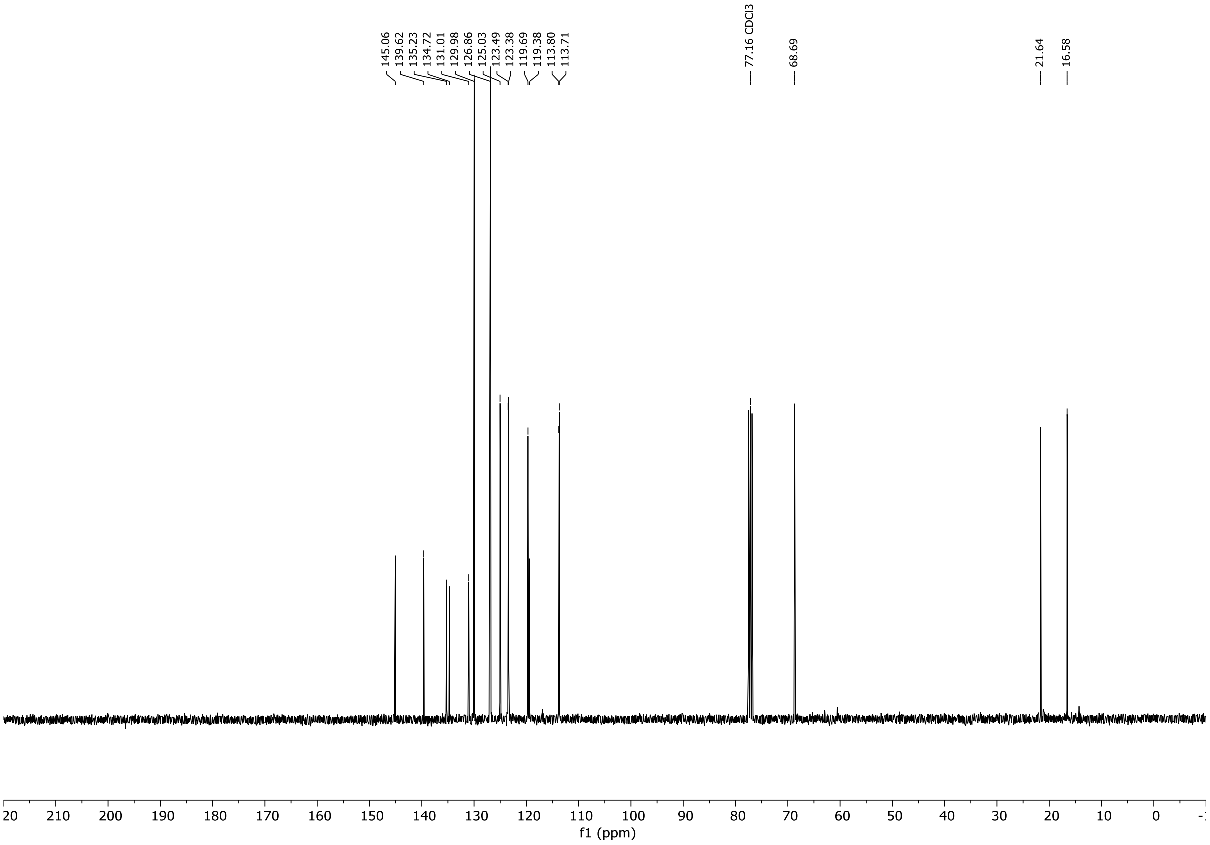

3-(1*H*-indol-3-yl)-4-methylpent-4-enoic acid

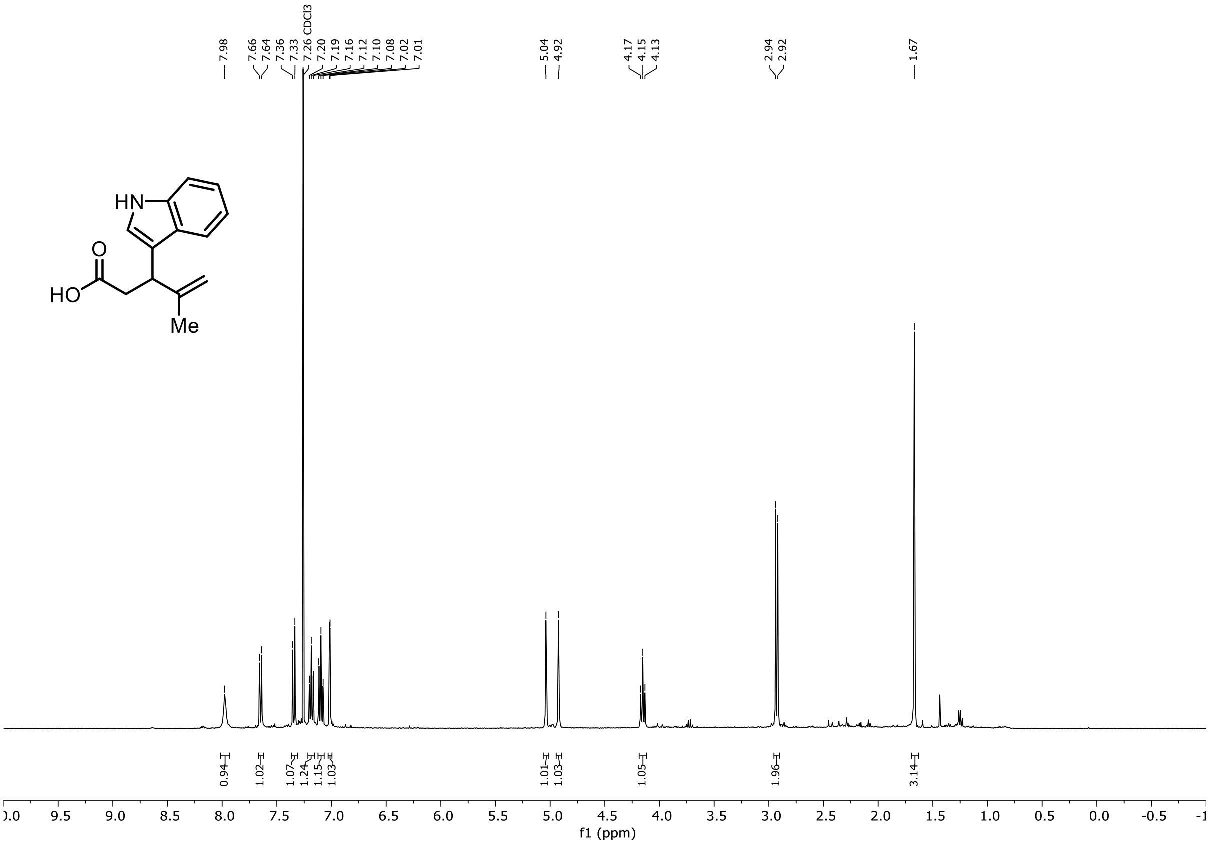

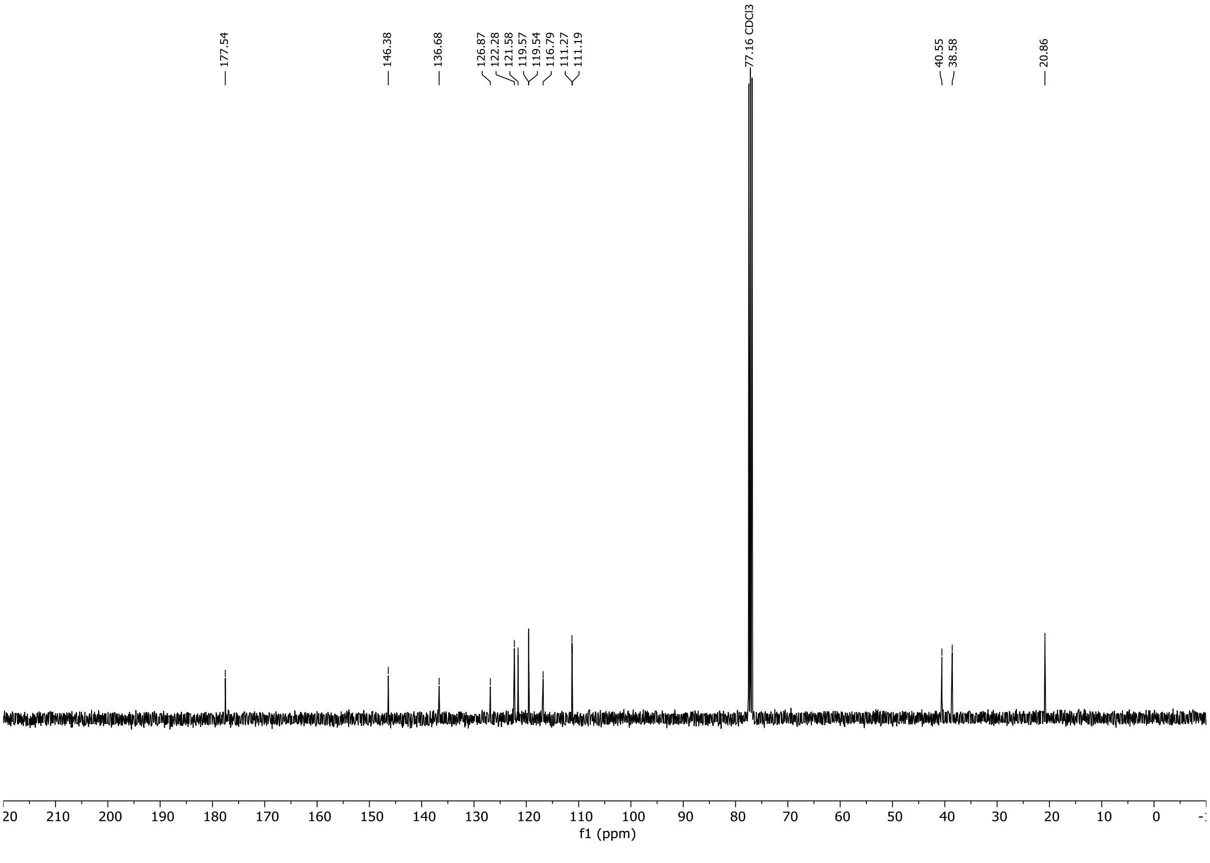

3-(1*H*-indol-3-yl)-4-methylpent-4-en-1-ol

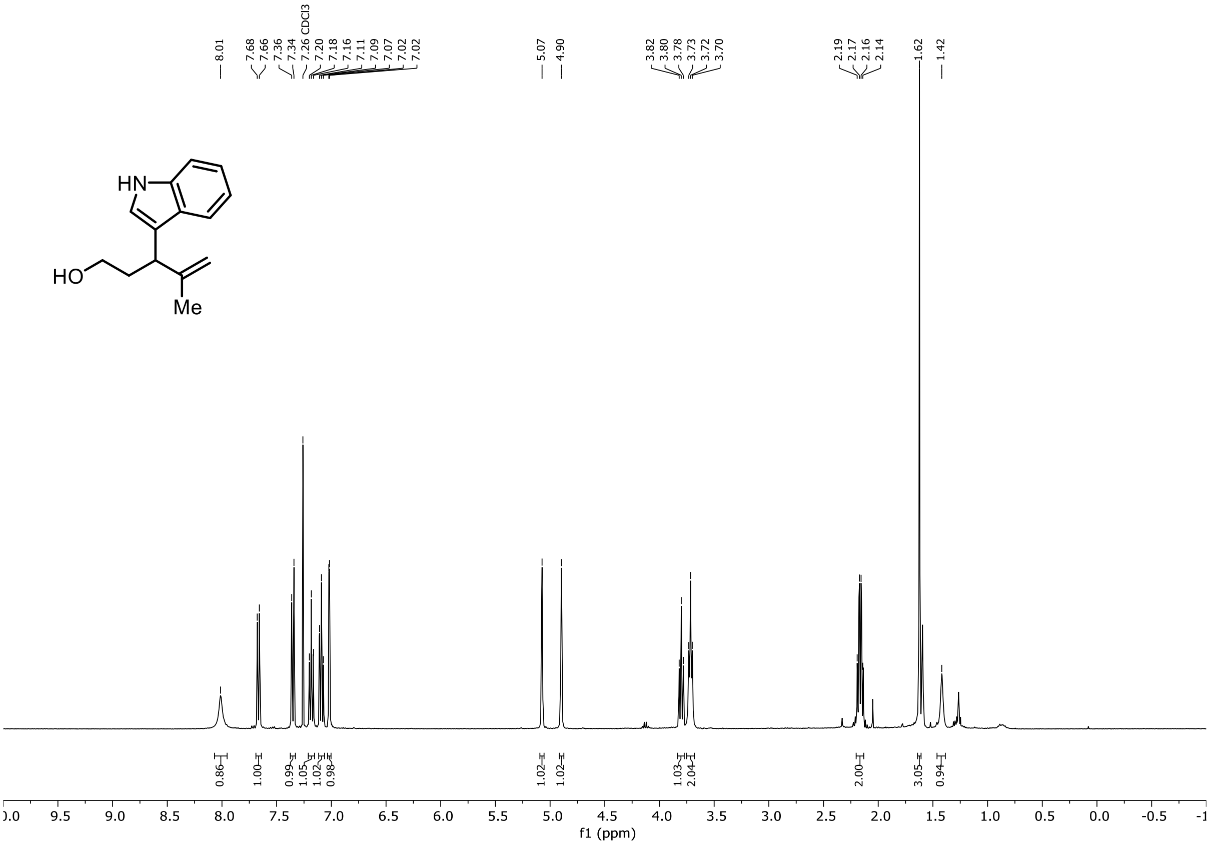

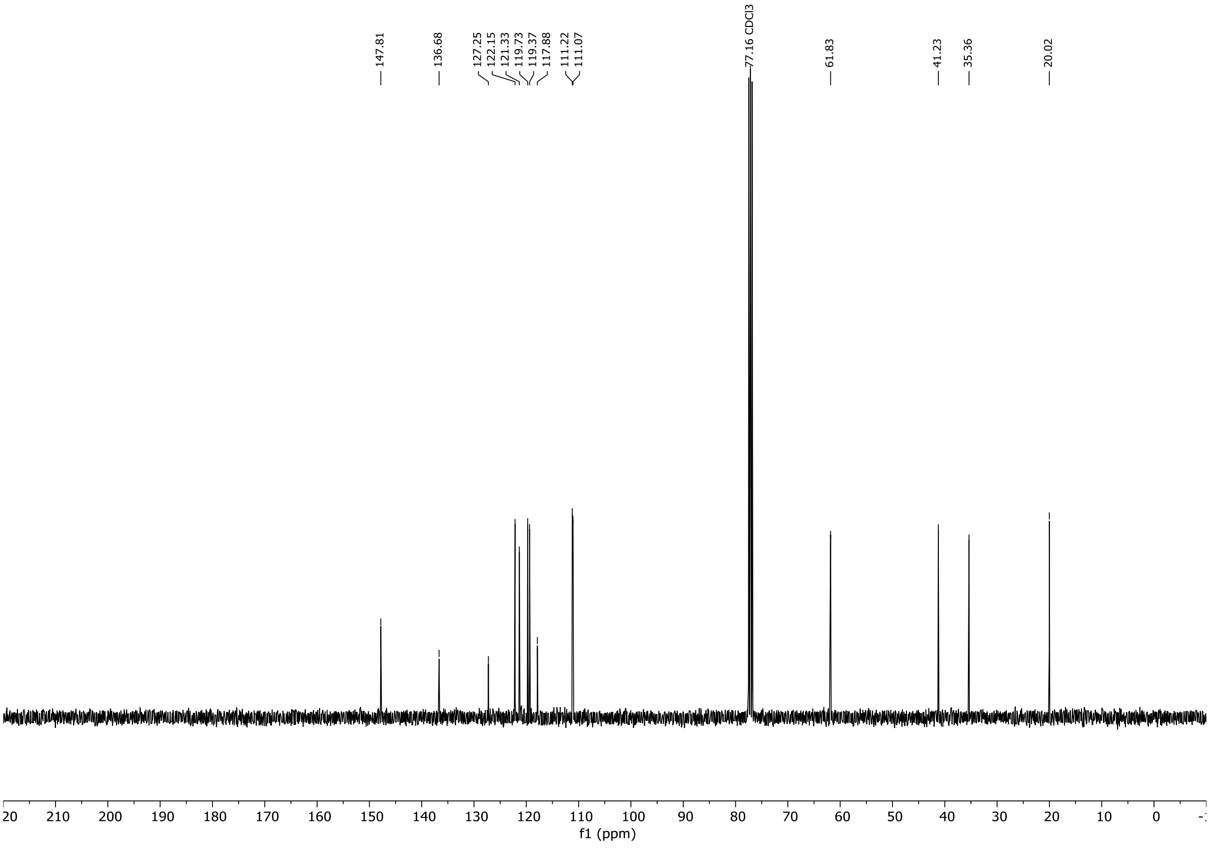

Benzyl (3-(1*H*-indol-3-yl)-4-methylpent-4-en-1-yl)((perfluorobenzoyl)oxy)carbamate (1n)

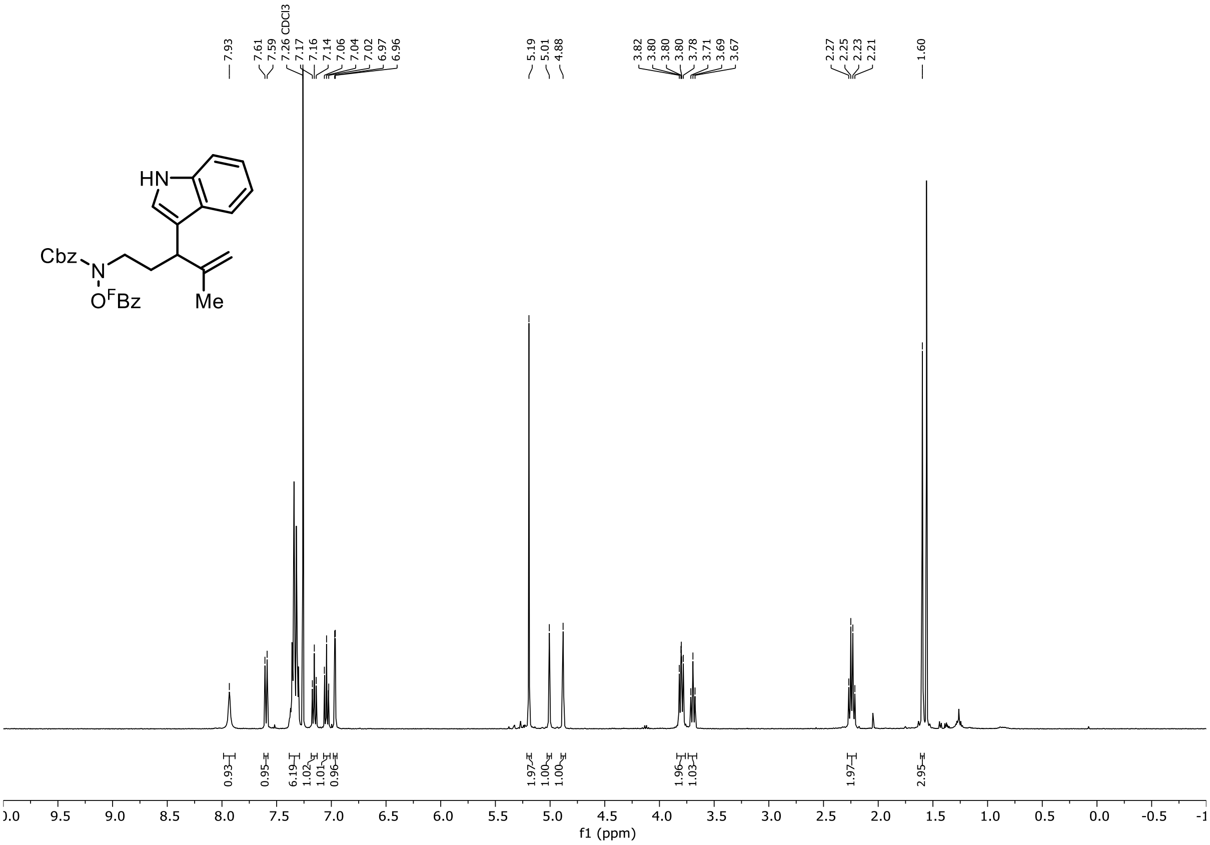

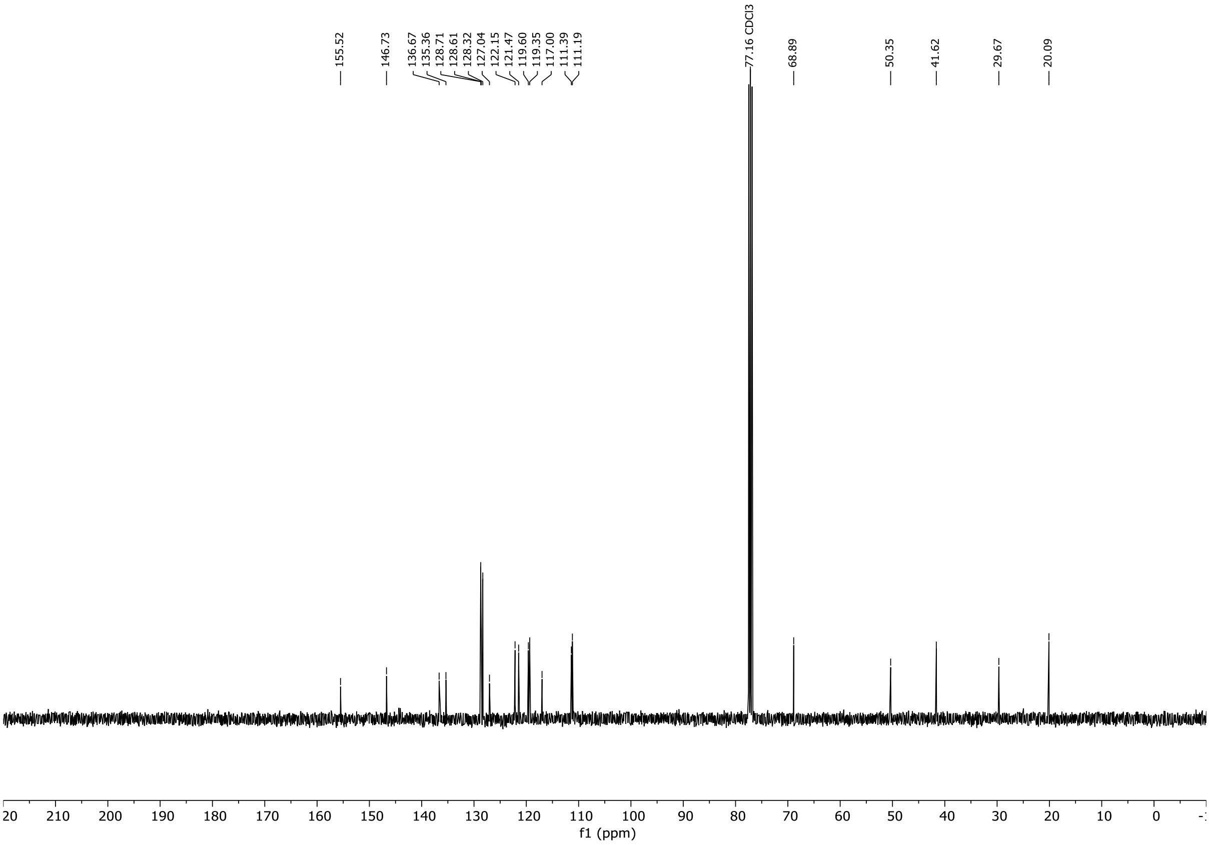

Benzyl 9a-methyl-2,3,3a,8,9,9a-hexahydro-1*H*-pyrrolo[3',2':3,4]cyclopenta[1,2-*b*]indole-1-carboxylate (2n)

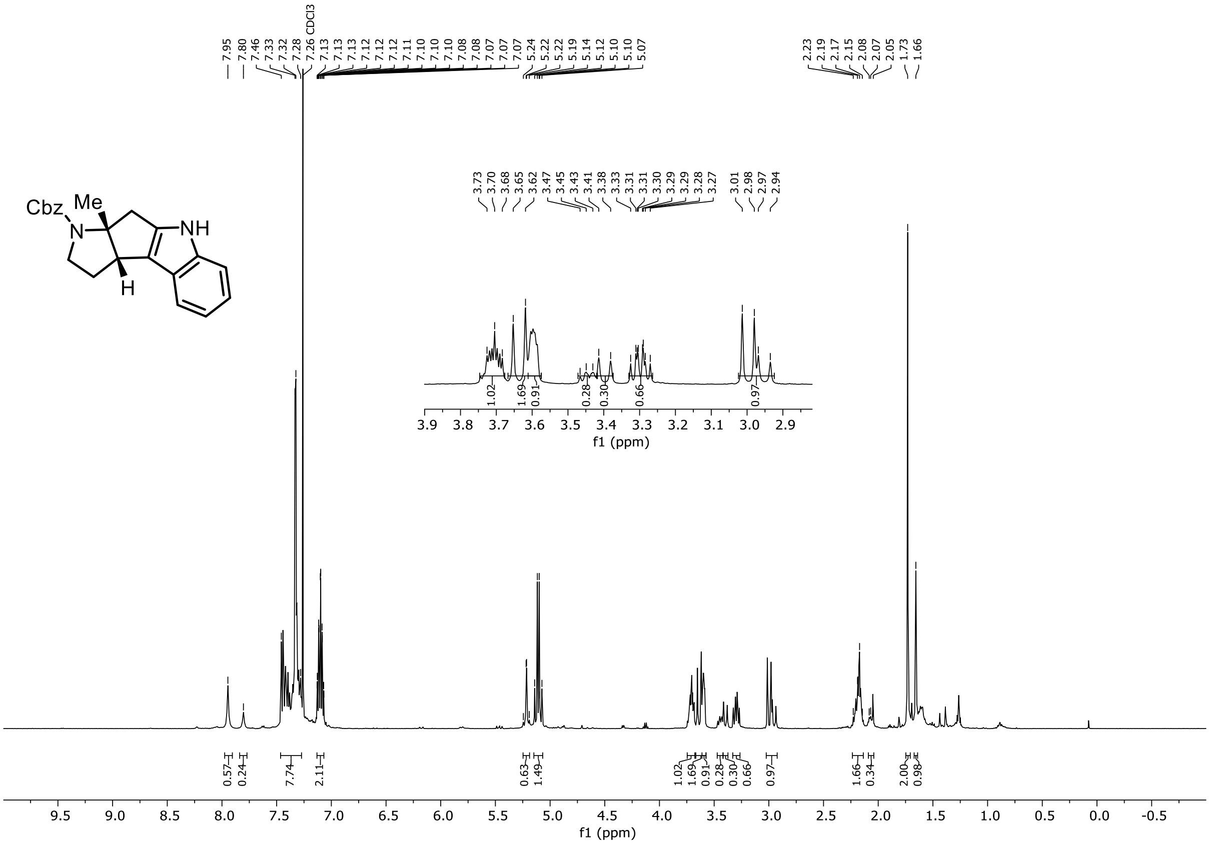

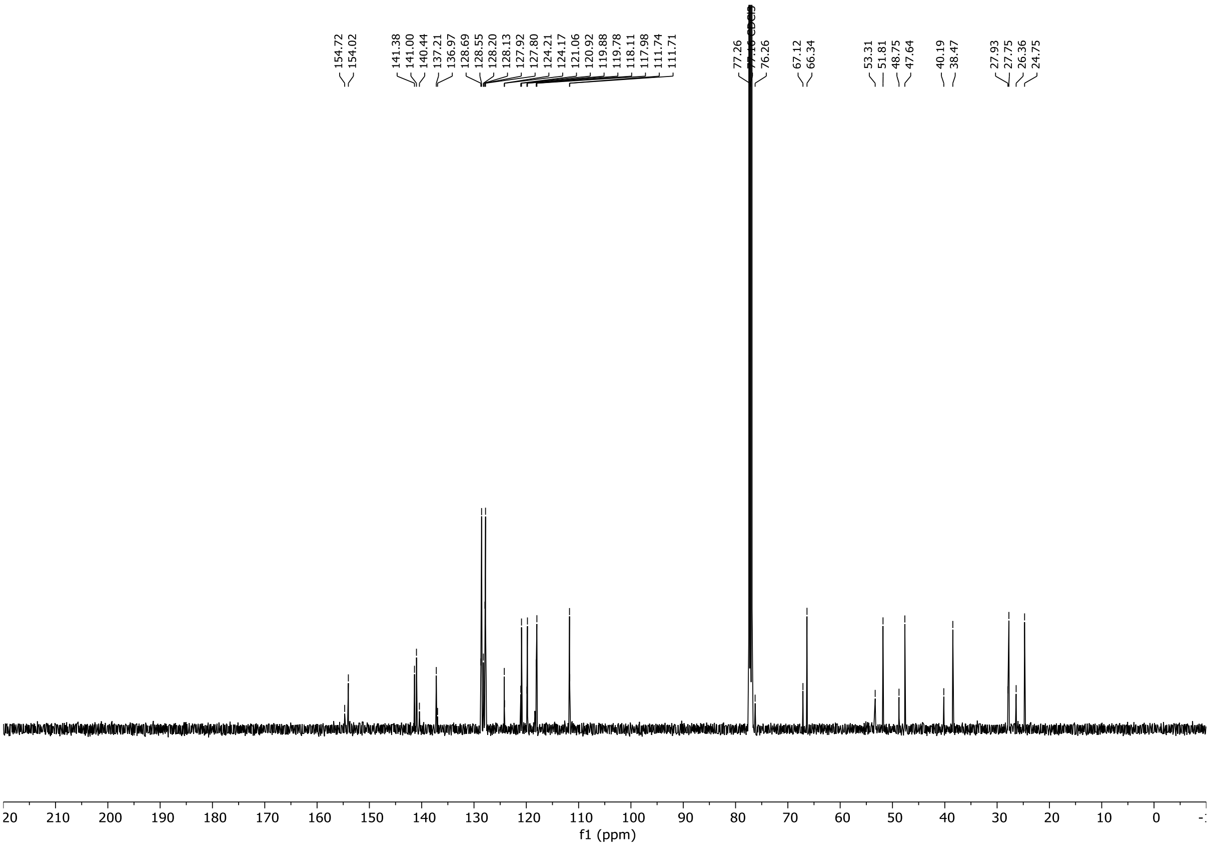

Ethyl 4-benzylpent-4-enoate

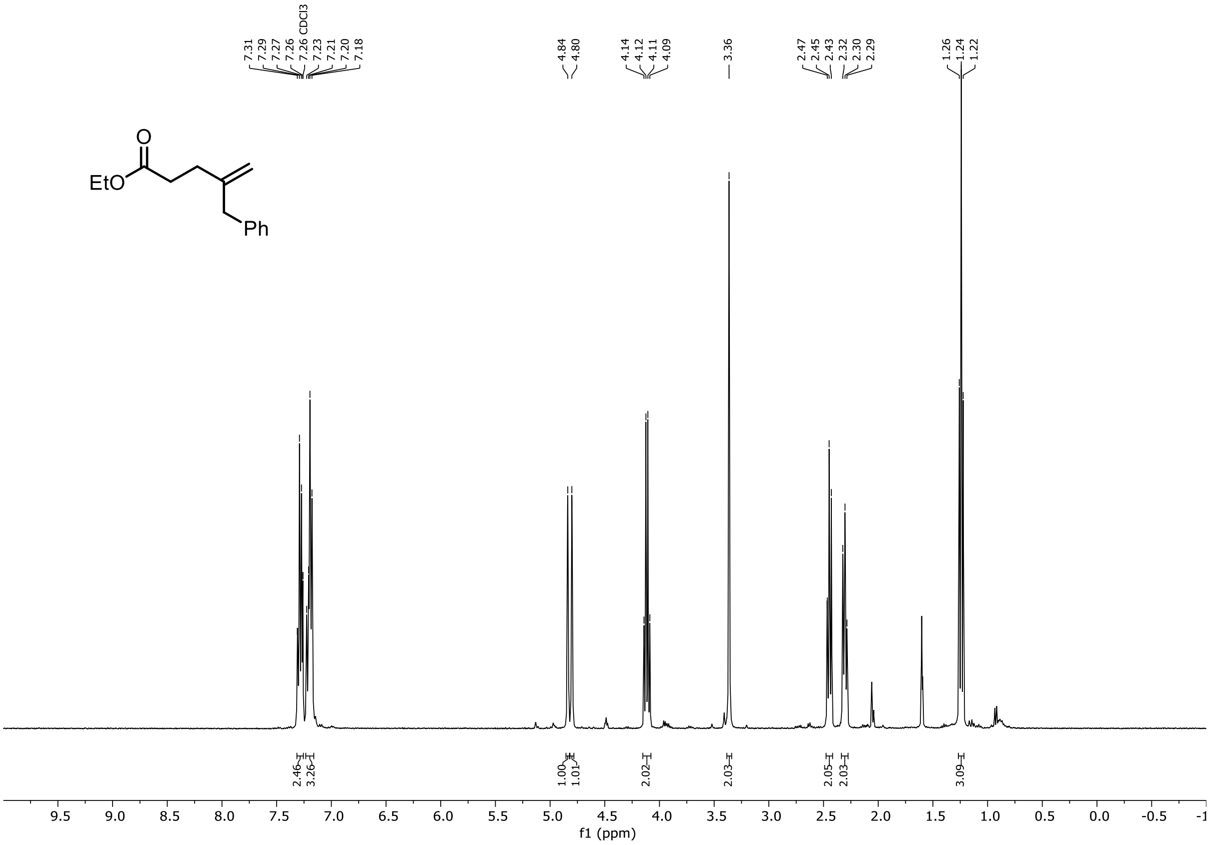

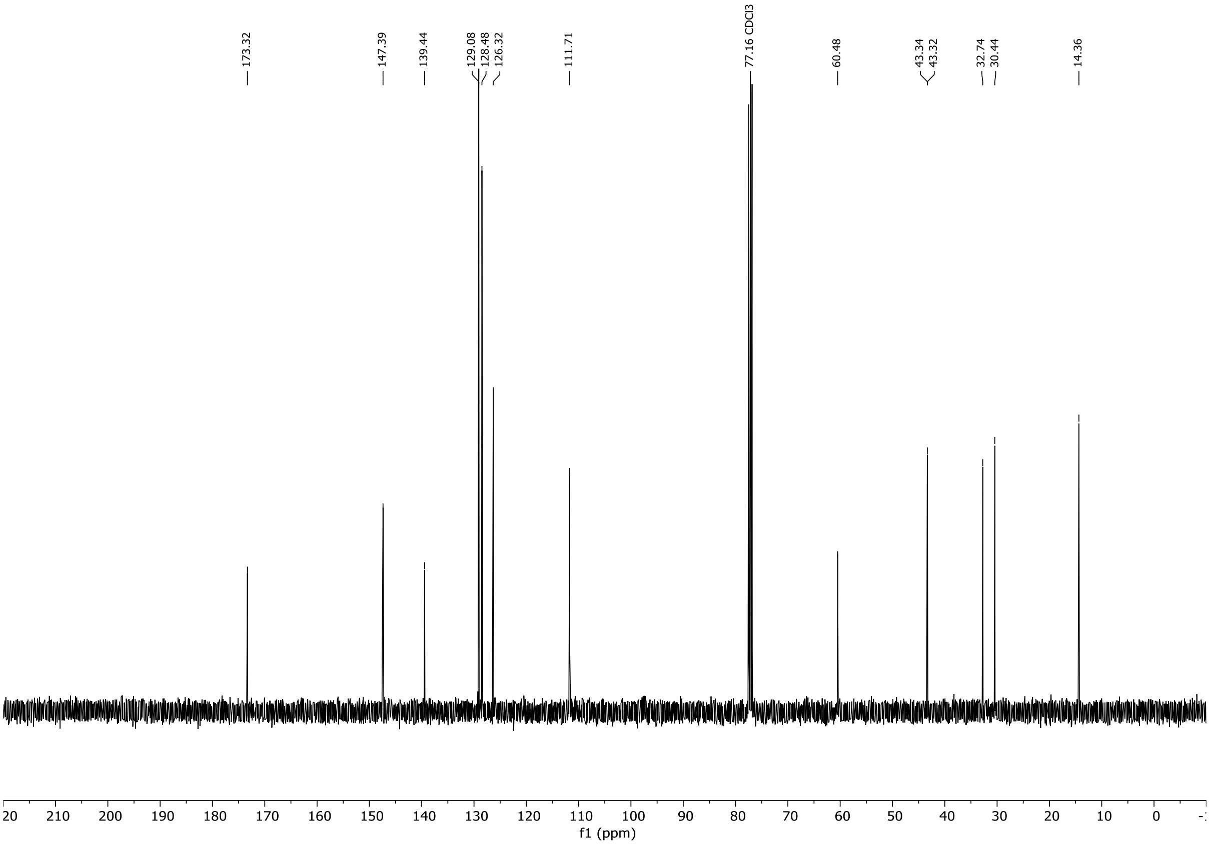

4-Benzylpent-4-en-1-ol

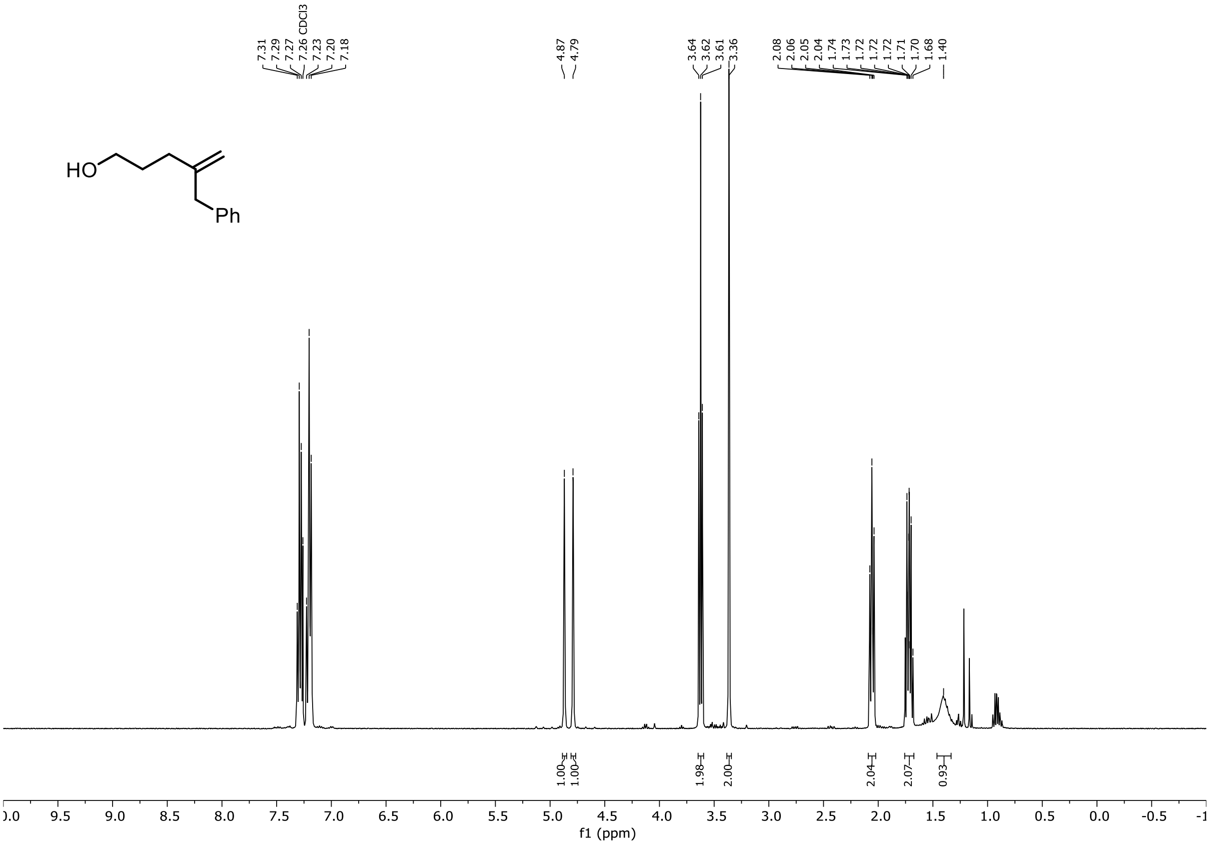

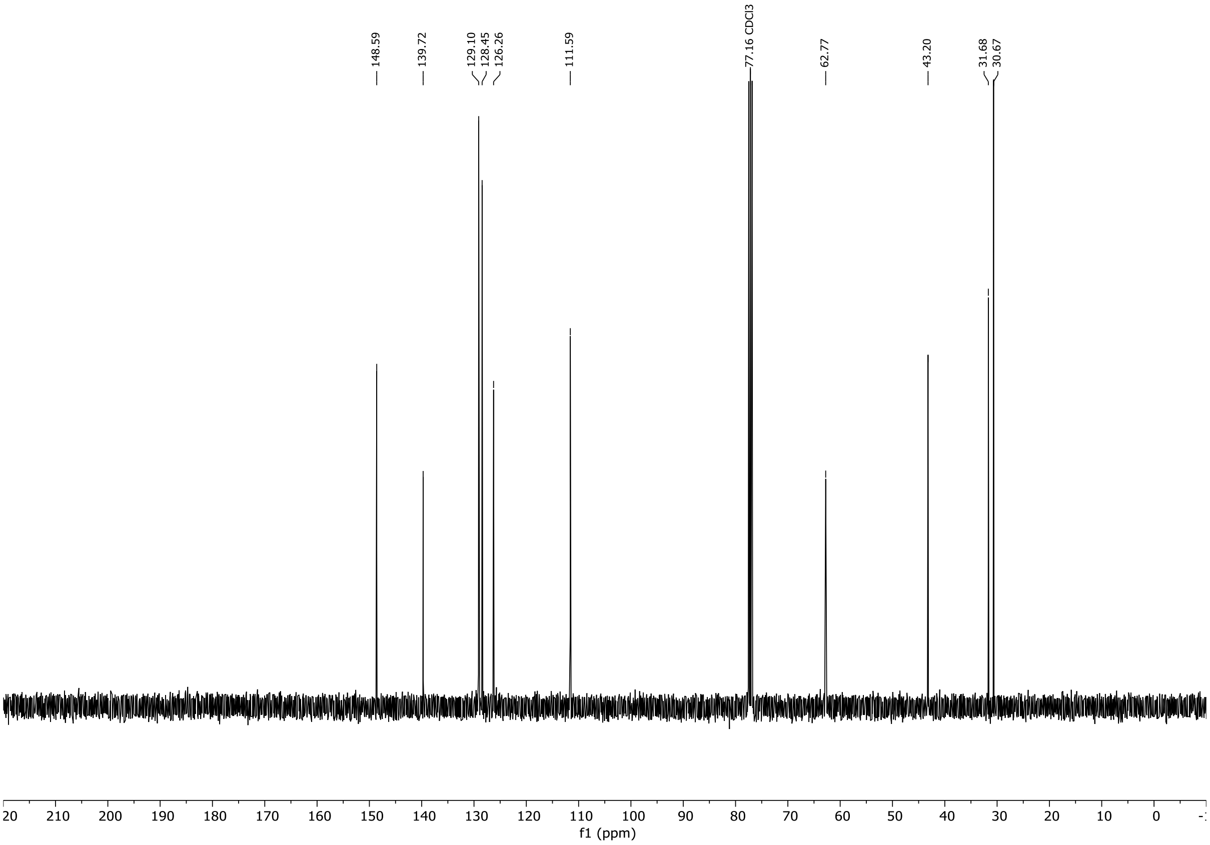

Benzyl (4-benzylpent-4-en-1-yl)((perfluorobenzoyl)oxy)carbamate (3a)

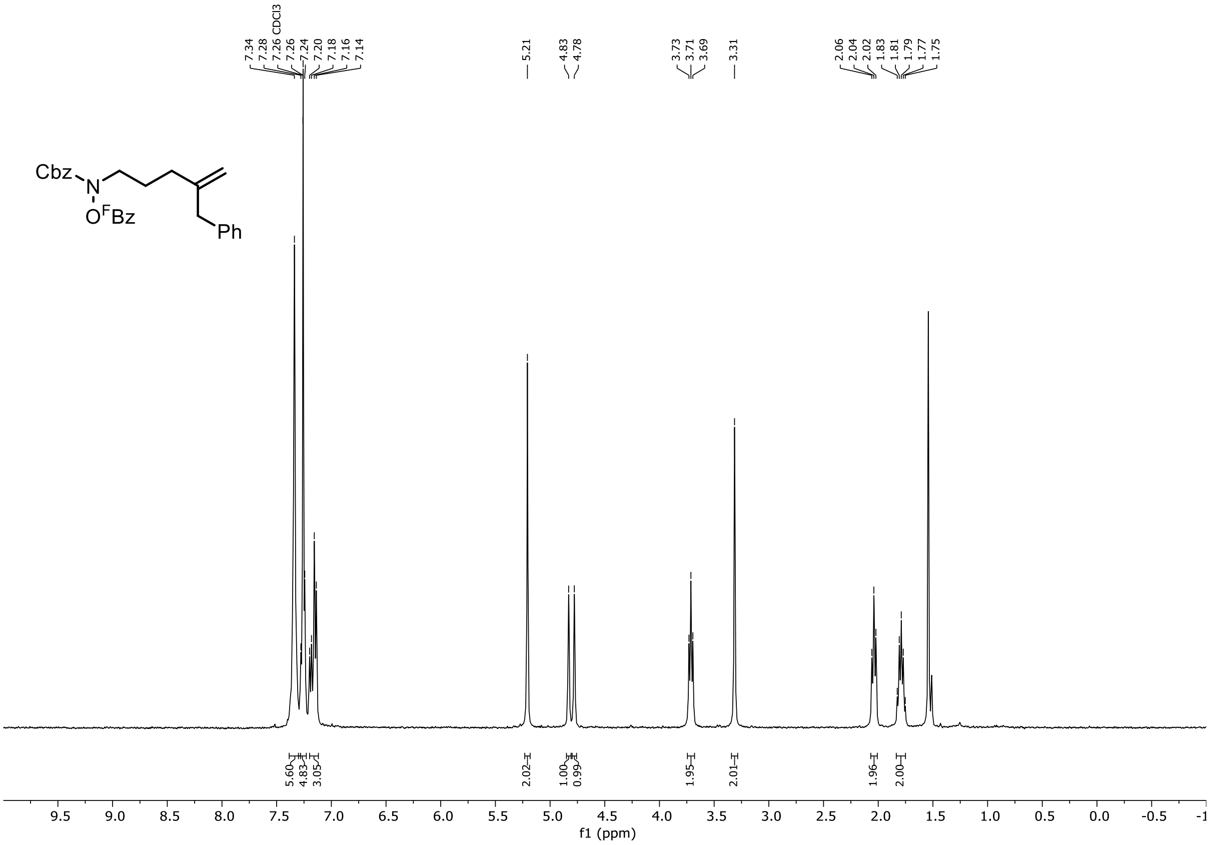

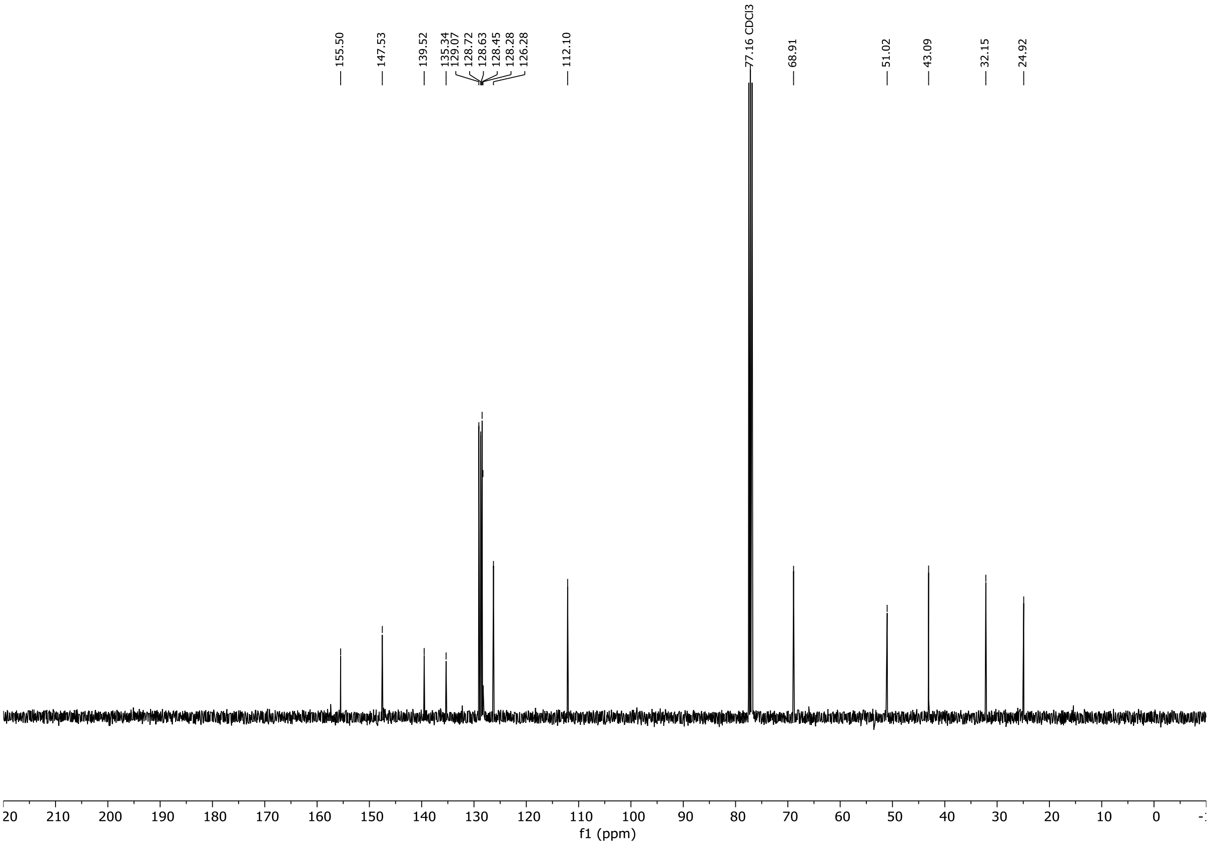

Benzyl 1,3-dihydrospiro[indene-2,2'-pyrrolidine]-1'-carboxylate (4a)

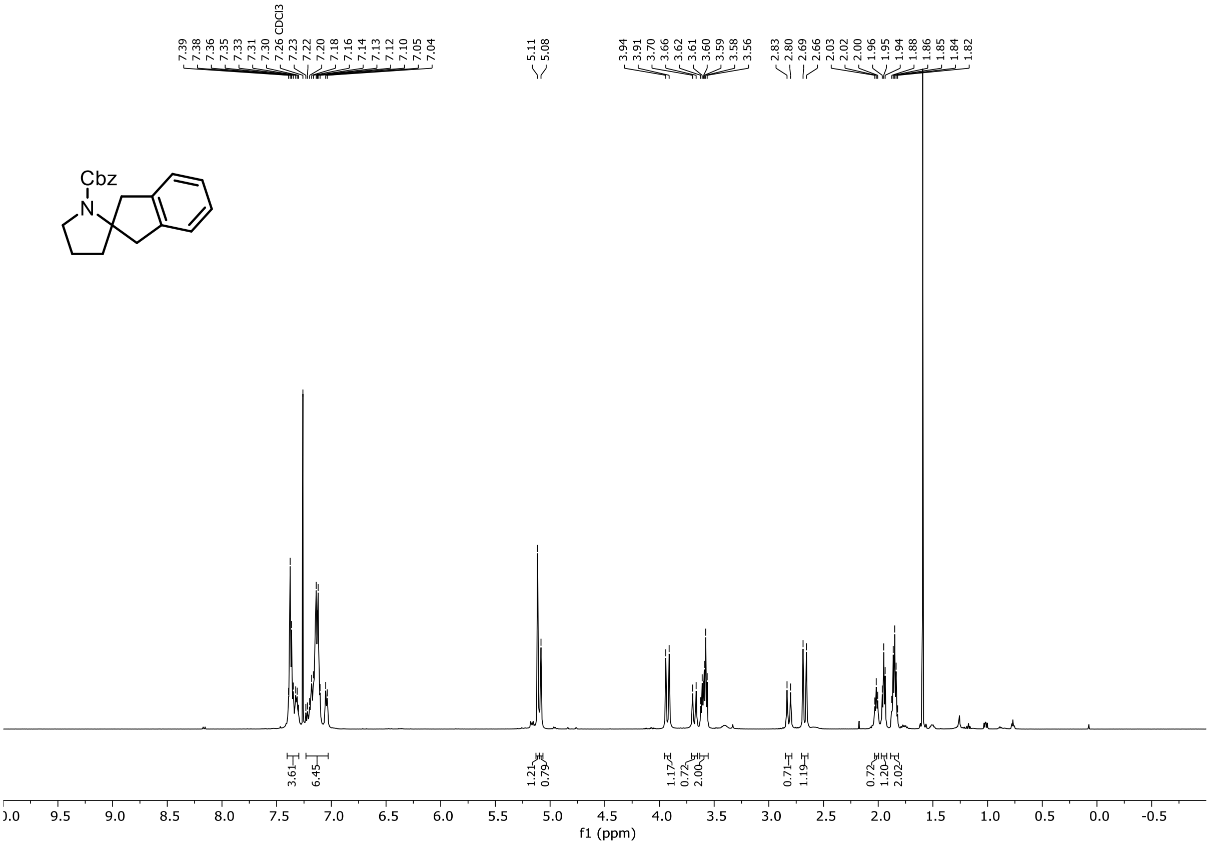

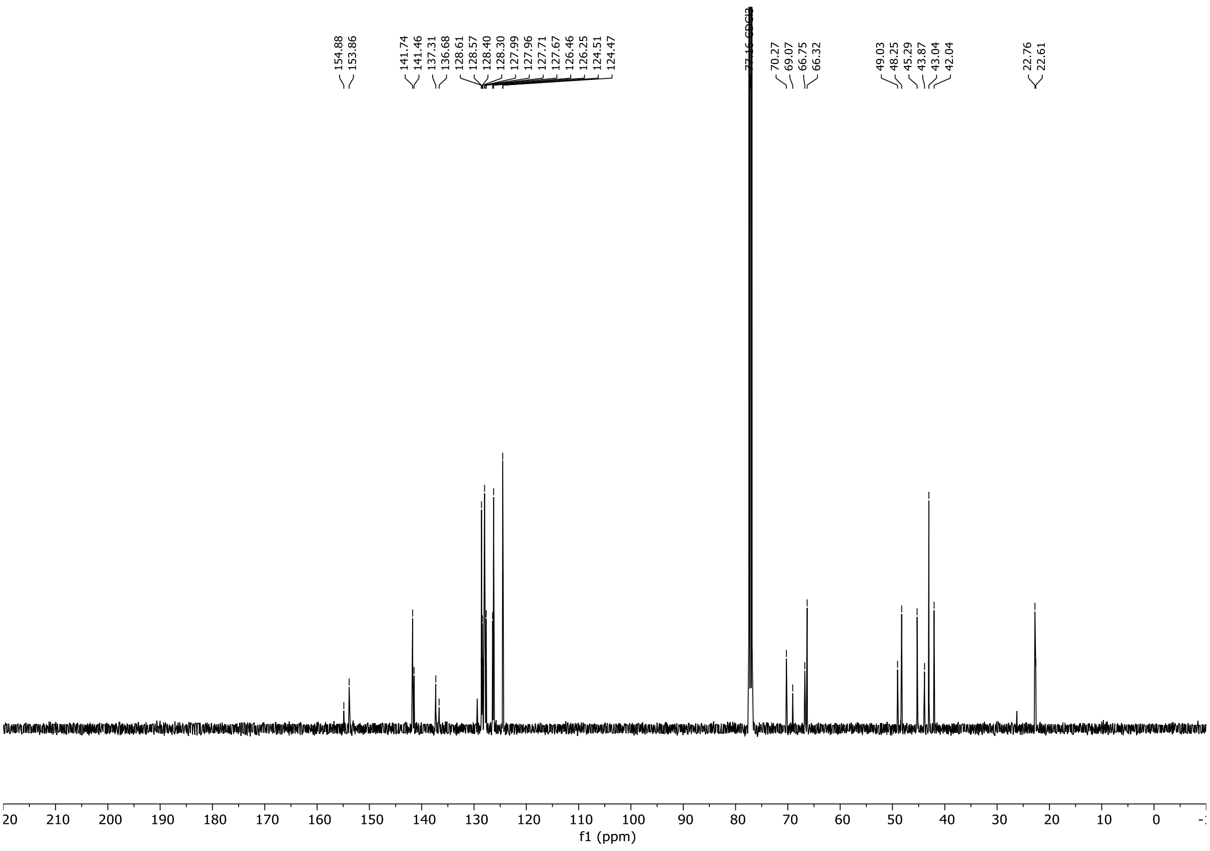

Ethyl 4-(4-methoxybenzyl)pent-4-enoate

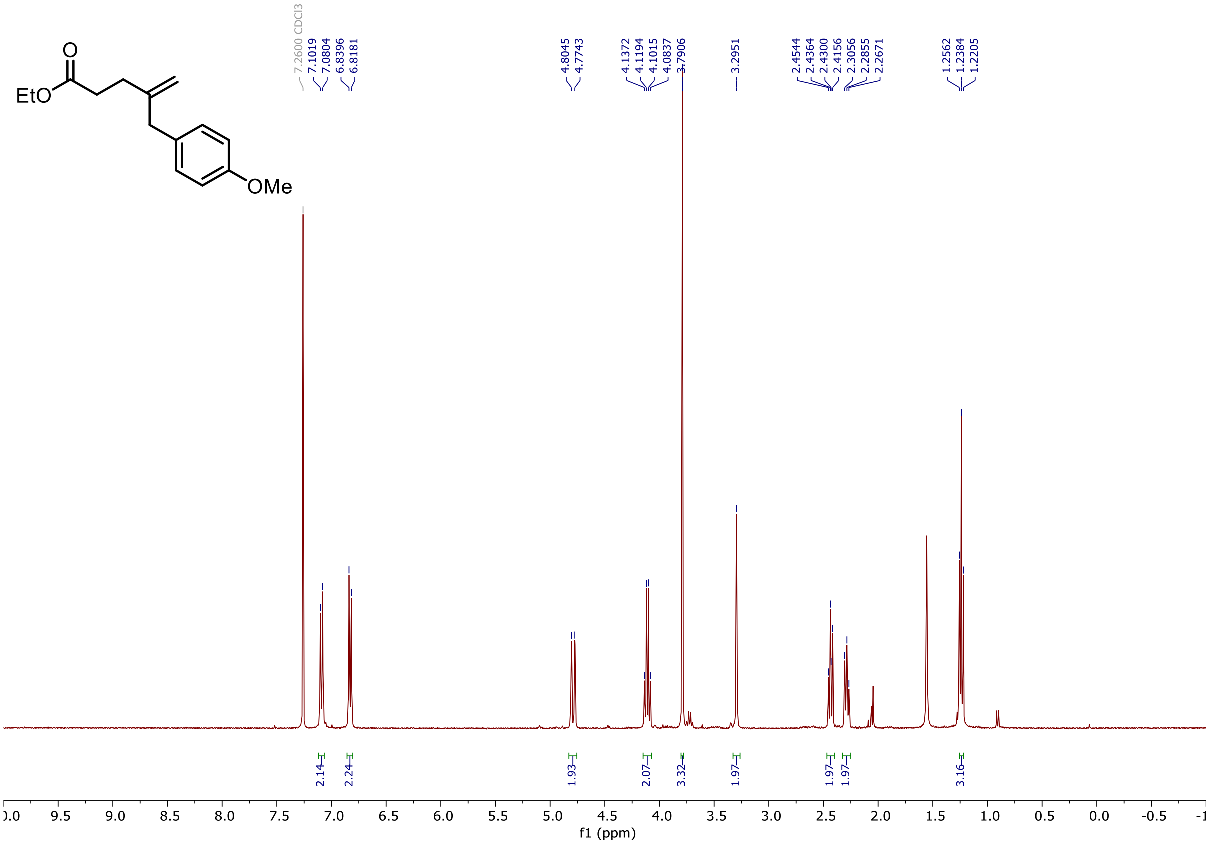

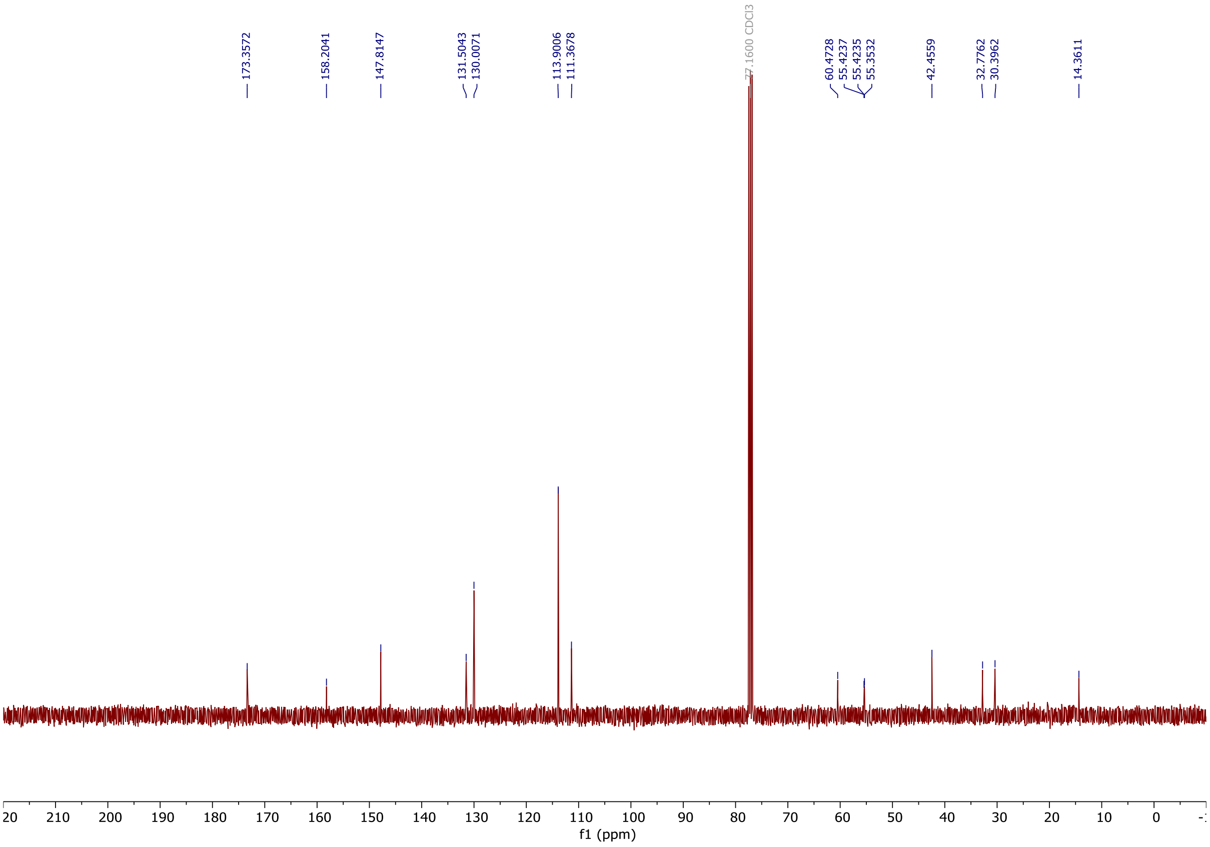

4-(4-Methoxybenzyl)pent-4-en-1-ol

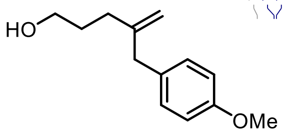

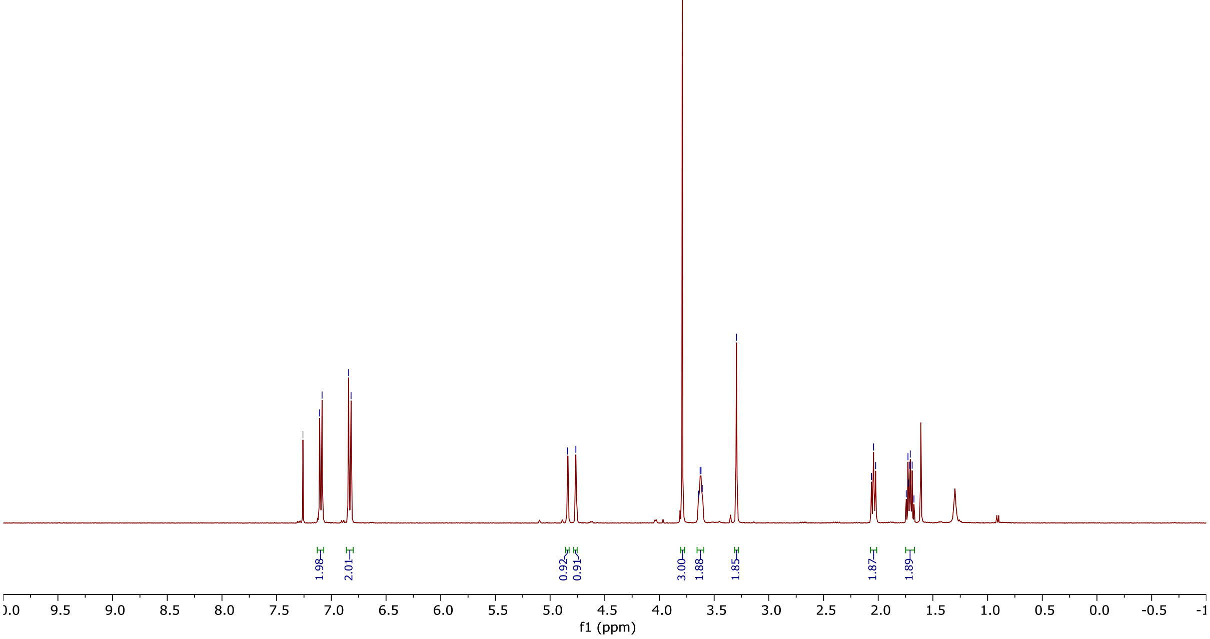

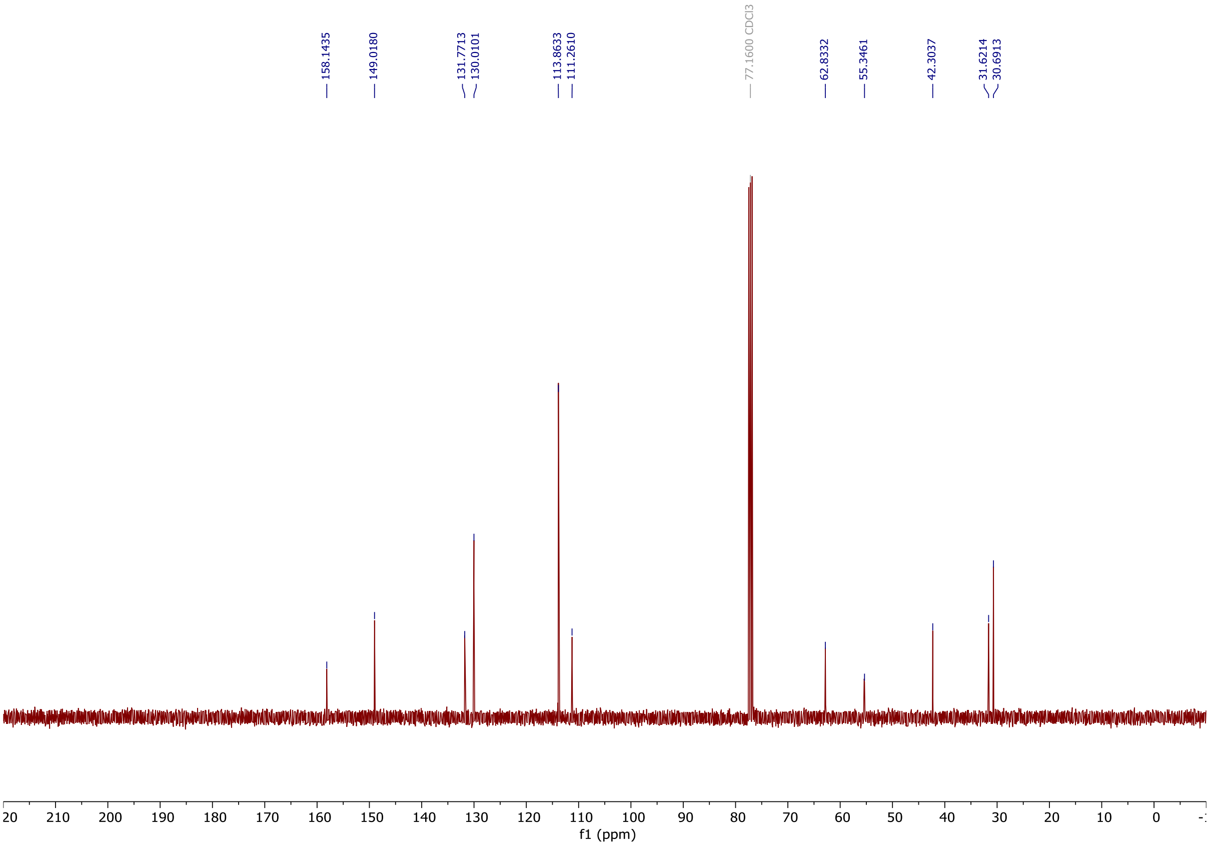

Benzyl (4-(4-methoxybenzyl)pent-4-en-1-yl)((perfluorobenzoyl)oxy)carbamate (3b)

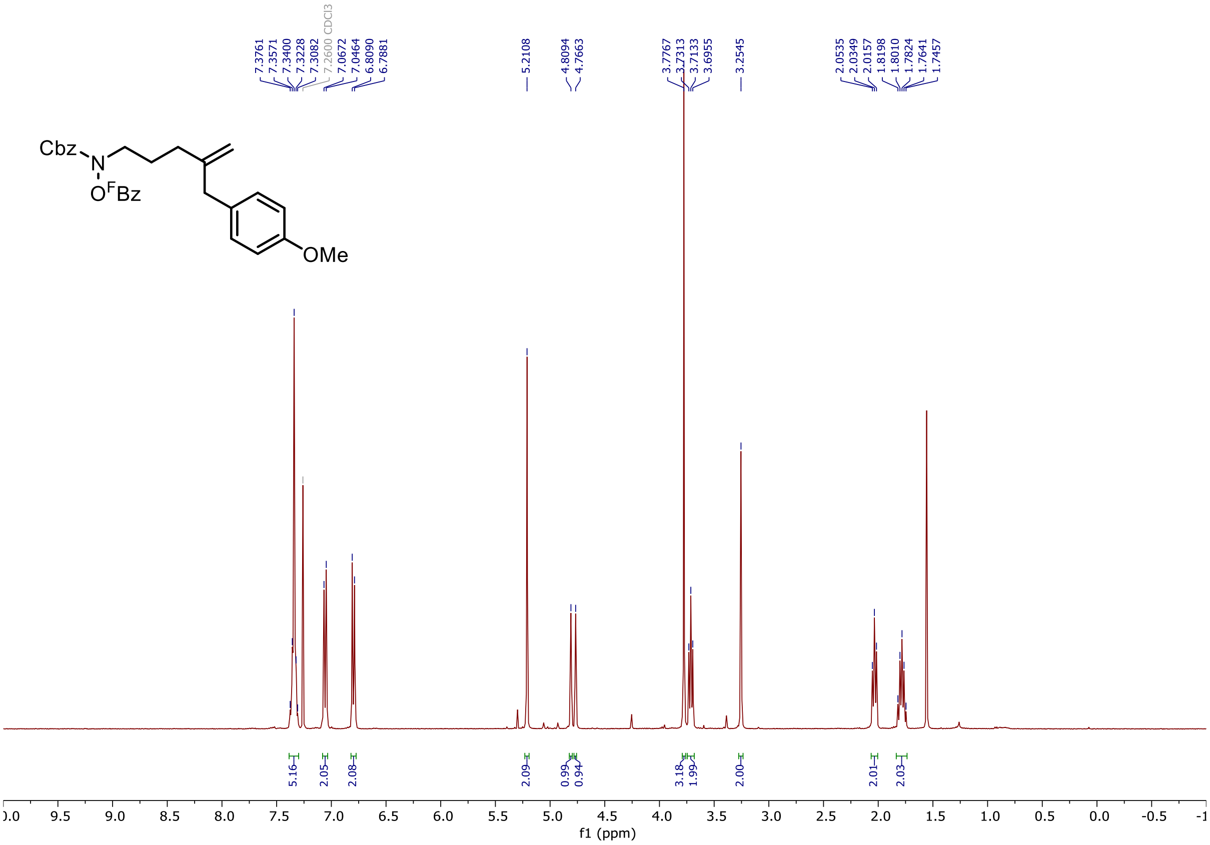

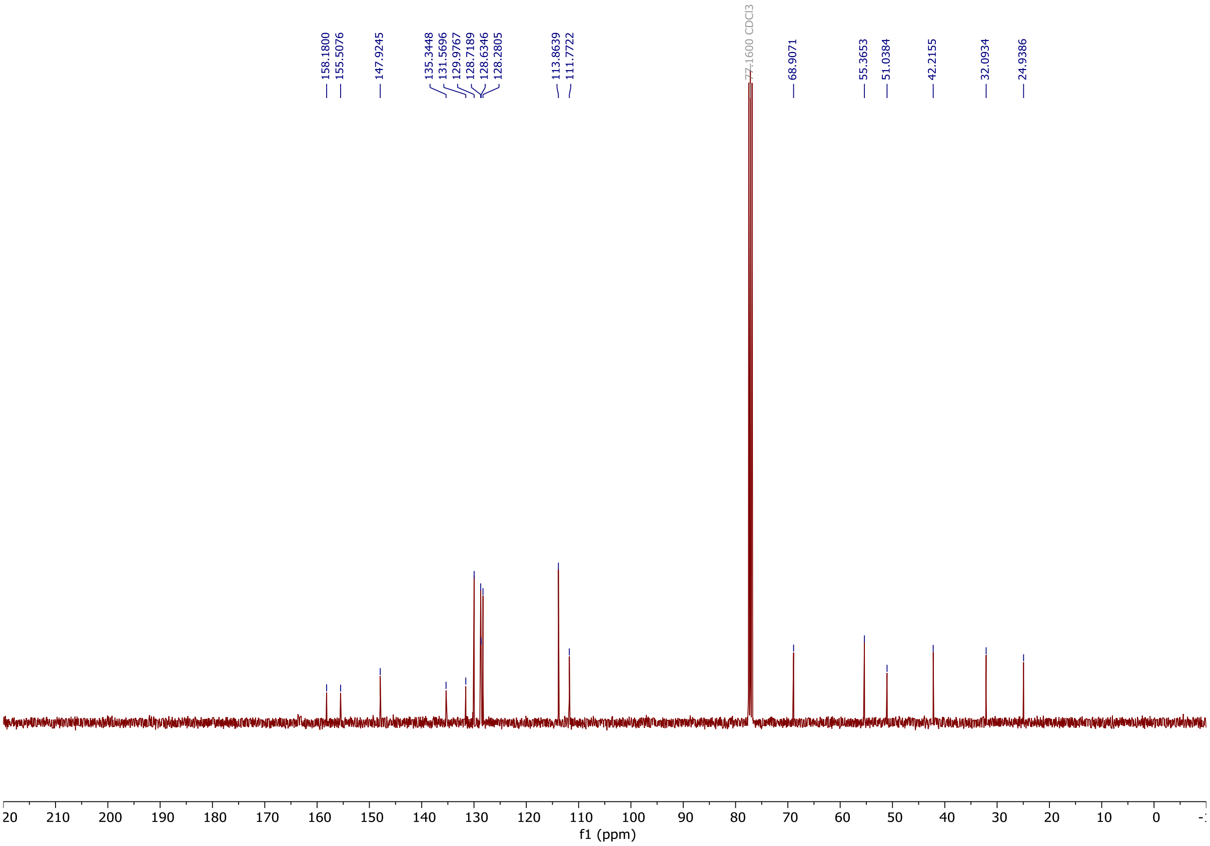

Benzyl 5-methoxy-1,3-dihydrospiro[indene-2,2'-pyrrolidine]-1'-carboxylate (4b)

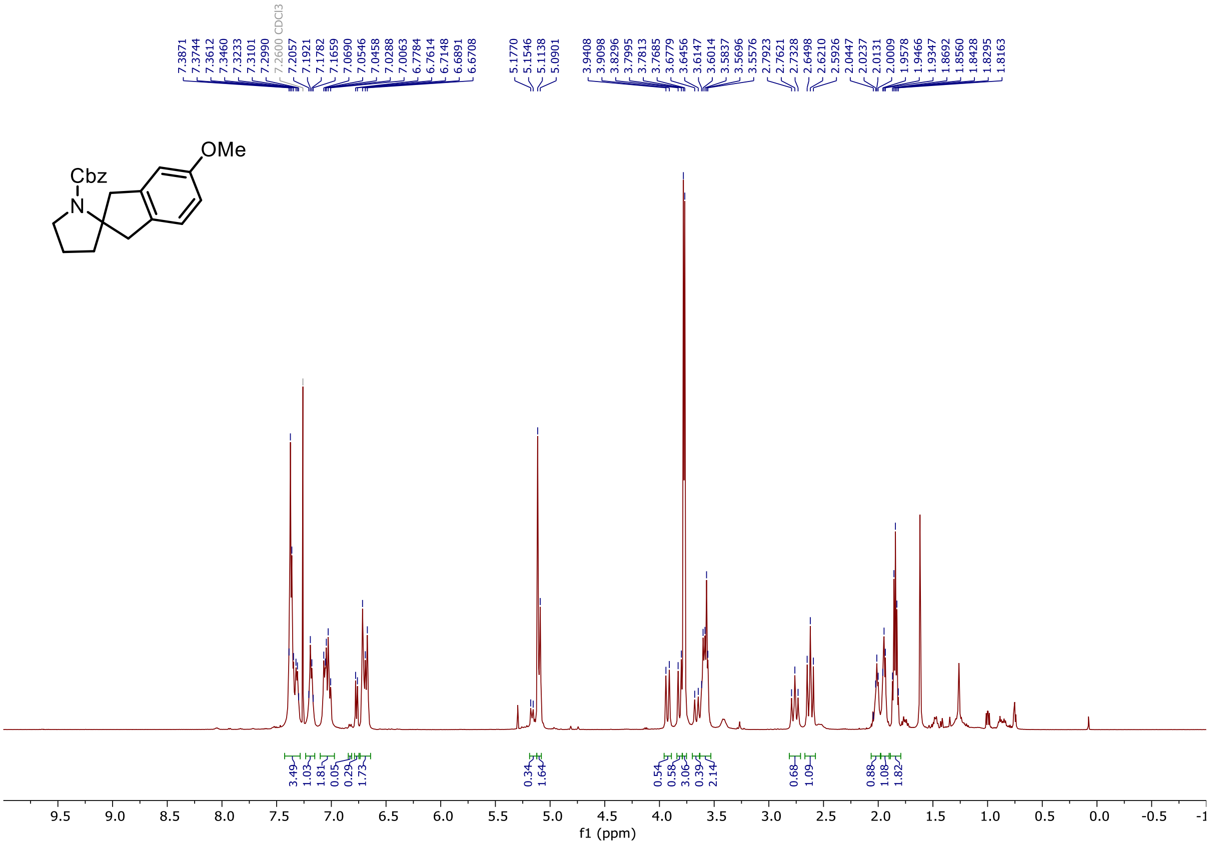

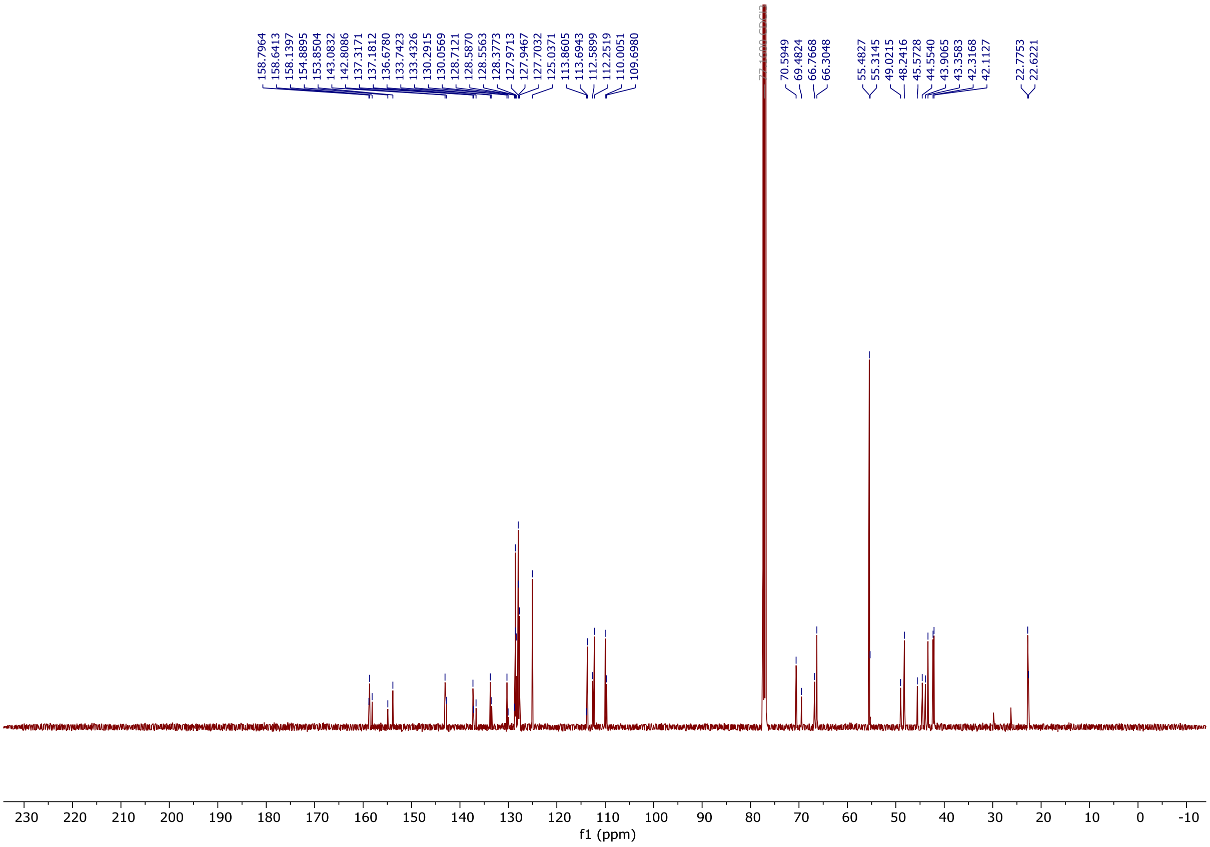

Ethyl 4-(4-nitrobenzyl)pent-4-enoate

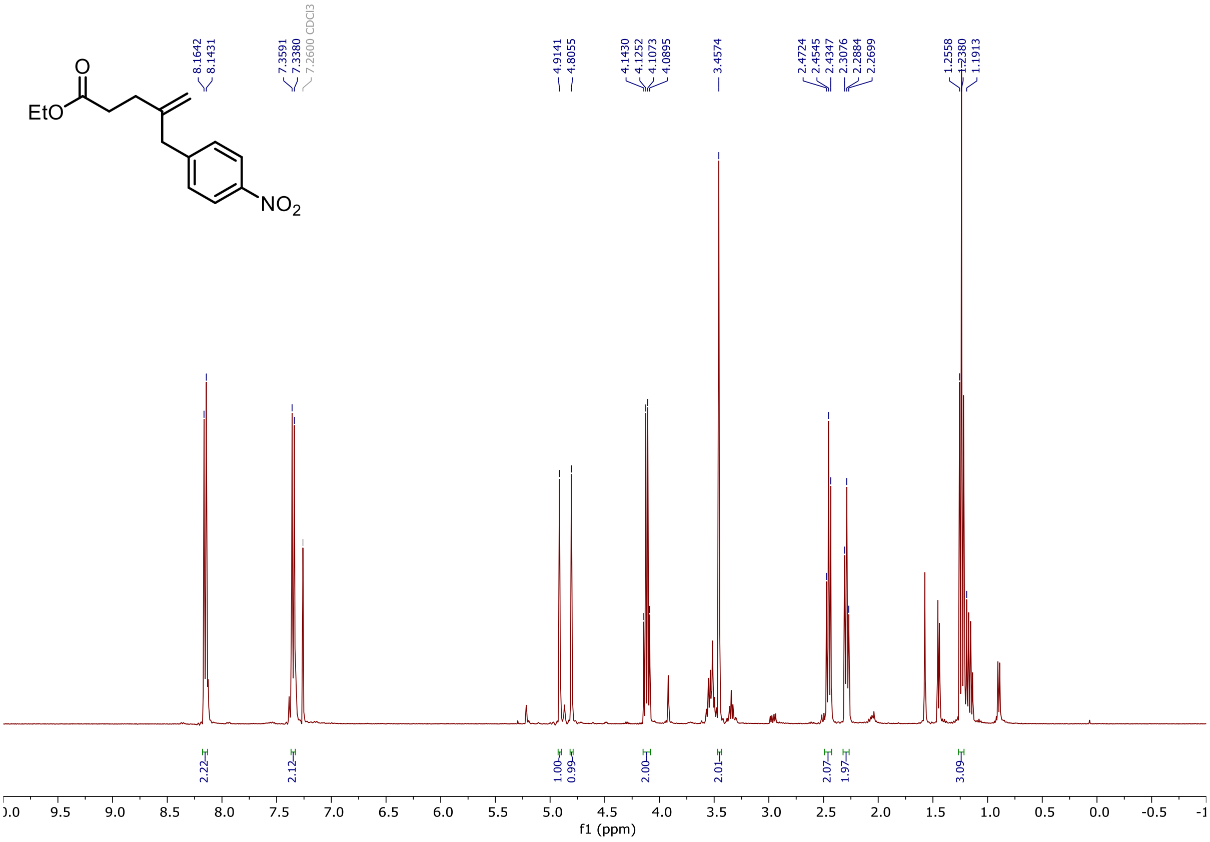

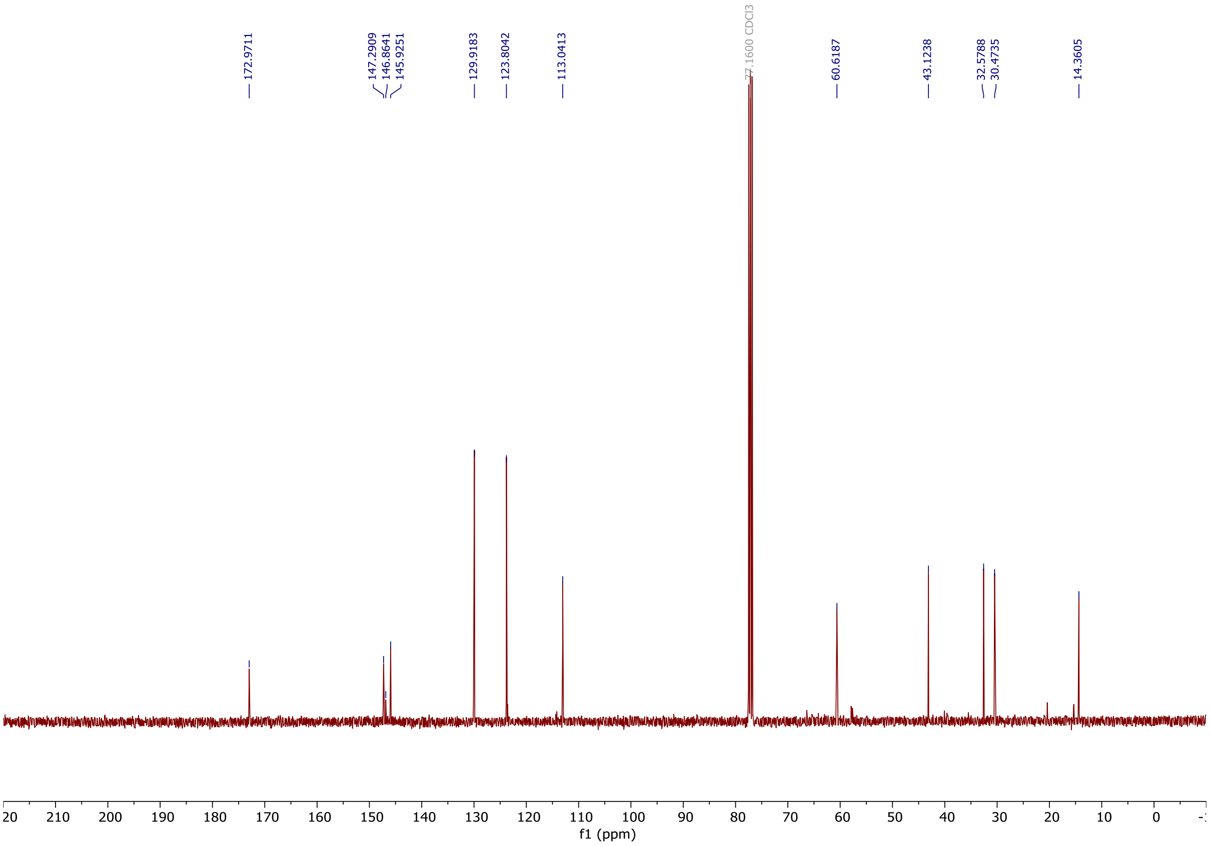

4-(4-Nitrobenzyl)pent-4-en-1-ol

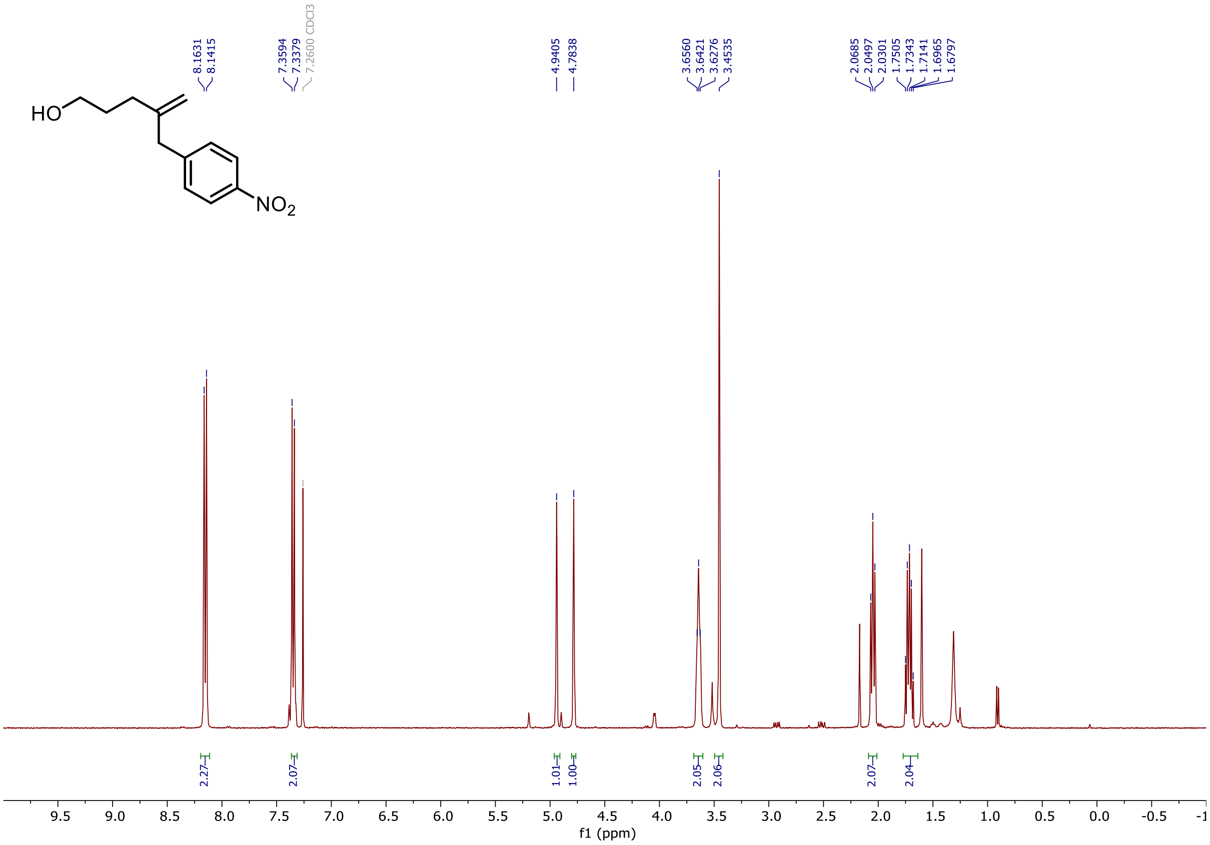

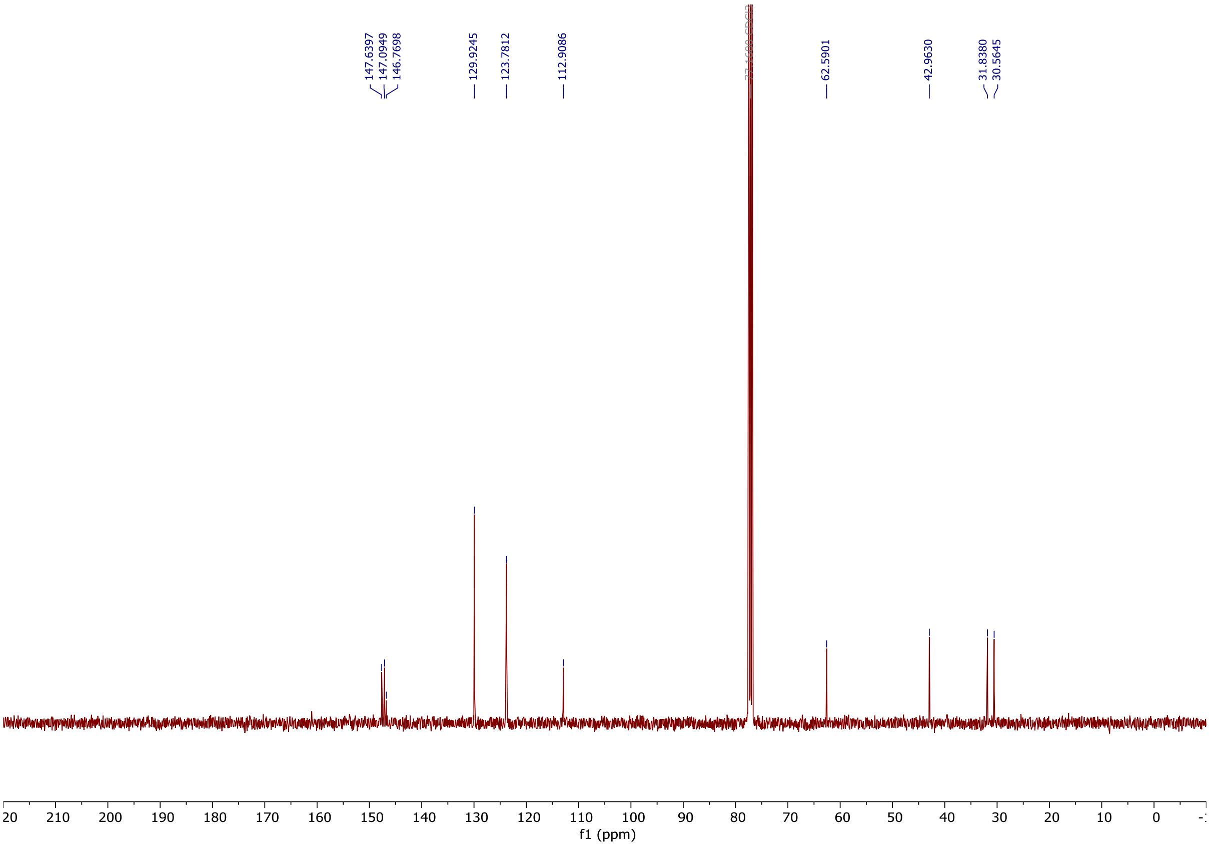

Benzyl (4-(4-nitrobenzyl)pent-4-en-1-yl)((perfluorobenzoyl)oxy)carbamate (3c)

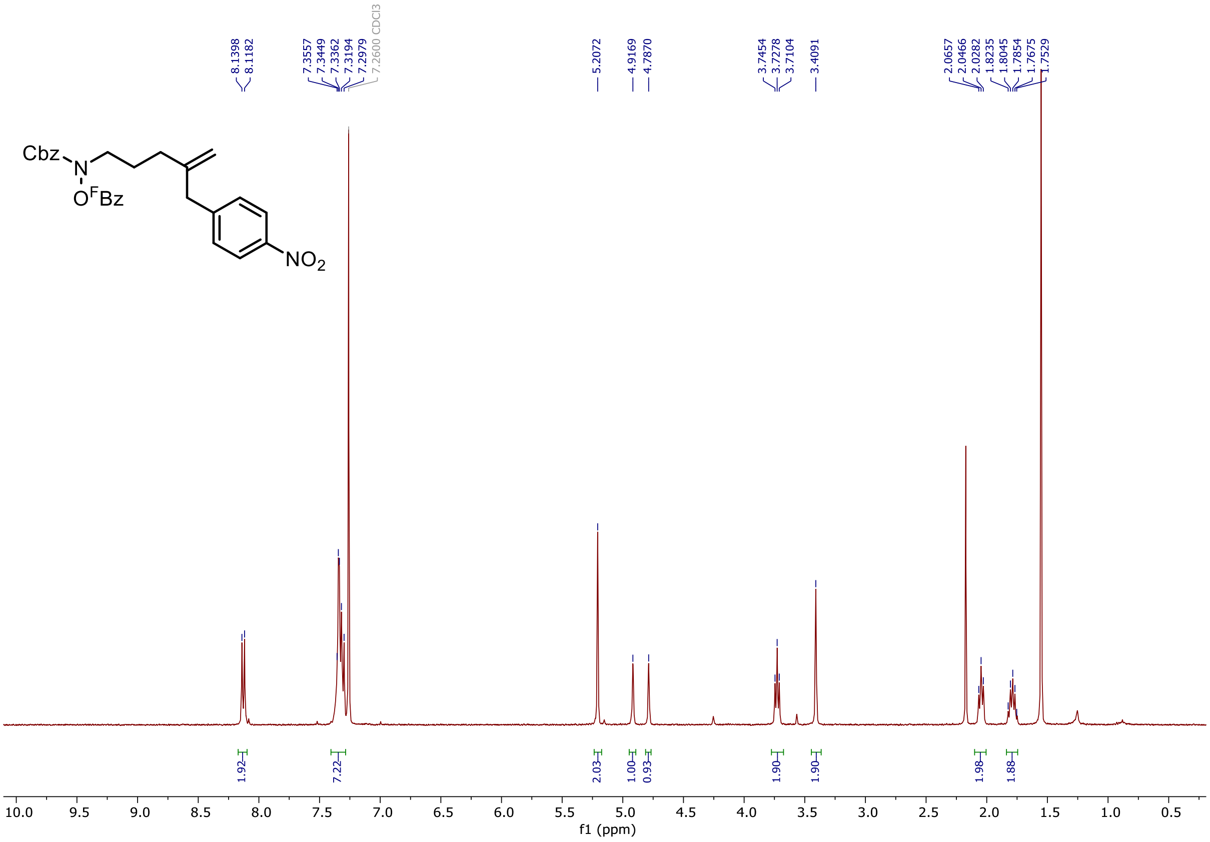

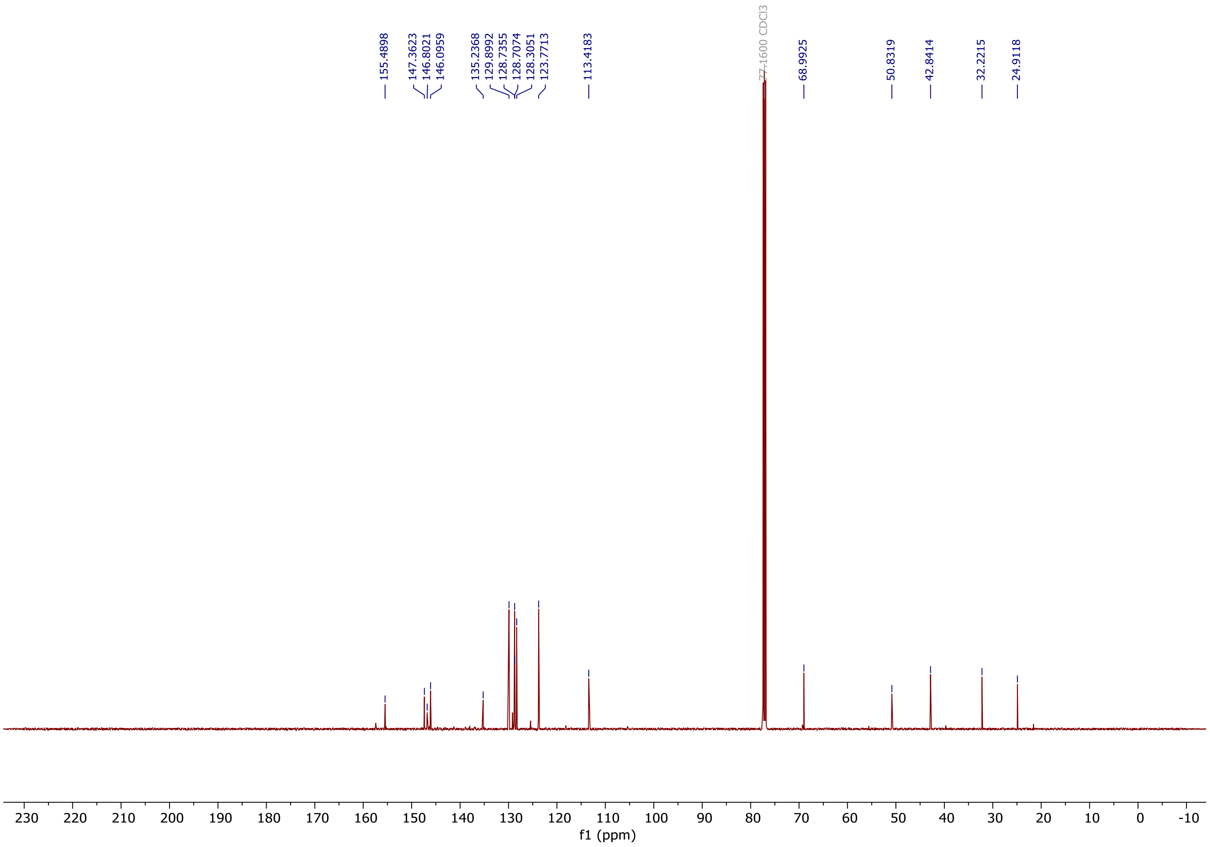

Benzyl 5-nitro-1,3-dihydrospiro[indene-2,2'-pyrrolidine]-1'-carboxylate (4c)

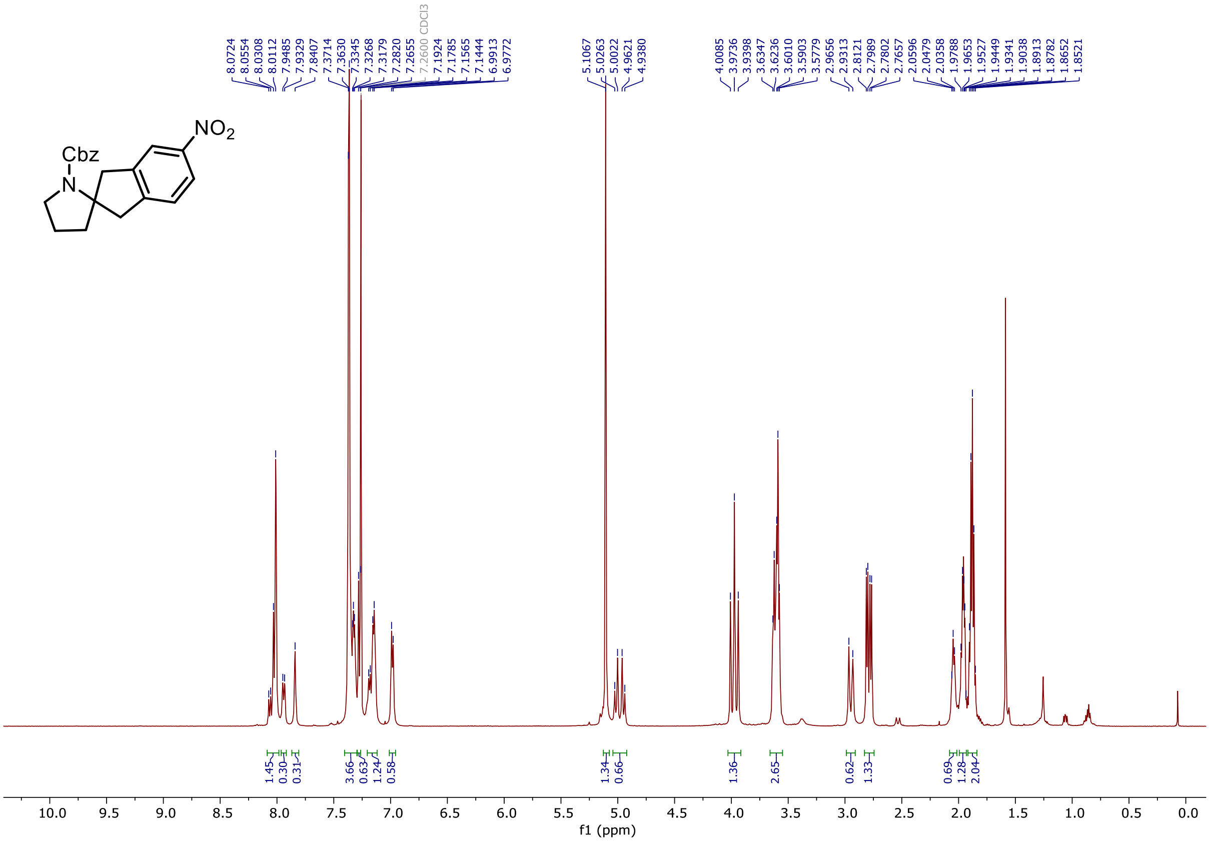

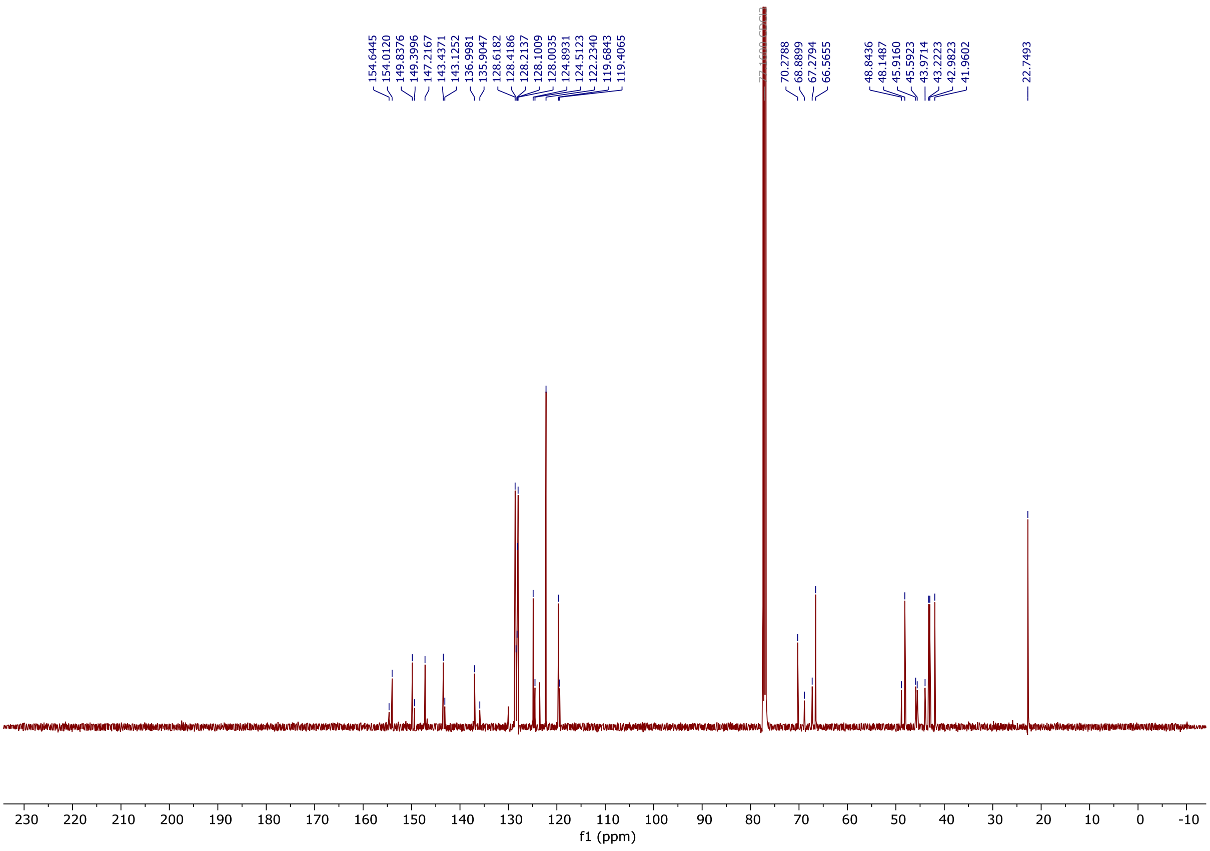

4-Benzyl-1-phenylpent-4-en-1-ol

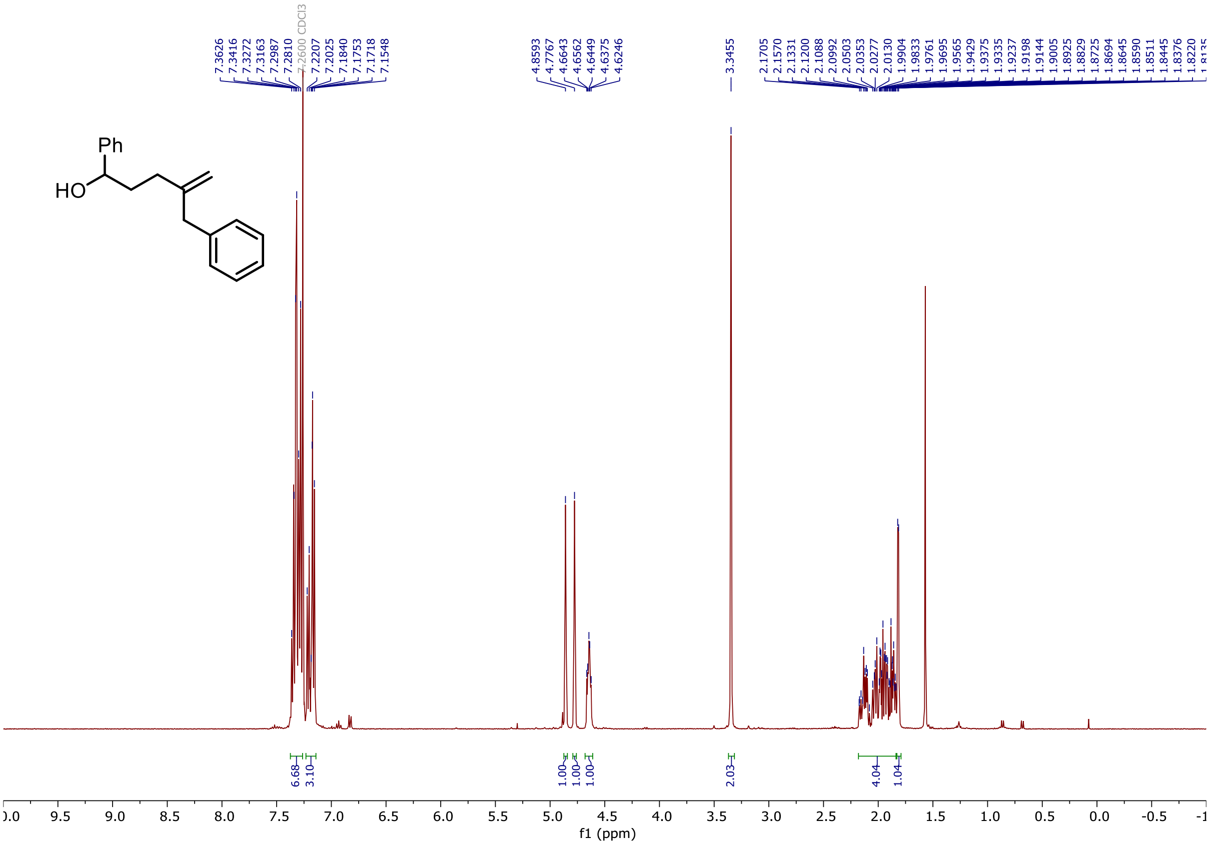

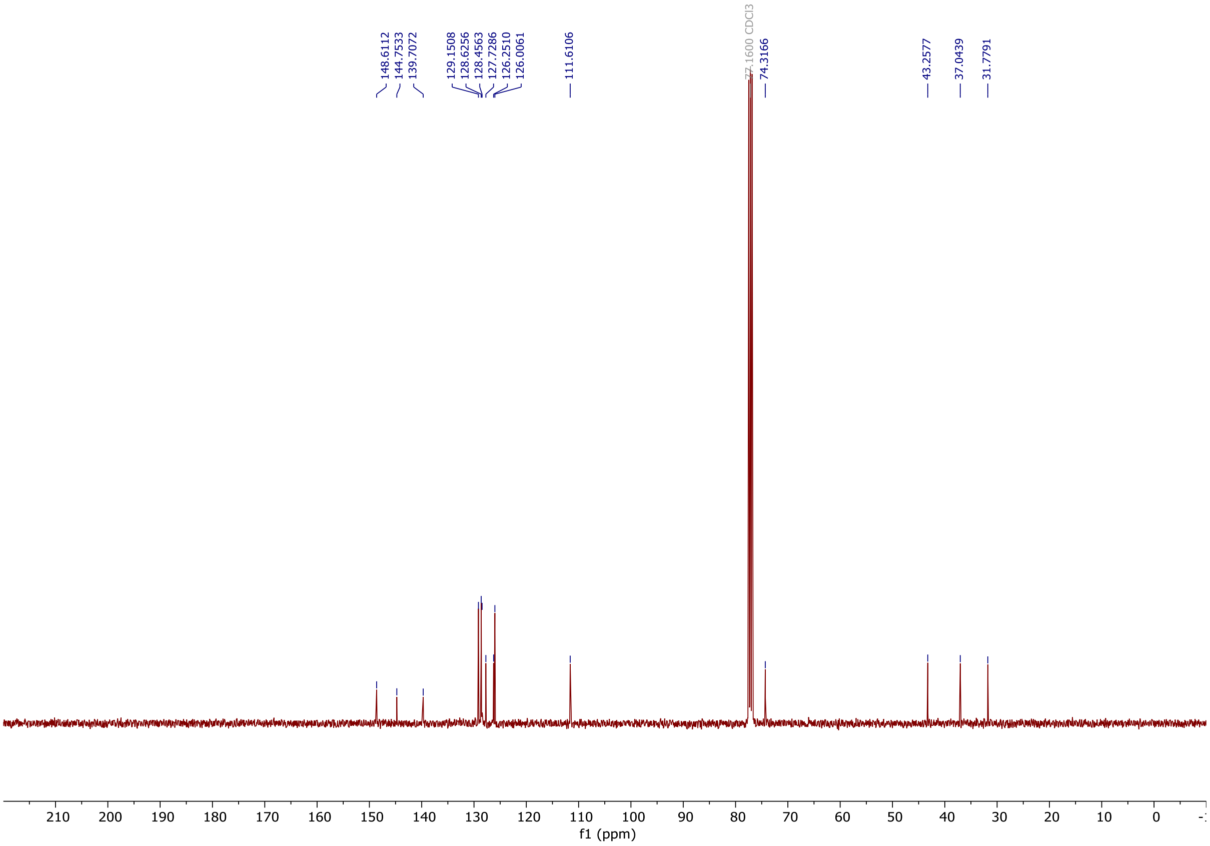

Benzyl (4-benzyl-1-phenylpent-4-en-1-yl)((perfluorobenzoyl)oxy)carbamate (3d)

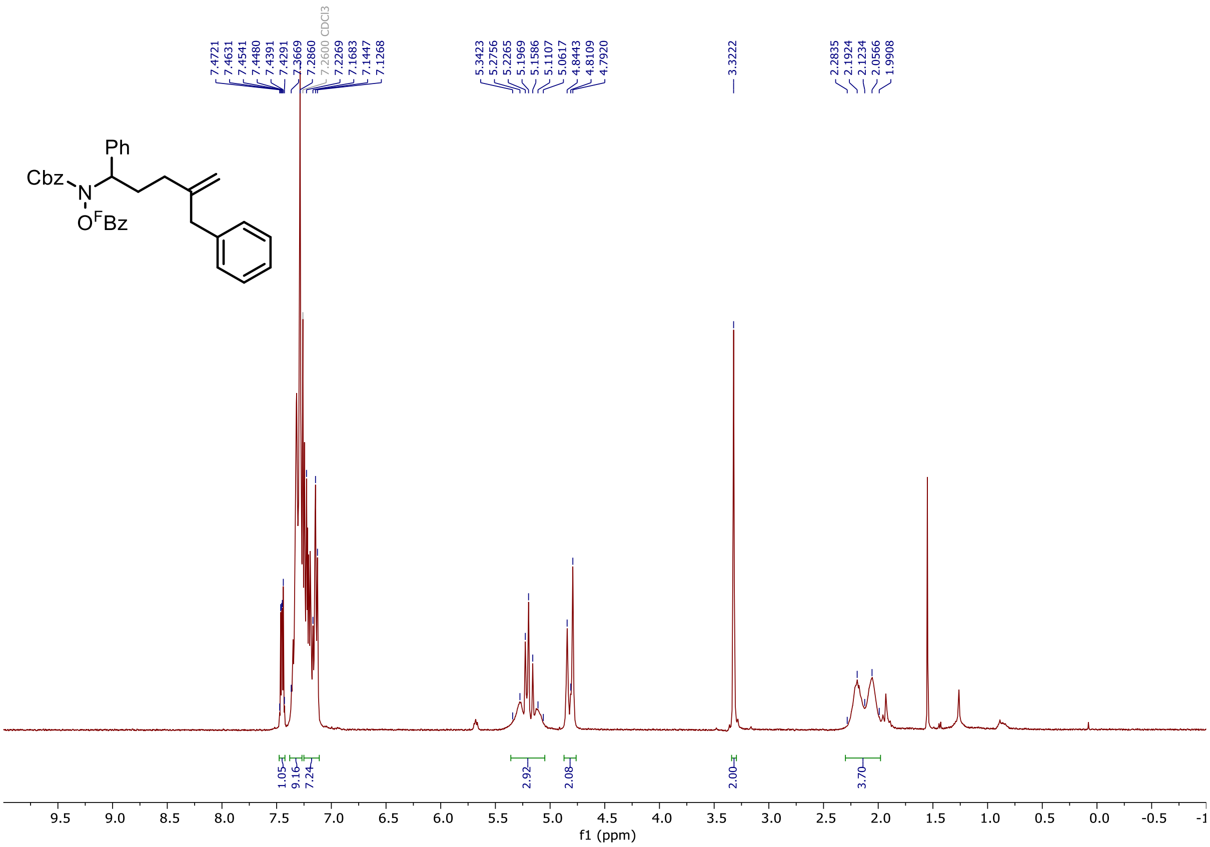

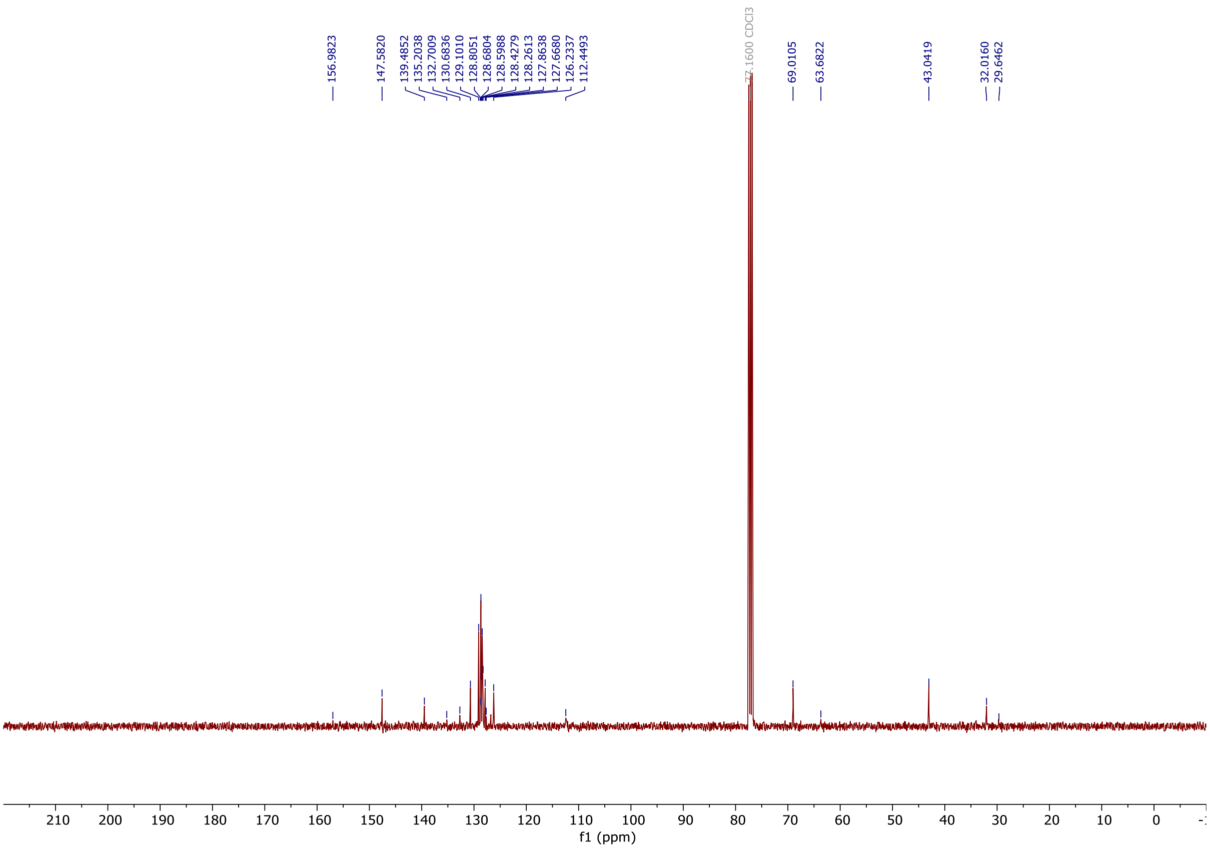

Benzyl 5'-phenyl-1,3-dihydrospiro[indene-2,2'-pyrrolidine]-1'-carboxylate (4d)

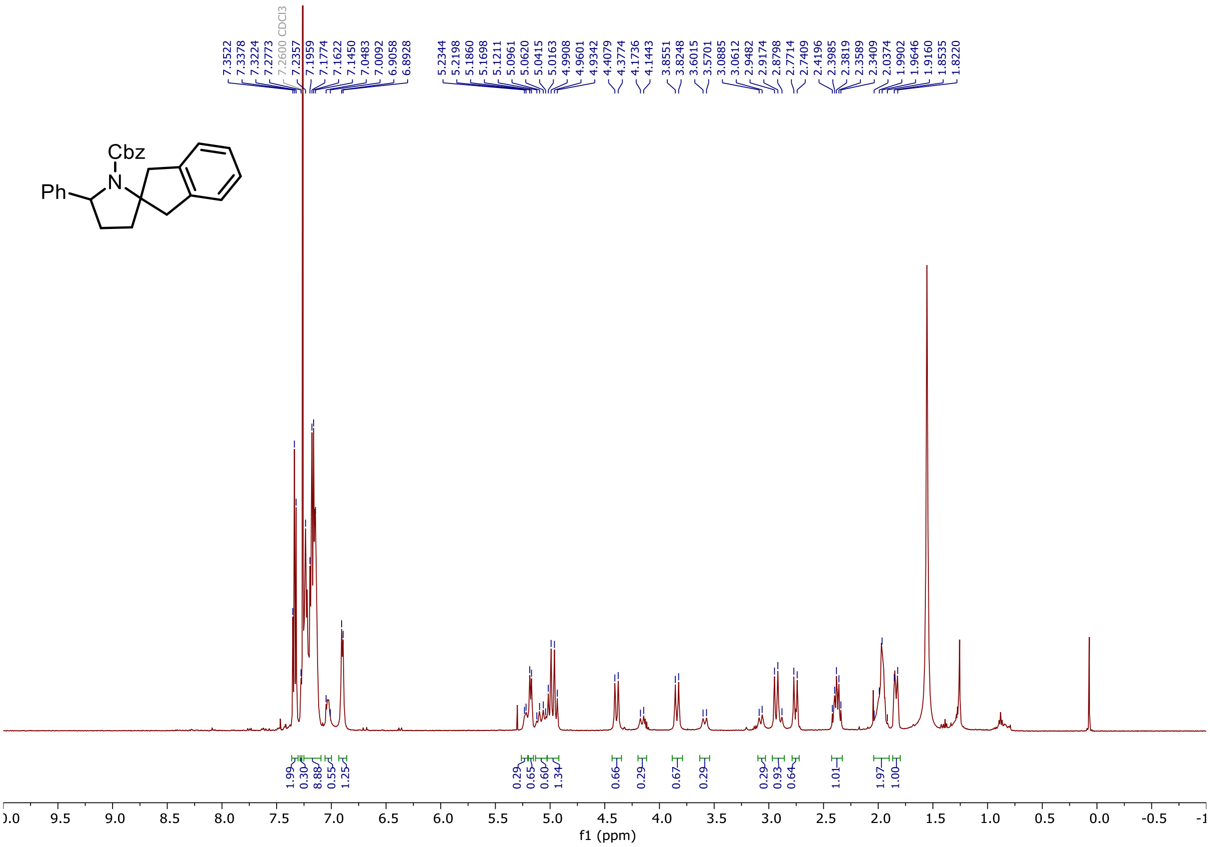

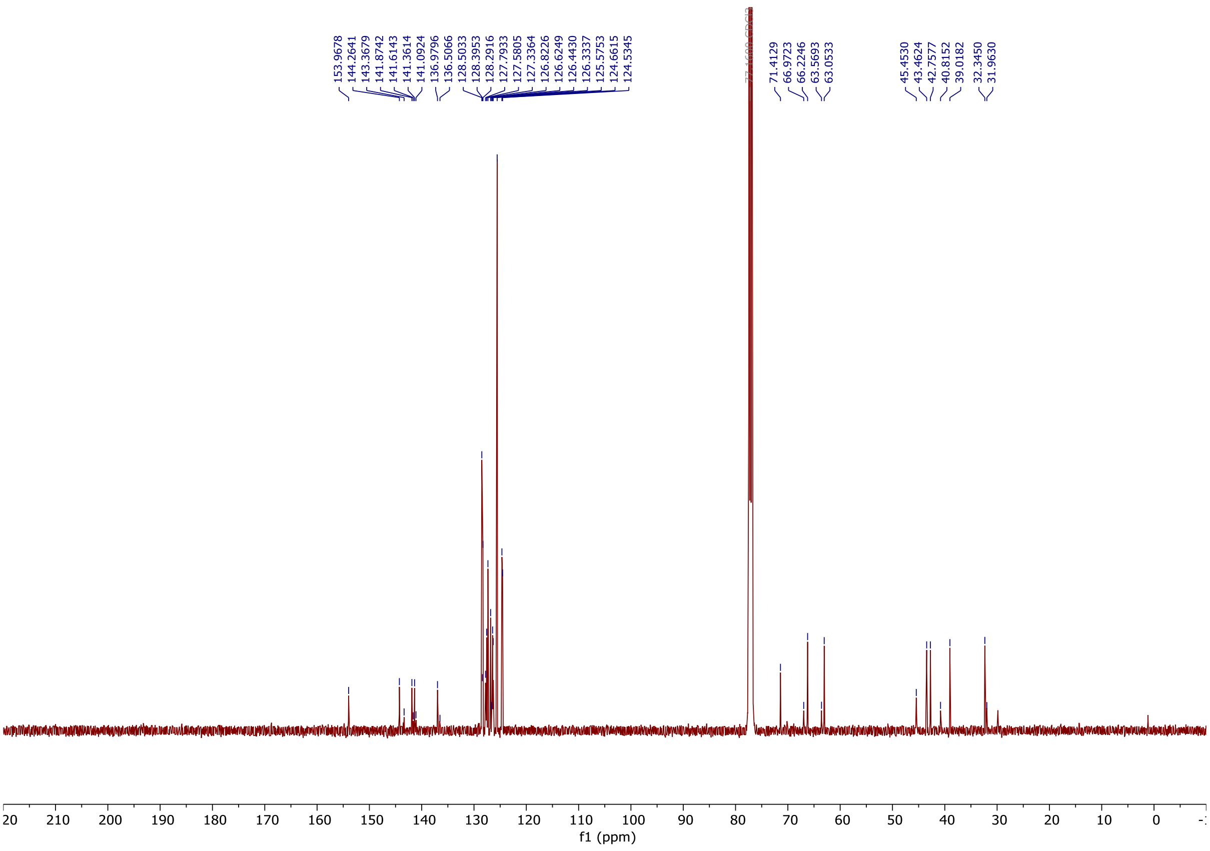

5-Benzylhex-5-en-2-ol

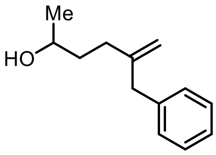

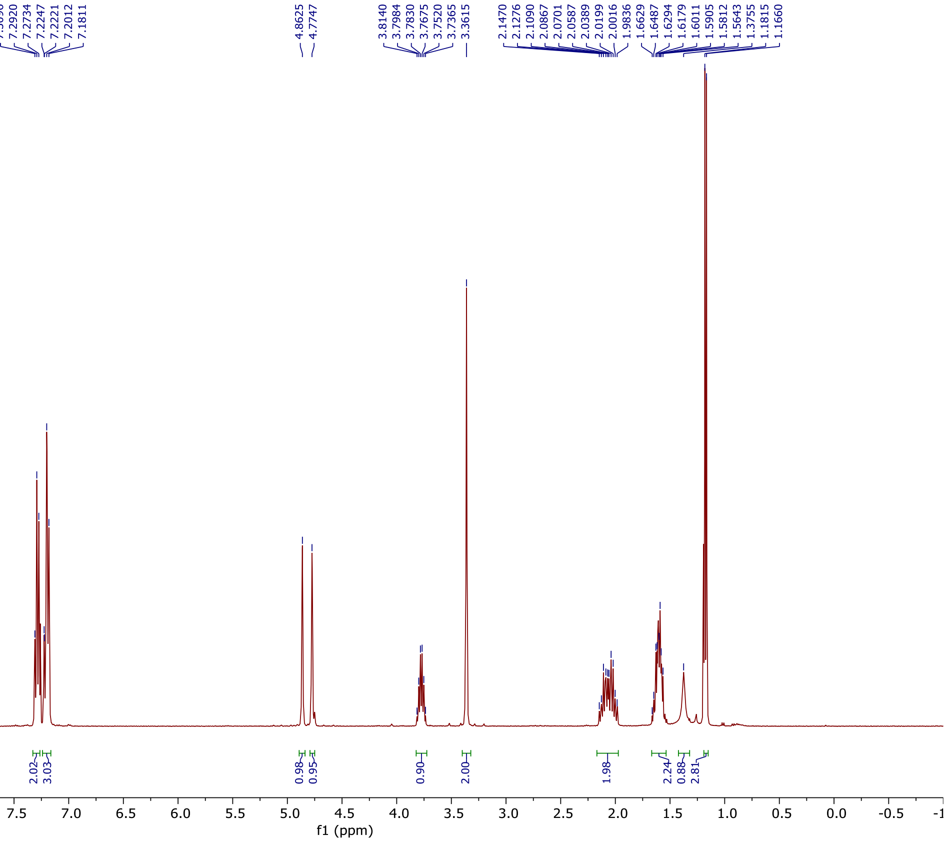

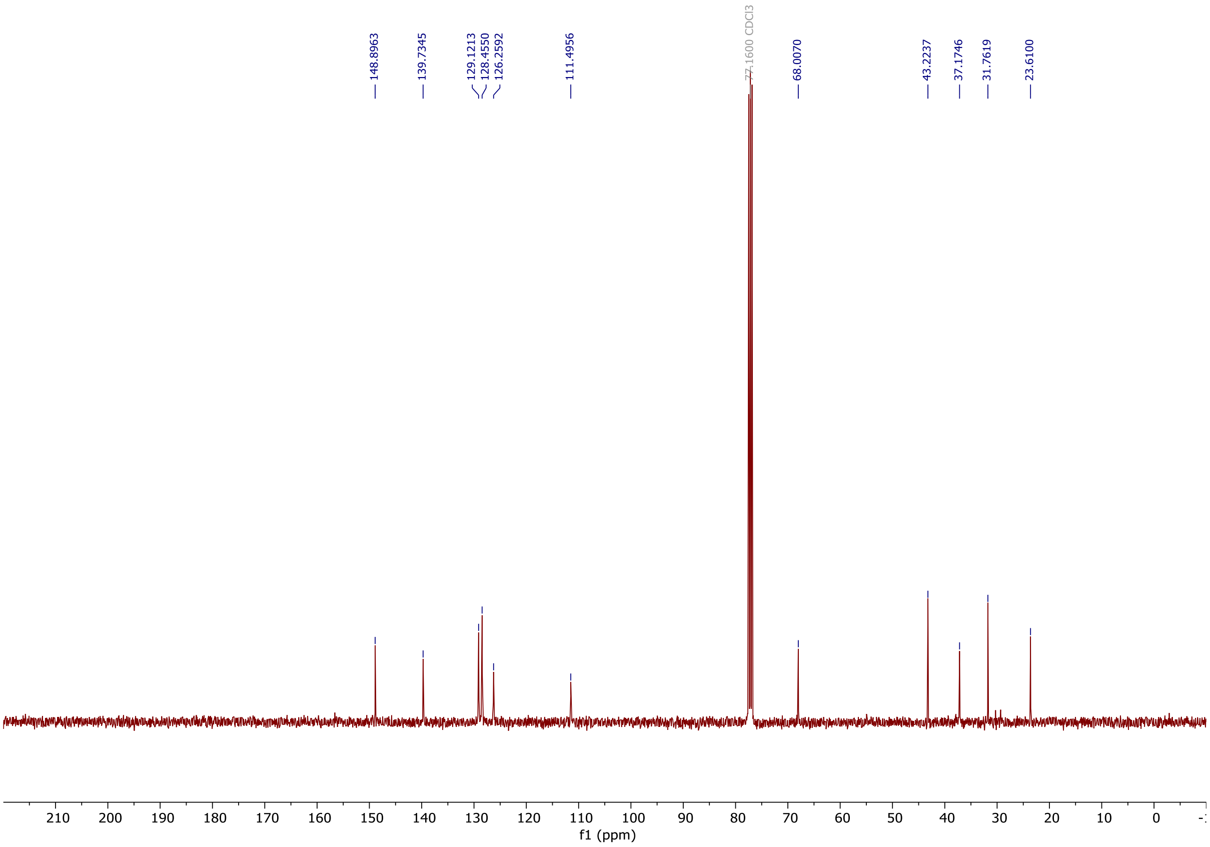

Benzyl (5-benzylhex-5-en-2-yl)((perfluorobenzoyl)oxy)carbamate (3e)

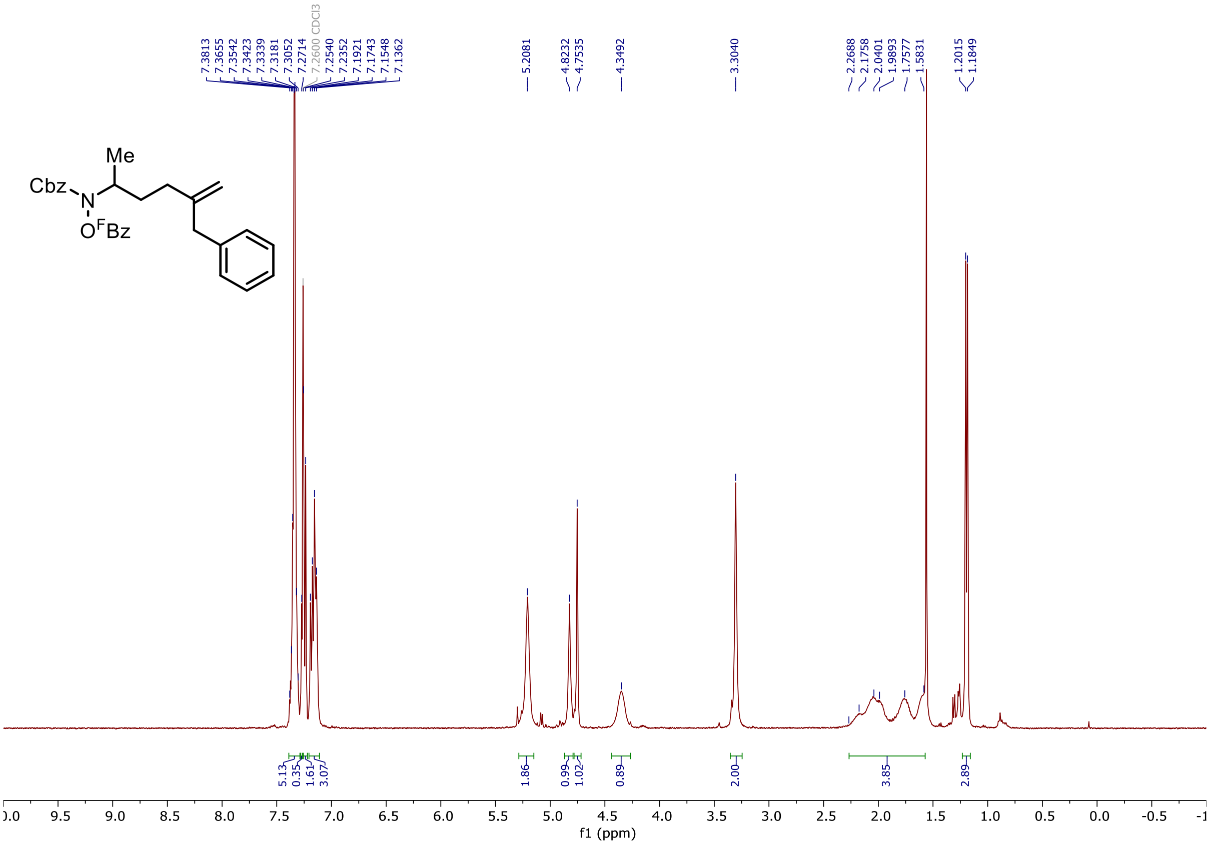

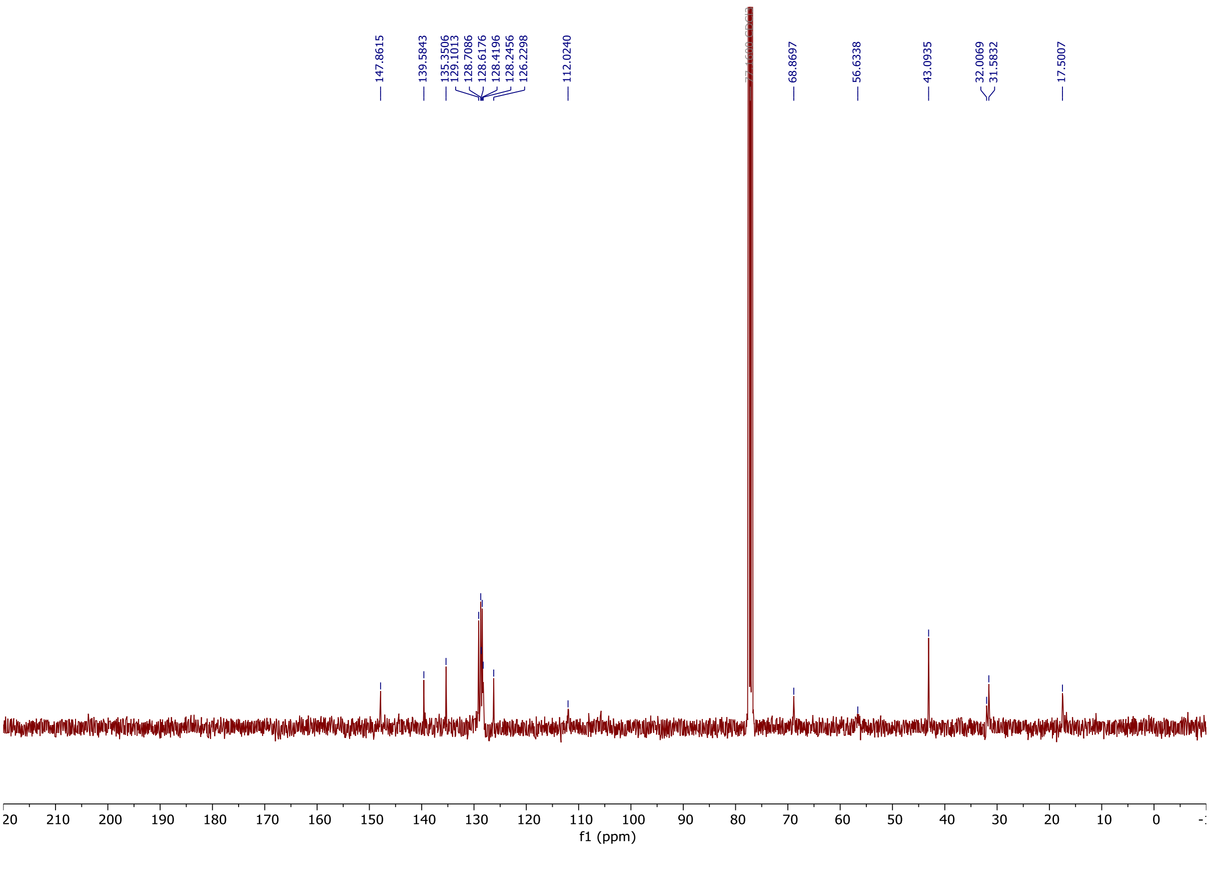

Benzyl 5'-methyl-1,3-dihydrospiro[indene-2,2'-pyrrolidine]-1'-carboxylate (4e)

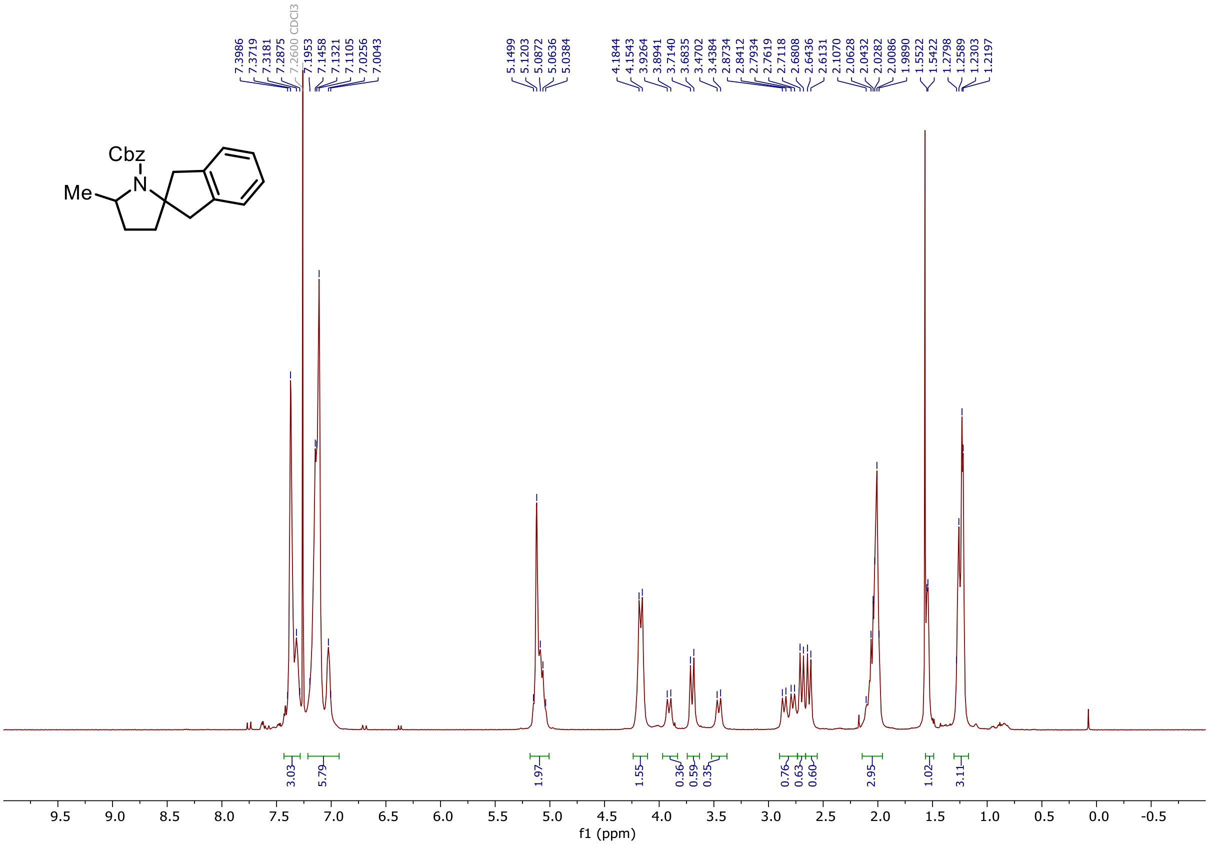

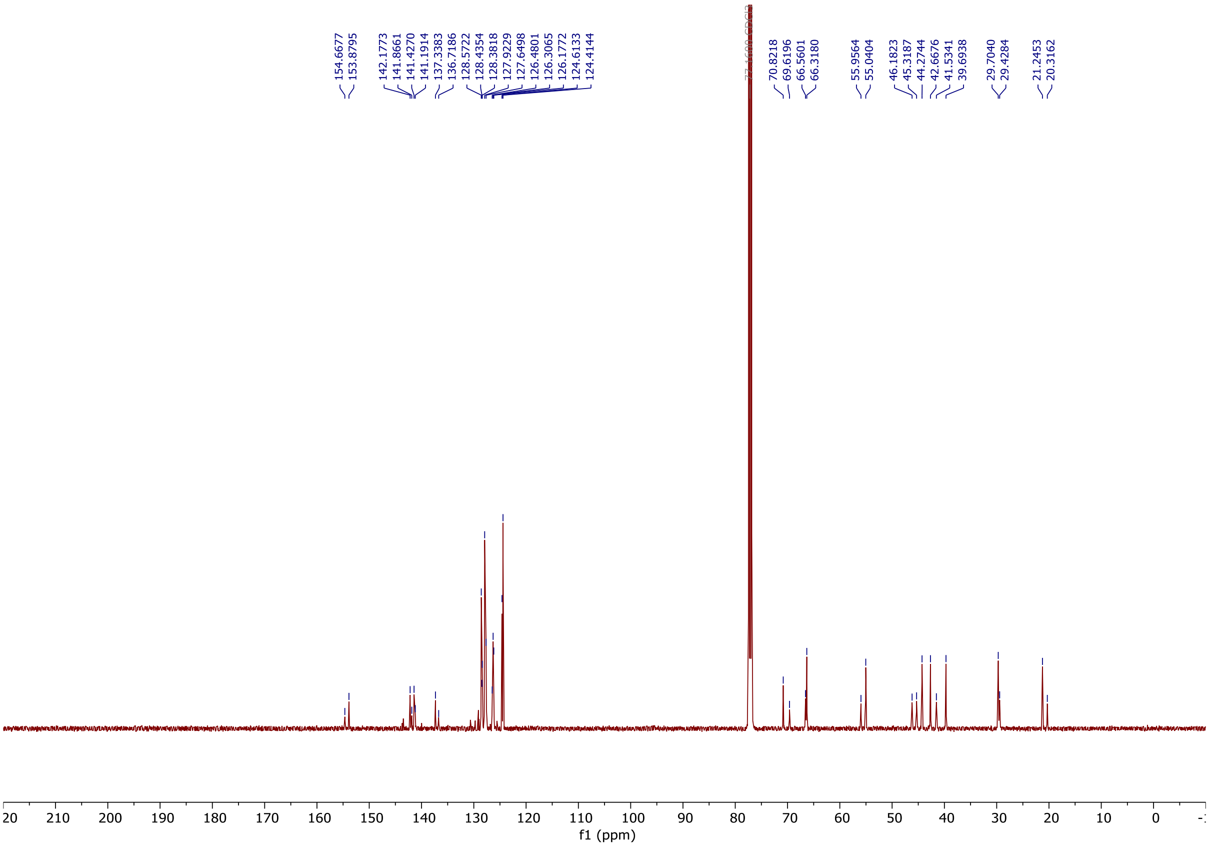

3-(Quinolin-3-yl)propanal

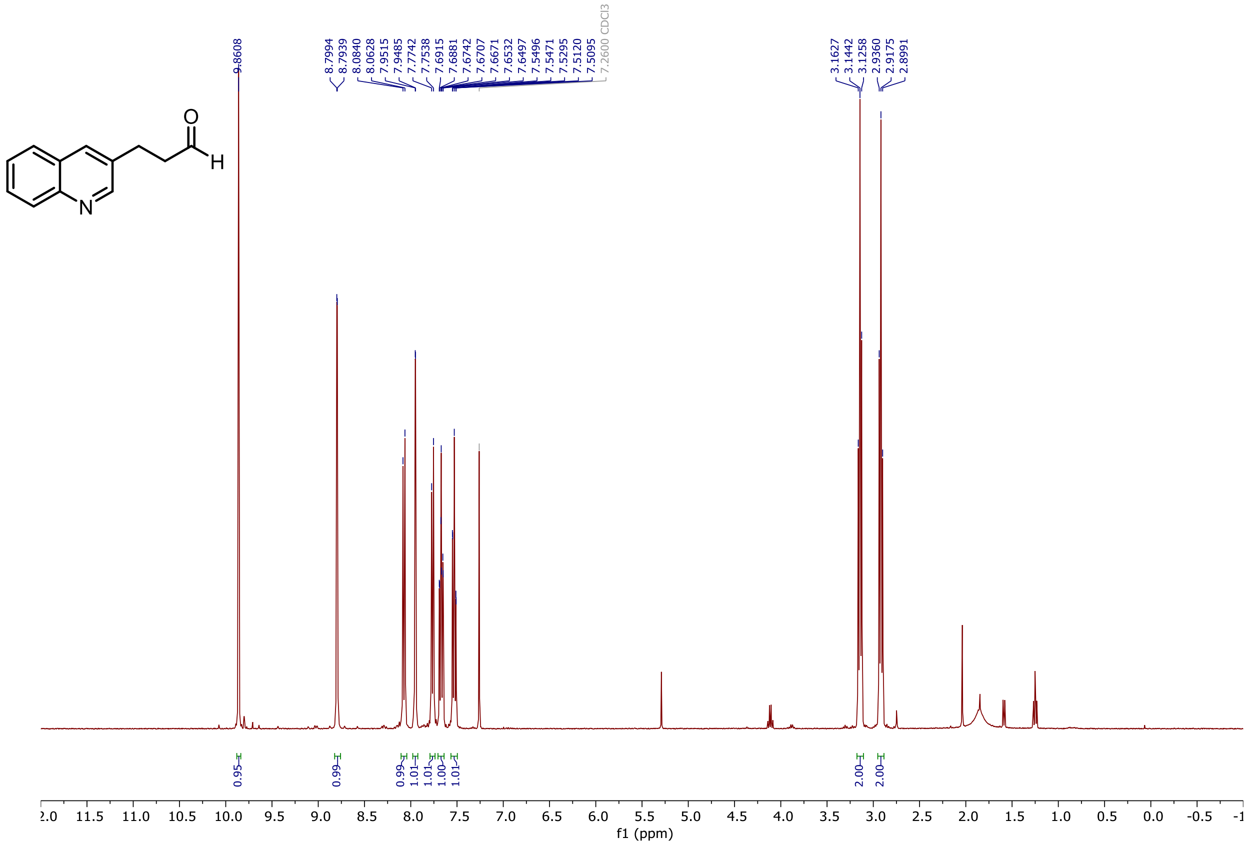

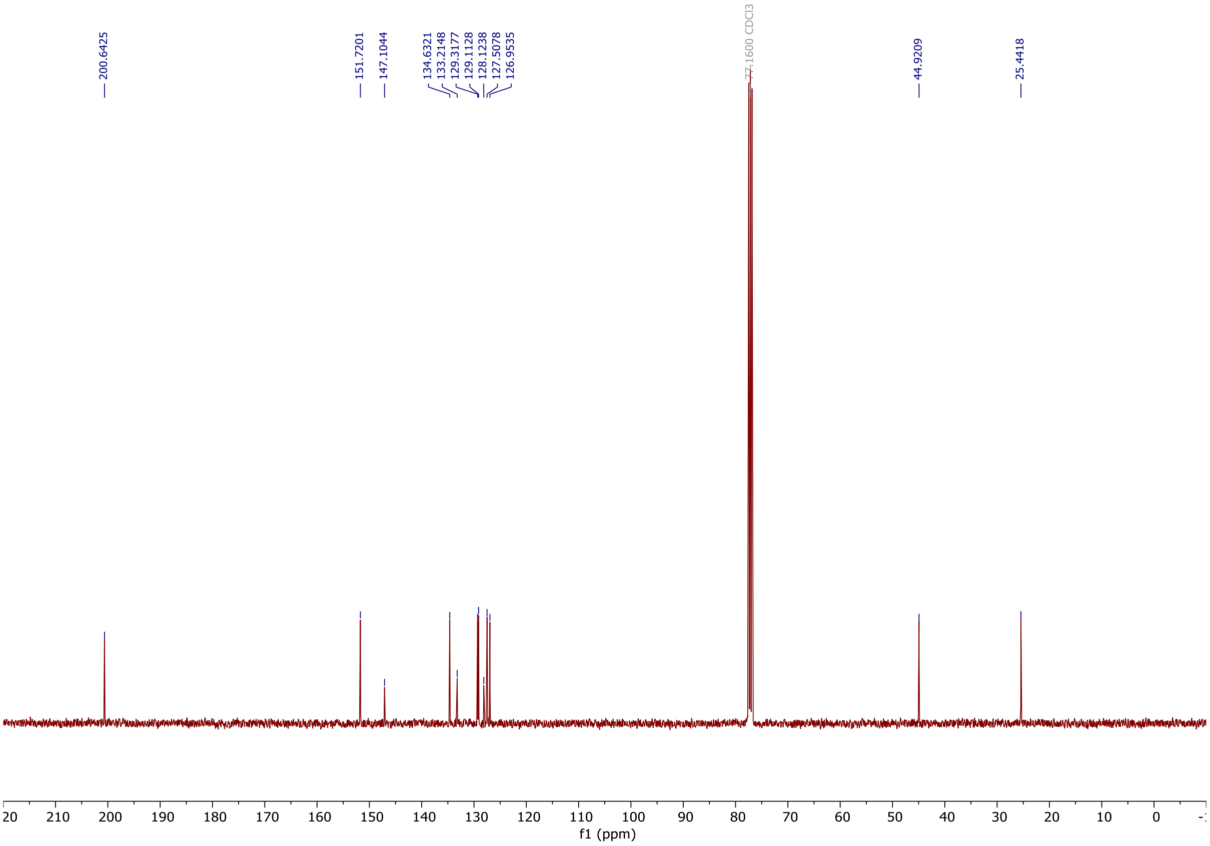

2-(Quinolin-3-ylmethyl)acrylaldehyde

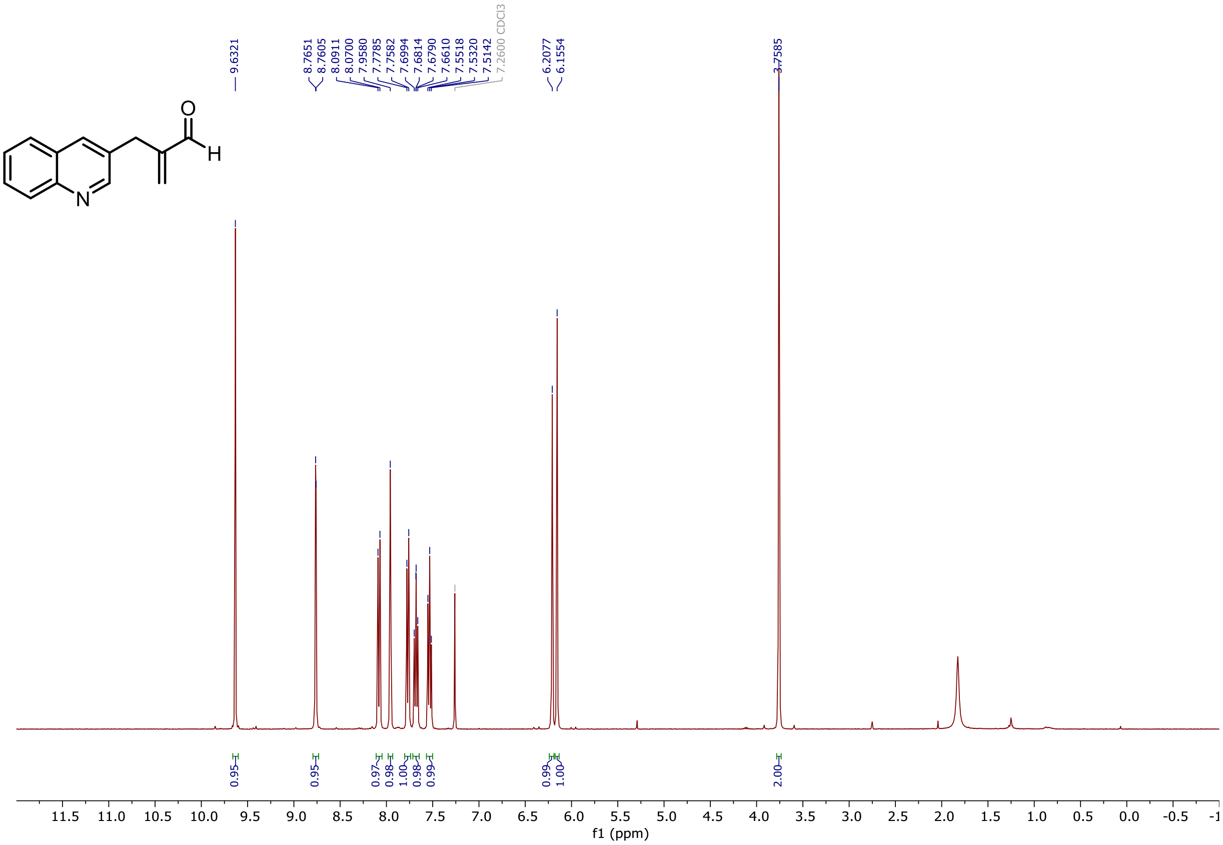

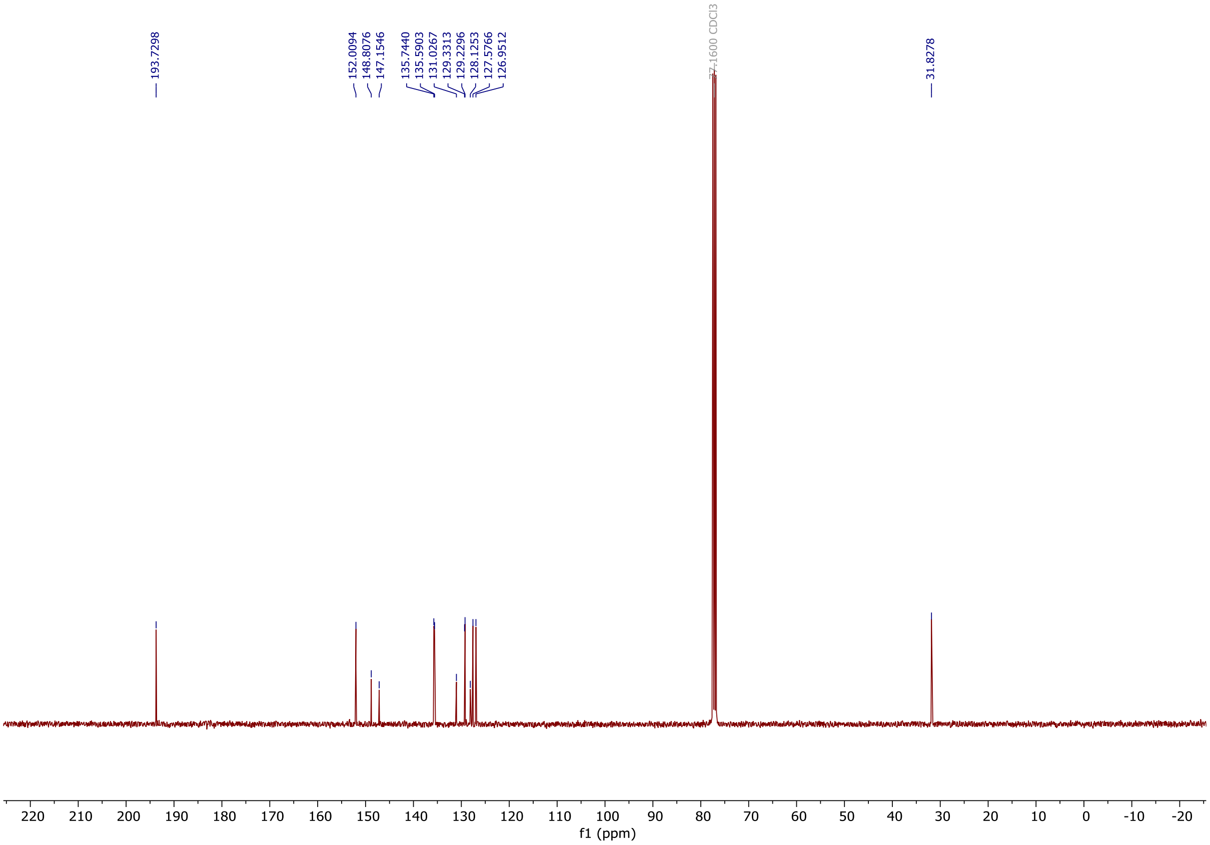

2-(Quinolin-3-ylmethyl)prop-2-en-1-ol

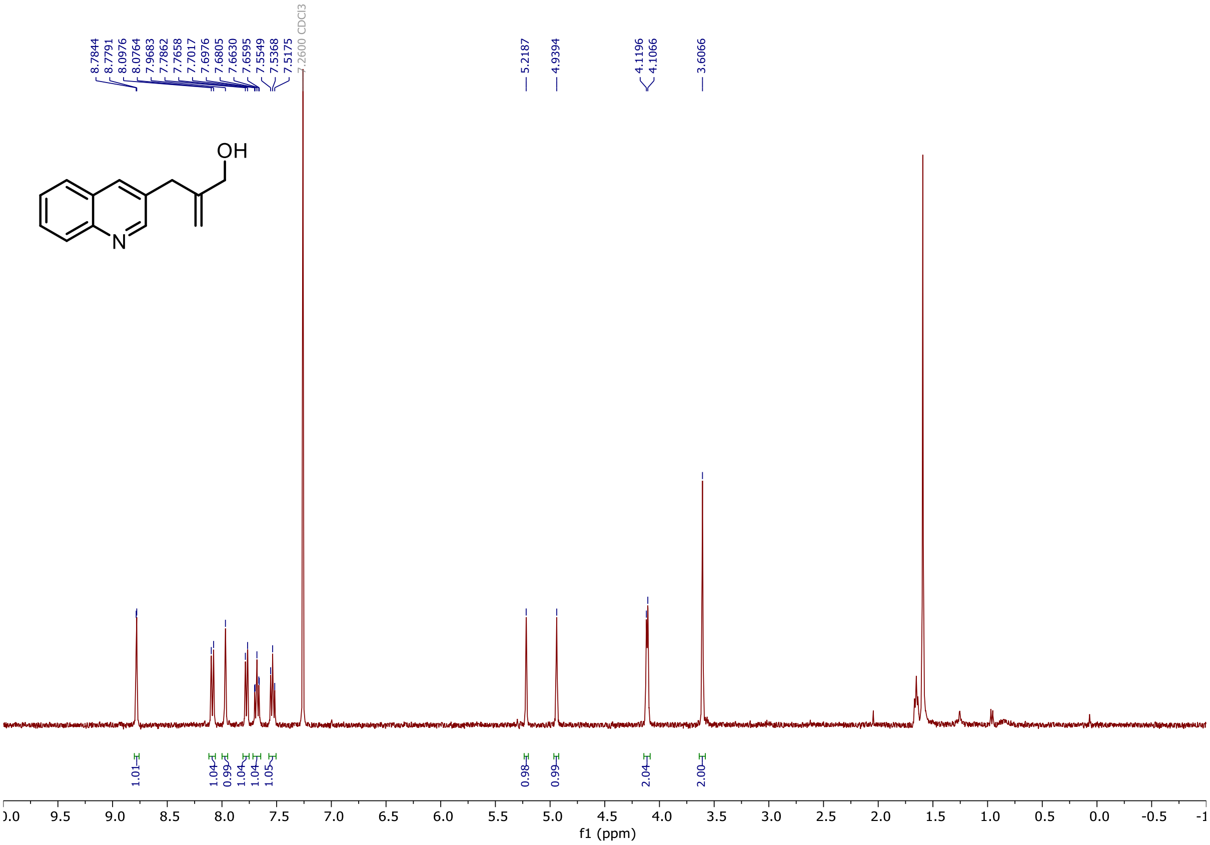

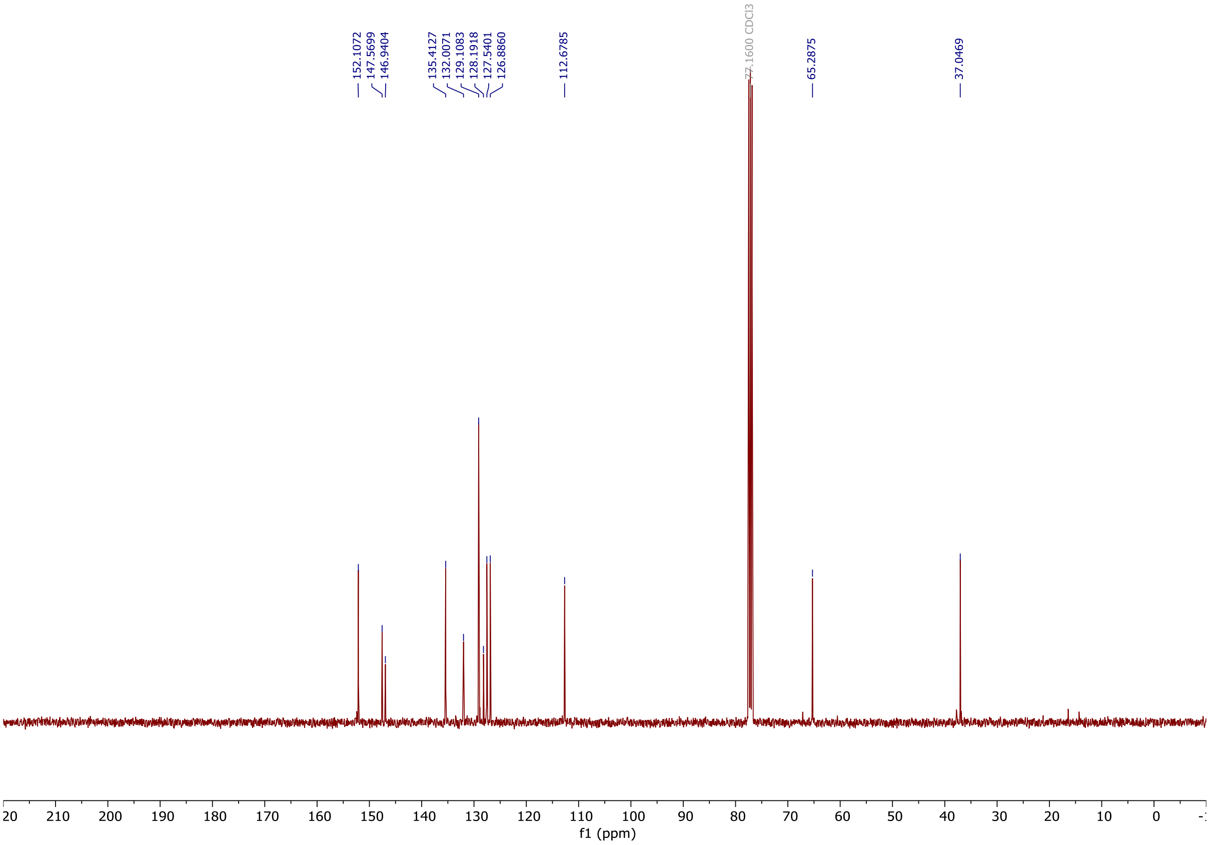

Ethyl 4-(quinolin-3-ylmethyl)pent-4-enoate

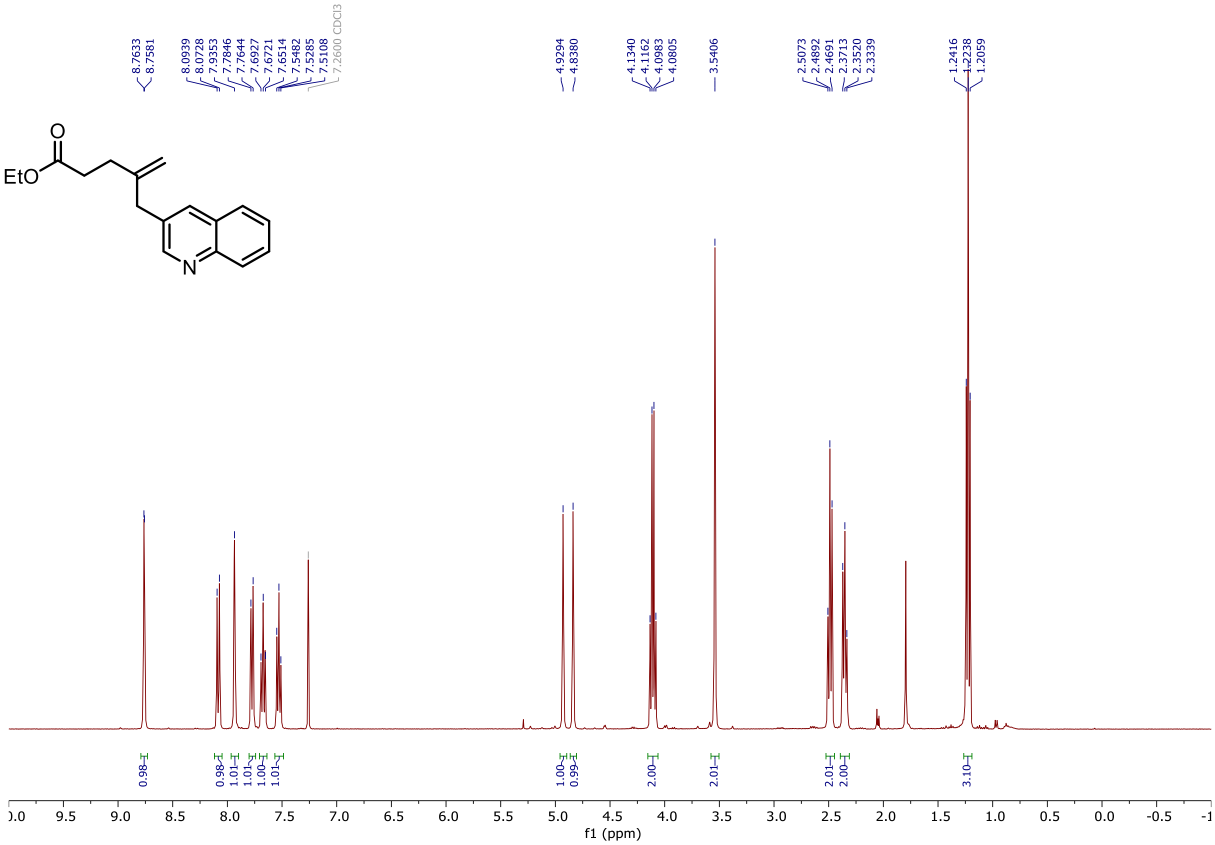

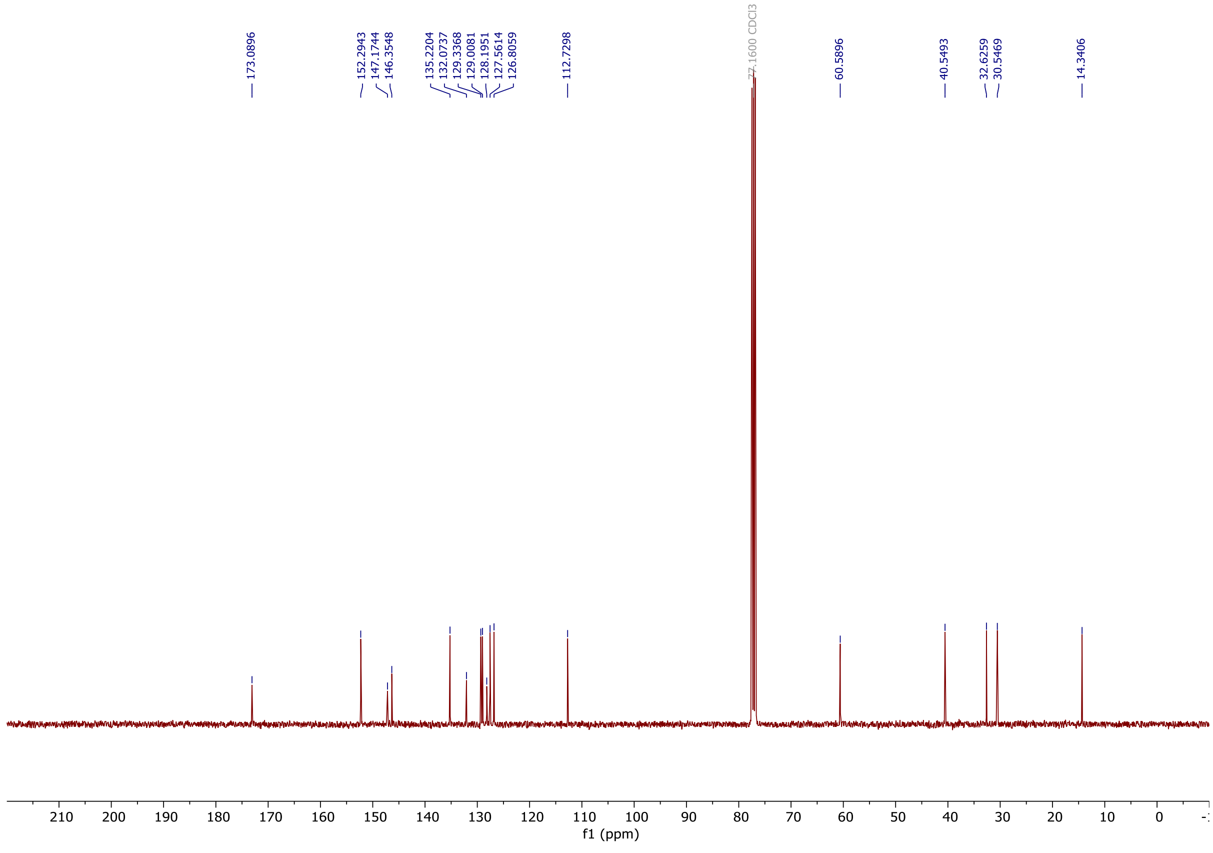

4-(Quinolin-3-ylmethyl)pent-4-en-1-ol

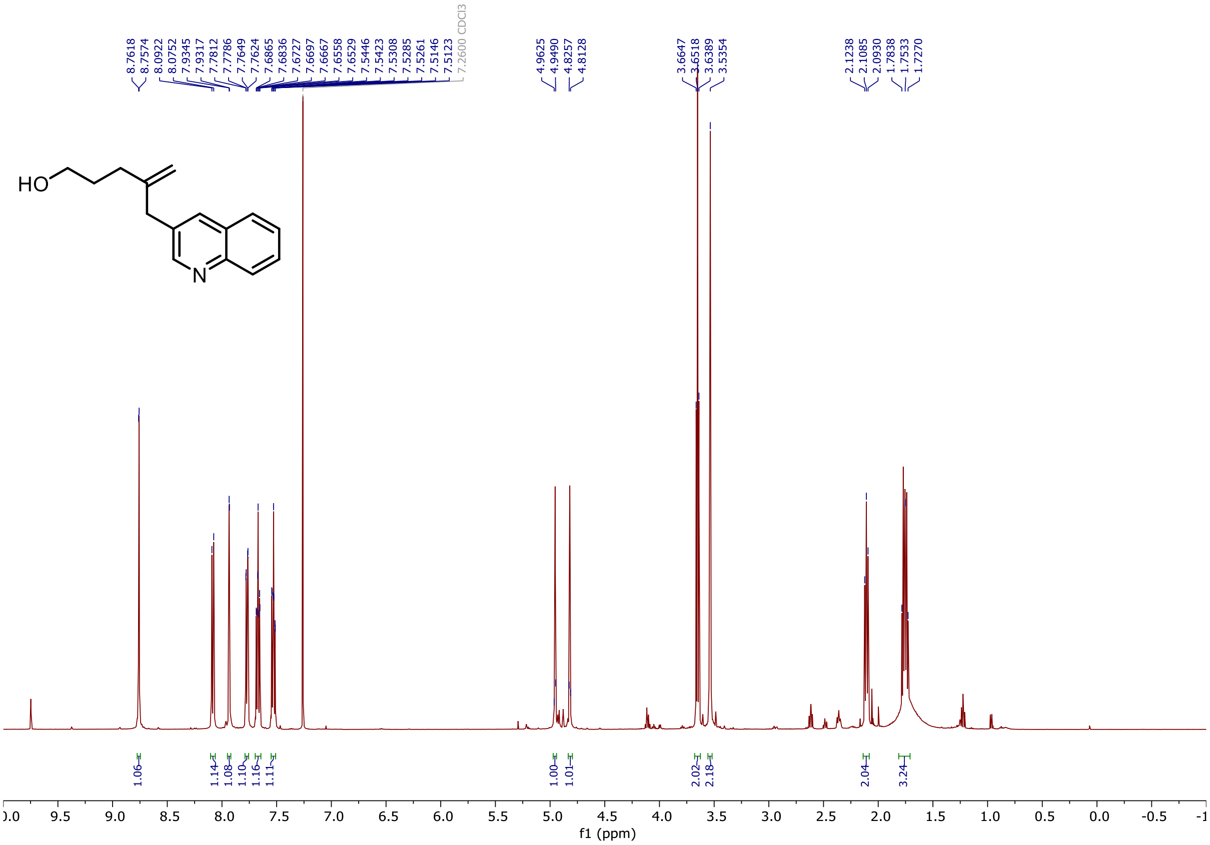

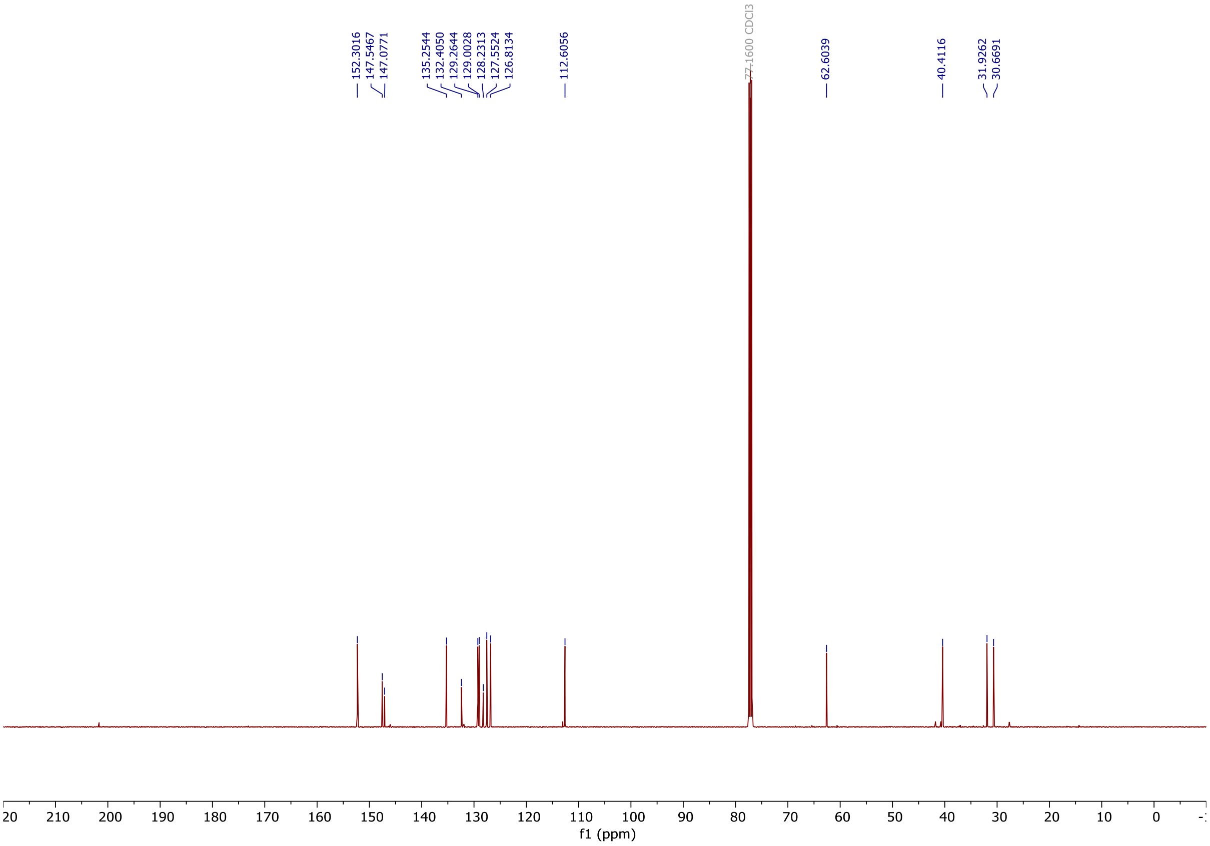

Benzyl ((perfluorobenzoyl)oxy)(4-(quinolin-3-ylmethyl)pent-4-en-1-yl)carbamate (3g)

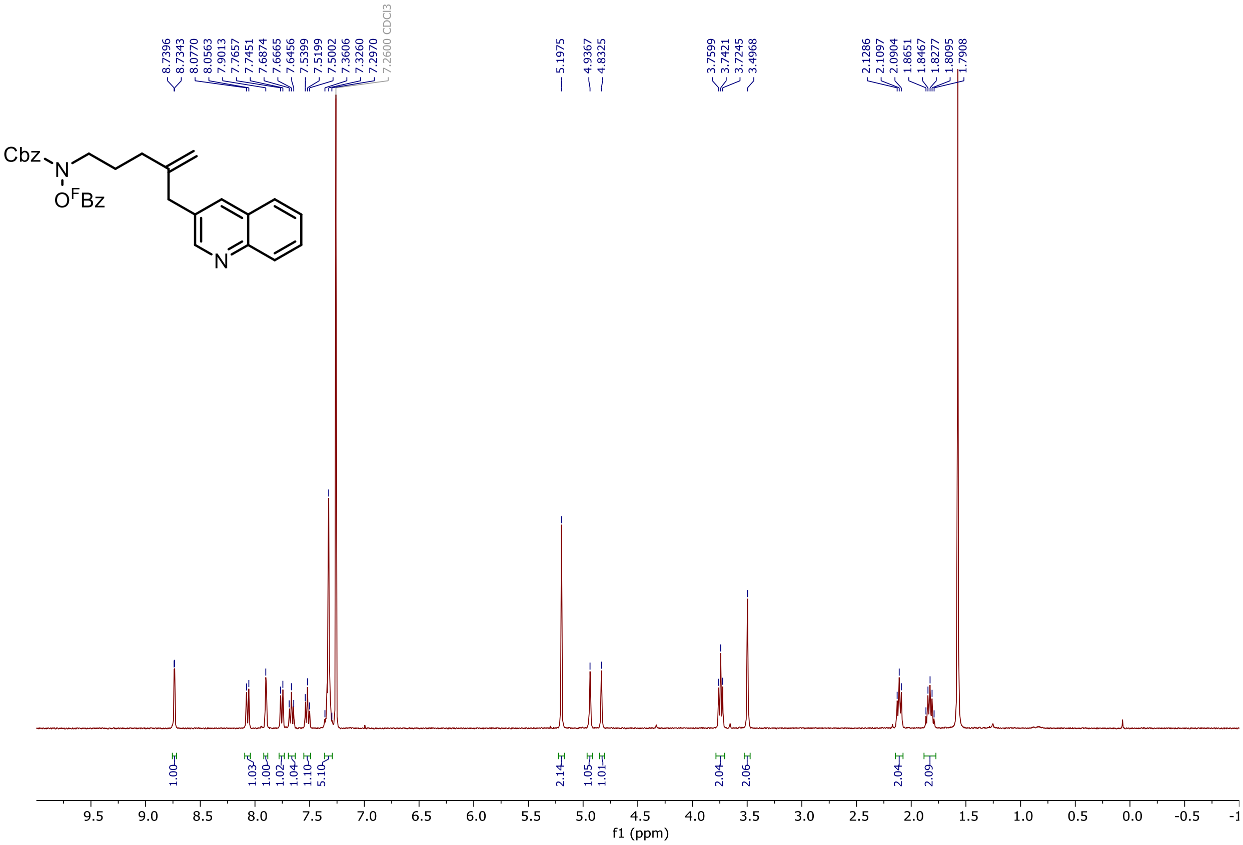

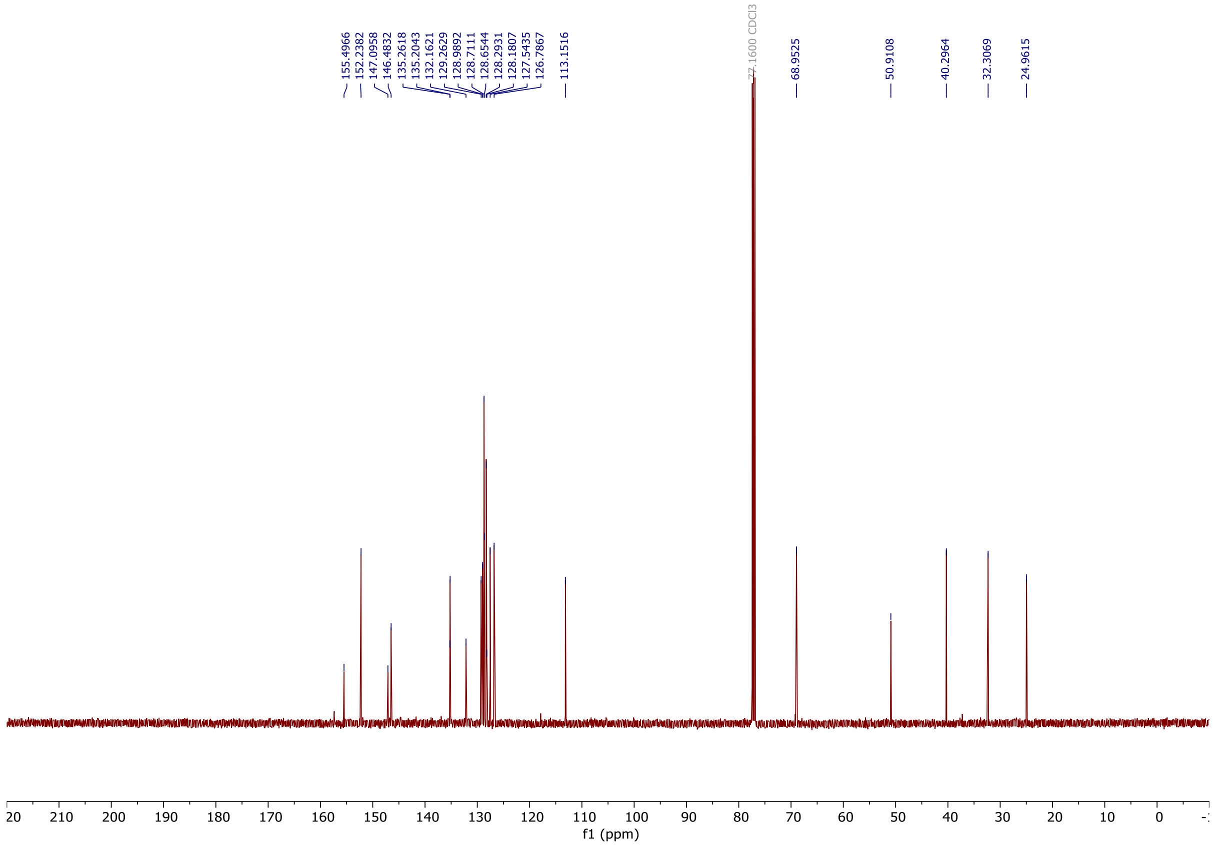

Benzyl 1,3-dihydrospiro[cyclopenta[*c*]quinoline-2,2'-pyrrolidine]-1'-carboxylate (4g)

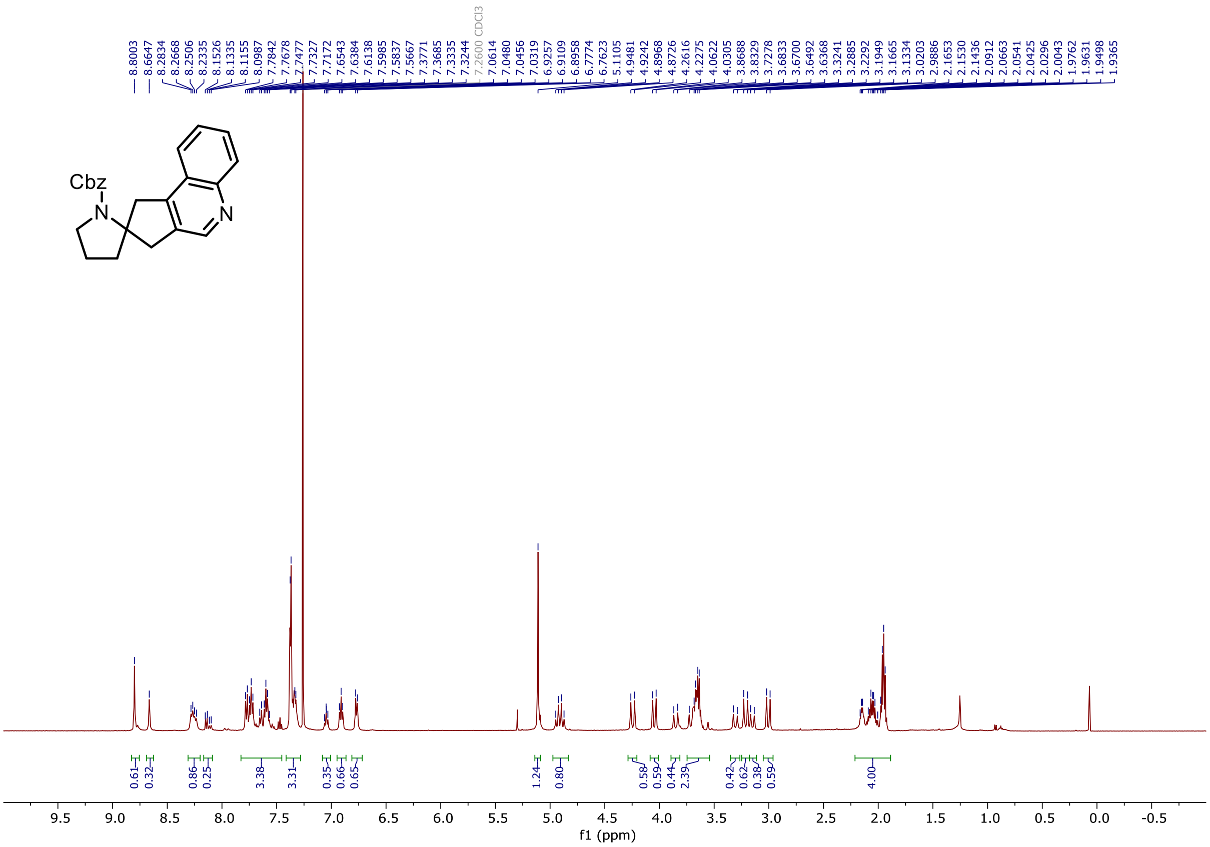

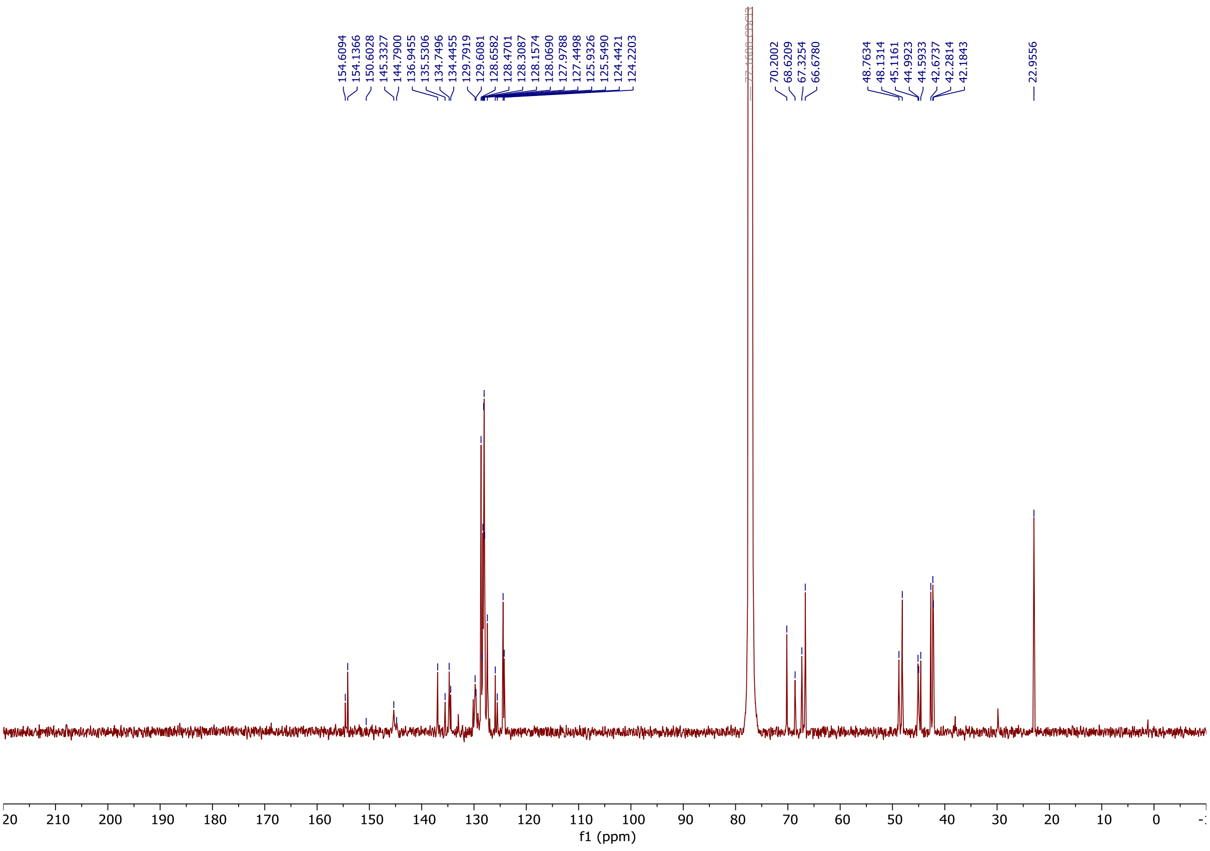

2-(Furan-3-ylmethyl)acrylaldehyde

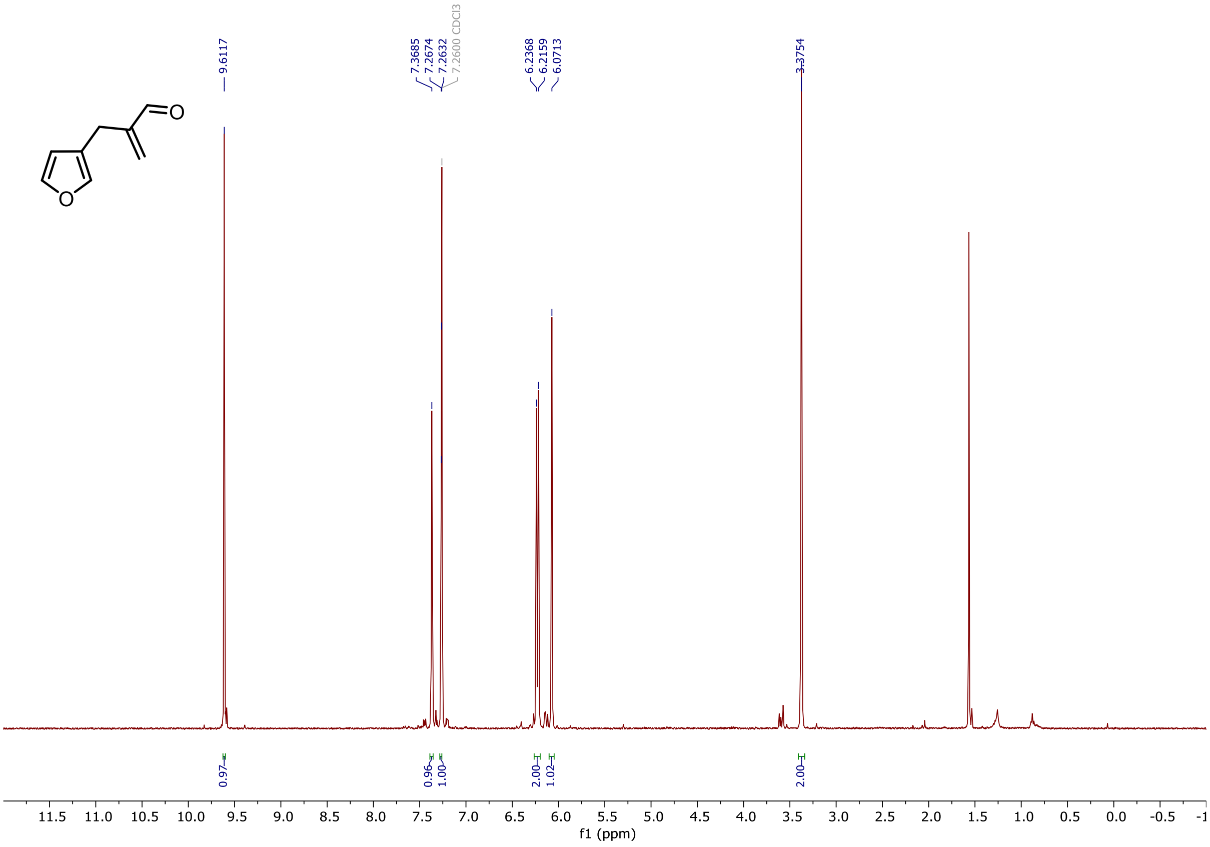

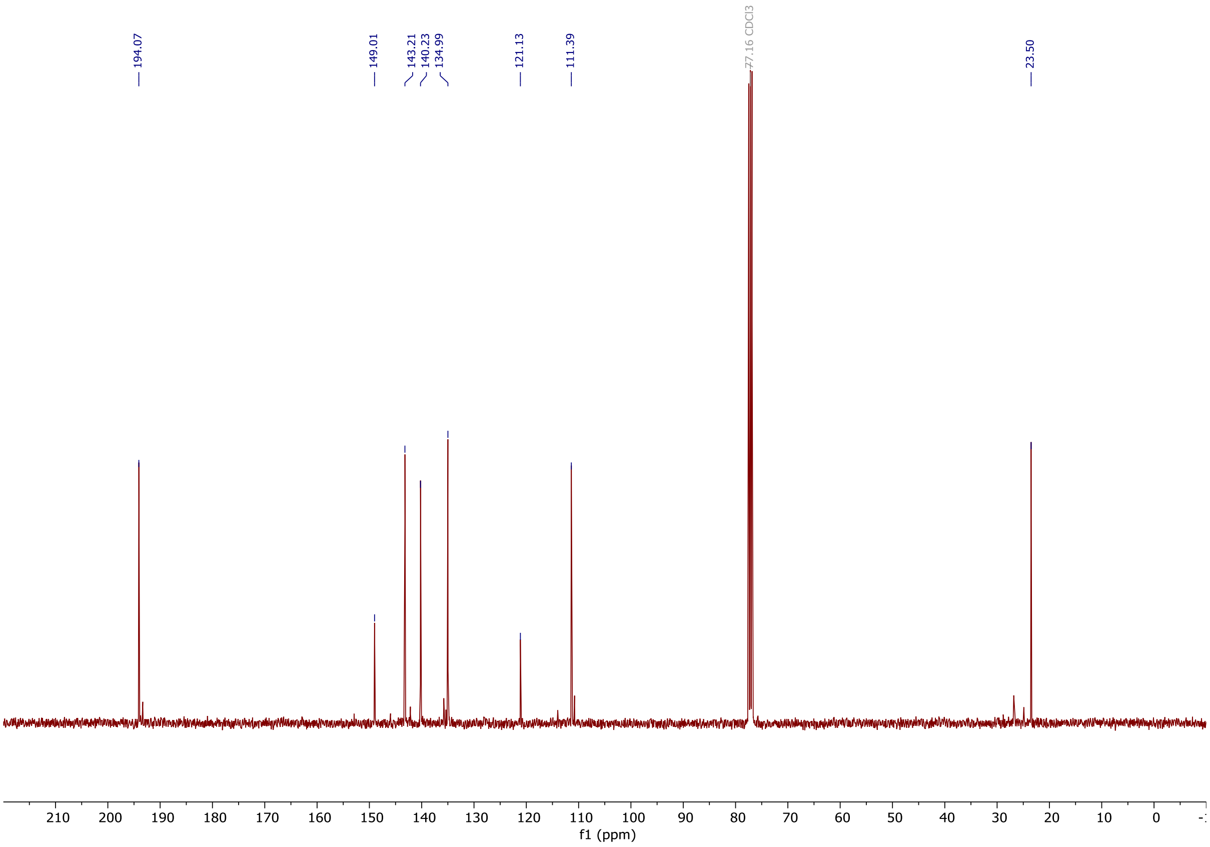

2-(Furan-3-ylmethyl)prop-2-en-1-ol

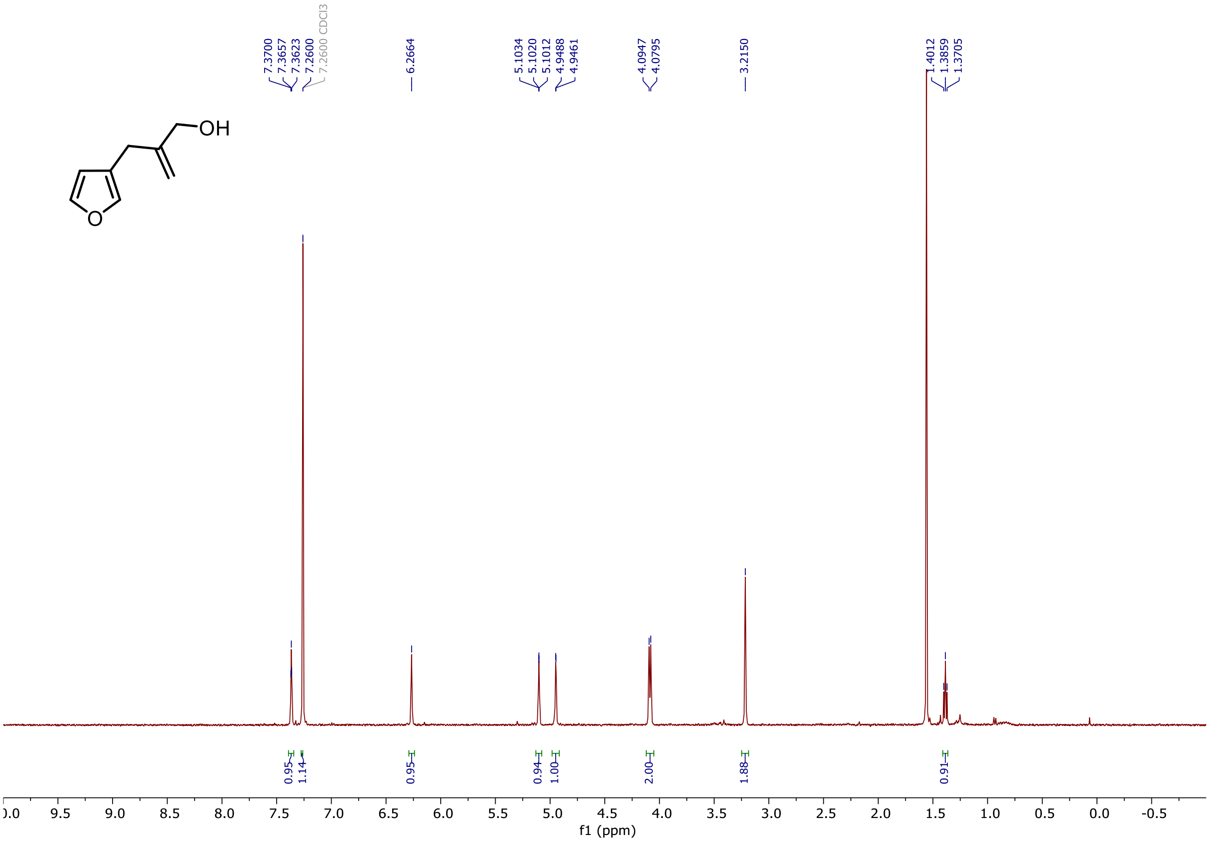

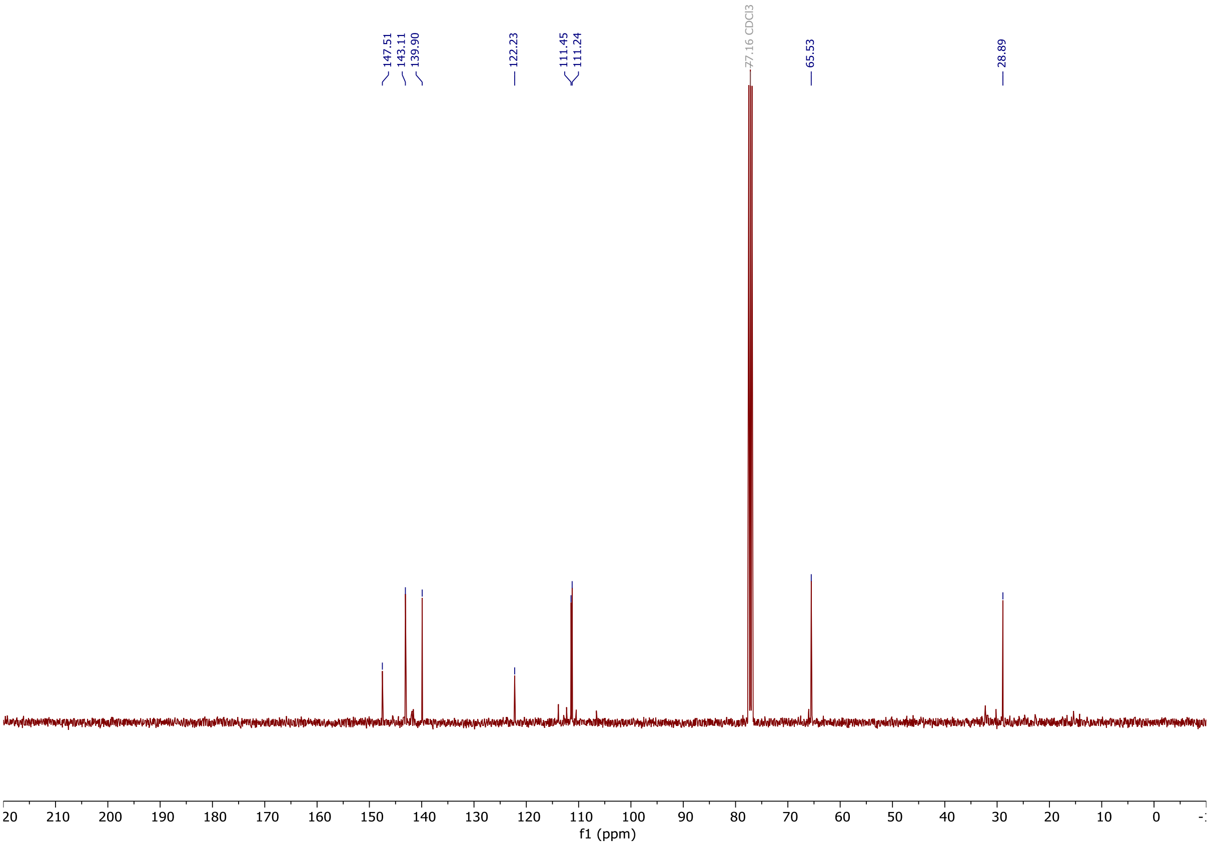

Ethyl 4-(furan-3-ylmethyl)pent-4-enoate

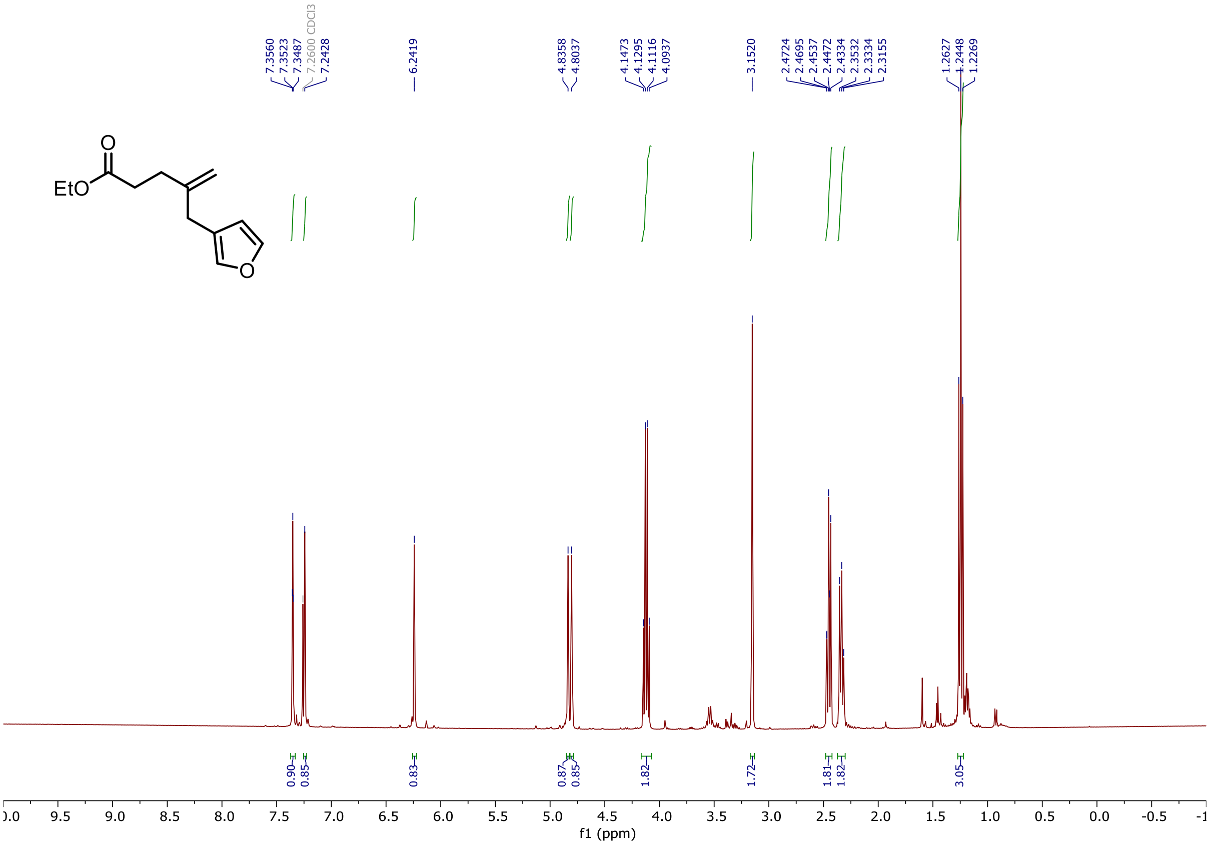

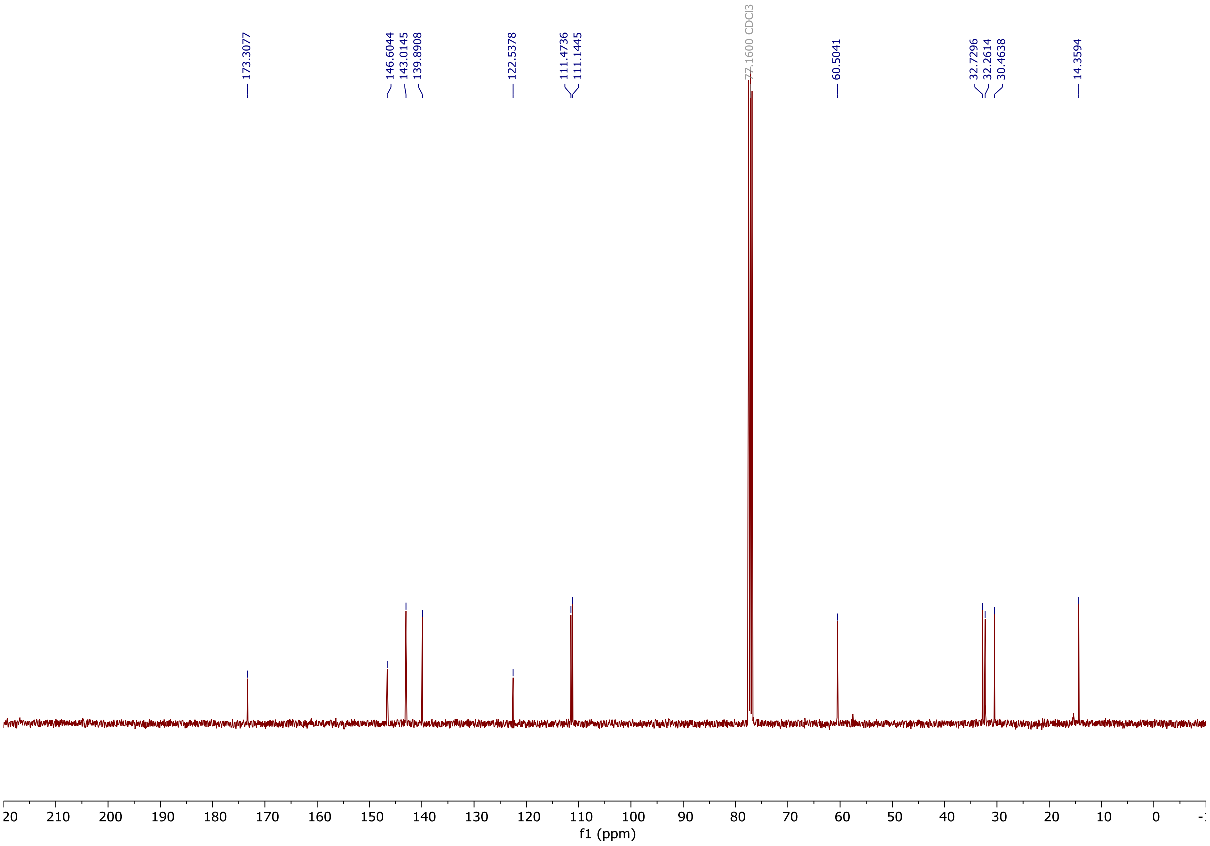

4-(Furan-3-ylmethyl)pent-4-en-1-ol

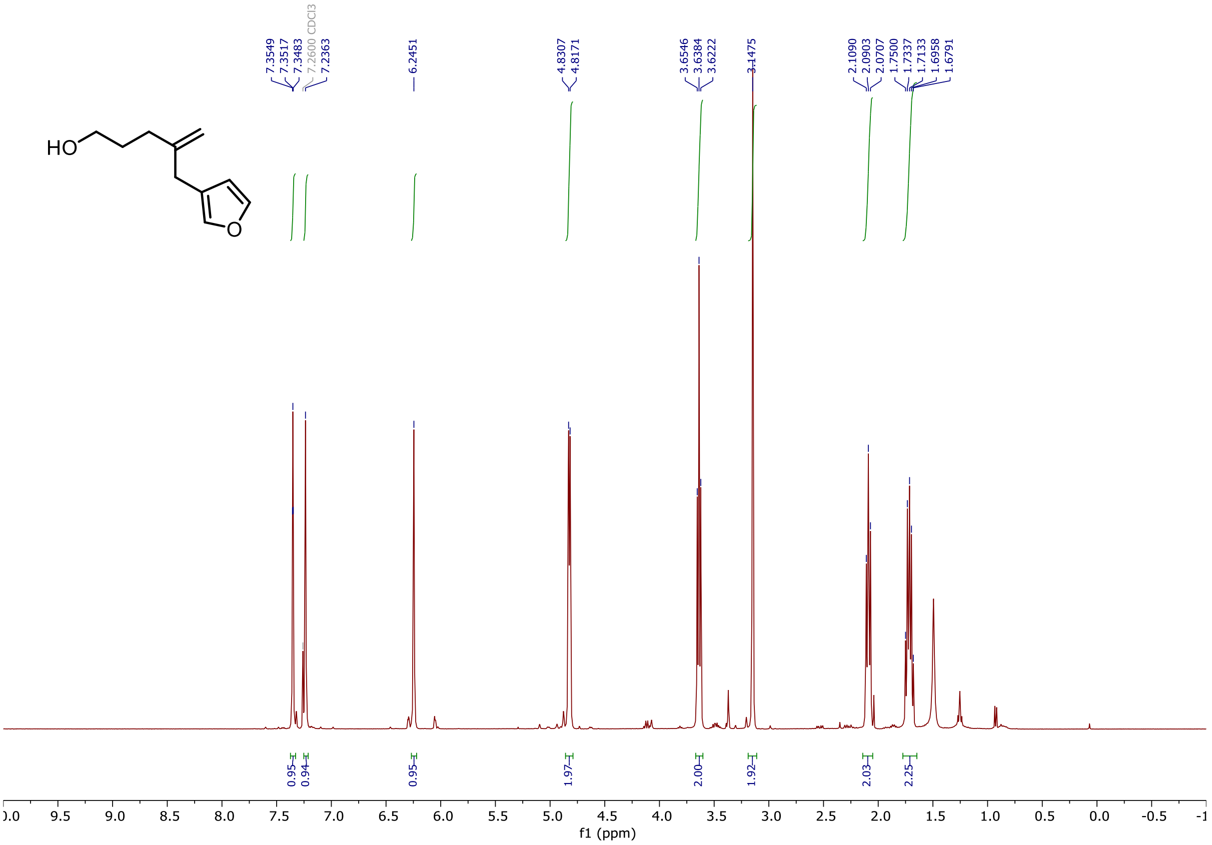

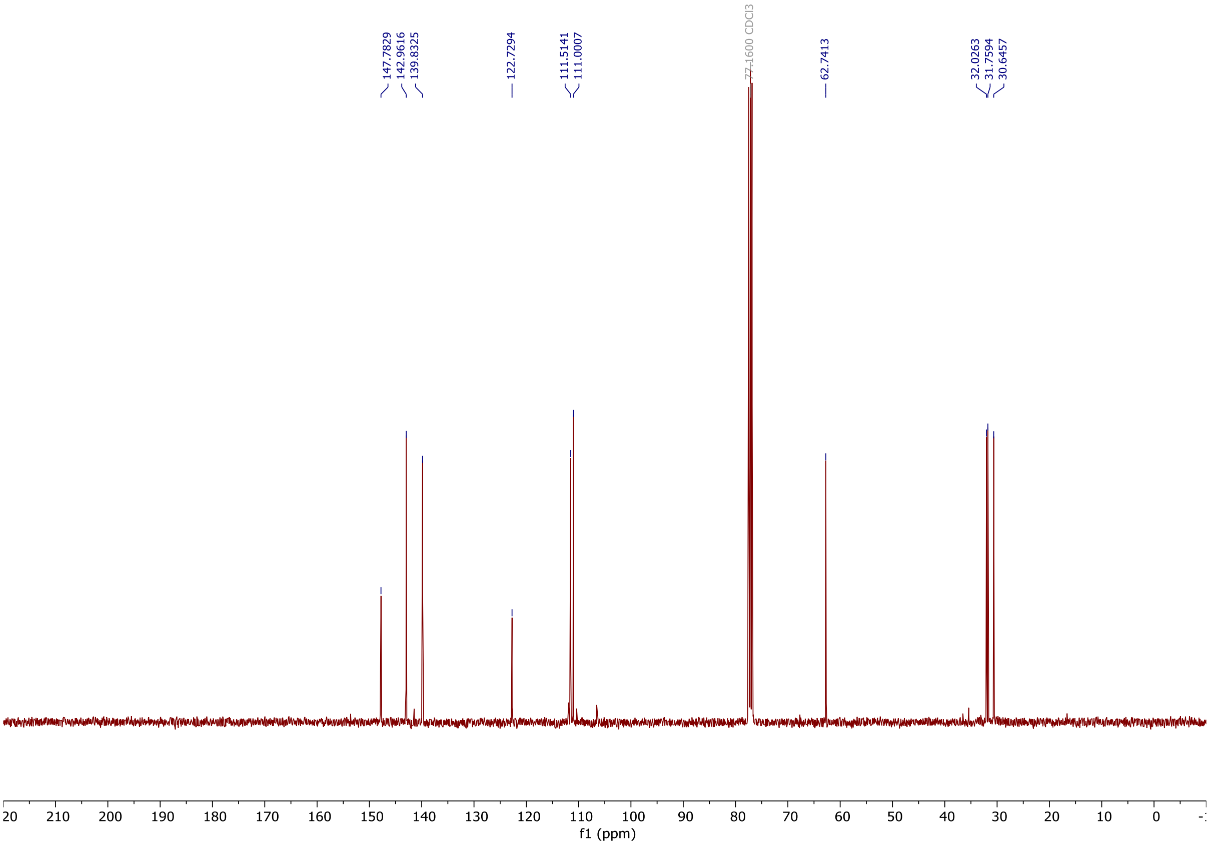

Benzyl (4-(furan-3-ylmethyl)pent-4-en-1-yl)((perfluorobenzoyl)oxy)carbamate (3f)

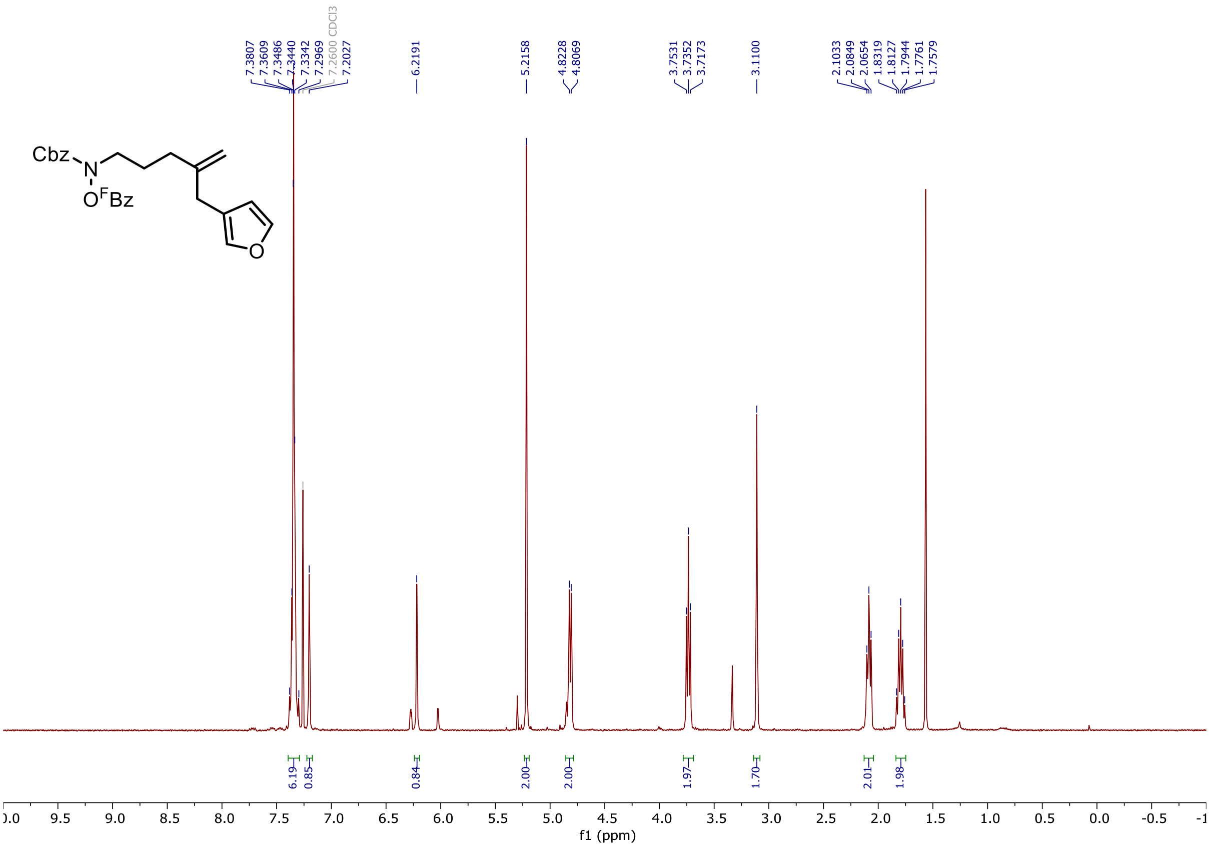

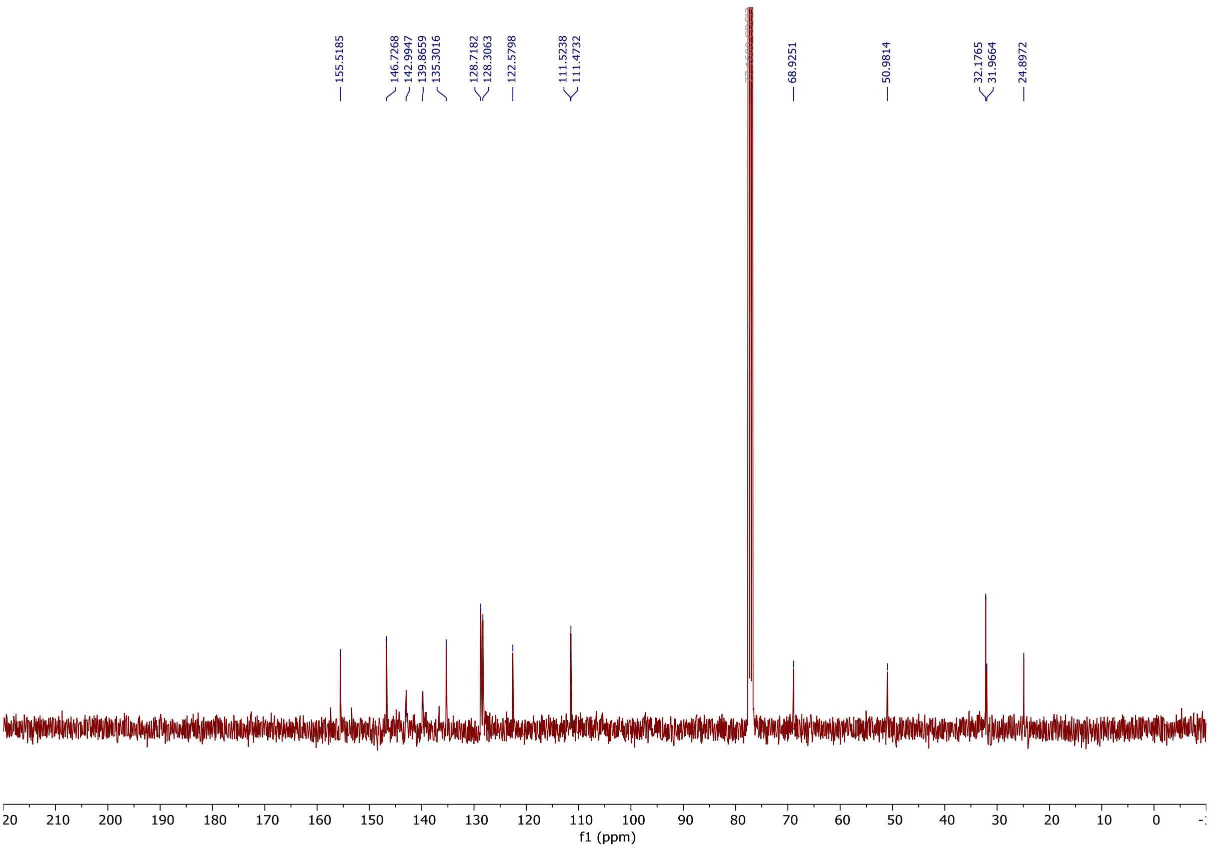

Benzyl 4,6-dihydrospiro[cyclopenta[*b*]furan-5,2'-pyrrolidine]-1'-carboxylate (4f)

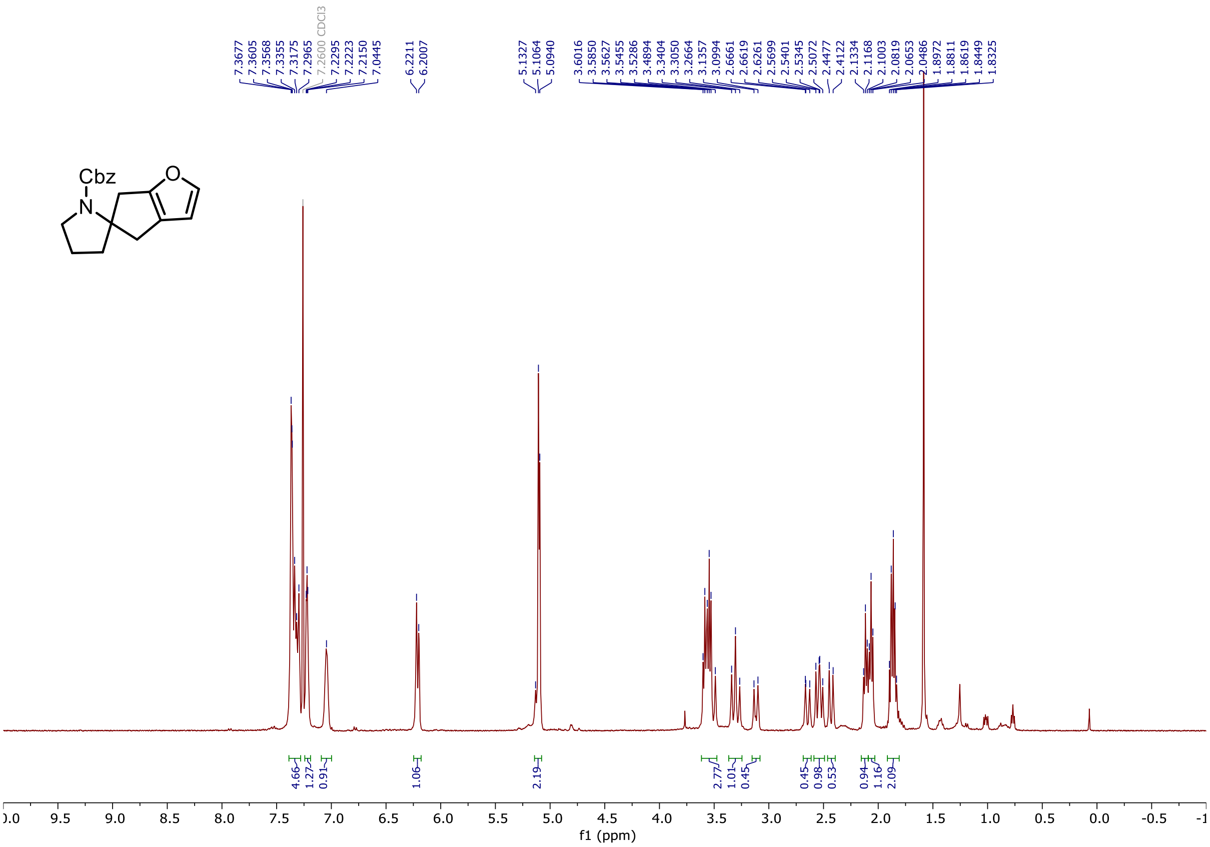

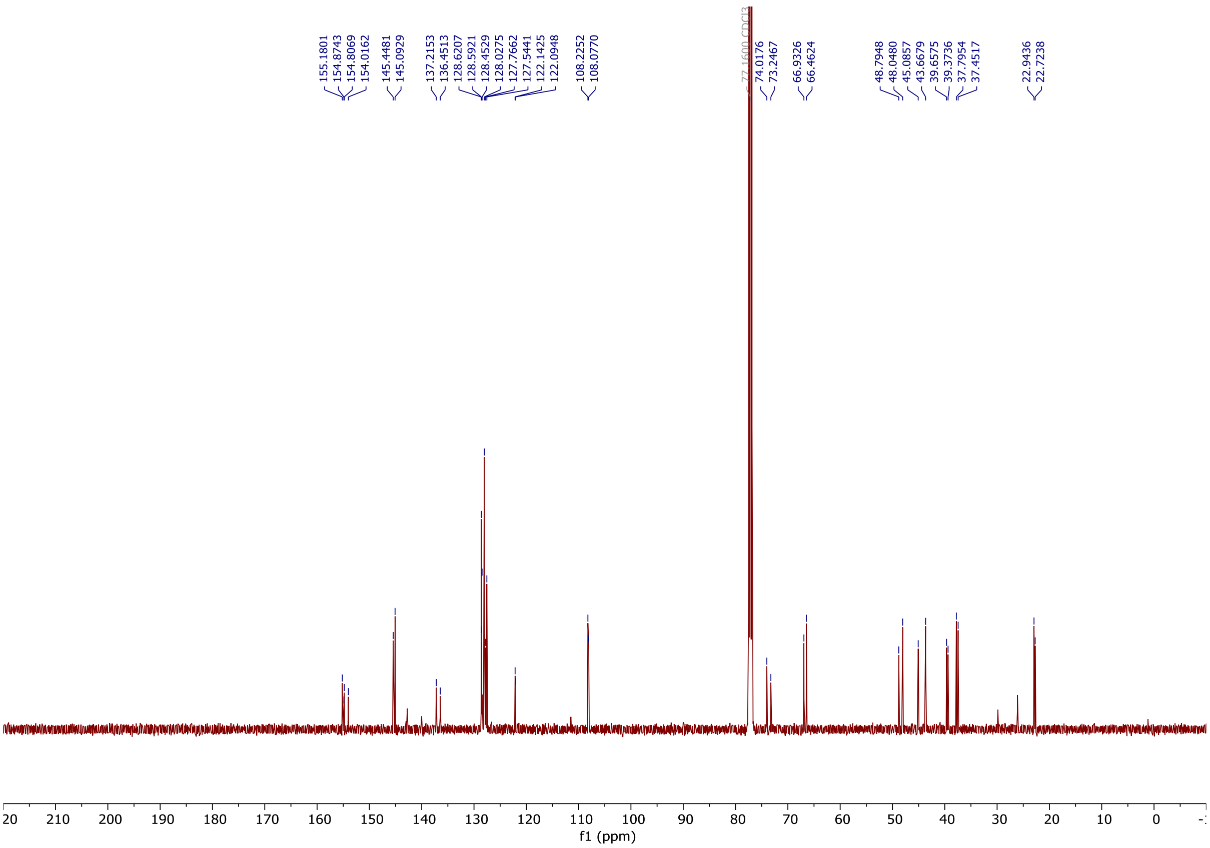

4-(4-Methoxybenzyl)pent-4-enal

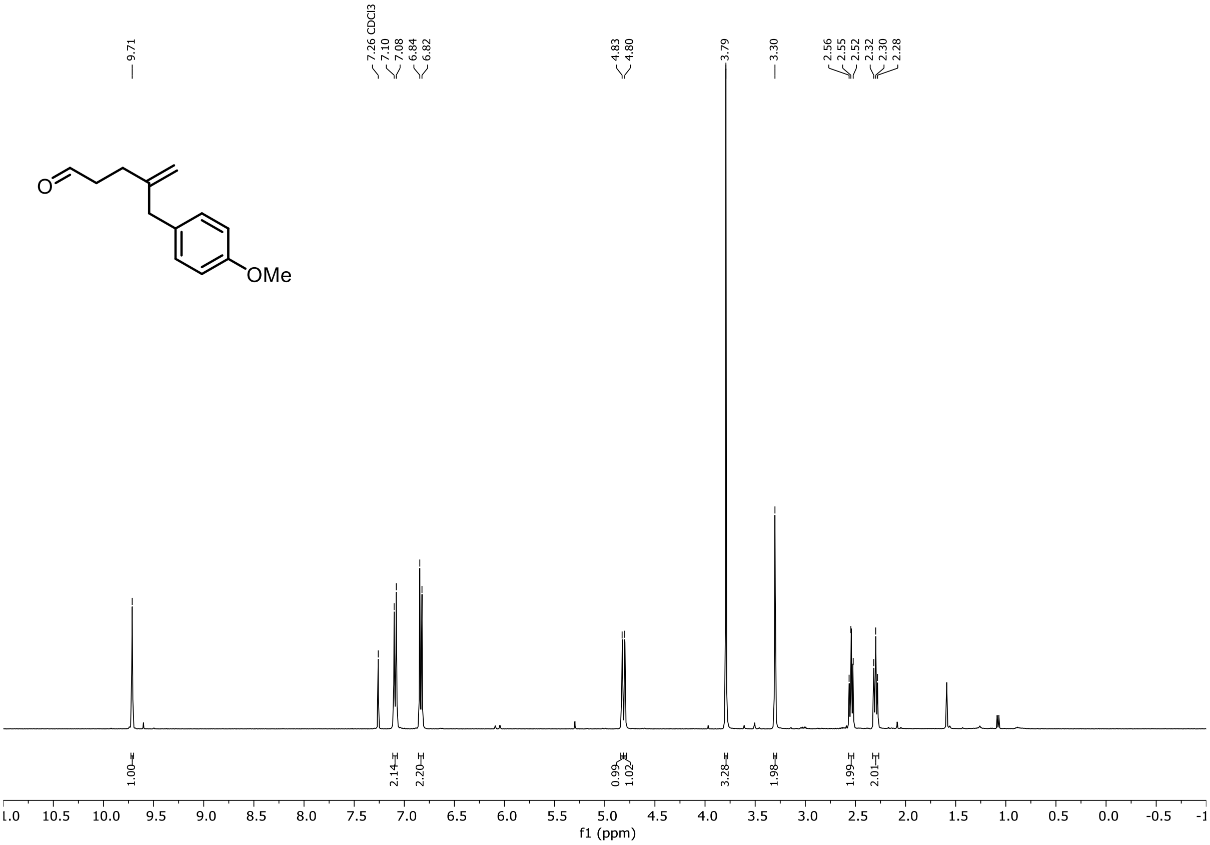

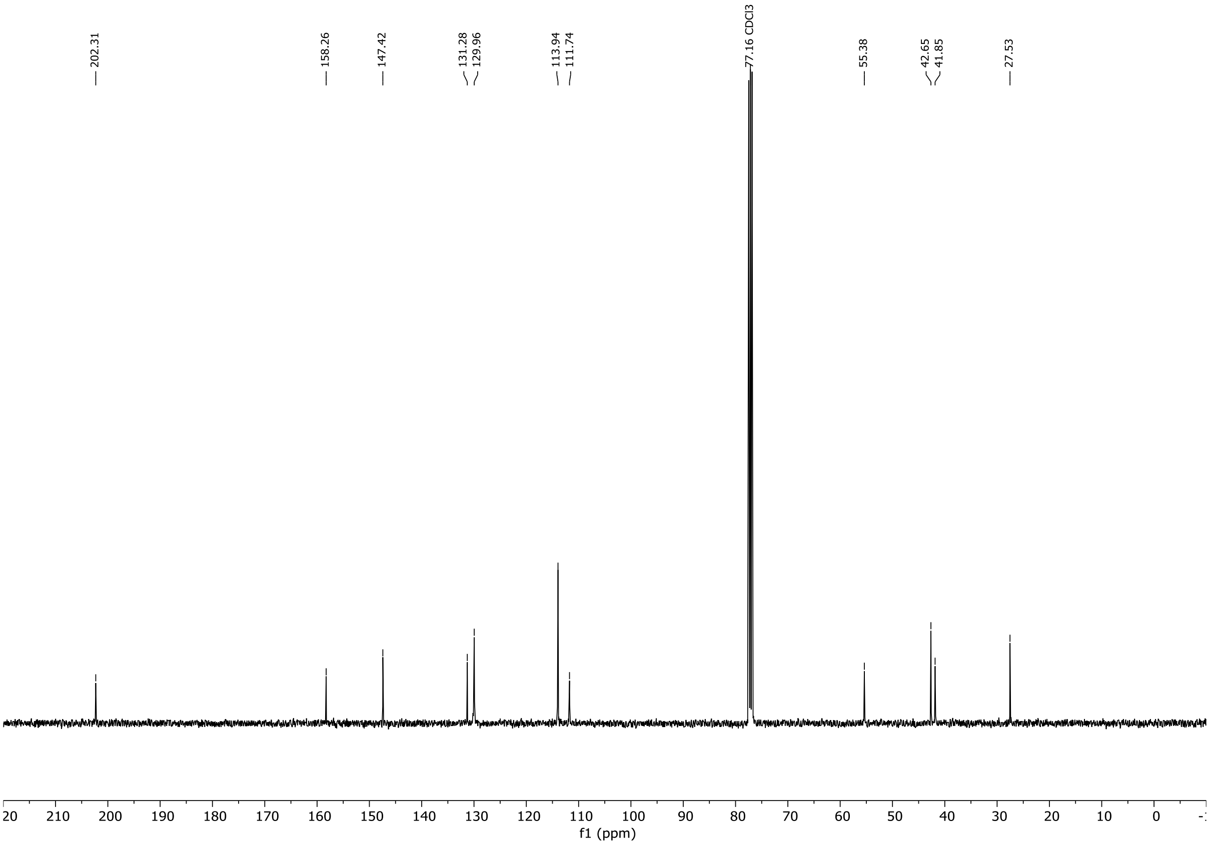

5-(4-Methoxybenzyl)hex-5-en-2-ol

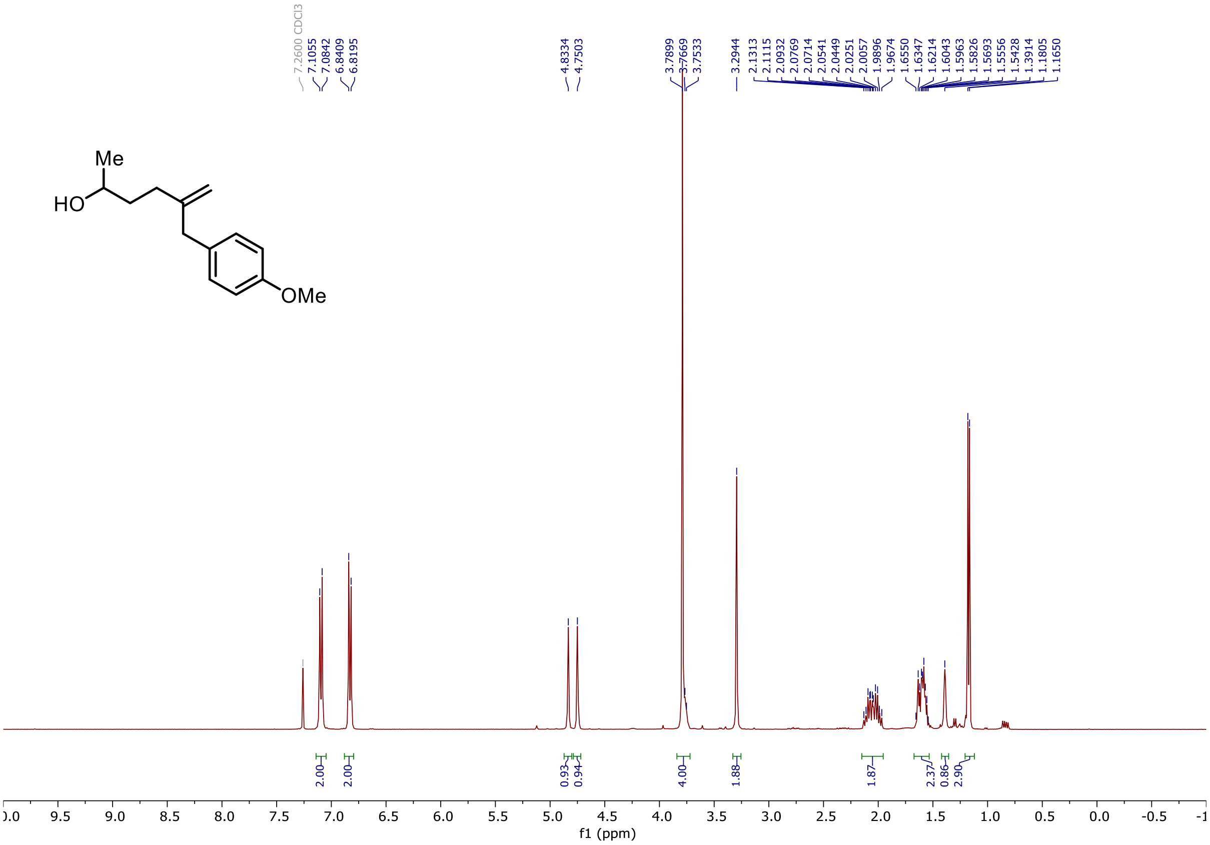

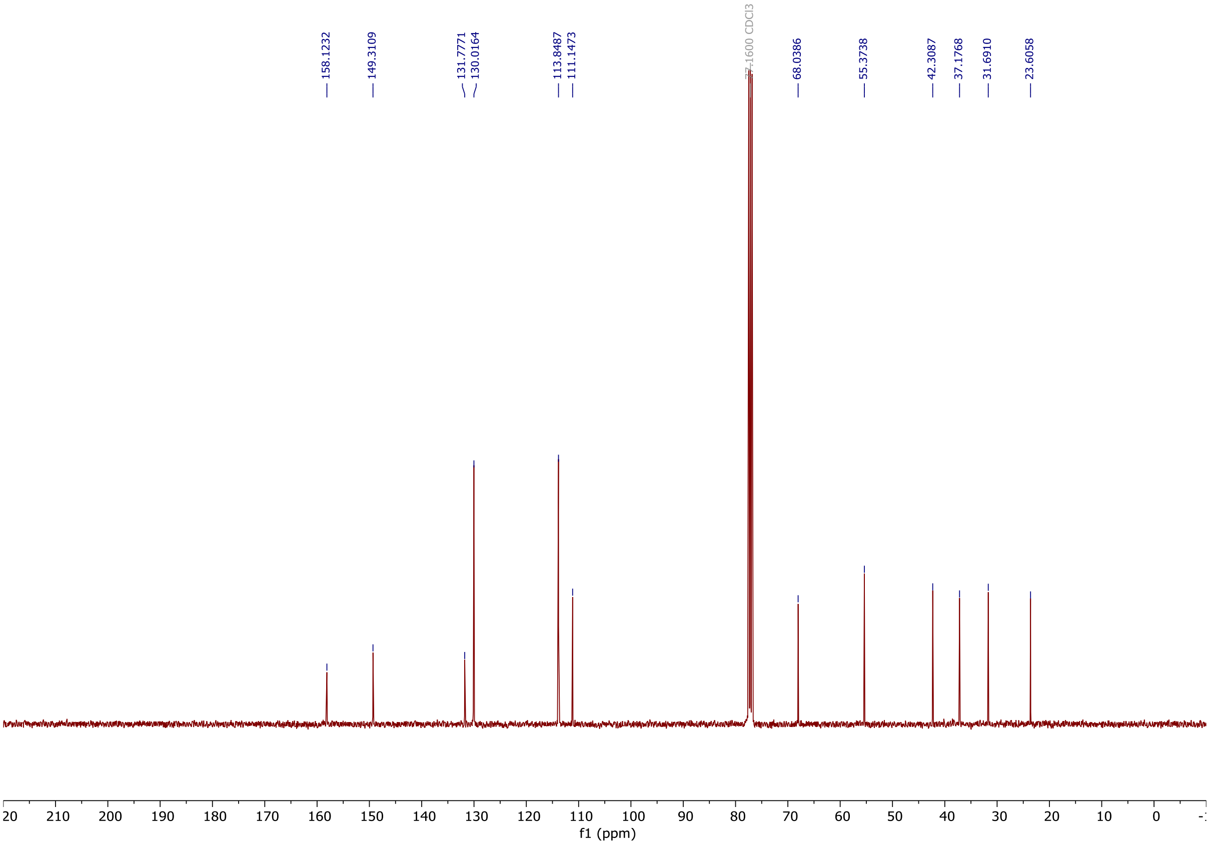

Benzyl (5-(4-methoxybenzyl)hex-5-en-2-yl)((perfluorobenzoyl)oxy)carbamate (3h)

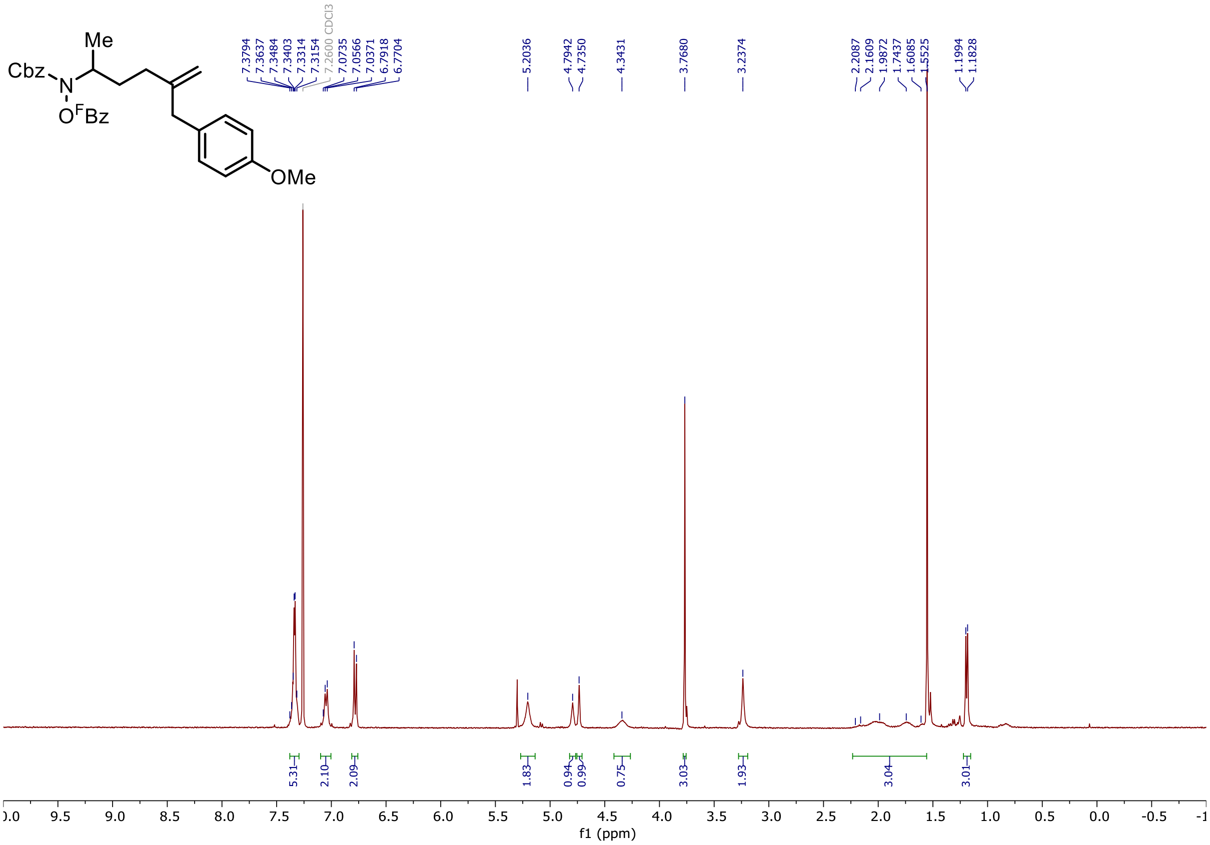

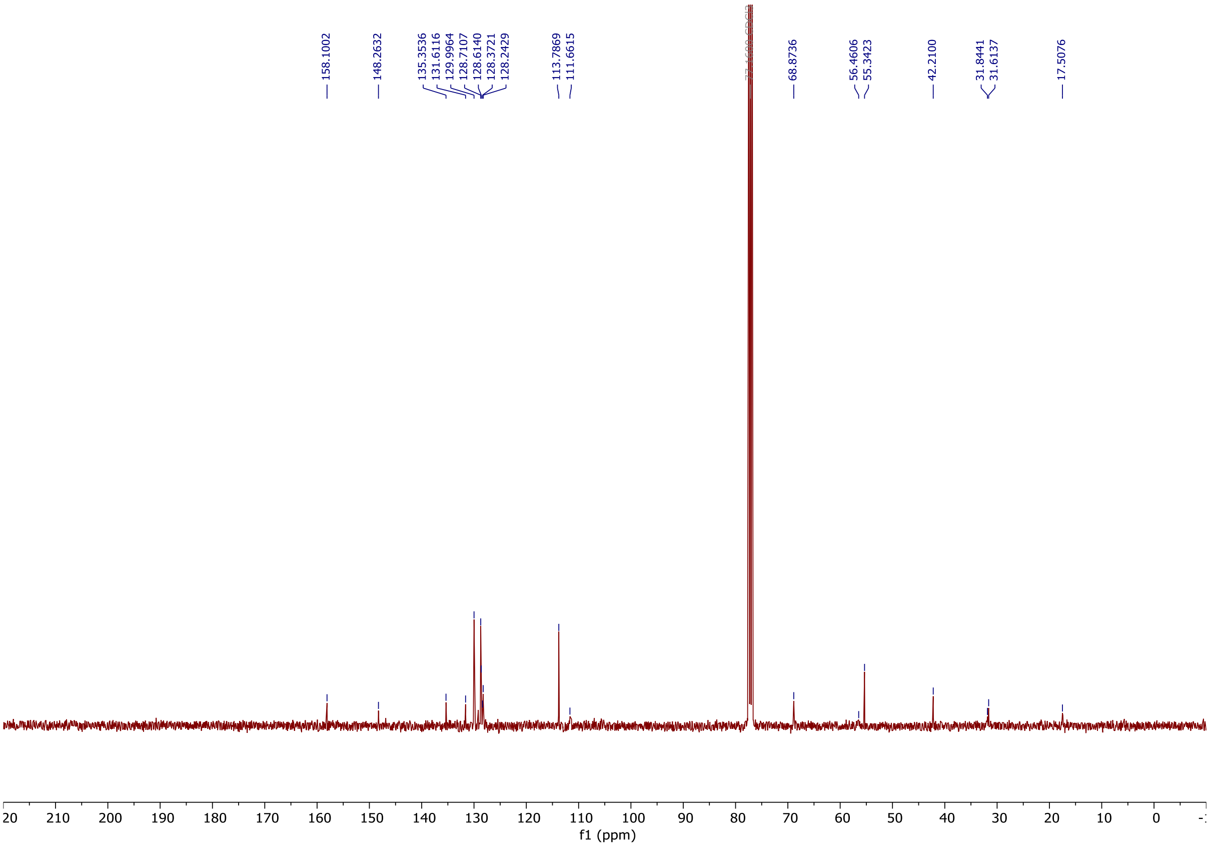

Benzyl 5-methoxy-5'-methyl-1,3-dihydrospiro[indene-2,2'-pyrrolidine]-1'-carboxylate (4h)

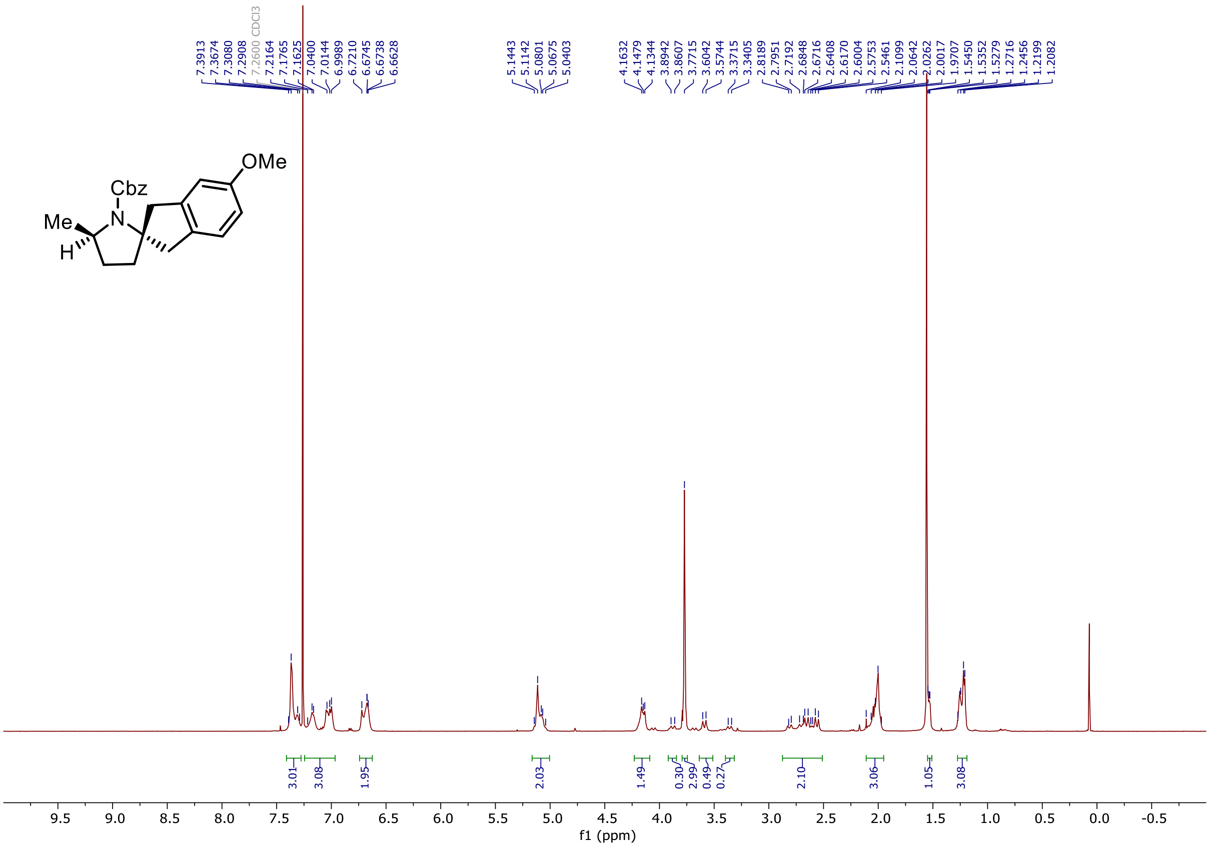

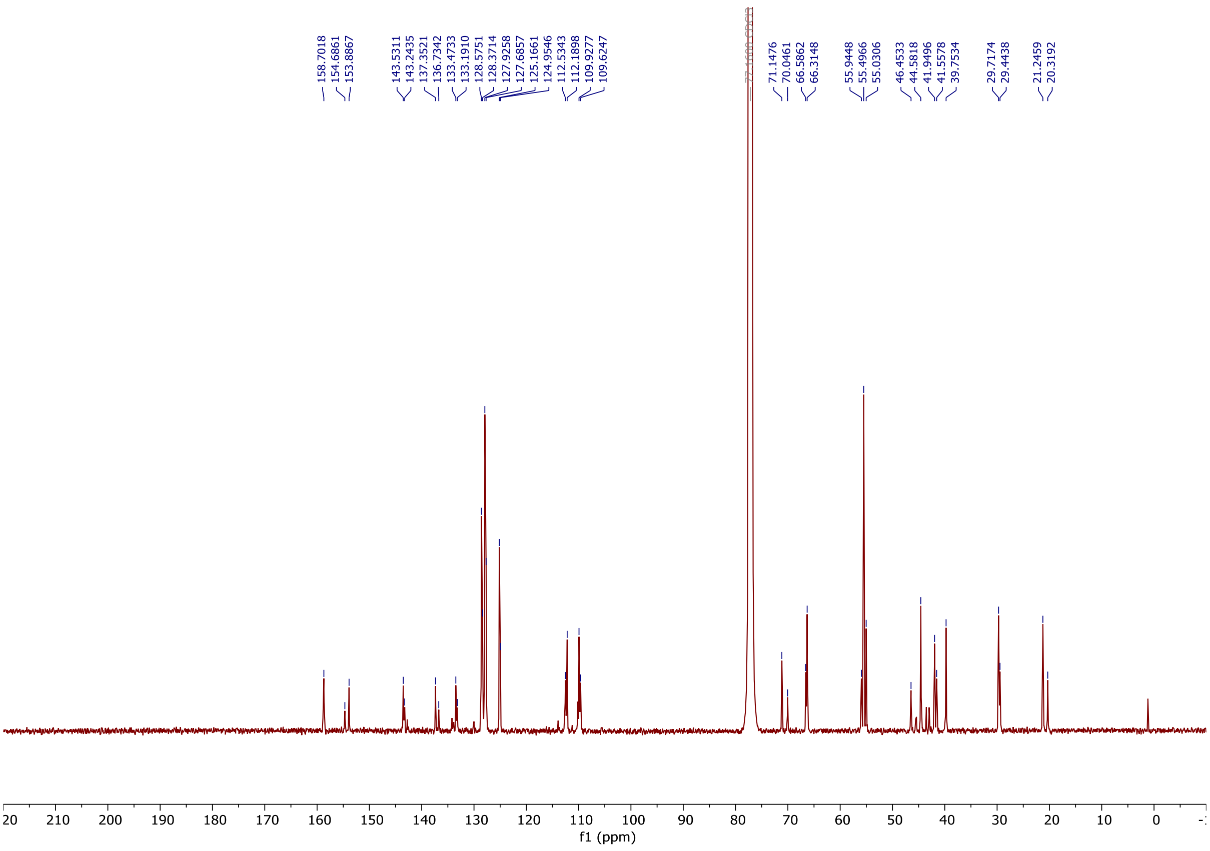

5-Methoxy-5'-methyl-1,3-dihydrospiro[indene-2,2'-pyrrolidine]

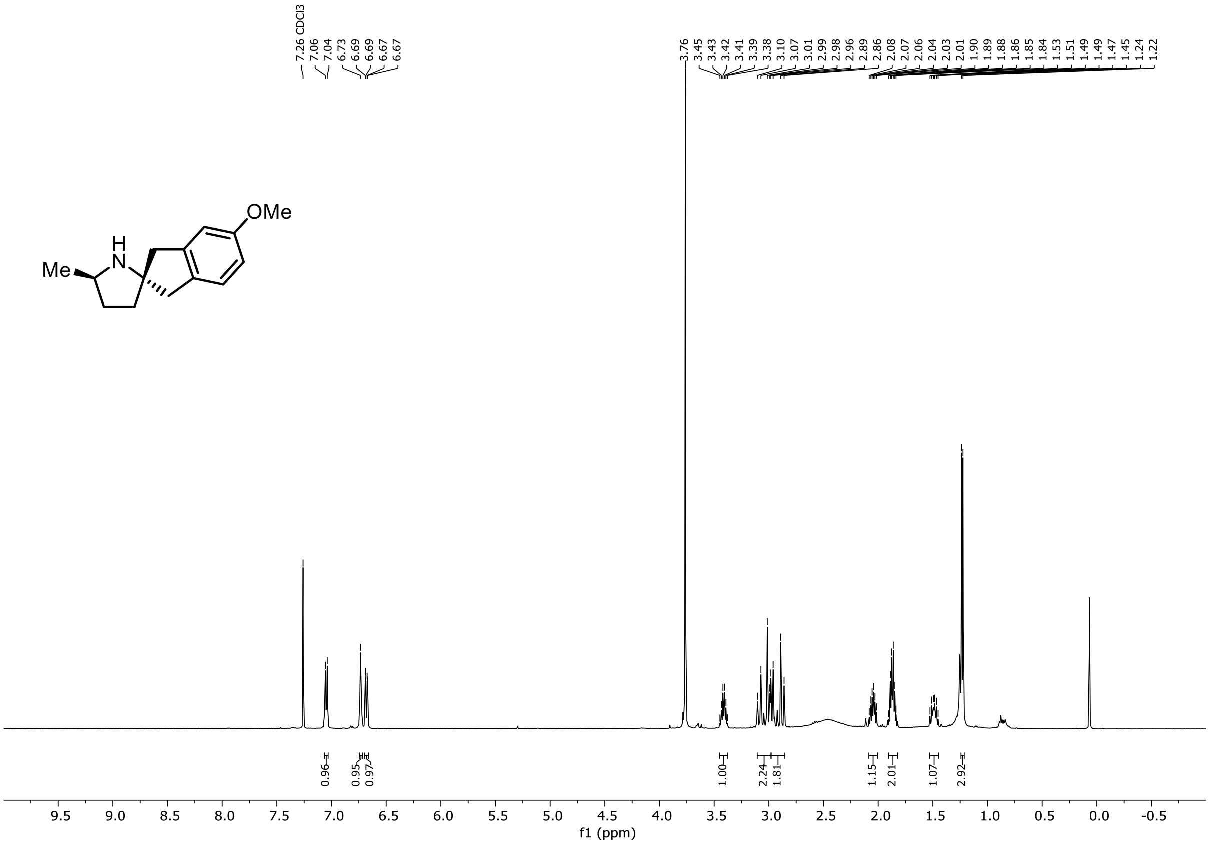

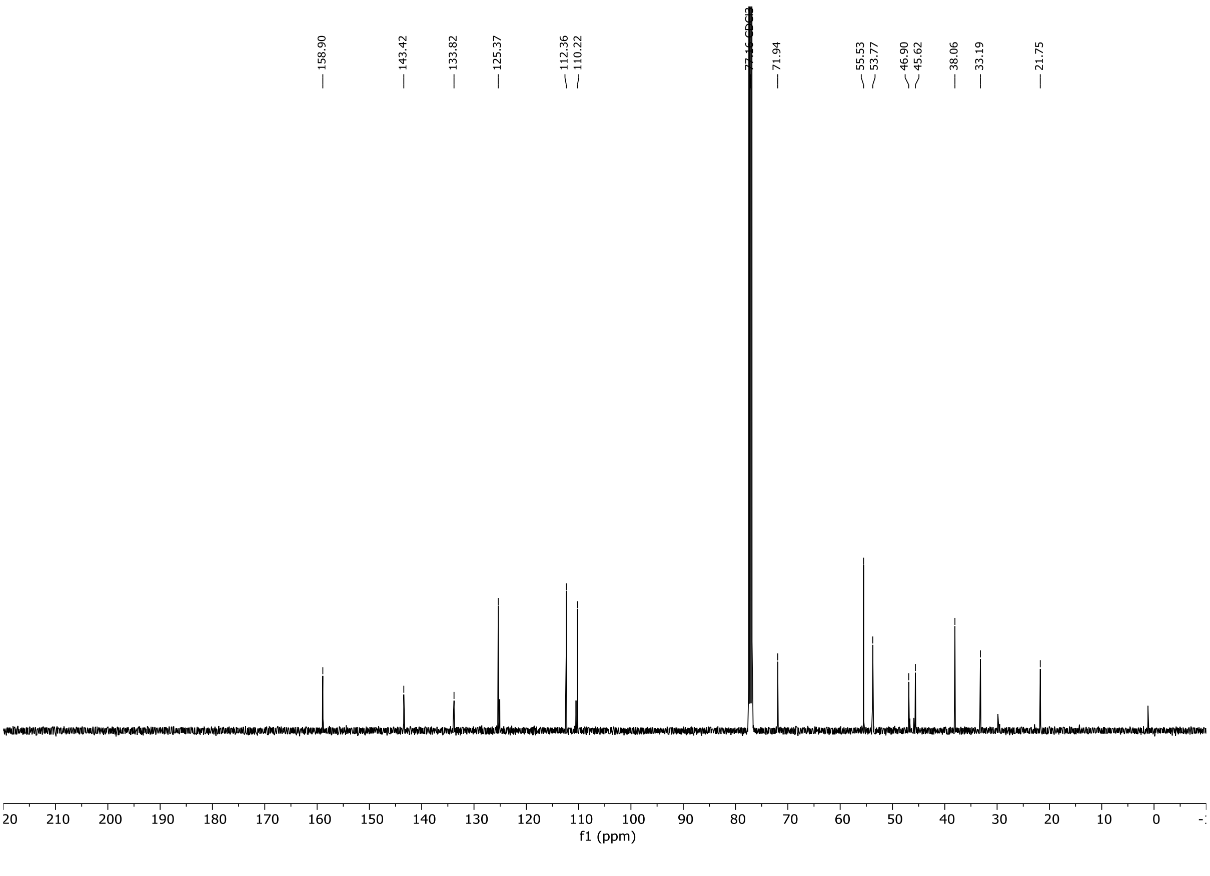

4-(4-Methoxybenzyl)-1-phenylpent-4-en-1-ol

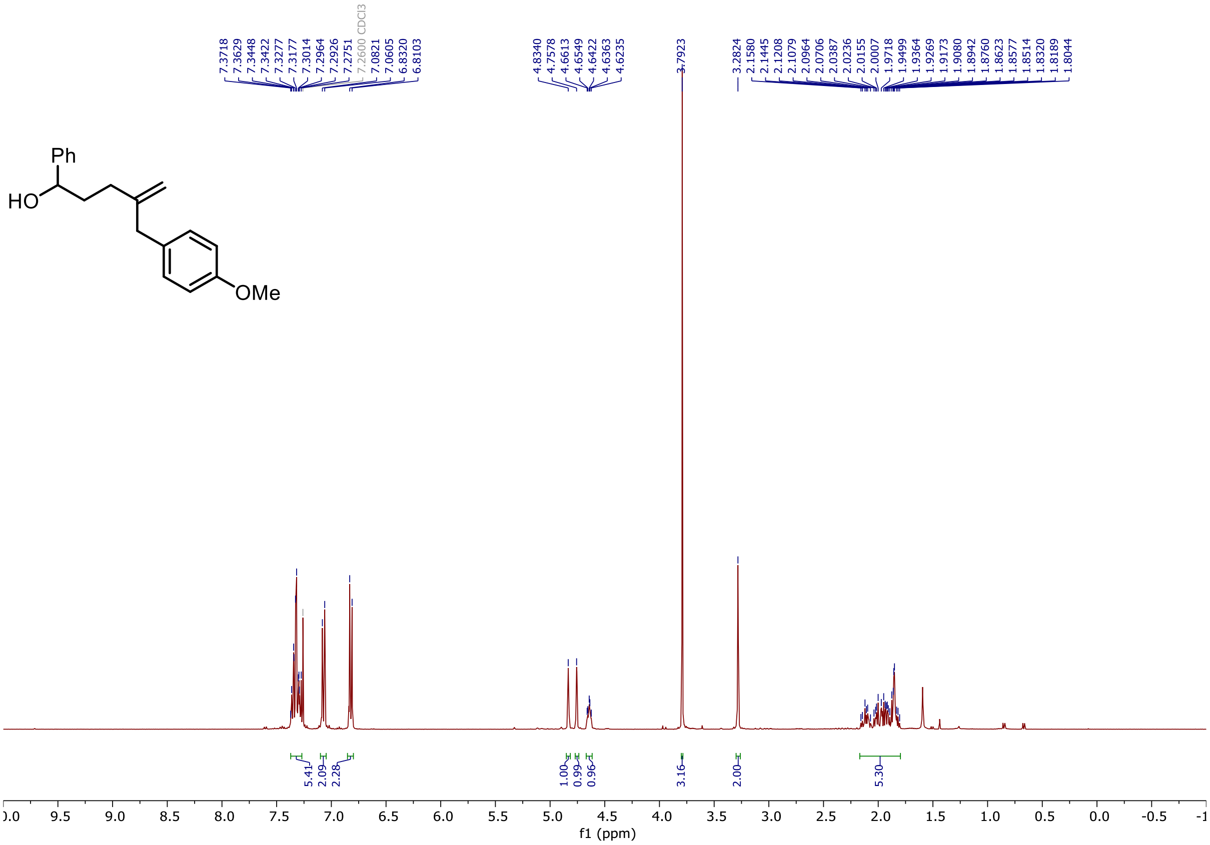

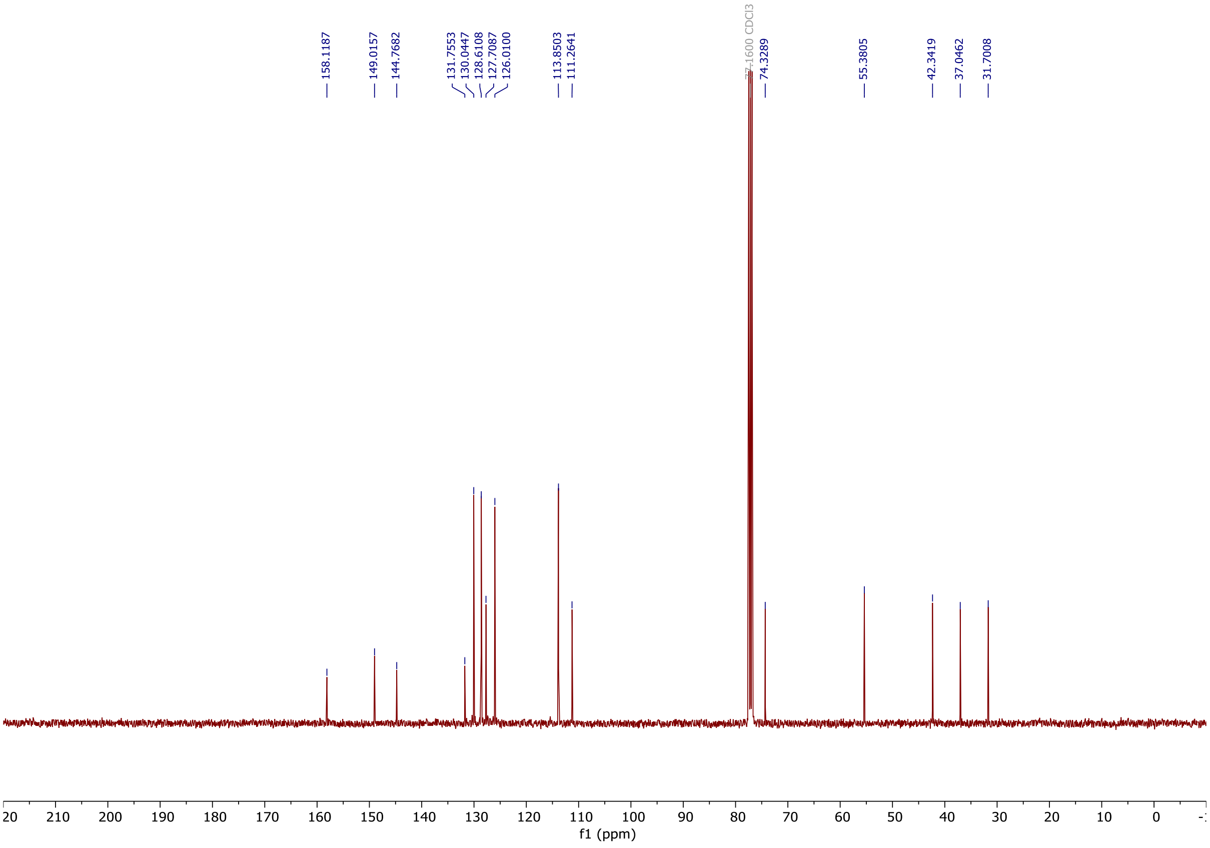

Benzyl (4-(4-methoxybenzyl)-1-phenylpent-4-en-1-yl)((perfluorobenzoyl)oxy)carbamate (3i)

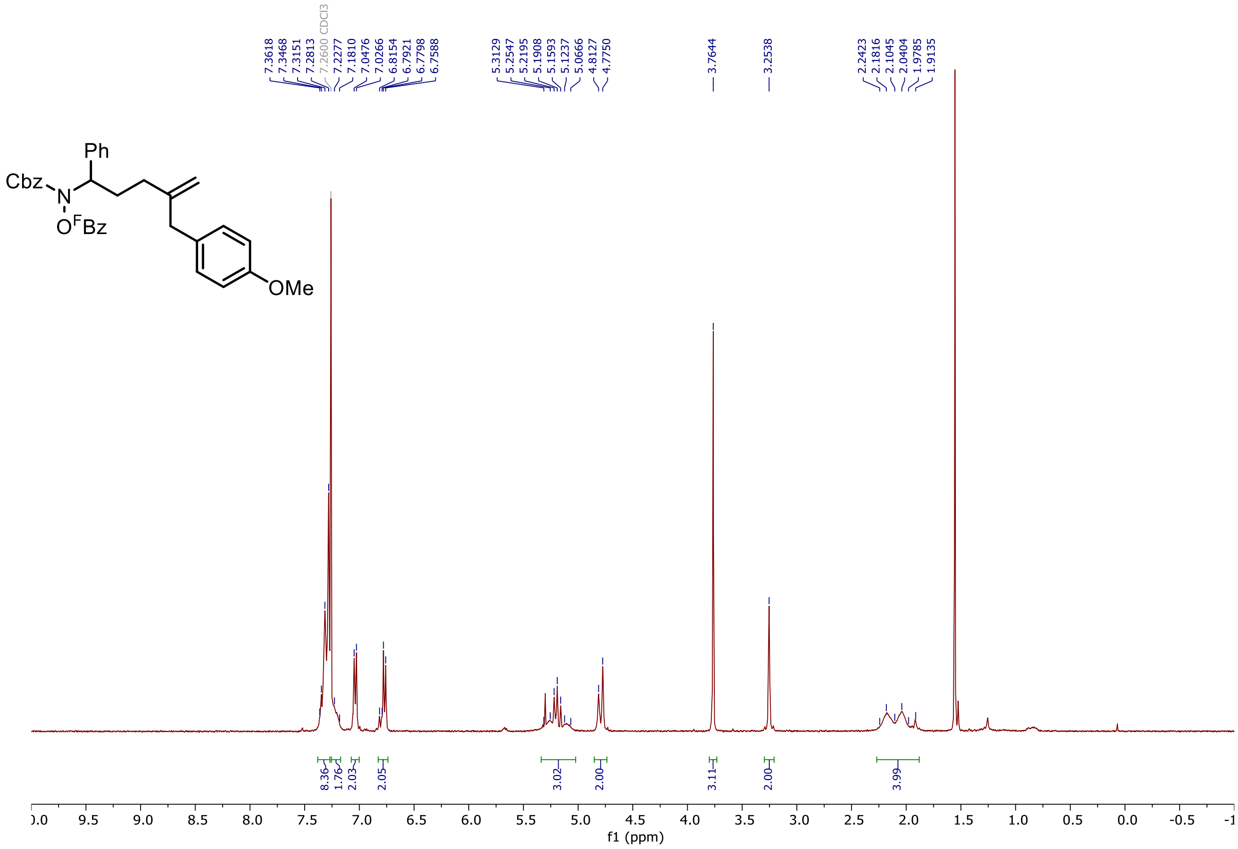

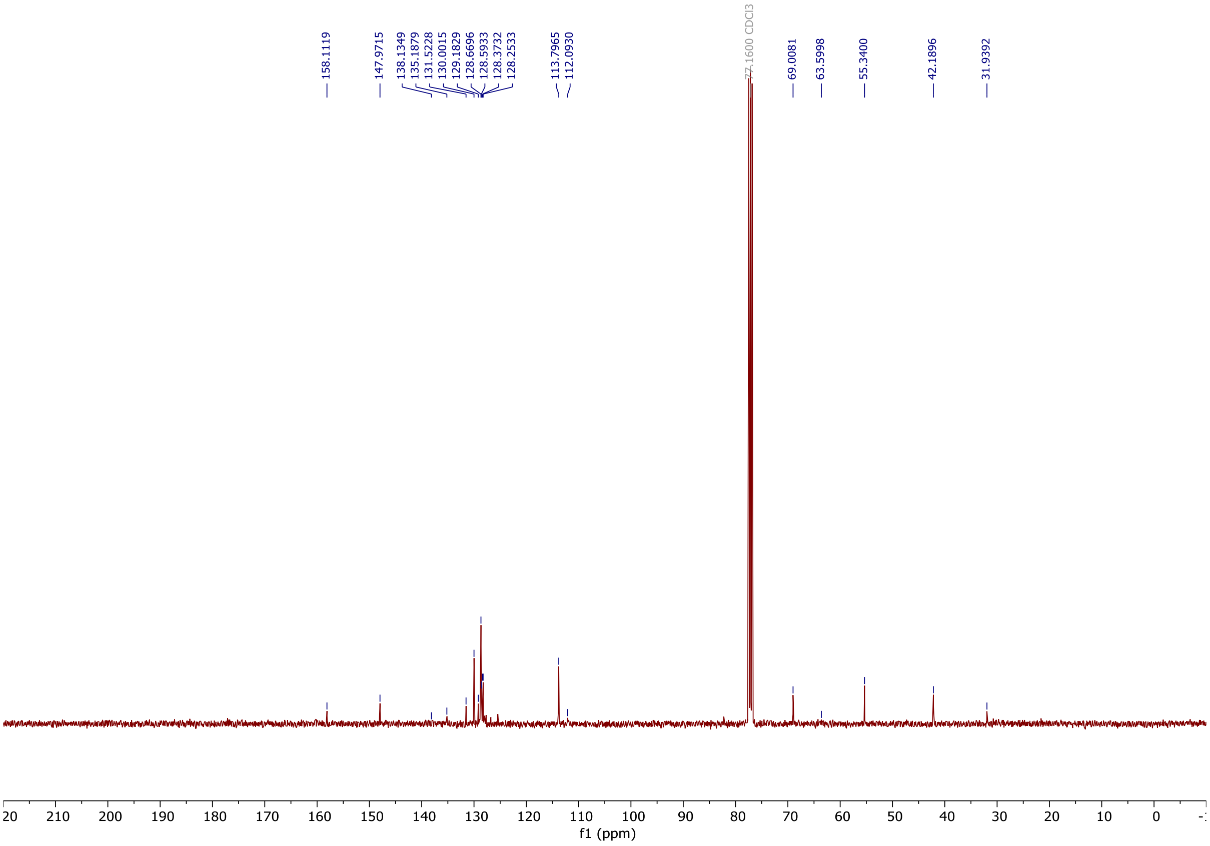

Benzyl 5-methoxy-5'-phenyl-1,3-dihydrospiro[indene-2,2'-pyrrolidine]-1'-carboxylate (4i)

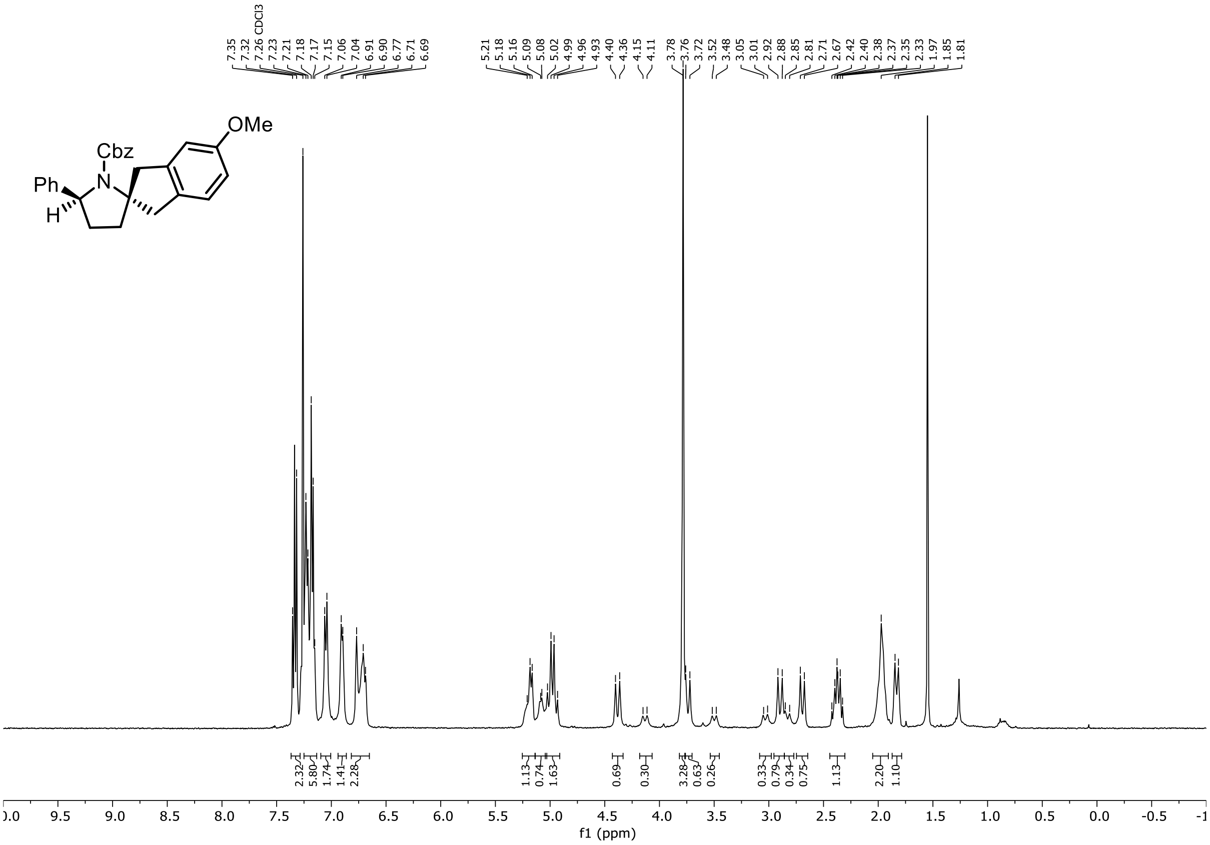

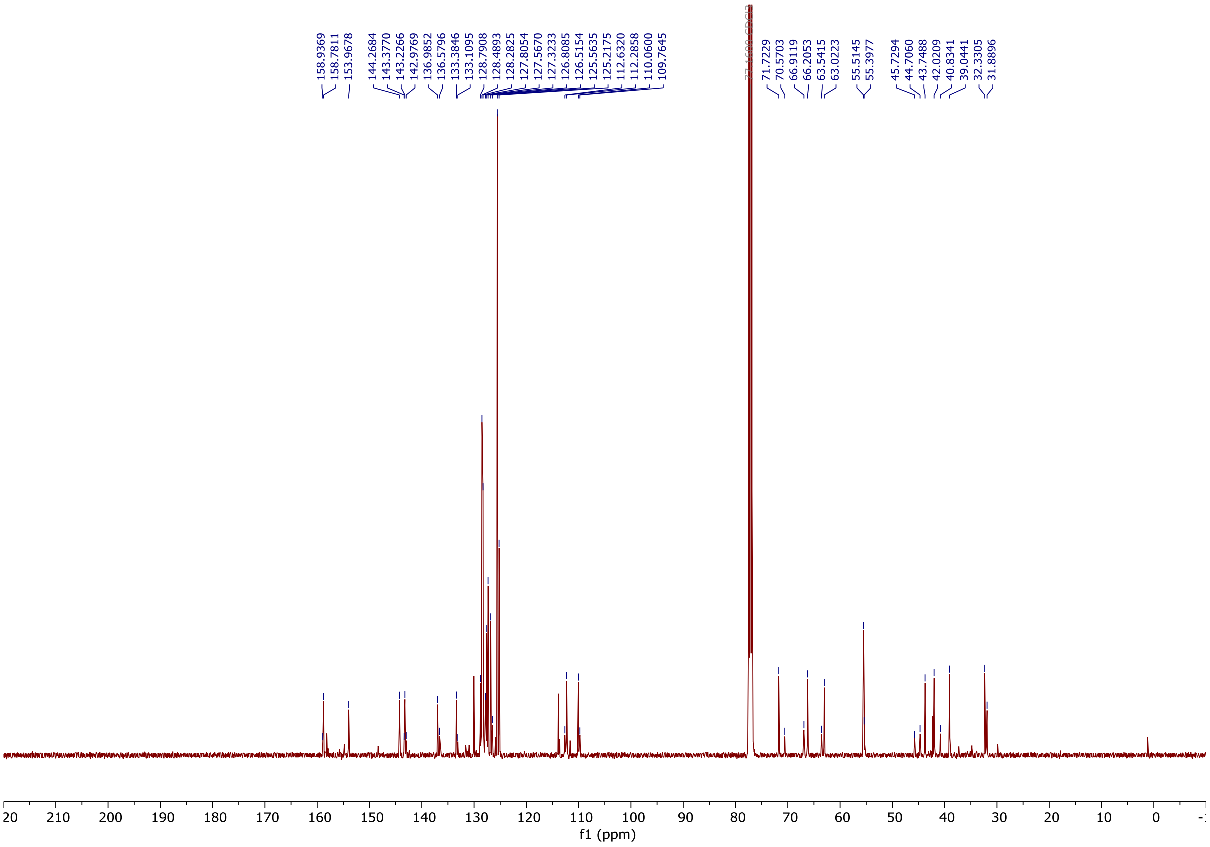

(3*R*,5*R*)-5-(4-(Benzyloxy)butyl)-3-(3,4-dimethoxyphenyl)dihydrofuran-2(3*H*)-one (*cis*-7)

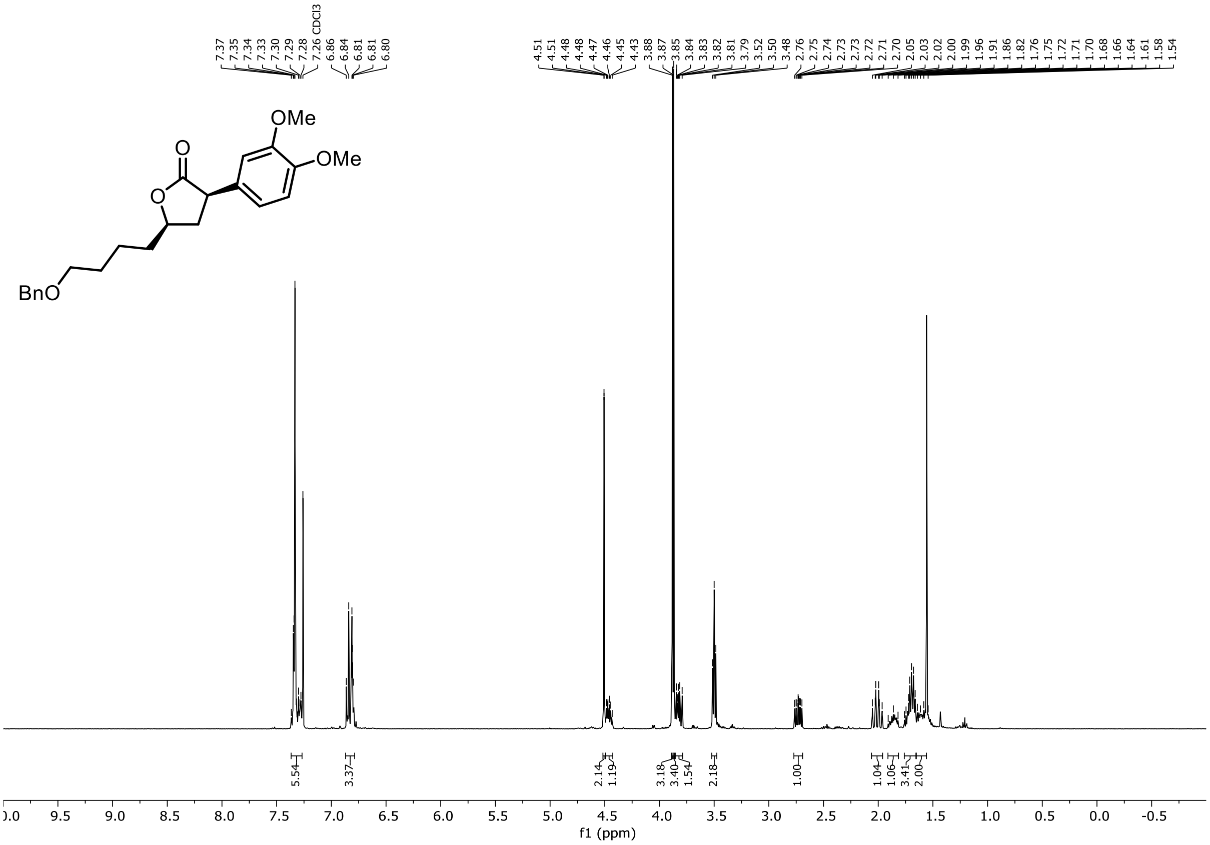

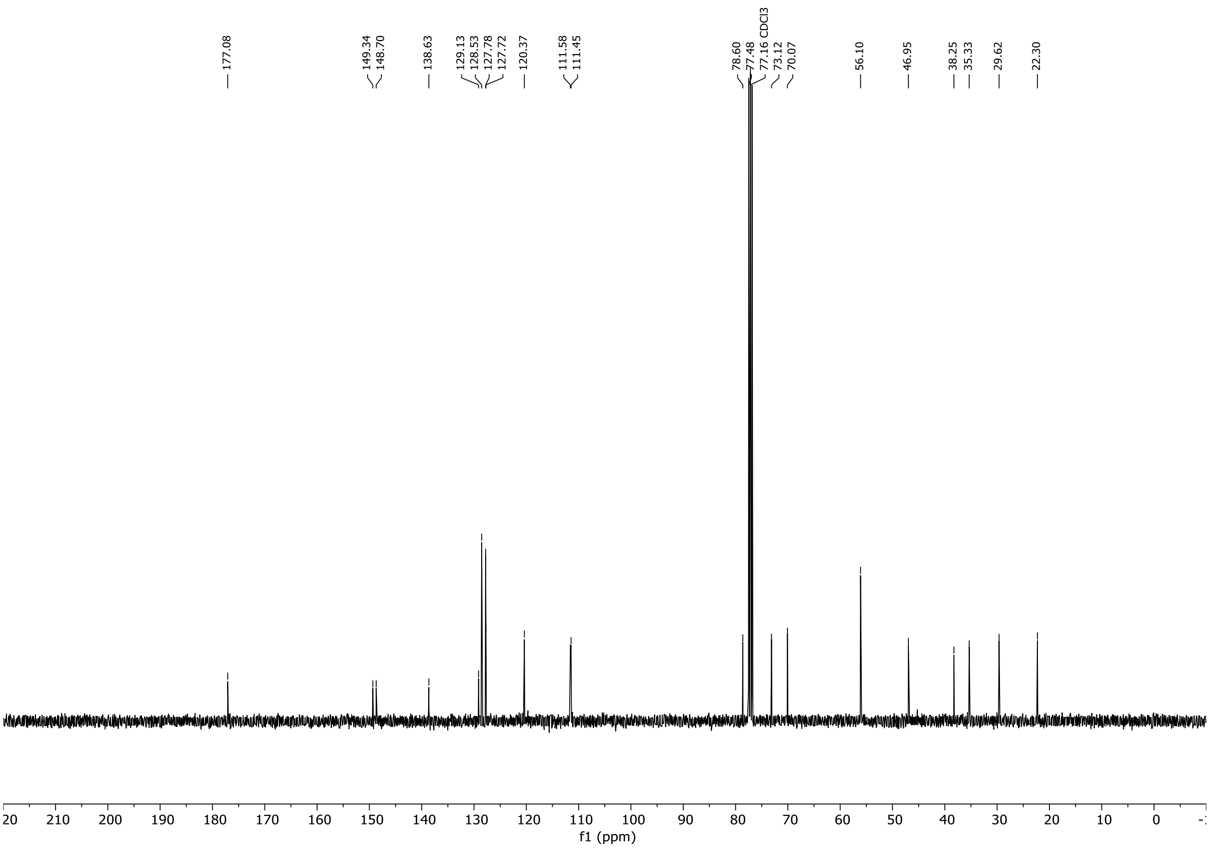

(3*S*,5*R*)-5-(4-(benzyloxy)butyl)-3-(3,4-dimethoxyphenyl)dihydrofuran-2(3*H*)-one (*trans*-7)

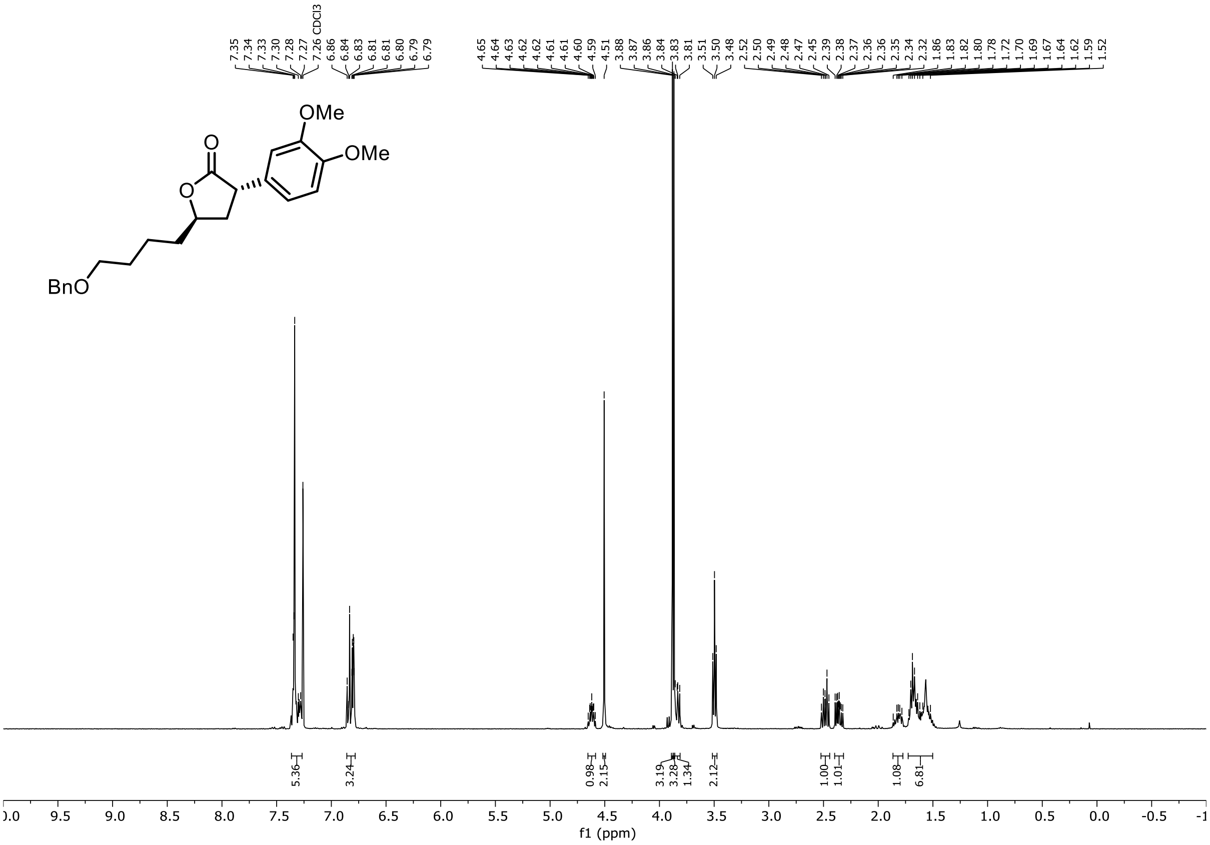

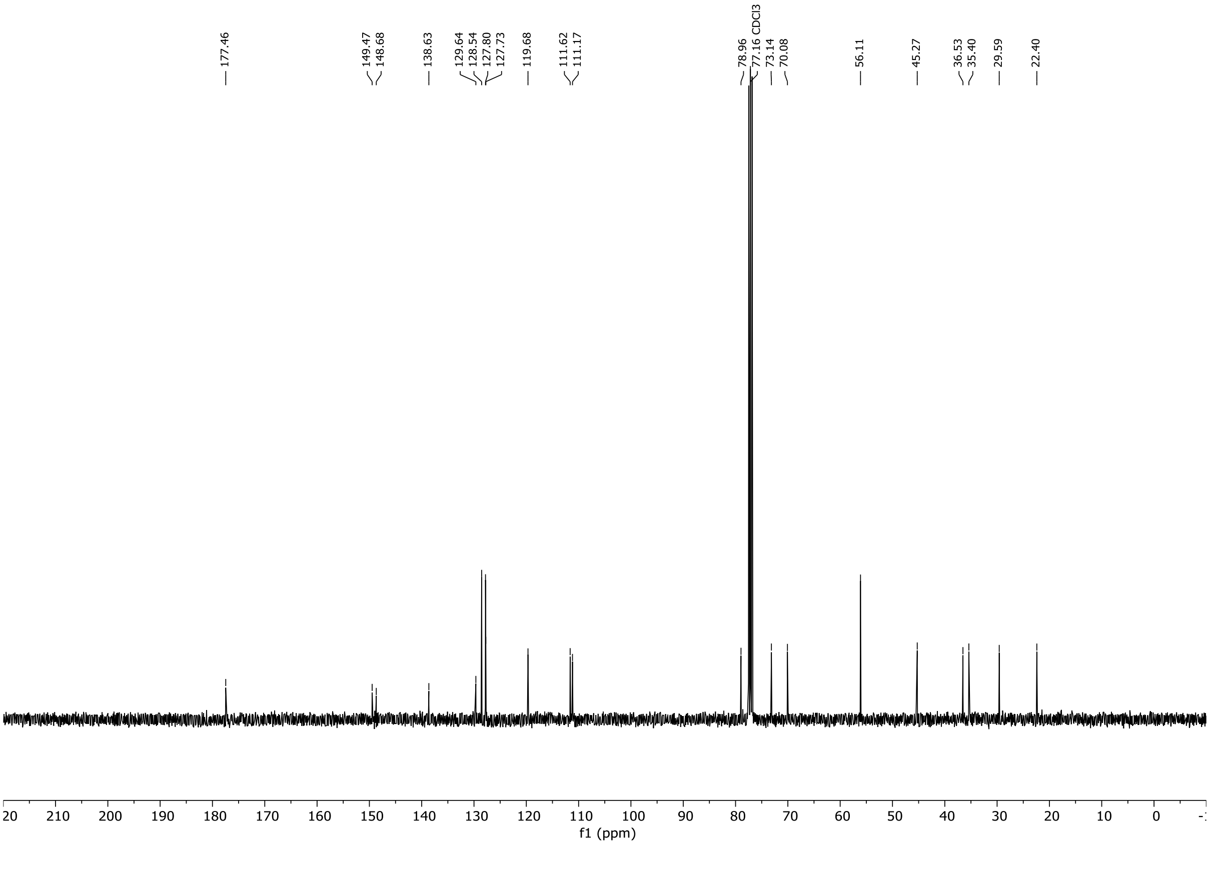

(3*R*,5*R*)-3-(3,4-Dimethoxyphenyl)-5-(4-hydroxybutyl)dihydrofuran-2(3*H*)-one

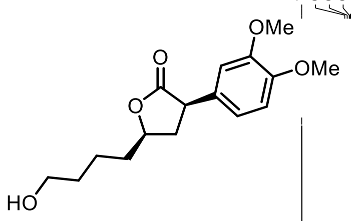

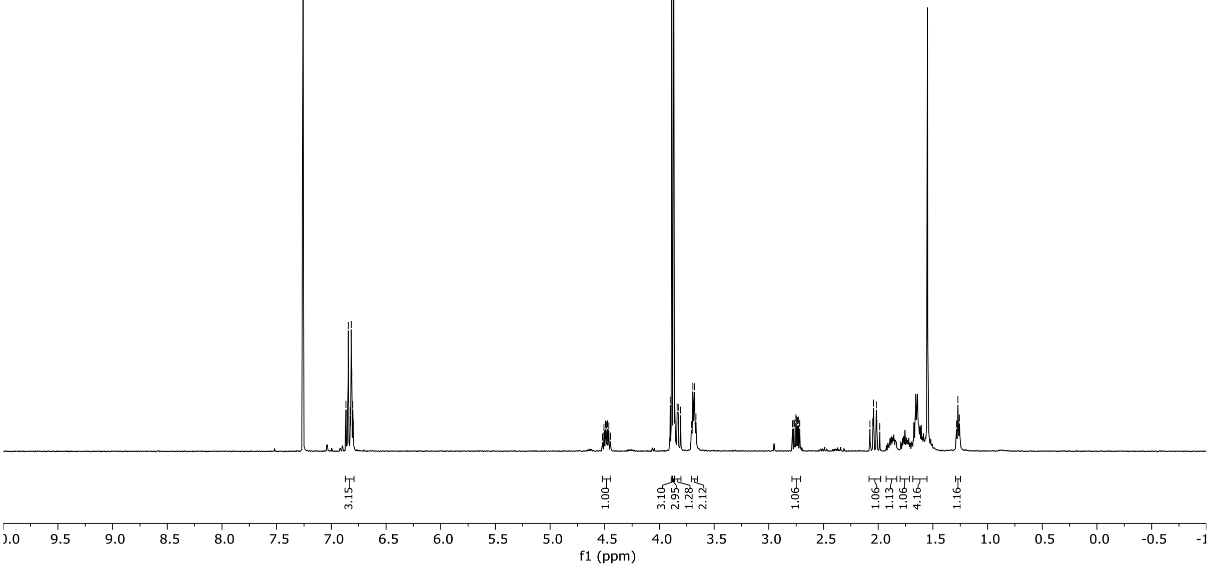

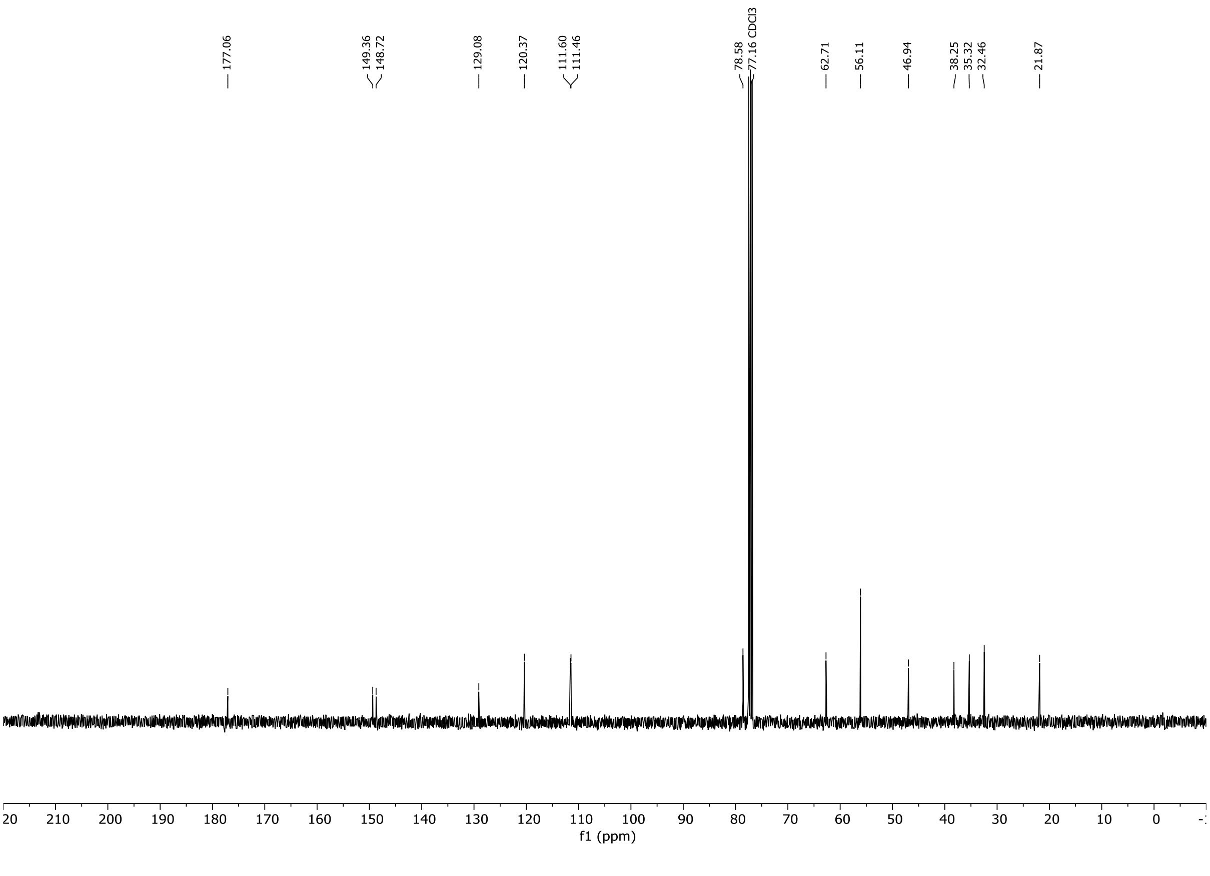

4-((2*R*,4*R*)-4-(3,4-Dimethoxyphenyl)-5-oxotetrahydrofuran-2-yl)butyl 4-bromobenzoate (8)

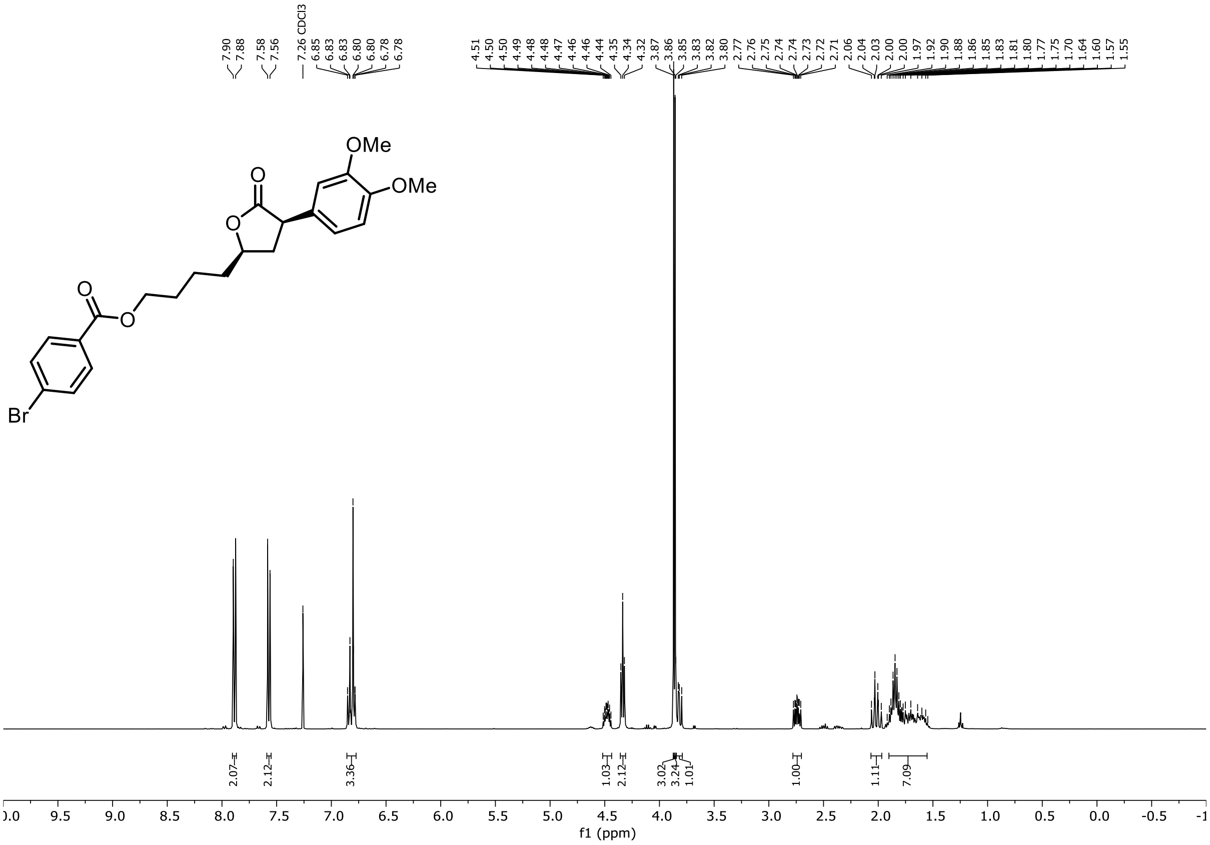

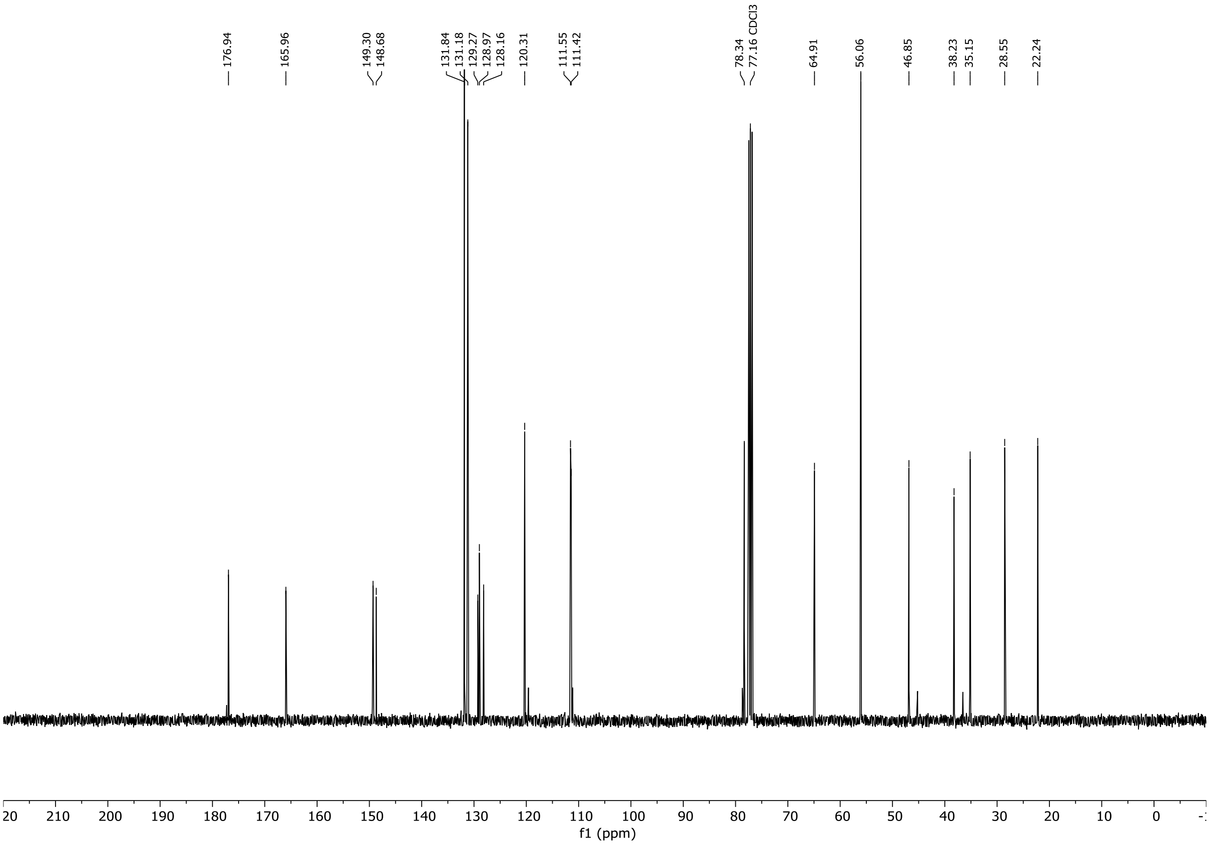

(3*R*,5*R*)-9-(Benzyloxy)-3-(3,4-dimethoxyphenyl)-2-(4-methoxyphenyl)non-1-en-5-ol (11)

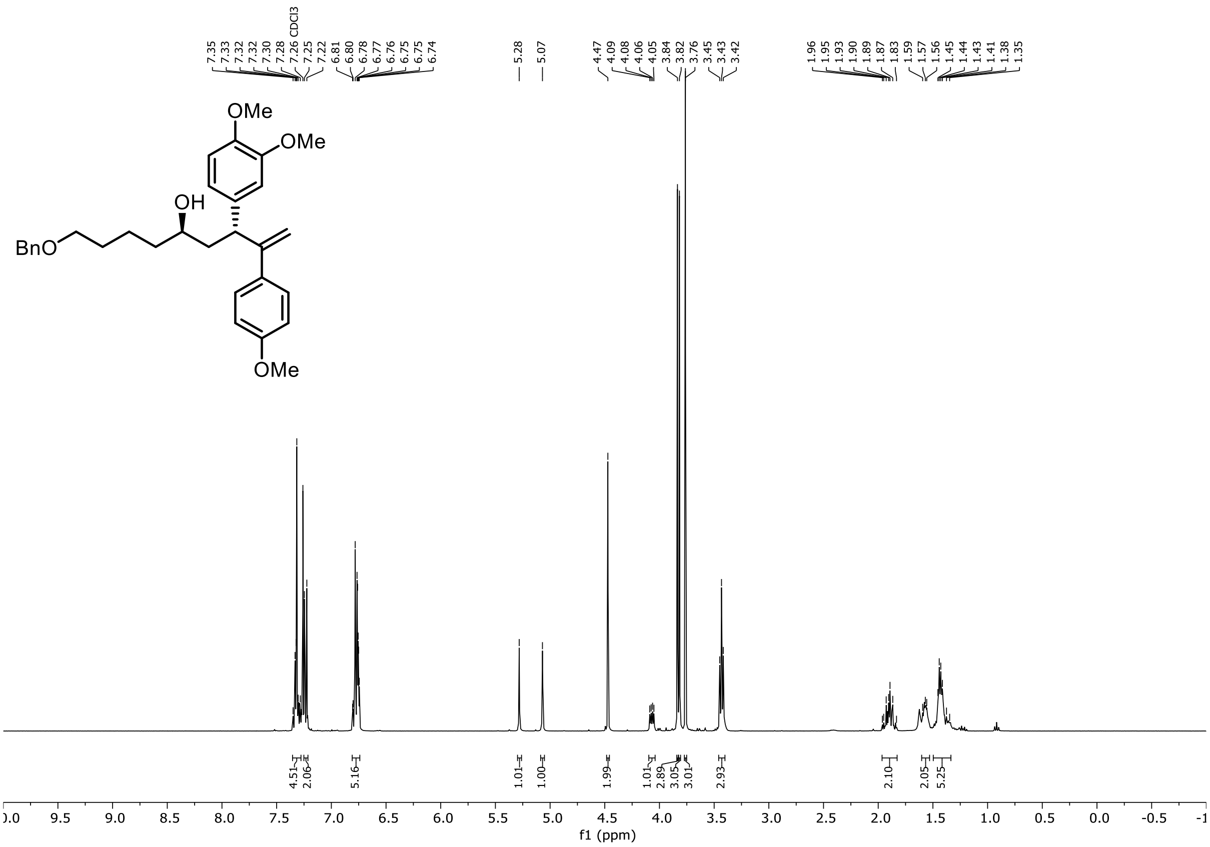

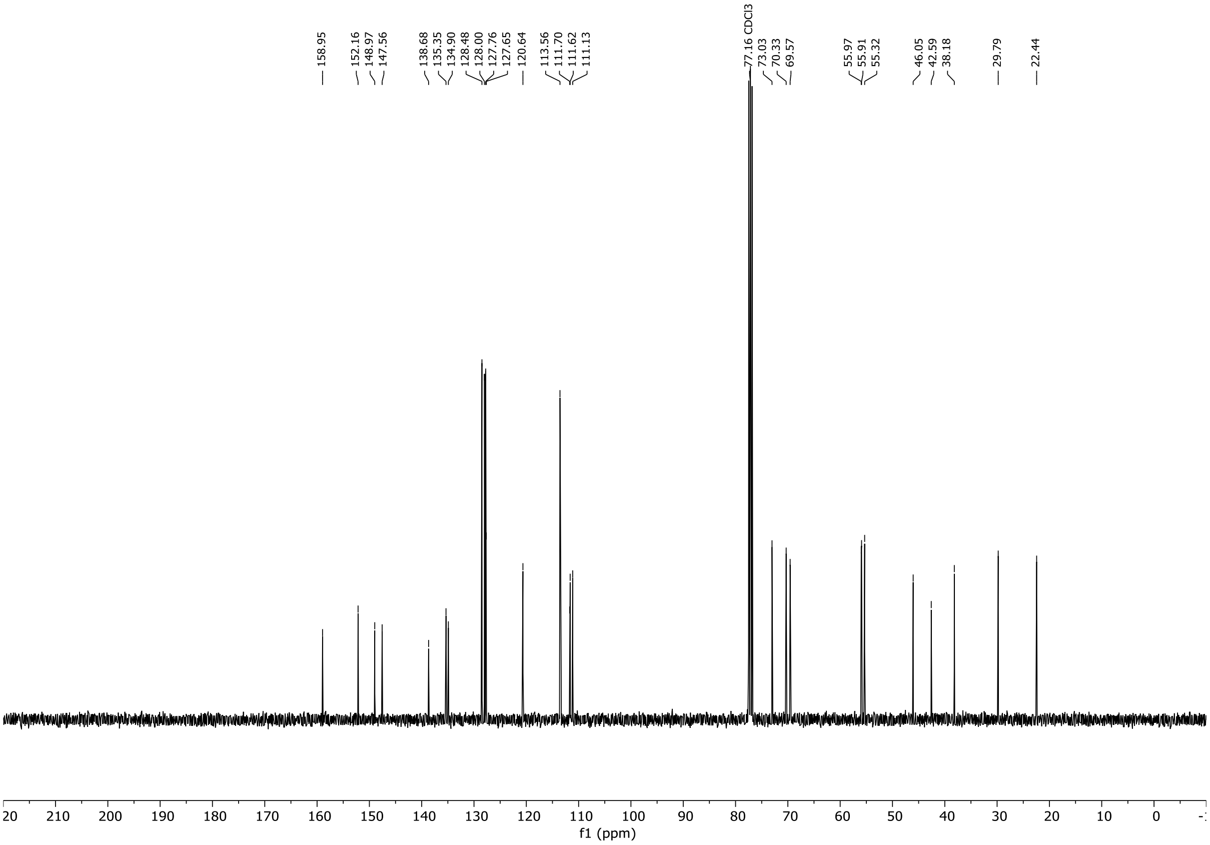

Benzyl ((3*R*,5*S*)-9-(benzyloxy)-3-(3,4-dimethoxyphenyl)-2-(4-methoxyphenyl)non-1-en-5-yl)((perfluorobenzoyl)oxy)carbamate (13)

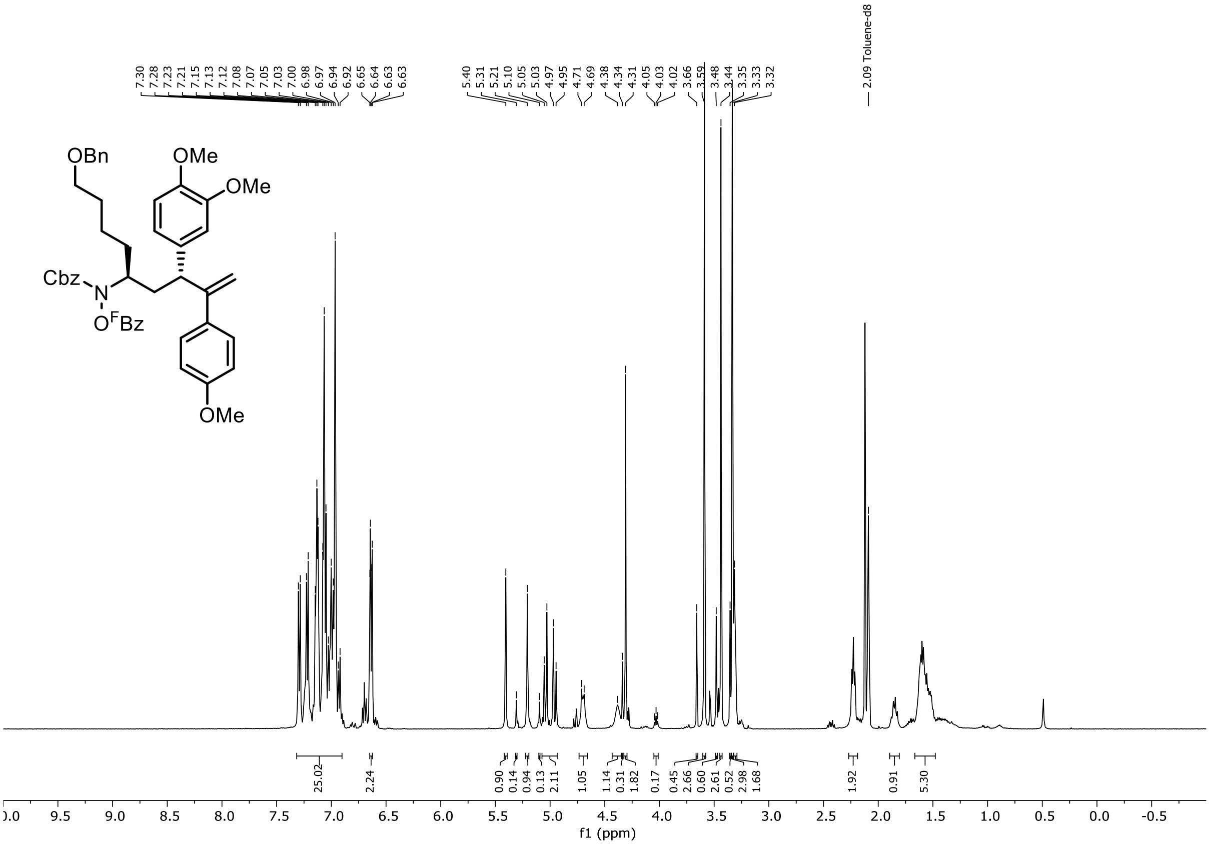

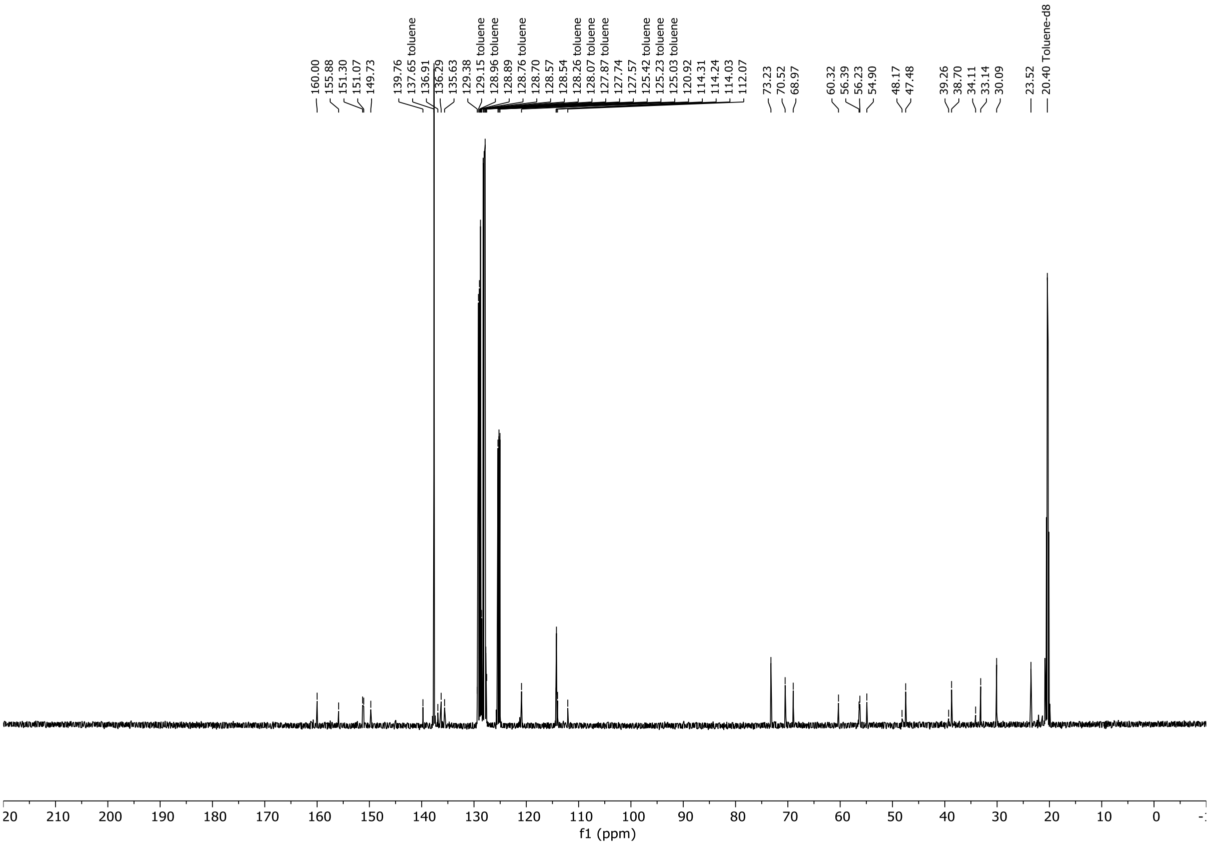

Benzyl (2*S*,3a*R*,8a*S*)-2-(4-(benzyloxy)butyl)-5,6-dimethoxy-8a-(4-methoxyphenyl)-3,3a,8,8a-tetrahydroindeno[2,1-*b*]pyrrole-1(2*H*)-carboxylate (15a)

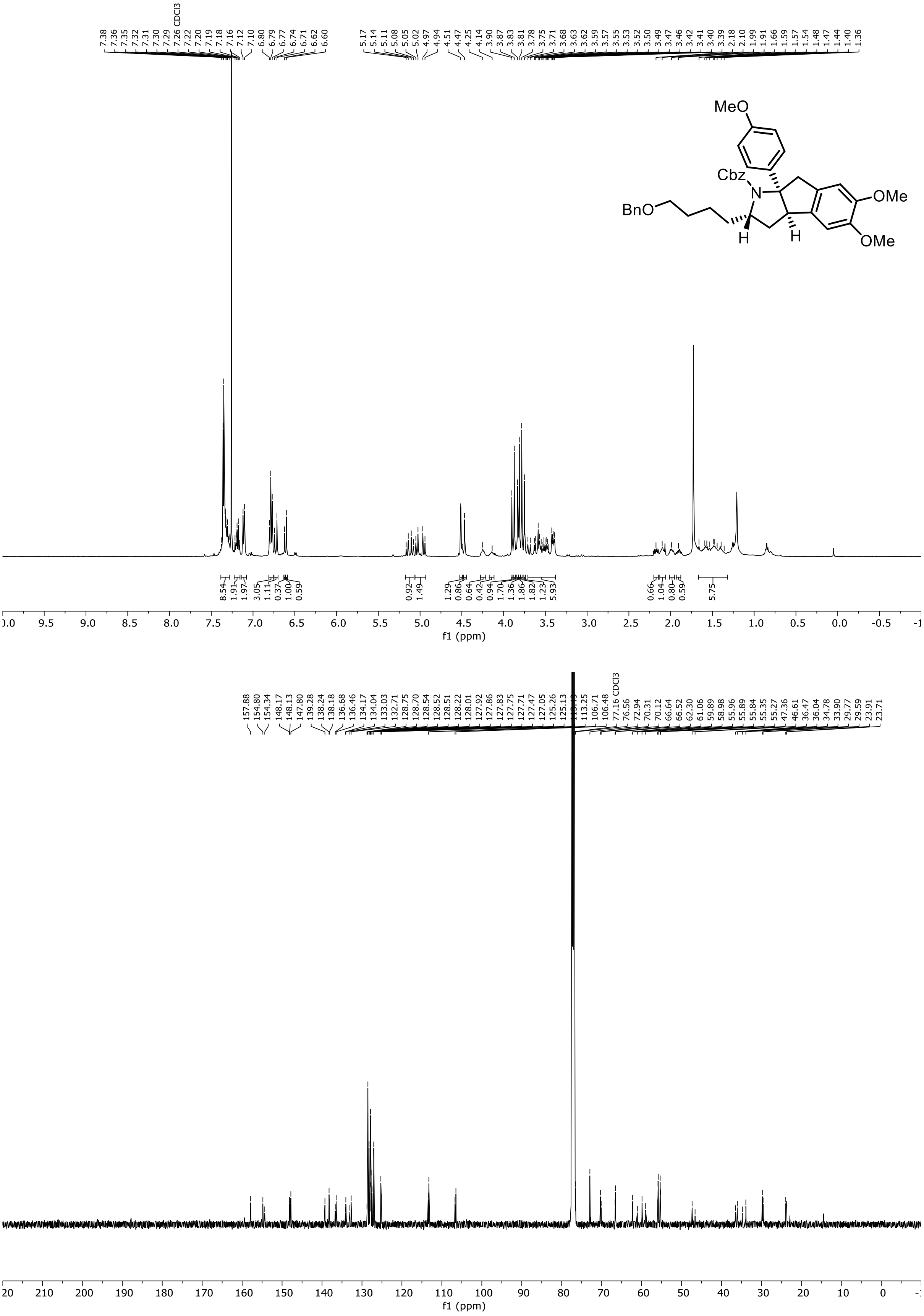

Benzyl-2-(4-(benzyloxy)butyl)-6,7-dimethoxy-8a-(4-methoxyphenyl)-3,3a,8,8a-tetrahydroindeno[2,1-*b*]pyrrole-1(2*H*)-carboxylate (15b)

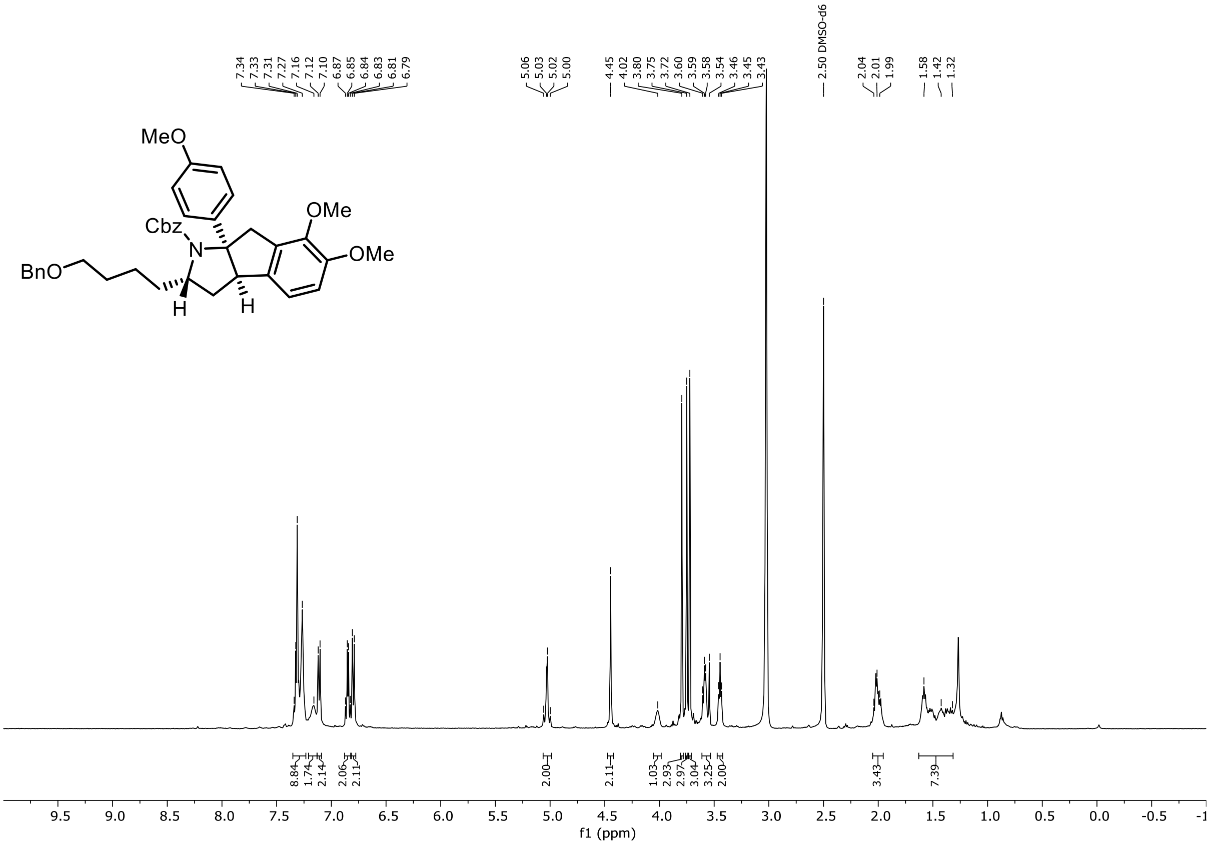

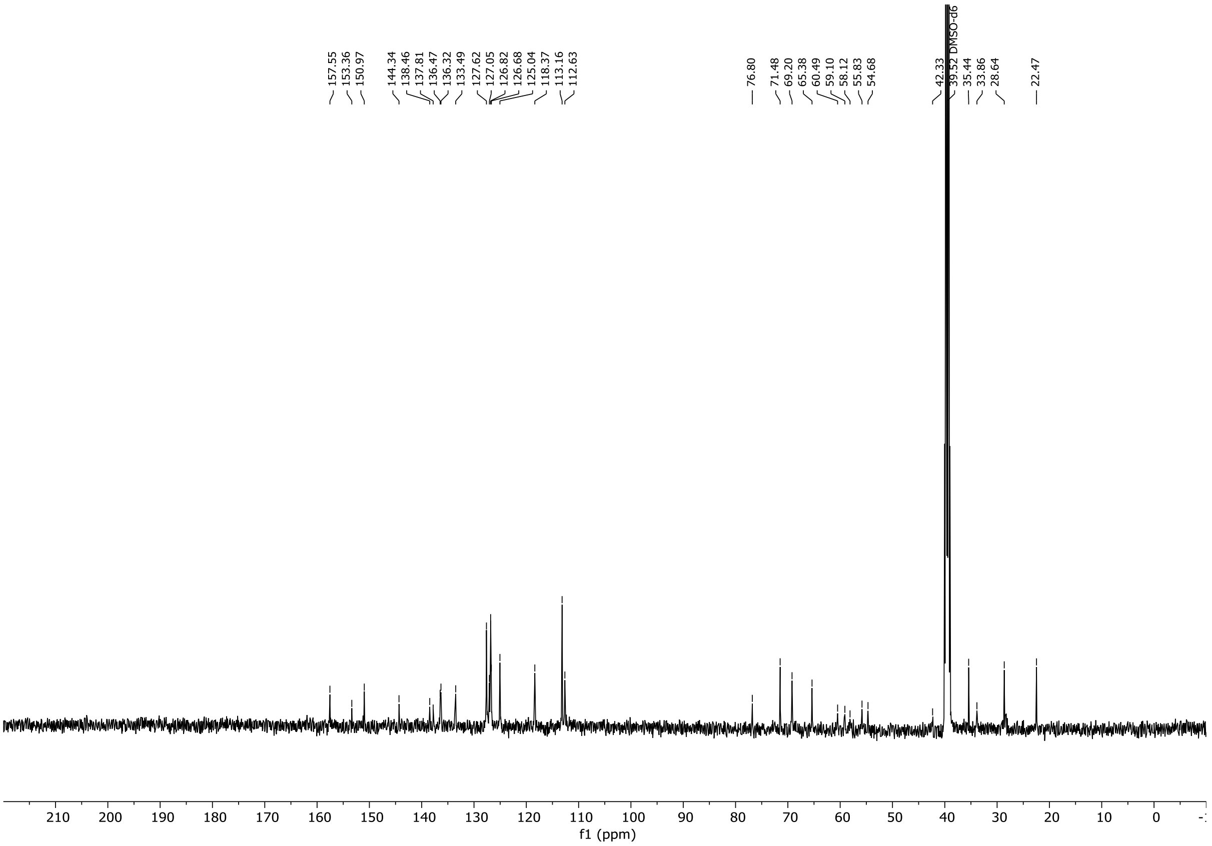

4-((2*S*,3a*R*,8a*S*)-5,6-Dimethoxy-8a-(4-methoxyphenyl)-1,2,3,3a,8,8a-hexahydroindeno[2,1-*b*]pyrrol-2-yl)butan-1-ol (16)

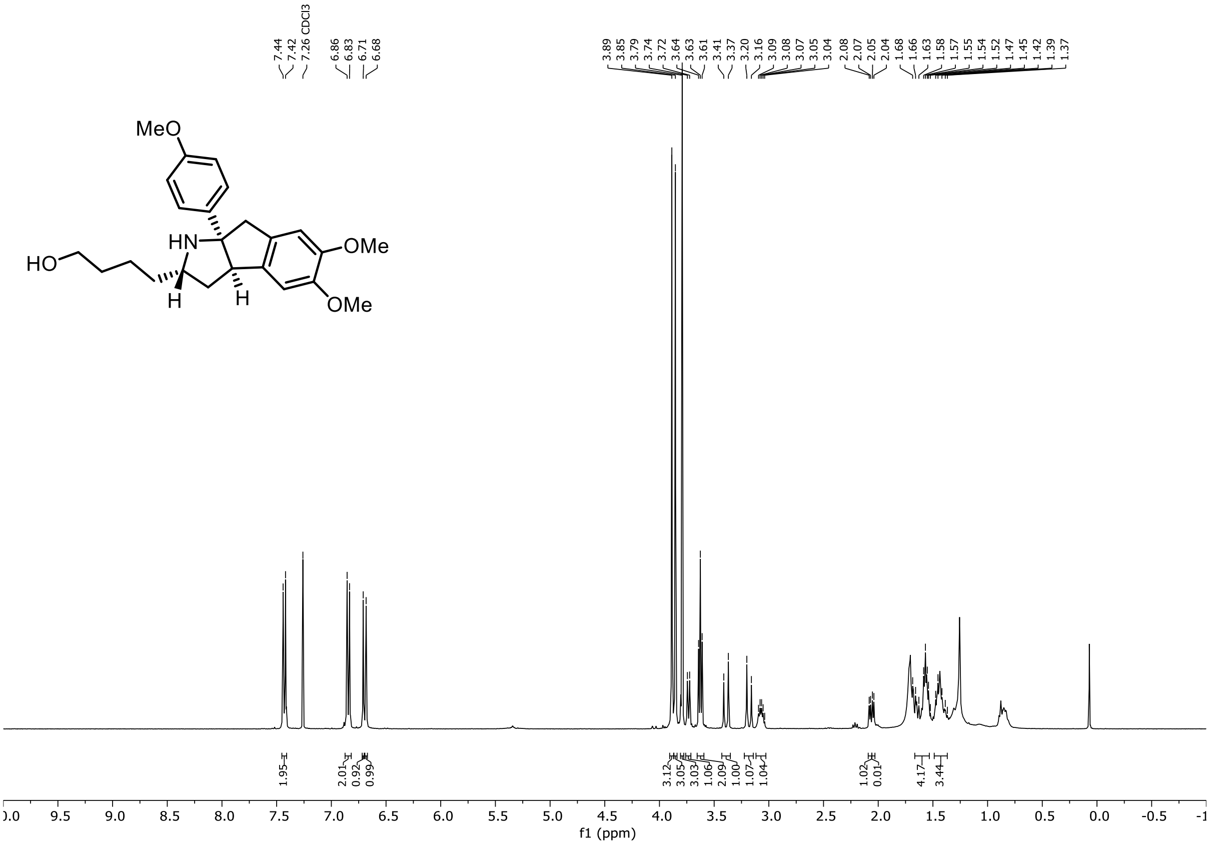

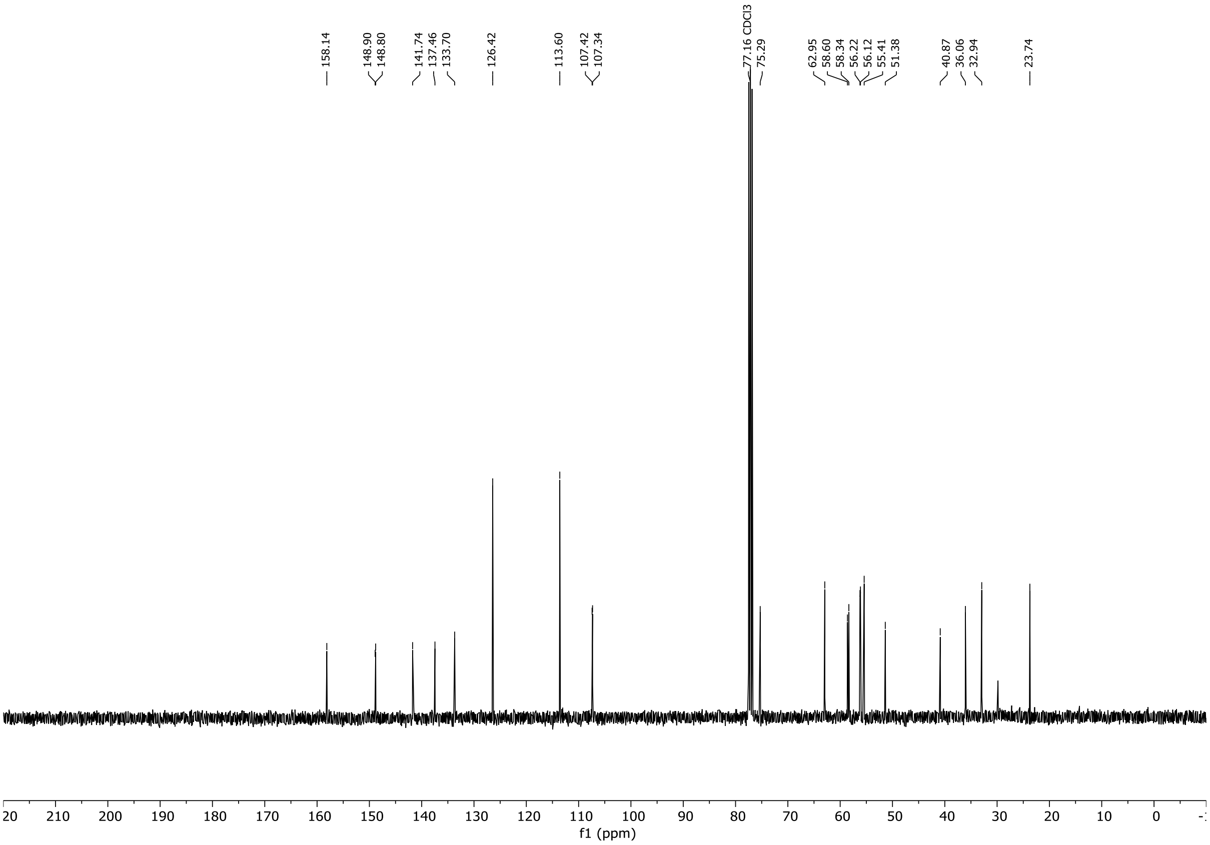

(+)-Pileamartine A (17)

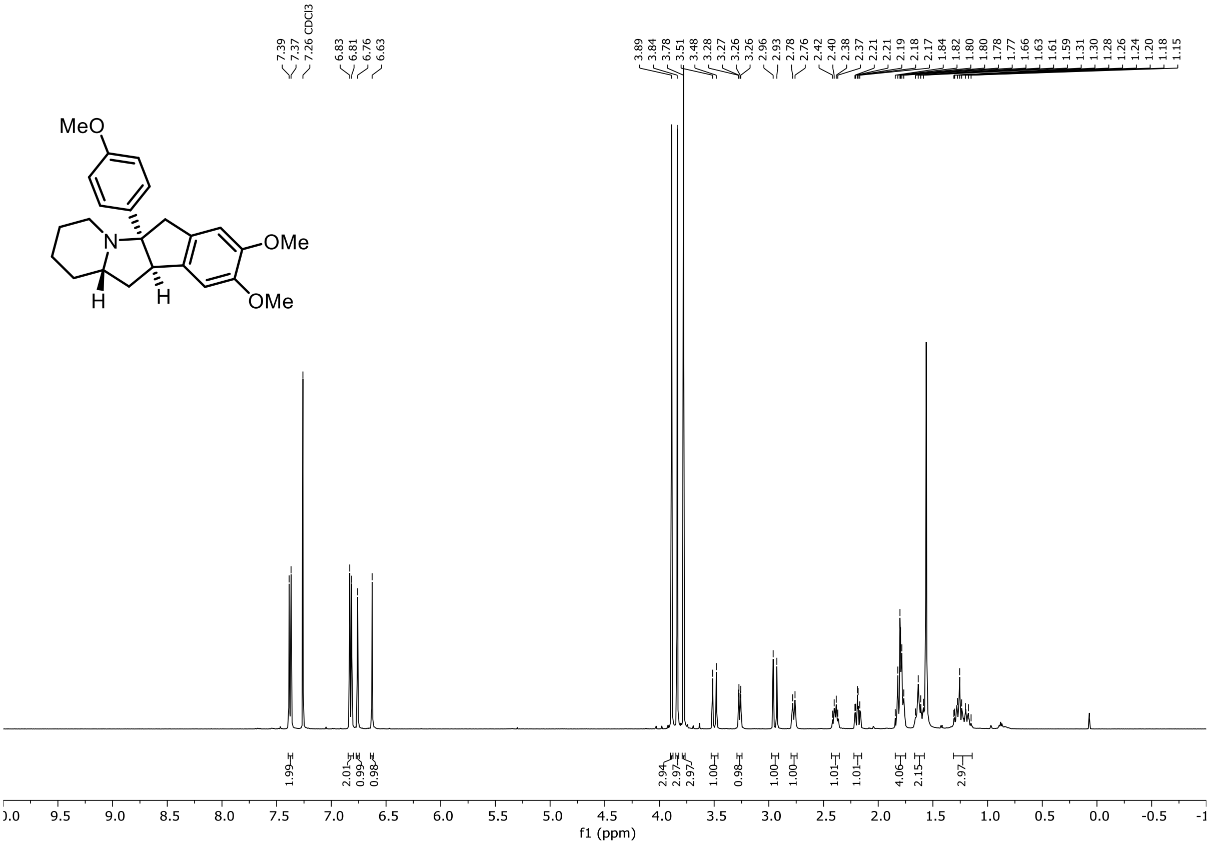

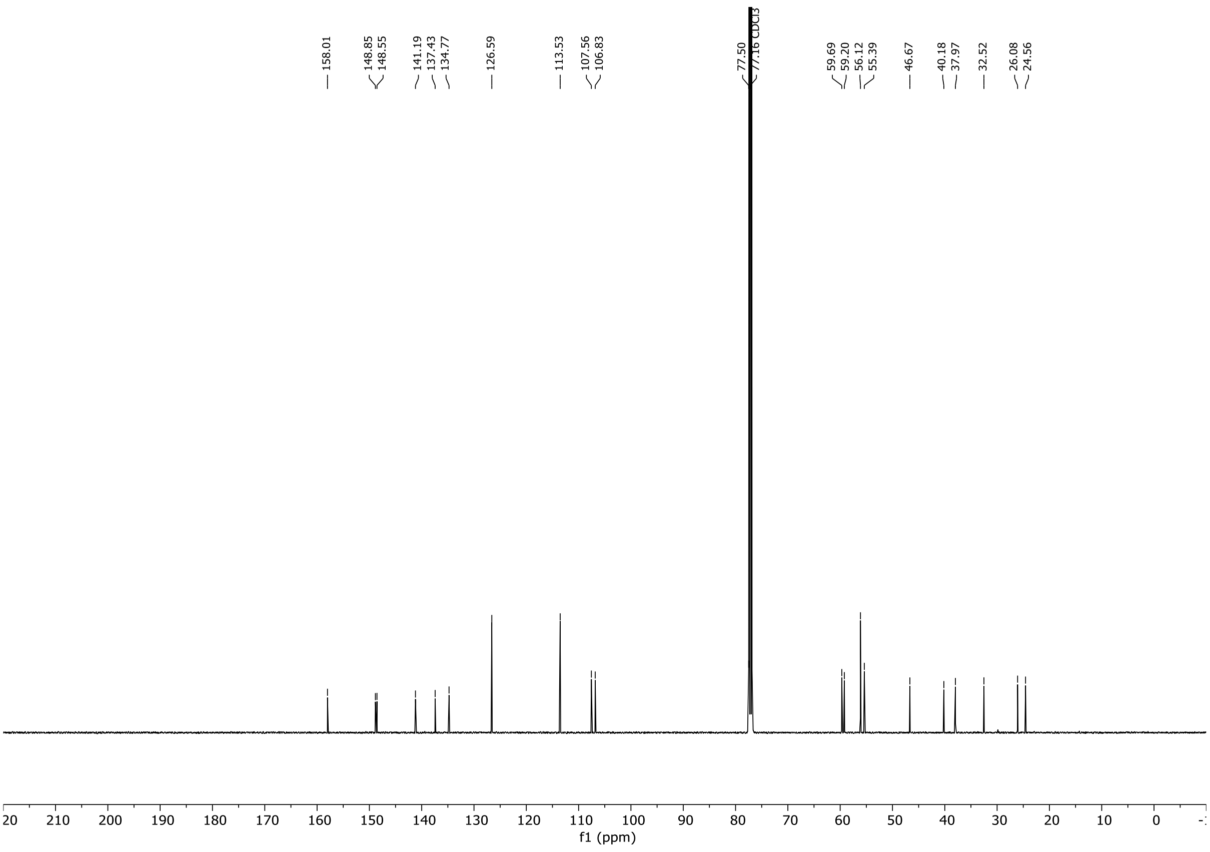

(2*S*,4*R*)-8-(Benzyloxy)-4-((tert-butyldimethylsilyl)oxy)-2-(3,4-dimethoxyphenyl)-1-(4-methoxyphenyl)octan-1-one

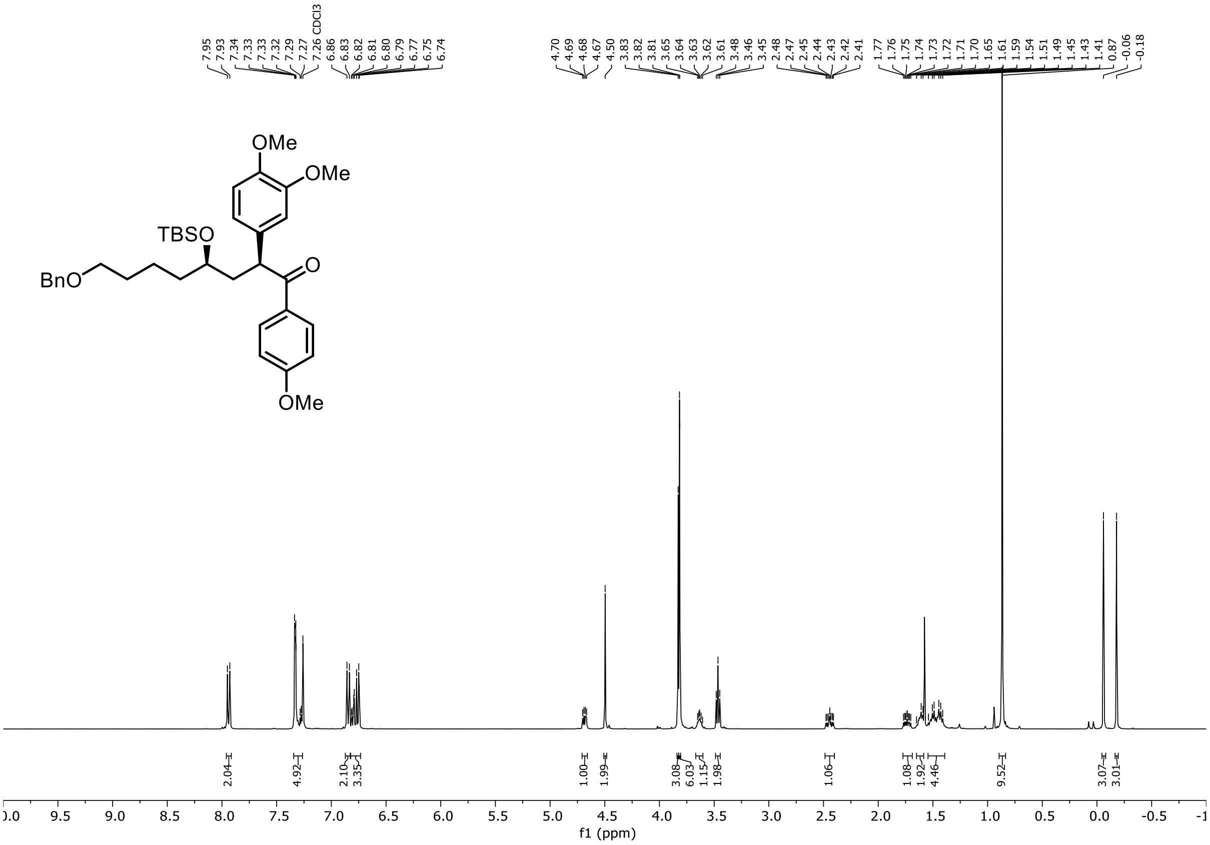

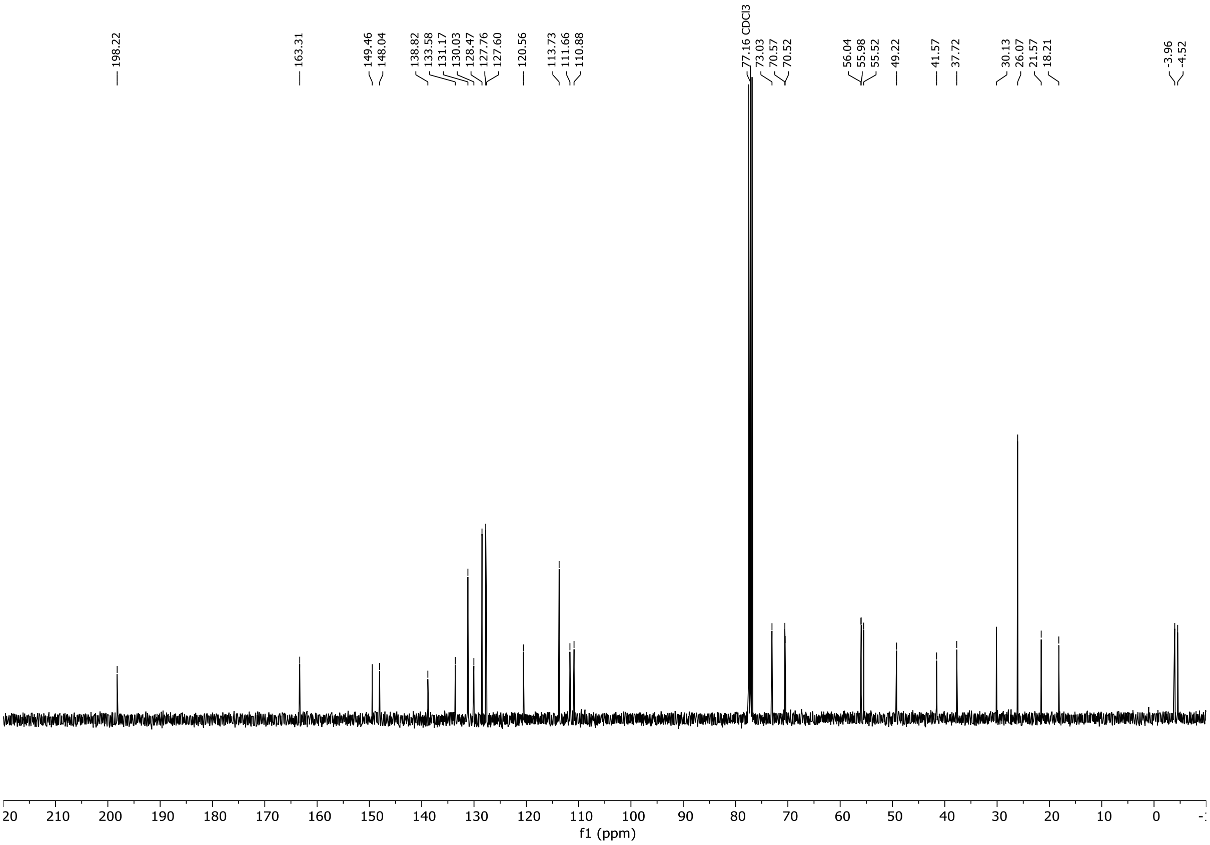

(((3*S*,5*R*)-9-(Benzyloxy)-3-(3,4-dimethoxyphenyl)-2-(4-methoxyphenyl)non-1-en-5-yl)oxy)(*tert*-butyl)dimethylsilane

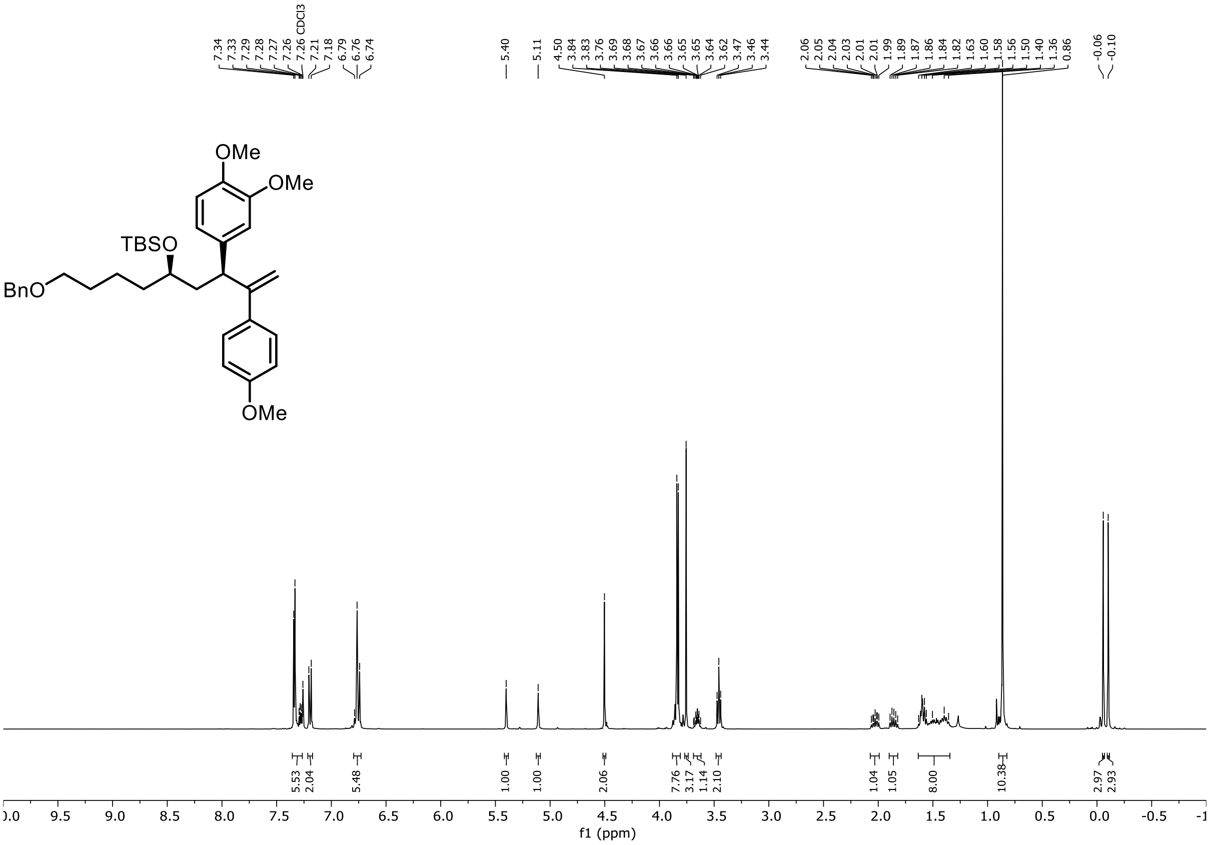

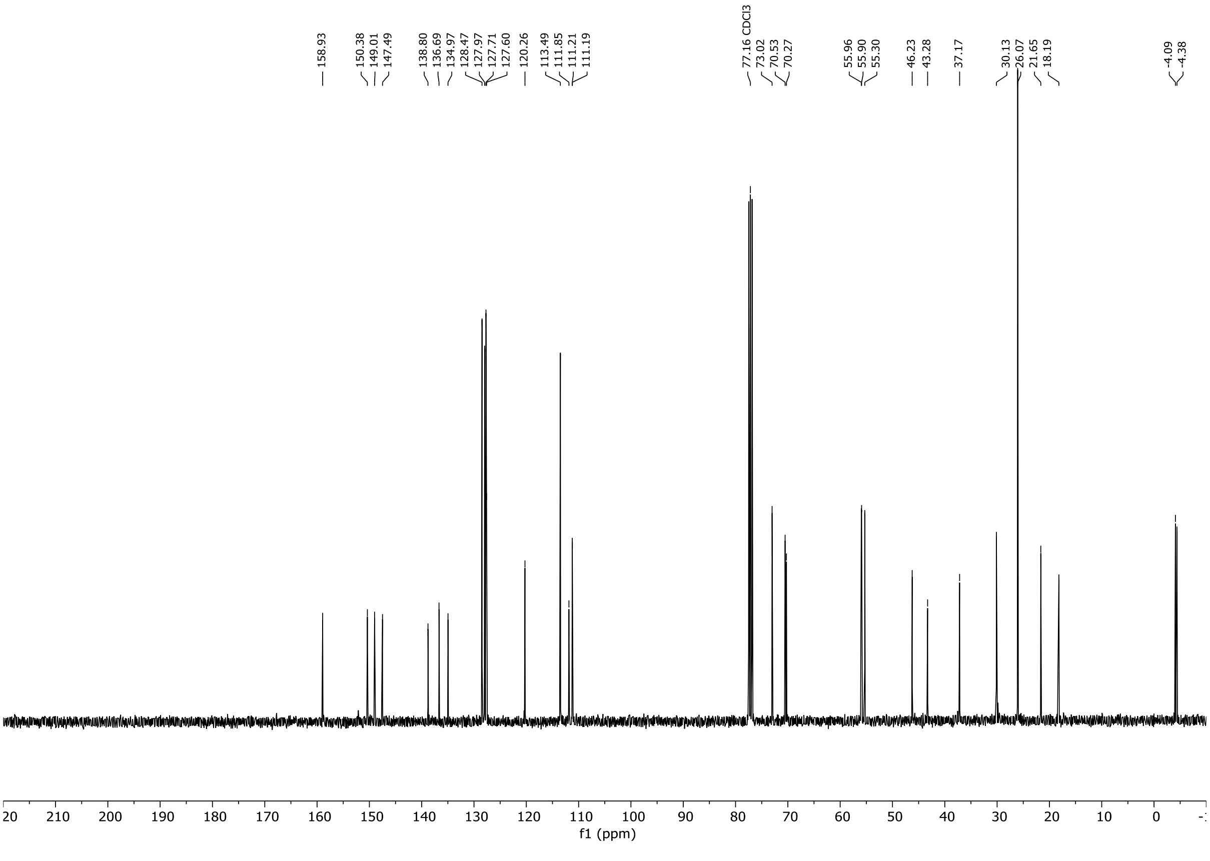

(3*S*,5*R*)-9-(Benzyloxy)-3-(3,4-dimethoxyphenyl)-2-(4-methoxyphenyl)non-1-en-5-ol (*cis*-11)

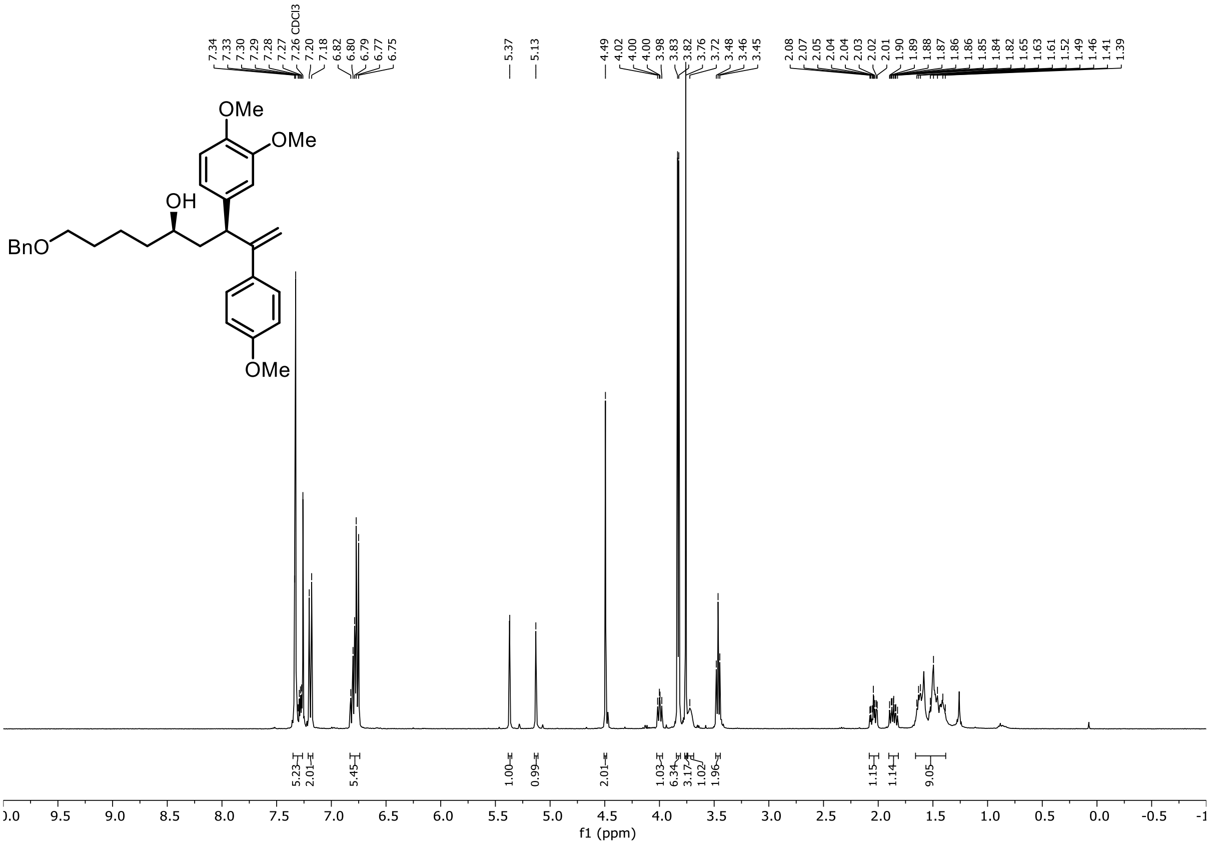

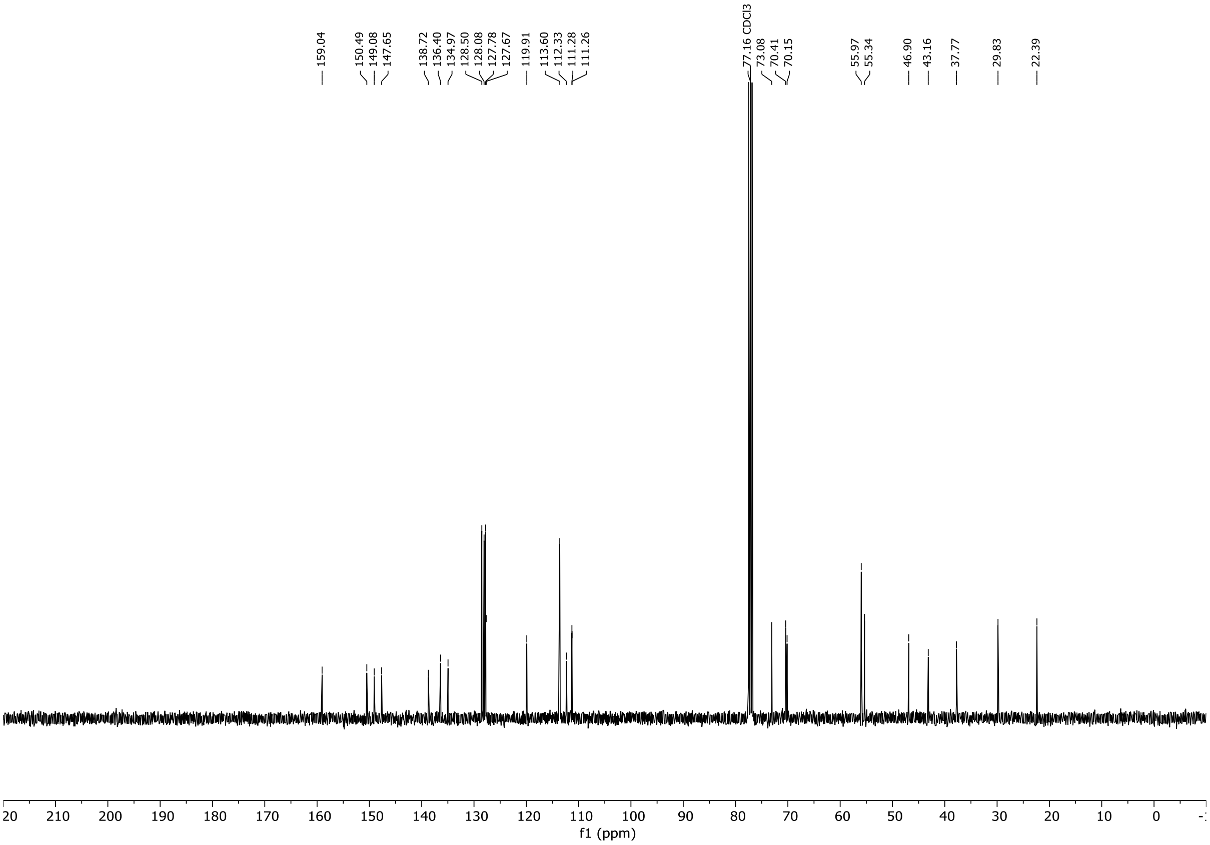

References

1. Hazelden, I. R.; Carmona, R. C.; Langer, T.; Pringle, P. G.; Bower, J. F., Pyrrolidines and Piperidines by Ligand-Enabled Aza-Heck Cyclizations and Cascades of *N*-(Pentafluorobenzoyloxy)carbamates. *Angew. Chem. Int. Ed.* **2018**, *57*, 5124-5128.
2. Lajiness, J. P.; Robertson, W. M.; Dunwiddie, I.; Broward, M. A.; Vielhauer, G. A.; Weir, S. J.; Boger, D. L., Design, Synthesis, and Evaluation of Duocarmycin *O*-Amino Phenol Prodrugs Subject to Tunable Reductive Activation. *J. Med. Chem.* **2010**, *53*, 7731-7738.
3. Porcheddu, A.; De Luca, L.; Giacomelli, G., A Straightforward Route to Piloty's Acid Derivatives: A Class of Potential Nitroxyl-Generating Prodrugs. *Synlett*, **2009**, 2149-2153.
4. Oae, S.; Shinhama, K. I.; Fujimori, K.; Kim, Y. H., Physical Properties and Various Reactions of Thionitrites and Related Substances. *Bull. Chem. Soc. Jpn.* **1980**, *53*, 775-784.
5. Hazelden, I. R.; Xiaofeng, M.; Thomas, L.; Bower, J. F., Diverse *N*-Heterocyclic Ring Systems via Aza-Heck Cyclizations of *N*-(Pentafluorobenzoyloxy)sulfonamides. *Angew. Chem. Int. Ed.* **2016**, *55*, 11198-11202.
6. Adjabeng, G.; Brenstrum, T.; Wilson, J.; Frampton, C.; Robertson, A.; Hillhouse, J.; McNulty, J.; Capretta, A., Novel Class of Tertiary Phosphine Ligands Based on a Phospha-adamantane Framework and Use in the Suzuki Cross-Coupling Reactions of Aryl Halides under Mild Conditions. *Org. Lett.* **2003**, *5*, 953-955.
7. Le, C. M.; Hou, X.; Sperger, T.; Schoenebeck, F.; Lautens, M., An Exclusively *trans*-Selective Chlorocarbamoylation of Alkynes Enabled by a Palladium/Phosphaadamantane Catalyst. *Angew. Chem. Int. Ed.* **2015**, *54*, 15897-15900.
8. Kalinowska, M.; Świsłocka, R.; Lewandowski, W., The Spectroscopic (FT-IR, FT-Raman, UV and ^{1}H, ^{13}C NMR) and Theoretical Studies of Alkali Metal *O*-Methoxybenzoates. *J. Mol. Struct.* **2006**, *792-793*, 130-138.
9. Świsłocka, R.; Samsonowicz, M.; Regulska, E.; Lewandowski, W., Theoretical and Experimental IR, Raman and NMR Spectra in Studying the Electronic Structure of 2-Nitrobenzoates. *J. Mol. Struct.* **2007**, *834-836*, 389-398.
10. Hao, J.; Gao, Y.; Li, Y.; Yan, Q.; Hu, J.; Ju, Y., Thermosensitive Triterpenoid-Appended Polymers with Broad Temperature Tunability Regulated by Host–Guest Chemistry. *Asian. J. Chem.* **2017**, *12*, 2231-2236.
11. Wang, S.-F.; Cao, X.-P.; Li, Y., Efficient Aryl Migration from an Aryl Ether to a Carboxylic Acid Group To Form an Ester by Visible-Light Photoredox Catalysis. *Angew. Chem. Int. Ed.* **2017**, *56*, 13809-13813.
12. Gonzalez-Gomez, J. C.; Ramirez, N. P.; Lana-Villarreal, T.; Bonete, P., A Photoredox-Neutral Smiles Rearrangement of 2-Aryloxybenzoic Acids. *Organic & Biomolecular Chemistry* **2017**, *15*, 9680-9684.
13. Grainger, R.; Cornella, J.; Blakemore, D. C.; Larrosa, I.; Campanera, J. M., The *ortho*-Substituent Effect on the Ag-Catalysed Decarboxylation of Benzoic Acids. *Chem. Eur. J.* **2014**, *20*, 16680-16687.
14. Betz, J.; Bauer, W., NMR and Calculational Studies on the Regioselective Lithiation of 1-Methoxynaphthalene. *J. Am. Chem. Soc.* **2002**, *124*, 8699-8706.
15. Yip, K.-T.; Yang, D., Pd(II)-Catalyzed Intramolecular Amidoarylation of Alkenes with Molecular Oxygen as Sole Oxidant. *Org. Lett.* **2011**, *13*, 2134-2137.
16. Zhu, R.; Buchwald, S. L., Combined Oxypalladation/C-H Functionalization: Palladium(II)-Catalyzed Intramolecular Oxidative Oxyarylation of Hydroxyalkenes. *Angew. Chem. Int. Ed.* **2012**, *51*, 1926-1929.
17. Süsse, L.; Vogler, M.; Mewald, M.; Kemper, B.; Irran, E.; Oestreich, M., Enantioselective Nazarov Cyclizations Catalyzed by an Axial Chiral C_6F_5-Substituted Boron Lewis Acid. *Angew. Chem. Int. Ed.* **2018**, *57*, 11441-11444.
18. Wang, Q.; Liu, X.; Liu, X.; Li, B.; Nie, H.; Zhang, S.; Chen, W., Highly Enantioselective Hydrogenation of 2-Substituted-2-Alkenols Catalysed by a ChenPhos–Rh Complex. *Chem. Commun.* **2014**, *50*, 978-980.

19. Carpenter, M. S.; Easter Jr., W. M. 4-*tert*. Butyl α-Methyl Hydrocinnamic Aldehyde. US2875131, 24th February, 1959.
20. Zhang, X.-L.; Pan, G.-F.; Zhu, X.-Q.; Guo, R.-L.; Gao, Y.-R.; Wang, Y.-Q., Dehydrogenative β-Arylation of Saturated Aldehydes Using Transient Directing Groups. *Org. Lett.* **2019**, *21*, 2731-2735.
21. Henrot, M.; Richter, M. E. A.; Maddaluno, J.; Hertweck, C.; De Paolis, M., Convergent Asymmetric Synthesis of (+)-Aureothin Employing an Oxygenase-Mediated Resolution Step. *Angew. Chem. Int. Ed.* **2012**, *51*, 9587-9591.
22. Bérubé, C.; Carpentier, C.; Voyer, N., Total Synthesis of Chrysamide B. *Tetrahedron Lett.* **2017**, *58*, 2334-2336.
23. Eisch, J. J.; Aradi, A. A.; Han, K. I., Nickel(0)-Mediated Hydrocyanation and Carbonylation Reactions of Alkynes with Trimethylsilyl(iso)cyanide. *Tetrahedron Lett.* **1983**, *24*, 2073-2076.
24. Zhang, Z.; Wang, Q.; Chen, C.; Han, Z.; Dong, X.-Q.; Zhang, X., Selective Rhodium-Catalyzed Hydroformylation of Alkynes to α,β-Unsaturated Aldehydes with a Tetraphosphoramidite Ligand. *Org. Lett.* **2016**, *18*, 3290-3293.
25. Unroe, M. R.; Reinhardt, B. A., One Pot Synthesis of *p*-Polyphenyls *via* the lntramolecular Cyclization of 3-Dimethyl-aminohex-5-en-1-ynes. *Synthesis*, **1987**, *1987*, 981-986.
26. Wang, M.-Y.; Jin, X.; Wang, X.; Xia, S.; Wang, Y.; Huang, S.; Li, Y.; He, L.-N.; Ma, X., Copper-Catalyzed and Proton-Directed Selective Hydroxymethylation of Alkynes with CO_2. *Angew. Chem. Int. Ed.* **2021**, *60*, 3984-3988.
27. Emer, E.; Pfeifer, L.; Brown, J. M.; Gouverneur, V., *cis*-Specific Hydrofluorination of Alkenylarenes under Palladium Catalysis through an Ionic Pathway. *Angew. Chem. Int. Ed.* **2014**, *53*, 4181-4185.
28. Jobashi, T.; Kawai, A.; Kawai, S.; Maeyama, K.; Oike, H.; Yoshida, Y.; Yonezawa, N., Intramolecular Electrophilic Aromatic Substitution of α-Alkylcinnamaldehydes Affording 1-Alkoxy-2-Alkylindenes. *Tetrahedron* **2006**, *62*, 5717-5724.
29. Rupnicki, L.; Saxena, A.; Lam, H. W., Aromatic Heterocycles as Activating Groups for Asymmetric Conjugate Addition Reactions. Enantioselective Copper-Catalyzed Reduction of 2-Alkenylheteroarenes. *J. Am. Chem. Soc.* **2009**, *131*, 10386-10387.
30. Spoehrle, S. S. M.; West, T. H.; Taylor, J. E.; Slawin, A. M. Z.; Smith, A. D., Tandem Palladium and Isothiourea Relay Catalysis: Enantioselective Synthesis of α-Amino Acid Derivatives via Allylic Amination and [2,3]-Sigmatropic Rearrangement. *J. Am. Chem. Soc.* **2017**, *139*, 11895-11902.
31. Ma, X.; Hazelden, I. R.; Langer, T.; Munday, R. H.; Bower, J. F., Enantioselective Aza-Heck Cyclizations of *N*-(Tosyloxy)carbamates: Synthesis of Pyrrolidines and Piperidines. *J. Am. Chem. Soc.* **2019**, *141*, 3356-3360.
32. Farndon, J. J.; Young, T. A.; Bower, J. F., Stereospecific Alkene Aziridination Using a Bifunctional Amino-Reagent: An Aza-Prilezhaev Reaction. *J. Am. Chem. Soc.* **2018**, *140*, 17846-17850.
33. Schaubach, S.; Gebauer, K.; Ungeheuer, F.; Hoffmeister, L.; Ilg, M. K.; Wirtz, C.; Fürstner, A., A Two-Component Alkyne Metathesis Catalyst System with an Improved Substrate Scope and Functional Group Tolerance: Development and Applications to Natural Product Synthesis. *Chem. Eur. J.* **2016**, *22*, 8494-8507.
34. Kona, C. N.; Ramana, C. V., Gold(I)-Catalysed [1,3] O→C Rearrangement of Allenyl Ethers. *Chem. Commun.* **2014**, *50*, 2152-2154.
35. Tamura, O.; Shiro, T.; Ogasawara, M.; Toyao, A.; Ishibashi, H., Stereoselective Syntheses of 4-Hydroxy 4-Substituted Glutamic Acids. *J. Org. Chem.* **2005**, *70*, 4569-4577.
36. Frost, C. G.; Hartley, B. C., Lewis Base-Promoted Hydrosilylation of Cyclic Malonates: Synthesis of β-Substituted Aldehydes and γ-Substituted Amines. *J. Org. Chem.* **2009**, *74*, 3599-3602.
37. Guo, C.-S.; Du, Y.-H.; Huang, Z.-Z., A Cascade Heck–Aldol–Heck Reaction by a Combination of Transition-Metal Catalysis and Aminocatalysis. *Chem. Commun.* **2011**, *47*, 3995-3997.

38. Ersoy, O.; Fleck, R.; Blanco, M.-J.; Masamune, S., Design and Syntheses of Three Haptens to Generate Catalytic Antibodies that Cleave Amide Bonds with Nucleophilic Catalysis. *Biorg. Med. Chem.* **1999**, *7*, 279-286.
39. Chen, L.-Y.; Chen, J.-R.; Cheng, H.-G.; Lu, L.-Q.; Xiao, W.-J., Enantioselective Synthesis of Tetrahydrofuran Derivatives by Sequential Henry Reaction and Iodocyclization of γ,δ-Unsaturated Alcohols. *Eur. J. Org. Chem.* **2014**, 4714-4719.
40. Chen, G.; Shigenari, T.; Jain, P.; Zhang, Z.; Jin, Z.; He, J.; Li, S.; Mapelli, C.; Miller, M. M.; Poss, M. A.; Scola, P. M.; Yeung, K.-S.; Yu, J.-Q., Ligand-Enabled β-C–H Arylation of α-Amino Acids Using a Simple and Practical Auxiliary. *J. Am. Chem. Soc.* **2015**, *137*, 3338-3351.
41. Pérez, M.; Pérez, D. I.; Martínez, A.; Castro, A.; Gómez, G.; Fall, Y., The First Enantioselective Synthesis of Palinurin. *Chem. Commun.* **2009**, *22*, 3252-3254.
42. Mei, G.; Liu, X.; Qiao, C.; Chen, W.; Li, C.-C., Type II Intramolecular [5+2] Cycloaddition: Facile Synthesis of Highly Functionalized Bridged Ring Systems. *Angew. Chem. Int. Ed.* **2015**, *54*, 1754-1758.
43. Ho, G.-M.; Judkele, L.; Bruffaerts, J.; Marek, I., Metal-Catalyzed Remote Functionalization of ω-Ene Unsaturated Ethers: Towards Functionalized Vinyl Species. *Angew. Chem. Int. Ed.* **2018**, *57*, 8012-8016.
44. Mountanea, O. G.; Limnios, D.; Kokotou, M. G.; Bourboula, A.; Kokotos, G., Asymmetric Synthesis of Saturated Hydroxy Fatty Acids and Fatty Acid Esters of Hydroxy Fatty Acids. *Eur. J. Org. Chem.* **2019**, 2010-2019.
45. Fabian, L.; Gómez, M.; Caturelli Kuran, J. A.; Moltrasio, G.; Moglioni, A., Efficient Microwave-Assisted Esterification Reaction Employing Methanesulfonic Acid Supported on Alumina as Catalyst. *Synth. Commun.* **2014**, *44*, 2386-2392.
46. Clarke, M. L.; Ellis, D.; Mason, K. L.; Orpen, A. G.; Pringle, P. G.; Wingad, R. L.; Zaher, D. A.; Baker, R. T., The Electron-Poor Phosphines $P\{C_6H_3(CF_3)_2\text{-}3,5\}_3$ and $P(C_6F_5)_3$ do not Mimic Phosphites as Ligands for Hydroformylation. A Comparison of the Coordination Chemistry of $P\{C_6H_3(CF_3)_2\text{-}3,5\}_3$ and $P(C_6F_5)_3$ and the Unexpectedly Low Hydroformylation Activity of their Rhodium Complexes. *Dalton Transactions* **2005**, 1294-1300.
47. Thuy, A. D. T.; Thanh, V. T. T.; Mai, H. D. T.; Le, H. T.; Litaudon, M.; Chau, V. M.; Pham, V. C., Pileamartines A and B: Alkaloids from Pilea Aff. Martinii with a New Carbon Skeleton. *Tetrahedron Lett.* **2018**, *59*, 1909-1912.
48. Faber, L.; Wiegrebe, W., Stereospezifische Synthese Zweier 9, 11, 12, 13, 13a, 14-Hexahydrodibenzo[*f,h*]pyrrolo[1, 2-*b*]isochinoline. *Helv. Chim. Acta* **1976**, *59*, 2201-2212.
49. Gupton, J. T.; Shimozono, A.; Crawford, E.; Ortolani, J.; Clark, E.; Mahoney, M.; Heese, C.; Noble, J.; Mandry, C. P.; Kanters, R.; Dominey, R. N.; Goldman, E. W.; Sikorski, J. A.; Fisher, D. C., Further Studies on the Application of Vinylogous Amides and β-Halovinylaldehydes to the Regiospecific Synthesis of Unsymmetrical, Polyfunctionalized 2,3,4- and 1,2,3,4-Substituted Pyrroles. *Tetrahedron* **2018**, *74*, 2650-2663.
50. Suhara, Y.; Oka, S.; Kittaka, A.; Takayama, H.; Waku, K.; Sugiura, T., Synthesis and Biological Evaluation of Several Structural Analogs of 2-Arachidonoylglycerol, an Endogenous Cannabinoid Receptor Ligand. *Biorg. Med. Chem.* **2007**, *15*, 854-867.
51. *Gaussian 16*, Revision B.01; Frisch, M. J.; Trucks, G. W.; Schlegel, H. B.; Scuseria, G. E.; Robb, M. A.; Cheeseman, J. R.; Scalmani, G.; Barone, V.; Petersson, G. A.; Nakatsuji, H.; Li, X.; Caricato, M.; Marenich, A. V.; Bloino, J.; Janesko, B. G.; Gomperts, R.; Mennucci, B.; Hratchian, H. P.; Ortiz, J. V.; Izmaylov, A. F.; Sonnenberg, J. L.; Williams-Young, D.; Ding, F.; Lipparini, F.; Egidi, F.; Goings, J.; Peng, B.; Petrone, A.; Henderson, T.; Ranasinghe, D.; Zakrzewski, V. G.; Gao, J.; Rega, N.; Zheng, G.; Liang, W.; Hada, M.; Ehara, M.; Toyota, K.; Fukuda, R.; Hasegawa, J.; Ishida, M.; Nakajima, T.; Honda, Y.; Kitao, O.; Nakai, H.; Vreven, T.; Throssell, K.; Montgomery, J., J. A.; Peralta, J. E.; Ogliaro, F.; Bearpark, M. J.; Heyd, J. J.; Brothers, E. N.; Kudin, K. N.; Staroverov, V. N.; Keith, T. A.; Kobayashi, R.; Normand, J.; Raghavachari, K.; Rendell, A. P.; Burant, J. C.; Iyengar, S. S.; Tomasi, J.; Cossi, M.; Millam, J. M.; Klene, M.; Adamo, C.; Cammi, R.; Ochterski, J. W.; Martin, R. L.; Morokuma, K.; Farkas, O.; Foresman, J. B.; Fox, D. J., Gaussian, Inc., Wallingford CT, 2016.
